# Unified Synthesis of Polycyclic Alkaloids by Complementary Carbonyl Activation**

**DOI:** 10.1002/anie.202102518

**Published:** 2021-05-01

**Authors:** Guoli He, Benjamin List, Mathias Christmann

**Affiliations:** ^1^ Freie Universität Berlin Institute of Chemistry and Biochemistry Takustrasse 3 14195 Berlin Germany; ^2^ Max-Planck-Institut für Kohlenforschung Kaiser-Wilhelm-Platz 1 45470 Mülheim an der Ruhr Germany

**Keywords:** disulfonimides, domino reactions, organocatalysis, polycyclic alkaloids, total synthesis

## Abstract

A complementary dual carbonyl activation strategy for the synthesis of polycyclic alkaloids has been developed. Successful applications include the synthesis of tetracyclic alkaloids harmalanine and harmalacinine, pentacyclic indoloquinolizidine alkaloid nortetoyobyrine, and octacyclic β‐carboline alkaloid peganumine A. The latter synthesis features a protecting‐group‐free assembly and an asymmetric disulfonimide‐catalyzed cyclization. Furthermore, formal syntheses of hirsutine, deplancheine, 10‐desbromoarborescidine A, and oxindole alkaloids rhynchophylline and isorhynchophylline have been achieved. Finally, a concise synthesis of berberine alkaloid ilicifoline B was completed.

## Graphical abstract

The efficient construction of polycyclic scaffolds of bioactive alkaloids by a novel annulation strategy enabled the synthesis of several natural products, including the dimeric berberine alkaloid ilicifoline B (see scheme). This versatile approach also served as the basis for a protecting‐group‐free synthesis of (+)‐ and (−)‐peganumine A featuring an asymmetric disulfonimide‐catalyzed cyclization cascade.

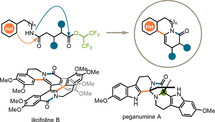

Despite the advancement of combinatorial strategies, natural products remain an indispensable source for the discovery of new molecular entities.^[1]^ Their diverse scaffolds with hydrogen bond donor and acceptor groups positioned in a well‐defined spatial arrangement make them attractive starting points and inspiration for drug development.^[2]^ Bioactive polycyclic alkaloids, such as yohimbine (**1**), hirsutine (**2**), deplancheine (**3**), eburnamonine (**4**), ilicifoline B (**5**), peganumine A (**6**), and reserpine (**7**), contain the common quinolizidine core **I** fused to different heterocyclic rings (Figure 1). We reasoned that developing a straightforward annulation method for efficient construction of these scaffolds is beneficial for the total synthesis of polycyclic natural products and their analogs. Since the indole substructure is a privileged^[3]^ and very common motif in these polycyclic natural products, we started our synthetic journey with the quinolizidine‐fused indole core. We strategized that incorporating an enamide motif into the A ring would provide a flexible handle for subsequent transformations. Therefore, intermediate **III** ^[4]^ was considered the central linchpin for a divergent synthesis^[5]^ of polycyclic alkaloids. It was envisioned to be derived from **IV** by an annulation sequence involving an electrophilic cyclization followed by lactamization. Toward this goal we identified two major challenges: 1) selective activation of the amide carbonyl group to participate in the electrophilic cyclization;^[6]^ 2) subsequent selective activation of the second carbonyl group to achieve lactamization.

**Figure 1:**
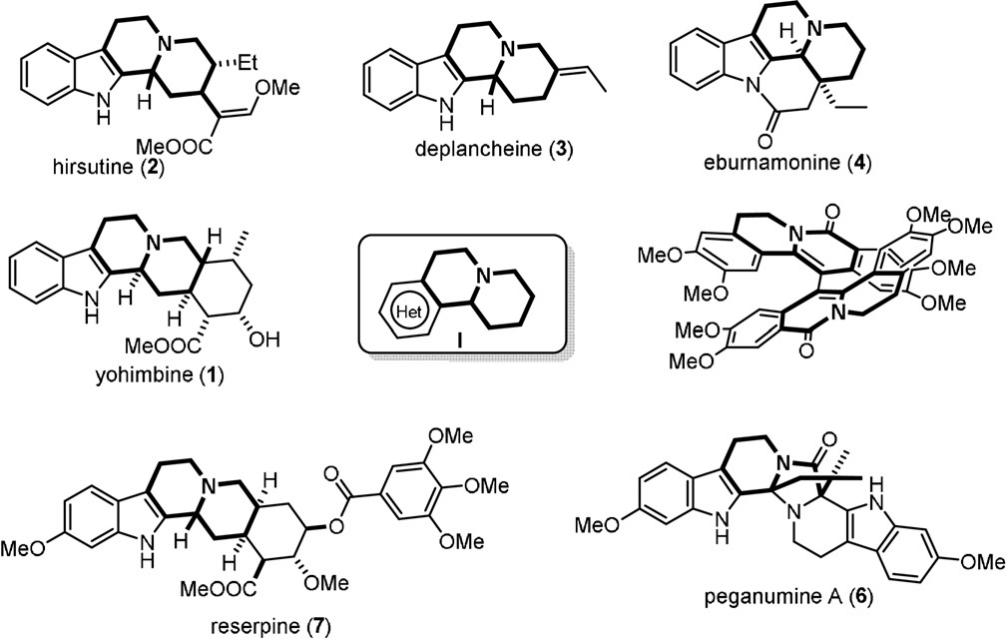
Common alkaloids with a tetracyclic substructure.

We conceived a one‐pot approach in which **IV** can be prepared by two‐fold condensation of **V**, **VI** and active ester component. By permutation of the substrates **V** and **VI**, a variety of polycyclic ring system with diverse substitution patterns could be accessed (Scheme 1).

**Scheme 1:**
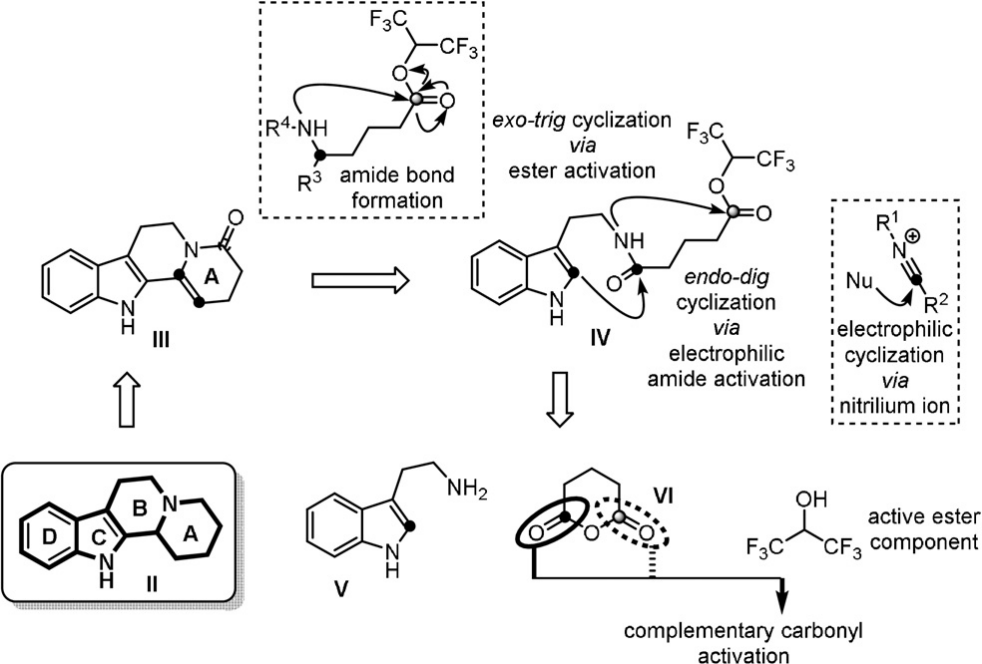
Synthetic strategy.

In order to orchestrate the subsequent activations, we initially investigated the reaction using the amide **8 a** as the model substrate (for its preparation see the Supporting Information). We tested different conditions to achieve selective amide activation via reactive nitrilium ions,^[3]^ including a variety of phosphorus(V) reagents frequently applied in the Bischler–Napieralski reaction^[7]^ and the von Braun amide degradation (Table 1, entries 1–4).^[8]^ We also screened strong electrophiles, such as Me_3_SiCl and (COCl)_2_,^[9]^ and the Tf_2_O/2‐chloropyridine system^[10]^ which have been used successfully in electrophilic amide activation recently (Table 1, entries 5–7). Most of these conditions afforded tricyclic imine **9 a** as the major product. Unfortunately, the subsequent imine acylation to give tetracyclic product turned out to be challenging. With POCl_3_, **10** was isolated in 6 % yield along with 84 % of imine **9 a** (Table 1, entry 1). This result indicated that the reaction had stopped after the first cyclization. We hypothesized that imine–enamine tautomerization during second cyclization and the leaving ability of the alkoxyl group could also be the critical prerequisites for the second cyclization.^[11]^ After a screening of bases (see the Supporting Information), we achieved a slight improvement to 10 % yield of **10** using K_2_CO_3_ (Table 1, entry 8). With *n*Bu_4_NBr as phase transfer catalyst and methanol, the yield of **10** was further increased to 18 % (Table 1, entry 9). Inspired by active ester activation strategies used in peptide synthesis,^[12]^ we tested a variety of ester derivatives (see the Supporting Information). Satisfyingly, with 1,1,1,3,3,3‐hexafluoro‐2‐propoxy ester **8 d**, we achieved a 90 % yield of **10** (Table 1, entry 12).

**Table 1:** Optimization of reaction conditions. 
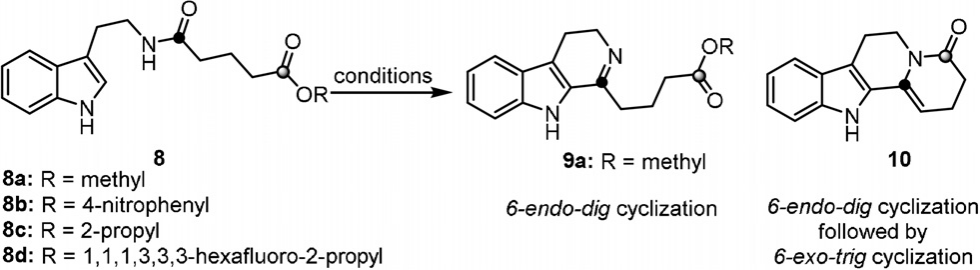

Entry^[a]^	**8**	Amide activation reagent	Solvent	*T* [°C]	Yield [%]^[b]^
1	**8 a**	POCl_3_	toluene	110	**9 a** (84) **10** (6)
2	**8 a**	P_2_O_5_	toluene	110	**9 a** (8) **10** (4)
3	**8 a**	T_3_P^[c]^	toluene	110	**9 a** (3)
4	**8 a**	PCl_5_	toluene	110	**9 a** (12) **10** (7)
5	**8 a**	TMSCl	THF	60	N.R.
6	**8 a**	(COCl)_2_	DCM	23	N.D.
7^[d]^	**8 a**	Tf_2_O	DCM	23	**9 a** (53)
8^[e]^	**8 a**	POCl_3_	toluene	110	**10** (10)
9^[f]^	**8 a**	POCl_3_	toluene/MeOH	110 to 80	**10** (18)
10^[f]^	**8 b**	POCl_3_	toluene/MeOH	110 to 80	**10** (62)
11^[f]^	**8 c**	POCl_3_	toluene/MeOH	110 to 80	**10** (18)
12^[f]^	**8 d**	POCl_3_	toluene/MeOH	110 to 80	**10** (90)

[a] Reactions were performed with substrate **8** (0.15 mmol) and the amide activation reagent (0.15 mmol) in solvent (2.0 mL) as stated. [b] Yield of the isolated product. [c] T_3_P is propanephosphonic acid anhydride. [d] 2‐Chloropyridine (0.18 mmol) was used. [e] K_2_CO_3_ (1.5 mmol) was used. [f] A mixture of K_2_CO_3_ (1.5 mmol) and *n*Bu_4_NBr (0.015 mmol) in MeOH (2.0 mL) was added, and the temperature was decreased to 80 °C after the addition. DCM=dichloromethane, Tf=trifluoromethanesulfonyl, TMS=trimethylsilyl.

With optimized conditions in hand, we explored the scope of the reaction for the synthesis of diverse polycyclic scaffolds (Table 2). Substitutions at the indole ring with electron donating groups (**12 a** and **12 b**) and electron withdrawing groups (**12 c** and **12 d**) were well tolerated, providing the corresponding tetracyclic scaffolds in good yields (76–86 %).

**Table 2:** Substrate scope.^[a]^ 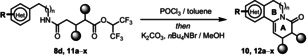

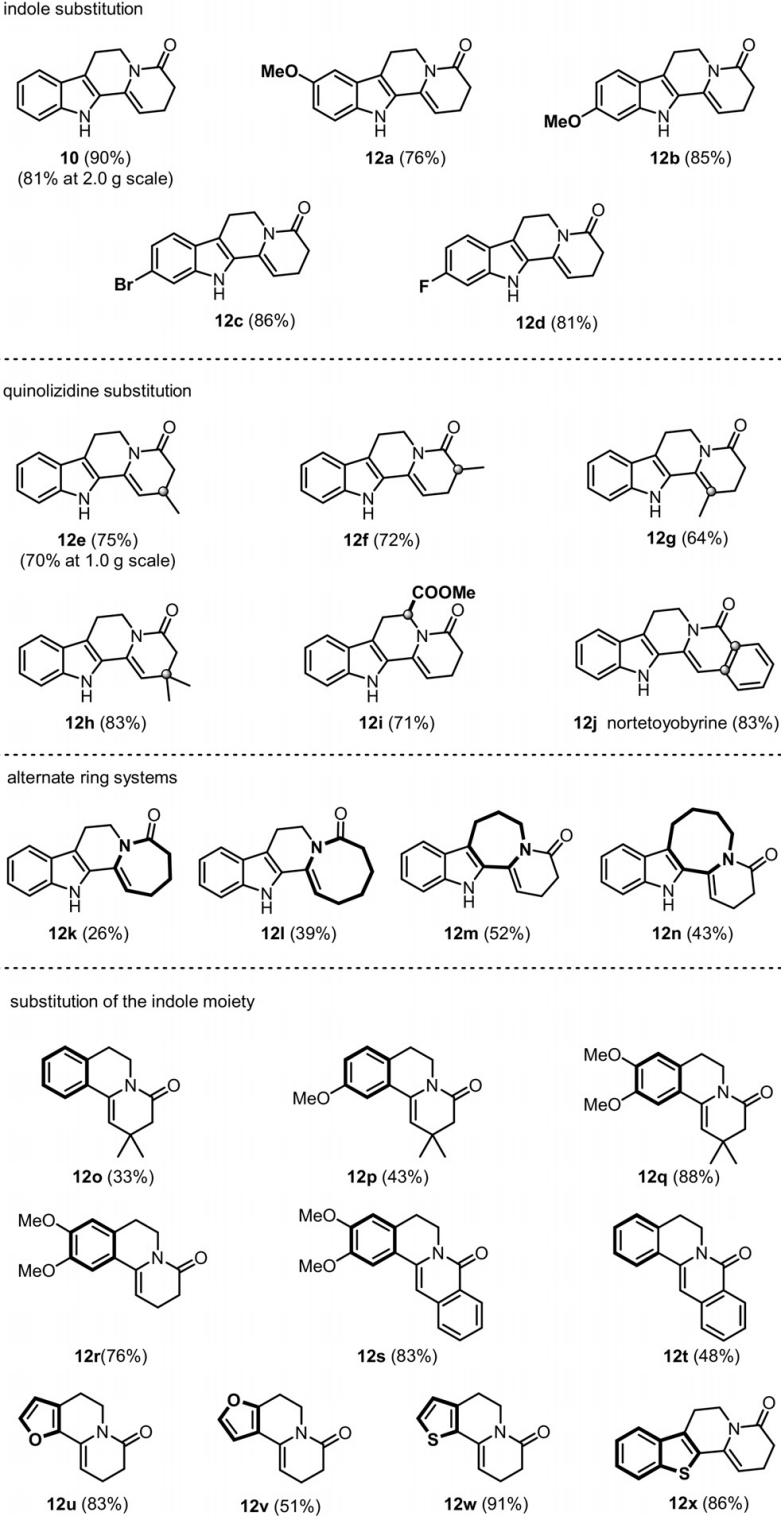

[a] Reactions were performed with substrate (0.10–4.7 mmol) using the standard procedure (yields are for the isolated product). See the Supporting Information for details.

Encouraged by these results, we investigated additional substitution patterns and ring systems. Substituting the quinolizidine core afforded the tetracycles **12 e**–**12 j** in good yields (64–83 %) thus providing access to the indoloquinolizidine‐type alkaloid nortetoyobyrine (**12 j**)^[13]^ in an additional step. The 7/6, 8/6, 6/7 and 6/8 fused ring systems were obtained in moderate yield (**12 k**–**12 n**, 26–52 %). Finally, we successfully expanded our strategy to benzene derivatives and heteroaromatic compounds, such as furan, thiophene, and benzothiophene (**12 o**–**12 x**, 33–91 %).

We next turned our attention to manipulations in the A ring in order to fully exploit our scaffold for natural product synthesis (Scheme 2). Through oxidation, a second double bond could be easily introduced to the 3,4‐position (**VII**). Reduction of the double bond in the 1,2‐position (**VIII**) could be achieved with or without concomitant reduction of the lactam. Moreover, introduction of a carbonyl group in 4‐position (**IX**) was key to the synthesis of more complex natural product.

**Scheme 2:**
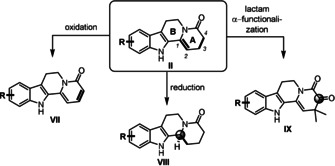
Diversification strategy for the tetracyclic scaffold.

Starting with the dehydrogenation, we tested selenium‐ and sulfur‐based reagents, such as PhSeCl, PhSeBr, PhSSPh and *N*‐*tert*‐butyl phenylsulfinimidoyl chloride (see the Supporting Information).^[14]^ Among standard protocols, only *N*‐*tert*‐butyl phenylsulfinimidoyl chloride afforded traces of the desired product. Gratifyingly, using the palladium‐catalyzed amide dehydrogenation protocol developed by Newhouse,^[15]^ demethoxyharmalanine (**14 a**), harmalanine (**14 b**), demethoxyharmalacinine (**14 c**) and harmalacinine (**14 d**),^[16]^ were successfully obtained in excellent yield (60–77 %; Scheme 3).

**Scheme 3:**
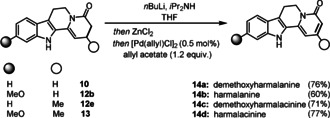
Oxidative diversification.

Racemic **15** can be obtained through selective catalytic hydrogenation of **10** using palladium on carbon (Scheme 4). From this intermediate, selenoxide elimination affords **16**, a key intermediate in the total synthesis of hirsutine (**2**), rhynchophylline (**17**) and isorhynchophylline (**18**).^[17]^ An asymmetric reduction of the C−C double bond was realized using chiral phosphoric acid (CPA) **19** and Hantzsch ester (**20**) system^[18]^ to give **15** in 80 % *ee* and 61 % yield. This material can be converted into (*S*)‐deplancheine (**3**) and (*S*)‐10‐desbromoarborescidine A (**21**) as previously reported.^[19]^

**Scheme 4:**
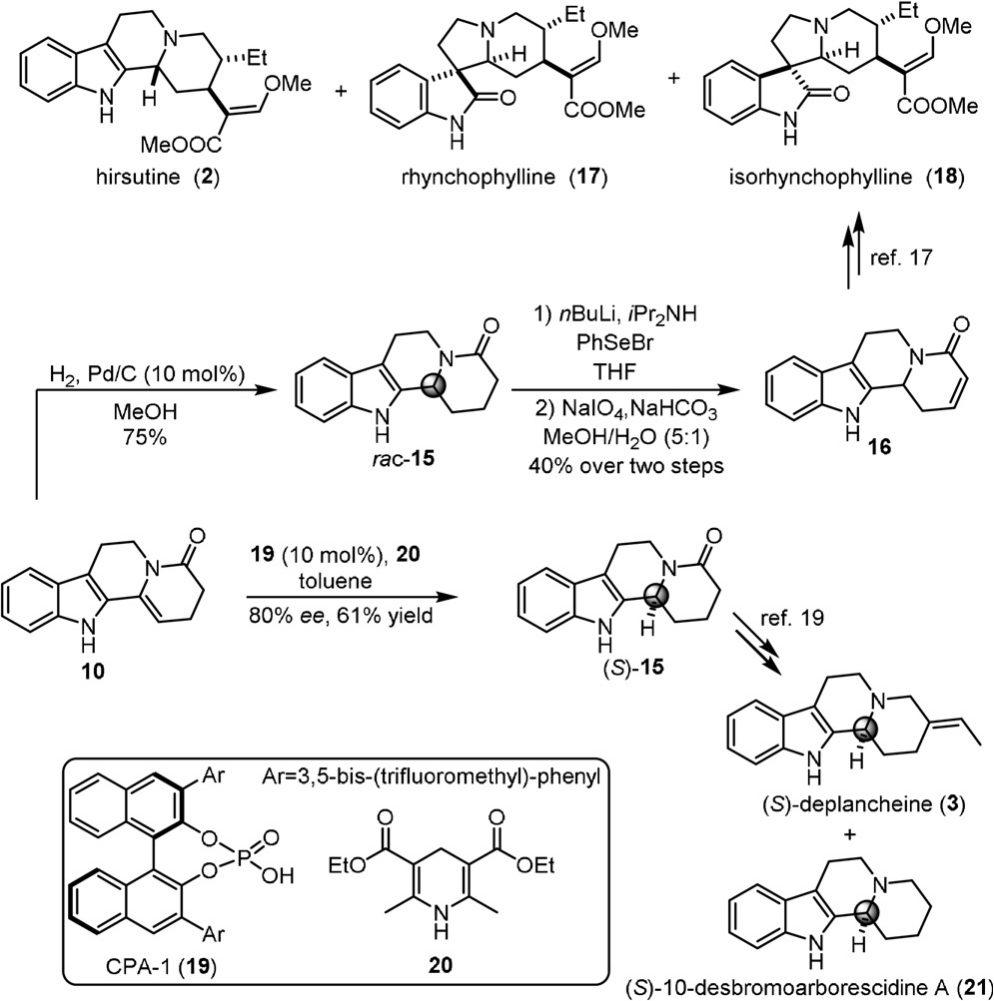
Reductive diversification.

To further demonstrate the synthetic potential of this method, we envisioned to use our annulation in a protecting‐group‐ and transition‐metal‐free asymmetric total synthesis of peganumine A (**6**).^[20]^ Following the established protocol, we successfully prepared the tetracyclic intermediate **25** in 85 % yield (Scheme 5). Subsequently, ketoenamide **26** was obtained in 50 % yield through a two‐step α‐oxidation sequence. The *tert*‐butoxycarbonyl (Boc) derivative of **26** constitutes an intermediate in Zhu's elegant total synthesis of peganumine A (**6**).^[21]^ At this point, we contemplated the possibility of a protecting‐group‐free synthesis. The key cascade cyclization was achieved using 0.2 equivalents of TFA in toluene to complete a protecting‐group‐free synthesis of (±)‐**6** in 42 % yield.

**Scheme 5:**
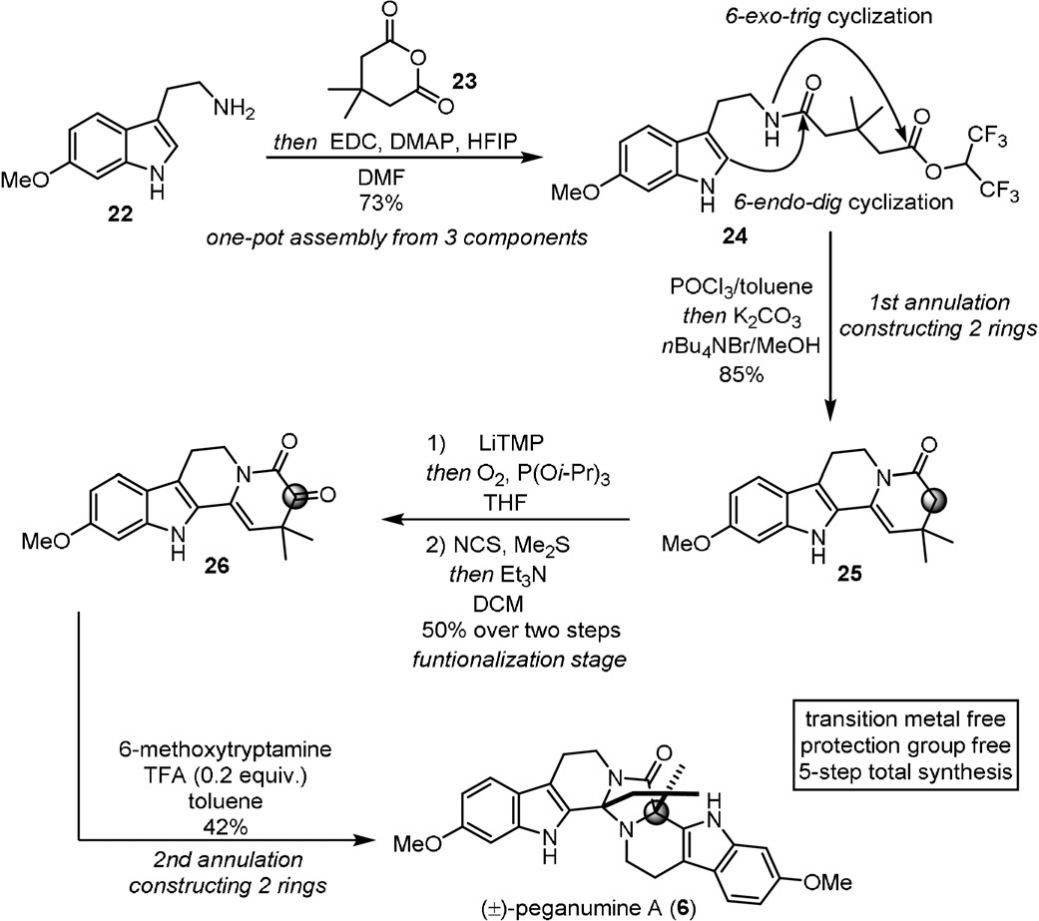
Protecting‐group‐free synthesis of (±)‐peganumine A. DMAP=4‐dimethylaminopyridine, DMF=*N*,*N*‐dimethylformamide, EDC=1‐ethyl‐3‐(3‐dimethylaminopropyl)carbodiimide, HFIP=hexafluoroisopropanol, TFA=trifluoroacetic acid, TMP=tetramethylpiperidide.

Encouraged by the success of the previous cascade cyclization, we initiated investigations toward an asymmetric total synthesis. First, we tested the chiral thiourea (CTU, Figure 2) and PhCO_2_H system developed by Jacobsen,^[22]^ which afforded 92 % *ee* in Zhu's synthesis^[21]^ for the Boc‐protected substrate. In our protecting‐group‐free substrate, with **27** and PhCO_2_H, the enantioselectivity was 9 % *ee* (Table 3). We speculated that the remarkable difference in enantioselectivity could be attributed to an impaired recognition between substrate and catalyst. It is possible that either the Boc group is crucial for the recognition, or that the free α‐ketoenamide **26** interrupted the substrate binding. Based on these considerations, we proposed to either apply a multi‐binding‐site catalyst to rigidify the transition state, or to use asymmetric counteranion directed catalysis (ACDC)^[23]^ as stronger chiral acid to activate the imine more efficiently.

**Figure 2:**
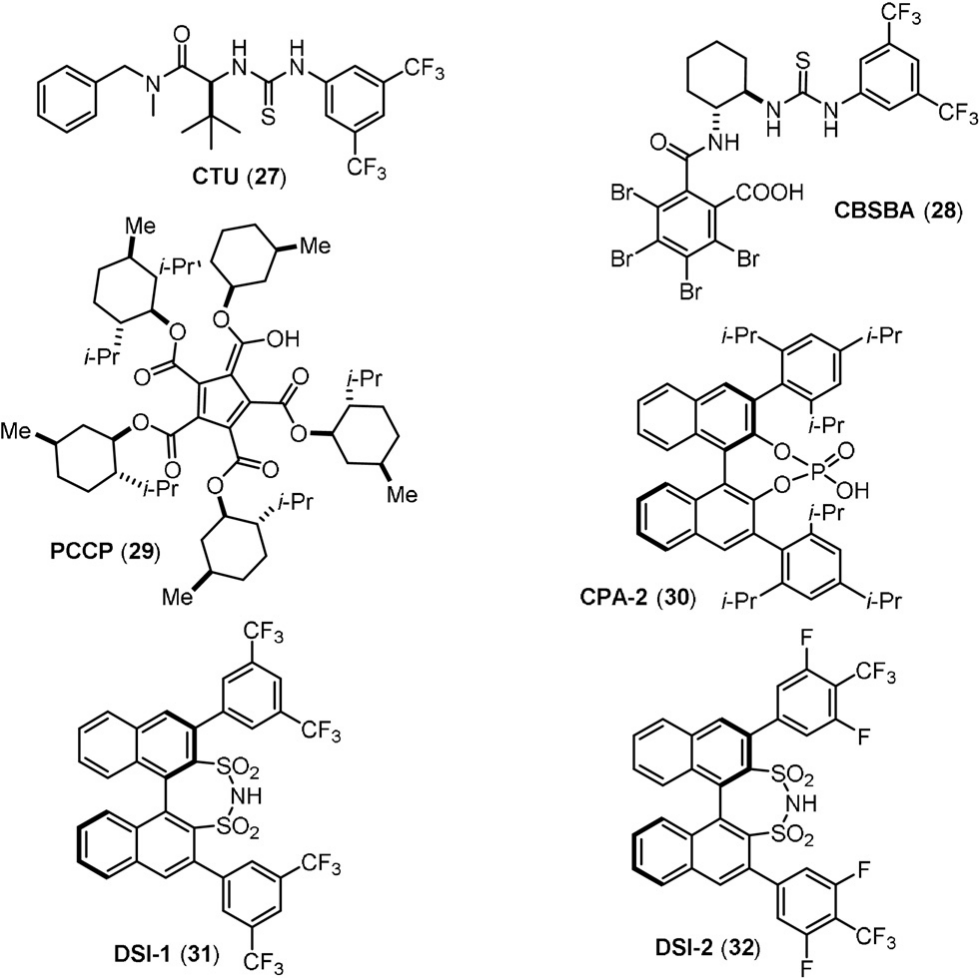
Representative chiral Brønsted acids.

**Table 3:** Optimization of the asymmetric Pictet–Spengler reaction cascade. 
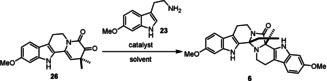

Entry	Catalyst (mol %)	Solvent	*T* [°C]	*ee* [%]	Yield [%]^[a]^
1	**CTU** (20) + PhCOOH (20)	toluene/DCM (9:1)	35	9	27
2	**CBSBA** (20)	toluene	110	N.D.	N.D.
3	**PCCP** (20)	toluene	110	4	44
4	**CPA‐2** (20)	toluene	110	10	32
5	**CPA‐2** (20)	toluene	90	31	60
6	**DSI‐1** (5)	toluene	70	79	53
7	**DSI‐2** (5)	toluene	70	83	66
8	**DSI‐2** (10)	toluene	70	94	34
9	**DSI‐2** (10)	toluene	60	97	81
10^[b]^	*ent*‐**DSI‐2** (10)	toluene	60	−97	68

[a] Yield of the isolated product; see the Supporting Information for detailed screening results. [b] The enantiomer of DSI‐2 was used to obtain the enantiomer of peganumine A.

First, we tested the conjugate‐base‐stabilized Brønsted acid (CBSBA) **28** developed by Seidel,^[24]^ and 1,2,3,4,5‐pentacarboxycyclopentadiene (PCCP) derived pentamenthyl ester **29**, a novel C−H acid discovered by Lambert,^[25]^ which are all multi‐binding‐site catalysts. However, no improvement of the enantioselectivity could be achieved with our substrate. Moving to the ACDC using CPA‐2 (**30**), a significant improvement of the enantioselectivity (31 % *ee*) was observed. Expanding on this idea, we applied the stronger chiral Brønsted acid disulfonimide (DSI)^[26]^ to the reaction and obtained 79 % *ee* with DSI‐1 (**31**). Encouraged by this promising result, and after intensive screening of DSIs (see the Supporting Information), we finally discovered that using DSI‐2 (**32**) could achieve 97 % *ee* and 81 % yield.

Inspired by the great potential of total synthesis of indole alkaloids, this annulation was further extended to the synthesis of dimeric berberine alkaloid ilicifoline B (**5**).^[27]^ Using our standard reaction sequence, 8‐oxopseudopalmatine (**36**)^[28]^ was obtained in 95 % yield for the annulation (Scheme 6). Using Opatz's dimerization procedure,^[29]^ racemic ilicifoline B was synthesized. Moreover, 8‐oxopseudopalmatine (**36**) can be transformed into the tetracyclic protoberberine alkaloid xylopinine (**37**) according to the reported method.^[30]^

**Scheme 6:**
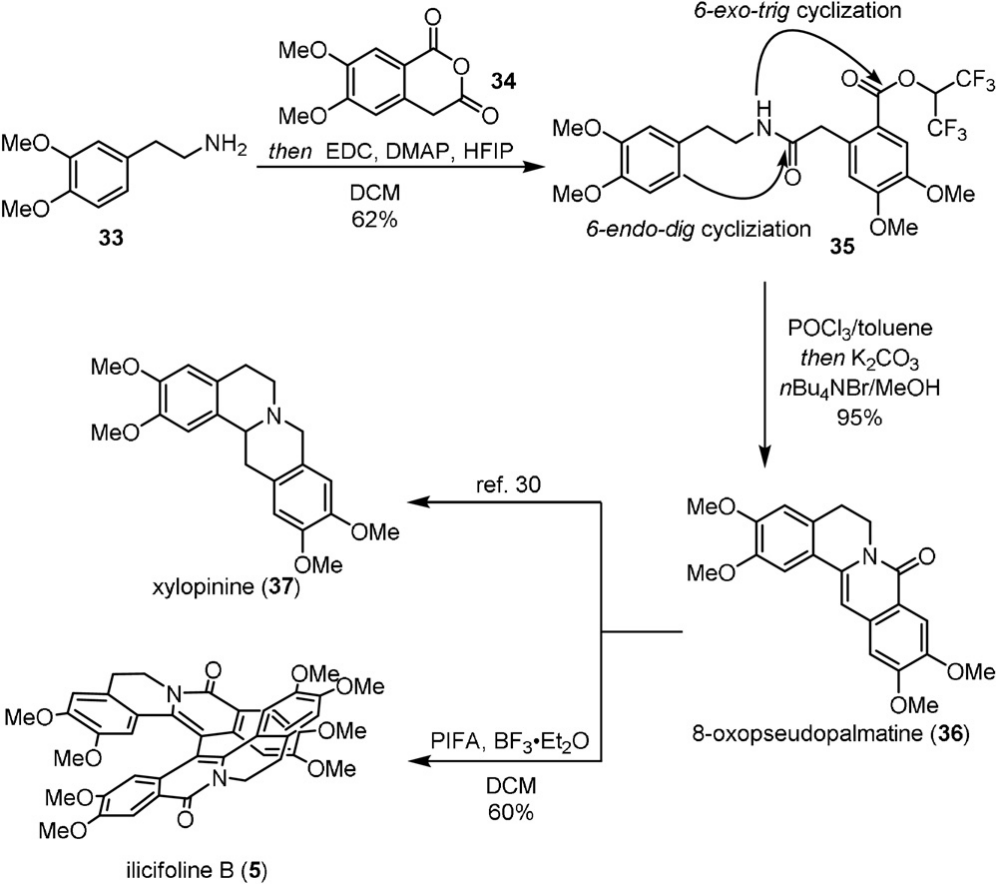
Synthesis of berberine alkaloids. PIFA=bis((trifluoroacetoxy)iodo)benzene.

In summary, we have developed an efficient method that is enabling to the rapid assembly of polycyclic scaffolds of bioactive alkaloids, through a straightforward annulation reaction featuring a complementary carbonyl activation strategy. Diverse polycyclic ring systems were accessed in good yields, enabling the total synthesis of different types of alkaloids and their analogs. Through diverging pathways, the total synthesis of five alkaloids and formal total synthesis of six alkaloids were completed. Among them, a synthesis of (+)‐ and (−)‐peganumine A (**6**) was achieved in a protecting‐group‐free sequence using a DSI catalyzed Pictet–Spengler reaction as the key step. Finally, we also applied this method to a synthesis of dimeric berberine alkaloid ilicifoline B (**5**).

## Conflict of interest

The authors declare no conflict of interest.

## Supporting information

As a service to our authors and readers, this journal provides supporting information supplied by the authors. Such materials are peer reviewed and may be re‐organized for online delivery, but are not copy‐edited or typeset. Technical support issues arising from supporting information (other than missing files) should be addressed to the authors.

Supplementary
