# Supplementary material for: Unified Synthesis of Polycyclic Alkaloids by Complementary Carbonyl Activation
Source: Angew Chem Int Ed Engl. 2021 May 1;60(24):13591–6. doi: 10.1002/anie.202102518 (PMC8252720; doi:10.1002/anie.202102518)

Angewandte Chemie
Eine Zeitschrift der Gesellschaft Deutscher Chemiker

Supporting Information

# Unified Synthesis of Polycyclic Alkaloids by Complementary Carbonyl Activation**

*Guoli He, Benjamin List,* and Mathias Christmann**

anie_202102518_sm_miscellaneous_information.pdf

# Table of Content

1. General information......3
2. Experimental procedures......5
2.1. Preparation of substrates......5
2.1.1. Substrates for the reaction optimization......6
2.1.2. Substrates for the reaction scope......15
2.1.3. Additional substrates......34
2.2. One-pot annulation reaction......40
2.2.1. Reaction optimization......40
2.2.2. Substrate scope......40
2.2.3. Detail and consideration of the annulation of the additional substrates......59
2.3. Oxidative pathway......62
2.3.1. Natural products and analogues synthesis......62
2.3.2. Optimization of regioselective dehydrogenation......66
2.4. Reductive pathway......67
2.5. Total synthesis of pegamunine A......71
2.5.1. Preparation of ketoamide......71
2.5.2. Racemic protection group free annulation reaction......74
2.5.3. Enantioselective protection group free annulation reaction......75
2.5.3.1. Catalyst preparation......75
2.5.3.2. Optimization of enantioselective protection group free cascade......82
2.5.3.4. NMR comparison between Hua's isolation and our synthesis......88
2.5.3.5. NMR comparison between Zhu's synthesis and our synthesis......90
2.5.3.6. Proposed mechanism and stereochemical model of the cascade......92
2.6. Total synthesis of ilicifoline B......93
2.7. Preparation of amines......98
2.7.1. Commercially available amines......98
2.7.2. Noncommercially available amines synthesis by reported methods......98
2.7.3. Self-made amines......99
2.8. Preparation of anhydrides......105
3. NMR Spectra......106

## 1. General information

Unless otherwise stated, all reactions were magnetically stirred and conducted in anhydrous solvents under argon, applying standard Schlenk techniques. Solvents and liquid reagents, as well as solutions of solid or liquid reagents were added via syringes, stainless steel cannulas through rubber septa or micropipettes in a weak stream of argon. Solid reagents were added through a weak argon counter-flow. Cooling baths were prepared in Dewar vessels, filled with ice/water (0 °C), or dry ice/acetone (–78 °C). Heated oil baths were used for reactions requiring elevated temperatures. Solvents were removed under reduced pressure at 40 °C using a rotary evaporator. All given yields are isolated yields of NMR spectroscopically pure materials, unless otherwise stated.

**Chemicals**

Chemicals (Abcr, Alfa Aesar, Apollo Scientific, Fisher Scientific, Fluorochem, Sigma-Aldrich, TCI) were purchased as reagent grade and used without further purification unless otherwise stated. Molecular sieves (MS) 4 Å were dried at 150 °C under vacuum and stored in Schlenk flask.

**Solvents**

Solvents (toluene, tetrahydrofuran (THF), dichloromethane (DCM)) were purified with the solvent purification system Braun MB-SPS-800 (Institut für Chemie und Biochemie, Freie Universität Berlin) or dried by distillation from an appropriate drying agent and stored in Schlenk flasks under argon (Max-Planck-Institut für Kohlenforschung). Additional solvents (dimethylformamide (DMF) and methanol) were purchased from Fisher Scientific in AcroSeal©-bottles under argon atmosphere with MS 4 Å.

**Thin-layer chromatography (TLC)**

TLC was performed using silica gel coated aluminum plates (ALUGRAM® Xtra SIL G/UV254, Macherey-Nagel) or pre-coated silica gel plastic sheets (Polygram SIL G/UV254, 0.20 mm, with fluorescent indicator; Macherey-Nagel) which were visualized under irradiation with UV light (λ = 254) or vanillin stain (500 mg vanillin, 5 mL $H_2SO_4$ (conc.), 10 mL HOAc, 100 mL MeOH).

**Column Chromatography**

Column chromatography was carried out using silica gel from Merck or Macherey & Nagel (60 Å, 230−400 mesh, particle size 40−63 µm) or neutral aluminium oxide from Fisher Scientific (Brockmann I, 60 Å, 50−200 µm) using technical grade solvents. Fractions containing a desired substance were combined and concentrated in vacuo. Solvent mixtures (mobile phase) are reported in terms of volume ratios (v/v).

**Nomenclature**

Nomenclature follows the suggestions proposed by the computer program ChemDraw Professional (17.1.0.105) of PerkinElmer.

**Nuclear Magnetic Resonance (NMR) Spectroscopy**

$^1$H and $^{13}$C NMR spectra were recorded on a JEOL ECX 400 (400 MHz), JEOL ECP 500 (500 MHz), Bruker Avance 500 (500 MHz), JEOL ECZ600 S (600 MHz) and Bruker Avance 700 (700 MHz) spectrometers in reported deuterated solvent. All spectra were processed with MestReNova 12.0.3. The residual deuterated solvent signal relative to tetramethylsilane (TMS) was used as the internal reference in $^1$H NMR spectra (DMSO δ [ppm] = 2.50; $CD_2Cl_2$ δ [ppm] = 5.32; $CD_3OD$ δ [ppm] = 3.31; $C_6D_6$ δ [ppm] = 7.16; $CDCl_3$ δ [ppm] = 7.26). Data are reported as follows: chemical shift, multiplicity (s = singlet, bs = broad singlet, d = doublet, t = triplet, q = quartet, m = multiplet), coupling constants (Hz) and integration. $^{13}$C NMR spectra reported in ppm from tetramethylsilane (TMS) with the solvent resonance as the internal standard (DMSO δ [ppm] = 39.5; $CD_2Cl_2$ δ [ppm] = 53.8; $CD_3OD$ δ [ppm] = 49.0, $CDCl_3$ δ [ppm] = 72.16; $C_6D_6$ δ [ppm] = 128.0).

**Mass Spectrometry**

High resolution mass spectrometry (HRMS) was performed on a Finnigan MAT 95 (EI), Bruker APEX III FTMS (ESI) or Agilent 6210 (ESI). The ionization method and mode of detection employed is indicated for the respective experiment and all masses are reported in atomic units per elementary charge (m/z).

**IR Spectroscopy**

IR Spectra were recorded on a JASCO FT/IR-4100 spectrometer. Characteristic absorption bands are reported in wavelengths $\nu$ in $cm^{-1}$ and were analyzed with the software Spectral Manager from JASCO.

**Specific Rotations**

Specific rotations $[\alpha]_D^T$ were measured on a Rudolph RA Autopol IV Automatic Polarimeter or JASCO P-2000 Polarimeter at the indicated temperature with a sodium lamp (sodium D line, $\lambda$ = 589 nm). Measurements were performed in an acid resistant 50 mm cell (Rudolph) or 100 mm (JASCO) with concentrations (g/100 mL) reported in the corresponding solvent.

**High Performance Liquid Chromatography (HPLC)**

HPLC was performed on Shimadzu LC-20AD liquid chromatograph (SIL-20AC auto sampler, CMB-20A communication bus module, DGU-20A5 degasser, CTO-20AC column oven, SPD-M20A diode array detector), Shimadzu LC-20AB liquid chromatograph (SIL-20ACHT auto sampler, DGU-20A5 degasser, CTO-20AC column oven, SPD-M20A diode array detector), or Agilent Technologies 1200 series with CHIRALPAK® IA. All solvents used were HPLC-grade solvents purchased from Sigma-Aldrich or VWR. The column employed and respective solvent mixture are indicated for each experiment.

## 2. Experimental procedures

### 2.1. Preparation of substrates

**General procedure A**

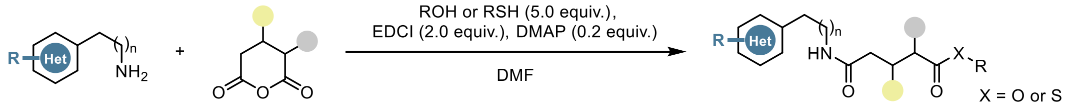


A mixture of the amine (1.0 equiv.) and the anhydride (1.0 equiv.) in DMF, was stirred overnight at room temperature. Then *N*-(3-dimethylaminopropyl)-*N'*-ethylcarbodiimide hydrochloride (EDCI) (2.0 equiv.), 4-dimethylaminopyridine (DMAP) (0.2 equiv.) and corresponding alcohol (5.0 equiv.) were added to the reaction

mixture subsequently. The resulting mixture was stirred for 24 h at room temperature, before the reaction mixture was diluted with $H_2O$ and EtOAc. After washing by brine for three times, the organic phase was dried over $MgSO_4$, and concentrated under reduced pressure. The crude product was purified by silica gel column chromatography. (Note: It is not necessary to perform the reaction under argon.)

**General procedure B**

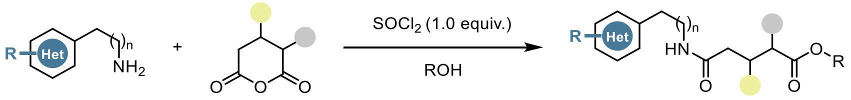


A mixture of the amine (1.0 equiv.) and the anhydride (1.0 equiv.) in corresponding alcohol was stirred overnight at room temperature. The mixture was then cooled to 0 ºC and $SOCl_2$ (1.0 equiv.) was added dropwise. Immediately after the addition, the reaction mixture was allowed to warm to room temperature and stirred for 2 h. The reaction was quenched with $NaHCO_3$ (sat. aq.) and extracted with DCM. The combined organic phases were washed with brine, dried over $MgSO_4$ and concentrated under reduced pressure. The crude product was purified by silica gel column chromatography.
(Note: It is not necessary to perform the reaction under argon.)

2.1.1. Substrates for the reaction optimization

**Methyl 5-((2-(1*H*-indol-3-yl)ethyl)amino)-5-oxopentanoate (8a)**.

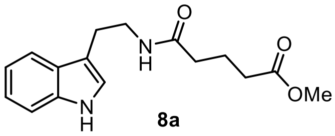

**8a**

**8a** was prepared according to **General procedure B**, starting from tryptamine (1.50 g, 9.38 mmol, 1.0 equiv.) and glutaric anhydride (1.07 g, 9.38 mmol, 1.0 equiv.) in MeOH (30 mL). The reaction mixture was subsequently treated with $SOCl_2$ (680 µL, 9.38 mmol, 1.0 equiv.). Purification by silica gel column chromatography (pentane/EtOAc = 3:2) afforded **8a** (2.39 g, 8.33 mmol, 88%) as a white solid.

**$^1$H NMR** (500 MHz, $CDCl_3$) δ [ppm] = 8.51 (s, 1H), 7.36 (dt, $J$ = 8.1, 1.0 Hz, 1H), 7.36

(dt, $J$ = 8.1, 1.0 Hz, 1H), 7.19 (ddd, $J$ = 8.2, 7.0, 1.2 Hz, 1H), 7.11 (ddd, $J$ = 8.0, 7.0, 1.0 Hz, 1H), 6.99 (d, $J$ = 2.3 Hz, 1H), 5.72 (s, 1H), 3.64 (s, 3H), 3.58 (td, $J$ = 6.8, 5.7 Hz, 2H), 2.95 (td, $J$ = 6.8, 0.8 Hz, 2H), 2.33 (t, $J$ = 7.3 Hz, 2H), 2.14 (t, $J$ = 7.4 Hz, 2H), 1.94–1.89 (m, 2H).

**$^{13}$C NMR** (126 MHz, $CDCl_3$) δ [ppm] = 173.8, 172.3, 136.6, 127.4, 122.3, 122.2, 119.4, 118.7, 112.8, 111.4, 51.7, 39.8, 35.6, 33.2, 25.4, 20.9.

**HRMS-ESI**: calcd. for $C_{16}H_{20}N_2O_3Na$ $[M + Na]^+$: 311.1366; found: 311.1371.

**FT-IR:** $v$ [$cm^{-1}$] = 3394, 3297, 2949, 1731, 1647, 1533, 1457, 1436, 1226, 1152, 743.

**4-Nitrophenyl 5-((2-(1*H*-indol-3-yl)ethyl)amino)-5-oxopentanoate (8b)**.

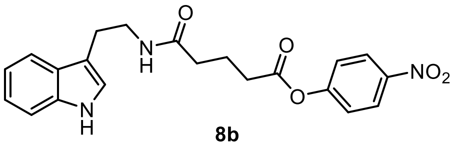


**8b**

**8b** was prepared according to **General procedure A**, starting from tryptamine (300 mg, 1.87 mmol, 1.0 equiv.) and glutaric anhydride (214 mg, 1.87 mmol, 1.0 equiv.) in DMF (10.0 mL). The reaction mixture was subsequently treated with EDCI (718 mg, 3.74 mmol, 2.0 equiv.), DMAP (45.8 mg, 0.374 mmol, 0.2 equiv.) and *p*-nitrophenol (1.30 g, 9.36 mmol, 5.0 equiv.). Purification by silica gel column chromatography (DCM/MeOH = 10:1) afforded **8b** (318 mg, 0.806 mmol, 43%) as a yellow solid.

**$^1$H NMR** (500 MHz, $CD_3OD$: $CDCl_3$ (v/v = 1:1)) δ [ppm] = 8.27–8.21 (m, 2H), 7.57–7.53 (m, 1H), 7.35–7.31 (m, 1H), 7.29–7.25 (m, 2H), 7.09 (ddd, $J$ = 8.2, 6.9, 1.2 Hz, 1H), 7.05–6.98 (m, 2H), 3.51 (t, $J$ = 7.2 Hz, 2H), 2.95 (t, $J$ = 7.3 Hz, 2H), 2.60 (t, $J$ = 7.4 Hz, 2H), 2.26 (t, $J$ = 7.3 Hz, 2H), 2.03–1.97 (m, 2H).

**$^{13}$C NMR** (176 MHz, $CD_3OD$: $CDCl_3$ (v/v = 1:1)) δ [ppm] = 173.0, 170.7, 155.2, 144.9, 136.3, 127.0, 124.7, 124.6, 122.1, 121.9, 121.0, 118.2, 117.8, 117.8, 111.6, 110.9, 39.7, 34.4, 32.7, 24.7, 20.2.

(Due the poor solubility of this compound, the mixture of $CD_3OD$: $CDCl_3$ (v/v = 1:1) was used for $^1$H NMR and $^{13}$C NMR spectroscopies.)

**HRMS-ESI**: calcd. for $C_{21}H_{21}N_3O_5K$ $[M + K]^+$: 434.1113; found: 434.1125.

**FT-IR**: $v$ [$cm^{-1}$] = 2925, 1591, 1522, 1336, 1290, 1208, 1109.

**Isopropyl 5-((2-(1*H*-indol-3-yl)ethyl)amino)-5-oxopentanoate (8c)**.

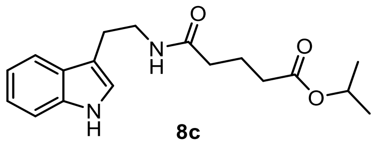

**8c**

**8c** was prepared according to **General procedure B**, starting from tryptamine (500 mg, 3.12 mmol, 1.0 equiv.) and glutaric anhydride (356 mg, 3.12 mmol, 1.0 equiv.) in *i*PrOH (10.0 mL). The reaction mixture was subsequently treated with $SOCl_2$ (221 µL, 3.12 mmol, 1.0 equiv.). Purification by silica gel column chromatography (pentane/EtOAc = 3:2) afforded **8c** (524 mg, 1.66 mmol, 53%) as a white solid.

**$^1$H NMR** (500 MHz, $CDCl_3$) δ [ppm] = 8.44 (s, 1H), 7.59 (d, *J* = 7.9 Hz, 1H), 7.36 (d, *J* = 8.1 Hz, 1H), 7.19 (t, *J* = 7.6 Hz, 1H), 7.11 (t, *J* = 7.5 Hz, 1H), 7.00 (s, 1H), 5.69 (s, 1H), 5.06–4.90 (m, 1H), 3.59 (q, *J* = 6.4 Hz, 2H), 2.96 (t, *J* = 6.8 Hz, 2H), 2.28 (t, *J* = 7.2 Hz, 2H), 2.14 (t, *J* = 7.4 Hz, 2H), 1.91 (t, *J* = 7.3 Hz, 2H), 1.22 (d, *J* = 1.9 Hz, 3H), 1.20 (d, *J* = 1.8 Hz, 3H).

**$^{13}$C NMR** (126 MHz, $CDCl_3$) δ [ppm] = 172.9, 172.4, 136.6, 127.4, 122.3, 122.2, 119.5, 118.8, 112.9, 111.4, 67.8, 39.8, 35.7, 33.8, 25.4, 21.9, 21.1.

**HRMS-ESI:** calcd. for $C_{18}H_{24}N_2O_3Na$ $[M + Na]^+$: 339.1679; found: 339.1692.

**FT-IR**: *ν* [cm$^{-1}$] = 3287, 2978, 1724, 1646, 1531, 1456, 1373, 1226, 1105, 740.

**1,1,1,3,3,3-Hexafluoropropan-2-yl 5-((2-(1*H*-indol-3-yl)ethyl)amino)-5-oxopentanoate (8d).**

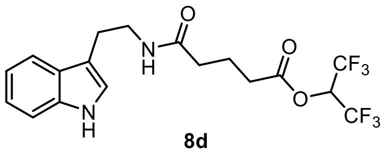

**8d**

**8d** was prepared according to **General procedure A**, starting from tryptamine (3.60 g, 22.5 mmol, 1.0 equiv.) and glutaric anhydride (2.56 g, 22.5 mmol, 1.0 equiv.) in DMF (80 mL). The reaction mixture was subsequently treated with EDCI (8.59 g, 45.0 mmol, 2.0 equiv.), DMAP (550 mg, 4.50 mmol, 0.2 equiv.) and hexafluoroisopropano (HFIP) (11.9 mL, 112 mmol, 5.0 equiv.). Purification by silica gel column chromatography (pentane/EtOAc = 3:2) afforded **8d** (4.80 g, 11.3 mmol, 50%) as a white solid.

**$^{1}$H NMR** (600 MHz, $CDCl_3$) δ [ppm] = 8.21 (s, 1H), 7.60 (d, *J* = 7.9 Hz, 1H), 7.37 (dd, *J* = 8.1, 1.0 Hz, 1H), 7.24–7.18 (m, 1H), 7.13 (ddd, *J* = 8.0, 7.0, 1.1 Hz, 1H), 7.01 (d, *J* = 2.3 Hz, 1H), 5.79–5.73 (m, 1H), 5.56 (t, *J* = 5.8 Hz, 1H), 3.62–3.59 (m, 2H), 2.98 (t, *J* = 6.8 Hz, 2H), 2.54 (t, *J* = 7.3 Hz, 2H), 2.15 (t, *J* = 7.2 Hz, 2H), 2.01–1.97 (m, 2H).

**$^{13}$C NMR** (151 MHz, $CDCl_3$) δ [ppm] = 171.5, 170.1, 136.6, 127.5, 122.4, 122.2, 120.5 (q, *J* = 282.2 Hz), 119.7, 118.8, 113.0, 111.4, 67.0–66.1 (m), 39.9, 34.9, 32.4, 25.4, 20.4.

**$^{19}$F NMR** (565 MHz, $CDCl_3$) δ [ppm] = –73.19 (d, *J* = 5.9 Hz).

**HRMS-ESI**: calcd. for $C_{18}H_{18}F_6N_2O_3Na$ $[M + Na]^+$: 447.1114; found: 447.1113.

**FT-IR**: *ν* [$cm^{-1}$] = 3418, 3328, 1777, 1753, 1633, 1540, 1384, 1359, 1286, 1228, 1201, 1108, 740.

**2,2,2-Trifluoroethyl 5-((2-(1*H*-indol-3-yl)ethyl)amino)-5-oxopentanoate (8e).**

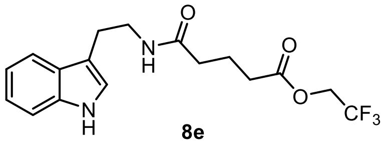


**8e**

**8e** was prepared according to **General procedure A**, starting from tryptamine (285 mg, 1.78 mmol, 1.0 equiv.) and glutaric anhydride (203 mg, 1.78 mmol, 1.0 equiv.) in DMF (10.0 mL). The reaction mixture was subsequently treated with EDCI (680 mg, 3.56 mmol, 2.0 equiv.), DMAP (43.5 mg, 0.356 mmol, 0.2 equiv.) and 2,2,2-trifluoroethanol (675 µL, 8.91 mmol, 5.0 equiv.). Purification by silica gel column chromatography (pentane/EtOAc = 3:2) afforded **8e** (275 mg, 0.772 mmol, 43%) as a white solid.

**$^{1}$H NMR** (500 MHz, $CDCl_3$) δ [ppm] = 8.13 (s, 1H), 7.60 (dd, *J* = 7.9, 1.1 Hz, 1H), 7.38 (dt, *J* = 8.1, 1.0 Hz, 1H), 7.21 (ddd, *J* = 8.2, 7.0, 1.2 Hz, 1H), 7.13 (ddd, *J* = 7.9, 7.0, 1.1 Hz, 1H), 7.04 (d, *J* = 2.3 Hz, 1H), 5.54 (s, 1H), 4.44 (q, *J* = 8.5 Hz, 2H), 3.61 (q, *J* = 6.5 Hz, 2H), 2.98 (td, *J* = 6.7, 0.8 Hz, 2H), 2.44 (t, *J* = 7.2 Hz, 2H), 2.16 (t, *J* = 7.3 Hz, 2H), 1.99–1.93 (m, 2H).

**$^{13}$C NMR** (126 MHz, $CDCl_3$) δ [ppm] = 171.8, 171.7, 136.6, 127.5, 123.1 (q, *J* = 277.0 Hz), 122.4, 122.2, 119.7, 118.8, 113.1, 111.4, 60.7–59.9 (m), 39.8, 35.3, 32.8, 25.4, 20.6.

**$^{19}$F NMR** (471 MHz, $CDCl_3$) δ [ppm] = –73.65 (t, *J* = 8.6 Hz).

**HRMS-ESI**: calcd. for $C_{17}H_{19}F_3N_2O_3Na$ $[M + Na]^+$: 379.1240; found: 379.1244.

**FT-IR**: *ν* [cm$^{-1}$] = 3388, 3322, 2927, 1736, 1630, 1545, 1411, 1275, 1261, 1179, 740.

**1,1,1-Trifluoropropan-2-yl 5-((2-(1*H*-indol-3-yl)ethyl)amino)-5-oxopentanoate(8f)**.

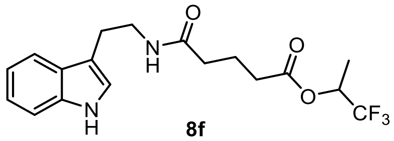

**8f**

**8f** was prepared according to **General procedure A**, starting from tryptamine (150 mg, 0.938 mmol, 1.0 equiv.) and glutaric anhydride (107 mg, 0.938 mmol, 1.0 equiv.) in DMF (5.0 mL). The reaction mixture was subsequently treated with EDCI (358 mg, 1.88 mmol, 2.0 equiv.), DMAP (22.9 mg, 0.188 mmol, 0.2 equiv.) and 1,1,1-trifluoro-2-propanol (424 µL, 4.69 mmol, 5.0 equiv.). Purification by silica gel column chromatography (pentane/EtOAc = 3:2) afforded **8f** (70.0 mg, 0.189 mmol, 20%) as a white solid.

**$^1$H NMR** (500 MHz, $CD_3OD$) δ [ppm] = 7.55 (dt, *J* = 7.9, 1.0 Hz, 1H), 7.32 (dt, *J* = 8.2, 0.9 Hz, 1H), 7.08 (ddd, *J* = 8.2, 6.9, 1.2 Hz, 1H), 7.04 (s, 1H), 7.00 (ddd, *J* = 7.9, 7.0, 1.0 Hz, 1H), 5.41–5.33 (m, 1H), 3.47 (t, *J* = 7.3 Hz, 2H), 2.98–2.88 (m, 2H), 2.35 (t, *J* = 7.5 Hz, 2H), 2.18 (t, *J* = 7.4 Hz, 2H), 1.90–1.84 (m, 2H), 1.36 (d, *J* = 6.6 Hz, 3H).

**$^{13}$C NMR** (126 MHz, $CD_3OD$) δ [ppm] = 174.9, 172.6, 138.1, 128.8, 125.6 (q, *J* = 279.1 Hz), 123.4, 119.6, 119.3, 113.2, 112.2, 68.2–67.4 (m), 41.4, 35.8, 33.7, 26.2, 21.9, 13.7, 13.7.

**$^{19}$F NMR** (471 MHz, $CD_3OD$) δ [ppm] = –80.2. (Due to the resolution, the coupling was not observed.)

**HRMS-ESI:** calcd. for $C_{18}H_{21}F_3N_2O_3Na$ $[M + Na]^+$: 393.1396; found: 393.1410.

**FT-IR**: *ν* [cm$^{-1}$] = 3396, 3324, 2932, 1749, 1730, 1631, 1542, 1458, 1386, 1278, 1187, 1165, 1118, 1092, 1017, 741.

**Perfluorophenyl 5-((2-(1*H*-indol-3-yl)ethyl)amino)-5-oxopentanoate (8g)**.

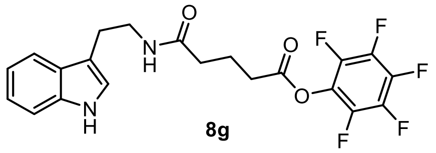

**8g**

**8g** was prepared according to **General procedure A**, starting from tryptamine (300 mg, 1.87 mmol, 1.0 equiv.) and glutaric anhydride (214 mg, 1.87 mmol, 1.0 equiv.) in DMF (10.0 mL). The reaction mixture was subsequently treated with EDCI (715 mg, 3.74 mmol, 2.0 equiv.), DMAP (45.8 mg, 0.374 mmol, 0.2 equiv.) and pentafluorophenol (1.71 g, 9.36 mmol, 5.0 equiv.). Purification by silica gel column chromatography (DCM/MeOH = 10:1) afforded **8g** (382 mg, 0.867 mmol, 46%) as a yellow solid.

**$^1$H NMR** (500 MHz, $CD_3OD$) δ [ppm] = 7.54 (dd, *J* = 7.9, 1.0 Hz, 1H), 7.30 (d, *J* = 8.1 Hz, 1H), 7.10–7.01 (m, 2H), 7.01–6.93 (m, 1H), 3.49 (t, *J* = 7.3 Hz, 2H), 2.94 (t, *J* = 7.3 Hz, 2H), 2.66 (t, *J* = 7.4 Hz, 2H), 2.26 (t, *J* = 7.4 Hz, 2H), 2.00–1.94 (m, 2H).

**$^{13}$C NMR** (126 MHz, $CD_3OD$) δ [ppm] = 174.7, 170.4, 143.4, 141.5, 140.2, 139.6, 138.1, 128.8, 126.4, 123.6, 123.4, 122.3, 119.6, 119.5, 119.3, 113.2, 112.2, 41.3, 35.6, 33.0, 26.2, 21.9. (Due to the complexity of the F–C coupling, the coupling constants were not calculated.)

**$^{19}$F NMR** (471 MHz, $CD_3OD$) δ [ppm] = –155.3 (d, *J* = 17.7 Hz), –161.3 (t, *J* = 20.7 Hz), –165.5 (m).

**HRMS-ESI**: calcd. for $C_{21}H_{17}F_5N_2O_3Na$ $[M + Na]^+$: 463.1051; found: 463.1067.

**FT-IR**: $\nu$ [cm$^{-1}$] = 3289, 2933, 1787, 1652, 1457, 1107, 1101, 743.

**3-Methylbut-2-en-1-yl 5-((2-(1*H*-indol-3-yl)ethyl)amino)-5-oxopentanoate (8h)**.

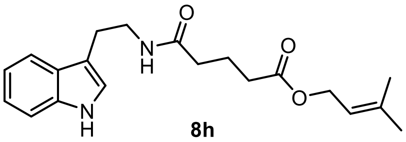

**8h**

**8h** was prepared according to **General procedure A**, starting from tryptamine (509 mg, 3.18 mmol, 1.0 equiv.) and glutaric anhydride (363 mg, 3.18 mmol, 1.0 equiv.) in DMF (15.0 mL). The reaction mixture was subsequently treated with EDCI (1.21 g,

6.36 mmol, 2.0 equiv.), DMAP (77.7 mg, 0.636 mmol, 0.2 equiv.) and 3-methyl-2-buten-1-ol (1.61 mL, 15.9 mmol, 5.0 equiv.). Purification by silica gel column chromatography (pentane/EtOAc = 5:2) afforded **8h** (468 mg, 1.37 mmol, 43%) as a light yellow solid.

**$^1$H NMR** (500 MHz, $CDCl_3$) δ [ppm] = 8.20 (s, 1H), 7.63–7.57 (m, 1H), 7.37 (dt, *J* = 8.2, 0.9 Hz, 1H), 7.21 (ddd, *J* = 8.2, 7.0, 1.2 Hz, 1H), 7.13 (ddd, *J* = 8.0, 7.0, 1.0 Hz, 1H), 7.03 (d, *J* = 2.4 Hz, 1H), 5.59 (s, 1H), 5.34–5.30 (m, 1H), 4.55 (d, *J* = 7.3 Hz, 2H), 3.60 (td, *J* = 6.7, 5.7 Hz, 2H), 2.97 (td, *J* = 6.8, 0.9 Hz, 2H), 2.33 (t, *J* = 7.2 Hz, 2H), 2.16 (t, *J* = 7.4 Hz, 2H), 1.95–1.89 (m, 2H), 1.75 (d, *J* = 1.4 Hz, 3H), 1.70 (d, *J* = 1.4 Hz, 3H).

**$^{13}$C NMR** (126 MHz, $CDCl_3$) δ [ppm] = 173.4, 172.3, 139.2, 136.6, 127.4, 122.24, 122.22, 119.5, 118.8, 118.6, 112.9, 111.4, 61.5, 39.8, 35.7, 33.5, 25.8, 25.4, 21.0, 18.1.

**HRMS-ESI**: calcd. for $C_{20}H_{26}N_2O_3Na$ $[M + Na]^+$: 365.1835; found: 365.1846.

**FT-IR**: *v* [$cm^{-1}$] = 3398, 3293, 2969, 2932, 1728, 1650, 1537, 1457, 1378, 1339, 1229, 1151, 956, 742.

**Benzhydryl 5-((2-(1*H*-indol-3-yl)ethyl)amino)-5-oxopentanoate (8i)**.

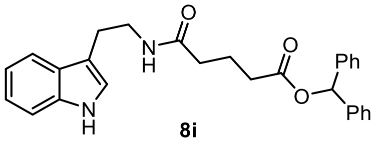

**8i**

**8i** was prepared according to **General procedure A**, starting from tryptamine (513 mg, 3.20 mmol, 1.0 equiv.) and glutaric anhydride (366 mg, 3.20 mmol, 1.0 equiv.) in DMF (18.0 mL). The reaction mixture was subsequently treated with EDCI (1.22 g, 6.41 mmol, 2.0 equiv.), DMAP (78.3 mg, 0.641 mmol, 0.2 equiv.) and diphenylmethanol (2.95 g, 16.0 mmol, 5.0 equiv.). Purification by silica gel column chromatography (pentane/EtOAc = 5:2) afforded **8i** (772 mg, 1.75 mmol, 55%) as a white solid.

**$^1$H NMR** (500 MHz, $CD_2Cl_2$) δ [ppm] = 8.30 (s, 1H), 7.55 (dd, *J* = 7.9, 2.7 Hz, 1H), 7.36–7.28 (m, 9H), 7.27–7.23 (m, 2H), 7.18–7.11 (m, 1H), 7.06 (dd, *J* = 8.6, 6.0 Hz, 1H), 6.97 (d, *J* = 2.7 Hz, 1H), 6.80 (d, *J* = 3.0 Hz, 1H), 5.55 (s, 1H), 3.52–3.48 (m, 2H),

2.89 (td, *J* = 7.0, 2.8 Hz, 2H), 2.47–2.37 (m, 2H), 2.06 (td, *J* = 7.5, 2.8 Hz, 2H), 1.95–1.84 (m, 2H).

**$^{13}$C NMR** (126 MHz, $CD_2Cl_2$) δ [ppm] = 172.2, 171.8, 140.6, 136.5, 128.6, 127.9, 127.4, 126.9, 122.2, 122.0, 119.3, 118.6, 112.9, 111.3, 76.9, 39.7, 35.4, 33.6, 25.4, 21.0.

**HRMS-ESI**: calcd. for $C_{28}H_{28}N_2O_3Na$ [M + Na]$^+$: 463.1992; found: 463.2008.

**FT-IR**: *ν* [cm$^{-1}$] = 3407, 3297, 2930, 1733, 1651, 1529, 1455, 1231, 1148, 743.

**Cyclohexyl 5-((2-(1*H*-indol-3-yl)ethyl)amino)-5-oxopentanoate (8j)**.

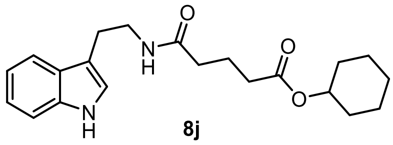


8j

**8j** was prepared according to **General procedure A**, starting from tryptamine (509 mg, 3.18 mmol, 1.0 equiv.) and glutaric anhydride (363 mg, 3.18 mmol, 1.0 equiv.) in DMF (18.0 mL). The reaction mixture was subsequently treated with EDCI (1.21 g, 6.36 mmol, 2.0 equiv.), DMAP (77.7 mg, 0.636 mmol, 0.2 equiv.) and cyclohexanol (1.65 mL, 15.9 mmol, 5.0 equiv.). Purification by silica gel column chromatography (pentane/EtOAc = 3:2) afforded **8j** (362 mg, 1.02 mmol, 32%) as a white solid.

**$^1$H NMR** (700 MHz, $CDCl_3$) δ [ppm] = 8.28 (s, 1H), 7.59 (dt, *J* = 7.8, 1.0 Hz, 1H), 7.37 (dt, *J* = 8.1, 0.9 Hz, 1H), 7.20 (ddd, *J* = 8.2, 7.0, 1.2 Hz, 1H), 7.12 (ddd, *J* = 8.0, 7.0, 1.0 Hz, 1H), 7.03–7.00 (m, 1H), 5.65 (s, 1H), 3.60 (td, *J* = 6.8, 5.8 Hz, 2H), 3.49 (td, *J* = 10.3, 4.0 Hz, 1H), 2.97 (td, *J* = 6.8, 0.9 Hz, 2H), 2.52 (t, *J* = 7.2 Hz, 2H), 2.14 (t, *J* = 7.4 Hz, 2H), 1.96–1.92 (m, 2H), 1.91–1.85 (m, 2H), 1.72–1.65 (m, 2H), 1.62–1.55 (m, 1H), 1.45–1.35 (m, 4H), 1.29–1.23 (m, 1H).

**$^{13}$C NMR** (126 MHz, $CDCl_3$) δ [ppm] = 172.8, 172.4, 136.6, 127.4, 122.24, 122.17, 119.5, 118.7, 112.8, 111.4, 72.8, 39.9, 35.7, 33.9, 31.7, 25.4, 23.8, 21.2. (One sp$^3$ carbon is missing, probably due to the resonance overlap.)

**HRMS-ESI**: calcd. for $C_{21}H_{28}N_2O_3Na$ [M + Na]$^+$: 379.1992; found: 379.2006.

**FT-IR**: *ν* [cm$^{-1}$] = 3357, 3325, 2948, 2920, 2853, 1707, 1635, 1539, 1386, 1189, 1011, 748, 727.

**Cyclohexyl 5-((2-(1*H*-indol-3-yl)ethyl)amino)-5-oxopentanethioate (8k)**.

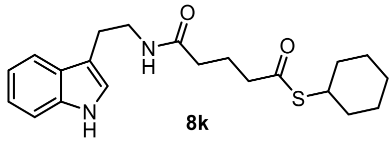

8k

**8k** was prepared according to **General procedure A**, starting from tryptamine (506 mg, 3.16 mmol, 1.0 equiv.) and glutaric anhydride (360 mg, 3.16 mmol, 1.0 equiv.) in DMF (18.0 mL). The reaction mixture was subsequently treated with EDCI (1.21 g, 6.36 mmol, 2.0 equiv.), DMAP (77.2 mg, 0.632 mmol, 0.2 equiv.) and cyclohexanethiol (1.93 mL, 15.8 mmol, 5.0 equiv.). Purification by silica gel column chromatography (pentane/EtOAc = 3:2) afforded **8k** (401 mg, 1.08 mmol, 34%) as a white solid.

**$^1$H NMR** (500 MHz, $CDCl_3$) δ [ppm] = 8.23 (s, 1H), 7.62 (dd, *J* = 7.9, 1.0 Hz, 1H), 7.39 (dt, *J* = 8.1, 1.0 Hz, 1H), 7.23 (ddd, *J* = 8.2, 7.0, 1.2 Hz, 1H), 7.15 (ddd, *J* = 8.0, 7.0, 1.1 Hz, 1H), 7.05 (d, *J* = 2.3 Hz, 1H), 5.62 (s, 1H), 3.62 (td, *J* = 6.8, 5.7 Hz, 2H), 3.51 (ddt, *J* = 10.3, 6.2, 3.6 Hz, 1H), 2.99 (td, *J* = 6.8, 0.8 Hz, 2H), 2.55 (t, *J* = 7.2 Hz, 2H), 2.16 (t, *J* = 7.4 Hz, 2H), 1.99–1.94 (m, 2H), 1.93–1.87 (m, 2H), 1.75–1.68 (m, 2H), 1.65–1.56 (m, 1H), 1.49–1.36 (m, 4H), 1.34–1.23 (m, 1H).

**$^{13}$C NMR** (176 MHz, $CDCl_3$) δ [ppm] = 199.2, 172.1, 136.6, 127.4, 122.3, 122.2, 119.6, 118.8, 113.0, 111.4, 43.1, 42.4, 39.8, 35.4, 33.1, 26.0, 25.6, 25.5, 21.7.

**HRMS-ESI:** calcd. for $C_{21}H_{28}N_2O_2SNa$ $[M + Na]^+$: 395.1763; found: 395.1782.

**FT-IR**: *ν* [cm$^{-1}$] = 3399, 3285, 2929, 2852, 1652, 1530, 1448, 1341, 998, 741.

***Tert*-butyl 5-((2-(1*H*-indol-3-yl)ethyl)amino)-5-oxopentanoate (8l)**.

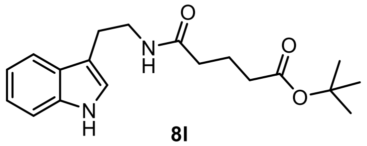

8l

**8l** was prepared according to **General procedure A**, starting from tryptamine (150 mg, 0.938 mmol, 1.0 equiv.) and glutaric anhydride (107 mg, 0.938 mmol, 1.0 equiv.) in DMF (5.0 mL). The reaction mixture was subsequently treated with EDCI (358 mg, 1.88 mmol, 2.0 equiv.), DMAP (22.9 mg, 0.188 mmol, 0.2 equiv.) and *t*BuOH (445 µL, 4.69 mmol, 5.0 equiv.). Purification by silica gel column chromatography (pentane/EtOAc = 3:2) afforded **8l** (21.0 mg, 636 µmol, 7%) as a white solid.

**$^1$H NMR** (600 MHz, $CDCl_3$) δ [ppm] = 8.28 (s, 1H), 7.63–7.58 (m, 1H), 7.37 (dd, *J* = 8.1, 1.0 Hz, 1H), 7.20 (ddd, *J* = 8.1, 7.1, 1.2 Hz, 1H), 7.12 (ddd, *J* = 8.0, 7.0, 1.0 Hz, 1H), 7.04–7.00 (m, 1H), 5.65 (s, 1H), 3.64–3.56 (m, 2H), 2.97 (t, *J* = 6.8 Hz, 2H), 2.23 (t, *J* = 7.2 Hz, 2H), 2.14 (t, *J* = 7.4 Hz, 2H), 1.90–1.86 (m, 2H), 1.43 (s, 9H).

**$^{13}$C NMR** (151 MHz, $CDCl_3$) δ [ppm] = 172.8, 172.4, 136.6, 127.4, 122.3, 122.2, 119.6, 118.8, 113.0, 111.4, 80.5, 39.8, 35.8, 34.8, 28.2, 25.5, 21.2.

**HRMS-ESI**: calcd. for $C_{19}H_{26}N_2O_3Na$ $[M + Na]^+$: 353.1835; found: 353.1852.

**FT-IR**: *ν* [$cm^{-1}$] = 3398, 3296, 2965, 2930, 1702, 1649, 1531, 1457, 1367, 1236, 1164, 1143, 741.

2.1.2. Substrates for the reaction scope

**1,1,1,3,3,3-Hexafluoropropan-2-yl 5-((2-(5-methoxy-1*H*-indol-3-yl)ethyl)amino)-5-oxopentanoate (11a).**

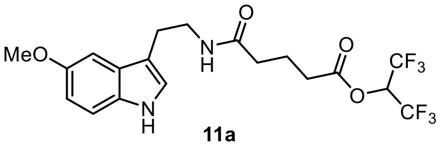


**11a**

**11a** was prepared according to **General procedure A**, starting from 5-methoxytryptamine (200 mg, 1.05 mmol, 1.0 equiv.) and glutaric anhydride (120 mg, 1.05 mmol, 1.0 equiv.) in DMF (5.0 mL). The reaction mixture was subsequently treated with EDCI (402 mg, 2.10 mmol, 2.0 equiv.), DMAP (25.7 mg, 0.211 mmol, 0.2 equiv.) and HFIP (556 μL, 5.26 mmol, 5.0 equiv.). Purification by silica gel column chromatography (pentane/EtOAc = 3:2) afforded **11a** (270 mg, 0.595 mmol, 56%) as a white solid.

**$^1$H NMR** (500 MHz, $CDCl_3$) δ [ppm] = 8.15 (s, 1H), 7.28–7.24 (m, 1H), 7.03 (d, *J* = 2.4 Hz, 1H), 7.00 (t, *J* = 2.4 Hz, 1H), 6.88 (dd, *J* = 8.8, 2.4 Hz, 1H), 5.82–5.71 (m, 1H), 5.60 (s, 1H), 3.86 (s, 3H), 3.65–3.57 (m, 2H), 2.97–2.91 (m, 2H), 2.56 (td, *J* = 7.3, 1.0 Hz, 2H), 2.17 (t, *J* = 7.2 Hz, 2H), 2.03–1.97 (m, 2H).

**$^{13}$C NMR** (126 MHz, $CDCl_3$) δ [ppm] = 171.6, 170.1, 154.2, 131.7, 127.8, 123.0, 120.5 (q, *J* = 285.6 Hz), 112.7, 112.5, 112.2, 100.7, 66.8–66.2 (m), 56.1, 39.7, 34.9, 32.4, 25.4, 20.4.

**$^{19}$F NMR** (565 MHz, $CDCl_3$) δ [ppm] = –73.18 (d, *J* = 5.8 Hz).

**HRMS-ESI**: calcd. for $C_{19}H_{21}F_6N_2O_4$ $[M + H]^+$: 455.1400; found: 455.1408.

**FT-IR**: *v* [cm$^{-1}$] = 3432, 3325, 2928, 1779, 1759, 1635, 1541, 1487, 1218, 1195, 1108, 927, 794.

**1,1,1,3,3,3-Hexafluoropropan-2-yl 5-((2-(6-methoxy-1*H*-indol-3-yl)ethyl)amino)-5-oxopentanoate (11b)**.

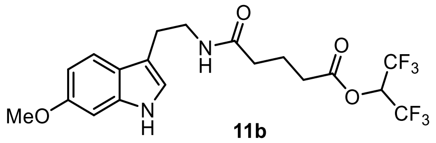


**11b**

**11b** was prepared according to **General procedure A**, starting from 6-methoxytryptamine (100 mg, 0.526 mmol, 1.0 equiv.) and glutaric anhydride (60.0 mg, 0.526 mmol, 1.0 equiv.) in DMF (3.0 mL). The reaction mixture was subsequently treated with EDCI (201 mg, 1.05 mmol, 2.0 equiv.), DMAP (12.9 mg, 0.105 mmol, 0.2 equiv.) and HFIP (278 µL, 2.63 mmol, 5.0 equiv.). Purification by silica gel column chromatography (pentane/EtOAc = 3:2) afforded **11b** (100 mg, 0.220 mmol, 42%) as a white solid.

**$^{1}$H NMR** (500 MHz, $CD_2Cl_2$) δ [ppm] = 8.14 (s, 1H), 7.44 (d, *J* = 8.6 Hz, 1H), 6.93 (dd, *J* = 2.2, 1.1 Hz, 1H), 6.87 (d, *J* = 2.3 Hz, 1H), 6.75 (dd, *J* = 8.7, 2.3 Hz, 1H), 5.86–5.79 (m, 1H), 5.59 (s, 1H), 3.82 (s, 3H), 3.54 (td, *J* = 6.9, 5.8 Hz, 2H), 2.90 (td, *J* = 6.9, 0.9 Hz, 2H), 2.56 (t, *J* = 7.4 Hz, 2H), 2.15 (t, *J* = 7.3 Hz, 2H), 2.00–1.90 (m, 2H).

**$^{13}$C NMR** (126 MHz, $CD_2Cl_2$) δ [ppm] = 171.5, 170.5, 157.1, 137.6, 122.2, 121.2, 120.9 (q, *J* = 282.4 Hz), 119.6, 113.3, 109.8, 94.9, 67.3–66.2 (m), 55.9, 40.1, 35.1, 32.7, 25.8, 20.7.

**$^{19}$F NMR** (471 MHz, $CD_2Cl_2$) δ [ppm] = –73.66. (Due to the resolution, the coupling was not observed.)

**HRMS-ESI**: calcd. for $C_{19}H_{20}F_6N_2O_4Na$ $[M + Na]^+$: 477.1219 found: 477.1222.

**FT-IR**: *v* [cm$^{-1}$] = 3302, 2965, 2937, 1764, 1635, 1384, 1281, 1257, 1107, 824.

**1,1,1,3,3,3-Hexafluoropropan-2-yl 5-((2-(6-bromo-1*H*-indol-3-yl)ethyl)amino)-5-oxopentanoate (11c)**.

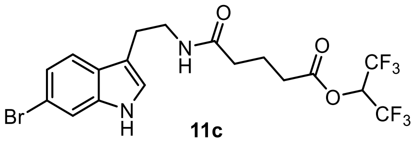

**11c**

**11c** was prepared according to **General procedure A**, starting from 6-bromotryptamine (100 mg, 0.420 mmol, 1.0 equiv.) and glutaric anhydride (47.9 mg, 0.420 mmol, 1.0 equiv.) in DMF (3.0 mL). The reaction mixture was subsequently treated with EDCI (160 mg, 0.840 mmol, 2.0 equiv.), DMAP (10.3 mg, 84.0 μmol, 0.2 equiv.) and HFIP (222 μL, 2.10 mmol, 5.0 equiv.). Purification by silica gel column chromatography (pentane/EtOAc = 3:2) afforded **11c** (120 mg, 0.239 mmol, 57%) as a white solid.

**$^{1}$H NMR** (700 MHz, $CDCl_3$) δ [ppm] = 8.23 (s, 1H), 7.52 (dd, *J* = 1.7, 0.6 Hz, 1H), 7.44 (dd, *J* = 8.4, 0.7 Hz, 1H), 7.22 (dd, *J* = 8.4, 1.7 Hz, 1H), 7.00 (dd, *J* = 2.3, 0.9 Hz, 1H), 5.77–5.74 (m, 1H), 5.53 (d, *J* = 6.2 Hz, 1H), 3.58 (td, *J* = 6.9, 5.8 Hz, 2H), 2.94 (td, *J* = 6.9, 0.9 Hz, 2H), 2.56 (t, *J* = 7.3 Hz, 2H), 2.18 (t, *J* = 7.3 Hz, 2H), 2.02–1.98 (m, 2H).

**$^{13}$C NMR** (176 MHz, $CDCl_3$) δ [ppm] = 171.6, 170.0, 137.3, 126.4, 123.0, 122.7, 121.3, 120.5 (q, *J* = 281.7 Hz), 120.0, 116.0, 114.4, 113.3, 66.9–66.1 (m), 39.9, 34.9, 32.4, 25.4, 20.4.

**$^{19}$F NMR** (471 MHz, $CDCl_3$) δ [ppm] = –73.17 (d, *J* = 6.4 Hz).

**HRMS-ESI**: calcd. for $C_{18}H_{17}BrF_6N_2O_3Na$ $[M + Na]^+$: 525.0219; found: 525.0244.

**FT-IR:** *v* [$cm^{-1}$] 3396, 3326, 2930, 1779, 1749, 1638, 1385, 1287, 1231, 1201, 1110, 803, 746.

**1,1,1,3,3,3-Hexafluoropropan-2-yl 5-((2-(6-fluoro-1*H*-indol-3-yl)ethyl)amino)-5-oxopentanoate (11d)**.

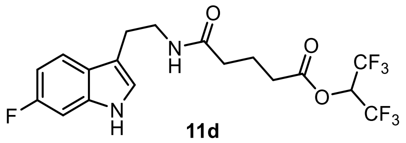

**11d**

**11d** was prepared according to **General procedure A**, starting from 6-fluorotryptamine

(260 mg, 1.46 mmol, 1.0 equiv.) and glutaric anhydride (166 mg, 1.46 mmol, 1.0 equiv.) in DMF (6.0 mL). The reaction mixture was subsequently treated with EDCI (558 mg, 2.92 mmol, 2.0 equiv.), DMAP (35.7 mg, 0.292 mmol, 0.2 equiv.) and HFIP (772 µL, 7.30 mmol, 5.0 equiv.). Purification by silica gel column chromatography (pentane/EtOAc = 3:2) afforded **11d** (350 mg, 0.792 mmol, 54%) as a white solid.

**$^{1}$H NMR** (400 MHz, $CDCl_3$) δ [ppm] = 8.07 (s, 1H), 7.50 (dd, *J* = 8.7, 5.3 Hz, 1H), 7.05 (dd, *J* = 9.7, 2.3 Hz, 1H), 7.01 (s, 1H), 6.90 (td, *J* = 9.1, 2.3 Hz, 1H), 5.78–5.72 (m, 1H), 5.51 (s, 1H), 3.59 (q, *J* = 6.5 Hz, 2H), 2.95 (t, *J* = 6.8 Hz, 2H), 2.56 (t, *J* = 7.2 Hz, 2H), 2.18 (t, *J* = 7.2 Hz, 2H), 2.04–1.97 (m, 2H).

**$^{13}$C NMR** (176 MHz, $CDCl_3$) δ [ppm] = 171.5, 170.1, 160.3 (d, *J* = 238.1 Hz), 136.5 (d, *J* = 12.6 Hz), 124.1, 122.3 (d, *J* = 2.9 Hz), 120.5 (d, *J* = 278.8 Hz), 119.6 (d, *J* = 10.1 Hz), 113.3, 108.6 (d, *J* = 24.5 Hz), 97.7 (d, *J* = 25.6 Hz), 66.9–66.1 (m), 39.8, 34.9, 32.4, 25.5, 20.4.

**$^{19}$F NMR** (376 MHz, $CDCl_3$) δ [ppm] = –73.17 (d, *J* = 10.2 Hz), (–120.71)–(–120.78) (m).

**HRMS-ESI**: calcd. for $C_{18}H_{18}F_7N_2O_3$ $[M + H]^+$: 443.1200; found: 443.1217.

**FT-IR:** *v* [$cm^{-1}$] = 3411, 3397, 3277, 1778, 1653, 1543, 1522, 1290, 1239, 1199, 1110.

**1,1,1,3,3,3-Hexafluoropropan-2-yl 5-((2-(1*H*-indol-3-yl)ethyl)amino)-3-methyl-5-oxopentanoate (11e)**.

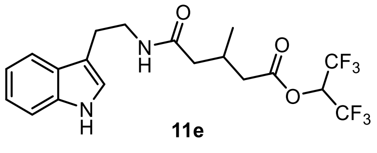


**11e**

**11e** was prepared according to **General procedure A**, starting from tryptamine (2.00 g, 12.5 mmol, 1.0 equiv.) and 3-methylglutaric anhydride (1.60 g, 12.5 mmol, 1.0 equiv.) in DMF (50 mL). The reaction mixture was subsequently treated with EDCI (4.78 g, 25.0 mmol, 2.0 equiv.), DMAP (305 mg, 2.50 mmol, 0.2 equiv.) and HFIP (6.60 mL, 62.5 mmol, 5.0 equiv.). Purification by silica gel column chromatography (pentane/EtOAc = 3:2) afforded **11e** (3.21 g, 7.33 mmol, 58%) as a white solid.

**$^{1}$H NMR** (500 MHz, $CD_2Cl_2$) δ [ppm] = 8.33 (s, 1H), 7.61–7.59 (m,, 1H), 7.38 (dt, *J* =

8.2, 1.0 Hz, 1H), 7.18 (ddd, $J$ = 8.2, 7.0, 1.2 Hz, 1H), 7.10 (ddd, $J$ = 7.9, 7.0, 1.0 Hz, 1H), 7.06–7.02 (m, 1H), 5.87–5.82 (m, 1H), 5.64 (s, 1H), 3.57 (td, $J$ = 6.8, 5.7 Hz, 2H), 2.96 (td, $J$ = 6.9, 0.9 Hz, 2H), 2.64 (dd, $J$ = 15.3, 5.3 Hz, 1H), 2.51–2.43 (m, 1H), 2.38 (dd, $J$ = 15.4, 8.0 Hz, 1H), 2.13 (dd, $J$ = 14.4, 6.8 Hz, 1H), 2.04 (dd, $J$ = 14.4, 7.1 Hz, 1H), 0.99 (d, $J$ = 6.7 Hz, 3H).

**$^{13}$C NMR** (126 MHz, $CD_2Cl_2$) δ [ppm] = 171.1, 169.8, 136.9, 127.8, 122.6, 122.4, 120.5 (q, $J$ = 282.1 Hz), 119.7, 119.0, 113.3, 111.6, 67.2–66.1 (m), 43.0, 40.1, 39.9, 28.2, 25.8, 19.5.

**$^{19}$F NMR** (471 MHz, $CD_2Cl_2$) δ [ppm] = –73.59 (d, $J$ = 12.9 Hz).

**HRMS-ESI**: calcd. for $C_{19}H_{20}F_6N_2O_3Na$ $[M + Na]^+$: 461.1270; found: 461.1269.

**FT-IR:** $\nu$ [cm$^{-1}$] = 3328, 1780, 1649, 1288, 1244, 1198, 1110, 933, 745.

**1,1,1,3,3,3-Hexafluoropropan-2-yl 5-((2-(1*H*-indol-3-yl)ethyl)amino)-2-methyl-5-oxopentanoate (11f)** and **1,1,1,3,3,3-hexafluoropropan-2-yl 5-((2-(1*H*-indol-3-yl)ethyl)amino)-4-methyl-5-oxopentanoate (11g)**.

**11f** and **11g** were prepared according to **General procedure A**, starting from tryptamine (500 mg, 3.12 mmol, 1.0 equiv.) and 2-methylglutaric anhydride (400 mg, 3.12 mmol, 1.0 equiv.) in DMF (10.0 mL). The reaction mixture was subsequently treated with EDCI (1.19 g, 6.25 mmol, 2.0 equiv.), DMAP (76.4 mg, 0.625 mmol, 0.2 equiv.) and HFIP (1.65 mL, 15.6 mmol, 5.0 equiv.). Purification by silica gel column chromatography (pentane/EtOAc = 3:2) afforded **11f** (190 mg, 0.434 mmol, 14%) as a light brown solid and **11g** (135 mg, 0.308 mmol, 10%) as a light brown solid.

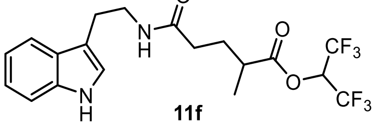


**11f**

**$^1$H NMR** (500 MHz, $CD_2Cl_2$) δ [ppm] = 8.23 (s, 1H), 7.62–7.57 (m, 1H), 7.38 (dd, $J$ = 8.1, 1.0 Hz, 1H), 7.18 (ddd, $J$ = 8.1, 7.0, 1.2 Hz, 1H), 7.10 (ddd, $J$ = 8.0, 7.0, 1.0 Hz, 1H), 7.06 (d, $J$ = 2.4 Hz, 1H), 5.85–5.80 (m, 1H), 5.55 (s, 1H), 3.55 (td, $J$ = 6.8, 5.7 Hz, 2H), 2.99–2.93 (m, 2H), 2.75–2.65 (m, 1H), 2.19–2.06 (m, 2H), 2.00–1.93 (m, 1H), 1.88–1.80 (m, 1H), 1.22 (d, $J$ = 7.0 Hz, 3H).

**$^{13}$C NMR** (126 MHz, $CD_2Cl_2$) δ [ppm] = 173.3, 171.6, 136.9, 127.8, 122.5, 122.4, 121.0 (d, *J* = 279.2 Hz), 119.7, 119.0, 113.4, 111.6, 67.2–66.1 (m), 40.1, 38.8, 33.8, 29.1, 25.7, 16.9.

**$^{19}$F NMR** (471 MHz, $CD_2Cl_2$) δ [ppm] = –73.64 (d, *J* = 28.6 Hz).

**HRMS-ESI**: calcd. for $C_{19}H_{20}F_6N_2O_3Na$ [M + Na]$^+$: 461.1270; found: 461.1270.

**FT-IR:** *v* [cm$^{-1}$] = 3328, 1781, 1635, 1457, 1388, 1288, 1241, 1200, 1109, 932, 741.

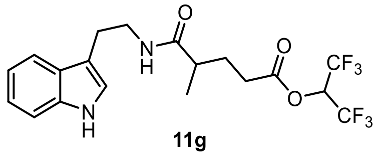

**11g**

**$^1$H NMR** (600 MHz, $CDCl_3$) δ [ppm] = 8.44 (s, 1H), 7.61 (dt, *J* = 7.9, 0.9 Hz, 1H), 7.36 (dt, *J* = 8.2, 0.9 Hz, 1H), 7.21 (ddd, *J* = 8.2, 7.0, 1.2 Hz, 1H), 7.13 (ddd, *J* = 8.0, 7.0, 1.0 Hz, 1H), 6.99 (d, *J* = 2.2 Hz, 1H), 5.79–5.68 (m, 1H), 5.69 (t, *J* = 5.9 Hz, 1H), 3.61 (td, *J* = 6.8, 5.8 Hz, 2H), 2.99–2.96 (m, 2H), 2.50 (ddd, *J* = 16.6, 8.7, 6.0 Hz, 1H), 2.41 (ddd, *J* = 16.6, 8.5, 7.0 Hz, 1H), 2.15–2.09 (m, 1H), 2.01–1.95 (m, 1H), 1.76–1.70 (m, 1H), 1.12 (d, *J* = 6.9 Hz, 3H).

**$^{13}$C NMR** (151 MHz, $CDCl_3$) δ [ppm] = 175.2, 170.2, 136.6, 127.4, 122.3, 122.2, 120.5 (d, *J* = 274.1 Hz), 119.5, 118.7, 112.7, 111.5, 66.9–66.0 (m), 40.2, 39.8, 31.0, 28.6, 25.4, 17.9.

**$^{19}$F NMR** (565 MHz, $CDCl_3$) δ [ppm] = –73.18 (d, *J* = 6.5 Hz).

**HRMS-ESI:** calcd. for $C_{19}H_{20}F_6N_2O_3Na$ [M + Na]$^+$: 461.1270; found: 461.1271.

**FT-IR:** *v* [cm$^{-1}$] = 3275, 2925, 2844, 1638, 1398, 1328, 1303, 1213, 1180, 742.

**1,1,1,3,3,3-Hexafluoropropan-2-yl 5-((2-(1*H*-indol-3-yl)ethyl)amino)-3,3-dimethyl-5-oxopentanoate (11h)**.

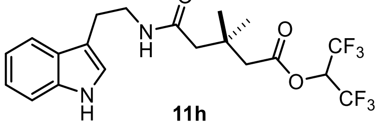

**11h**

**11h** was prepared according to **General procedure A**, starting from tryptamine (400 mg, 2.50 mmol, 1.0 equiv.) and 3,3-dimethylglutaric anhydride (355 mg, 2.50

mmol, 1.0 equiv.) in DMF (10.0 mL). The reaction mixture was subsequently treated with EDCI (955 mg, 5.00 mmol, 2.0 equiv.), DMAP (61.1 mg, 500 µmol, 0.2 equiv.) and HFIP (1.32 mL, 12.5 mmol, 5.0 equiv.). Purification by silica gel column chromatography (pentane/EtOAc = 3:2) afforded **11h** (697 mg, 1.54 mmol, 62%) as a white solid.

**$^{1}$H NMR** (500 MHz, $CDCl_3$) δ [ppm] = 8.21 (s, 1H), 7.60 (dt, *J* = 7.9, 0.9 Hz, 1H), 7.37 (dt, *J* = 8.1, 0.9 Hz, 1H), 7.21 (ddd, *J* = 8.2, 7.0, 1.2 Hz, 1H), 7.12 (ddd, *J* = 8.0, 7.0, 1.0 Hz, 1H), 7.01 (d, *J* = 2.4 Hz, 1H), 5.81 (s, 1H), 5.80–5.72 (m, 1H), 3.61 (td, *J* = 6.8, 5.7 Hz, 2H), 2.97 (td, *J* = 6.9, 0.9 Hz, 2H), 2.56 (s, 2H), 2.16 (s, 2H), 1.08 (s, 6H).

**$^{13}$C NMR** (126 MHz, $CDCl_3$) δ [ppm] = 170.7, 169.3, 136.6, 127.4, 122.3, 122.1, 120.6 (q, *J* = 283.3 Hz), 119.6, 118.8, 113.0, 111.4, 66.8–65.7 (m), 47.3, 43.8, 39.7, 33.5, 28.0, 25.4.

**$^{19}$F NMR** (565 MHz, $CDCl_3$) δ [ppm] = –72.98 (d, *J* = 6.4 Hz).

**HRMS-ESI**: calcd. for $C_{20}H_{23}F_6N_2O_3$ $[M + H]^+$: 453.1608; found: 453.1611.

**FT-IR:** *ν* [$cm^{-1}$] = 3238, 2963, 1779, 1647, 1522, 1388, 1289, 1236, 1198, 1109, 746.

**(*S*)-1,1,1,3,3,3-Hexafluoropropan-2-yl-5-((3-(1*H*-indol-3-yl)-1-methoxy-1-oxopropan-2-yl)amino)-5-oxopentanoate (11i)**.

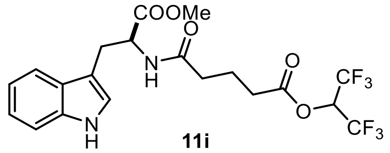


**11i**

**11i** was prepared according to **General procedure A**, starting from (*S*)-tryptophan methyl ester (300 mg, 1.38 mmol, 1.0 equiv.) and glutaric anhydride (157 mg, 1.38 mmol, 1.0 equiv.) in DMF (10.0 mL). The reaction mixture was subsequently treated with EDCI (526 mg, 2.75 mmol, 2.0 equiv.), DMAP (33.6 mg, 0.275 mmol, 0.2 equiv.) and HFIP (727 µL, 6.88 mmol, 5.0 equiv.). Purification by silica gel column chromatography (pentane/EtOAc = 3:2) afforded **11i** (150 mg, 0.311 mmol, 23%) as a white solid.

**$^{1}$H NMR** (500 MHz, $CDCl_3$) δ [ppm] = 8.32 (s, 1H), 7.53–7.50 (m, 1H), 7.34 (dt, *J* = 8.1, 0.9 Hz, 1H), 7.19 (ddd, *J* = 8.2, 7.0, 1.2 Hz, 1H), 7.11 (ddd, *J* = 8.0, 7.0, 1.0 Hz,

1H), 6.95 (d, $J$ = 2.4 Hz, 1H), 6.05 (d, $J$ = 8.0 Hz, 1H), 5.78–5.73 (m, 1H), 4.95 (dt, $J$ = 7.9, 5.5 Hz, 1H), 3.70 (s, 3H), 3.36–3.26 (m, 2H), 2.49 (t, $J$ = 7.4 Hz, 2H), 2.18 (t, $J$ = 7.4 Hz, 2H), 1.98–1.92 (m, 2H).

**$^{13}$C NMR** (126 MHz, $CDCl_3$) δ [ppm] = 172.5, 171.4, 170.0, 136.3, 127.8, 122.8, 122.4, 120.5 (q, $J$ = 278.7 Hz), 119.8, 118.5, 111.5, 110.0, 67.0–65.9 (m), 53.1, 52.5, 34.6, 32.2, 27.7, 20.1.

**$^{19}$F NMR** (471 MHz, $CDCl_3$) δ [ppm] = –73.19 (d, $J$ = 6.4 Hz).

**HRMS-ESI**: calcd. for $C_{20}H_{20}F_6N_2O_5Na$ $[M + Na]^+$: 505.1168; found: 505.1191.

**FT-IR**: $\nu$ [cm$^{-1}$] 3624, 3376, 2953, 1779, 1736, 1661, 1518, 1440, 1289, 1228, 1200, 1110, 781, 753.

$[\alpha]_D^{26}$ = +9.2($c$ = 0.7, $CHCl_3$).

**1,1,1,3,3,3-Hexafluoropropan-2-yl 2-(2-((2-(1*H*-indol-3-yl)ethyl)amino)-2-oxoethyl)benzoate (11j)**.

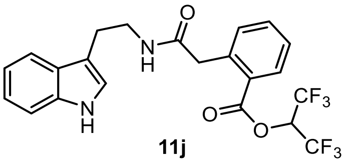


**11j**

**11j** was prepared according to **General procedure A**, starting from tryptamine (150 mg, 0.938 mmol, 1.0 equiv.) and homophthalic anhydride (152 mg, 0.938 mmol, 1.0 equiv.) in DMF (5.0 mL). The reaction mixture was subsequently treated with EDCI (197 mg, 1.03 mmol, 2.0 equiv.), DMAP (22.9 mg, 0.188 mmol, 0.2 equiv.) and HFIP (495 µL, 4.69 mmol, 5.0 equiv.). Purification by silica gel column chromatography (pentane/EtOAc = 5:2) afforded **11j** (32.0 mg, 678 µmol, 7%) as a white solid.

**$^1$H NMR** (500 MHz, $CDCl_3$) δ [ppm] = 8.06 (s, 1H), 8.02 (dd, $J$ = 7.9, 1.5 Hz, 1H), 7.60–7.54 (m, 2H), 7.43 (dd, $J$ = 7.8, 1.3 Hz, 1H), 7.40 (td, $J$ = 7.7, 1.3 Hz, 1H), 7.34 (dd, $J$ = 8.2, 1.0 Hz, 1H), 7.19 (ddd, $J$ = 8.1, 7.0, 1.2 Hz, 1H), 7.10 (ddd, $J$ = 8.1, 7.0, 1.0 Hz, 1H), 6.90 (d, $J$ = 2.3 Hz, 1H), 5.96 (s, 1H), 5.90–5.68 (m, 1H), 3.83 (s, 2H), 3.56 (td, $J$ = 6.9, 5.7 Hz, 2H), 2.98–2.86 (m, 2H).

**$^{13}$C NMR** (126 MHz, $CDCl_3$) δ [ppm] = 170.0, 163.8, 138.8, 136.5, 134.6, 132.9, 131.6, 127.8, 127.5, 125.9, 122.3, 122.1, 120.6 (q, $J$ = 282.9 Hz), 119.6, 118.8, 113.1, 111.3,

67.5–66.4 (m), 42.2, 40.1, 25.2.

**$^{19}$F NMR** (471 MHz, $CDCl_3$) δ [ppm] = –72.97. (It should be a doublet peak, but due to the resolution, only singlet peak was observed.)

**HRMS-ESI**: calcd. for $C_{22}H_{18}F_6N_2O_3Na$ $[M + Na]^+$: 495.1114; found: 495.1118.

**FT-IR**: *v* [cm$^{-1}$] = 3306, 1755, 1651, 1551, 1360, 1242, 1193, 1104, 1019, 915, 715.

**1,1,1,3,3,3-Hexafluoropropan-2-yl 6-((2-(1*H*-indol-3-yl)ethyl)amino)-6-oxohexanoate (11k)**.

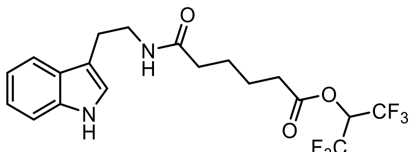


**11k**

**11k** was prepared according to **General procedure A**, starting from tryptamine (400 mg, 2.50 mmol, 1.0 equiv.) and oxepane-2,7-dione (320 mg, 2.50 mmol, 1.0 equiv.) in DMF (10.0 mL). The reaction mixture was subsequently treated with EDCI (955 mg, 5.00 mmol, 2.0 equiv.), DMAP (61.1 mg, 0.500 mmol, 0.2 equiv.) and HFIP (1.32 mL, 12.5 mmol, 5.0 equiv.). Purification by silica gel column chromatography (pentane/EtOAc = 3:2) afforded **11k** (289 mg, 0.660 mmol, 26%) as a white solid.

**$^1$H NMR** (500 MHz, $CDCl_3$) δ [ppm] = 8.73 (s, 1H), 7.59 (d, *J* = 7.9 Hz, 1H), 7.35 (d, *J* = 8.1 Hz, 1H), 7.21 (ddd, *J* = 8.1, 7.0, 1.2 Hz, 1H), 7.15–7.08 (m, 1H), 6.96 (d, *J* = 2.3 Hz, 1H), 5.87–5.75 (m, 2H), 3.61–3.57 (m, 2H), 2.96 (t, *J* = 6.8 Hz, 2H), 2.46 (t, *J* = 6.7 Hz, 2H), 2.15–2.01 (m, 2H), 1.67–1.62 (m, 4H).

**$^{13}$C NMR** (126 MHz, $CDCl_3$) δ [ppm] = 172.6, 170.3, 136.6, 127.5, 123.9, 122.2, 120.6 (q, *J* = 284.4 Hz), 119.4, 118.7, 112.7, 111.5, 67.0–65.9 (m), 40.0, 36.0, 33.0, 25.4, 24.8, 24.1.

**$^{19}$F NMR** (565 MHz, $CDCl_3$) δ [ppm] = –73.24 (d, *J* = 6.3 Hz).

**HRMS-ESI**: calcd. for $C_{19}H_{20}F_6N_2O_3Na$ $[M + Na]^+$: 461.1270; found: 461.1285.

**FT-IR**: *v* [cm$^{-1}$] = 2928, 2855, 1713, 1659, 1626, 1442, 1329, 1230, 745.

**$N$-(2-(1$H$-indol-3-yl)ethyl)-8-((1,1,1,3,3,3-hexafluoropropan-2-yl)oxy)-7-oxooctanamide (11l)**.

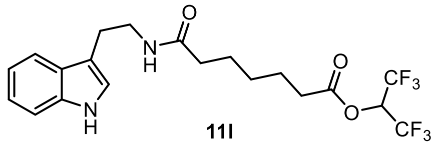

**11l**

**11l** was prepared according to **General procedure A**, starting from tryptamine (400 mg, 2.50 mmol, 1.0 equiv.) and oxocane-2,8-dione (355 mg, 2.50 mmol, 1.0 equiv.) in DMF (10.0 mL). The reaction mixture was subsequently treated with EDCI (955 mg, 5.00 mmol, 2.0 equiv.), DMAP (61.1 mg, 0.500 mmol, 0.2 equiv.) and HFIP (1.32 mL, 12.5 mmol, 5.0 equiv.). Purification by silica gel column chromatography (pentane/EtOAc = 3:2) afforded **11l** (300 mg, 0.644 mmol, 26%) as a white solid.

**$^1$H NMR** (500 MHz, $CDCl_3$) δ [ppm] = 8.62 (s, 1H), 7.60–7.58 (m, 1H), 7.36 (dt, $J$ = 8.1, 0.9 Hz, 1H), 7.20 (ddd, $J$ = 8.2, 7.0, 1.2 Hz, 1H), 7.11 (ddd, $J$ = 8.0, 7.0, 1.1 Hz, 1H), 6.98 (d, $J$ = 2.3 Hz, 1H), 5.80–5.75 (m, 1H), 5.70 (t, $J$ = 5.8 Hz, 1H), 3.59 (td, $J$ = 6.8, 5.7 Hz, 2H), 2.96 (td, $J$ = 6.8, 0.9 Hz, 2H), 2.44 (t, $J$ = 7.5 Hz, 2H), 2.08 (t, $J$ = 7.5 Hz, 2H), 1.71–1.52 (m, 4H), 1.34–1.20 (m, 2H).

**$^{13}$C NMR** (126 MHz, $CDCl_3$) δ [ppm] = 172.9, 170.4, 136.6, 127.5, 122.3, 122.2, 120.6 (q, $J$ = 281.4 Hz), 119.5, 118.7, 112.8, 111.5, 67.0–65.9 (m), 39.9, 36.4, 33.1, 28.4, 25.4, 25.2, 24.3.

**$^{19}$F NMR** (565 MHz, $CDCl_3$) δ [ppm] = –73.23 (d, $J$ = 6.4 Hz).

**HRMS-ESI**: calcd. for $C_{20}H_{22}F_6N_2O_3Na$ $[M + Na]^+$: 475.1427; found: 475.1442.

**FT-IR**: $\nu$ [cm$^{-1}$] = 3286, 2934, 2870, 1778, 1648, 1532, 1387, 1288, 1236, 1199, 1110, 745.

**1,1,1,3,3,3-Hexafluoropropan-2-yl 5-((3-(1$H$-indol-3-yl)propyl)amino)-5-oxopentanoate (11m)**.

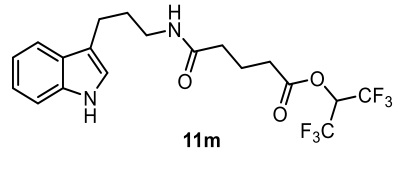

**11m**

**11m** was prepared according to **General procedure A**, starting from 1$H$-indole-3-

propanamine (85.0 mg, 0.489 mmol, 1.0 equiv.) and glutaric anhydride (55.7 mg, 0.489 mmol, 1.0 equiv.) in DMF (2.0 mL). The reaction mixture was subsequently treated with EDCI (187 mg, 0.977 mmol, 2.0 equiv.), DMAP (11.9 mg, 98.0 µmol, 0.2 equiv.) and HFIP (258 µL, 2.44 mmol, 5.0 equiv.). Purification by silica gel column chromatography (pentane/EtOAc = 3:2) afforded **11m** (120 mg, 0.274 mmol, 56%) as a white solid.

**$^1$H NMR** (500 MHz, $CDCl_3$) δ [ppm] = 8.11 (s, 1H), 7.59–7.57 (m, 1H), 7.36–7.34 (m, 1H), 7.19 (ddd, *J* = 8.2, 7.0, 1.2 Hz, 1H), 7.14–7.10 (m, 1H), 6.98 (dd, *J* = 2.3, 1.2 Hz, 1H), 5.80–5.75 (m, 1H), 5.46 (s, 1H), 3.32 (td, *J* = 7.1, 5.9 Hz, 2H), 2.80 (td, *J* = 7.3, 0.9 Hz, 2H), 2.55 (t, *J* = 7.2 Hz, 2H), 2.12 (t, *J* = 7.3 Hz, 2H), 2.00–1.89 (m, 4H).

**$^{13}$C NMR** (126 MHz, $CDCl_3$) δ [ppm] = 171.5, 170.1, 136.6, 127.4, 122.2, 121.6, 119.4, 118.8, 115.5, 111.3, 66.8–66.3 (m), 39.6, 34.9, 32.4, 29.8, 22.8, 20.4. (Due to the resolution or/and overlapping, the resonance of $CF_3$ was not observed.)

**$^{19}$F NMR** (471 MHz, $CDCl_3$) δ [ppm] = –73.18 (d, *J* = 6.4 Hz).

**HRMS-ESI**: calcd. for $C_{19}H_{20}F_6N_2O_3Na$ $[M + Na]^+$: 461.1270; found: 461.1287.

**FT-IR**: *v* [$cm^{-1}$] = 3404, 3295, 2925, 1780, 1649, 1385, 1356, 1288, 1228, 1200, 1110, 744.

**1,1,1,3,3,3-Hexafluoropropan-2-yl 5-((4-(1*H*-indol-3-yl)butyl)amino)-5-oxopentanoate (11n)**.

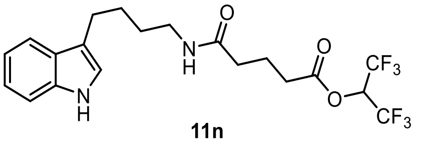


11n

**11n** was prepared according to **General procedure A**, starting from 1*H*-indole-3-butanamine (120 mg, 0.638 mmol, 1.0 equiv.) and glutaric anhydride (72.8 mg, 0.638 mmol, 1.0 equiv.) in DMF (2.0 mL). The reaction mixture was subsequently treated with EDCI (244 mg, 1.28 mmol, 2.0 equiv.), DMAP (15.6 mg, 0.128 mmol, 0.2 equiv.) and HFIP (337 µL, 3.19 mmol, 5.0 equiv.). Purification by silica gel column chromatography (pentane/EtOAc = 3:2) afforded **11n** (140 mg, 0.310 mmol, 48%) as a white solid.

**$^1$H NMR** (500 MHz, $CDCl_3$) δ [ppm] = 8.06 (s, 1H), 7.62–7.56 (m, 1H), 7.35 (dt, *J* = 8.2, 0.9 Hz, 1H), 7.19 (ddd, *J* = 8.1, 7.0, 1.2 Hz, 1H), 7.11 (ddd, *J* = 8.0, 7.0, 1.0 Hz, 1H), 6.96 (dd, *J* = 2.3, 1.0 Hz, 1H), 5.80–5.75 (m, 1H), 5.43 (s, 1H), 3.26 (td, *J* = 7.2, 5.7 Hz, 2H), 2.78 (td, *J* = 7.4, 0.9 Hz, 2H), 2.57 (t, *J* = 7.2 Hz, 2H), 2.16 (t, *J* = 7.3 Hz, 2H), 2.01 (q, *J* = 7.3 Hz, 2H), 1.80–1.69 (m, 2H), 1.60–1.54 (m, 2H).

**$^{13}$C NMR** (126 MHz, $CDCl_3$) δ [ppm] = 171.5, 170.1, 136.5, 127.5, 122.0, 121.5, 120.5 (d, *J* = 282.6 Hz), 119.2, 119.0, 116.3, 111.3, 66.7–66.2 (m), 39.6, 34.9, 32.4, 29.4, 27.4, 24.81, 20.4.

**$^{19}$F NMR** (471 MHz, $CDCl_3$) δ [ppm] = –73.17 (d, *J* = 6.3 Hz).

**HRMS-ESI**: calcd. for $C_{20}H_{22}F_6N_2O_3Na$ $[M + Na]^+$: 475.1427; found: 475.1436.

**FT-IR**: *v* [$cm^{-1}$] = 3401, 2924, 2854, 1781, 1651, 1288, 1228, 1200, 1110, 927, 906, 744.

**1,1,1,3,3,3-Hexafluoropropan-2-yl 3,3-dimethyl-5-oxo-5-(phenethylamino)pentanoate (11o)**.

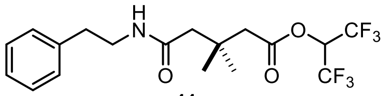


**11o**

**11o** was prepared according to **General procedure A**, starting from phenethylamine (300 mg, 2.48 mmol, 1.0 equiv.) and 3,3-dimethylglutaric anhydride (352 mg, 2.48 mmol, 1.0 equiv.) in DMF (10.0 mL). The reaction mixture was subsequently treated with EDCI (947 mg, 4.96 mmol, 2.0 equiv.), DMAP (60.6 mg, 0.496 mmol, 0.2 equiv.) and HFIP (1.31 mL, 12.4 mmol, 5.0 equiv.). Purification by silica gel column chromatography (pentane/EtOAc = 5:2) afforded **11o** (452 mg, 1.09 mmol, 44%) as a white solid.

**$^1$H NMR** (500 MHz, $CDCl_3$) δ [ppm] = 7.31–7.27 (m, 2H), 7.24–7.17 (m, 3H), 5.80–5.71 (m, 2H), 3.54 (td, *J* = 7.0, 5.8 Hz, 2H), 2.82 (t, *J* = 6.9 Hz, 2H), 2.50 (s, 2H), 2.17 (s, 2H), 1.08 (s, 6H).

**$^{13}$C NMR** (126 MHz, $CDCl_3$) δ [ppm] = 170.6, 169.4, 138.9, 128.8, 128.8, 126.7, 120.5 (q, *J* = 279.6 Hz), 66.8–65.7 (m), 47.2, 43.7, 40.4, 35.7, 33.5, 28.0.

**$^{19}$F NMR** (471 MHz, $CDCl_3$) δ [ppm] = –73.02 (d, *J* = 6.3 Hz).

**HRMS-ESI**: calcd. for $C_{18}H_{21}F_6NO_3Na$ $[M + Na]^+$: 436.1318; found: 436.1335.

**FT-IR**: *v* [cm$^{-1}$] = 3309, 1967, 1645, 1543, 1282, 1268, 1200, 1107.

**1,1,1,3,3,3-Hexafluoropropan-2-yl 5-((4-methoxyphenethyl)amino)-3,3-dimethyl-5-oxopentanoate (11p)**.

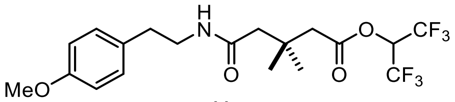


**11p**

**11p** was prepared according to **General procedure A**, starting from 4-methoxyphenethylamine (300 mg, 1.99 mmol, 1.0 equiv.) and 3,3-dimethylglutaric anhydride (282 mg, 1.99 mmol, 1.0 equiv.) in DMF (10.0 mL). The reaction mixture was subsequently treated with EDCI (759 mg, 3.97 mmol, 2.0 equiv.), DMAP (48.5 mg, 0.397 mmol, 0.2 equiv.) and HFIP (1.05 mL, 9.93 mmol, 5.0 equiv.). Purification by silica gel column chromatography (pentane/EtOAc = 5:2) afforded **11p** (523 mg, 1.18 mmol, 59%) as a white solid.

**$^1$H NMR** (500 MHz, $CDCl_3$) δ [ppm] = 7.13–7.06 (m, 2H), 6.85–6.79 (m, 2H), 5.72–5.70 (m, 2H), 3.78 (s, 3H), 3.49 (td, *J* = 7.0, 5.8 Hz, 2H), 2.75 (t, *J* = 7.0 Hz, 2H), 2.53 (s, 2H), 2.17 (s, 2H), 1.08 (s, 6H).

**$^{13}$C NMR** (126 MHz, $CDCl_3$) δ [ppm] = 170.6, 169.4, 158.5, 130.8, 129.8, 120.6 (q, *J* = 291.0 Hz), 114.2, 66.6–66.0 (m), 55.3, 47.2, 43.7, 40.6, 34.8, 33.5, 28.0.

**$^{19}$F NMR** (471 MHz, $CDCl_3$) δ [ppm] = –73.03 (d, *J* = 6.3 Hz).

**HRMS-ESI**: calcd. for $C_{19}H_{23}F_6NO_4Na$ $[M + Na]^+$: 466.1423; found: 466.1442.

**FT-IR**: *v* [cm$^{-1}$] = 2970, 1738, 1515, 1440, 1366, 1217, 1105.

**1,1,1,3,3,3-Hexafluoropropan-2-yl 5-((3,4-dimethoxyphenethyl)amino)-3,3-dimethyl-5-oxopentanoate (11q)**.

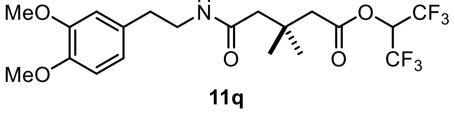


**11q**

**11q** was prepared according to **General procedure A**, starting from 3,4-

dimethoxyphenethylamine (500 mg, 2.76 mmol, 1.0 equiv.) and 3,3-dimethylglutaric anhydride (392 mg, 2.76 mmol, 1.0 equiv.) in DMF (15.0 mL). The reaction mixture was subsequently treated with EDCI (1.05 g, 5.52 mmol, 2.0 equiv.), DMAP (67.5 mg, 0.552 mmol, 0.2 equiv.) and HFIP (1.46 mL, 13.8 mmol, 5.0 equiv.). Purification by silica gel column chromatography (pentane/EtOAc = 5:2) afforded **11q** (670 mg, 1.42 mmol, 51%) as a white solid.

**$^1$H NMR** (500 MHz, $CDCl_3$) δ [ppm] = 6.80–6.77 (m, 1H), 6.71 (d, $J$ = 7.6 Hz, 2H), 5.80–5.70 (m, 2H), 3.86 (s, 3H), 3.85 (s, 3H), 3.51 (td, $J$ = 7.0, 5.8 Hz, 2H), 2.75 (t, $J$ = 7.0 Hz, 2H), 2.54 (s, 2H), 2.18 (s, 2H), 1.08 (s, 6H).

**$^{13}$C NMR** (126 MHz, $CDCl_3$) δ [ppm] = 170.6, 169.4, 149.2, 147.8, 131.3, 120.7, 120.5 (q, $J$ = 280.8 Hz), 111.8, 111.4, 66.5–65.7 (m), 56.0, 55.9, 47.2, 43.7, 40.5, 35.3, 33.5, 28.1.

**$^{19}$F NMR** (471 MHz, $CDCl_3$) δ [ppm] = –73.01 (d, $J$ = 6.5 Hz).

**HRMS-ESI**: calcd. for $C_{20}H_{25}F_6NO_5Na$ $[M + Na]^+$: 496.1529; found: 496.1518.

**FT-IR:** $v$ [$cm^{-1}$] = 2371, 2354, 1649, 1518, 1288, 1243, 1107.

**1,1,1,3,3,3-Hexafluoropropan-2-yl 5-((3,4-dimethoxyphenethyl)amino)-5-oxopentanoate (11r)**.

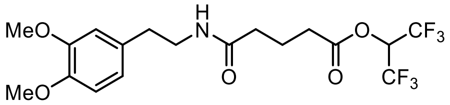


**11r**

**11r** was prepared according to **General procedure A**, starting from 3,4-dimethoxyphenethylamine (500 mg, 2.76 mmol, 1.0 equiv.) and glutaric anhydride (315 mg, 2.76 mmol, 1.0 equiv.) in DMF (15.0 mL). The reaction mixture was subsequently treated with EDCI (1.05 g, 5.52 mmol, 2.0 equiv.), DMAP (67.5 mg, 0.552 mmol, 0.2 equiv.) and HFIP (1.46 mL, 13.8 mmol, 5.0 equiv.). Purification by silica gel column chromatography (pentane/EtOAc = 5:2) afforded **11r** (670 mg, 1.42 mmol, 55%) as a white solid.

**$^1$H NMR** (500 MHz, $CDCl_3$) δ [ppm] = 6.79 (d, $J$ = 8.7 Hz, 1H), 6.72–6.67 (m, 2H), 5.77–5.72 (m, 1H), 5.54 (s, 1H), 3.85 (d, $J$ = 3.5 Hz, 6H), 3.49 (td, $J$ = 7.0, 5.9 Hz, 2H),

2.74 (t, $J$ = 7.0 Hz, 2H), 2.57 (t, $J$ = 7.2 Hz, 2H), 2.18 (t, $J$ = 7.2 Hz, 2H), 2.04–1.94 (m, 2H).

**$^{13}$C NMR** (126 MHz, $CDCl_3$) δ [ppm] = 171.5, 170.0, 149.2, 147.9, 131.3, 120.7, 120.5 (q, $J$ = 279.6 Hz), 111.9, 111.5, 67.0–65.9 (m), 56.0, 55.9, 40.8, 35.3, 34.8, 32.4, 20.4.

**$^{19}$F NMR** (565 MHz, $CDCl_3$) δ [ppm] = –73.22 (d, $J$ = 6.4 Hz).

**HRMS-ESI**: calcd. for $C_{18}H_{21}F_6NO_5Na$ [M + Na]$^+$: 468.1216; found: 468.1217.

**FT-IR:** $v$ [cm$^{-1}$] = 2942, 1682, 1602, 1513, 1377, 1341, 1272, 1162, 752.

**1,1,1,3,3,3-Hexafluoropropan-2-yl 2-(2-((3,4-dimethoxyphenethyl)amino)-2-oxo-ethyl)benzoate (11s)**.

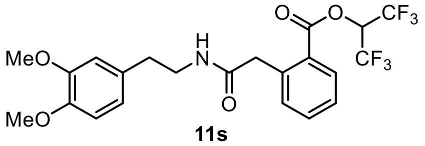

**11s**

**11s** was prepared according to **General procedure A**, starting from 3,4-dimethoxyphenethylamine (300 mg, 1.66 mmol, 1.0 equiv.) and homophthalic anhydride (268 mg, 1.66 mmol, 1.0 equiv.) in DMF (10.0 mL). The reaction mixture was subsequently treated with EDCI (633 mg, 3.31 mmol, 2.0 equiv.), DMAP (40.5 mg, 0.331 mmol, 0.2 equiv.) and HFIP (1.39 mL, 8.29 mmol, 5.0 equiv.). Purification by silica gel column chromatography (pentane/EtOAc = 5:2) afforded **11s** (189 mg, 0.383 mmol, 23%) as a white solid.

**$^{1}$H NMR** (600 MHz, $CDCl_3$) δ [ppm] = 8.00 (dd, $J$ = 7.9, 1.4 Hz, 1H), 7.55 (td, $J$ = 7.6, 1.4 Hz, 1H), 7.40 (dd, $J$ = 7.8, 1.3 Hz, 1H), 7.37 (td, $J$ = 7.7, 1.3 Hz, 1H), 6.69 (d, $J$ = 8.1 Hz, 1H), 6.63 (d, $J$ = 2.0 Hz, 1H), 6.58 (dd, $J$ = 8.1, 2.0 Hz, 1H), 6.07 (t, $J$ = 5.8 Hz, 1H), 5.96–5.90 (m, 1H), 3.81 (s, 2H), 3.80 (s, 3H), 3.79 (s, 3H), 3.42 (td, $J$ = 7.1, 5.7 Hz, 2H), 2.67 (t, $J$ = 7.1 Hz, 2H).

**$^{13}$C NMR** (151 MHz, $CDCl_3$) δ [ppm] = 169.8, 163.7, 148.9, 147.6, 138.7, 134.4, 132.7, 131.4, 131.3, 127.6, 125.7, 120.6, 120.5 (q, $J$ = 286.3 Hz), 111.9, 111.2, 67.3–66.4 (m), 55.8, 55.7, 41.9, 40.8, 35.0.

**$^{19}$F NMR** (565 MHz, $CDCl_3$) δ [ppm] = –72.99 (d, $J$ = 6.9 Hz).

**HRMS-ESI:** calcd. for $C_{22}H_{21}F_6NO_5Na$ [M + Na]$^+$: 516.1216; found: 516.1201.

**FT-IR:** $\nu$ [cm$^{-1}$] = 2937, 1755, 1652, 1515, 1237, 1194, 1109, 1096, 1025, 913, 715.

**1,1,1,3,3,3-Hexafluoropropan-2-yl 2-(2-oxo-2-(phenethylamino)ethyl)benzoate (11t).**

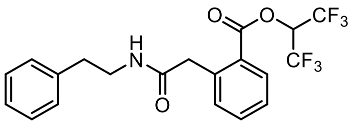

**11t**

**11t** was prepared according to **General procedure A**, starting from phenethylamine (250 mg, 2.07 mmol, 1.0 equiv.) and homophthalic anhydride (335 mg, 2.07 mmol, 1.0 equiv.) in DMF (10.0 mL). The reaction mixture was subsequently treated with EDCI (789 mg, 4.13 mmol, 2.0 equiv.), DMAP (50.5 mg, 0.413 mmol, 0.2 equiv.) and HFIP (1.09 mL, 10.3 mmol, 5.0 equiv.). Purification by silica gel column chromatography (pentane/EtOAc = 5:2) afforded **11t** (189 mg, 0.436 mmol, 21%) as a white solid.

**$^{1}$H NMR** (700 MHz, $CDCl_3$) δ [ppm] = 8.05 (dd, $J$ = 8.0, 1.5 Hz, 1H), 7.59 (td, $J$ = 7.6, 1.5 Hz, 1H), 7.45 (dd, $J$ = 7.8, 1.3 Hz, 1H), 7.41 (td, $J$ = 7.7, 1.3 Hz, 1H), 7.25–7.20 (m, 2H), 7.20–7.16 (m, 1H), 7.09–7.03 (m, 2H), 6.11 (t, $J$ = 5.9 Hz, 1H), 5.96–5.92 (m, 1H), 3.84 (s, 2H), 3.47 (td, $J$ = 7.0, 5.8 Hz, 2H), 2.75 (t, $J$ = 7.0 Hz, 2H).

**$^{13}$C NMR** (176 MHz, $CDCl_3$) δ [ppm] = 169.9, 163.8, 138.85, 138.78, 134.5, 132.8, 131.5, 128.7, 128.5, 127.7, 126.4, 125.8, 120.6 (q, $J$ = 282.8 Hz), 67.5–66.3 (m), 42.0, 40.8, 35.5.

**$^{19}$F NMR** (376 MHz, $CDCl_3$) δ [ppm] = –72.94 (d, $J$ = 12.5 Hz).

**HRMS-ESI:** calcd. for $C_{20}H_{17}F_6NO_3Na$ [M + Na]$^+$: 456.1005; found: 456.1012.

**FT-IR:** $\nu$ [cm$^{-1}$] = 3305, 1754, 1651, 1549, 1359, 1239, 1190, 1099, 1018, 914, 899, 714.

**1,1,1,3,3,3-Hexafluoropropan-2-yl 5-((2-(furan-3-yl)ethyl)amino)-5-oxopentanoate (11u).**

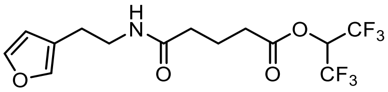

**11u**

**11u** was prepared according to **General procedure A**, starting from 2-(furan-3-

yl)ethan-1-amine (180 mg, 1.62 mmol, 1.0 equiv.) and glutaric anhydride (185 mg, 1.62 mmol, 1.0 equiv.) in DMF (5.0 mL). The reaction mixture was subsequently treated with EDCI (620 mg, 3.24 mmol, 2.0 equiv.), DMAP (39.6 mg, 0.324 mmol, 0.2 equiv.) and HFIP (857 µL, 8.11 mmol, 5.0 equiv.). Purification by silica gel column chromatography (pentane/EtOAc = 5:2) afforded **11u** (200 mg, 0.533 mmol, 33%) as a light yellow solid.

**$^1$H NMR** (700 MHz, $CDCl_3$) δ [ppm] = 7.36 (t, *J* = 1.7 Hz, 1H), 7.25–7.24 (m, 1H), 6.27 (dd, *J* = 1.9, 0.9 Hz, 1H), 5.77–5.73 (m, 1H), 5.63 (s, 1H), 3.44 (td, *J* = 6.9, 5.8 Hz, 2H), 2.62 (td, *J* = 6.9, 1.0 Hz, 2H), 2.56 (t, *J* = 7.3 Hz, 2H), 2.20 (t, *J* = 7.3 Hz, 2H), 2.01–1.97 (m, 2H).

**$^{13}$C NMR** (176 MHz, $CDCl_3$) δ [ppm] = 171.5, 170.0, 143.4, 139.7, 121.8, 120.5 (q, *J* = 282.0 Hz), 110.8, 66.9–66.1 (m), 39.5, 34.8, 32.4, 25.0, 20.4.

**$^{19}$F NMR** (565 MHz, $CDCl_3$) δ [ppm] = –73.29 (d, *J* = 6.5 Hz).

**HRMS-ESI**: calcd. for $C_{14}H_{15}F_6NO_4Na$ $[M + Na]^+$: 398.0797; found: 398.0811.

**FT-IR:** *v* [$cm^{-1}$] = 2920, 2850, 1780, 1659, 1447, 1386, 1288, 1227, 1198, 1110, 924, 789.

**1,1,1,3,3,3-Hexafluoropropan-2-yl 5-((2-(furan-2-yl)ethyl)amino)-5-oxopentanoate (11v)**.

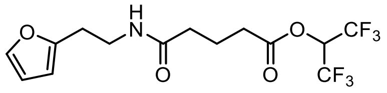

**11v**

**11v** was prepared according to **General procedure A**, starting from 2-(furan-2-yl)ethan-1-amine (180 mg, 1.62 mmol, 1.0 equiv.) and glutaric anhydride (185 mg, 1.62 mmol, 1.0 equiv.) in DMF (5.0 mL). The reaction mixture was subsequently treated with EDCI (620 mg, 3.24 mmol, 2.0 equiv.), DMAP (39.6 mg, 0.324 mmol, 0.2 equiv.) and HFIP (857 µL, 8.11 mmol, 5.0 equiv.). Purification by silica gel column chromatography (pentane/EtOAc = 3:2) afforded **11v** (250 mg, 0.667 mmol, 41%) as a light yellow solid.

**$^1$H NMR** (700 MHz, $CDCl_3$) δ [ppm] = 7.33 (dd, *J* = 1.9, 0.8 Hz, 1H), 6.32–6.28 (m,

1H), 6.07–6.06 (m, 1H), 5.78–5.74 (m, 1H), 5.58 (s, 1H), 3.59–3.49 (m, 2H), 2.85 (td, $J$ = 6.5, 0.8 Hz, 2H), 2.58 (t, $J$ = 7.3 Hz, 2H), 2.22 (t, $J$ = 7.2 Hz, 2H), 2.06–1.97 (m, 2H).

**$^{13}$C NMR** (176 MHz, $CDCl_3$) δ [ppm] = 171.5, 170.1, 153.1, 141.8, 120.6 (d, $J$ = 281.9 Hz), 110.5, 106.6, 66.7–66.3 (m), 38.3, 34.9, 32.4, 28.2, 20.4.

**$^{19}$F NMR** (376 MHz, $CDCl_3$) δ [ppm] = –73.19 (d, $J$ = 6.0 Hz).

**HRMS-ESI**: calcd. for $C_{14}H_{15}F_6NO_4Na$ [M + Na]$^+$: 398.0797; found: 398.0786.

**FT-IR:** $\nu$ [cm$^{-1}$] = 3314, 2930, 1781, 1647, 1551, 1386, 1288, 1232, 1200, 1111, 907, 780.

**1,1,1,3,3,3-Hexafluoropropan-2-yl 5-oxo-5-((2-(thiophen-3-yl)ethyl)amino)pentanoate (11w)**.

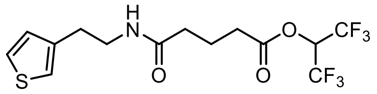

**11w**

**11w** was prepared according to **General procedure A**, starting from 2-(thiophen-3-yl)ethan-1-amine (150 mg, 1.18 mmol, 1.0 equiv.) and glutaric anhydride (135 mg, 1.18 mmol, 1.0 equiv.) in DMF (5.0 mL). The reaction mixture was subsequently treated with EDCI (451 mg, 2.36 mmol, 2.0 equiv.), DMAP (28.9 mg, 0.236 mmol, 0.2 equiv.) and HFIP (624 µL, 5.91 mmol, 5.0 equiv.). Purification by silica gel column chromatography (pentane/EtOAc = 3:2) afforded **11w** (200 mg, 0.512 mmol, 43%) as a white solid.

**$^1$H NMR** (500 MHz, $CDCl_3$) δ [ppm] = 7.29–7.24 (m, 1H), 6.97–6.98 (m, 1H), 6.93 (dd, $J$ = 4.9, 1.3 Hz, 1H), 5.77–5.71 (m, 1H), 5.63 (s, 1H), 3.53–3.48 (m, 2H), 2.83 (t, $J$ = 6.9 Hz, 2H), 2.54 (td, $J$ = 7.2, 0.8 Hz, 2H), 2.18 (t, $J$ = 7.2 Hz, 2H), 2.05–1.93 (m, 2H).

**$^{13}$C NMR** (126 MHz, $CDCl_3$) δ [ppm] = 171.5, 170.0, 139.1, 128.1, 126.2, 121.5, 120.5 (q, $J$ = 282.0 Hz), 67.0–65.9 (m), 39.9, 34.8, 32.3, 30.3, 20.4.

**$^{19}$F NMR** (471 MHz, $CDCl_3$) δ [ppm] = –73.24 (d, $J$ = 6.4 Hz).

**HRMS-ESI**: calcd. for $C_{14}H_{15}F_6NO_3SK$ [M + K]$^+$: 430.0309; found: 430.0328.

**FT-IR:** $\nu$ [cm$^{-1}$] = 3319, 1770, 1638, 1534, 1385, 1281, 1201, 1106, 720.

**1,1,1,3,3,3-Hexafluoropropan-2-yl 5-((2-(benzo[b]thiophen-3-yl)ethyl)amino)-5-oxopentanoate (11x)**.

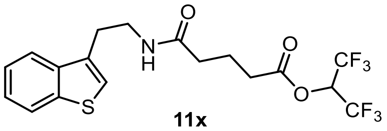


**11x**

**11x** was prepared according to **General procedure A**, starting from 2-(benzo[*b*]thiophen-3-yl)ethan-1-amine (140 mg, 0.791 mmol, 1.0 equiv.) and glutaric anhydride (90.2 mg, 0.791 mmol, 1.0 equiv.) in DMF (3.0 mL). The reaction mixture was subsequently treated with EDCI (302 mg, 1.58 mmol, 2.0 equiv.), DMAP (19.3 mg, 0.158 mmol, 0.2 equiv.) and HFIP (418 µL, 3.95 mmol, 5.0 equiv.). Purification by silica gel column chromatography (pentane/EtOAc = 2:1) afforded **11x** (183 mg, 0.415 mmol, 52%) as a white solid.

**$^{1}$H NMR** (700 MHz, $CDCl_3$) δ [ppm] = 7.86 (dt, $J$ = 7.8, 1.0 Hz, 1H), 7.77 (dt, $J$ = 8.0, 1.0 Hz, 1H), 7.39 (ddd, $J$ = 8.0, 7.0, 1.2 Hz, 1H), 7.35 (ddd, $J$ = 8.2, 7.0, 1.3 Hz, 1H), 7.14 (s, 1H), 5.80–5.73 (m, 2H), 3.60 (td, $J$ = 7.0, 5.9 Hz, 2H), 3.05 (td, $J$ = 6.9, 1.0 Hz, 2H), 2.55 (t, $J$ = 7.3 Hz, 2H), 2.17 (t, $J$ = 7.3 Hz, 2H), 2.01–1.97 (m, 2H).

**$^{13}$C NMR** (176 MHz, $CDCl_3$) δ [ppm] = 171.6, 170.0, 140.6, 138.8, 133.4, 124.5, 124.2, 123.0, 122.5, 121.6, 120.5 (q, $J$ = 282.7 Hz), 66.9–66.1 (m), 39.1, 34.8, 32.4, 28.7, 20.3.

**$^{19}$F NMR** (376 MHz, $CDCl_3$) δ [ppm] = –73.20. (It should be a doublet peak, but due to the resolution, only singlet peak was observed.)

**HRMS-ESI:** calcd. for $C_{18}H_{17}F_6NO_3SNa$ [M + Na]$^+$: 464.0725; found: 464.0745.

**FT-IR:** $\nu$ [cm$^{-1}$] = 2928, 2850, 1736, 1698, 1215, 747.

**1,1,1,3,3,3-Hexafluoropropan-2-yl 5-((2-(6-methoxy-1*H*-indol-3-yl)ethyl)amino)-3-methyl-5-oxopentanoate (11y)**.

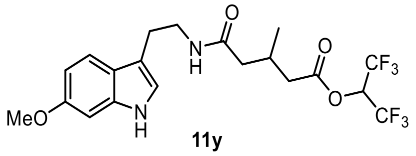


**11y**

**11y** was prepared according to **General procedure A**, starting from 6-methoxytrytamine (100 mg, 0.526 mmol, 1.0 equiv.) and glutaric anhydride (67.4 mg, 0.526 mmol, 1.0 equiv.) in DMF (3.0 mL). The reaction mixture was subsequently treated with EDCI (201 mg, 1.05 mmol, 2.0 equiv.), DMAP (12.9 mg, 0.105 mmol, 0.2 equiv.) and HFIP (278 µL, 2.63 mmol, 5.0 equiv.). Purification by silica gel column chromatography (pentane/EtOAc = 3:2) afforded **11y** (150 mg, 0.320 mmol, 61%) as a white solid.

**$^1$H NMR** (700 MHz, $CDCl_3$) δ [ppm] = 8.19 (s, 1H), 7.44 (d, *J* = 8.6 Hz, 1H), 6.89–6.87 (m, 1H), 6.84 (d, *J* = 2.3 Hz, 1H), 6.79 (dd, *J* = 8.6, 2.3 Hz, 1H), 5.79–5.75 (m, 1H), 5.66 (s, 1H), 3.83 (d, *J* = 0.7 Hz, 3H), 3.58 (dt, *J* = 8.5, 6.8 Hz, 2H), 2.95–2.89 (m, 2H), 2.65–2.59 (m, 1H), 2.52–2.48 (m, 1H), 2.48–2.34 (m, 1H), 2.13 (dd, *J* = 14.3, 6.9 Hz, 1H), 2.03 (ddd, *J* = 14.5, 7.4, 1.7 Hz, 1H), 0.99 (d, *J* = 6.8 Hz, 3H).

**$^{13}$C NMR** (176 MHz, $CDCl_3$) δ [ppm] = 171.1, 169.4, 156.8, 137.3, 121.9, 120.9, 120.5 (q, *J* = 282.4 Hz), 119.3, 112.9, 109.6, 94.9, 66.8–66.0 (m), 55.8, 42.8, 39.8, 39.5, 27.9, 25.5, 19.4.

**$^{19}$F NMR** (565 MHz, $CDCl_3$) δ [ppm] = –73.11 (d, *J* = 7.7 Hz).

**HRMS-ESI:** calcd. for $C_{20}H_{22}F_6N_2O_4Na$ $[M + Na]^+$: 491.1374; found: 491.1376.

**FT-IR:** *ν* [cm$^{-1}$] 2958, 1776, 1648, 1386, 1288, 1200, 1110, 937, 812.

2.1.3. Additional substrates

**1,1,1,3,3,3-Hexafluoropropan-2-yl 4-((2-(1*H*-indol-3-yl)ethyl)amino)-4-oxobutanoate (S1).**

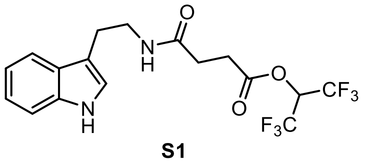

**S1**

**S1** was prepared according to **General procedure A**, starting from tryptamine (250 mg, 1.56 mmol, 1.0 equiv.) and succinic anhydride (156 mg, 1.56 mmol, 1.0 equiv.) in DMF (5.0 mL). The reaction mixture was subsequently treated with EDCI (328 mg, 1.72 mmol, 2.0 equiv.), DMAP (38.2 mg, 0.313 mmol, 0.2 equiv.) and HFIP (825 µL, 7.81 mmol, 5.0 equiv.). Purification by silica gel column chromatography

(pentane/EtOAc = 3:2) afforded **S1** (286 mg, 0.698 mmol, 45%) as a white solid.

**$^{1}$H NMR** (600 MHz, $CDCl_3$) δ [ppm] = 8.22 (s, 1H), 7.59 (dd, *J* = 7.9, 1.1 Hz, 1H), 7.37 (dt, *J* = 8.1, 0.9 Hz, 1H), 7.22 (ddd, *J* = 8.2, 7.0, 1.2 Hz, 1H), 7.13 (ddd, *J* = 8.0, 7.0, 1.0 Hz, 1H), 7.01 (d, *J* = 2.3 Hz, 1H), 5.79–5.75 (m, 1H), 5.63 (d, *J* = 5.6 Hz, 1H), 3.60 (q, *J* = 6.5 Hz, 2H), 2.96 (td, *J* = 6.8, 0.9 Hz, 2H), 2.84 (t, *J* = 6.9 Hz, 2H), 2.44 (t, *J* = 6.9 Hz, 2H).

**$^{13}$C NMR** (151 MHz, $CDCl_3$) δ [ppm] = 170.2, 170.0, 136.6, 127.4, 122.4, 122.2, 120.5 (d, *J* = 283.0 Hz), 119.6, 118.8, 112.9, 111.4, 67.2–66.3 (m), 40.1, 30.4, 28.7, 25.3.

**$^{19}$F NMR** (565 MHz, $CDCl_3$) δ [ppm] = –73.14 (d, *J* = 6.6 Hz).

**HRMS-ESI:** calcd. for $C_{17}H_{16}F_6N_2O_3Na$ $[M + Na]^+$: 433.0957; found: 433.0974.

**FT-IR:** *ν* [$cm^{-1}$] = 3422, 3328, 1754, 1633, 1384, 1286, 1201, 1108, 905, 738.

**1,1,1,3,3,3-Hexafluoropropan-2-yl 5-(((1H-indol-3-yl)methyl)amino)-5-oxopent-anoate (S2)**.

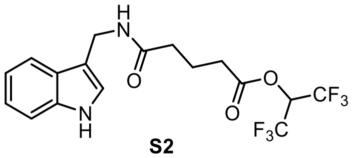


**S2**

**S2** was prepared according to **General procedure A**, starting from 1*H*-indole-3-methanamine (310 mg, 2.12 mmol, 1.0 equiv.) and glutaric anhydride (242 mg, 2.12 mmol, 1.0 equiv.) in DMF (10.0 mL). The reaction mixture was subsequently treated with EDCI (811 mg, 4.25 mmol, 2.0 equiv.), DMAP (51.9 mg, 0.425 mmol, 0.2 equiv.) and HFIP (1.12 mL, 10.6 mmol, 5.0 equiv.). Purification by silica gel column chromatography (pentane/EtOAc = 3:2) afforded **S2** (300 mg, 0.732 mmol, 34%) as a white solid.

**$^{1}$H NMR** (700 MHz, $CDCl_3$) δ [ppm] = 8.28 (s, 1H), 7.62–7.60 (m, 1H), 7.39 (dt, *J* = 8.1, 0.9 Hz, 1H), 7.23 (ddd, *J* = 8.1, 7.0, 1.2 Hz, 1H), 7.18–7.12 (m, 2H), 5.79–5.72 (m, 1H), 5.70 (s, 1H), 4.62 (dd, *J* = 5.2, 0.8 Hz, 2H), 2.60 (t, *J* = 7.3 Hz, 2H), 2.23 (t, *J* = 7.2 Hz, 2H), 2.07–2.03 (m, 2H).

**$^{13}$C NMR** (176 MHz, $CDCl_3$) δ [ppm] = 171.3, 170.1, 136.5, 126.6, 123.4, 122.7, 120.5 (d, *J* = 286.2 Hz), 120.1, 118.8, 112.7, 111.5, 66.9–66.1 (m), 35.3, 34.9, 32.5, 20.5.

$^{19}$**F NMR** (565 MHz, $CDCl_3$) δ [ppm] = –73.20 (d, *J* = 6.3 Hz).

**HRMS-ESI:** calcd. for $C_{17}H_{16}F_6N_2O_3Na$ $[M + Na]^+$: 433.0957; found: 433.0972.

**FT-IR:** *ν* [cm$^{-1}$] = 3259, 1738, 1660, 1364, 1204, 1108, 744.

**1,1,1,3,3,3-Hexafluoropropan-2-yl 10-((2-(1H-indol-3-yl)ethyl)amino)-10-oxodecanoate (S3)**.

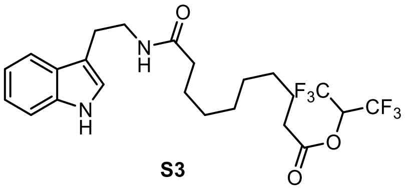

**S3**

**S3** was prepared according to **General procedure A**, starting from tryptamine (200 mg, 1.25 mmol, 1.0 equiv.) and oxacycloundecane-2,11-dione (230 mg, 1.25 mmol, 1.0 equiv.) in DMF (5.0 mL). The reaction mixture was subsequently treated with EDCI (477 mg, 2.50 mmol, 2.0 equiv.), DMAP (30.5 mg, 0.250 mmol, 0.2 equiv.) and HFIP (660 μL, 6.25 mmol, 5.0 equiv.). Purification by silica gel column chromatography (pentane/EtOAc = 3:2) afforded **S3** (150 mg, 0.304 mmol, 24%) as a white solid.

$^{1}$**H NMR** (500 MHz, $CDCl_3$) δ [ppm] = 8.72 (s, 1H), 7.60 (dd, *J* = 8.0, 1.1 Hz, 1H), 7.39–7.34 (m, 1H), 7.20 (ddd, *J* = 8.1, 7.0, 1.2 Hz, 1H), 7.11 (ddd, *J* = 8.0, 7.0, 1.0 Hz, 1H), 6.98 (d, *J* = 2.0 Hz, 1H), 5.85–5.67 (m, 2H), 3.62–3.58 (m, 2H), 2.97 (t, *J* = 6.8 Hz, 2H), 2.49 (t, *J* = 7.4 Hz, 2H), 2.10 (t, *J* = 7.6 Hz, 2H), 1.69–1.65 (m, 2H), 1.62–1.56 (m, 2H), 1.32–1.25 (m, 8H).

$^{13}$**C NMR** (126 MHz, $CDCl_3$) δ [ppm] = 173.4, 170.5, 136.6, 127.5, 122.3, 122.1, 120.5 (q, *J* = 282.5 Hz), 119.4, 118.7, 112.8, 111.5, 66.9–65.8 (m), 39.9, 36.8, 33.3, 29.2, 29.1, 28.9, 28.7, 25.7, 25.4, 24.5.

$^{19}$**F NMR** (471 MHz, $CDCl_3$) δ [ppm] = –73.26 (d, *J* = 5.7 Hz).

**HRMS-ESI:** calcd. for $C_{23}H_{28}F_6N_2O_3Na$ $[M + Na]^+$: 517.1896; found: 517.1921.

**FT-IR:** *ν* [cm$^{-1}$] = 2939, 2921, 2146, 2042, 1779, 1649, 1289, 1228, 1202, 1111, 760.

**1,1,1,3,3,3-Hexafluoropropan-2-yl (*S*)-5-((3-(4-hydroxyphenyl)-1-methoxy-1-oxopropan-2-yl)amino)-5-oxopentanoate (S4)**.

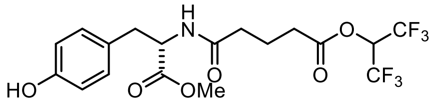


**S4**

**S4** was prepared according to **General procedure A**, starting from (*S*)-tyrosine methyl ester (300 mg, 1.54 mmol, 1.0 equiv.) and glutaric anhydride (175 mg, 1.54 mmol, 1.0 equiv.) in DMF (10.0 mL). The reaction mixture was subsequently treated with EDCI (588 mg, 3.01 mmol, 2.0 equiv.), DMAP (37.6 mg, 0.308 mmol, 0.2 equiv.) and HFIP (813 µL, 7.69 mmol, 5.0 equiv.). Purification by silica gel column chromatography (pentane/EtOAc = 3:2) afforded **S4** (170 mg, 0.370 mmol, 24%) as a white solid.

**$^1$H NMR** (500 MHz, $CDCl_3$) δ [ppm] = 7.04 (s, 1H), 6.95–6.89 (m, 2H), 6.75–6.69 (m, 2H), 6.15 (d, *J* = 8.1 Hz, 1H), 5.79–5.74 (m, 1H), 4.85 (ddd, *J* = 8.1, 6.4, 5.6 Hz, 1H), 3.73 (s, 3H), 3.07 (dd, *J* = 14.1, 5.5 Hz, 1H), 2.95 (dd, *J* = 14.1, 6.5 Hz, 1H), 2.51 (td, *J* = 7.3, 1.3 Hz, 2H), 2.25 (t, *J* = 7.5 Hz, 2H), 1.99–1.93 (m, 2H).

**$^{13}$C NMR** (126 MHz, $CDCl_3$) δ [ppm] = 172.5, 172.0, 170.0, 155.7, 130.3, 127.0, 120.5 (q, *J* = 284.3 Hz), 115.7, 67.1–66.0 (m), 53.4, 52.6, 37.2, 34.7, 32.2, 20.2.

**$^{19}$F NMR** (471 MHz, $CDCl_3$) δ [ppm] = –73.24 (d, *J* = 6.7 Hz).

**HRMS-ESI:** calcd. for $C_{18}H_{19}F_6NO_6K$ $[M + K]^+$: 498.0749; found: 498.0774.

**FT-IR:** *ν* [cm$^{-1}$] = 2960, 1780, 1740, 1651, 1516, 1286, 1223, 1198, 1108, 905, 733.

$[\alpha]_D^{26}$ = +19.0 (*c* = 0.4, $CHCl_3$).

**1,1,1,3,3,3-Hexafluoropropan-2-yl 5-oxo-5-(phenethylamino)pentanoate (S5)**.

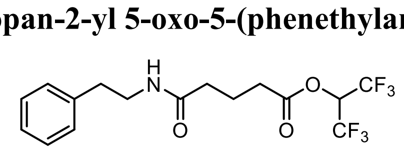


**S5**

**S5** was prepared according to **General procedure A**, starting from phenethylamine (200 mg, 1.65 mmol, 1.0 equiv.) and glutaric anhydride (188 mg, 1.65 mmol, 1.0 equiv.) in DMF (5.0 mL). The reaction mixture was subsequently treated with EDCI (631 mg, 3.31 mmol, 2.0 equiv.), DMAP (40.4 mg, 0.331 mmol, 0.2 equiv.) and HFIP (873 µL,

8.26 mmol, 5.0 equiv.). Purification by silica gel column chromatography (pentane/EtOAc = 5:2) afforded **S5** (200 mg, 0.519 mmol, 31%) as a white solid.

**$^1$H NMR** (500 MHz, $CDCl_3$) δ [ppm] = 7.33–7.28 (m, 2H), 7.26–7.21 (m, 1H), 7.18 (dt, *J* = 7.9, 1.7 Hz, 2H), 7.79–7.73 (m, 1H), 5.54 (s, 1H), 3.58–3.48 (m, 2H), 2.81 (td, *J* = 7.0, 1.7 Hz, 2H), 2.54 (td, *J* = 7.3, 1.8 Hz, 2H), 2.18 (td, *J* = 7.2, 1.8 Hz, 2H), 2.02–1.97 (m, 2H).

**$^{13}$C NMR** (126 MHz, $CDCl_3$) δ [ppm] = 171.5, 170.1, 138.8, 128.84, 128.78, 126.7, 120.5 (d, *J* = 282.0 Hz), 67.0–65.9 (m), 40.6, 35.7, 34.8, 32.4, 20.4.

**$^{19}$F NMR** (471 MHz, $CDCl_3$) δ [ppm] = –73.22 (d, *J* = 6.3 Hz).

**HRMS-ESI:** calcd. for $C_{16}H_{17}F_6NO_3Na$ $[M + Na]^+$: 408.1005; found: 408.1007.

**FT-IR:** *ν* [cm$^{-1}$] = 3309, 1967, 1645, 1543, 1282, 1268, 1107.

**1,1,1,3,3,3-Hexafluoropropan-2-yl 5-((4-methoxyphenethyl)amino)-5-oxopentanoate (S6)**.

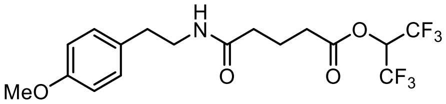


**S6**

**S6** was prepared according to **General procedure A**, starting from 4-methoxyphenethylamine (200 mg, 1.32 mmol, 1.0 equiv.) and glutaric anhydride (151 mg, 1.32 mmol, 1.0 equiv.) in DMF (5.0 mL). The reaction mixture was subsequently treated with EDCI (506 mg, 2.65 mmol, 2.0 equiv.), DMAP (32.4 mg, 0.365 mmol, 0.2 equiv.) and HFIP (699 μL, 6.62 mmol, 5.0 equiv.). Purification by silica gel column chromatography (pentane/EtOAc = 5:2) afforded **S6** (225 mg, 0.542 mmol, 41%) as a white solid.

**$^1$H NMR** (500 MHz, $CDCl_3$) δ [ppm] = 7.08 (d, *J* = 8.6 Hz, 2H), 6.83 (d, *J* = 8.6 Hz, 2H), 5.78–5.73 (m, 1H), 5.63 (s, 1H), 3.77 (s, 3H), 3.46 (td, *J* = 7.0, 5.8 Hz, 2H), 2.74 (t, *J* = 7.0 Hz, 2H), 2.55 (t, *J* = 7.3 Hz, 2H), 2.18 (t, *J* = 7.2 Hz, 2H), 2.01–1.97 (m,, 2H).

**$^{13}$C NMR** (126 MHz, $CDCl_3$) δ [ppm] = 171.5, 170.0, 158.4, 130.8, 129.8, 120.5 (q, *J* = 279.4 Hz), 114.1, 67.0–65.9 (m), 55.30, 55.28, 40.8, 34.8, 32.4, 20.4.

**$^{19}$F NMR** (471 MHz, $CDCl_3$) δ [ppm] = –73.25 (d, *J* = 8.6 Hz).

**HRMS-ESI:** calcd. for $C_{17}H_{19}F_6NO_4Na$ [M + Na]$^+$: 438.1110; found: 438.1109.

**FT-IR:** *ν* [cm$^{-1}$] 3299, 1767, 1636, 1542, 1282, 1257, 1202, 1107, 1038.

**1,1,1,3,3,3-Hexafluoropropan-2-yl 5-oxo-5-((2-(pyridin-2-yl)ethyl)amino)pentanoate (S7)**.

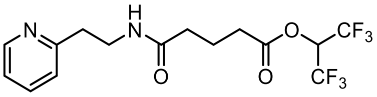


**S7**

**S7** was prepared according to **General procedure A**, starting from 2-(pyridin-2-yl)ethan-1-amine (300 mg, 2.46 mmol, 1.0 equiv.) and glutaric anhydride (280 mg, 2.46 mmol, 1.0 equiv.) in DMF (10.0 mL). The reaction mixture was subsequently treated with EDCI (939 mg, 4.92 mmol, 2.0 equiv.), DMAP (60.1 mg, 0.492 mmol, 0.2 equiv.) and HFIP (1.30 mL, 12.3 mmol, 5.0 equiv.). Purification by silica gel column chromatography (pentane/EtOAc = 3:2) afforded **S7** (670 mg, 1.51 mmol, 61%) as a colorless oil.

**$^1$H NMR** (500 MHz, $CDCl_3$) δ [ppm] = 8.44 (ddd, *J* = 4.9, 1.9, 0.9 Hz, 1H), 7.56 (td, *J* = 7.7, 1.9 Hz, 1H), 7.14–7.06 (m, 2H), 6.75 (s, 1H), 5.74–5.69 (m,, 1H), 3.60 (dt, *J* = 6.6, 5.7 Hz, 2H), 2.98–2.89 (m, 2H), 2.50 (t, *J* = 7.4 Hz, 2H), 2.17 (t, *J* = 7.2 Hz, 2H), 2.02–1.88 (m, 2H).

**$^{13}$C NMR** (126 MHz, $CDCl_3$) δ [ppm] = 171.5, 170.0, 159.6, 149.1, 136.7, 123.5, 121.6, 120.4 (q, *J* = 279.4 Hz), 66.9–65.8 (m), 38.7, 36.9, 34.9, 32.4, 20.4.

**$^{19}$F NMR** (471 MHz, $CDCl_3$) δ [ppm] = –73.19 (d, *J* = 6.5 Hz).

**HRMS-ESI:** calcd. for $C_{15}H_{16}F_6N_2O_3Na$ [M + Na]$^+$: 409.0957; found: 409.0972.

**FT-IR:** *ν* [cm$^{-1}$] = 2950, 1781, 1652, 1288, 1230, 1199, 1110, 747.

## 2.2. One-pot annulation reaction

### 2.2.1. Reaction optimization

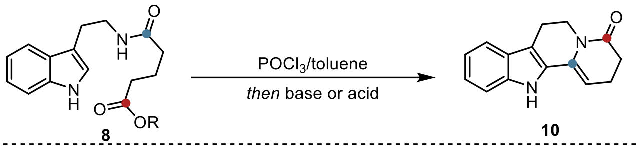


**8** **10**

**8a:** R = methyl
**8b:** R = 4-nitrophenyl
**8c:** R = 2-propyl
**8d:** R = 2-hexafluoropropyl
**8e:** R = 2,2,2-trifluoroethyl
**8f:** R = 2,2,2-trifluoro-1-methylethyl
**8g:** R = pentafluorophenyl
**8h:** R = prenylyl
**8i:** R = diphenylmethyl
**8j:** R = cyclohexylyl
**8k:** R = cyclohexylthio
**8l:** R = *t*-butyl

| entry | substrate | base or acid | yield |
|---|---|---|---|
| 1 | **8a** | $K_2CO_3$ | 18 |
| 2 | **8a** | $Cs_2CO_3$ | 4 |
| 3 | **8a** | KOH | 6 |
| 4 | **8a** | TMSOK | 6 |
| 5 | **8a** | KO*t*-Bu | 15 |
| 6 | **8a** | $ZrCl_4$ | N.D.[a] |
| 7 | **8a** | $In(OTf)_2$ | N.D. |
| 8 | **8a** | $Sc(OTf)_3$ | N.D. |
| 9 | **8a** | Ti(O*i*-Pr)$_4$ | N.D. |
| 10 | **8a** | *p*-TsOH | N.D. |
| 11 | **8b** | $K_2CO_3$, $Bu_4NBr$ | 62 |
| 12 | **8c** | $K_2CO_3$, $Bu_4NBr$ | 18 |
| 13 | **8d** | $K_2CO_3$, $Bu_4NBr$ | 90 |
| 14 | **8e** | $K_2CO_3$, $Bu_4NBr$ | 42 |
| 15 | **8f** | $K_2CO_3$, $Bu_4NBr$ | 41 |
| 16 | **8g** | $K_2CO_3$, $Bu_4NBr$ | 65 |
| 17 | **8h** | $K_2CO_3$, $Bu_4NBr$ | 20 |
| 18 | **8i** | $K_2CO_3$, $Bu_4NBr$ | 47 |
| 19 | **8j** | $K_2CO_3$, $Bu_4NBr$ | 32 |
| 20 | **8k** | $K_2CO_3$, $Bu_4NBr$ | 42 |
| 21 | **8l** | $K_2CO_3$, $Bu_4NBr$ | 30 |

[a] Not determined

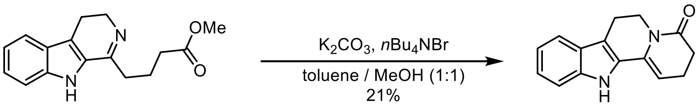


**9a** **10**

### 2.2.2. Substrate scope

## General procedure C

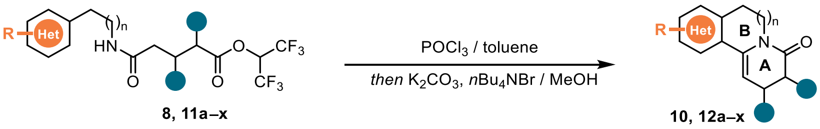


**8, 11a–x** **10, 12a–x**

To a solution of the substrate **8** or **11a–x** (1.0 equiv.) in toluene was added $POCl_3$ (1.0 equiv.) dropwise. Then the mixture was heated to 115 ºC (oil bath temperature) until the starting material was fully consumed. After allowing the mixture to cool down to 80 ºC (oil bath temperature), the same volume of MeOH, $K_2CO_3$ (10.0 equiv.) and

$n$Bu$_4$NBr (0.1 equiv.) were added subsequently. Afterwards the resulting mixture was stirred overnight at 80 ºC, cooled to room temperature, filtered through Celite®, concentrated under reduced pressure, and purified by silica gel or neutral aluminium oxide column chromatography.

(Note: It is not necessary to perform the reaction under argon.)

**Methyl 4-(4,9-dihydro-3*H*-pyrido[3,4-*b*]indol-1-yl)butanoate (9)**.

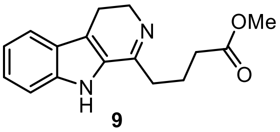


**9**

To a solution of the substrate **8a** (100 mg, 0.347 mmol, 1.0 equiv.) in toluene (3.0 mL), was added POCl$_3$ (31.7 µL, 0.347 mmol, 1.0 equiv.). The mixture was heated to 115 ºC (oil bath temperature) until the starting material was fully consumed. Then the mixture was concentrated under reduced pressure and purified by silica gel flash column chromatography (DCM/MeOH= 10:1). **9** (79.0 mg, 0.293 mmol, 84%) was afforded as a dark yellow solid.

**$^1$H NMR** (500 MHz, CDCl$_3$) δ [ppm] = 11.39 (s, 1H), 7.63 (d, $J$ = 8.2 Hz, 1H), 7.59 (dd, $J$ = 8.5, 0.9 Hz, 1H), 7.46 (ddd, $J$ = 8.4, 6.9, 1.2 Hz, 1H), 7.22 (ddd, $J$ = 8.1, 6.9, 1.4 Hz, 1H), 4.02 (t, $J$ = 8.8 Hz, 2H), 3.76 (s, 3H), 3.36–3.28 (m, 2H), 3.26–3.19 (m, 2H), 2.66–2.57 (m, 2H), 2.24–2.10 (m, 2H).

**$^{13}$C NMR** (126 MHz, CDCl$_3$) δ [ppm] = 173.9, 169.3, 141.7, 129.1, 125.7, 124.3, 124.0, 122.0, 121.4, 114.1, 52.0, 42.4, 32.8, 31.7, 23.4, 19.5.

**HRMS-ESI:** calcd. for $C_{16}H_{19}N_2O_2$ $[M + H]^+$: 271.1441; found: 271.1455.

**FT-IR:** $v$ [cm$^{-1}$] = 2925, 1733, 1630, 1554, 1434, 1336, 1229, 747.

**2,6,7,12-Tetrahydroindolo[2,3-*a*]quinolizin-4(3*H*)-one (10)**.

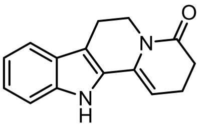


**10**

**10** was prepared according to **General procedure C**, starting from **8d** (100 mg,

0.236 mmol, 1.0 equiv.) and $POCl_3$ (21.5 μL, 0.236 mmol, 1.0 equiv.) in toluene (3.0 mL). The reaction mixture was subsequently treated with MeOH (3.0 mL), $K_2CO_3$ (326 mg, 2.36 mmol, 10 equiv.) and *n*$Bu_4$NBr (7.6 mg, 24.0 μmol, 0.1 equiv.). Purification by silica gel column chromatography (pentane/EtOAc = 2:1) afforded **10** (51.0 mg, 0.214 mmol, 90%) as a yellow solid.

Scale-up reaction starting from **8d** (2.00 g, 4.72 mmol, 1.0 equiv.) and $POCl_3$ (430 μL, 4.72 mmol, 1.0 equiv.) in toluene (80 mL). The reaction mixture was subsequently treated with MeOH (80 mL), $K_2CO_3$ (6.52 g, 47.2 mmol, 10 equiv.) and *n*$Bu_4$NBr (152 mg, 0.472 mmol, 0.1 equiv.). Purification by silica gel column chromatography (pentane/EtOAc = 2:1) afforded **10** (910 mg, 3.82 mmol, 81%) as a yellow solid.

**$^1$H NMR** (500 MHz, $CDCl_3$) δ [ppm] = 7.97 (s, 1H), 7.53–7.51 (m, 1H), 7.34 (dt, *J* = 8.2, 0.9 Hz, 1H), 7.23 (ddd, *J* = 8.2, 7.0, 1.1 Hz, 1H), 7.12 (ddd, *J* = 8.0, 7.1, 1.0 Hz, 1H), 5.49 (t, *J* = 5.0 Hz, 1H), 4.11 (t, *J* = 6.0 Hz, 2H), 2.91 (t, *J* = 6.0 Hz, 2H), 2.70–2.59 (m, 2H), 2.53–2.42 (m, 2H).

**$^{13}$C NMR** (126 MHz, $CDCl_3$) δ [ppm] = 169.8, 137.3, 131.4, 128.1, 126.9, 123.6, 120.2, 119.1, 112.0, 111.1, 99.4, 39.3, 31.9, 20.8, 19.6.

**HRMS-ESI**: calcd. for $C_{15}H_{15}N_2O$ $[M + H]^+$: 239.1179; found: 239.1179.

**FT-IR:** $\nu$ [cm$^{-1}$] = 3287, 2926, 1636, 1436, 1400, 1232, 1214, 742.

**9-Methoxy-2,6,7,12-tetrahydroindolo[2,3-*a*]quinolizin-4(3*H*)-one (12a)**.

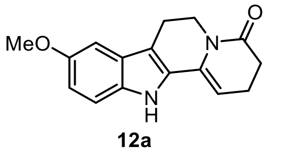


**12a**

**12a** was prepared according to **General procedure C**, starting from **11a** (100 mg, 0.220 mmol, 1.0 equiv.) and $POCl_3$ (20.1 μL, 0.220 mmol, 1.0 equiv.) in toluene (3.0 mL). The reaction mixture was subsequently treated with MeOH (3.0 mL), $K_2CO_3$ (304 mg, 2.20 mmol, 10 equiv.) and *n*$Bu_4$NBr (7.1 mg, 22.0 μmol, 0.1 equiv.). Purification by silica gel column chromatography (pentane/EtOAc = 2:1) afforded **12a** (45.0 mg, 0.168 mmol, 76%) as a yellow solid.

**$^1$H NMR** (500 MHz, $CDCl_3$) δ [ppm] = 8.15 (s, 1H), 7.22 (dd, *J* = 8.8, 0.6 Hz, 1H),

6.94 (d, $J$ = 2.5 Hz, 1H), 6.87 (dd, $J$ = 8.8, 2.5 Hz, 1H), 5.51 (t, $J$ = 4.9 Hz, 1H), 4.11 (t, $J$ = 6.0 Hz, 2H), 3.86 (s, 3H), 2.88 (t, $J$ = 6.0 Hz, 2H), 2.63 (t, $J$ = 7.8 Hz, 2H), 2.45 (td, $J$ = 7.7, 4.9 Hz, 2H).

**$^{13}$C NMR** (126 MHz, $CDCl_3$) δ [ppm] = 169.9, 154.5, 132.4, 131.4, 128.9, 127.2, 113.8, 111.9, 111.8, 100.8, 99.2, 56.0, 39.3, 31.9, 20.8, 19.6.

**HRMS-ESI**: calcd. for $C_{16}H_{16}N_2O_2Na$ [M + Na]$^+$: 291.1104; found: 291.1105.

**FT-IR:** $v$ [cm$^{-1}$] = 3308, 2927, 2834, 1665, 1639, 1487, 1396, 1214, 1170, 1025, 801, 753.

**10-methoxy-2,6,7,12-tetrahydroindolo[2,3-*a*]quinolizin-4(3*H*)-one (12b)**.

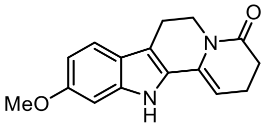

**12b**

**12b** was prepared according to **General procedure C**, starting from **11b** (80.0 mg, 0.176 mmol, 1.0 equiv.) and $POCl_3$ (16.1 µL, 0.176 mmol, 1.0 equiv.) in toluene (3.0 mL). The reaction mixture was subsequently treated with MeOH (3.0 mL), $K_2CO_3$ (243 mg, 1.76 mmol, 10 equiv.) and *n*$Bu_4NBr$ (5.7 mg, 18.0 µmol, 0.1 equiv.). Purification by silica gel column chromatography (pentane/EtOAc = 2:1) afforded **12b** (40.0 mg, 0.149 mmol, 85%) as a yellow solid.

**$^1$H NMR** (500 MHz, $CD_2Cl_2$) δ [ppm] = 8.12 (s, 1H), 7.37 (d, $J$ = 8.6 Hz, 1H), 6.85 (d, $J$ = 2.3 Hz, 1H), 6.75 (dd, $J$ = 8.6, 2.3 Hz, 1H), 5.45 (t, $J$ = 4.9 Hz, 1H), 4.05 (t, $J$ = 6.0 Hz, 2H), 3.83 (s, 3H), 2.85 (t, $J$ = 6.0 Hz, 2H), 2.57 (t, $J$ = 7.7 Hz, 2H), 2.44 (td, $J$ = 7.7, 4.9 Hz, 2H).

**$^{13}$C NMR** (126 MHz, $CD_2Cl_2$) δ [ppm] = 169.8, 158.0, 138.7, 131.7, 127.6, 121.6, 119.9, 112.2, 110.1, 98.5, 95.0, 55.9, 39.4, 32.3, 21.1, 19.9.

**HRMS-ESI**: calcd. for $C_{16}H_{17}N_2O_2$ [M + H]$^+$: 269.1284; found: 269.1284.

**FT-IR:** $v$ [cm$^{-1}$] = 3309, 2930, 2835, 1665, 1639, 1487, 1397, 1214, 1170, 1043, 1025, 754.

**10-Bromo-2,6,7,12-tetrahydroindolo[2,3-*a*]quinolizin-4(3*H*)-one (12c)**.

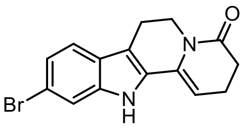

**12c**

**12c** was prepared according to **General procedure C**, starting from **11c** (37.0 mg, 74.0 µmol, 1.0 equiv.) and $POCl_3$ (6.7 µL, 74.0 µmol, 1.0 equiv.) in toluene (2.0 mL). The reaction mixture was subsequently treated with MeOH (2.0 mL), $K_2CO_3$ (99.0 mg, 740 µmol, 10 equiv.) and *n*$Bu_4NBr$ (2.4 mg, 7.0 µmol, 0.1 equiv.). Purification by silica gel column chromatography (pentane/EtOAc = 2:1) afforded **12c** (20.0 mg, 63.0 µmol, 86%) as a yellow solid.

**$^1$H NMR** (700 MHz, $d_6$-DMSO) δ [ppm] = 11.42 (s, 1H), 7.48 (dd, *J* = 1.8, 0.6 Hz, 1H), 7.44 (d, *J* = 8.4 Hz, 1H), 7.13 (dd, *J* = 8.4, 1.8 Hz, 1H), 5.87 (t, *J* = 4.9 Hz, 1H), 3.93 (t, *J* = 5.9 Hz, 2H), 2.81 (t, *J* = 6.0 Hz, 2H), 2.48 (d, *J* = 7.7 Hz, 2H), 2.42 – 2.36 (m, 2H).

**$^{13}$C NMR** (176 MHz, $d_6$-DMSO) δ [ppm] = 168.4, 138.0, 130.5, 129.2, 125.1, 121.9, 120.3, 115.1, 113.6, 110.0, 100.9, 38.4, 31.1, 20.1, 18.9.

**HRMS-ESI:** calcd. for $C_{15}H_{12}BrN_2O$ $[M – H]^-$: 315.0138; found: 315.0127.

**FT-IR:** *v* [cm$^{-1}$] = 2961, 2926, 1667, 1652, 1458, 1390, 1213, 1180, 1045, 1180, 1045, 798, 749.

**10-Fluoro-2,6,7,12-tetrahydroindolo[2,3-*a*]quinolizin-4(3*H*)-one (12d)**.

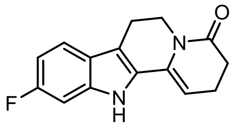

**12d**

**12d** was prepared according to **General procedure C**, starting from **11d** (100 mg, 0.226 mmol, 1.0 equiv.) and $POCl_3$ (20.6 µL, 0.226 mmol, 1.0 equiv.) in toluene (3.0 mL). The reaction mixture was subsequently treated with MeOH (3.0 mL), $K_2CO_3$ (313 mg, 2.26 mmol, 10 equiv.) and *n*$Bu_4NBr$ (7.3 mg, 23.0 µmol, 0.1 equiv.). Purification by silica gel column chromatography (pentane/EtOAc = 2:1) afforded **12d** (49.0 mg, 0.184 mmol, 81%) as a yellow solid.

**$^1$H NMR** (700 MHz, $d_6$-DMSO) δ [ppm] = 11.36 (s, 1H), 7.46 (dd, *J* = 8.6, 5.5 Hz, 1H), 7.09 (dd, *J* = 10.0, 2.3 Hz, 1H), 6.86 (ddd, *J* = 9.8, 8.6, 2.4 Hz, 1H), 5.83 (t, *J* = 4.9 Hz, 1H), 3.93 (t, *J* = 5.9 Hz, 2H), 2.80 (t, *J* = 6.0 Hz, 2H), 2.48 (t, *J* = 8.0 Hz, 2H), 2.38 (ddd, *J* = 8.0, 6.6, 3.2 Hz, 2H).

**$^{13}$C NMR** (176 MHz, $d_6$-DMSO) δ [ppm] = 168.5, 159.6 (d, *J* = 235.6 Hz), 137.2 (d, *J* = 12.7 Hz), 130.7, 129.0, 122.9, 119.7 (d, *J* = 10.7 Hz), 109.9, 107.4 (d, *J* = 24.3 Hz), 100.1, 97.2 (d, *J* = 25.8 Hz), 38.4, 31.2, 20.1, 18.9.

**$^{19}$F NMR** (471 MHz, $d_6$-DMSO) δ [ppm] = –119.64 (d, *J* = 11.5 Hz).

**HRMS-ESI**: calcd. for $C_{15}H_{13}FN_2ONa$ $[M + Na]^+$: 279.0904; found: 279.0904.

**FT-IR:** *ν* [cm$^{-1}$] = 2954, 2920, 1681, 1457, 1329, 1205, 1174, 1043, 745.

**2-Methyl-2,6,7,12-tetrahydroindolo[2,3-*a*]quinolizin-4(3*H*)-one (12e)**.

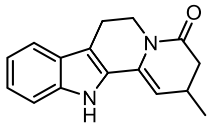

**12e**

**12e** was prepared according to **General procedure C**, starting from **11e** (150 mg, 0.342 mmol, 1.0 equiv.) and $POCl_3$ (31.3 µL, 0.342 mmol, 1.0 equiv.) in toluene (4.0 mL). The reaction mixture was subsequently treated with MeOH (4.0 mL), $K_2CO_3$ (473 mg, 3.42 mmol, 10 equiv.) and *n*$Bu_4NBr$ (11.0 mg, 34.0 µmol, 0.1 equiv.). Purification by silica gel column chromatography (pentane/EtOAc = 2:1) afforded **12e** (70.0 mg, 0.258 mmol, 75%) as a yellow solid.

<u>Scale-up reaction</u> starting from **11e** (1.00 g, 2.28 mmol, 1.0 equiv.) and $POCl_3$ (208 µL, 2.28 mmol, 1.0 equiv.) in toluene (30 mL). The reaction mixture was subsequently treated with MeOH (30 mL), $K_2CO_3$ (3.15 g, 22.8 mmol, 10 equiv.) and *n*$Bu_4NBr$ (73.6 mg, 0.228 mmol, 0.1 equiv.). Purification by silica gel column chromatography (pentane/EtOAc = 2:1) afforded **12e** (405 mg, 1.61 mmol, 70%) as a yellow solid.

**$^1$H NMR** (500 MHz, $CDCl_3$) δ [ppm] = 8.43 (s, 1H), 7.51 (d, *J* = 7.9 Hz, 1H), 7.34 (dd, *J* = 8.2, 1.1 Hz, 1H), 7.24–7.19 (m, 1H), 7.15–7.09 (m, 1H), 5.45 (t, *J* = 4.0 Hz, 1H), 4.44–4.38 (m, 1H), 3.84–3.78 (m, 1H), 2.98–2.84 (m, 2H), 2.81–2.65 (m, 2H), 2.39–2.33 (m, 1H), 1.17 (d, *J* = 6.9 Hz, 3H).

$^{13}$**C NMR** (126 MHz, $CDCl_3$) δ [ppm] = 169.8, 137.4, 130.1, 128.0, 126.8, 123.6, 120.1, 119.1, 112.1, 111.1, 106.0, 40.0, 39.3, 26.2, 20.8, 20.4.

**HRMS-ESI**: calcd. for $C_{16}H_{17}N_2O$ $[M + H]^+$: 253.1335; found: 253.1335.

**FT-IR:** $\nu$ [cm$^{-1}$] = 3286, 1638, 1399, 1328, 1303, 1213, 1180, 742.

**3-Methyl-2,6,7,12-tetrahydroindolo[2,3-*a*]quinolizin-4(3*H*)-one (12f)**.

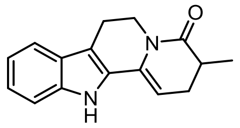

**12f**

**12f** was prepared according to **General procedure C**, starting from **11f** (132 mg, 0.301 mmol, 1.0 equiv.) and $POCl_3$ (27.5 µL, 0.301 mmol, 1.0 equiv.) in toluene (3.5 mL). The reaction mixture was subsequently treated with MeOH (3.5 mL), $K_2CO_3$ (416 mg, 3.01 mmol, 10 equiv.) and $n$$Bu_4NBr$ (9.7 mg, 30.0 µmol, 0.1 equiv.). Purification by silica gel column chromatography (pentane/EtOAc = 2:1) afforded **12f** (55.0 mg, 0.218 mmol, 72%) as a yellow solid.

$^{1}$**H NMR** (700 MHz, $CD_2Cl_2$) δ [ppm] = 8.68 (s, 1H), 7.52–7.51 (m, 1H), 7.36 (dt, $J$ = 8.1, 0.9 Hz, 1H), 7.21 (ddd, $J$ = 8.2, 7.0, 1.2 Hz, 1H), 7.10 (ddd, $J$ = 8.0, 7.0, 1.0 Hz, 1H), 5.61 (dd, $J$ = 5.9, 4.0 Hz, 1H), 4.50 (dt, $J$ = 12.7, 5.1 Hz, 1H), 3.72–3.68 (m, 1H), 2.95–2.85 (m, 2H), 2.67–2.60 (m, 1H), 2.51 (ddd, $J$ = 16.9, 6.6, 5.9 Hz, 1H), 2.24 (ddd, $J$ = 16.9, 11.4, 4.1 Hz, 1H), 1.27 (d, $J$ = 6.9 Hz, 3H).

$^{13}$**C NMR** (176 MHz, $CD_2Cl_2$) δ [ppm] = 173.1, 137.7, 131.3, 128.7, 127.1, 123.6, 120.2, 119.2, 111.8, 111.4, 99.2, 39.9, 36.1, 28.0, 21.1, 16.1.

**HRMS-ESI**: calcd. for $C_{16}H_{16}N_2ONa$ $[M + Na]^+$: 275.1155; found: 275.1161.

**FT-IR:** $\nu$ [cm$^{-1}$] = 3275, 1638, 1452, 1398, 1328, 1303, 1213, 1180, 742.

**1-Methyl-2,6,7,12-tetrahydroindolo[2,3-*a*]quinolizin-4(3*H*)-one (12g)**.

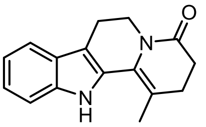

**12g**

**12g** was prepared according to **General procedure C**, starting from **11g** (108 mg,

0.247 mmol, 1.0 equiv.) and $POCl_3$ (22.5 µL, 0.247 mmol, 1.0 equiv.) in toluene (3.0 mL). The reaction mixture was subsequently treated with MeOH (3.0 mL), $K_2CO_3$ (341 mg, 2.47 mmol, 10 equiv.) and *n*$Bu_4NBr$ (7.9 mg, 25.0 µmol, 0.1 equiv.). Purification by silica gel column chromatography (pentane/EtOAc = 2:1) afforded **12g** (40.0 mg, 0.159 mmol, 64%) as a yellow solid.

**$^1$H NMR** (700 MHz, $CD_2Cl_2$) δ [ppm] = 8.42 (s, 1H), 7.54–7.52 (m, 1H), 7.41 (dt, *J* = 8.1, 0.9 Hz, 1H), 7.20 (ddd, *J* = 8.2, 7.0, 1.2 Hz, 1H), 7.12 (ddd, *J* = 8.0, 7.0, 1.0 Hz, 1H), 4.05 (t, *J* = 5.9 Hz, 2H), 2.88 (t, *J* = 5.9 Hz, 2H), 2.57–2.48 (m, 2H), 2.47–2.41 (m, 2H), 2.29 (d, *J* = 1.3 Hz, 3H).

**$^{13}$C NMR** (176 MHz, $CD_2Cl_2$) δ [ppm] = 169.6, 137.4, 129.4, 126.5, 126.3, 123.3, 120.4, 119.0, 113.1, 112.8, 111.4, 39.7, 32.0, 29.4, 21.4, 19.8.

**HRMS-ESI:** calcd. for $C_{16}H_{16}N_2ONa$ $[M + Na]^+$: 275.1155; found: 275.1169.

**FT-IR:** *ν* [cm$^{-1}$] = 3356, 1638, 1405, 1356, 1210, 1040, 744.

**2,2-Dimethyl-2,6,7,12-tetrahydroindolo[2,3-*a*]quinolizin-4(3*H*)-one (12h)**.

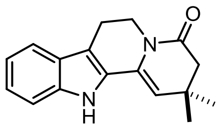


**12h**

**12h** was prepared according to **General procedure C**, starting from **11h** (100 mg, 0.221 mmol, 1.0 equiv.) and $POCl_3$ (20.2 µL, 0.221 mmol, 1.0 equiv.) in toluene (3.0 mL). The reaction mixture was subsequently treated with MeOH (3.0 mL), $K_2CO_3$ (306 mg, 2.21 mmol, 10 equiv.) and *n*$Bu_4NBr$ (7.1 mg, 22.0 µmol, 0.1 equiv.). Purification by silica gel column chromatography (pentane/EtOAc = 2:1) afforded **12h** (49.0 mg, 0.184 mmol, 83%) as a yellow solid.

**$^1$H NMR** (500 MHz, $CDCl_3$) δ [ppm] = 8.63 (s, 1H), 7.53–7.51 (m, 1H), 7.34 (dt, *J* = 8.2, 0.9 Hz, 1H), 7.23 (ddd, *J* = 8.2, 7.1, 1.2 Hz, 1H), 7.12 (ddd, *J* = 8.0, 7.1, 1.0 Hz, 1H), 5.44 (s, 1H), 4.14 (t, *J* = 6.0 Hz, 2H), 2.93 (t, *J* = 6.0 Hz, 2H), 2.52 (s, 2H), 1.18 (s, 6H).

**$^{13}$C NMR** (126 MHz, $CDCl_3$) δ [ppm] = 169.7, 137.4, 128.9, 128.0, 126.8, 123.5, 120.0, 119.0, 112.1, 111.1, 110.7, 46.5, 39.4, 30.8, 28.2, 20.7.

**HRMS-ESI:** calcd. for $C_{17}H_{18}N_2ONa$ [M + Na]$^+$: 289.1311; found: 289.1311.

**FT-IR:** *ν* [cm$^{-1}$] = 2930, 1666, 1646, 1395, 1215, 752, 740.

**(*S*)-Methyl-4-oxo-2,3,4,6,7,12-hexahydroindolo[*2,3-a*]quinolizine-6-carboxylate (12i)**.

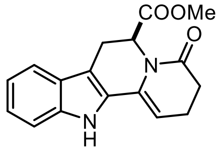


**12i**

**12i** was prepared according to **General procedure C**, starting from **11i** (69 mg, 0.143 mmol, 1.0 equiv.) and $POCl_3$ (13.1 µL, 0.134 mmol, 1.0 equiv.) in toluene (2.0 mL). The reaction mixture was subsequently treated with MeOH (2.0 mL), $K_2CO_3$ (198 mg, 1.43 mmol, 10 equiv.) and *n*$Bu_4NBr$ (4.6 mg, 14.0 µmol, 0.1 equiv.). Purification by silica gel column chromatography (pentane/EtOAc = 1:1) afforded **12i** (30.0 mg, 0.101 mmol, 71%) as a yellow solid.

**$^1$H NMR** (700 MHz, $CDCl_3$) δ [ppm] = 8.02 (s, 1H), 7.53–7.51 (m, 1H), 7.33–7.29 (m, 1H), 7.23–7.20 (m, 1H), 7.12 (ddd, *J* = 8.0, 7.1, 1.0 Hz, 1H), 5.90 (dd, *J* = 6.5, 1.5 Hz, 1H), 5.55 (dt, *J* = 8.0, 3.8 Hz, 1H), 3.60–3.58 (m, 4H), 3.14 (dd, *J* = 16.1, 6.5 Hz, 1H), 2.76–2.71 (m, 1H), 2.67–2.61 (m, 2H), 2.50–2.40 (m, 1H).

**$^{13}$C NMR** (176 MHz, $CDCl_3$) δ [ppm] = 171.3, 170.1, 137.4, 130.2, 127.8, 126.8, 123.8, 120.3, 119.2, 111.1, 109.0, 100.0, 52.8, 50.9, 31.6, 23.1, 19.6.

**HRMS-ESI**: calcd. for $C_{17}H_{16}N_2O_3Na$ [M + Na]$^+$: 319.1053; found: 319.1044.

**FT-IR:** *ν* [cm$^{-1}$] = 2928, 2850, 1736, 1698, 1366, 1331, 1215, 747.

$[\alpha]_D^{26}$ = +4.6 (*c* = 0.2, $CHCl_3$).

**8,13-Dihydroindolo[2',3':3,4]pyrido[1,2-*b*]isoquinolin-5(7*H*)-one (12j)**.

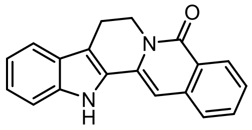


**12j** nortetoyobyrine

**12j** was prepared according to **General procedure C**, starting from **11j** (50.0 mg, 0.106 mmol, 1.0 equiv.) and $POCl_3$ (9.7 µL, 0.106 mmol, 1.0 equiv.) in toluene

(1.5 mL). The reaction mixture was subsequently treated with MeOH (1.5 mL), $K_2CO_3$ (146 mg, 1.06 mmol, 10 equiv.) and *n*$Bu_4NBr$ (3.4 mg, 11.0 µmol, 0.1 equiv.). Purification by silica gel column chromatography (pentane/EtOAc = 3:1) afforded **12j** (25.0 mg, 87.4 µmol, 82%) as a light yellow solid.

**$^1$H NMR** (500 MHz, *d*$_6$-DMSO) δ [ppm] = 11.69 (s, 1H), 8.24 (dd, *J* = 8.0, 1.3 Hz, 1H), 7.71 (td, *J* = 7.5, 1.3 Hz, 1H), 7.61 (dd, *J* = 16.4, 7.8 Hz, 2H), 7.45 (dd, *J* = 18.8, 7.9 Hz, 2H), 7.21 (ddd, *J* = 8.2, 6.9, 1.2 Hz, 1H), 7.10–7.04 (m, 2H), 4.40 (t, *J* = 6.5 Hz, 2H), 3.09 (t, *J* = 6.6 Hz, 2H).

**$^{13}$C NMR** (126 MHz, *d*$_6$-DMSO) δ [ppm] = 161.2, 138.0, 136.2, 132.6, 132.4, 128.2, 127.5, 126.1, 126.0, 125.5, 124.5, 123.5, 119.5, 119.1, 112.5, 111.6, 99.0, 40.4, 19.3.

**HRMS-ESI:** calcd. for $C_{19}H_{13}N_2O$ $[M - H]^-$: 285.1033; found: 285.1038.

**FT-IR:** *v* [cm$^{-1}$] = 2923, 2853, 1738, 1650, 1614, 1595, 1365, 1217, 742.

**2,3,4,7,8,13-Hexahydro-5*H*-azepino[1',2':1,2]pyrido[3,4-*b*]indol-5-one (12k)**.

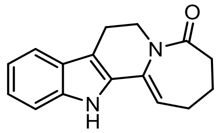


**12k**

**12k** was prepared according to **General procedure C**, starting from **11k** (200 mg, 0.457 mmol, 1.0 equiv.) and $POCl_3$ (41.7 µL, 0.457 mmol, 1.0 equiv.) in toluene (5.0 mL). The reaction mixture was subsequently treated with MeOH (5.0 mL), $K_2CO_3$ (631 mg, 4.57 mmol, 10 equiv.) and *n*$Bu_4NBr$ (14.7 mg, 46.0 µmol, 0.1 equiv.). Purification by silica gel column chromatography (pentane/EtOAc = 2:1) afforded **12k** (30.0 mg, 0.119 mmol, 26%) as a yellow solid.

**$^1$H NMR** (700 MHz, $CD_2Cl_2$) δ [ppm] = 9.10 (s, 1H), 7.48–7.46 (m, 1H), 7.35 (dt, *J* = 8.1, 0.9 Hz, 1H), 7.18 (ddd, *J* = 8.2, 7.0, 1.2 Hz, 1H), 7.07 (ddd, *J* = 8.0, 7.0, 1.0 Hz, 1H), 6.08 (t, *J* = 7.3 Hz, 1H), 4.07 (t, *J* = 5.9 Hz, 2H), 2.84 (t, *J* = 5.9 Hz, 2H), 2.55 (t, *J* = 7.1 Hz, 2H), 2.37 (q, *J* = 7.5 Hz, 2H), 2.20–2.16 (m, 2H).

**$^{13}$C NMR** (176 MHz, $CD_2Cl_2$) δ [ppm] = 175.8, 139.5, 136.6, 131.7, 129.4, 125.3, 122.0, 121.1, 114.1, 114.0, 113.4, 43.4, 37.6, 32.1, 26.1, 23.9.

**HRMS-ESI**: calcd. for $C_{16}H_{16}N_2ONa$ $[M + Na]^+$: 275.1155; found: 275.1152.

**FT-IR:** $\nu$ [cm$^{-1}$] 2928, 2855, 1713, 1659, 1625, 1442, 1329, 1230, 1170, 745.

**2,4,5,8,9,14-Hexahydroazocino[1',2':1,2]pyrido[3,4-*b*]indol-6(3*H*)-one (12l)**.

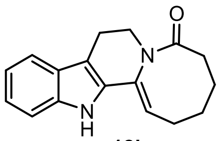

**12l**

**12l** was prepared according to **General procedure C**, starting from **11l** (200 mg, 0.429 mmol, 1.0 equiv.) and $POCl_3$ (39.2 µL, 0.429 mmol, 1.0 equiv.) in toluene (5.0 mL). The reaction mixture was subsequently treated with MeOH (5.0 mL), $K_2CO_3$ (593 mg, 4.29 mmol, 10 equiv.) and *n*$Bu_4NBr$ (13.8 mg, 43.0 µmol, 0.1 equiv.). Purification by silica gel column chromatography (pentane/EtOAc = 2:1) afforded **12l** (50.0 mg, 0.168 mmol, 39%) as a yellow solid.

**$^1$H NMR** (700 MHz, $CD_2Cl_2$) δ [ppm] = 8.34 (s, 1H), 7.47–7.46 (m, 1H), 7.34 (dt, *J* = 8.1, 0.9 Hz, 1H), 7.18 (ddd, *J* = 8.2, 7.1, 1.2 Hz, 1H), 7.08 (ddd, *J* = 8.0, 7.0, 1.0 Hz, 1H), 5.77 (t, *J* = 8.2 Hz, 1H), 5.07 (ddd, *J* = 12.5, 5.4, 1.2 Hz, 1H), 3.18–3.14 (m, 1H), 2.97 (ddd, *J* = 15.7, 12.4, 5.5 Hz, 1H), 2.76 (ddd, *J* = 15.9, 4.3, 1.2 Hz, 1H), 2.55 (td, *J* = 12.2, 1.6 Hz, 1H), 2.49–2.39 (m, 2H), 2.10 (dddd, *J* = 14.2, 11.9, 8.9, 1.3 Hz, 1H), 1.99–1.90 (m, 1H), 1.69–1.55 (m, 2H), 1.39–1.28 (m, 1H).

**$^{13}$C NMR** (176 MHz, $CD_2Cl_2$) δ [ppm] = 174.0, 137.3, 133.1, 130.4, 127.6, 123.4, 120.2, 119.1, 116.0, 112.3, 111.3, 42.8, 35.0, 26.6, 25.5, 25.1, 22.1.

**HRMS-ESI:** calcd. for $C_{17}H_{18}N_2ONa$ [M + Na]$^+$: 289.1311; found: 289.1315.

**FT-IR:** $\nu$ [cm$^{-1}$] = 3260, 2928, 2854, 1620, 1450, 1406, 1230, 1170, 743 2.

**2,3,6,7,8,13-Hexahydro-4*H*-pyrido[*1',2':1,2*]azepino[*3,4-b*]indol-4-one (12m)**.

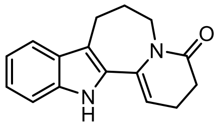

**12m**

**12m** was prepared according to **General procedure C**, starting from **11m** (50.0 mg, 0.114 mmol, 1.0 equiv.) and $POCl_3$ (10.4 µL, 0.114 mmol, 1.0 equiv.) in toluene (1.5 mL). The reaction mixture was subsequently treated with MeOH (1.5 mL), $K_2CO_3$

(158 mg, 1.14 mmol, 10 equiv.) and *n*$Bu_4NBr$ (3.7 mg, 11.0 µmol, 0.1 equiv.). Purification by silica gel column chromatography (pentane/EtOAc = 2:1) afforded **12m** (15.0 mg, 59.5 µmol, 52%) as a yellow solid.

**$^1$H NMR** (700 MHz, $CDCl_3$) δ [ppm] = 8.00 (s, 1H), 7.55–7.54 (m, 1H), 7.33 (dt, *J* = 8.1, 0.9 Hz, 1H), 7.21 (ddd, *J* = 8.2, 7.0, 1.2 Hz, 1H), 7.13 (ddd, *J* = 8.0, 7.0, 1.0 Hz, 1H), 5.54 (t, *J* = 5.1 Hz, 1H), 3.91–3.83 (m, 2H), 2.93 (t, *J* = 7.3 Hz, 2H), 2.60 (dd, *J* = 8.6, 6.7 Hz, 2H), 2.48–2.39 (m, 2H), 2.10–2.06 (m, 2H).

**$^{13}$C NMR** (176 MHz, $CDCl_3$) δ [ppm] = 170.6, 137.2, 136.0, 129.2, 128.5, 123.2, 120.1, 118.9, 113.3, 110.9, 104.4, 42.2, 31.9, 26.2, 20.1, 19.8.

**HRMS-ESI:** calcd. for $C_{16}H_{16}N_2ONa$ $[M + Na]^+$: 275.1155; found: 275.1156.

**FT-IR:** *ν* [$cm^{-1}$] 2925, 2858, 1653, 1398, 1328, 1260, 1239, 1171, 745.

**2,6,7,8,9,14-Hexahydropyrido[1',2':1,2]azocino[3,4-*b*]indol-4(3*H*)-one (12n)**.

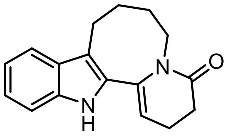

**12n**

**12n** was prepared according to **General procedure C**, starting from **11n** (39.0 mg, 86.0 µmol, 1.0 equiv.) and $POCl_3$ (7.9 µL, 86.0 µmol, 1.0 equiv.) in toluene (1.5 mL). The reaction mixture was subsequently treated with MeOH (1.5 mL), $K_2CO_3$ (119 mg, 863 µmol, 10 equiv.) and *n*$Bu_4NBr$ (2.8 mg, 9.0 µmol, 0.1 equiv.). Purification by silica gel column chromatography (pentane/EtOAc = 2:1) afforded **12n** (10.0 mg, 37.6 µmol, 43%) as a yellow solid.

**$^1$H NMR** (700 MHz, $CDCl_3$) δ [ppm] = 7.83 (s, 1H), 7.55 (dt, *J* = 7.9, 0.9 Hz, 1H), 7.33 (dt, *J* = 8.1, 0.9 Hz, 1H), 7.22 (ddd, *J* = 8.1, 7.0, 1.1 Hz, 1H), 7.14 (ddd, *J* = 8.0, 7.0, 1.0 Hz, 1H), 5.44 (t, *J* = 4.9 Hz, 1H), 2.92–2.88 (m, 2H), 2.63 (dd, *J* = 8.4, 7.3 Hz, 2H), 2.46–2.42 (m, 2H), 1.93–1.80 (m, 6H).

**$^{13}$C NMR** (176 MHz, $CDCl_3$) δ [ppm] = 170.2, 136.9, 135.2, 128.6, 128.0, 122.9, 119.8, 118.8, 114.9, 110.7, 106.4, 43.3, 31.9, 28.9, 24.1, 23.6, 20.0.

**HRMS-ESI:** calcd. for $C_{17}H_{18}N_2ONa$ $[M + Na]^+$: 289.1311; found: 289.1388.

**FT-IR:** *ν* [$cm^{-1}$] = 2954, 2922, 2853, 1737, 1661, 1458, 1378, 1217, 1033, 743.

**2,2-Dimethyl-2,3,6,7-tetrahydro-4*H*-pyrido[2,1-*a*]isoquinolin-4-one (12o)**.

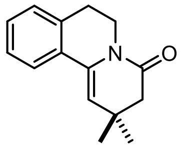

**12o**

**12o** was prepared according to **General procedure C**, starting from **11o** (138 mg, 0.334 mmol, 1.0 equiv.) and $POCl_3$ (76.2 µL, 0.835 mmol, 2.5 equiv.) in toluene (4.0 mL). The reaction mixture was subsequently treated with MeOH (4.0 mL), $K_2CO_3$ (462 mg, 3.34 mmol, 10 equiv.) and *n*$Bu_4NBr$ (10.8 mg, 33.0 µmol, 0.1 equiv.). Purification by neutral aluminium oxide column chromatography (pentane/DCM = 1:1) afforded **12o** (25.0 mg, 0.110 mmol, 33%) as a white solid.

**$^1$H NMR** (700 MHz, $C_6D_6$) δ [ppm] = 7.36–7.33 (m, 1H), 7.01–6.95 (m, 2H), 6.76–6.72 (m, 1H), 5.34 (s, 1H), 3.79 (dd, $J$ = 6.4, 5.4 Hz, 2H), 2.32 (d, $J$ = 0.8 Hz, 2H), 2.31 (td, $J$ = 5.9, 0.9 Hz, 2H), 0.92 (s, 6H).

**$^{13}$C NMR** (176 MHz, $C_6D_6$) δ [ppm] = 168.3, 135.1, 134.1, 130.9, 128.4, 128.4, 126.8, 124.3, 112.7, 46.0, 38.3, 30.5, 29.4, 27.9.

**HRMS-ESI:** calcd. for $C_{15}H_{17}NONa$ $[M + Na]^+$: 250.1202; found: 250.1208.

**FT-IR:** $\nu$ [cm$^{-1}$] = 2948, 2925, 2858, 1974, 1733, 1626, 1492, 1465, 1429, 1320, 1208, 1126, 743.

**10-Methoxy-2,2-dimethyl-2,3,6,7-tetrahydro-4*H*-pyrido[2,1-*a*]isoquinolin-4-one (12p)**.

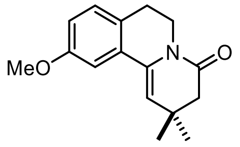

**12p**

**12p** was prepared according to **General procedure C**, starting from **11p** (80.0 mg, 0.181 mmol, 1.0 equiv.) and $POCl_3$ (16.5 µL, 0.181 mmol, 1.0 equiv.) in toluene (3.0 mL). The reaction mixture was subsequently treated with MeOH (3.0 mL), $K_2CO_3$ (250 mg, 1.81 mmol, 10 equiv.) and *n*$Bu_4NBr$ (5.8 mg, 18 µmol, 0.1 equiv.). Purification by neutral aluminium oxide column chromatography (pentane/DCM = 1:1)

afforded **12p** (20.0 mg, 77.8 μmol, 43%) as a white solid.

**$^1$H NMR** (700 MHz, $C_6D_6$) δ [ppm] = 6.70 (dd, *J* = 8.3, 0.42 Hz,1H), 6.66 (dd, *J* = 8.3, 2.5 Hz, 1H), 5.40 (d, *J* = 0.9 Hz, 1H), 3.88–3.82 (m, 2H), 3.32 (s, 3H), 2.35–2.29 (m, 4H), 0.91 (s, 6H). (Due to the solvent peak overlapping, one resonance is missing. But the ABX system could be proved by the coupling constant of the rest two Hs.)

**$^{13}$C NMR** (176 MHz, $C_6D_6$) δ [ppm] = 168.4, 159.1, 134.3, 131.9, 129.4, 127.6, 114.2, 112.9, 109.6, 54.9, 46.0, 38.6, 30.5, 28.7, 27.9.

**HRMS-ESI:** calcd. for $C_{16}H_{19}NO_2Na$ $[M + Na]^+$: 280.1308; found: 280.1301.

**FT-IR:** *ν* [cm$^{-1}$] = 2926, 2854, 1746, 1655, 1605, 1518, 1466, 1360, 1271, 1232, 1193, 1140, 1109, 1029, 910.

**9,10-Dimethoxy-2,2-dimethyl-2,3,6,7-tetrahydro-4*H*-pyrido[2,1-*a*]isoquinolin-4-one (12q)**.

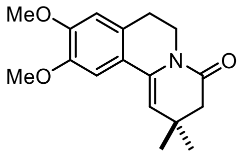


**12q**

**12q** was prepared according to **General procedure C**, starting from **11q** (150 mg, 0.317 mmol, 1.0 equiv.) and $POCl_3$ (28.9 μL, 0.317 mmol, 1.0 equiv.) in toluene (5.0 mL). The reaction mixture was subsequently treated with MeOH (5.0 mL), $K_2CO_3$ (438 mg, 3.17 mmol, 10 equiv.) and *n*$Bu_4NBr$ (10.2 mg, 32.0 μmol, 0.1 equiv.). Purification by silica gel column chromatography (pentane/EtOAc = 3:1) afforded **12q** (80.5 mg, 0.280 mmol, 88%) as a yellow solid.

**$^1$H NMR** (500 MHz, $CDCl_3$) δ [ppm] = 7.02 (s, 1H), 6.61 (s, 1H), 5.47 (s, 1H), 3.91–3.89 (m, 5H), 3.88 (s, 3H), 2.77 (t, *J* = 5.9 Hz, 2H), 2.42 (s, 2H), 1.14 (s, 6H).

**$^{13}$C NMR** (126 MHz, $CDCl_3$) δ [ppm] = 169.7, 149.3, 148.2, 133.2, 127.6, 122.3, 112.0, 110.8, 106.8, 56.2, 56.1, 46.0, 38.5, 30.5, 28.9, 28.2.

**HRMS-ESI:** calcd. for $C_{17}H_{21}NO_3Na$ $[M + Na]^+$: 310.1413; found: 310.1428.

**FT-IR:** *ν* [cm$^{-1}$] = 2964, 2934, 1775, 1645, 1516, 1388, 1358, 1287, 1263, 1234, 1198, 1109, 937.

**9,10-Dimethoxy-2,3,6,7-tetrahydro-4*H*-pyrido[2,1-*a*]isoquinolin-4-one (12r)**.

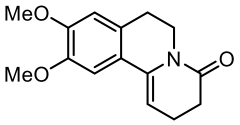


**12r**

**12r** was prepared according to **General procedure C**, starting from **11r** (100 mg, 0.225 mmol, 1.0 equiv.) and $POCl_3$ (20.5 µL, 0.225 mmol, 1.0 equiv.) in toluene (3.0 mL). The reaction mixture was subsequently treated with MeOH (3.0 mL), $K_2CO_3$ (311 mg, 2.25 mmol, 10 equiv.) and *n*$Bu_4NBr$ (7.2 mg, 22 µmol, 0.1 equiv.). Purification by neutral aluminium oxide column chromatography (pentane/DCM = 1:1) afforded **12r** (44.0 mg, 0.170 mmol, 76%) as a yellow solid.

**$^1$H NMR** (500 MHz, $C_6D_6$) δ [ppm] = 6.92 (s, 1H), 6.20 (s, 1H), 5.31 (td, *J* = 5.0, 1.9 Hz, 1H), 3.93 (td, *J* = 5.9, 1.8 Hz, 2H), 3.46 (s, 3H), 3.36 (s, 3H), 2.41 (td, *J* = 7.9, 1.8 Hz, 2H), 2.38–2.31 (m, 2H), 2.00–1.95 (m, 2H).

**$^{13}$C NMR** (126 MHz, $C_6D_6$) δ [ppm] = 169.0, 150.4, 149.2, 136.4, 127.8, 123.0, 111.6, 108.4, 99.7, 55.9, 55.5, 38.6, 31.6, 29.0, 19.9.

**HRMS-ESI:** calcd. for $C_{15}H_{17}NO_3Na$ $[M + Na]^+$: 282.1100; found: 282.1106.

**FT-IR:** $\nu$ [cm$^{-1}$] = 2942, 1682, 1602, 1513, 1465, 1377, 1341, 1272, 1202, 1179, 1162, 752.

**2,3-Dimethoxy-5,6-dihydro-8*H*-isoquinolino[3,2-*a*]isoquinolin-8-one (12s)**.

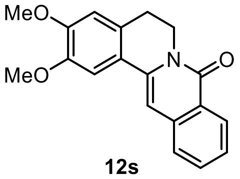


**12s**

**12s** was prepared according to **General procedure C**, starting from **11s** (193 mg, 0.391 mmol, 1.0 equiv.) and $POCl_3$ (35.7 µL, 0.391 mmol, 1.0 equiv.) in toluene (5.0 mL). The reaction mixture was subsequently treated with MeOH (5.0 mL), $K_2CO_3$ (541 mg, 3.92 mmol, 10 equiv.) and *n*$Bu_4NBr$ (12.6 mg, 39.0 µmol, 0.1 equiv.). Purification by silica gel column chromatography (pentane/EtOAc = 3:1) afforded **12s** (100 mg, 0.326 mmol, 83%) as a yellow solid.

**$^1$H NMR** (600 MHz, $CDCl_3$) δ [ppm] = 8.41 (dt, *J* = 8.1, 1.7 Hz, 1H), 7.62–7.59 (m,

1H), 7.58–7.53 (m, 1H), 7.44–7.40 (m, 1H), 7.26 (d, *J* = 2.1 Hz, 1H), 6.86 (t, *J* = 2.1 Hz, 1H), 6.73 (d, *J* = 2.4 Hz, 1H), 4.39–4.32 (m, 2H), 3.98 (s, 3H), 3.93 (s, 3H), 2.93 (m, 2H).

**$^{13}$C NMR** (151 MHz, $CDCl_3$) δ [ppm] = 162.3, 150.5, 148.6, 137.5, 136.8, 132.4, 128.8, 128.1, 126.3, 126.0, 124.7, 122.4, 110.6, 108.0, 101.5, 56.4, 56.1, 39.8, 28.2.

**HRMS-ESI:** calcd. for $C_{19}H_{17}NO_3Na$ $[M + Na]^+$: 330.1100 found: 330.1115.

**FT-IR:** *ν* [cm$^{-1}$] = 3001, 2937, 2839, 1644, 1606, 1512, 1466, 1362, 1269, 1234, 1104, 1018, 754.

**5,6-Dihydro-8*H*-isoquinolino[3,2-*a*]isoquinolin-8-one (12t)**.

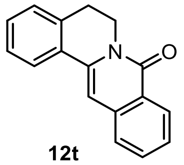

**12t**

**12t** was prepared according to **General procedure C**, starting from **11t** (217 mg, 0.501 mmol, 1.0 equiv.) and $POCl_3$ (45.7 µL, 0.501 mmol, 1.0 equiv.) in toluene (6.0 mL). The reaction mixture was subsequently treated with MeOH (6.0 mL), $K_2CO_3$ (693 mg, 5.01 mmol, 10 equiv.) and *n*$Bu_4NBr$ (16.2 mg, 50.0 µmol, 0.1 equiv.). Purification by silica gel column chromatography (pentane/EtOAc = 5:1) afforded **12t** (60 mg, 0.243 mmol, 48%) as a yellow solid.

**$^{1}$H NMR** (600 MHz, $CD_2Cl_2$) δ [ppm] = 8.36 (dd, *J* = 8.1, 1.3 Hz, 1H), 7.90–7.84 (m, 1H), 7.65 (ddd, *J* = 8.2, 6.9, 1.4 Hz, 1H), 7.63–7.60 (m, 1H), 7.46 (ddd, *J* = 8.1, 6.9, 1.4 Hz, 1H), 7.41–7.34 (m, 2H), 7.33–7.27 (m, 1H), 7.05 (s, 1H), 4.37–4.30 (m, 2H), 3.06–2.98 (m, 2H).

**$^{13}$C NMR** (151 MHz, $CD_2Cl_2$) δ [ppm] = 162.2, 138.0, 137.0, 136.1, 132.6, 130.7, 129.7, 128.4, 128.1, 127.8, 126.8, 126.7, 125.4, 125.3, 102.8, 40.0, 28.9.

**HRMS-ESI:** calcd. for $C_{17}H_{13}NONa$ $[M + Na]^+$: 270.0889; found: 270.0903.

**FT-IR:** *ν* [cm$^{-1}$] = 2945, 2896, 1647, 1619, 1596, 1491, 1341, 1315, 1166, 766.

**4,5,8,9-Tetrahydro-7*H*-furo[2,3-*a*]quinolizin-7-one (12u)**.

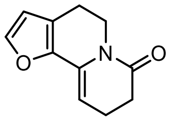

**12u**

**12u** was prepared according to **General procedure C**, starting from **11u** (43.0 mg, 0.115 mmol, 1.0 equiv.) and $POCl_3$ (10.5 µL, 0.115 mmol, 1.0 equiv.) in toluene (1.5 mL). The reaction mixture was subsequently treated with MeOH (1.5 mL), $K_2CO_3$ (158 mg, 1.15 mmol, 10 equiv.) and *n*$Bu_4$NBr (3.7 mg, 11 µmol, 0.1 equiv.). Purification by neutral aluminium oxide column chromatography (pentane/DCM = 1:1) afforded **12u** (18.0 mg, 95.2 µmol, 83%) as a yellow solid.

**$^1$H NMR** (700 MHz, $C_6D_6$) δ [ppm] = 6.90 (t, *J* = 1.7 Hz, 1H), 5.80 (t, *J* = 1.7 Hz, 1H), 5.45 (t, *J* = 5.0 Hz, 1H), 3.82 (t, *J* = 6.0 Hz, 2H), 2.30 (t, *J* = 7.7 Hz, 2H), 2.06 (t, *J* = 6.0 Hz, 2H), 1.85 (td, *J* = 7.7, 5.0 Hz, 2H).

**$^{13}$C NMR** (176 MHz, $C_6D_6$) δ [ppm] = 168.4, 145.3, 142.6, 130.4, 118.2, 110.8, 98.1, 38.7, 32.0, 21.6, 19.5.

**HRMS-ESI:** calcd. for $C_{11}H_{11}NO_2Na$ $[M + Na]^+$: 212.0682; found: 212.0675.

**FT-IR:** *ν* [cm$^{-1}$] = 2929, 2855, 1725, 1553, 1457, 1380, 1335, 1181, 1019, 742.

**4,5,8,9-Tetrahydro-7*H*-furo[3,2-*a*]quinolizin-7-one (12v)**.

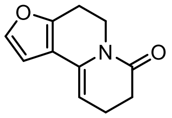

**12v**

**12v** was prepared according to **General procedure C**, starting from **11v** (40.0 mg, 0.107 mmol, 1.0 equiv.) and $POCl_3$ (9.7 µL, 0.107 mmol, 1.0 equiv.) in toluene (1.5 mL). The reaction mixture was subsequently treated with MeOH (1.5 mL), $K_2CO_3$ (147 mg, 1.07 mmol, 10 equiv.) and *n*$Bu_4$NBr (3.4 mg, 11 µmol, 0.1 equiv.). Purification by neutral aluminium oxide column chromatography (pentane/DCM = 1:1) afforded **12v** (10.0 mg, 54.0 µmol, 51%) as a yellow solid.

**$^1$H NMR** (700 MHz, $C_6D_6$) δ [ppm] = 6.90 (dd, *J* = 2.0, 0.7 Hz, 1H), 6.11 (d, *J* = 2.0 Hz, 1H), 4.93 (t, *J* = 4.9 Hz, 1H), 3.81 (t, *J* = 6.1 Hz, 2H), 2.33 (t, *J* = 7.7 Hz, 2H), 2.22–2.18 (m, 2H), 1.87–1.83 (m, 2H).

**$^{13}$C NMR** (176 MHz, $C_6D_6$) δ [ppm] = 168.7, 150.0, 142.3, 132.5, 115.2, 106.3, 100.0, 38.3, 32.2, 23.0, 19.8.

(Note: as the product owns a vinylogous fragment, which is highly reactive, the NMR shows some impurity, thought we used quite mild isolation method.)

**HRMS-ESI:** calcd. for $C_{11}H_{11}NO_2Na$ $[M + Na]^+$: 212.0682; found: 212.0688.

**FT-IR:** *ν* [cm$^{-1}$] = 2925, 1697, 1368, 1301, 1221, 1168, 1122, 1079, 1041, 746.

**4,5,8,9-Tetrahydro-7*H*-thieno[2,3-*a*]quinolizin-7-one (12w)**.

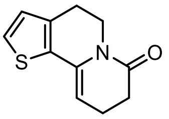

**12w**

**12w** was prepared according to **General procedure C**, starting from **11w** (48.0 mg, 0.123 mmol, 1.0 equiv.) and $POCl_3$ (11.2 μL, 0.123 mmol, 1.0 equiv.) in toluene (1.5 mL). The reaction mixture was subsequently treated with MeOH (1.5 mL), $K_2CO_3$ (170 mg, 1.23 mmol, 10 equiv.) and *n*$Bu_4NBr$ (4.0 mg, 12 μmol, 0.1 equiv.). Purification by neutral aluminium oxide column chromatography (pentane/DCM = 1:1) afforded **12w** (23.0 mg, 0.112 mmol, 91%) as a yellow solid.

**$^1$H NMR** (500 MHz, $C_6D_6$) δ [ppm] = 6.61 (dd, *J* = 5.1, 2.0 Hz, 1H), 6.31 (dd, *J* = 5.1, 2.0 Hz, 1H), 5.20 (td, *J* = 5.0, 2.0 Hz, 1H), 3.83 (td, *J* = 6.0, 2.0 Hz, 2H), 2.29 (td, *J* = 7.8, 1.9 Hz, 2H), 2.17 (td, *J* = 6.0, 2.0 Hz, 2H), 1.79 (tdd, *J* = 7.5, 4.9, 1.9 Hz, 2H).

**$^{13}$C NMR** (126 MHz, $C_6D_6$) δ [ppm] = 168.9, 136.6, 133.9, 132.4, 128.0, 124.5, 101.1, 39.0, 32.3, 25.6, 20.2.

**HRMS-ESI:** calcd. for $C_{11}H_{11}NOSNa$ $[M + Na]^+$: 228.0453; found: 228.0453.

**FT-IR:** *ν* [cm$^{-1}$] = 2930, 1681, 1433, 1367, 1331, 1304, 1225, 1171, 1136, 1035, 875, 764.

**2,3,6,7-tetrahydro-4*H*-benzo[4,5]thieno[2,3-*a*]quinolizin-4-one (12x)**.

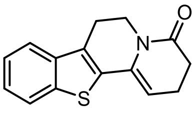

**12x**

**12x** was prepared according to **General procedure C**, starting from **11x** (30.0 mg, 68.0 µmol, 1.0 equiv.) and $POCl_3$ (6.2 µL, 68 µmol, 1.0 equiv.) in toluene (1.5 mL). The reaction mixture was subsequently treated with MeOH (1.5 mL), $K_2CO_3$ (94.0 mg, 0.680 mmol, 10 equiv.) and *n*$Bu_4NBr$ (2.2 mg, 7.0 µmol, 0.1 equiv.). Purification by neutral aluminium oxide column chromatography (pentane/DCM = 1:1) afforded **12w** (15.0 mg, 58.8 µmol, 86%) as a yellow solid.

**$^1$H NMR** (700 MHz, $CD_2Cl_2$) δ [ppm] = 7.79 (ddd, *J* = 7.8, 1.3, 0.7 Hz, 1H), 7.63 (ddd, *J* = 7.8, 1.4, 0.7 Hz, 1H), 7.40–7.36 (m, 1H), 7.36–7.33 (m, 1H), 5.66 (t, *J* = 5.0 Hz, 1H), 4.08 (t, *J* = 6.0 Hz, 2H), 2.99–2.90 (m, 2H), 2.58 (t, *J* = 7.8 Hz, 2H), 2.51–2.40 (m, 2H).

**$^{13}$C NMR** (176 MHz, $CD_2Cl_2$) δ [ppm] = 169.3, 139.1, 133.5, 131.7, 130.8, 125.8, 125.0, 122.8, 121.9, 104.6, 38.4, 31.9, 23.6, 20.2. (One sp$^2$ carbon is missing, probably due to the resonance overlap.)

**HRMS-ESI:** calcd. for $C_{15}H_{13}NOSNa$ $[M + Na]^+$: 278.0610; found: 278.0624.

**FT-IR:** *v* [cm-1] = 2929, 2876, 1652, 1458, 1398, 1382, 1329, 1215, 1045, 745.

**10-Methoxy-2-methyl-2,6,7,12-tetrahydroindolo[2,3-*a*]quinolizin-4(3*H*)-one (13)**.

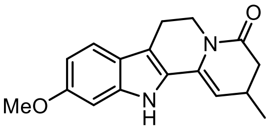


**13**

**13** was prepared according to **General procedure C**, starting from **11y** (100 mg, 0.214 mmol, 1.0 equiv.) and $POCl_3$ (19.5 µL, 0.214 mmol, 1.0 equiv.) in toluene (3.0 mL). The reaction mixture was subsequently treated with MeOH (3.0 mL), $K_2CO_3$ (295 mg, 2.14 mmol, 10 equiv.) and *n*$Bu_4NBr$ (6.9 mg, 21 µmol, 0.1 equiv.). Purification by silica gel column chromatography (pentane/EtOAc = 2:1) afforded **13** (47.0 mg, 0.167 mmol, 78%) as a yellow solid.

**$^1$H NMR** (700 MHz, $CD_2Cl_2$) δ [ppm] = 8.37 (s, 1H), 7.37 (dt, *J* = 8.6, 0.6 Hz, 1H), 6.85 (dd, *J* = 2.3, 0.5 Hz, 1H), 6.75 (dd, *J* = 8.6, 2.3 Hz, 1H), 5.37 (d, *J* = 4.0 Hz, 1H), 4.34 (dt, *J* = 12.7, 5.3 Hz, 1H), 3.83 (s, 3H), 3.79–3.75 (m, 1H), 2.88–2.83 (m, 2H), 2.79–2.71 (m, 1H), 2.65 (ddd, *J* = 15.4, 5.9, 1.0 Hz, 1H), 2.32 (ddd, *J* = 15.4, 10.3, 0.7

Hz, 1H), 1.15 (d, $J$ = 7.0 Hz, 3H).

**$^{13}$C NMR** (176 MHz, $CD_2Cl_2$) δ [ppm] = 169.7, 158.0, 138.7, 130.4, 127.4, 121.5, 119.9, 112.4, 110.0, 105.2, 95.0, 55.9, 40.4, 39.4, 26.5, 21.0, 20.5.

**HRMS-EI:** calcd. for $C_{17}H_{18}N_2O_2$ $[M]^+$: 282.1362; found: 282.1363.

**FT-IR:** $\nu$ [cm$^{-1}$] 2952, 2931, 1641, 1625, 1454, 1398, 1262, 1159, 1038, 770.

2.2.3. Detail and consideration of the annulation of the additional substrates

The annulation of the additional substrates can be divided into two parts. One is no observation of product by the NMR (group I), the other is NMR showed the product, but liable to decompose (group II).

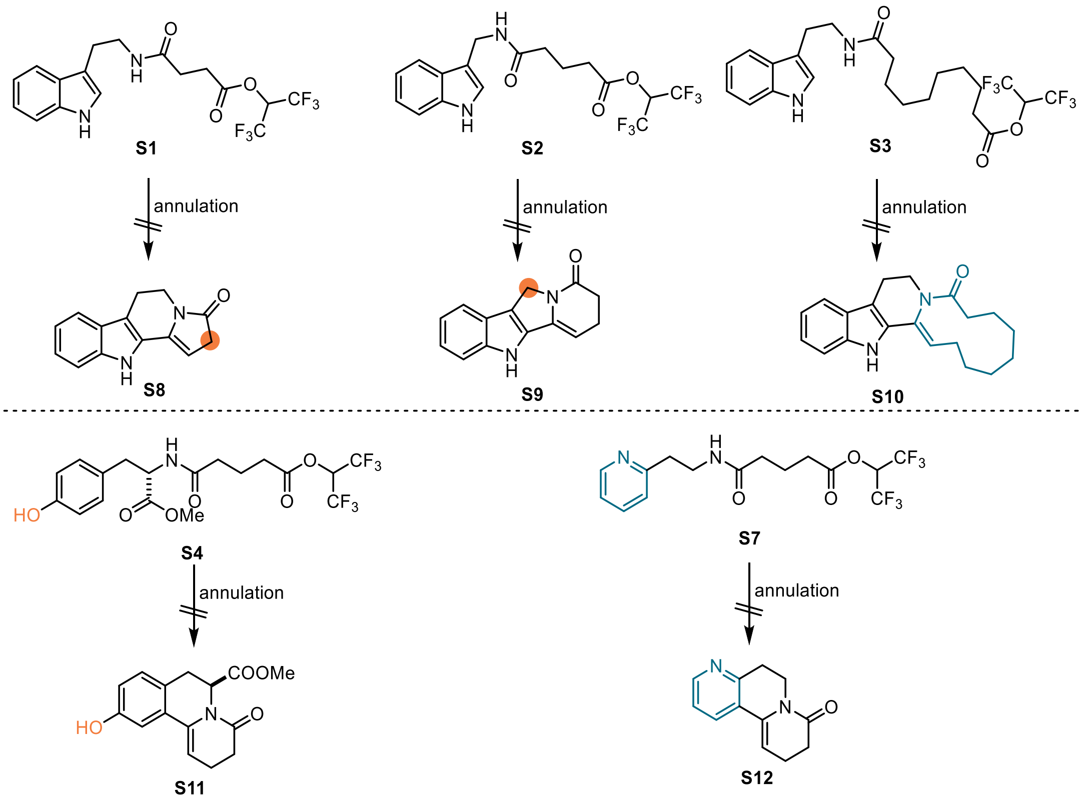


**Scheme S-1**: Group I examples

There was no proof that the annulation products of **S1–3** were formed. We proposed the **S8** and **S9** have a reactive methylene group, which might cause **S8** and **S9** unstable under the reaction condition. **S12** contains an 11-membered ring with a C–C double

bond. The unsuccessful result of **S3** could be attributed to the difficulty of the macrolactamization or the geometry of the C–C double bond. Failure of the annulation of **S4** could be caused by the reaction between the free hydroxyl group and the phosphorus (V) reagent. Unsuccessful annulation of **S7** indicates that the reaction is relied on electrophilicity of the heterocycles. (**Scheme S–1**)

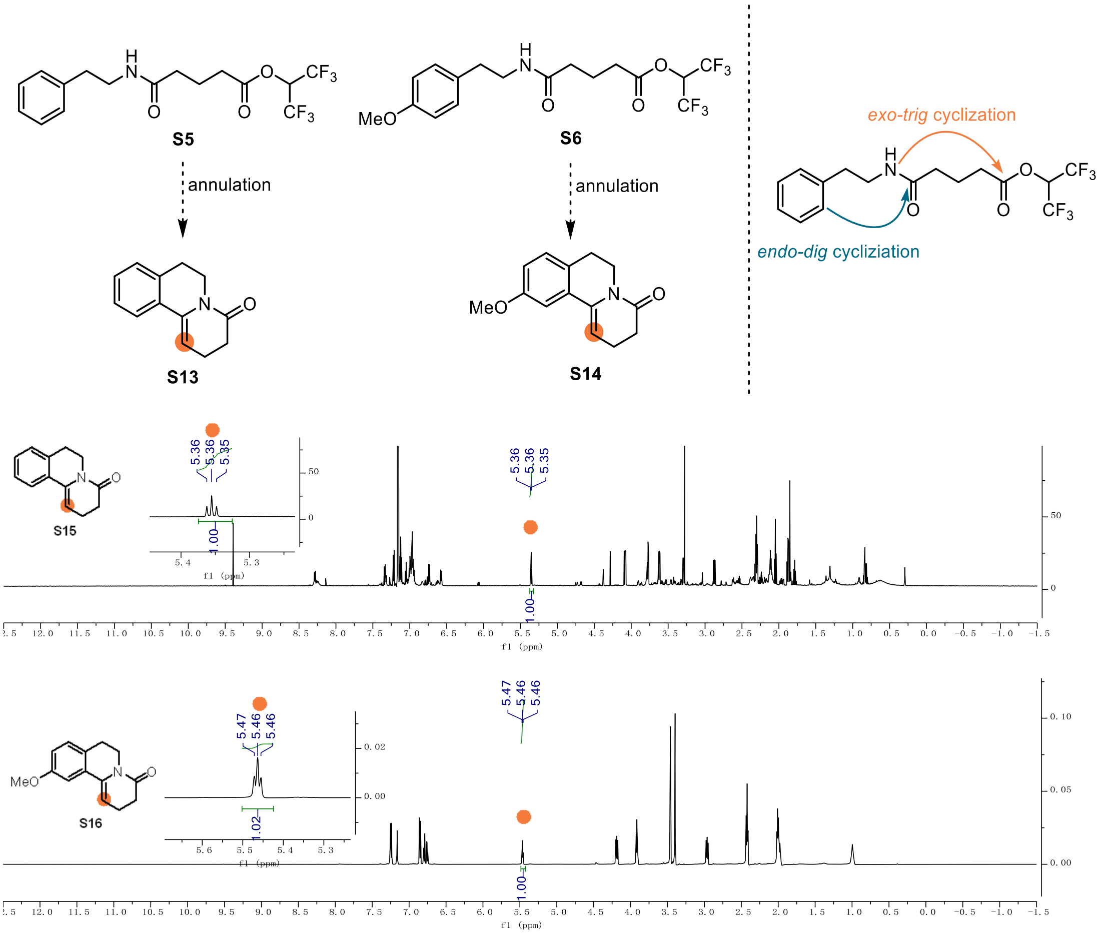


**Scheme S-2:** Group II examples

The reaction of **S5** and **S6** showed the identical peak of this type of compound on the $^1$H NMR, but there are some impurities which might be the side products formed during the reaction period or in the isolation. We hypothesized that the electrophilicity of the benzene ring influenced the reaction rate of the *endo-dig* cyclization. If the rate of the *endo-dig* cyclization is slow, some competition reactions could happen, leading to side products. In the *exo-trig* cyclization stage, the Thorpe–Ingold effect could increase the rate and drive the whole reaction equilibrium to form the desired product. (**Scheme S–2**)

In general, these compounds containing the [2,3-*a*]quinolizinone motif are sensitive to acid, in some case even the $CDCl_3$ can lead to partly decomposition. The stability is dependent on the fused ring system. According to our experience, the indole fused compounds are stable. But the larger ring system is relatively unstable. The other heterocycles mentioned above, without another aromatic ring like **12s** and **12t**, are relatively unstable.

2.3. Oxidative pathway

2.3.1. Natural products and analogues synthesis

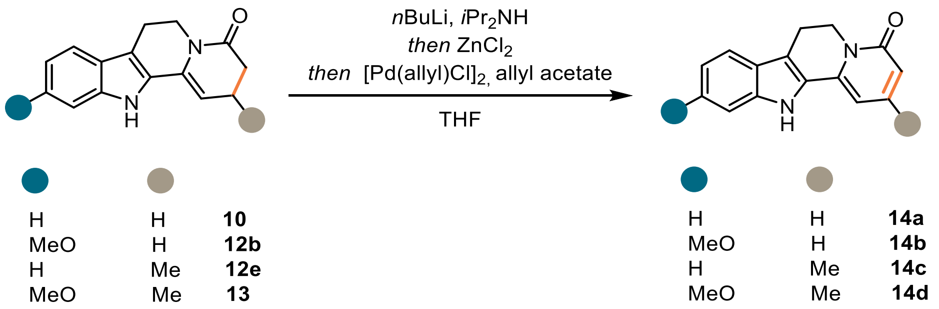


**General procedure D**[1]

To a 0 ºC solution of diisopropylamine (10.0 equiv.) in THF was added *n*BuLi (2.5 M in hexane, 10.0 equiv.). The reaction mixture was stirred for 1 h. A solution of enamide (1.0 equiv.) in THF was added dropwise into the resulting mixture. The reaction mixture was stirred for 1 h. $ZnCl_2$ (1.9 M in 2-Me-THF, 3.5 equiv.) was added and stirred for 1 h. A stock solution of $[Pd(allyl)Cl]_2$ (2.5 mol%) and allyl acetate (1.2 equiv.) in THF was next added at the same temperature. The reaction mixture was moved into a preheated 60 ºC oil bath and stirred for 3 d. The resulting mixture was cooled to room temperature and quenched by $NH_4Cl$ (sat. aq.), diluted with DCM, and the organic phase was separated. The aqueous phase was extracted with DCM and the combined organic layers were washed with brine, dried over $MgSO_4$, filtered, and concentrated under reduced pressure by rotary evaporation and purified by silica gel column chromatography.

(Note: Preparation of $[Pd(allyl)Cl]_2$, allyl acetate stock solution: [Pd(allyl)Cl]2 (27 mg, 0.075 mmol) was weighed into a flame dried vial, the vial was evacuated and backfilled with argon (this process was repeated 3 times). Allyl acetate (0.39 mL, 0.36 g, 3.6 mmol) and THF (2.6 mL) were added sequentially and the solution was stirred for 0.5 h.)

[1] Y. Chen, A. Turlik, T. R. Newhouse, *J. Am. Chem. Soc.* **2016**, *138*, 1166–1169

**7,12-Dihydroindolo[2,3-*a*]quinolizin-4(6*H*)-one (14a)**.

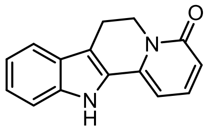


demethoxyharmalanine (**14a**)

**14a** was prepared according to **General procedure D**, starting from diisopropylamine (0.29 mL, 2.1 mmol, 10.0 equiv.) in THF (5.0 mL) was added *n*BuLi (2.5 M in hexane, 0.84 mL, 2.1 mmol, 10.0 equiv.). A solution of **10** (50 mg, 0.21 mmol, 1.0 equiv.) in THF (5.0 mL) was added dropwise into the resulting mixture. Then the reaction mixture was treated with $ZnCl_2$ (1.9 M in 2-Me-THF, 0.39 mL, 0.73 mmol, 3.5 equiv.). Afterwards a stock solution of $[Pd(allyl)Cl]_2$ (1.9 mg, 5.1 µmol, 2.5 mol%) and allyl acetate (27 µL, 25 µmol, 1.2 equiv.) in THF (1.0 mL) was next added. Purification by silica gel column chromatography (pentane/EtOAc = 1:2) afforded **14a** (38 mg, 0.16 mmol, 76%) as a yellow solid.

**$^1$H NMR** (700 MHz, $CD_3OD$) δ [ppm] = 8.10 (dt, *J* = 8.0, 1.0 Hz, 1H), 8.06 (dd, *J* = 9.0, 7.1 Hz, 1H), 7.93 (dt, *J* = 8.3, 0.9 Hz, 1H), 7.76 (ddd, *J* = 8.2, 7.0, 1.1 Hz, 1H), 7.61 (ddd, *J* = 8.0, 7.0, 0.9 Hz, 1H), 7.23 (dd, *J* = 7.1, 1.2 Hz, 1H), 6.99 (dd, *J* = 8.9, 1.2 Hz, 1H), 4.95 (t, *J* = 7.0 Hz, 2H), 3.64 (dd, *J* = 7.5, 6.5 Hz, 2H).

**$^{13}$C NMR** (176 MHz, $CD_3OD$) δ [ppm] = 165.0, 141.2, 140.3, 140.0, 128.6, 126.9, 125.5, 121.1, 120.4, 117.8, 115.3, 112.8, 102.4, 42.1, 20.3.

**HRMS-ESI:** calcd. for $C_{15}H_{13}N_2O$ $[M + H]^+$: 237.1023; found: 237.1021.

**FT-IR:** $\nu$ [cm$^{-1}$] = 2927, 2185, 1652, 1568, 1539, 1500, 1452, 1324, 1143, 777, 740.

**10-Methoxy-7,12-dihydroindolo[2,3-*a*]quinolizin-4(6*H*)-one (14b)**.

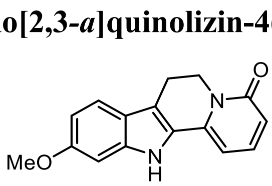


harmalanine (**14b**)

**14b** was prepared according to **General procedure D**, starting from diisopropylamine (0.13 mL, 0.93 mmol, 10.0 equiv.) in THF (2.5 mL) was added *n*BuLi (2.5 M in hexane, 0.37 mL, 0.93 mmol, 10.0 equiv.). A solution of **12b** (25 mg, 93 µmol, 1.0 equiv.) in THF (2.5 mL) was added dropwise into the resulting mixture. Then the reaction mixture

was treated with $ZnCl_2$ (1.9 M in 2-Me-THF, 0.17 mL, 0.33 mmol, 3.5 equiv.). After that, the stock solution of $[Pd(allyl)Cl]_2$ (0.85 mg, 2.3 μmol, 2.5 mol%) and allyl acetate (11.2 μL, 0.11 mmol, 1.2 equiv.) in THF (0.5 mL) was next added. Purification by silica gel column chromatography (pentane/EtOAc = 1:2) afforded **14a** (15 mg, 56 μmol, 60%) as a yellow solid.

**$^1$H NMR** (500 MHz, $CDCl_3$) δ [ppm] = 8.27 (s, 1H), 7.46 (d, *J* = 8.7 Hz, 1H), 7.33 (dd, *J* = 9.1, 7.0 Hz, 1H), 6.87 (d, *J* = 2.2 Hz, 1H), 6.83 (dd, *J* = 8.7, 2.3 Hz, 1H), 6.48 (dd, *J* = 9.1, 1.2 Hz, 1H), 6.25 (dd, *J* = 7.0, 1.2 Hz, 1H), 4.43 (t, *J* = 6.9 Hz, 2H), 3.87 (s, 3H), 3.06 (t, *J* = 7.0 Hz, 2H).

**$^{13}$C NMR** (126 MHz, $CD_3OD$) δ [ppm] = 165.0, 159.9, 141.4, 141.2, 140.2, 127.5, 121.3, 121.2, 116.9, 115.8, 111.9, 101.7, 95.3, 55.9, 42.0, 20.4.

**HRMS-ESI:** calcd. for $C_{16}H_{15}N_2O_2$ $[M + H]^+$: 267.1128; found: 267.1127.

**FT-IR:** *ν* [cm$^{-1}$] = 2922, 2853, 1651, 1567, 1540, 1457, 1375, 1271, 1201, 1161, 1143, 798.

**2-Methyl-7,12-dihydroindolo[2,3-*a*]quinolizin-4(6*H*)-one (14c).**

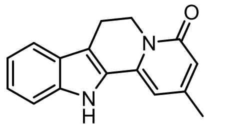

demethoxyharmalacinine (**14c**)

**14c** was prepared according to **General procedure D**, starting from diisopropylamine (0.17 mL, 1.2 mmol, 10.0 equiv.) in THF (3.0 mL) was added *n*BuLi (2.5 M in hexane, 0.48 mL, 1.2 mmol, 10.0 equiv.). A solution of **12e** (30 mg, 0.12 mmol, 1.0 equiv.) in THF (3.0 mL) was added dropwise into the resulting mixture. Then the reaction mixture was treated with $ZnCl_2$ (1.9 M in 2-Me-THF, 0.22 mL, 0.42 mmol, 3.5 equiv.). After that, the stock solution of $[Pd(allyl)Cl]_2$ (1.1 mg, 3.0 μmol, 2.5 mol%) and allyl acetate (15 μL, 0.14 mmol, 1.2 equiv.) in THF (0.5 mL) was next added. Purification by silica gel column chromatography (pentane/EtOAc = 1:2) afforded **14c** (21 mg, 84 μmol, 71%) as a yellow solid.

**$^1$H NMR** (500 MHz, $CDCl_3$+$CD_3OD$ (v/v = 1:1)) δ [ppm] = 7.82–7.76 (m, 1H), 7.65 (dd, *J* = 8.3, 1.1 Hz, 1H), 7.48 (ddd, *J* = 8.2, 7.1, 1.3 Hz, 1H), 7.34 (ddd, *J* = 8.0, 7.0,

1.1 Hz, 1H), 6.83 (d, $J$ = 1.8 Hz, 1H), 6.59–6.52 (m, 1H), 4.62 (t, $J$ = 7.0 Hz, 2H), 3.34 (t, $J$ = 6.9 Hz, 2H), 2.51 (s, 3H).

**$^{13}$C NMR** (126 MHz, $CDCl_3$+$CD_3OD$ (v/v = 1:1)) δ [ppm] = 163.2, 151.5, 138.4, 137.3, 127.0, 125.2, 123.9, 119.6, 118.9, 115.2, 113.6, 111.4, 103.7, 40.4, 20.5, 19.1.

**HRMS-ESI:** calcd. for $C_{16}H_{13}N_2O$ [M – H]$^-$: 249.1033; found: 249.1035.

**FT-IR:** $\nu$ [cm$^{-1}$] = 2924, 1649, 1583, 1568, 1543, 1499, 1364, 1148, 743.

**10-Methoxy-2-methyl-7,12-dihydroindolo[2,3-*a*]quinolizin-4(6*H*)-one (14d)**.

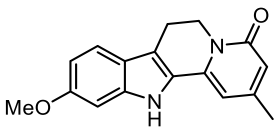


harmalacinine (**14d**)

**14d** was prepared according to **General procedure D**, starting from diisopropylamine (0.35 mL, 2.5 mmol, 15.0 equiv.) in THF (3.0 mL) was added *n*BuLi (2.5 M in hexanes, 1.0 mL, 2.5 mmol, 15.0 equiv.). A solution of **13** (47 mg, 0.17 mmol, 1.0 equiv.) in THF (3.0 mL) was added dropwise into the resulting mixture. Then the reaction mixture was treated with $ZnCl_2$ (1.9 M in 2-Me-THF, 0.44 mL, 0.83 mmol, 3.5 equiv.). After that, the stock solution of $[Pd(allyl)Cl]_2$ (0.15 mg, 4.2 μmol, 2.5 mol%) and allyl acetate (22 μL, 0.20 mmol, 1.2 equiv.) in THF (0.5 mL) was next added. Purification by silica gel column chromatography (pentane/EtOAc = 1:2) afforded **14d** (36 mg, 0.129 mmol, 77%) as a yellow solid.

**$^1$H NMR** (500 MHz, $CDCl_3$) δ [ppm] = 9.11 (s, 1H), 7.44 (d, $J$ = 8.7 Hz, 1H), 6.87 (d, $J$ = 2.2 Hz, 1H), 6.82 (dd, $J$ = 8.7, 2.3 Hz, 1H), 6.31 (t, $J$ = 1.4 Hz, 1H), 6.26 (d, $J$ = 1.7 Hz, 1H), 4.41 (t, $J$ = 6.9 Hz, 2H), 3.85 (s, 3H), 3.03 (t, $J$ = 6.9 Hz, 2H), 2.17 (s, 3H).

**$^{13}$C NMR** (126 MHz, $CDCl_3$) δ [ppm] = 163.2, 158.5, 150.2, 139.6, 137.2, 126.7 120.5, 120.4, 116.4, 115.2 111.0, 102.0, 94.9, 55.8, 40.4, 21.5, 19.9.

**HRMS-ESI:** calcd. for $C_{17}H_{17}N_2O_2$ [M + H]$^+$: 281.1284; found: 281.1284.

**FT-IR:** $\nu$ [cm$^{-1}$] 3184, 2922, 1651, 1626, 1567, 1460, 1374, 1270, 1201, 1139, 1031, 814, 753.

## 2.3.2. Optimization of regioselective dehydrogenation

With the classic dehydrogenation methods, the reactions turned out to be unsatisfying. The best result was obtained with LDA, sulfinimidoyl chloride system. We consiedered that the enolation was a reasonable strategy to afford the regioselectivity.

**Table S-1:** Dehydrogenation by classic conditions

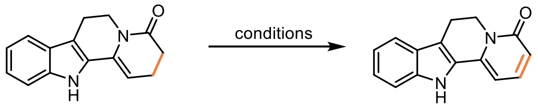


| entry | conditions | solvent | yield |
|---|---|---|---|
| 1 | $MnO_2$ | DCM | trace |
| 2 | $\gamma$-$MnO_2$ | DCM | trace |
| 3 | NBS, BPO | $CDCl_3$ | no conversion |
| 4 | S, Dowtherm® A | -- | complex mixture |
| 5 | DDQ | Dioxane | complex mixture |
| 6 | $Cu(OAc)_2$, $Na_2CO_3$, py, $O_2$ | toluene | no conversion |
| 7 | $CuBr_2$ | MeCN | no conversion |
| 8 | $Pd(OAc)_2$, $O_2$ | DMSO | trace |
| 9 | $V(acac)_2$, TFA, $Bu_4NBr$, $O_2$ | Dioxane | complex mixture |
| 10 | LiTMP, sulfinimidoyl chloride | THF | complex mixture |
| 11 | NaHMDS, sulfinimidoyl chloride | THF | trace |
| 12 | MeLi, sulfinimidoyl chloride | THF | 14% |
| 13 | LDA, PhSeCl | THF | trace |
| 14 | LDA, PhSOOMe | THF | complex mixture |
| 15 | LDA, sulfinimidoyl chloride | THF | 33% |

With the protocol reported by Newhouse et al., initially the resulted in conversion. Finally, LDA was the best base for the reaction affording 76% yield.

**Table S-2:** Optimization of Newhouse's procedure

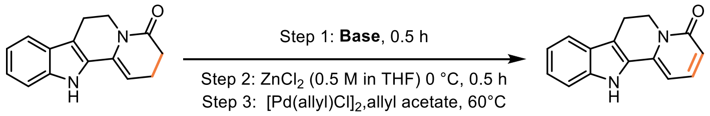


| entry | Step 1 | Step 2 | Step 3 | yield[a] |
|---|---|---|---|---|
| 1 | LiCyan (2.5 eq.), –40 °C | $ZnCl_2$ (3.5 eq.) | [Pd] (2.5 mol%), 14h | SM:P=9.8:1 |
| 2 | LiCyan (2.5 eq.), –40 °C | $ZnCl_2$ (3.5 eq.) | [Pd] (2.5 mol%), 38h | SM:P=5.1:1 |
| 3 | LiCyan (2.5 eq.), –40 °C | $ZnCl_2$ (3.5 eq.) | [Pd] (2.5 mol%), 62h | SM:P=6.5:1 |
| 4 | LiCyan (2.5 eq.), –40 °C | $ZnCl_2$ (3.5 eq.) | [Pd] (2.5 mol%), 86h | SM:P=2.1:1 |
| 5 | LiCyan (5.0 eq.), –40 °C | $ZnCl_2$ (7.0 eq.) | [Pd] (5.0 mol%), 14h | SM:P=3.9:1 |
| 6 | LiCyan (5.0 eq.), –40 °C | $ZnCl_2$ (7.0 eq.) | [Pd] (5.0 mol%), 38h | SM:P=3.8:1 |
| 7 | LiCyan (5.0 eq.), –40 °C | $ZnCl_2$ (7.0 eq.) | [Pd] (5.0 mol%), 62h | SM:P=3.4:1 |
| 8 | LiCyan (5.0 eq.), –40 °C | $ZnCl_2$ (7.0 eq.) | [Pd] (5.0 mol%), 86h | SM:P=2.3:1 |
| 9 | LiCyan (2.5 eq.), 0 °C | $ZnCl_2$ (3.5 eq.) | [Pd] (2.5 mol%), 14h | SM:P=36:1 |
| 10 | LiCyan (2.5 eq.), 0 °C | $ZnCl_2$ (3.5 eq.) | [Pd] (2.5 mol%), 75h | SM:P=9.4:1 |
| 11 | LiCyan (2.5 eq.), 0 °C | $ZnCl_2$ (3.5 eq.) | [Pd] (2.5 mol%), 96h | SM:P=9.5:1 |
| 12 | LiTMP (2.5 eq.), 0 °C | $ZnCl_2$ (3.5 eq.) | [Pd] (2.5 mol%), 14h | SM:P=4.0:1 |
| 13 | LiTMP (2.5 eq.), 0 °C | $ZnCl_2$ (3.5 eq.) | [Pd] (2.5 mol%), 75h | SM:P=2.3:1 |
| 14 | LiTMP (2.5 eq.), 0 °C | $ZnCl_2$ (3.5 eq.) | [Pd] (2.5 mol%), 96h | SM:P=2.4:1 |
| 15 | LiCyan (10 eq.), –40 °C | $ZnCl_2$ (3.5 eq.) | [Pd] (2.5 mol%), 21h | SM:P=3:1 |
| 16 | LiCyan (10 eq.), –40 °C | $ZnCl_2$ (3.5 eq.) | [Pd] (2.5 mol%), 51h | SM:P=2.9:1 |
| 17 | LiCyan (10 eq.), –40 °C | $ZnCl_2$ (3.5 eq.) | [Pd] (2.5 mol%), 88h | SM:P=1.5:1 |
| 18 | LiTMP (10 eq.), 0 °C | $ZnCl_2$ (3.5 eq.) | [Pd] (2.5 mol%), 21h | SM:P=1.7:1 |
| 19 | LiTMP (10 eq.), 0 °C | $ZnCl_2$ (3.5 eq.) | [Pd] (2.5 mol%), 51h | SM:P=2.0:1 |
| 20 | LiTMP (10 eq.), 0 °C | $ZnCl_2$ (3.5 eq.) | [Pd] (2.5 mol%), 88h | SM:P=0.5:1 |
| 21 | LDA (10 eq.), 0 °C | $ZnCl_2$ (3.5 eq.) | [Pd] (2.5 mol%), 88h | 76%[b] |

[a] ratio was determined by NMR; [b] isolated yield

2.4. Reductive pathway

**2,3,6,7,12,12*b*-Hexahydroindolo[2,3-*a*]quinolizin-4(1*H*)-one (*rac*-15)**.

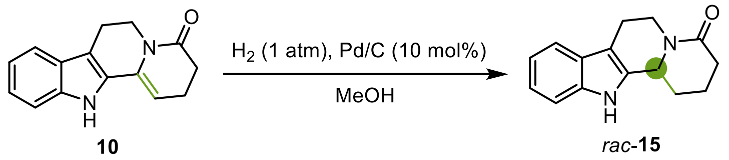


To a solution of enamide **10** (0.10 g, 0.42 mmol, 1.0 equiv.) in MeOH (10 mL), was added Pd/C (10% Pd on charcoal, 45 mg, 40 μmol, 0.1 equiv.). Then hydrogen was charged to the reaction mixture with a balloon. After the mixture was stirred overnight, it was filtered through Celite®, and concentrated under reduced pressure. Purification by silica gel column chromatography (pentane/EtOAc = 1:1) afforded ***rac*-15** (76 mg, 0.32 mmol, 75%) as a light yellow powder.

**2,3,6,7,12,12*b*-hexahydroindolo[2,3-*a*]quinolizin-4(1*H*)-one ((*S*)-15).**

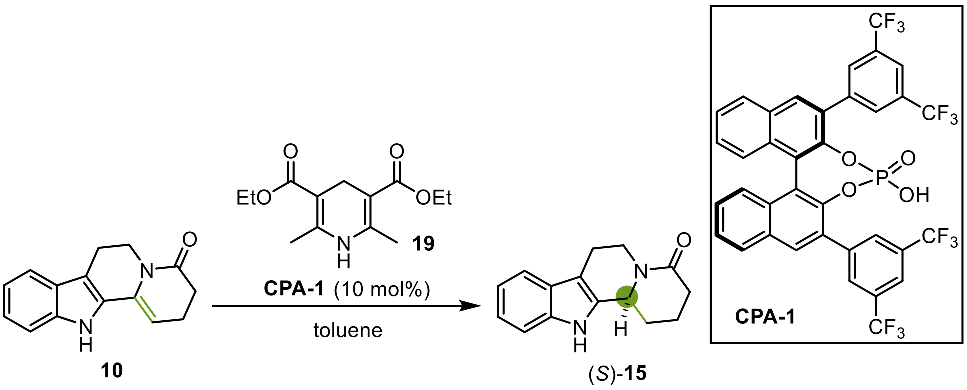


To a mixture of enamide **10** (26 mg, 0.11 mmol, 1.0 equiv.) and Hantzsch ester **19** (42 mg, 0.16 mmol, 1.5 equiv.) in toluene (2.5 mL), was added a solution of **CPA-1** (8.4 mg, 10 μmol, 0.1 equiv.) in toluene (1.0 mL). Then the reaction mixture was immediately moved to a pre-heated 60 ºC oil bath. After stirring for 24 h, the resulting mixture was quenched by $NaHCO_3$ (sat. aq.), diluted with DCM. The organic phase was separated. The aqueous phase was extracted with DCM and the combined organic layers were washed with brine, dried over $MgSO_4$, filtered, and concentrated under reduced pressure. Purification by silica gel column chromatography (pentane/EtOAc = 1:1) afforded **(*S*)-15** (16 mg, 70 μmol, 61%, 99% (b.r.s.m.), 80% *ee*) as a light yellow powder.

**$^{1}$H NMR** (500 MHz, $CDCl_3$) δ [ppm] = 8.09 (s, 1H), 7.51 (dd, *J* = 7.8, 1.1 Hz, 1H), 7.34 (dt, *J* = 8.0, 0.9 Hz, 1H), 7.18 (ddd, *J* = 8.2, 7.0, 1.2 Hz, 1H), 7.13 (ddd, *J* = 8.0, 7.0, 1.0 Hz, 1H), 5.23–5.14 (m, 1H), 4.82–4.75 (m, 1H), 2.87 (m, 2H), 2.81–2.73 (m, 1H), 2.62–2.57 (m, 1H), 2.50–2.43 (m, 1H), 2.40 (td, *J* = 11.6, 5.8 Hz, 1H), 2.02–1.93 (m, 1H), 1.92–1.83 (m, 1H), 1.83–1.73 (m, 1H).

**$^{13}$C NMR** (126 MHz, $CDCl_3$) δ [ppm] = 169.5, 136.3, 133.4, 127.0, 122.3, 120.0, 118.6, 111.1, 109.8, 54.6, 40.4, 32.5, 29.2, 21.1, 19.5.

**HRMS-EI:** calcd. for $C_{15}H_{16}N_2O$ $[M]^+$: 240.1257; found: 240.1259.

**FT-IR:** *v* [cm$^{-1}$] = 3223, 2923, 2853, 1615, 1470, 1445, 1415, 1266, 1235, 741.

$[\alpha]_D^{26}$ = –191.2 (*c* = 0.8, $CHCl_3$); Lit.[2]: $[\alpha]_D^{25}$ = –220.2 (*c* = 0.10 $CHCl_3$) (94% *ee*).

**HPLC:** 10% EtOH/Hexane, Chiralpak IA, 1.0 mL/min

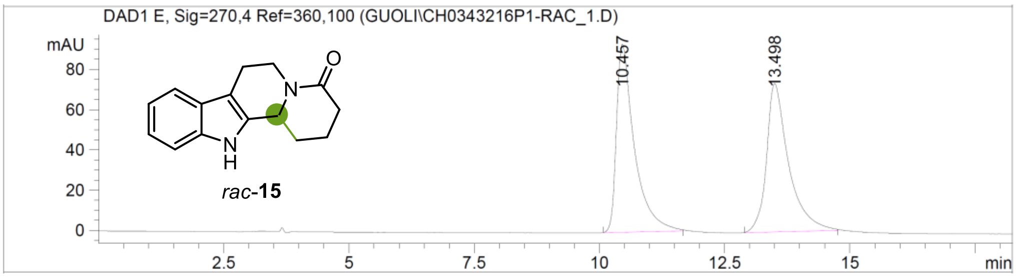


Signal 3: DAD1 E, Sig=270,4 Ref=360,100

| Peak # | RetTime [min] | Type | Width [min] | Area [mAU*s] | Height [mAU] | Area % |
|---|---|---|---|---|---|---|
| 1 | 10.457 | BB | 0.3627 | 2324.59595 | 93.30048 | 50.6335 |
| 2 | 13.498 | BB | 0.4409 | 2266.42432 | 73.67532 | 49.3665 |
| Totals : | | | | 4591.02026 | 166.97581 | |

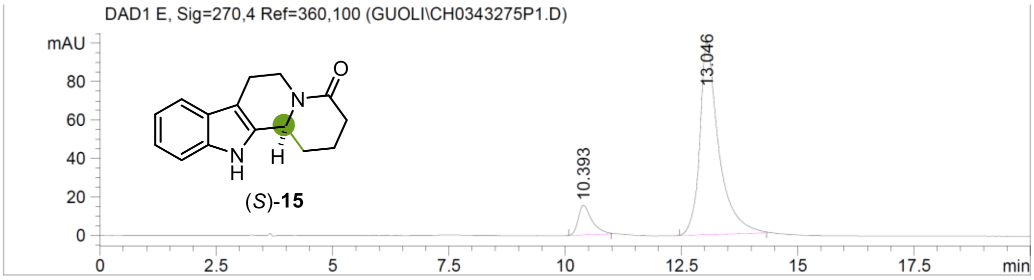


[2] P. Mondal, N. P. Argade *J. Org. Chem.* **2013**, *78*, 6802−6808

Signal 3: DAD1 E, Sig=270,4 Ref=360,100

| Peak # | RetTime [min] | Type | Width [min] | Area [mAU*s] | Height [mAU] | Area % |
|---|---|---|---|---|---|---|
| 1 | 10.393 | BB | 0.3036 | 316.36719 | 15.40351 | 10.0180 |
| 2 | 13.046 | BB | 0.4101 | 2841.63501 | 99.83503 | 89.9820 |
| Totals : | | | | 3158.00220 | 115.23854 | |

**6,7,12,12*b*-Tetrahydroindolo[2,3-*a*]quinolizin-4(1*H*)-one (16).**[3]

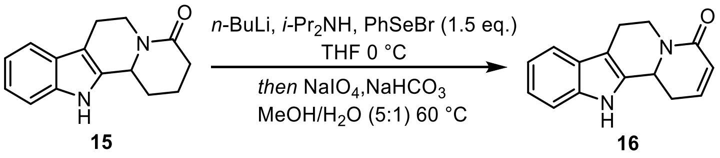


Diisopropylamine (234 µL, 1.67 mmol, 10.0 equiv.) was added to dry THF (5.0 mL) at 0 °C. *n*BuLi (2.5 M in hexane, 667 µL, 1.67 mmol, 10.0 equiv.) was added and the mixture was stirred for 30 min before cooling to –78 °C. A solution of *rac*-**15** (40.0 mg, 0.167 mmol, 1.0 equiv.) in THF (2.0 mL) was added dropwise. The reaction was stirred at –78 °C for 1 h. A solution of PhSeBr (59.0 mg, 250 µmol, 1.5 equiv.) in THF (2.0 mL) was the added dropwise and the reaction mixture was stirred for 24 h. The reaction was quenched by $NH_4Cl$ (sat. aq.) and extracted with DCM. The combined organic phases were washed with brine, dried over $MgSO_4$, filtered and concentrated under reduced pressure. The crude selenide was dissolved in methanol (5.0 mL) and water (1.0 mL). $NaIO_4$ (71.0 mg, 333 µmol, 2.0 equiv.) and $NaHCO_3$ (16.8 mg, 200 µmol, 1.2 equiv.) were added and the reaction was heated at 60 °C for 24 h. The reaction was quenched by $NaHCO_3$ (sat. aq.) and extracted with DCM. The combined organic phases were washed with brine, dried over $MgSO_4$ and concentrated under reduced pressure. The crude product was purified by silica gel column chromatography (pentane/EtOAc = 1:1) to give **16** (16.0 mg, 67.2 µmol, 40%) as light yellow powder.

**$^1$H NMR** (500 MHz, $d_6$-DMSO) δ [ppm] = 10.96 (s, 1H), 7.44 (dd, *J* = 7.9, 1.1 Hz, 1H), 7.33 (dt, *J* = 8.0, 0.9 Hz, 1H), 7.08 (ddd, *J* = 8.2, 7.1, 1.2 Hz, 1H), 6.99 (ddd, *J* = 8.0, 7.1, 1.0 Hz, 1H), 6.81 (ddd, *J* = 9.7, 6.2, 2.1 Hz, 1H), 5.92 (dd, *J* = 9.7, 2.8 Hz, 1H), 4.91–4.82 (m, 1H), 4.78 (ddd, *J* = 13.6, 5.2, 1.9 Hz, 1H), 3.02 (ddd, *J* = 17.7, 6.3, 4.9

[3] P. Horrocks, S. Fallon, L. Denman, O. Devine, L. J. Duffy, A. Harper, E. Meredith, S. Hasenkamp, A. Sidaway, D. Monnery, T.R. Phillips, S. M. Allin, *Bioorg. Med. Chem. Lett* . **2012**, *22*, 1770–1773.

Hz, 1H), 2.88–2.75 (m, 2H), 2.67 (m, 1H), 2.32–2.19 (m, 1H).

**$^{13}$C NMR** (126 MHz, $d_6$-DMSO) δ [ppm] = 164.1, 139.4, 136.3, 133.6, 126.1, 124.7, 121.1, 118.6, 117.9, 111.1, 107.0, 51.2, 38.2, 30.3, 20.5.

**HRMS-EI:** calcd. for $C_{15}H_{14}N_2O$ $[M]^+$: 238.1100; found: 238.1099.

**FT-IR:** *v* [cm$^{-1}$] = 3780, 2918, 2849, 1737, 1655, 1601, 1436, 1324, 1303, 817, 740.

2.5. Total synthesis of pegamunine A

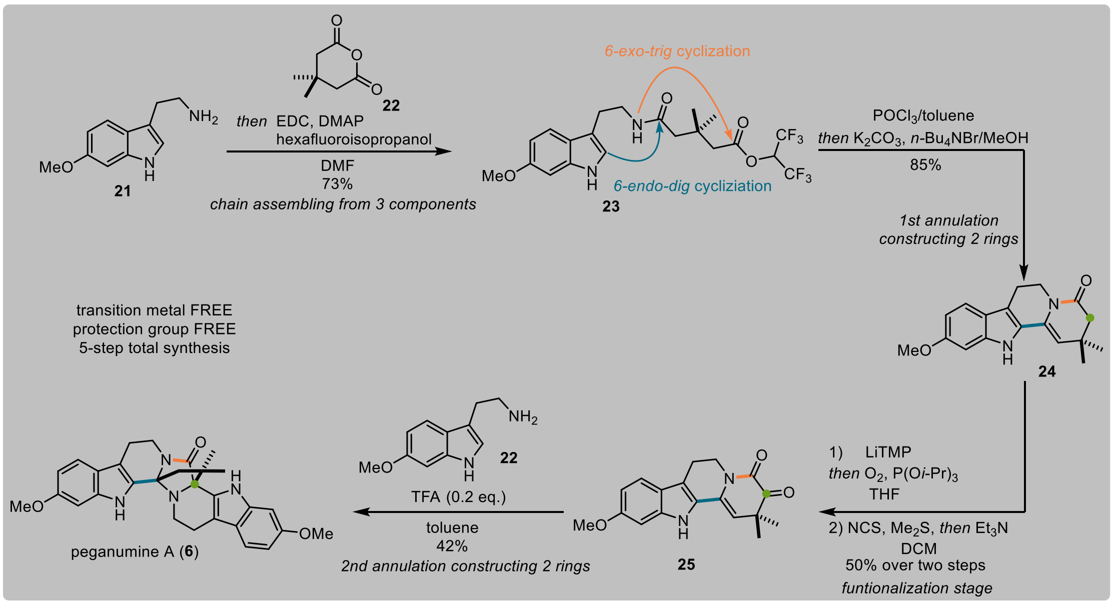


## 2.5.1. Preparation of ketoamide

**1,1,1,3,3,3-Hexafluoropropan-2-yl 5-((2-(6-methoxy-1*H*-indol-3-yl)ethyl)amino)-3,3-dimethyl-5-oxopentanoate (24)**.

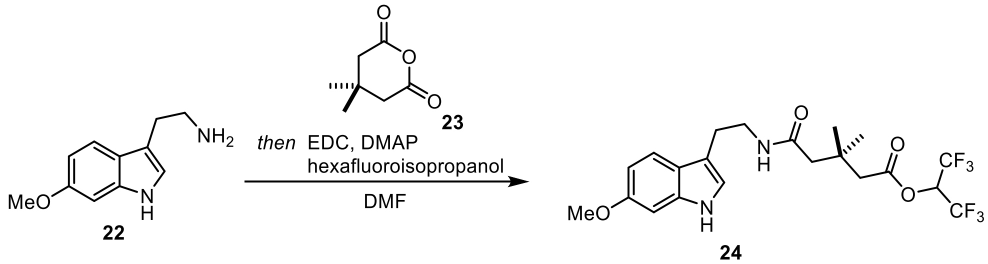


**24** was prepared according to **General procedure A**, starting from 6-methoxytryptamine (200 mg, 1.05 mmol, 1.0 equiv.) and 3,3-dimethylglutaric anhydride (150 mg, 1.05 mmol, 1.0 equiv.) in DMF (5.0 mL). The reaction mixture was subsequently treated with EDCI (402 mg, 2.11 mmol, 2.0 equiv.), DMAP (25.7 mg, 0.211 mmol, 0.2 equiv.) and HFIP (556 μL, 5.26 mmol, 5.0 equiv.). Purification by silica gel column chromatography (pentane/EtOAc = 3:2) afforded **24** (370 mg, 0.768 mmol, 73%) as a yellow solid.

**$^1$H NMR** (500 MHz, $CD_2Cl_2$) δ [ppm] = 8.13 (s, 1H), 7.45 (d, *J* = 8.6 Hz, 1H), 6.92 (dt, *J* = 2.1, 1.0 Hz, 1H), 6.86 (d, *J* = 2.2 Hz, 1H), 6.75 (dd, *J* = 8.6, 2.3 Hz, 1H), 5.86–5.78

(m, 1H), 5.75 (s, 1H), 3.82 (s, 3H), 3.54 (td, $J$ = 6.9, 5.7 Hz, 2H), 2.94–2.87 (m, 2H), 2.62 (s, 2H), 2.15 (s, 2H), 1.08 (s, 6H).

**$^{13}$C NMR** (126 MHz, $CD_2Cl_2$) δ [ppm] = 170.7, 169.5, 157.1, 137.7, 122.2, 121.2, 119.6, 113.3, 109.8, 94.9, 66.7–66.2 (m), 55.9, 47.5, 44.0, 39.9, 33.5, 27.9, 25.8. (Due to the resolution or/and overlapping, the resonance of $CF_3$ is missing.)

**$^{19}$F NMR** (471 MHz, $CD_2Cl_2$) δ [ppm] = –73.47.

**HRMS-EI**: calcd. for $C_{21}H_{24}F_6N_2O_4Na$ $[M + Na]^+$: 505.1532; found: 505.1533.

**FT-IR:** $v$ [cm$^{-1}$] = 2969, 2941, 1766, 1737, 1649, 1456, 1360, 1289, 1200, 1109, 771.

**10-Methoxy-2,2-dimethyl-2,6,7,12-tetrahydroindolo[2,3-*a*]quinolizin-4(3*H*)-one (25).**

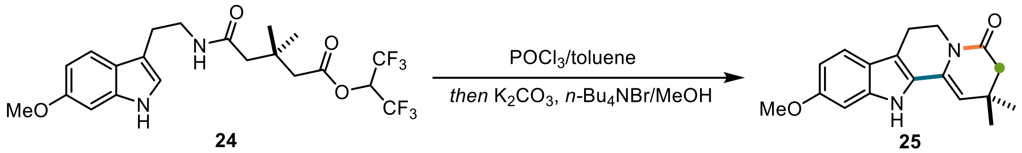


**25** was prepared according to **General procedure C**, starting from **24** (100 mg, 0.207 mmol, 1.0 equiv.) and $POCl_3$ (18.9 μL, 0.207 mmol, 1.0 equiv.) in toluene (3.0 mL). The reaction mixture was subsequently treated with MeOH (3.0 mL), $K_2CO_3$ (287 mg, 2.08 mmol, 10 equiv.) and $n$$Bu_4NBr$ (6.7 mg, 0.021 mmol, 0.1 equiv.). Purification by silica gel column chromatography (pentane/EtOAc = 2:1) afforded **25** (49.0 mg, 0.165 mmol, 85%) as a yellow solid.

**$^1$H NMR** (700 MHz, $CDCl_3$) δ [ppm] = 8.09 (s, 1H), 7.37 (d, $J$ = 8.5 Hz, 1H), 6.83 (s, 1H), 6.78 (d, $J$ = 8.5 Hz, 1H), 5.27 (s, 1H), 4.09 (t, $J$ = 5.9 Hz, 2H), 3.85 (s, 3H), 2.88 (t, $J$ = 5.9 Hz, 2H), 2.48 (s, 2H), 1.16 (s, 6H).

**$^{13}$C NMR** (176 MHz, $CDCl_3$) δ [ppm] = 169.6, 157.6, 138.3, 128.9, 126.9, 121.3, 119.7, 112.4, 109.7, 109.5, 95.0, 55.8, 46.6, 39.3, 30.8, 28.3, 20.8.

**HRMS-EI**: calcd. for $C_{18}H_{20}N_2O_2$ $[M]^+$: 296.1519; found: 296.1520.

**FT-IR:** $v$ [cm$^{-1}$] = 2954, 2919, 2850, 1736, 1663, 1628, 1461, 1365, 1230, 12174, 1149.

**3-Hydroxy-10-methoxy-2,2-dimethyl-2,6,7,12-tetrahydroindolo[2,3-*a*]quinolizin-4(3*H*)-one (S15)**.

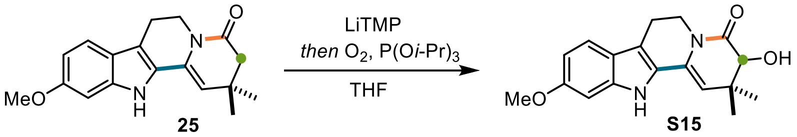


To a solution of tetracyclic lactam **25** (100 mg, 0.34 mmol, 1.0 equiv.) in THF, was added LiTMP (1.0 M solution in THF, 3.40 mL, 3.38 mmol, 10.0 equiv.) at –78 °C. The resulting mixture was stirred at that temperature for 1 h before $P(O\textit{i}Pr)_3$ (250 µL, 1.01 mmol, 2.5 equiv.) was added. $O_2$ was bubbled through the mixture for 20 min, and the mixture was stirred for additional 2 h under $O_2$ atmosphere. Then the reaction mixture was quenched with saturated $NaHCO_3$ (sat. aq.). The aqueous phase was extracted with DCM. The combined organic phases were washed with brine, dried with $MgSO_4$, filtered and concentrated under reduced pressure. The crude product was purified by silica gel column chromatography (pentane/EtOAc = 3:2) afforded **S15** (75.0 mg, 0.240 mmol, 71%) as yellow solid.

**$^1$H NMR** (500 MHz, $CDCl_3$) δ [ppm] = 8.09 (s, 1H), 7.37 (d, *J* = 8.6 Hz, 1H), 6.82 (d, *J* = 2.2 Hz, 1H), 6.79 (dd, *J* = 8.6, 2.2 Hz, 1H), 5.27 (s, 1H), 4.86 (ddd, *J* = 12.7, 5.2, 2.1 Hz, 1H), 4.08 (d, *J* = 2.5 Hz, 1H), 3.89 (d, *J* = 2.6 Hz, 1H), 3.85 (s, 3H), 3.28 (td, *J* = 12.1, 5.4 Hz, 1H), 2.96–2.83 (m, 2H), 1.30 (s, 3H), 1.00 (s, 3H).

**$^{13}$C NMR** (126 MHz, $CDCl_3$) δ [ppm] = 171.7, 157.9, 138.4, 127.8, 126.1, 121.2, 119.8, 112.4, 110.1, 109.3, 94.9, 75.0, 55.8, 40.7, 35.2, 26.5, 20.6, 19.9.

**HRMS-ESI**: calcd. for $C_{18}H_{20}N_2O_3Na$ $[M + Na]^+$: 335.1366; found: 335.1367.

**FT-IR:** *ν* [$cm^{-1}$] = 3343, 2958, 2924, 1653, 1393, 1360, 1328, 1231, 1151, 1045, 1027, 920, 743.

**10-Methoxy-2,2-dimethyl-2,6,7,12-tetrahydroindolo[2,3-*a*]quinolizine-3,4-dione (26)**.[4]

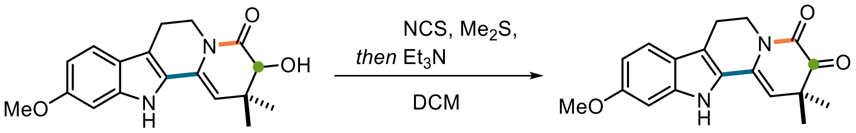


**S15** **26**

To a solution of NCS (171 mg, 1.28 mmol, 5.0 equiv.) in DCM (5.0 mL) at 0 °C was added $Me_2S$ (471 µL, 6.41 mmol, 25 equiv.) and the mixture was stirred at –78 °C for 1 h. The **S15** (80 mg, 0.256 mmol, 1.0 equiv.) in DCM (2.0 mL) was added dropwise. The resulting mixture was stirred at –78 °C for another two hours before triethylamine (712 µL, 5.13 mmol, 20 equiv.) was added. Then the mixture was stirred at –78 °C for 2 h. The mixture was quenched with $NH_4Cl$ (sat. aq.) and extracted with DCM. The combined organic phases were washed with brine, dried over $MgSO_4$, filtered and concentrated under reduced pressure. The crude product was purified by silica gel column chromatography (pentane/EtOAc = 3:2) afforded **26** (57.0 mg, 0.184 mmol, 71%) as an orange solid.

**$^1$H NMR** (700 MHz, $CDCl_3$) δ [ppm] = 8.08 (s, 1H), 7.42–7.37 (m, 1H), 6.87–6.84 (m, 1H), 6.80 (dd, *J* = 8.6, 2.2 Hz, 1H), 5.31 (s, 1H), 4.20 (t, *J* = 6.0 Hz, 2H), 3.86 (s, 3H), 2.98 (t, *J* = 6.0 Hz, 2H), 1.39 (s, 6H).

**$^{13}$C NMR** (176 MHz, $CDCl_3$) δ [ppm] = 196.1, 158.1, 157.4, 138.7, 127.0, 125.9, 121.0, 120.0, 113.0, 110.3, 106.0, 95.1, 55.9, 44.9, 40.5, 25.0, 20.4.

**HRMS-EI**: calcd. for $C_{18}H_{18}N_2O_3$ $[M]^+$: 310.1312; found: 310.1314.

**FT-IR**: *v* [$cm^{-1}$] = 3284(br), 2956, 2925, 1642, 1392, 1305, 1184, 1143, 1047, 799, 756.

2.5.2. Racemic protection group free annulation reaction

**Imine pre-formation one-pot procedure.**[4]

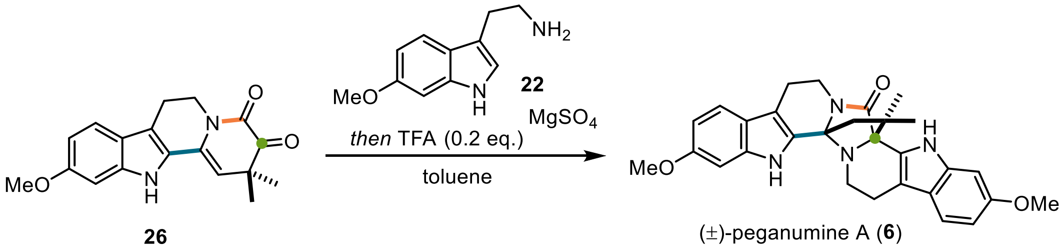


A mixture of ketoenamide **25** (56.0 mg, 0.181 mmol, 1.0 equiv.), 6-methoxytryptamine (**22**) (41.2 mg, 0.217 mmol, 1.2 equiv.) and $MgSO_4$ (217 mg, 1.81 mmol, 10.0 equiv.) in toluene (10 mL) was heated to 115 ºC (*oil bath temperature*) overnight. The reaction

[4] C. Piemontesi, Q. Wang, J. Zhu *J. Am. Chem. Soc.* **2016**, *138*, 35, 11148–11151

mixture was cooled to room temperature and TFA (2.8 µL, 36 µmol, 0.2 equiv.) were added. The mixture was then heated to reflux for 2 days, before the reaction was quenched with saturated $NaHCO_3$ (sat. aq.). The aqueous phase was extracted with DCM. The combined organic phases were washed with brine, dried with $MgSO_4$, filtered and concentrated under reduced pressure. The crude product was purified by silica gel column chromatography (pentane/EtOAc = 3:2) afforded **6** (34.0 mg, 70.5 µmol, 39%) as light yellow solid.

**Cascade procedure.**

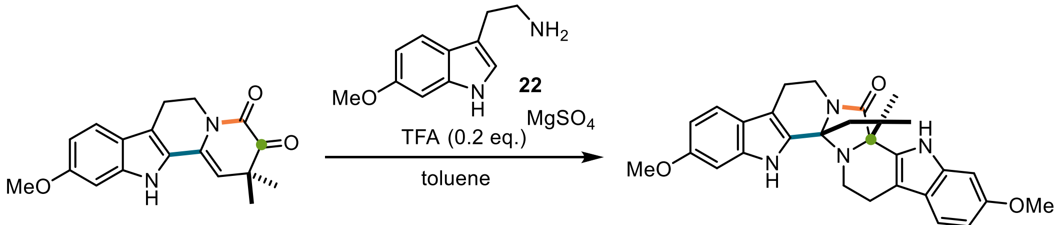


To a mixture of ketoenamide **25** (35.0 mg, 0.113 mmol, 1.0 equiv.), 6-methoxytryptamine (**22**) (25.7 mg, 0.135 mmol, 1.2 equiv.) and $MgSO_4$ (136 mg, 1.13 mmol, 10.0 equiv.) in 10 mL toluene under argon atmosphere, was added TFA (1.7 µL, 23 µmol, 0.2 equiv.). The resulting reaction mixture was heated to 115 ºC (oil bath temperature) for 2 days. Then the reaction was quenched by $NaHCO_3$ (sat. aq.). The aqueous phase was extracted with DCM. The combined organic phases were washed with brine, dried with $MgSO_4$, filtered and concentrated under reduced pressure. The crude product was purified by silica gel column chromatography (pentane/EtOAc = 3:2) afforded **6** (23.0 mg, 47.7 µmol, 42%) as a light yellow solid.

### 2.5.3. Enantioselective protection group free annulation reaction

#### 2.5.3.1. Catalyst preparation

**S17** was prepared from **S16** by a procedure of Lee et al..[5] The spectroscopic data are in agreement with literature. **28** was synthesized from **S17** following the procedure of Seidel et al..[6] The spectroscopic data are in agreement with literature.

[5] P. S. Akula, B.-C. Hong, G.-H. Lee *Org. Lett.* **2018**, *20*, 7835–7839.
[6] C. Min, N. Mittal, D. X. Sun, D. Seidel, *Angew. Chem. Int. Ed.* **2013**, *52*, 14084–14088.

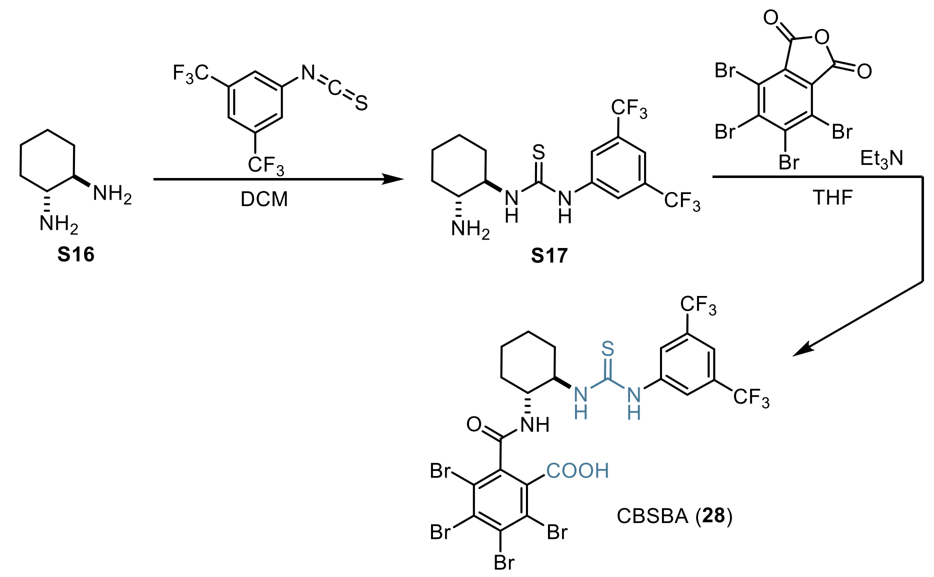


**Scheme S-3:** Synthesis of CBSBA.

1,2,3,4,5-pentacarboxycyclopentadiene (PCCP) derived pentamenthyl ester **29** was synthesized from **S18** according to a procedure by Lambert et al..[7] The spectroscopic data are in agreement with literature.

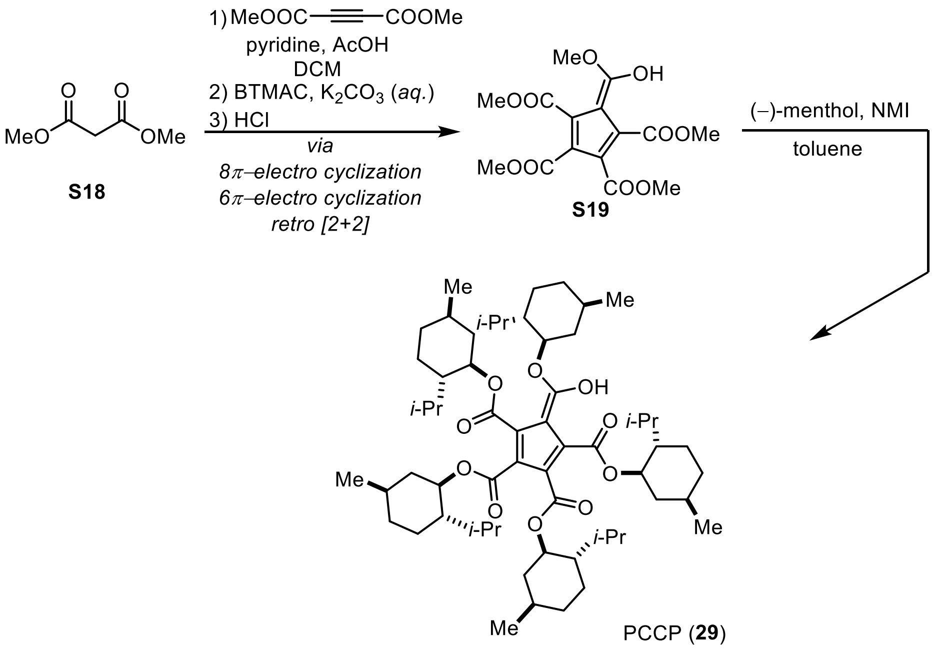


**Scheme S-4:** Synthesis of PCCP.

## Chiral DSIs:

Generally, the synthesis of **S20** needs 7 steps from BINOL. With the **S14** in hand, DSIs could be obtained by Suzuki coupling with boronic acid.[8]

[7] M. A. Radtke, C. C. Dudley, J. M. O'Leary, T. H. Lambert, *Synthesis* **2019**, *51*, 1135–1138.
[8] S. Gandhi, B. List *Angew. Chem. Int. Ed.* **2013**, *52*, 2573–2576.

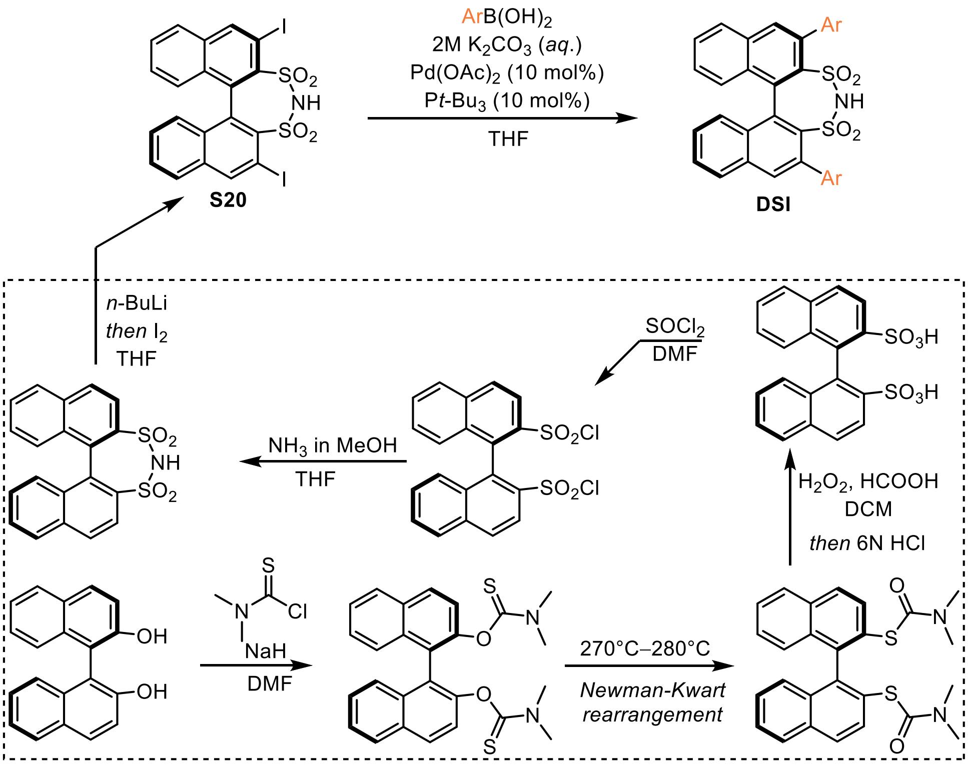


**Scheme S-5:** Synthesis of DSIs.

The readily available DSIs:

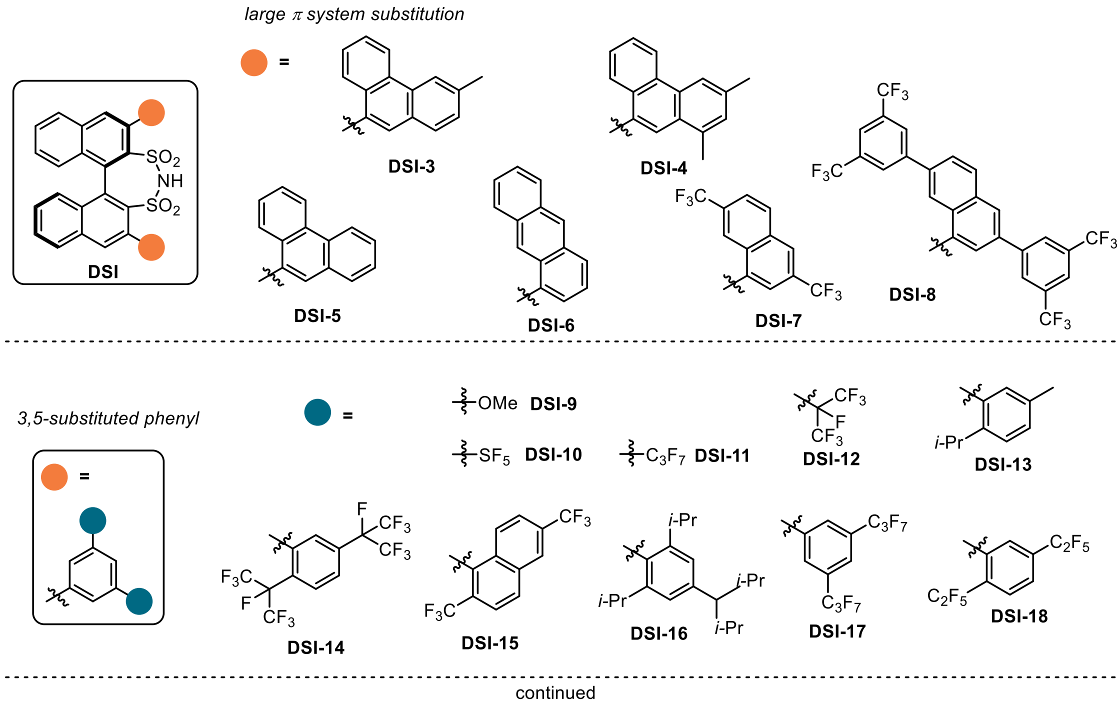

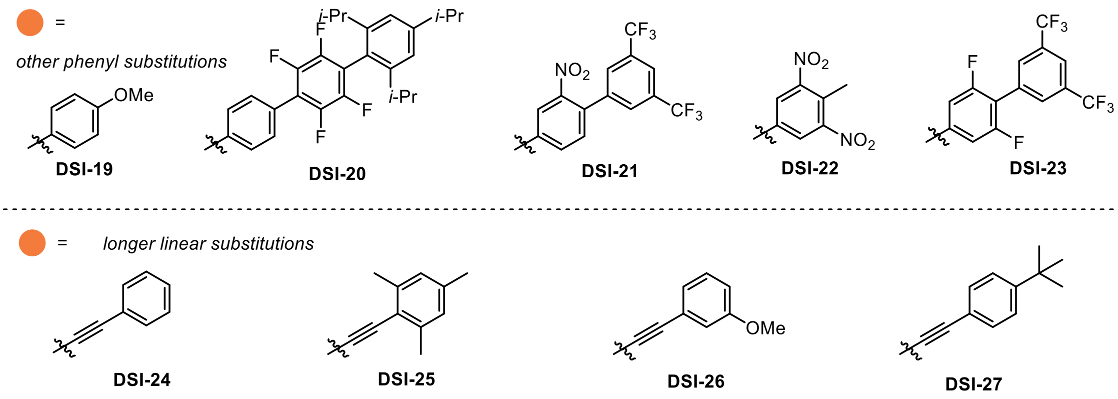


**Figure S-1:** Screened the readily available DSIs.

## General procedure E

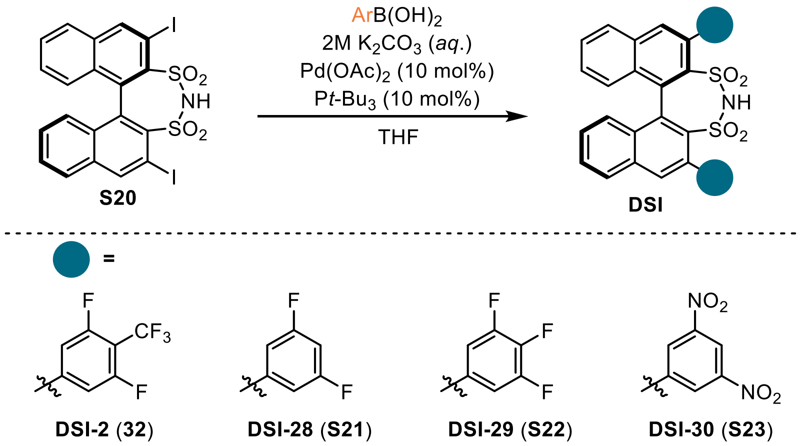


2M $K_2CO_3$ was added to a solution of (*R*)-DSI iodide (**S20**) (1.0 equiv.), boronic acid (3.0 equiv.), $Pd(OAc)_2$ (0.1 equiv.) in THF at room temperature. The reaction flask was degassed and filled with argon three times before P*t*-$Bu_3$ (1.0 M in toluene, 0.1 equiv.) was added. The reaction mixture was then put to a preheated 85 ºC oil bath for 24 h. 10% aqueous HCl solution was added and the reaction mixture was extracted three times with DCM. The combined organic layers were dried over $MgSO_4$ and concentrated under reduced pressure. The residue was purified by column chromatography on silica gel (pentane/EtOAc = 3:2) to give a white solid which was dissolved in DCM and stirred with 4N HCl for 1 h. The organic layer was then separated, dried over $MgSO_4$ and concentrated under reduced pressure to give the desired compound as a white solid.

**(*R*)-2,6-Bis(3,5-difluorophenyl)-4*H*-dinaphtho[2,1-d:1',2'-*f*][1,3,2]dithiazepine 3,3,5,5-tetraoxide (S21)**.

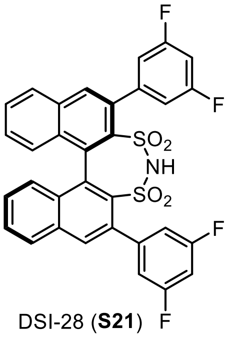


DSI-28 (**S21**)

**S21** was prepared according to **General procedure E**, starting from **S20** (50 mg, 77 µmol, 1.0 equiv.), (3,5-difluorophenyl)boronic acid (38 mg, 0.23 mmol, 3.0 equiv.) and $Pd(OAc)_2$ (1.7 mg, 8.0 µmol, 0.1 equiv.) in THF (3.0 mL). After degassing, the mixture was treated with P*t*-$Bu_3$ (1.0 M in toluene, 7.7 µL, 8.0 µmol, 0.1 equiv.). Purification by silica gel column chromatography (pentane/EtOAc = 2:1) afforded **S21** (28 mg, 45 µmol, 58%) as a white solid.

**$^1$H NMR** (500 MHz, $CDCl_3$) δ [ppm] = 8.02 (d, *J* = 9.0 Hz, 4H), 7.72 (ddd, *J* = 8.2, 6.8, 1.1 Hz, 2H), 7.45 (ddd, *J* = 8.4, 6.9, 1.2 Hz, 2H), 7.16 (d, *J* = 8.6 Hz, 2H), 7.08 (d, *J* = 8.6 Hz, 2H), 6.99 (d, *J* = 8.6 Hz, 2H), 6.90–6.85 (m, 2H), 5.76 (s, 1H).

**$^{13}$C NMR** (126 MHz, $CDCl_3$) δ [ppm] = 162.6, 162.5, 162.1, 162.0, 160.6, 160.5, 160.1, 160.0, 141.2, 141.1, 141.0, 137.5, 133.6, 133.4, 132.6, 131.1, 130.8, 129.4, 128.0, 127.7, 127.2, 113.1, 112.9, 111.3, 111.1, 102.8, 102.6, 102.4. (Due to the complexity of the F–C coupling, the coupling constants were not calculated.)

**$^{19}$F NMR** (471 MHz, $CDCl_3$) δ [ppm] = –109.97, –110.59.

**HRMS-ESI:** calcd. for $C_{32}H_{16}F_4NO_4S_2$ [M – H]$^-$: 618.0462; found: 618.0472.

**FT-IR**: *v* [cm$^{-1}$] = 3158, 2927, 1617, 1529, 1439, 1423, 1362, 1340, 1319, 1044, 845, 751.

**(*R*)-2,6-Bis(3,4,5-trifluorophenyl)-4*H*-dinaphtho[2,1-d:1',2'-*f*][1,3,2]dithiazepine 3,3,5,5-tetraoxide (S22)**.

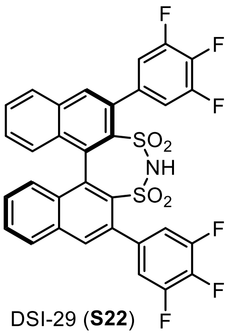


DSI-29 (**S22**)

**S22** was prepared according to **General procedure E**, starting from **S20** (50 mg, 77 µmol, 1.0 equiv.), (3,4,5-trifluorophenyl)boronic acid (41 mg, 0.23 mmol, 3.0 equiv.) and $Pd(OAc)_2$ (1.7 mg, 8.0 µmol, 0.1 equiv.) in THF (3.0 mL). After degassing, the mixture was treated with P*t*-$Bu_3$ (1.0 M in toluene, 7.7 µL, 8.0 µmol, 0.1 equiv.). Purification by silica gel column chromatography (pentane/EtOAc = 2:1) afforded **S23** (36 mg, 54 µmol, 70%) as a white solid.

**$^1$H NMR** (500 MHz, $CDCl_3$) δ [ppm] = 8.02 (d, *J* = 8.7 Hz, 4H), 7.74 (ddd, *J* = 8.2, 6.9, 1.1 Hz, 2H), 7.46 (ddd, *J* = 8.4, 6.9, 1.3 Hz, 2H), 7.20–7.01 (m, 6H).

**$^{13}$C NMR** (126 MHz, $CDCl_3$) δ [ppm] = 151.8, 151.5, 149.9, 149.8, 149.5, 149.5, 138.7, 135.0, 134.9, 134.9, 134.9, 134.8, 134.8, 134.5, 134.0, 133.9, 132.3, 131.9, 130.7, 129.3, 128.8, 128.3, 115.3, 115.1, 113.6, 113.5. (Due to the complexity of the F–C coupling, the coupling constants were not calculated.)

**$^{19}$F NMR** (471 MHz, $CDCl_3$) δ [ppm] = (–138.15)–(–140.64) (m), –167.04 (t, *J* = 19.9 Hz).

**HRMS-ESI:** calcd. for $C_{32}H_{14}F_6NO_4S_2$ $[M – H]^-$: 654.0274; found: 654.0282.

**FT-IR**: *ν* [$cm^{-1}$] = 3161, 2924, 1738, 1618, 1529, 1424, 1362, 1217, 1045, 848, 750.

**(*R*)-2,6-Bis(3,5-dinitrophenyl)-4*H*-dinaphtho[2,1-d:1',2'-*f*][1,3,2]dithiazepine 3,3,5,5-tetraoxide (S23)**.

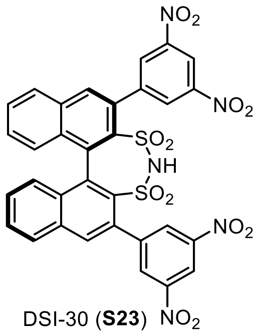


DSI-30 (**S23**)

**S23** was prepared according to **General procedure E**, starting from **S20** (15 mg, 23 µmol, 1.0 equiv.), (3,5-dinitrophenyl)boronic acid (15 mg, 0.70 mmol, 3.0 equiv.) and $Pd(OAc)_2$ (0.50 mg, 2.0 µmol, 0.1 equiv.) in THF (1.0 mL). After degassing, the mixture was treated with P*t*-$Bu_3$ (1.0 M in toluene, 2.3 µL, 2.0 µmol, 0.1 equiv.). Purification by silica gel column chromatography (pentane/EtOAc = 2:1) afforded **S23** (10 mg, 14 µmol, 59%) as a white solid.

**$^1$H NMR** (500 MHz, $CD_2Cl_2$) δ [ppm] = 9.05 (t, *J* = 2.1 Hz, 2H), 8.76 – 8.61 (m, 4H), 8.12 (d, *J* = 6.6 Hz, 4H), 7.82 (t, *J* = 7.6 Hz, 2H), 7.55 (dd, *J* = 8.8, 6.8 Hz, 2H), 7.25 (d, *J* = 8.6 Hz, 2H).

**$^{13}$C NMR** (126 MHz, $CD_2Cl_2$) δ [ppm] = 148.2, 147.6, 142.7, 138.9, 134.6, 134.5, 132.5, 132.1, 131.8, 131.2, 130.9, 130.0, 129.3, 129.2, 128.4, 118.5.

**HRMS-ESI:** calcd. for $C_{32}H_{16}N_5O_{12}S_2$ $[M - H]^-$: 726.0256; found: 726.0242.

**FT-IR**: *ν* [$cm^{-1}$] = 2924, 1738, 1541, 1343, 1175, 831, 750.

**(*R*)-2,6-Bis(3,5-difluoro-4-(trifluoromethyl)phenyl)-4*H*-dinaphtho[2,1-d:1',2'-*f*][1,3,2]dithiazepine 3,3,5,5-tetraoxide (32)**.

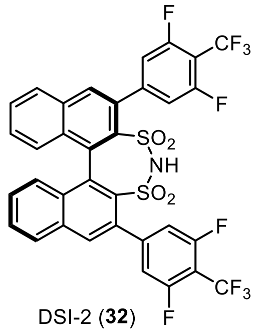


DSI-2 (**32**)

**32** was prepared according to **General procedure E**, starting from **S20** (300 mg, 0.464 mmol, 1.0 equiv.), (3,5-difluoro-4-(trifluoromethyl)phenyl)boronic acid (315 mg, 1.39 mmol, 3.0 equiv.) and $Pd(OAc)_2$ (10.4 mg, 46.4 µmol, 0.1 equiv.) in THF (15 mL). After degassing, the mixture was treated with P*t*-$Bu_3$ (1.0 M in toluene, 46.4 µL, 46.4 µmol, 0.1 equiv.). Purification by silica gel column chromatography (pentane/EtOAc = 2:1) afforded **32** (320 mg, 0.424 mmol, 91%) as a white solid.

**$^1$H NMR** (500 MHz, $CDCl_3$) δ [ppm] = 8.07–7.97 (m, 4H), 7.76 (t, *J* = 7.6 Hz, 2H), 7.54–7.46 (m, 2H), 7.24–7.07 (m, 6H).

**$^{13}$C NMR** (126 MHz, $CDCl_3$) δ [ppm] = 160.4, 159.9, 158.3, 157.8, 145.6, 138.5, 134.2, 133.5, 133.1, 132.1, 131.4, 130.8, 129.5, 128.9, 128.2, 122.8, 120.7, 115.2, 115.0, 113.6, 113.5. (Due to the complexity of the F–C coupling, the coupling constants were not calculated.)

**$^{19}$F NMR** (471 MHz, $CDCl_3$) δ [ppm] = –56.16 (t, *J* = 21.7 Hz), –111.17 (dq, *J* = 329.9, 22.4, 21.7 Hz).

**HRMS-ESI:** calcd. for $C_{34}H_{14}F_{10}NO_4S_2$ $[M-H]^-$: 754.0219; found: 754.0210.

**FT-IR**: *v* [$cm^{-1}$] = 3075, 1641, 1493, 1429, 1352, 1306, 1137, 1046, 856, 753.

2.5.3.2. Optimization of enantioselective protection group free cascade

**General Procedure for the small scale (0.50 mg or 1.00 mg) screening reactions**

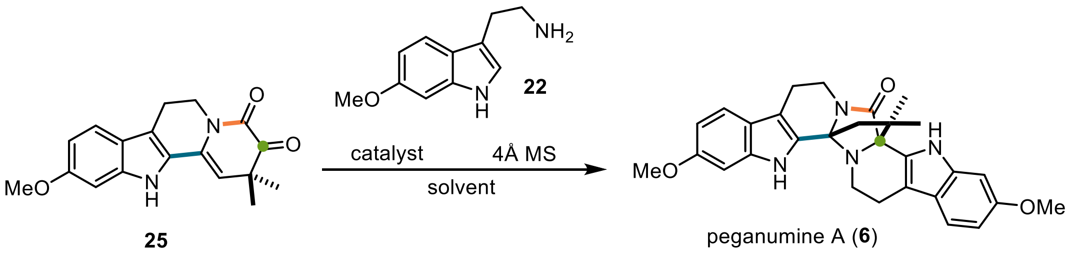


A mixture of ketoamide **25** (1.00 mg, 3.00 µmol, 1.0 equiv.), 6-methoxytryptamine (**22**) (0.75 mg, 3.60 µmol, 1.2 equiv.) and 4 Å MS in corresponding solvent (0.5 mL) was stirred at room temperature under argon in sealed vial. A solution of corresponding catalyst in the same solvent was added to the reaction mixture and the reaction mixture was heated up to the corresponding temperature for the set duration. The reaction

mixture was cooled to room temperature and purified by silica gel column chromatography (pentane/EtOAc = 3:2) to yield the pure product as solid.

(Note: since the ketoamide is not a fine power and cannot dissolve in toluene (the frequently used solvent), the practical way to prepare the 1.00 mg or 0.50 mg ketoamide was that, using fine balance to take 10.00 mg ketoamide which was then dissolved in 5.0 mL DCM (10.0 mL for 0.50 mg preparation), then adding 0.5 mL of such solvent to the 1.5 mL glass reaction vials, finally removing the DCM by rotary evaporator. And the catalyst was prepared in the corresponding solvent to load into the reaction.)

**Table S-3:** Screening of DSIs from catalyst pool of AK List

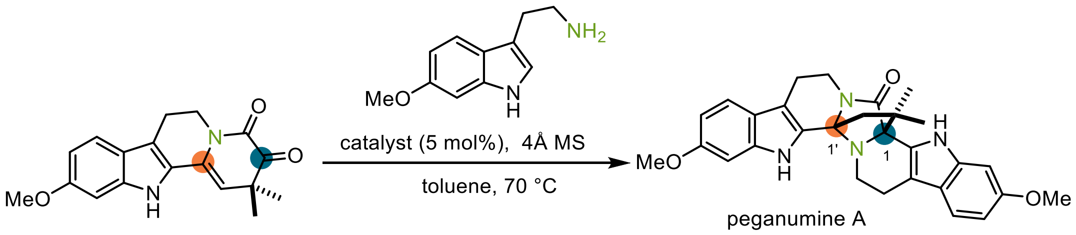


| entry | catalyst | *ee* (%) | yield (%) |
|---|---|---|---|
| 1 | **DSI-3** | 20.6 | 96 |
| 2 | **DSI-4** | 37.8 | 14 |
| 3 | **DSI-5** | 22 | 21 |
| 4 | **DSI-6** | 2.2 | 20 |
| 5 | **DSI-7** | 26.6 | 17 |
| 6 | **DSI-8** | 4.2 | 60 |
| 7 | **DSI-9** | 14 | 33 |
| 8 | **DSI-10** | 63 | 30 |
| 9 | **DSI-11** | 73 | 24 |
| 10 | **DSI-12** | 8.2 | 33 |
| 11 | **DSI-13** | 63.4 | 17 |
| 12 | **DSI-14** | 2.6 | 29 |
| 13 | **DSI-15** | 66.4 | 40 |
| 14 | **DSI-16** | 16.8 | 37 |
| 15 | **DSI-17** | 4.2 | 25 |
| 16 | **DSI-18** | 39.4 | 41 |
| 17 | **DSI-19** | 20.6 | 10 |
| 18 | **DSI-20** | 2.6 | 29 |
| 19 | **DSI-21** | 14.2 | 44 |
| 20 | **DSI-22** | 75.6 | 28 |
| 21 | **DSI-23** | 61.2 | 21 |
| 22 | **DSI-24** | 7.0 | 17 |
| 23 | **DSI-25** | 21.8 | 9 |
| 24 | **DSI-26** | 33.8 | 31 |
| 25 | **DSI-27** | 8.6 | 50 |

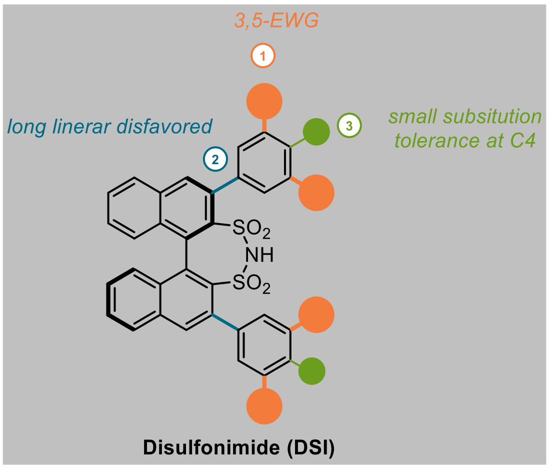


**Figure S-2:** Proposed SAR of DSIs

**Table S-4:** Screening for the newly designed DSIs

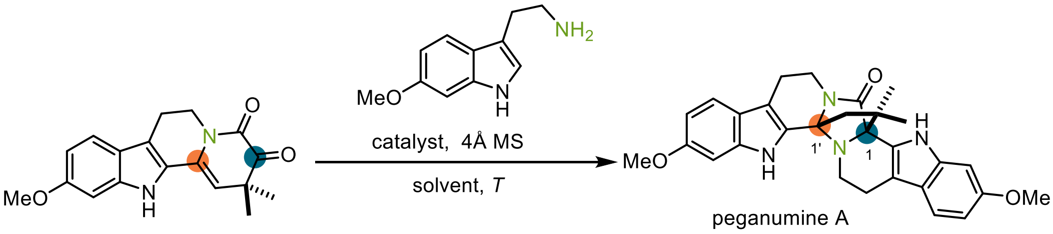


| entry | catalyst (mol%) | solvent | *T* (°C) | *ee* (%) | yield (%) |
|---|---|---|---|---|---|
| 1 | **DSI-28** (5) | toluene | 60 | 41.0 | 57 |
| 2 | **DSI-28** (5) | fluorobenzene | 60 | 32.2 | 26 |
| 3 | **DSI-28** (5) | *p*-xylene | 60 | 7.7 | 42. |
| 4 | **DSI-28** (5) | toluene | 70 | 38.6 | 35 |
| 5 | **DSI-28** (10) | toluene | 60 | 83.8 | 48 |
| 6 | **DSI-29** (5) | toluene | 60 | 47.4 | 20 |
| 7 | **DSI-29** (5) | fluorobenzene | 60 | 48.2 | 30 |
| 8 | **DSI-29** (5) | *p*-xylene | 60 | 52.0 | 27 |
| 9 | **DSI-29** (5) | toluene | 70 | 80.8 | 23 |
| 10 | **DSI-29** (10) | toluene | 60 | 83.8 | 58 |
| 11 | **DSI-30** (5) | toluene | 60 | 10.8 | 48 |
| 12 | **DSI-30** (5) | toluene | 70 | 4.4 | 44 |
| 13 | **DSI-30** (10) | toluene | 60 | 48.2 | 39 |

**Table S-5:** Optimization with DSI-2

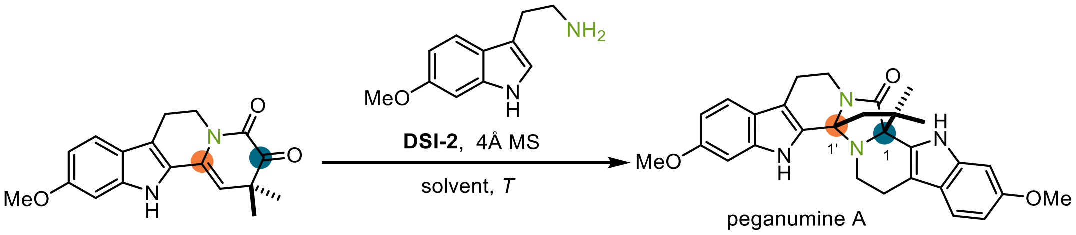


| entry | loading (mol%) | solvent | *T* (°C) | *ee* (%) | yield (%) |
|---|---|---|---|---|---|
| 1 | 5 | toluene | 50 | N.D. | N.D. |
| 2 | 10 | toluene | 50 | 87.4 | 39 |
| 3 | 5 | *p*-xylene | 50 | 89.4 | 26. |
| 4 | 5 | *m*-xylene | 50 | 75.2 | 6 |
| 5 | 5 | benzene | 50 | N.D. | N.D. |
| 6 | 5 | toluene | 60 | 83.0 | 66 |
| 7 | 10 | toluene | 60 | 92.4 | 62 |
| 8 | 5 | *p*-xylene | 60 | 91.0 | 62 |
| 9 | 5 | *m*-xylene | 60 | 90.2 | 47 |
| 10 | 5 | benzene | 60 | 67.8 | 15 |
| 11 | 5 | toluene | 70 | 83.0 | 66 |
| 12 | 10 | toluene | 70 | 93.6 | 34 |
| 13 | 5 | *p*-xylene | 70 | 91.0 | 62 |
| 14 | 5 | *m*-xylene | 70 | 90.2 | 47 |
| 15 | 5 | benzene | 70 | 67.8 | 15 |

## Procedure for the scale-up reaction:

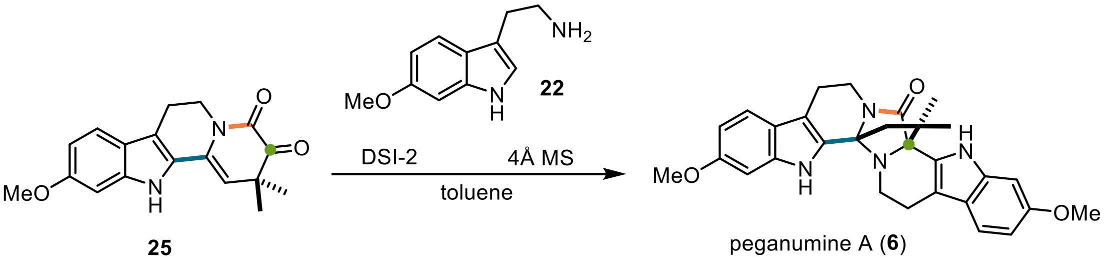


A mixture of ketoamide **25** (66.0 mg, 0.213 mmol, 1.0 equiv.), 6-methoxytryptamine (48.5 mg, 0.255 mmol, 1.2 equiv.) and 4 Å molecular sieves (50.0 mg) in toluene (50 mL) was stirred at room temperature. A solution of **DSI-2** (16.1 mg, 21.3 µmol, 0.1 equiv.) in toluene (5.0 mL) was added to the reaction mixture and the reaction mixture was stirred at 60 °C for 60 h. After the reaction mixture was cooled to room temperature, filtered through Celite®, washed by $NaHCO_3$ (sat. aq.), dried over $MgSO_4$ and concentrated under reduced pressure. The crude product was purified by silica gel column chromatography (pentane/EtOAc = 3:2) afforded **6** (83.3 mg, 0.173 mmol, 81%) as a white solid.

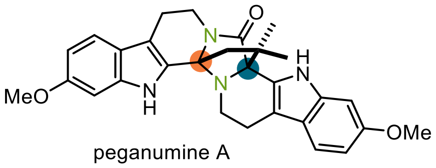

peganumine A

**$^{1}$H NMR** (700 MHz, **$CD_3OD$**) δ [ppm] = 7.38 (d, *J* = 8.7 Hz, 1H), 7.26 (d, *J* = 8.6 Hz, 1H), 6.99 (d, *J* = 2.2 Hz, 1H), 6.93 (d, *J* = 2.2 Hz, 1H), 6.73 (dd, *J* = 8.7, 2.2 Hz, 1H), 6.67 (dd, *J* = 8.6, 2.3 Hz, 1H), 4.14 (dd, *J* = 13.0, 5.8 Hz, 1H), 3.83 (s, 3H), 3.82 (s, 3H), 3.21–3.16 (m, 1H), 2.98 (dd, *J* = 15.4, 4.4 Hz, 1H), 2.90–2.76 (m, 2H), 2.69 (dd, *J* = 15.5, 3.5 Hz, 1H), 2.59 (dd, *J* = 10.8, 5.3 Hz, 1H), 2.48–2.41 (m, 1H), 2.39 (d, *J* = 11.5 Hz, 1H), 1.94 (d, *J* = 11.5 Hz, 1H), 1.44 (s, 3H), 1.24 (s, 3H).

**$^{13}$C NMR** (176 MHz, **$CD_3OD$**) δ [ppm] = 175.2, 158.3, 157.6, 139.5, 139.4, 127.8, 126.4, 122.3, 122.2, 119.9, 119.2, 112.9, 112.2, 110.5, 109.8, 96.0, 95.7, 81.2, 79.7, 56.0, 55.9, 51.9, 41.7, 41.6, 37.3, 27.3, 26.3, 22.5, 22.1.

**$^{1}$H NMR** (500 MHz, ***$d_6$*-DMSO**) δ [ppm] = 11.24 (s, 1H), 10.77 (s, 1H), 7.38 (d, *J* = 8.6 Hz, 1H), 7.25 (d, *J* = 8.6 Hz, 1H), 6.93 (d, *J* = 2.3 Hz, 1H), 6.88 (d, *J* = 2.3 Hz, 1H), 6.70 (dd, *J* = 8.6, 2.3 Hz, 1H), 6.63 (dd, *J* = 8.6, 2.3 Hz, 1H), 4.00 (dd, *J* = 12.9, 5.8 Hz, 1H), 3.78 (s, 3H), 3.77 (s, 3H), 3.10 (td, *J* = 12.6, 4.5 Hz, 1H), 2.90 (dd, *J* = 15.3, 4.2 Hz, 1H), 2.76–2.69 (m, 1H), 2.69–2.65 (m, 1H), 2.64–2.60 (m, 1H), 2.48–2.43 (m, 1H), 2.34 (dd, *J* = 10.8, 4.9 Hz, 1H), 2.30 (d, *J* = 11.3 Hz, 1H), 1.88 (d, *J* = 11.2 Hz, 1H), 1.38 (s, 3H), 1.15 (s, 3H).

**$^{13}$C NMR** (126 MHz, *$d_6$*-DMSO) δ [ppm] = 171.3, 156.1, 155.4, 137.6, 137.5, 127.3, 125.7, 120.5, 120.4, 119.0, 118.2, 111.2, 109.5, 109.0, 108.3, 94.9, 94.7, 78.8, 77.4, 55.23, 55.18, 50.4, 40.1, 39.9, 35.6, 26.8, 26.0, 21.0, 20.9.

**HRMS-ESI**: calcd. for $C_{29}H_{31}N_4O_3$ $[M + H]^+$: 483.2391; found: 483.2384.

**FT-IR**: *v* [cm$^{-1}$] = 2954, 2923, 2857, 1736, 1457, 1366, 1217, 820.

**HPLC:** 40% *i*PrOH/Heptane, IA, 1.0 mL/min

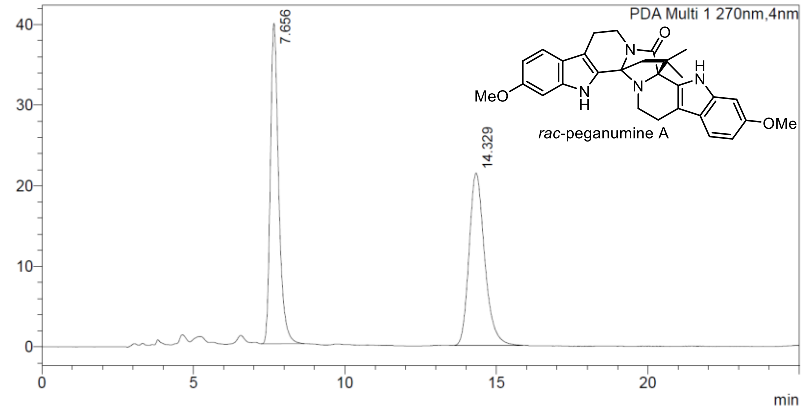


**<Peak Table>**

PDA Ch1 270nm

| Peak# | Ret. Time | Area | Area% | Height | Name |
|---|---|---|---|---|---|
| 1 | 7.656 | 759375 | 49.795 | 39781 | |
| 2 | 14.329 | 765637 | 50.205 | 21402 | |
| Total | | 1525012 | 100.000 | 61184 | |

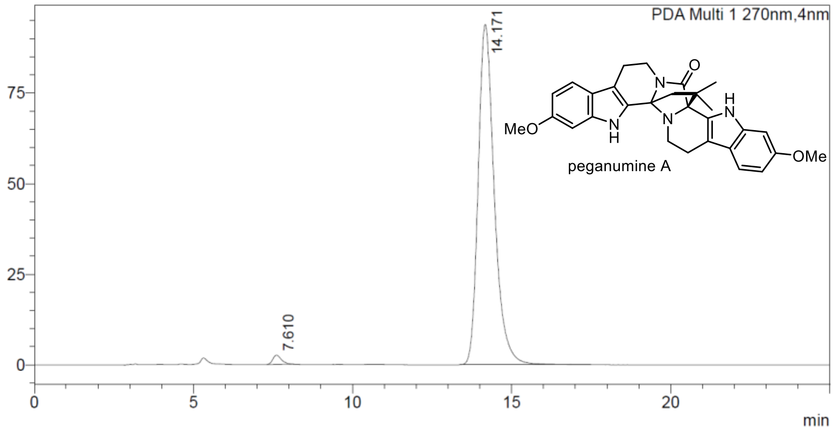


**<Peak Table>**

PDA Ch1 270nm

| Peak# | Ret. Time | Area | Area% | Height | Name |
|---|---|---|---|---|---|
| 1 | 7.610 | 48338 | 1.441 | 2562 | |
| 2 | 14.171 | 3305614 | 98.559 | 93896 | |
| Total | | 3353952 | 100.000 | 96458 | |

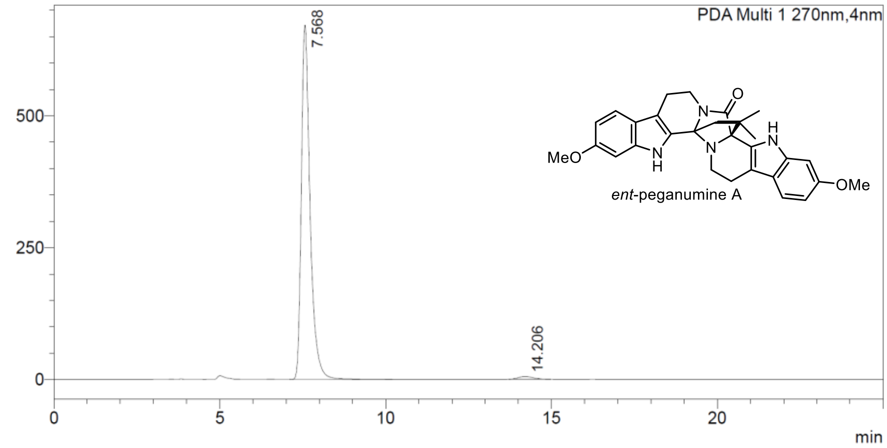


**<Peak Table>**

PDA Ch1 270nm

| Peak# | Ret. Time | Area | Area% | Height | Name |
|---|---|---|---|---|---|
| 1 | 7.568 | 12333154 | 98.548 | 671511 | |
| 2 | 14.206 | 181749 | 1.452 | 5539 | |
| Total | | 12514902 | 100.000 | 677050 | |

2.5.3.4. NMR comparison between Hua's isolation and our synthesis

| $^{1}$H-NMR (ppm) in $d_6$-DMSO | | | $^{13}$C-NMR (ppm) in $d_6$-DMSO | | |
|---|---|---|---|---|---|
| Hua's isolation (600 MHz) | Ours (500 MHz) | △δ | Hua's isolation (150 MHz) | Ours (125 MHz) | △δ |
| **11.25** (br.s, 1H) | **11.24** (br.s, 1H) | –0.01 | **171.4** | **171.3** | –0.1 |
| **10.77** (br.s, 1H) | **10.77** (br.s, 1H) | 0 | **156.1** | **156.1** | 0 |
| **7.38** (d, $J$ = 8.6 Hz, 1H) | **7.38** (d, $J$ = 8.6 Hz, 1H) | 0 | **155.4** | **155.4** | 0 |
| **7.24** (d, $J$ = 8.6 Hz, 1H) | **7.25** (d, $J$ = 8.6 Hz, 1H) | 0.01 | **137.6** | **137.6** | 0 |
| **6.93** (d, $J$ = 1.6 Hz, 1H) | **6.93** (d, $J$ = 2.3 Hz, 1H) | 0 | **137.5** | **137.5** | 0 |
| **6.87** (d, $J$ = 1.8 Hz, 1H) | **6.88** (d, $J$ = 2.3 Hz, 1H) | 0.01 | **127.3** | **127.3** | 0 |
| **6.70** (dd, $J$ = 8.6, 1.8 Hz, 1H) | **6.70** (dd, $J$ = 8.6, 2.3 Hz, 1H) | 0 | **125.7** | **125.7** | 0 |
| **6.63** (dd, $J$ = 8.6, 1.6 Hz, 1H) | **6.63** (dd, $J$ = 8.6, 2.3 Hz, 1H) | 0 | **120.5** | **120.5** | 0 |
| **4.00** (dd, $J$ = 12.6, 5.7 Hz 1H) | **4.00** (dd, $J$ = 12.9, 5.8 Hz 1H) | 0 | **120.4** | **120.4** | 0 |
| **3.78** (s, 3H) | **3.78** (s, 3H) | 0 | **119.0** | **119.0** | 0 |
| **3.77** (s, 3H) | **3.77** (s, 3H) | 0 | **118.2** | **118.2** | 0 |
| **3.09** (td, $J$ = 12.6, 4.4 Hz, 1H) | **3.10** (td, $J$ = 12.6, 4.5 Hz, 1H) | 0.01 | **111.3** | **111.2** | –0.1 |
| **2.90** (dd, $J$ = 15.1, 4.4 Hz, 1H) | **2.90** (dd, $J$ = 15.3, 4.2 Hz, 1H) | 0 | **109.5** | **109.5** | 0 |
| **2.70** (ddd, $J$ = 15.1, 12.6, 5.7 Hz, 1H) | **2.76–2.69** (m, 1H) | | **109.0** | **109.0** | 0 |

| | | | | | |
|---|---|---|---|---|---|
| **2.64** (dd, *J* = 11.0, 4.9 Hz, 1H) | **2.69–2.65** (m, 1H) | | **108.3** | **108.3** | 0 |
| **2.63** (dd, *J* = 11.0, 4.9 Hz, 1H) | **2.64–2.60** (m, 1H) | | **94.9** | **94.9** | 0 |
| **2.45** (dd, *J* = 10.9, 4.9 Hz, 1H) | **2.48–2.43** (m, 1H) | | **94.7** | **94.7** | 0 |
| **2.34** (dd, *J* = 10.9, 4.9 Hz, 1H) | **2.34** (dd, *J* = 10.8, 4.9 Hz, 1H) | 0 | **78.8** | **78.8** | 0 |
| **2.30** (d, *J* = 10.9 Hz, 1H) | **2.30** (d, *J* = 11.3 Hz, 1H) | 0 | **77.4** | **77.4** | 0 |
| **1.88** (d, *J* = 10.9 Hz, 1H) | **1.88** (d, *J* = 11.2 Hz, 1H) | 0 | **55.2 (55.23)** | **55.2 (55.23)** | 0 |
| **1.38** (s, 3H) | **1.38** (s, 3H) | 0 | **55.2 (55.18)** | **55.2 (55.18)** | 0 |
| **1.15** (s, 3H) | **1.15** (s, 3H) | 0 | **50.4** | **50.4** | 0 |
| | | | **40.0** | **40.1** | 0.1 |
| | | | **40.0** | **39.9** | –0.1 |
| | | | **35.6** | **35.6** | 0 |
| | | | **26.8** | **26.8** | 0 |
| | | | **26.0** | **26.0** | 0 |
| | | | **21.0** | **21.0** | 0 |
| | | | **20.9** | **20.9** | 0 |

2.5.3.5. NMR comparison between Zhu's synthesis and our synthesis

| $^{1}$H-NMR (ppm) in $CD_3OD$ | | | $^{13}$C-NMR (ppm) in $CD_3OD$ | | |
|---|---|---|---|---|---|
| Zhu's (400 MHz) | Ours (500 MHz) | △δ | Zhu's (101 MHz) | Ours (176 MHz) | △δ |
| **7.38** (d, *J* = 8.6 Hz, 1H) | **7.38** (d, *J* = 8.6 Hz, 1H) | 0 | **175.2** | **175.2** | 0 |
| **7.26** (d, *J* = 8.5 Hz, 1H) | **7.26** (d, *J* = 8.6 Hz, 1H) | 0 | **158.4** | **158.3** | –0.1 |
| **6.99** (d, *J* = 2.3 Hz, 1H) | **6.99** (d, *J* = 2.2 Hz, 1H) | 0 | **157.6** | **157.6** | 0 |
| **6.93** (d, *J* = 2.2 Hz, 1H) | **6.93** (d, *J* = 2.2 Hz, 1H) | 0 | **139.5** | **139.5** | 0 |
| **6.73** (dd, *J* = 8.6, 2.3 Hz, 1H) | **6.73** (dd, *J* = 8.6, 2.2 Hz, 1H) | 0 | **139.4** | **139.4** | 0 |
| **6.67** (dd, *J* = 8.6, 2.3 Hz, 1H) | **6.67** (dd, *J* = 8.5, 2.3 Hz, 1H) | 0 | **127.8** | **127.8** | 0 |
| **4.14** (dd, *J* = 13.0, 5.8 Hz, 1H) | **4.14** (dd, *J* = 13.0, 5.8 Hz, 1H) | 0 | **126.5** | **126.4** | –0.1 |
| **3.83** (s, 3H) | **3.83** (s, 3H) | 0 | **122.3** | **122.3** | 0 |
| **3.82** (s, 3H) | **3.82** (s, 3H) | 0 | **122.2** | **122.2** | 0 |
| **3.18** (td, *J* = 12.3, 4.7 Hz, 1H) | **3.21–3.16** (m, 1H) | | **120.0** | **119.9** | –0.1 |
| **2.98** (dd, *J* = 15.4, 4.5 Hz, 1H) | **2.98** (dd, *J* = 15.4, 4.3 Hz, 1H) | 0 | **119.2** | **119.2** | 0 |
| **2.90–2.74** (m, 2H) | **2.90–2.76** (m, 2H) | | **112.9** | **112.9** | 0 |
| **2.69** (dd, *J* = 15.2, 3.8 Hz, 1H) | **2.69** (dd, *J* = 15.5, 3.5 Hz 1H) | 0 | **112.2** | **112.2** | 0 |
| **2.59** (dd, *J* = 10.8, 5.2 Hz, 1H) | **2.59** (dd, *J* = 10.8, 5.3 Hz, 1H) | 0 | **110.5** | **110.5** | 0 |

| | | | | | |
|---|---|---|---|---|---|
| **2.44** (dd, *J* = 11.2, 4.1 Hz, 1H) | **2.48–2.41** (m, 1H) | | **109.8** | **109.8** | 0 |
| **2.39** (d, *J* = 11.6 Hz, 1H) | **2.39** (d, *J* = 11.5 Hz, 1H) | 0 | **96.0** | **96.0** | 0 |
| **1.94** (d, *J* = 11.5 Hz, 1H) | **1.94** (d, *J* = 11.5 Hz, 1H) | 0 | **95.7** | **95.7** | 0 |
| **1.44** (s, 3H) | **1.44** (s, 3H) | 0 | **81.3** | **81.2** | –0.1 |
| **1.24** (s, 3H) | **1.24** (s, 3H) | 0 | **79.7** | **79.7** | 0 |
| | | | **56.0** | **56.0** | 0 |
| | | | **56.0** | **55.9** | –0.1 |
| | | | **51.9** | **51.9** | 0 |
| | | | **41.7** | **41.7** | 0 |
| | | | **41.6** | **41.6** | 0 |
| | | | **37.3** | **37.3** | 0 |
| | | | **27.3** | **27.3** | 0 |
| | | | **26.3** | **26.3** | 0 |
| | | | **22.5** | **22.5** | 0 |
| | | | **22.1** | **22.1** | 0 |

2.5.3.6. Proposed mechanism and stereochemical model of the cascade

We propose the cascade reaction could divided into two phases, the Pictet–Spengler reaction phase and the acyl-iminium ion cyclization phase. Since the attack of the acyl-iminium ion is not possible to undergo crossing the ring, the stereocenter is determined by the Pictet–Spengler reaction. The ion-pair complex of the DSI and the substrate is stabilized by hydrogen-bonding and π–π stacking. The hydrogen-bonding between the free indole N–H and DSI, in our opinion, is a crucial factor.

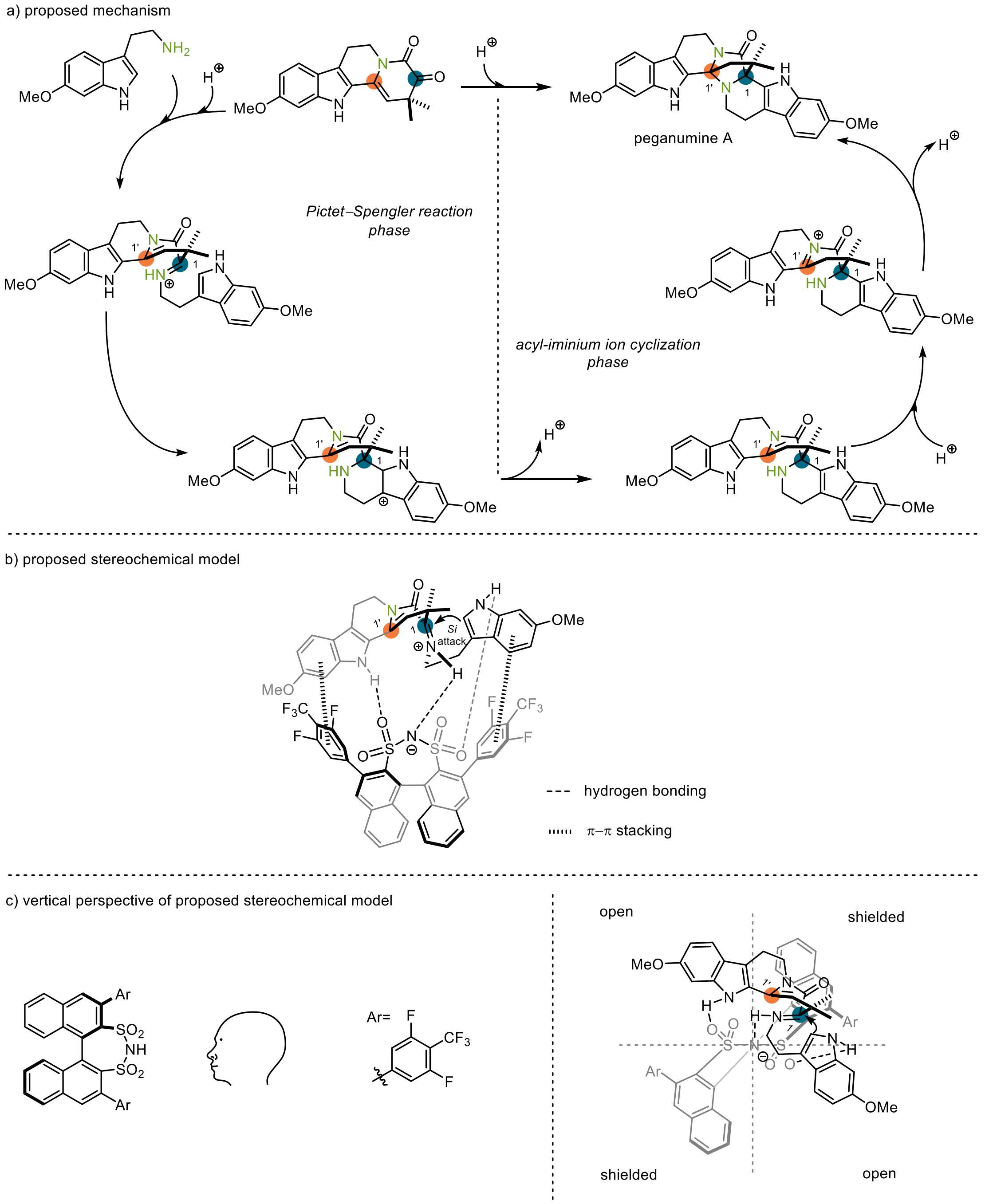


**Scheme S–6:** Proposed mechanism and stereochemical model.

## 2.6. Total synthesis of ilicifoline B

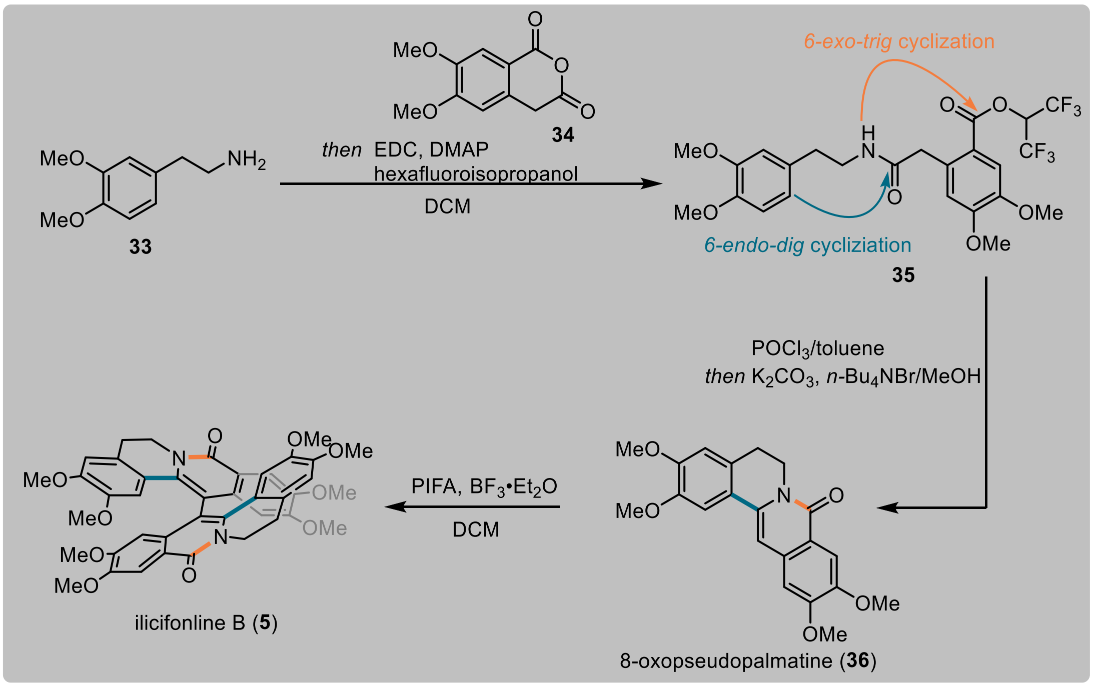


**1,1,1,3,3,3-Hexafluoropropan-2-yl 2-(2-((3,4-dimethoxyphenethyl)amino)-2-oxo-ethyl)-4,5-dimethoxybenzoate (35).**

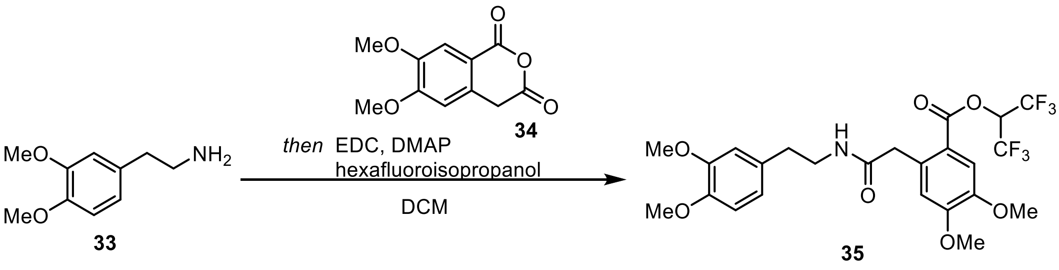


**35** was prepared according to **General procedure A**, starting from **33** (100 mg, 0.552 mmol, 1.0 equiv.) and **34** (123 mg, 0.552 mmol, 1.0 equiv.) in DCM (5.0 mL). The reaction mixture was subsequently treated with EDCI (211 mg, 1.10 mmol, 2.0 equiv.), DMAP (13.5 mg, 0.110 mmol, 0.2 equiv.) and HFIP (292 µL, 2.76 mmol, 5.0 equiv.). Purification by silica gel column chromatography (pentane/EtOAc = 3:2) afforded **35** (190 mg, 0.342 mmol, 62%) as a light yellow solid.

**$^1$H NMR** (700 MHz, $CDCl_3$) δ [ppm] = 7.46 (s, 1H), 6.91 (s, 1H), 6.71 (d, *J* = 8.0 Hz, 1H), 6.65 (d, *J* = 2.0 Hz, 1H), 6.59 (dd, *J* = 8.1, 2.0 Hz, 1H), 6.08 (s, 1H), 5.93–5.90 (m, 1H), 3.95 (s, 3H), 3.91 (s, 3H), 3.83 (s, 3H), 3.82 (s, 3H), 3.79 (s, 2H), 3.43 (td, *J* = 7.1, 5.7 Hz, 2H), 2.68 (t, *J* = 7.1 Hz, 2H).

**$^{13}$C NMR** (176 MHz, $CDCl_3$) δ [ppm] = 170.2, 163.4, 154.1, 149.1, 147.9, 147.8, 134.3, 131.3, 120.69 (q, $J$ = 283.7 Hz), 120.65, 116.9, 115.0, 113.5, 112.0, 111.2, 67.3–66.5 (m), 56.4, 56.1, 55.9, 42.0, 40.9, 35.2. (Based on the intensity of the resonance, the peak at δ [ppm] = 55.9 is considered as two methoxy carbons.)

**$^{19}$F NMR** (565 MHz, $CDCl_3$) δ [ppm] = –72.98 (d, $J$ = 6.1 Hz).

**HRMS-ESI**: calcd. for $C_{24}H_{25}F_6NO_7Na$ $[M + Na]^+$: 576.1427; found: 576.1401.

**FT-IR:** $\nu$ [cm$^{-1}$] = 2934, 1749, 1674, 1518, 1263, 1234, 1194, 1110, 768.

**2,3,10,11-Tetramethoxy-5,6-dihydro-8*H*-isoquinolino[3,2-*a*]isoquinolin-8-one (36)**.

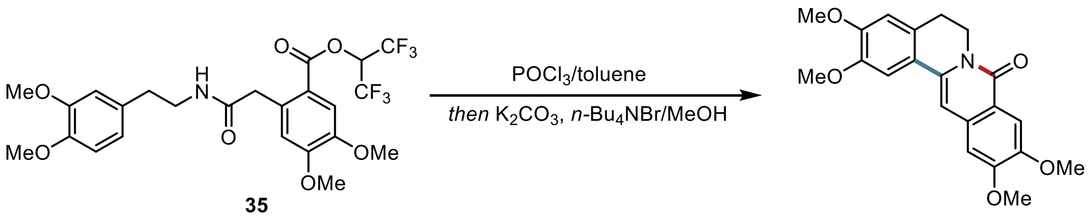

8-oxopseudopalmatine (**36**)

**36** was prepared according to **General procedure C**, starting from **35** (60 mg, 0.11 mmol, 1.0 equiv.) and $POCl_3$ (9.9 µL, 0.11 mmol, 1.0 equiv.) in toluene (3.0 mL). The reaction mixture was subsequently treated with MeOH (3.0 mL), $K_2CO_3$ (150 mg, 1.1 mmol, 10 equiv.) and *n*$Bu_4NBr$ (3.5 mg, 0.022 mmol, 0.1 equiv.). Purification by silica gel column chromatography (pentane/EtOAc = 1:1) afforded **36** (38 mg, 0.104 mmol, 95%) as a yellow solid.

**$^1$H NMR** (700 MHz, $CDCl_3$) δ [ppm] = 7.81 (s, 1H), 7.25 (s, 1H), 6.94 (s, 1H), 6.83 (s, 1H), 6.74 (s, 1H), 4.39–4.34 (m, 2H), 4.02 (s, 3H), 4.01 (s, 3H), 3.98 (s, 3H), 3.94 (s, 3H), 2.96–2.92 (m, 2H).

**$^{13}$C NMR** (176 MHz, $CDCl_3$) δ [ppm] = 161.6, 153.7, 150.3, 149.2, 148.6, 136.3, 132.3, 128.6, 122.7, 118.7, 110.7, 108.0, 107.8, 106.1, 101.3, 56.4, 56.4, 56.2, 39.9, 28.3. (Based on the intensity of the resonance, the peak at δ [ppm] = 56.2 is considered as two methoxy carbons.)

**HRMS-ESI:** calcd. for $C_{21}H_{21}NO_5Na$ $[M + Na]^+$: 390.1312 found: 390.1323.

**FT-IR:** $\nu$ [cm$^{-1}$] = 2929, 1644, 1594, 1509, 1466, 1426, 1255, 1235, 1097, 1027, 747.

**2,2',3,3',10,10',11,11'-Octamethoxy-5,5',6,6'-tetrahydro-8*H*,8'*H*-[13,13'-biisoquinolino[3,2-*a*]isoquinoline]-8,8'-dione (5)**.

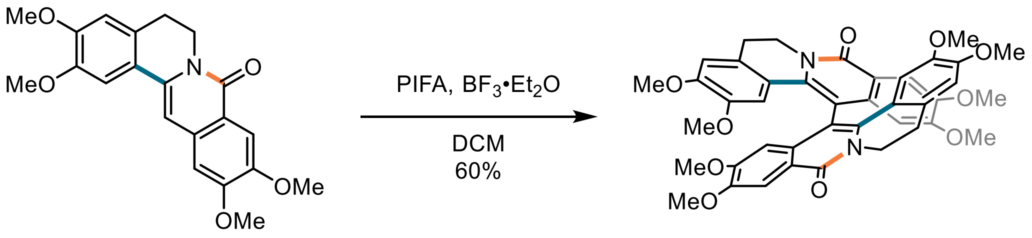


8-oxopseudopalmatine (**36**)　　　ilicifoline B (**5**)

A mixture of 8-oxypseudopalmatine (18 mg, 50 µmol, 1.0 equiv.) and PIFA (16 mg, 0.05 mmol, 1.0 equiv.) in DCM (2.0 mL), was added $BF_3$•$Et_2O$ (9.7 µL, 0.10 mmol, 2.0 equiv.) at –78 °C. The mixture was stirred for 3 h, then quenched with $NaHCO_3$ (sat. aq.) and diluted with DCM, and the organic phase was separated. The aqueous phase was extracted with DCM and the combined organic layers were washed with brine, dried over $MgSO_4$, filtered, and concentrated under reduced pressure. Purification by silica gel column chromatography (pentane/EtOAc = 1:1 to EtOAc) afforded **5** (11 mg, 15 µmol, 60%) as a yellow solid.

**$^1$H NMR** (700 MHz, $CDCl_3$) δ [ppm] = 7.95 (s, 2H), 6.76 (s, 2H), 6.43 (s, 2H), 6.34 (s, 2H), 5.05 (dt, $J$ = 13.2, 3.8 Hz, 2H), 4.06 (s, 6H), 3.82 (s, 6H), 3.62 (s, 6H), 3.33 (td, $J$ = 13.2, 3.0 Hz, 2H), 3.03 (s, 6H), 2.52 (dt, $J$ = 15.1, 3.1 Hz, 2H), 2.13 (ddd, $J$ = 14.5, 12.7, 4.2 Hz, 2H).

**$^{13}$C NMR** (176 MHz, $CDCl_3$) δ [ppm] = 161.0, 153.9, 149.5, 149.2, 146.6, 137.0, 133.6, 131.3, 122.5, 119.0, 111.6, 110.8, 109.2, 108.1, 105.8, 56.4, 56.3, 56.2, 55.2, 41.4, 29.0.

**HRMS-EI:** calcd. for $C_{42}H_{40}N_2O_{10}Na$ $[M + Na]^+$: 755.2575; found: 755.2576.

**FT-IR:** $\nu$ [cm$^{-1}$] = 3004, 2928, 1633, 1607, 1587, 1498, 1464, 1440, 1374, 1271, 1226, 1133, 1096, 1008, 876, 749.

NMR comparison between Opatz's synthesis and our synthesis

| $^1$H-NMR (ppm) in $CDCl_3$ | | | $^{13}$C-NMR (ppm) in $CDCl_3$ | | |
|---|---|---|---|---|---|
| Opatz's (600 MHz) | Ours (700 MHz) | △δ | Opatz's (150 MHz) | Ours (176 MHz) | △δ |
| **7.95** (s, 2H) | **7.95** (s, 1H) | 0 | **160.9** | **161.0** | 0.1 |
| **6.75** (s, 2H) | **6.76** (s, 2H) | 0.01 | **153.8** | **153.9** | 0.1 |
| **6.42** (s, 2H) | **6.43** (s, 2H) | 0.01 | **149.4** | **149.5** | 0.1 |
| **6.33** (s, 2H) | **6.34** (s, 2H) | 0.01 | **149.1** | **149.2** | 0.1 |
| **5.09 – 4.99** (m, 2H) | **5.05** (dt, *J* = 13.2, 3.8 Hz, 2H) | | **137.0** | **137.0** | 0 |
| **4.06** (s, 6H) | **4.06** (s, 6H) | 0 | **133.6** | **133.6** | 0 |
| **3.82** (s, 6H) | **3.82** (s, 6H) | 0 | **131.3** | **131.3** | 0 |
| **3.62** (s, 6H) | **3.62** (s, 6H) | 0 | **122.5** | **122.5** | 0 |
| **3.40 – 3.27** (m, 2H) | **3.33** (td, *J* = 13.2, 3.0 Hz, 2H) | | **119.0** | **119.0** | 0 |
| **3.02** (s, 6H) | **3.03** (s, 6H) | 0.01 | **111.5** | **111.6** | 0.1 |
| **2.57 – 2.47** (m, 2H) | **2.52** (dt, *J* = 15.1, 3.1 Hz, 2H) | | **110.8** | **110.8** | 0 |
| **2.19 – 2.07** (m, 2H) | **2.13** (ddd, *J* = 14.5, 12.7, 4.2 Hz, 2H) | | **109.1** | **109.2** | 0.1 |
| | | | **108.0** | **108.1** | 0.1 |
| | | | **105.8** | **105.8** | 0 |

| | | | | | |
|---|---|---|---|---|---|
| | | | **56.4** | **56.4** | 0 |
| | | | **56.3** | **56.3** | 0 |
| | | | **56.1** | **56.2** | 0.1 |
| | | | **55.2** | **55.2** | 0 |
| | | | **41.4** | **41.4** | 0 |
| | | | **29.0** | **29.0** | 0 |

## 2.7. Preparation of amines

### 2.7.1. Commercially available amines

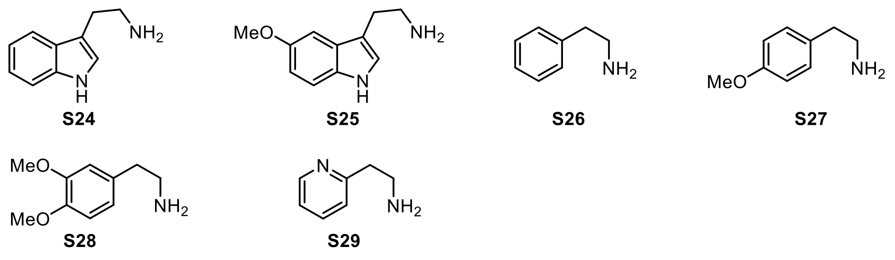


### 2.7.2. Noncommercially available amines synthesis by reported methods

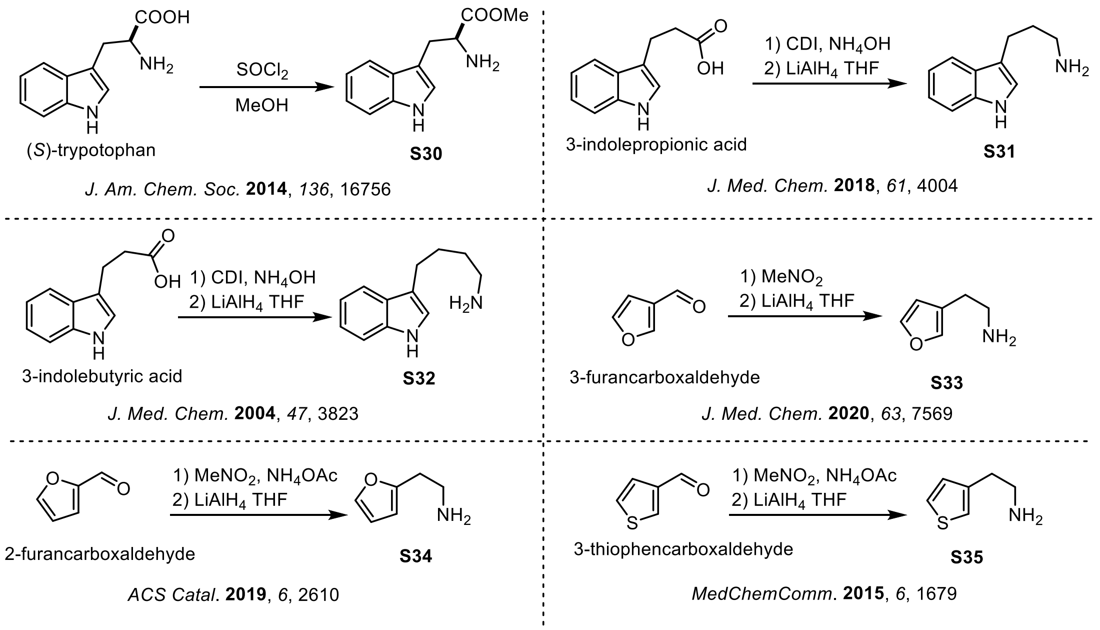


**S30** was prepared from (*S*)-trypotophan by the procedure of Carreira et al..[9] **S31** was synthesized from 3-indolepropionic acid according to the procedure of Weinstock et al..[10] **S32** was obtained from 3-indolebutyric acid following the procedure of Mewshaw et al..[11] **S33** was prepared from 3-furancarboxaldehyde via the procedure of Liotta et al..[12] **S34** was prepared from 2-furancarboxaldehyde through the procedure

[9] J.Ruchti, E. M. Carreira *J. Am. Chem. Soc.* **2014**, *136*, 16756–16759.
[10] S. Zeeli, T. Weill, E. Finkin-Groner, C. Bejar, M. Melamed, S. Furman, M. Zhenin, A. Nudelman, M. Weinstock *J. Med. Chem.* **2018**, *61*, 4004–4019.
[11] R. E. Mewshaw, D. Zhou, P. Zhou, X. Shi, G. Hornby, T. Spangler, R. Scerni, D. Smith, L. E. Schechter, T. H. Andree *J. Med. Chem.* **2004**, *47*, 3823–3842.
[12] M. P. Epplin, A. Mohan, L. D. Harris, Z. Zhu, K. L. Strong, J. Bacsa, P. Le, D. S. Menaldino, S. F. Traynelis, D.

of Huang et al..[13] **S35** was prepared from 3-thiophencarboxaldehyde using the procedure of Enzensperger et al..[14] The spectroscopic data are in agreement with literatures.

2.7.3. Self-made amines

The amines (**22** and **S36–S38**) were synthesized from the corresponding heterocycles. The preparation of the aldehydes depended on the starting material supply. For **22**, **S36** and **S37**, the aldehydes were synthesized from the corresponding indoles through a Vilsmeier–Haack reaction. For **S39**, the aldehydes were prepared via bromination and formylation. The Henry reaction with $MeNO_2$ and the reduction by $LiAlH_4$ followed the same procedure.

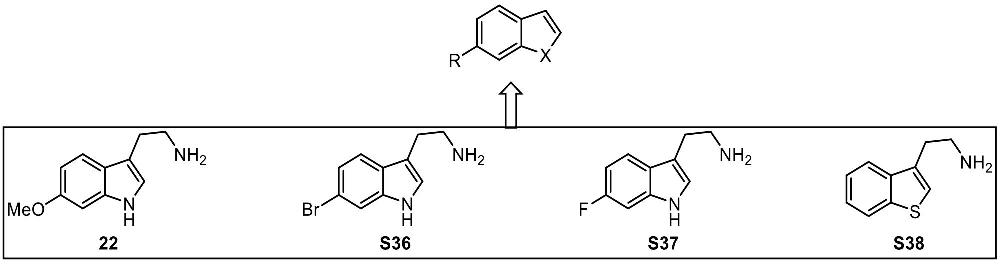


**General procedure F**

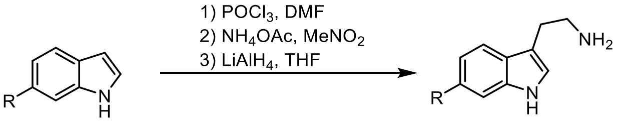


Vilsmeier–Haack reaction: To a dried Schlenk flask with DMF, was added $POCl_3$ (3.0 equiv.) dropwise at 0 °C. After the mixture was stirred for 30 min, a solution of corresponding indole (1.0 equiv.) in DMF was added to the reaction mixture dropwise. The resulting mixture was allowed to stir at room temperature for another 2 h. Then the reaction mixture was poured to a flask with ice, and carefully neutralized by $NaHCO_3$ (sat. aq.) until pH = 7–8. The suspension was left to rest overnight at room temperature,

C. Liotta *J. Med. Chem.* **2020**, *63*, 7569−7600.

[13] J. Liu, P. Chakraborty, H. Zhang, L. Zhong, Z. Wang, X. Huang *ACS Catal.* **2019**, *9*, 2610−2617.

[14] M. A. O. Abdel-Fattah, A. H. Abadi, J. Lehmann, P. M. Schweikerta, C. Enzensperger *Med. Chem. Commun.*, **2015**, *6*, 1679–1686.

filtered, collected the solid and dried under reduced pressure. This crude product was used without further purification.

<u>Henry reaction:</u> A flask with $NH_4OAc$ (3.0 equiv.) and corresponding aldehyde (1.0 equiv.) was added $MeNO_2$ (75 equiv.) and the reaction mixture was stirred at 115 °C for 2h. After allowing the mixture to cool down to room temperature, the reaction mixture was diluted with water and DCM, and the organic phase was separated. The aqueous phase was extracted with DCM. The combined organic layers were washed with brine, dried over $MgSO_4$, filtered, and concentrated under reduced pressure to afford the crude product which was used directly without purification.

<u>Reduction:</u> To a Schlenk flash charged with solid $LiAlH_4$ (6.0 equiv.) was added THF carefully at 0 °C. Then a solution of the crude product of the Henry reaction in THF was added dropwise. The resulting reaction mixture was replaced with an oil bath and heated to 65 °C for 3h, then cooled down to 0 °C. Excess $LiAlH_4$ was quenched by the drop-wise addition of NaOH (aq.) and $H_2O$. The suspension was allowed to warm to room temperature overnight and then filtered through a plug of Celite®. The organic phase was separated. The aqueous phase was extracted with DCM and the combined organic layers were washed with brine, dried over $MgSO_4$, filtered, and concentrated under reduced pressure. The crude material was purified by silica gel column chromatography.

**2-(6-Methoxy-1*H*-indol-3-yl)ethan-1-amine (22)**.

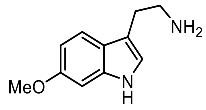

**22**

**22** was prepared according to **General procedure F**, starting from 6-methoxyindole (530 mg, 3.60 mmol, 1.0 equiv.) and $POCl_3$ (1.01 mL, 10.8 mmol, 3.0 equiv.) in DMF (3.0 mL). The reaction mixture was subsequently treated with $NH_4OAc$ (833 mg, 10.8 mmol, 3.0 equiv.), $MeNO_2$ (14.5 mL, 270 mmol, 75 equiv.) and $LiAlH_4$ (821 mg, 10.8 mmol, 6.0 equiv.) in THF (25 mL). Purification by silica gel column chromatography (DCM/MeOH/TEA = 80:20:5) afforded **22** (270 mg, 1.42 mmol, 39%)

as a light brown solid.

**$^1$H NMR** (500 MHz, $CD_3OD$) δ [ppm] = 7.37 (d, *J* = 8.6 Hz, 1H), 6.91 (s, 1H), 6.87 (d, *J* = 2.3 Hz, 1H), 6.67 (dd, *J* = 8.6, 2.3 Hz, 1H), 3.76 (s, 3H), 2.87 (t, *J* = 6.8 Hz, 2H), 2.81 (t, *J* = 6.7 Hz, 2H).

**$^{13}$C NMR** (126 MHz, $CD_3OD$) δ [ppm] = 157.6, 138.9, 123.2, 122.3, 119.9, 113.3, 109.9, 95.6, 56.0, 42.9, 29.4.

**HRMS-ESI:** calcd. for $C_{11}H_{15}N_2O$ $[M + H]^+$: 191.1179; found: 191.1179.

**FT-IR:** $\nu$ [$cm^{-1}$] = 3401, 2924, 2834, 1627, 1456, 1305, 1262, 1200, 1160, 1026, 800, 753.

**2-(6-Fluoro-1*H*-indol-3-yl)ethan-1-amine (S36)**.

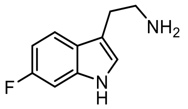

**S36**

**S36** was prepared according to **General procedure F**, starting from 6-fluoroindole (1.70 g, 12.6 mmol, 1.0 equiv.) and $POCl_3$ (3.53 mL, 37.7 mmol, 3.0 equiv.) in DMF (10.0 mL). The reaction mixture was subsequently treated with $NH_4OAc$ (2.91 g, 37.8 mmol, 3.0 equiv.), $MeNO_2$ (50.5 mL, 944 mmol, 75 equiv.) and $LiAlH_4$ (2.86 g, 75.5 mmol, 6.0 equiv.) in THF (80.0 mL). Purification by silica gel column chromatography (DCM/MeOH/TEA = 80:20:5) afforded **S36** (580 mg, 3.26 mmol, 26%) as a brown solid.

**$^1$H NMR** (500 MHz, $CD_3OD$) δ [ppm] = 7.50 (dd, *J* = 8.7, 5.3 Hz, 1H), 7.13 (s, 1H), 7.06 (dd, *J* = 9.9, 2.3 Hz, 1H), 6.86–6.77 (m, 1H), 3.11 (dd, *J* = 7.9, 6.4 Hz, 2H), 3.02 (t, *J* = 7.2 Hz, 2H).

**$^{13}$C NMR** (126 MHz, $CD_3OD$) δ [ppm] = 161.3 (d, *J* = 235.3 Hz), 138.2 (d, *J* = 12.4 Hz), 125.1, 124.5 (d, *J* = 3.3 Hz), 119.9 (d, *J* = 10.2 Hz), 111.7, 108.3 (d, *J* = 24.9 Hz), 98.3 (d, *J* = 26.0 Hz), 41.8, 26.2.

**$^{19}$F NMR** (471 MHz, $CD_3OD$) δ [ppm] = –124.28.

**HRMS-ESI:** calcd. for $C_{10}H_{12}FN_2$ $[M + H]^+$: 179.0979; found: 179.0979.

**FT-IR:** $\nu$ [$cm^{-1}$] = 2918, 2840, 1626, 1456, 1345, 1225, 1144, 1099, 951, 799.

**2-(6-Bromo-1*H*-indol-3-yl)ethan-1-amine (S37)**.

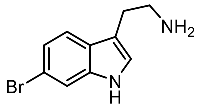


**S37**

**S37** was prepared according to **General procedure F**, starting from 6-bromoindole (1.0 g, 5.2 mmol, 1.0 equiv.) and $POCl_3$ (1.4 mL, 15 mmol, 3.0 equiv.) in DMF (5.0 mL). The reaction mixture was subsequently treated with $NH_4OAc$ (1.2 g, 15 mmol, 3.0 equiv.), $MeNO_2$ (21 mL, 386 mmol, 75 equiv.) and $LiAlH_4$ (1.2 g, 31 mmol, 6.0 equiv.) in THF (50 mL). Purification by silica gel column chromatography (DCM/MeOH/TEA = 80:20:5) afforded **S37** (580 mg, 3.26 mmol, 63%) as a light brown solid.

**$^1$H NMR** (500 MHz, $CD_3OD$) δ [ppm] = 7.50 (d, $J$ = 1.7 Hz, 1H), 7.45 (d, $J$ = 8.4 Hz, 1H), 7.10 (dd, $J$ = 8.4, 1.8 Hz, 1H), 7.08 (d, $J$ = 0.8 Hz, 1H), 2.95 – 2.90 (m, 2H), 2.90 – 2.85 (m, 2H).

**$^{13}$C NMR** (126 MHz, $CD_3OD$) δ [ppm] = 139.0, 127.7, 124.5, 122.7, 120.7, 115.8, 115.1, 113.7, 43.0, 29.1.

**HRMS-ESI:** calcd. for $C_{10}H_{12}BrN_2$ $[M + H]^+$: 239.0179; found: 239.0191.

**FT-IR:** $\nu$ [cm$^{-1}$] = 3423, 3126, 2935, 2874, 1613, 1578, 1456, 1334, 1232, 1048, 894, 802, 771.

**2-(Benzo[*b*]thiophen-3-yl)ethan-1-amine (S38)**.

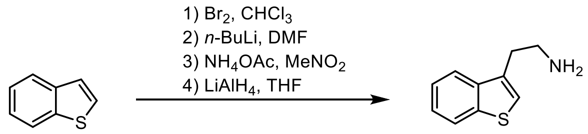


benzo[*b*]thiophene **S38**

Bromination:[15] Benzo[*b*]thiophene (2.0 g, 15 mmol, 1.0 equiv.) was dissolved in chloroform (30 mL) and cooled by an ice bath. A solution of bromine (0.84 mL, 16 mmol, 1.1 equiv.) in chloroform (10 mL) was slowly added. The reaction mixture was stirred for 1 h and quenched by NaOH (1.0 M aq.) and $Na_2S_2O_3$ (sat. aq.). The resulting mixture was extracted with DCM. After evaporation of the solvent, the intermediate

[15] S. Fredrich, A. Bonasera, V. Valderrey, S. Hecht *J. Am. Chem. Soc.* **2018**, *140*, 6432–6440

was dissolved in ethanol and was added dropwise to a solution of KOH (sat. EtOH solution, 20 mL) was added at ice bath. After refluxing for 2 h, $H_2O$ was added and the EtOH was evaporated. The residue was extracted by DCM, washed with brine, dried with $MgSO_4$, filtered and concentrated under reduced pressure to afford crude 3-bromo-benzo[*b*]thiophene as yellow oil. The crude product was used without purification.

Formylation procedure:[16] Crude 3-bromo-benzo[*b*]thiophene (3.4 g, 16 mmol, 1.0 equiv.) was dissolved in $Et_2O$ and the solution was cooled to –78 °C. After the dropwise addition *n*BuLi (2.5 M solution in hexane, 6.4 mL, 16 mmol, 1.0 equiv.), the mixture was stirred for 30 min. Following the addition of DMF (1.2 mL, 16 mmol, 1.0 equiv.) at –78 °C, the mixture was allowed to warm to room temperature for 1 h. The reaction mixture was quenched by $NH_4Cl$ (sat. aq.) and extracted with DCM. Combined organic layers were dried over $MgSO_4$, filtered and concentrated under reduced pressure to afford crude 3- benzo[*b*]thiophene-3-carbaldehyde as yellow oil..

Henry reaction: A flask with $NH_4OAc$ (2.3 g, 30 mmol, 3.0 equiv.) and crude 3-benzo[*b*]thiophene-3-carbaldehyde (1.6 g, 9.9 mmol, 1.0 equiv.) was added $MeNO_2$ (40 mL) and the reaction mixture was stirred at 115 °C for 2h. After cooled to room temperature, the reaction mixture was diluted with water and DCM, and the organic phase was separated. The aqueous phase was extracted with DCM and the combined organic layers were washed with brine, dried over $MgSO_4$, filtered and concentrated under reduced pressure to afford the crude 3-(2-nitrovinyl)benzo[*b*]thiophene which could use directly without purification.

Reduction: To a Schlenk flash charged with solid $LiAlH_4$ (2.2 g, 59 mmol, 6.0 equiv.), was added THF carefully at 0 °C. Then a solution of crude 3-(2-nitrovinyl)benzo[*b*]thiophene in THF (20 mL) was added dropwise. The resulting reaction mixture was replaced with an oil bath and heated to 65 °C for 3h, then cooled first to room temperature and then to 0 °C. Excess $LiAlH_4$ was quenched by the drop-wise addition of NaOH (sat. aq.) and $H_2O$. The suspension was allowed to warm to room temperature overnight and then filtered through a plug of Celite®. The organic

[16] W. Gong, Y. Liu, J. Zhang, Y. Jiao, J. Xue, Y. Li *Chem. Asian J.* **2013**, *8*, 546–551

phase was separated. The aqueous phase was extracted with DCM and the combined organic layers were washed with brine, dried over $MgSO_4$, filtered, and concentrated under reduced pressure. The crude material was purified by silica gel column chromatography (DCM/MeOH/TEA = 80:20:5) afforded **S38** (300 mg, 1.69 mmol, 11% over 4 steps) as a dark brown solid.

**$^1$H NMR** (500 MHz, $CD_3OD$: $CDCl_3$ (v/v = 1:1)) δ [ppm] = 7.54 (dt, *J* = 7.9, 1.1 Hz, 1H), 7.46 (dt, *J* = 8.0, 0.9 Hz, 1H), 7.07 (ddd, *J* = 8.0, 7.0, 1.3 Hz, 1H), 7.03 (ddd, *J* = 8.3, 7.3, 1.3 Hz, 1H), 6.92 (s, 1H), 2.75 (s, 4H).

**$^{13}$C NMR** (126 MHz, $CD_3OD$: $CDCl_3$ (v/v = 1:1)) δ [ppm] = 140.2, 138.2, 132.5, 123.9, 123.6, 122.4, 122.3, 121.0, 40.0, 30.1.

**HRMS-ESI:** calcd. for $C_{10}H_{12}NS$ $[M + H]^+$: 178.0685; found: 178.0693.

**FT-IR:** *v* [cm$^{-1}$] = 3057, 2924, 2864, 1585, 1458, 1427, 1020, 760, 732.

2.8. Preparation of anhydrides

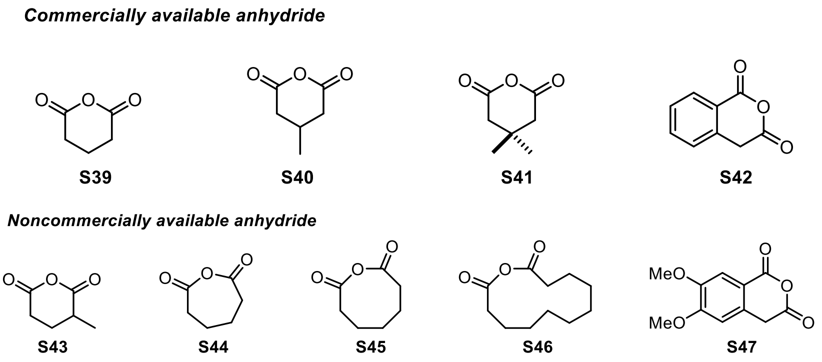


In general, the anhydrides (**S43 – S46**) were synthesized from the corresponding dicarboxylic acids.

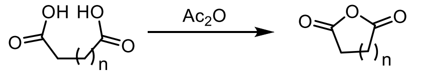


**S43** was prepared by the procedure of Wilson et al.[17] **S44** was prepared by the procedure of Köper et al.[18] **S45** was prepared by the procedure of Sun et al.[19] **S46** was prepared by the same procedure as **S45**.

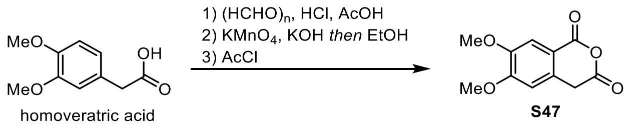


**S47** was prepared from homoveratric acid by the procedure of Cushman et al..[20]

[17] D. J. Bennetta, A. J. Blakec, P. A. Cooke, C. R. A. Godfrey, P. L. Pickering, N. S. Simpkins. M. D. Walker, C. Wilson *Tetrahedron* **2004**, *60*, 4491–4511.
[18] J. Andersson, J. J. Knobloch, M. V. Perkins, S. A. Holt, I. Köper Langmuir **2017,** *33*, 4444–4451.
[19] B. Sun, C. Luo, X. Zhang, M. Guo, M. Sun, H. Yu, Q. Chen, W. Yang, M. Wang, S. Zuo, P. Chen, Q. Kan, H. Zhang, Y. Wang, Z. He, J. Sun *Nat. Commun.* **2019**, *10*, 3211.
[20] D. E. Beck, K. Agama, C. Marchand, A. Chergui, Y. Pommier, M. Cushman *J. Med. Chem.* **2014**, *57*, 1495–1512.

## 3. NMR Spectra

**$^{1}$H NMR** (500 MHz, $CDCl_3$) of **8a.**

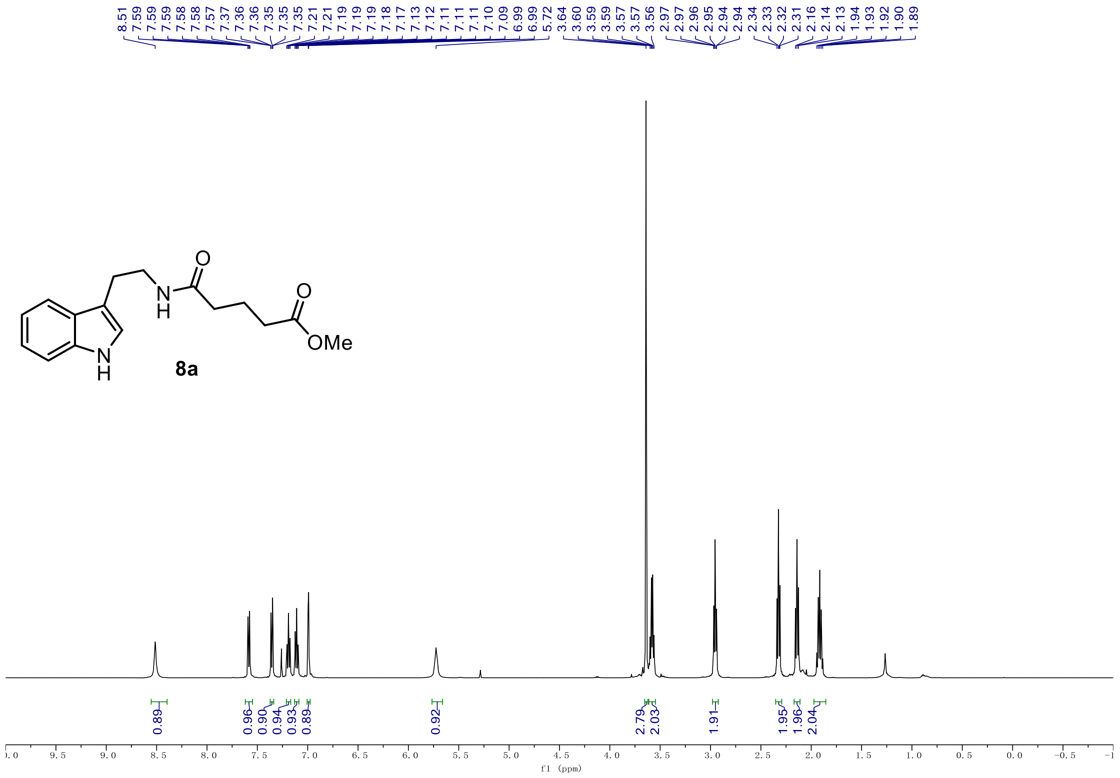


**$^{13}$C NMR** (126 MHz, $CDCl_3$) of **8a.**

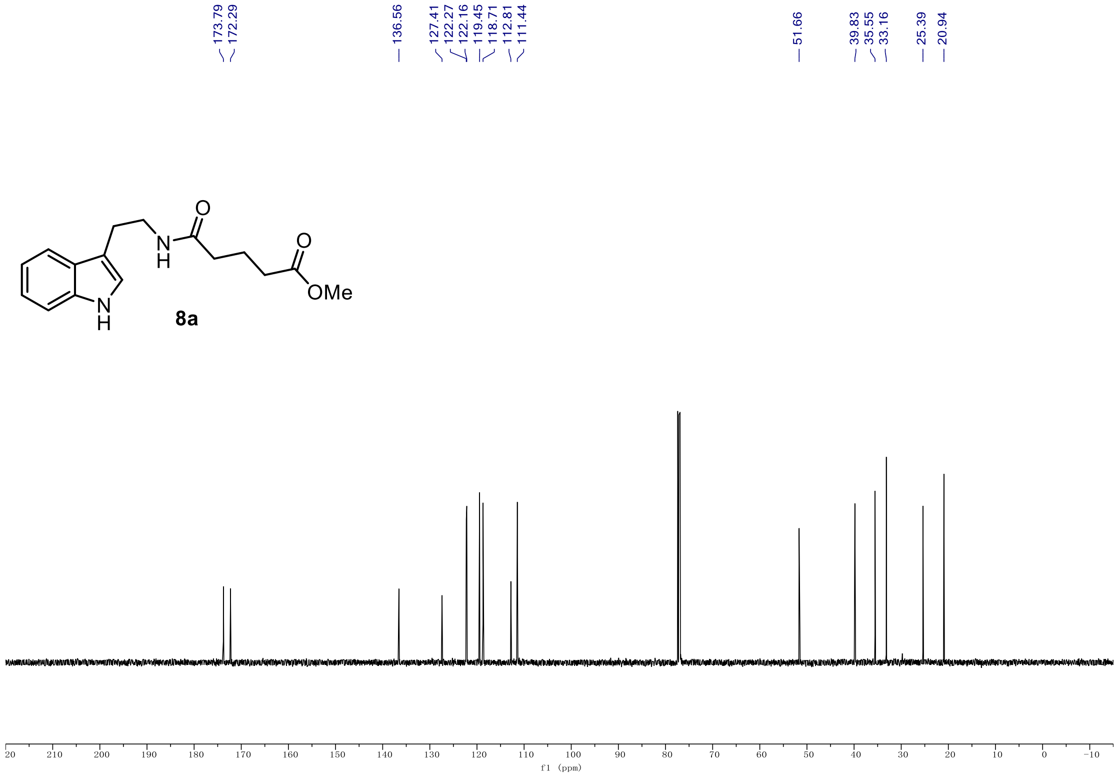

**$^{1}$H NMR** (500 MHz, $CD_3OD$: $CDCl_3$ (v/v = 1:1)) of **8b.**

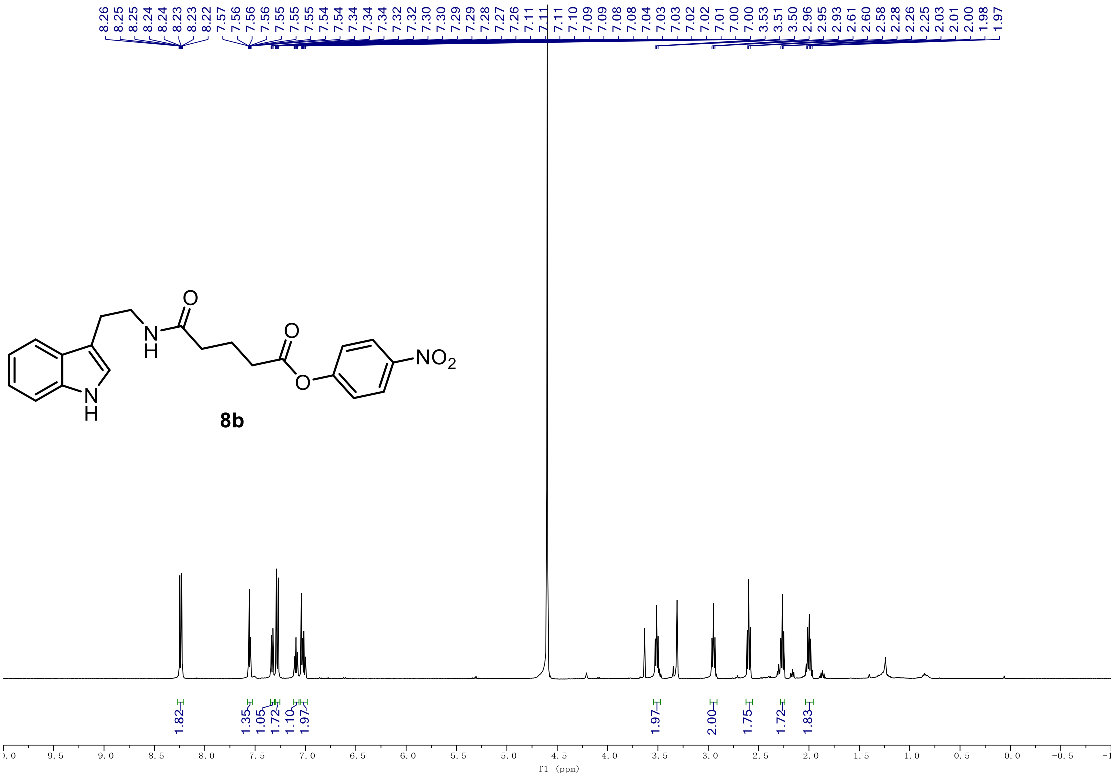


**$^{13}$C NMR** (176 MHz, $CD_3OD$: $CDCl_3$ (v/v = 1:1)) **8b.**

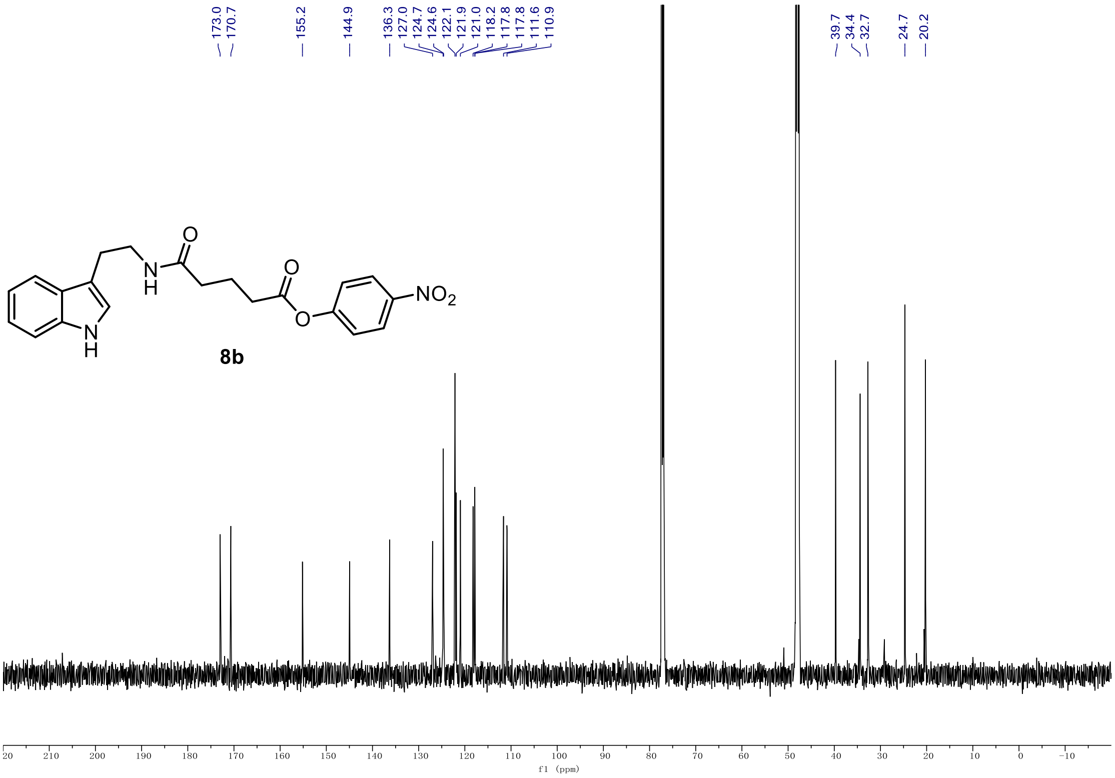

**$^{1}$H NMR** (500 MHz, $CDCl_3$) of **8c.**

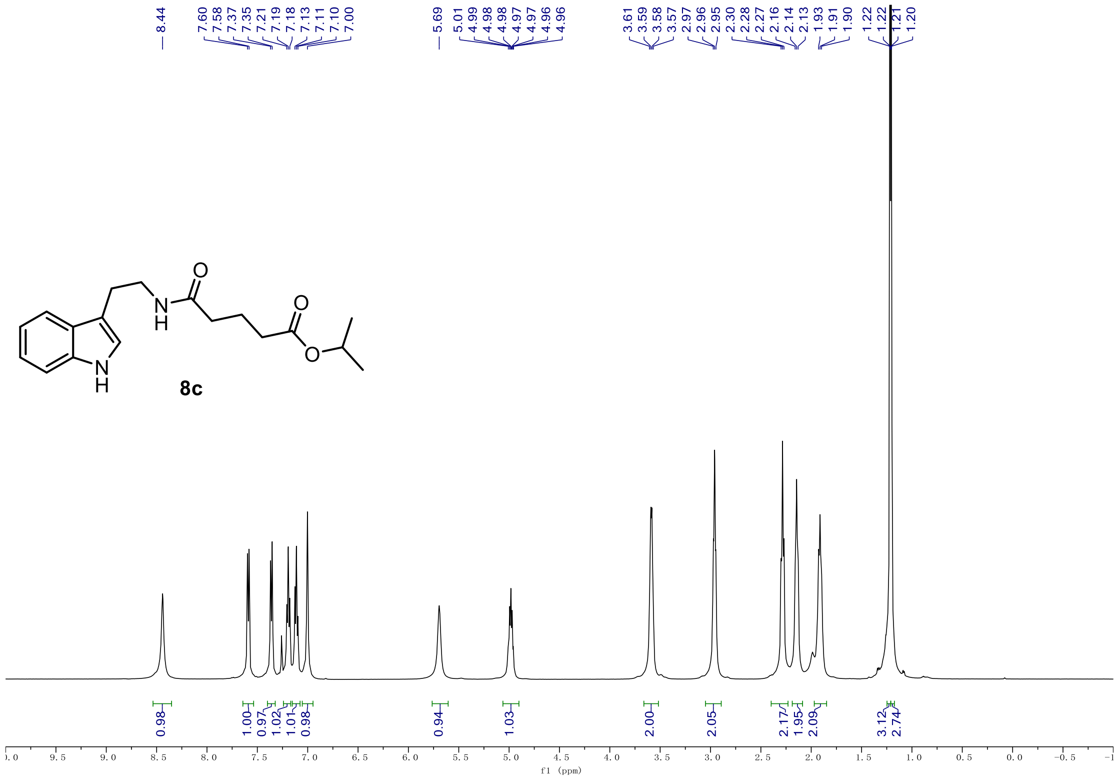


**$^{13}$C NMR** (126 MHz, $CDCl_3$) **8c.**

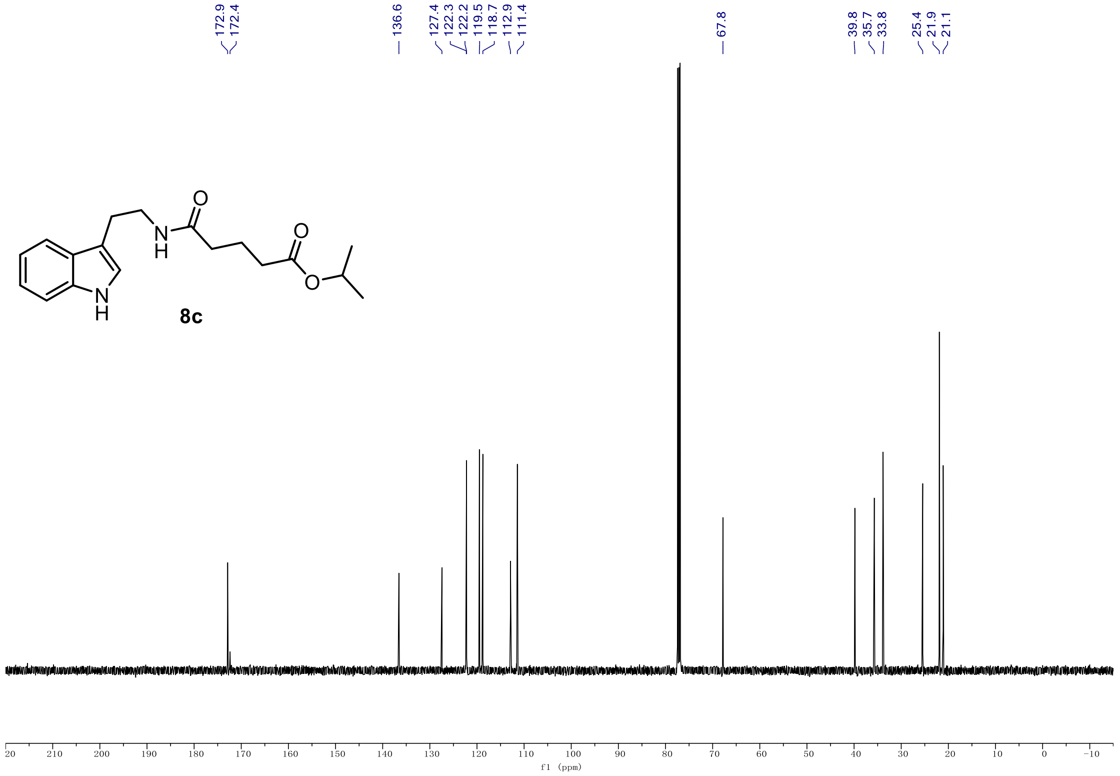

**$^{1}$H NMR** (600 MHz, $CDCl_3$) **8d**.

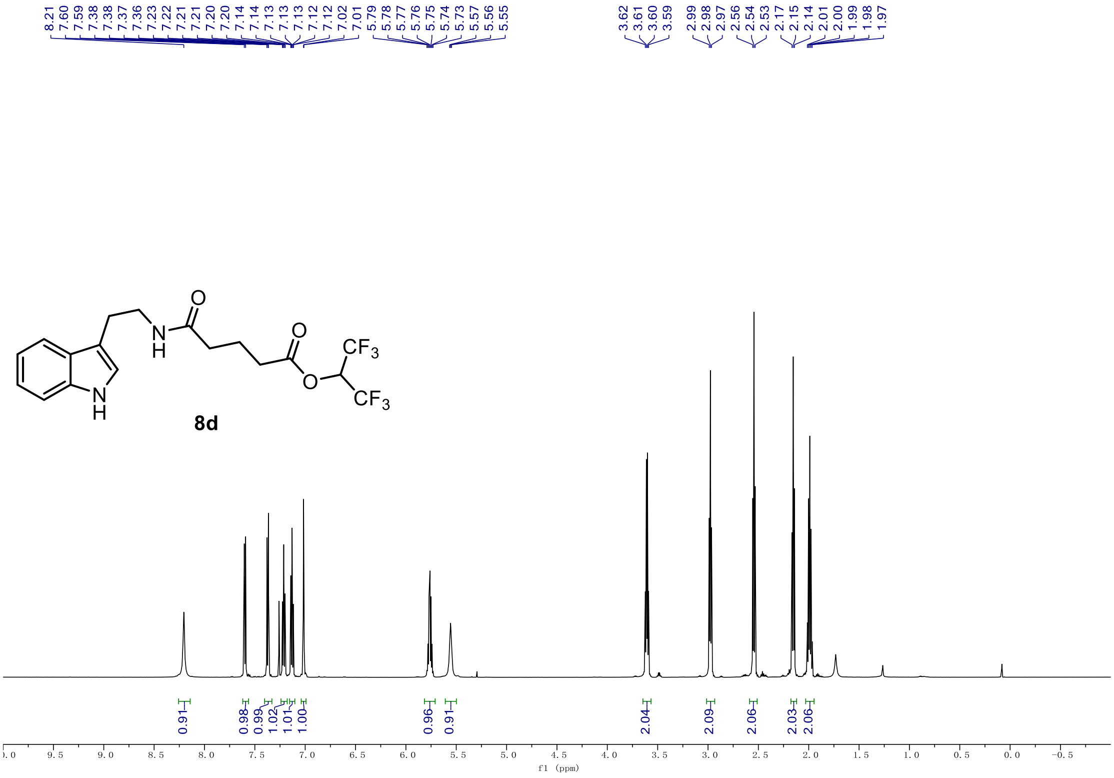

**$^{13}$C NMR** (151 MHz, $CDCl_3$) **8d**.

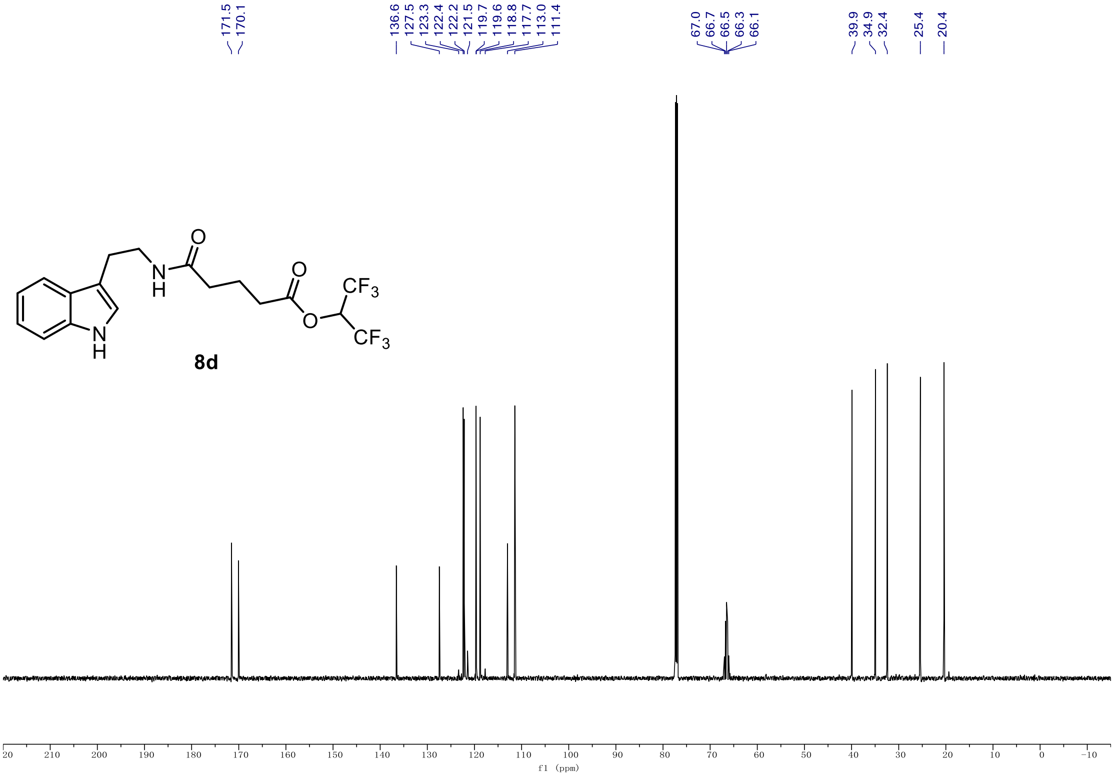

**$^{19}$F NMR** (565 MHz, $CDCl_3$) **8d**.

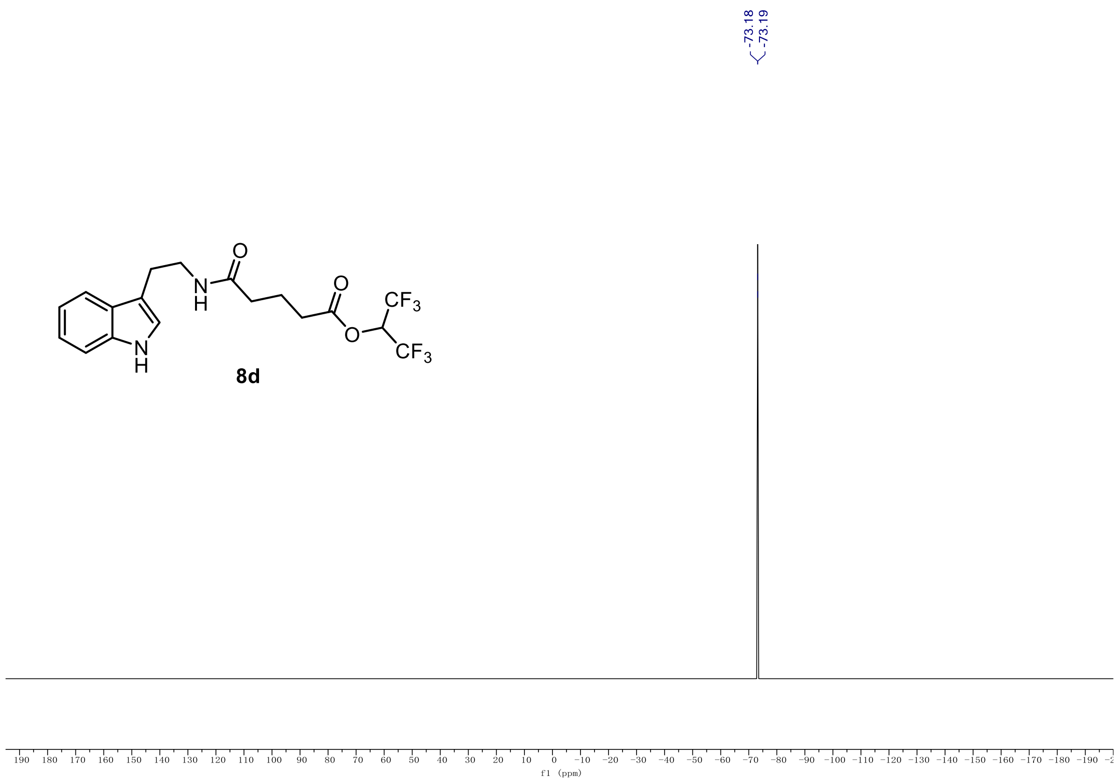


**$^{1}$H NMR** (500 MHz, $CDCl_3$) **8e**.

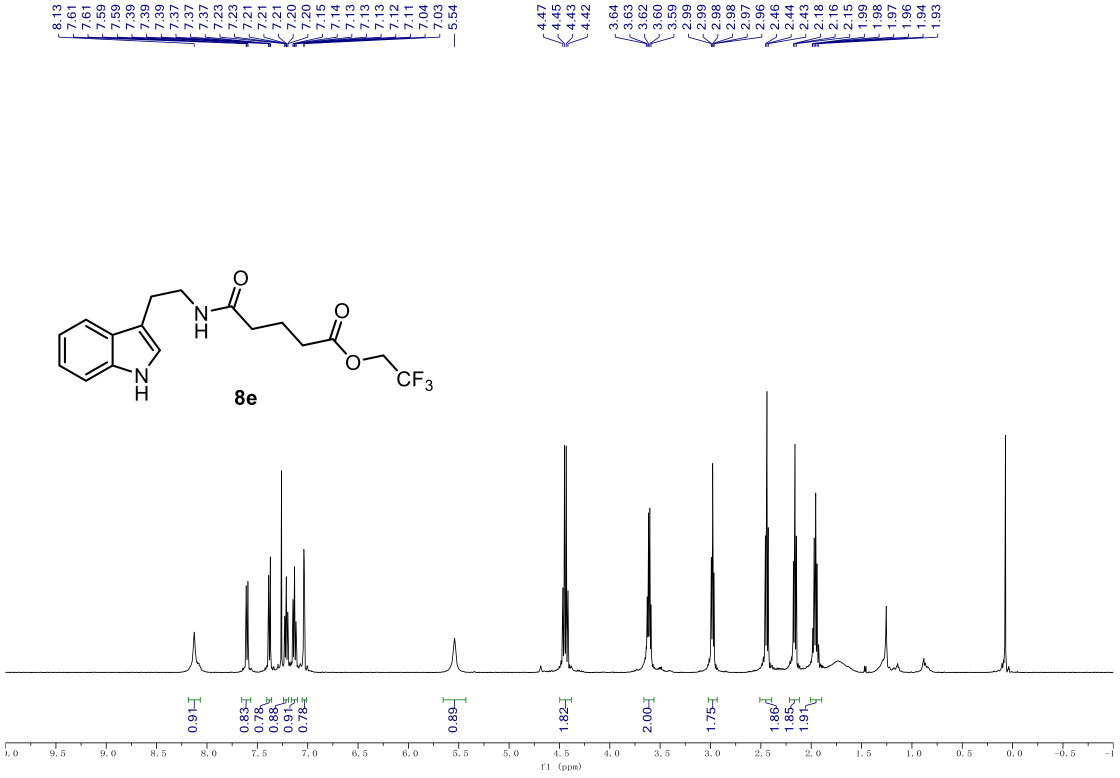

**$^{13}$C NMR** (126 MHz, $CDCl_3$) **8e**.

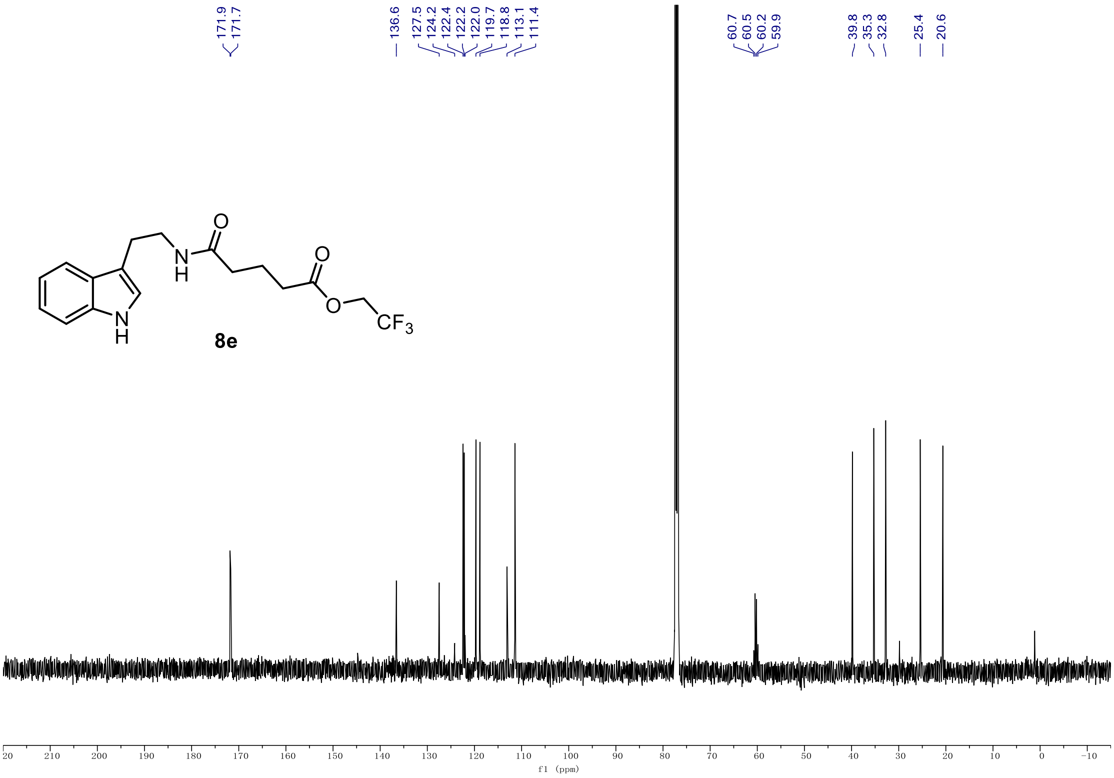


**$^{19}$F NMR** (471 MHz, $CDCl_3$) **8e**.

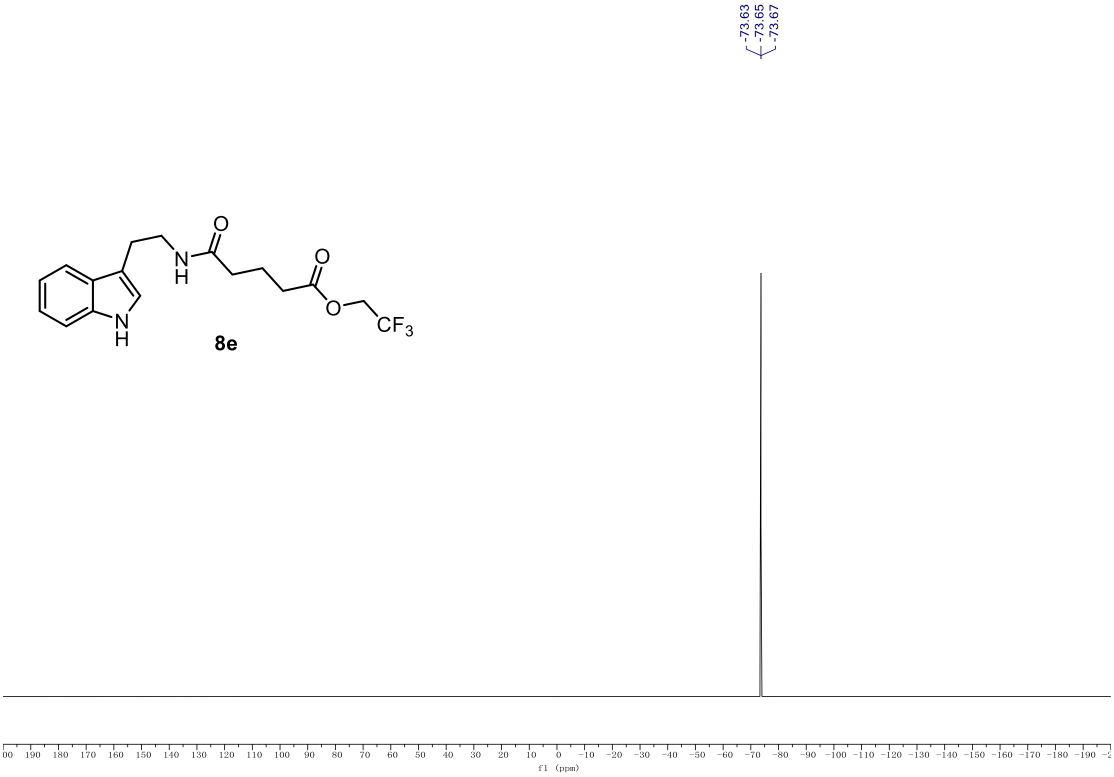

**¹H NMR** (500 MHz, $CD_3OD$) **8f**.

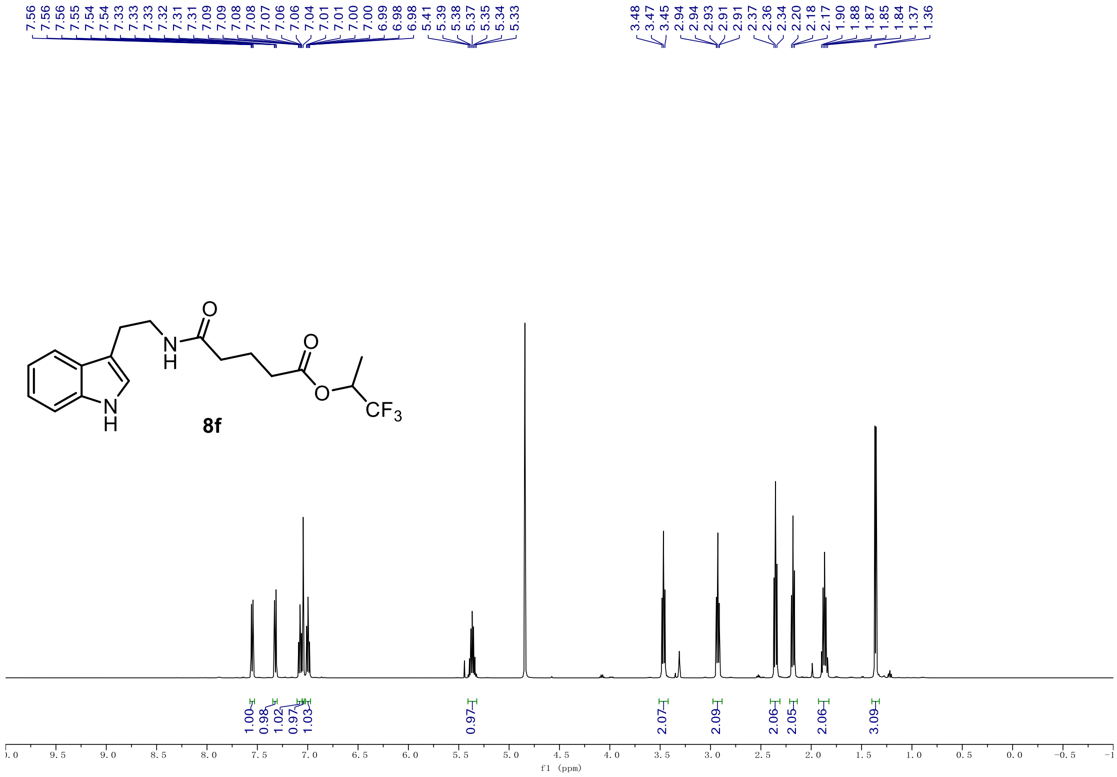


**¹³C NMR** (126 MHz, $CD_3OD$) **8f**.

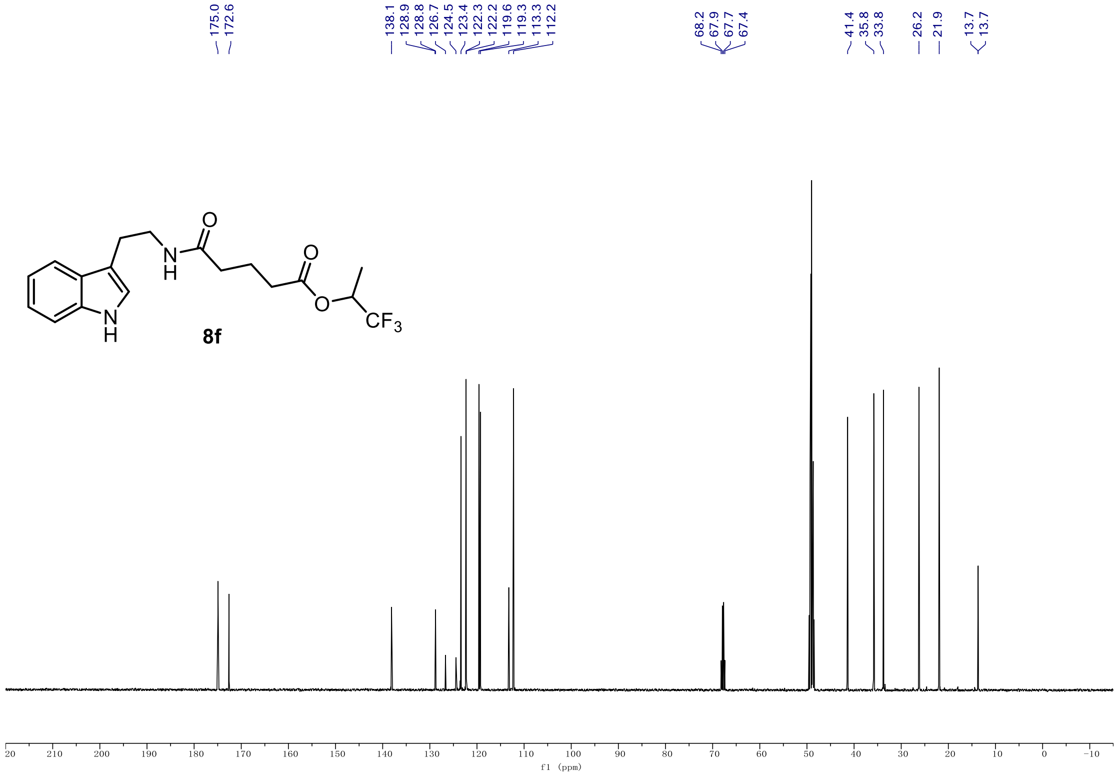

**$^{19}$F NMR** (471 MHz, $CD_3OD$) **8f**.

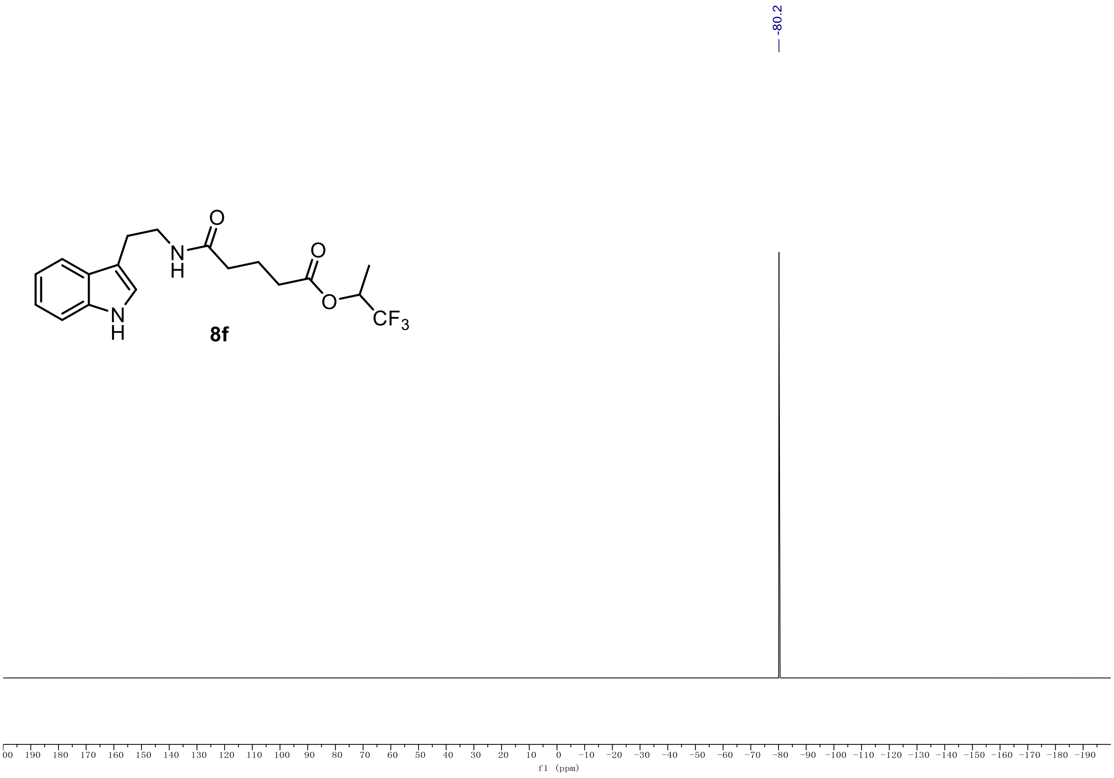


**$^{1}$H NMR** (500 MHz, $CD_3OD$) **8g**.

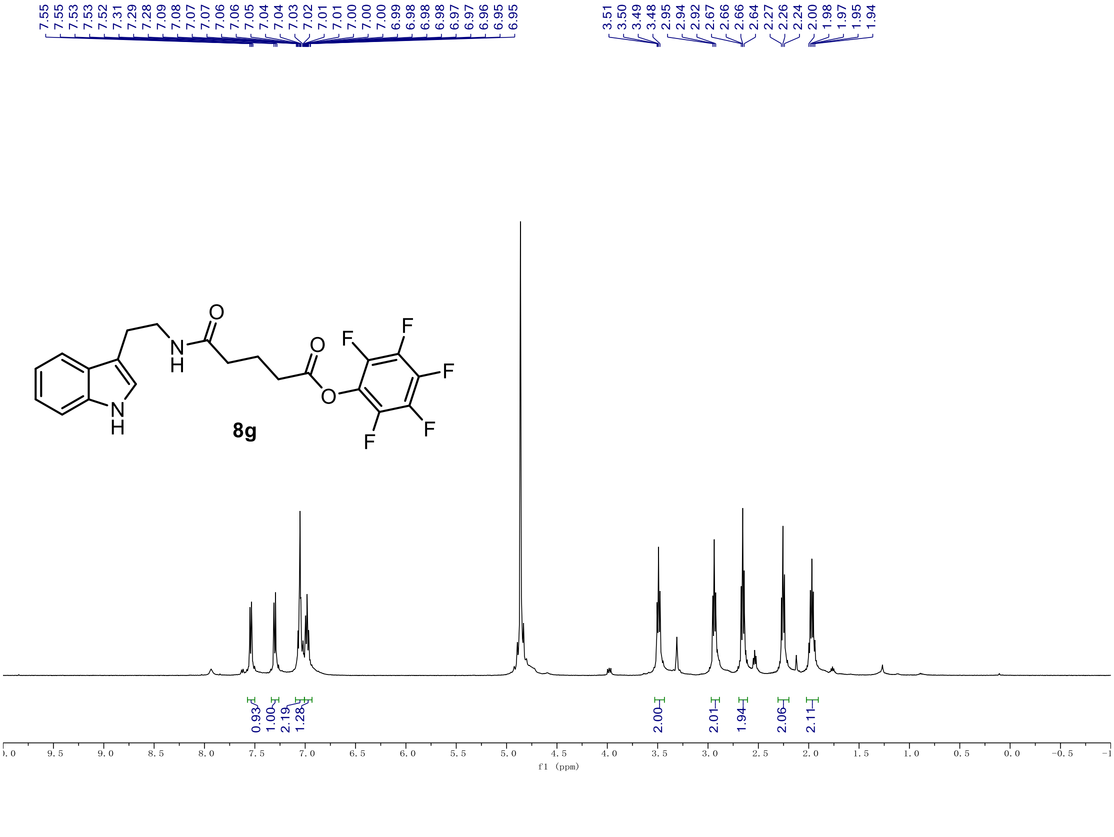

**¹³C NMR** (126 MHz, $CD_3OD$) **8g**.

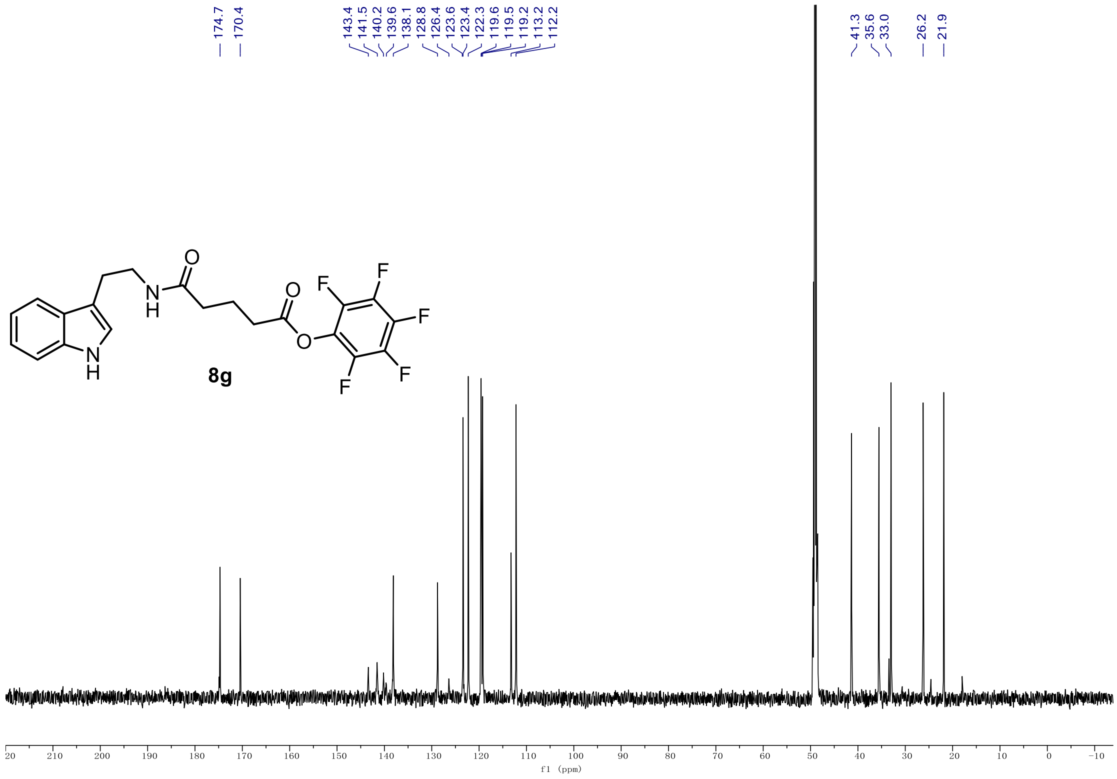


**¹⁹F NMR** (471 MHz, CD3OD) **8g**.

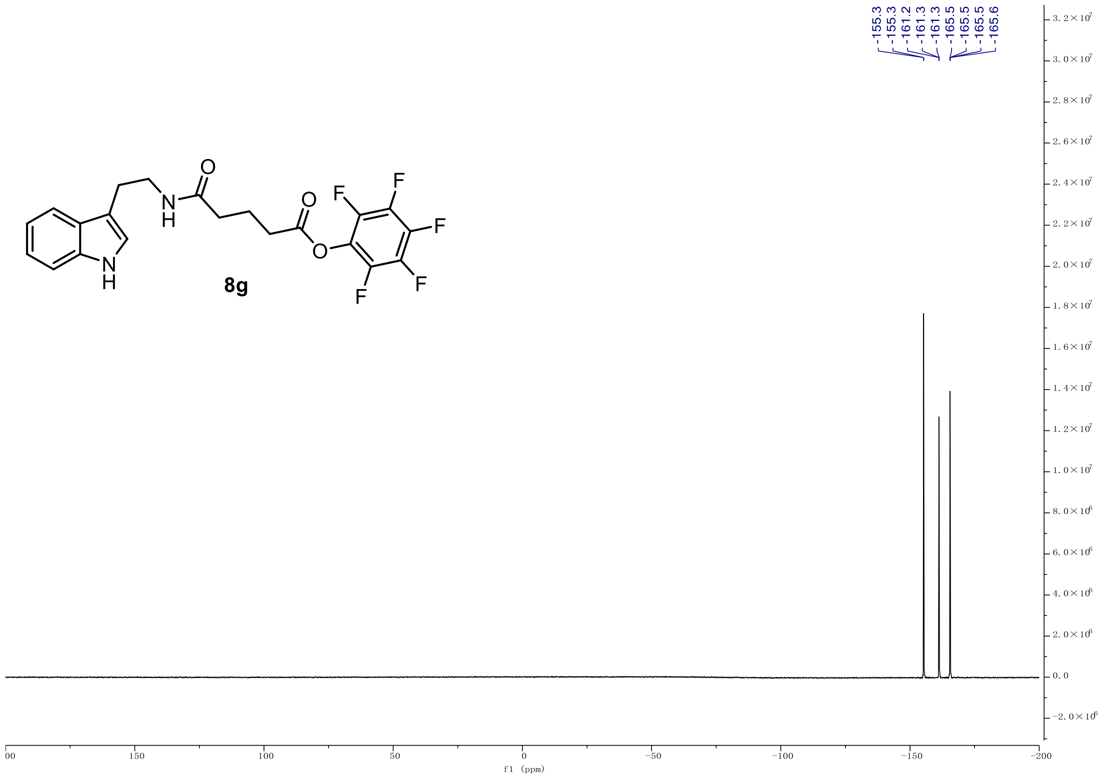

**$^{1}$H NMR** (500 MHz, $CDCl_3$) **8h**.

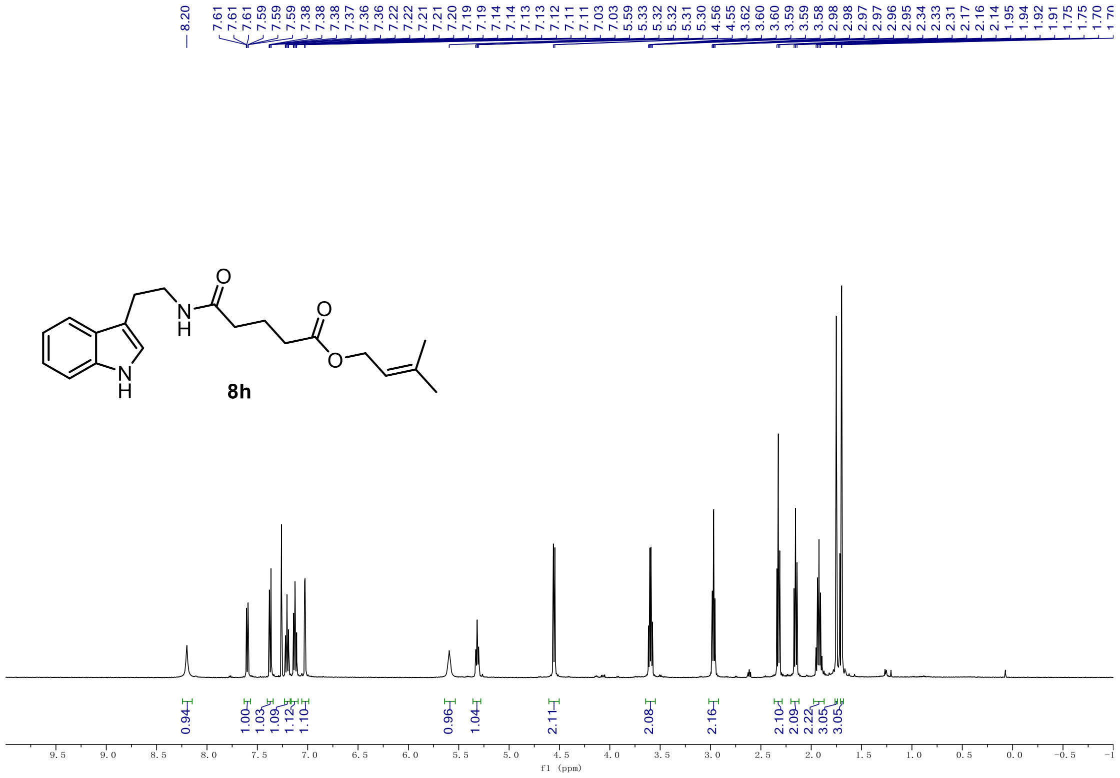


**$^{13}$C NMR** (126 MHz, $CDCl_3$) **8h**.

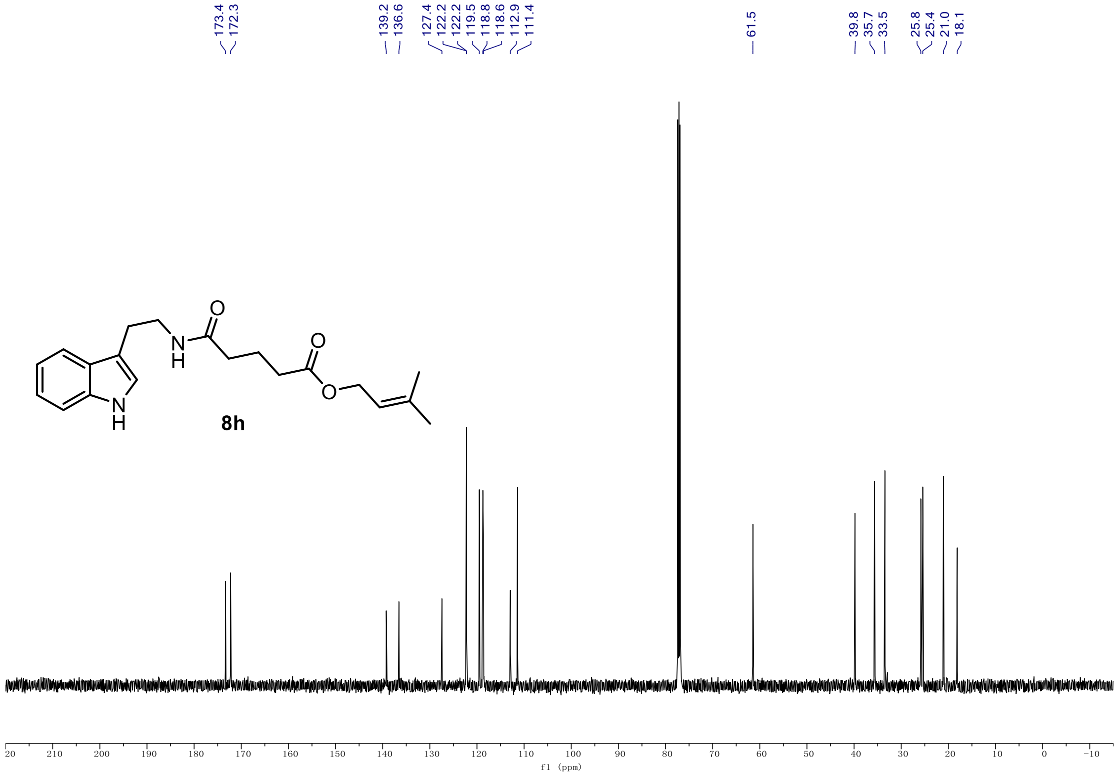

**$^{1}$H NMR** (500 MHz, $CD_2Cl_2$) **8i**.

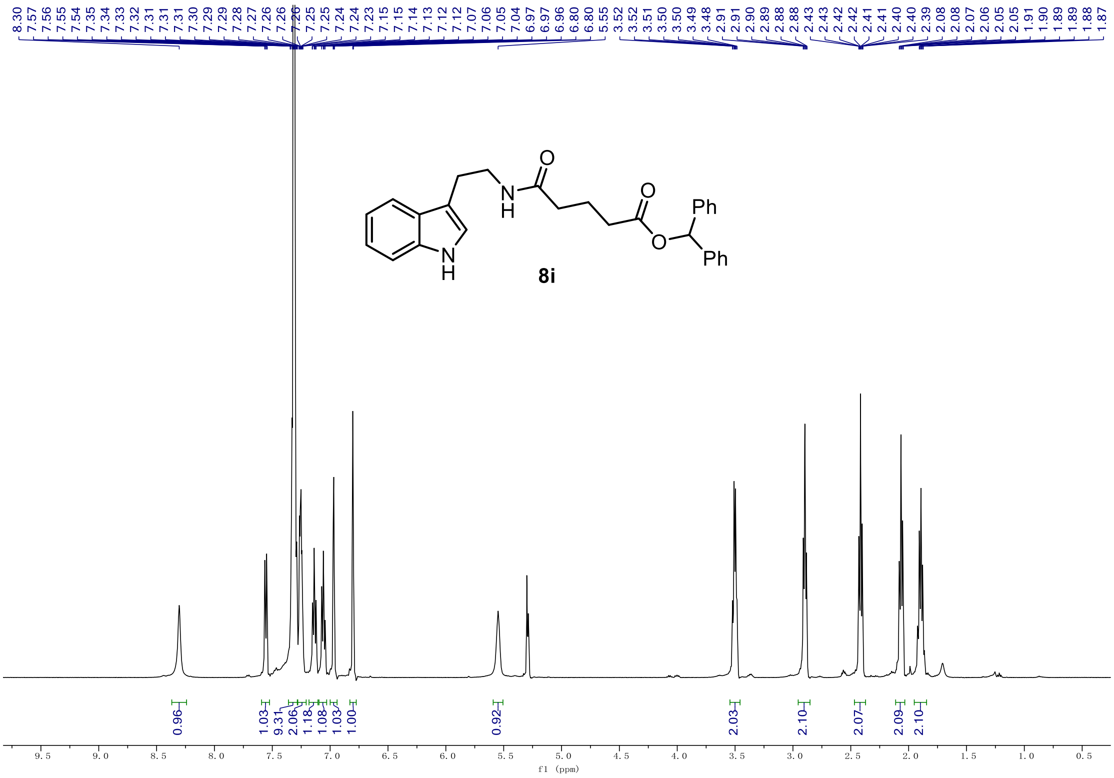


**$^{13}$C NMR** (126 MHz, $CD_2Cl_2$) **8i**.

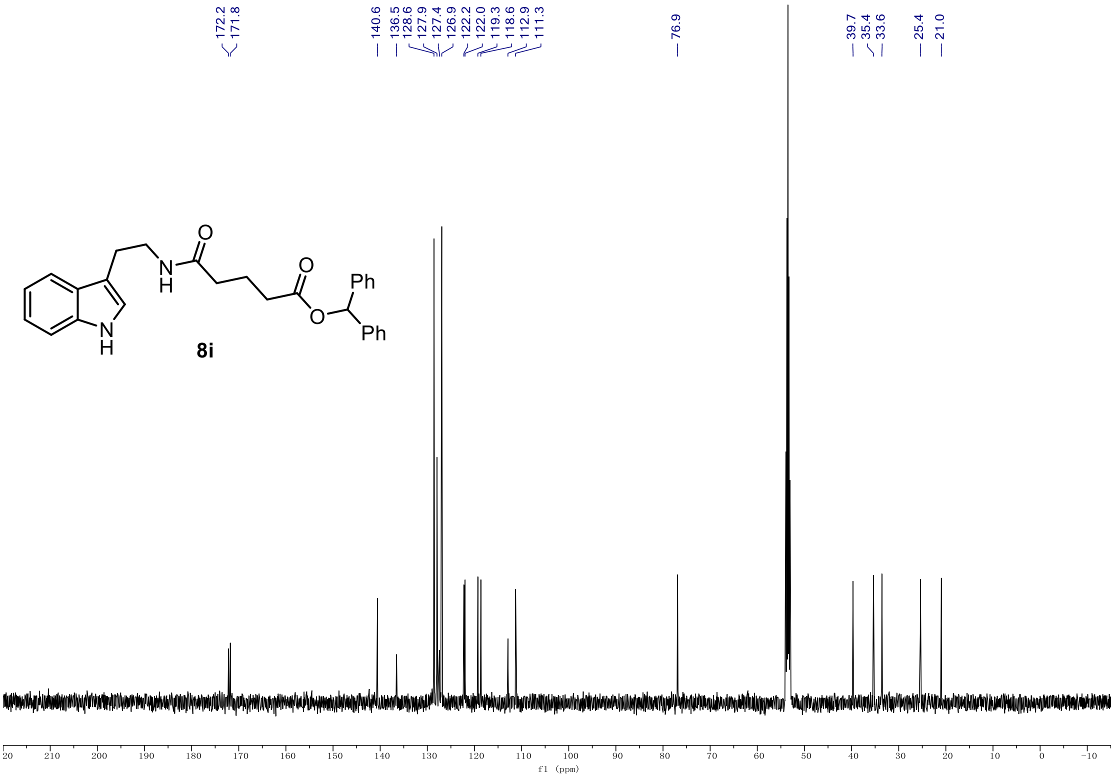

**$^{1}$H NMR** (700 MHz, $CDCl_3$) **8j**.

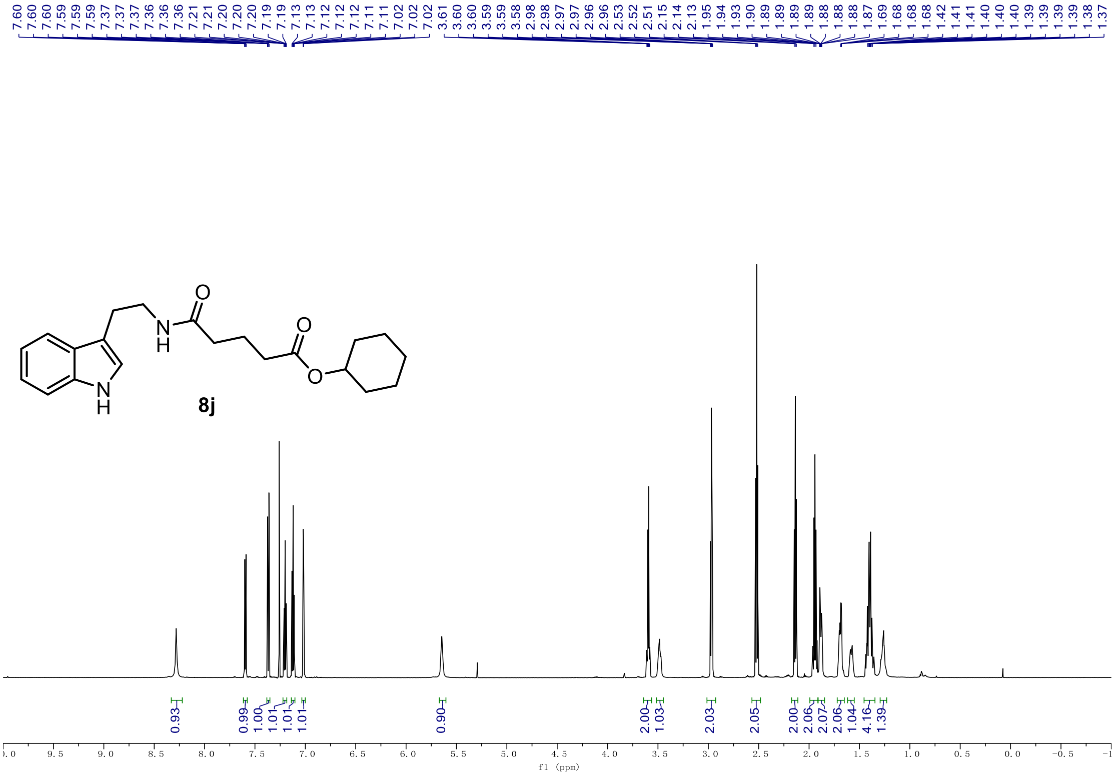

**$^{13}$C NMR** (126 MHz, $CDCl_3$) **8j**.

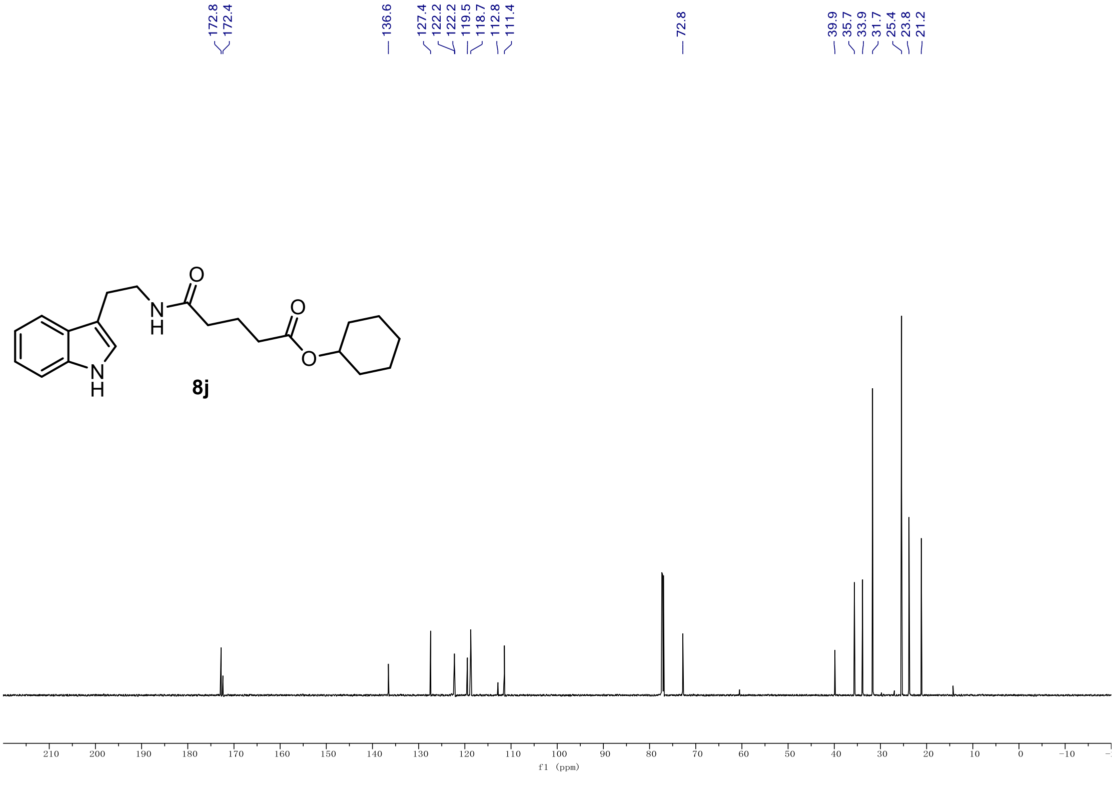

**$^{1}$H NMR** (500 MHz, $CDCl_3$) **8k**.

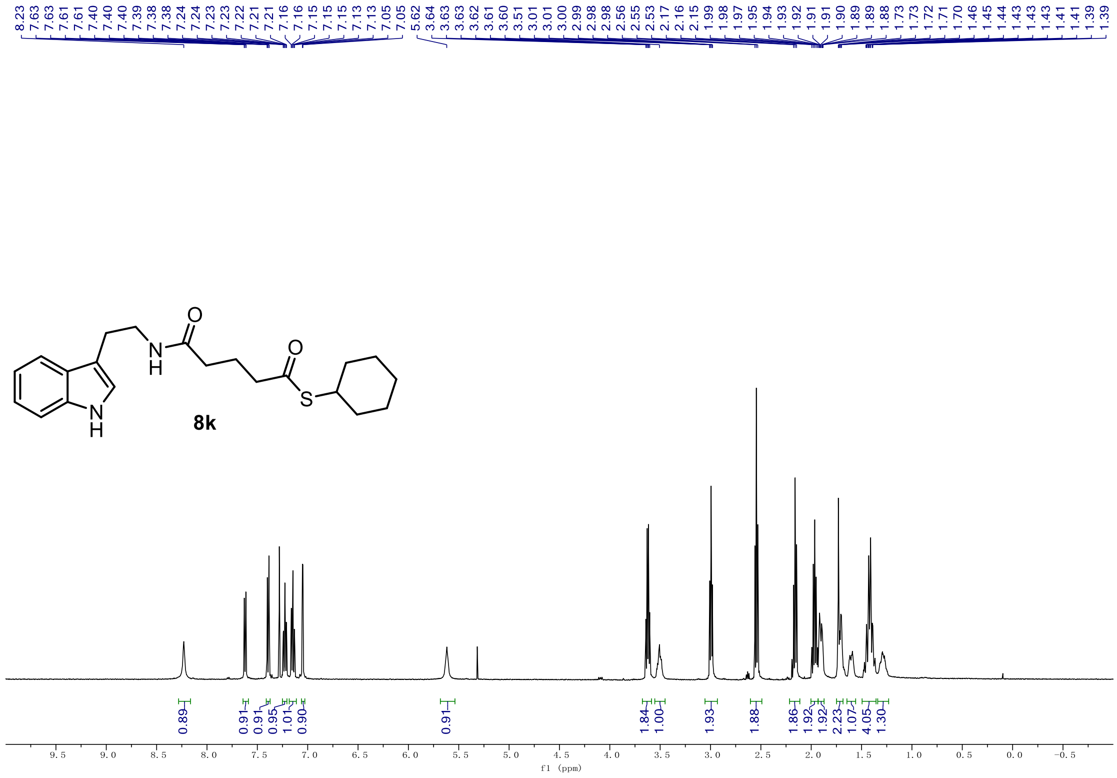


**$^{13}$C NMR** (176 MHz, $CDCl_3$) **8k**.

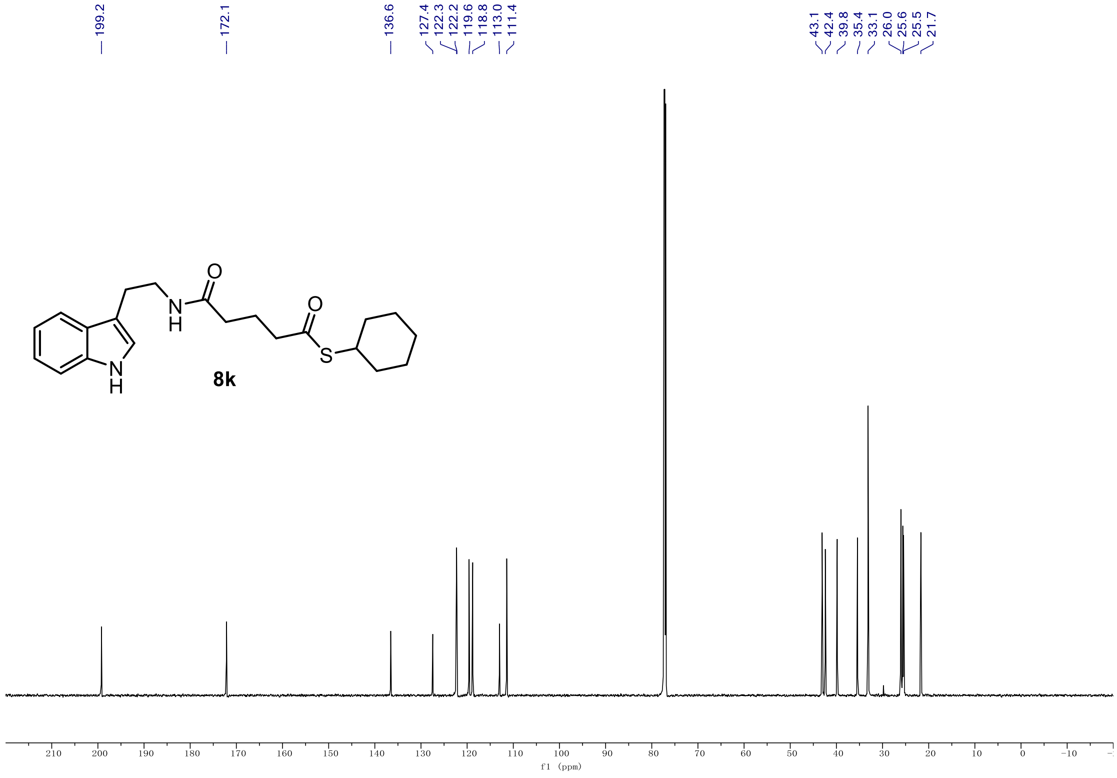

**$^{1}$H NMR** (600 MHz, $CDCl_3$) **8l**.

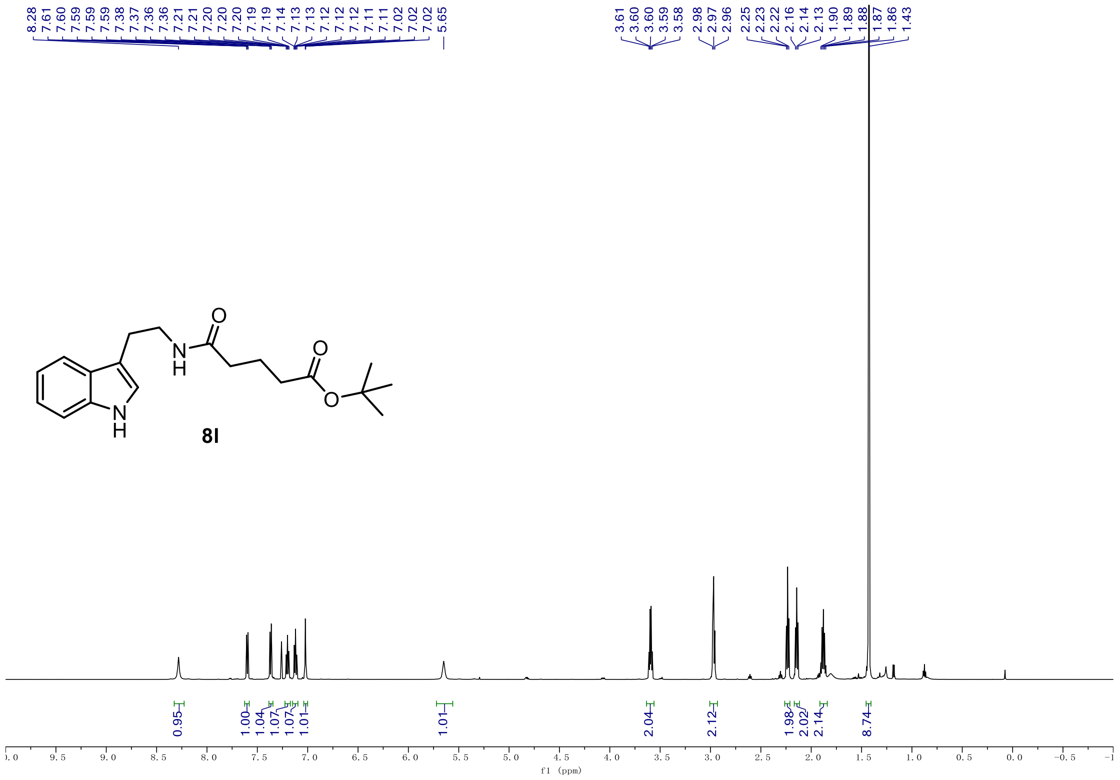


**$^{13}$C NMR** (151 MHz, $CDCl_3$) **8l**.

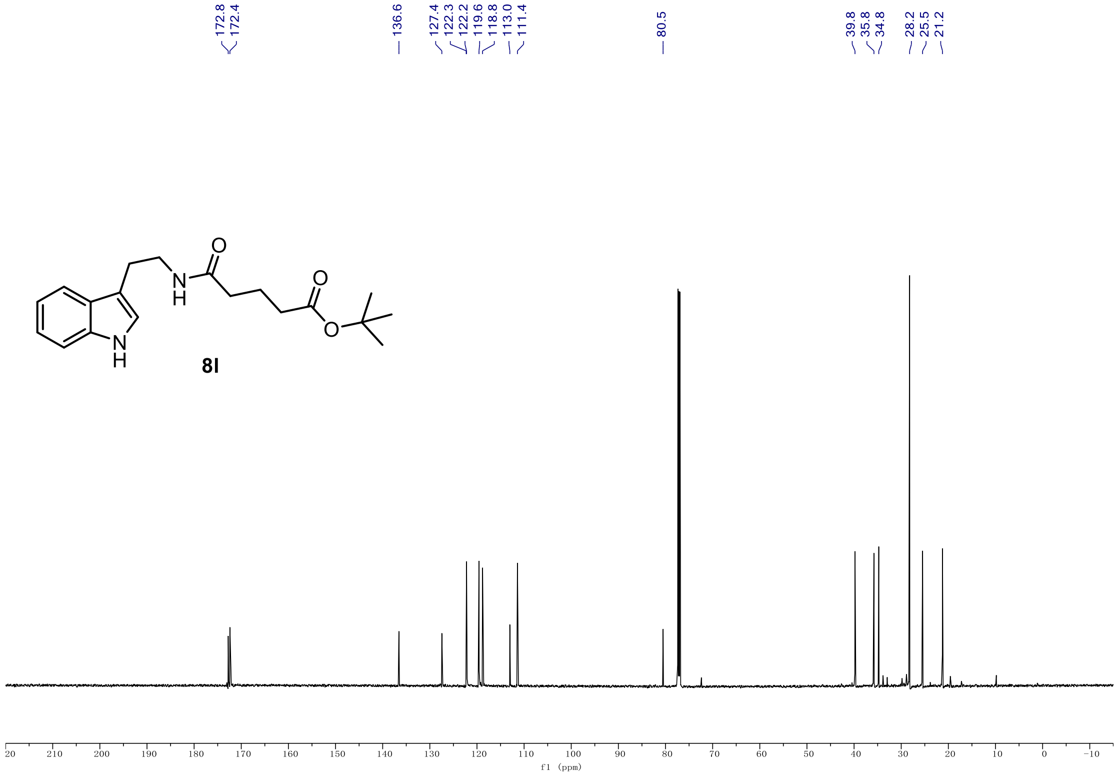

**$^{1}$H NMR** (500 MHz, $CDCl_3$) **11a**.

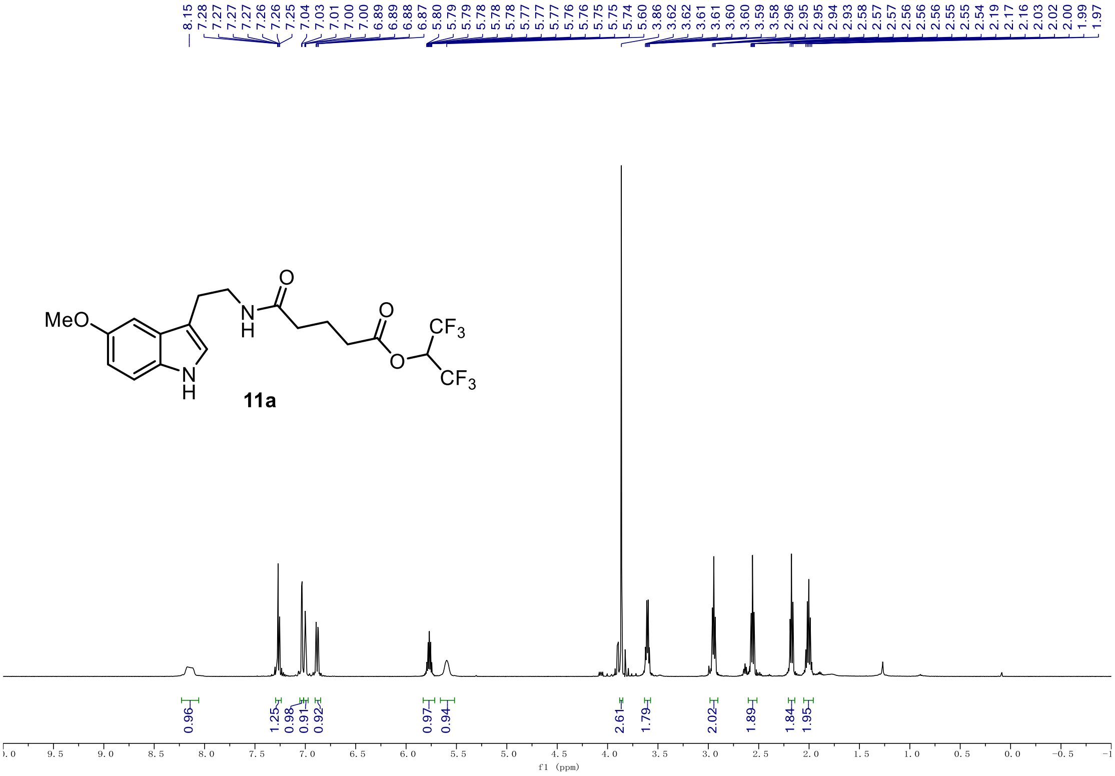


**$^{13}$C NMR** (126 MHz, $CDCl_3$) **11a**.

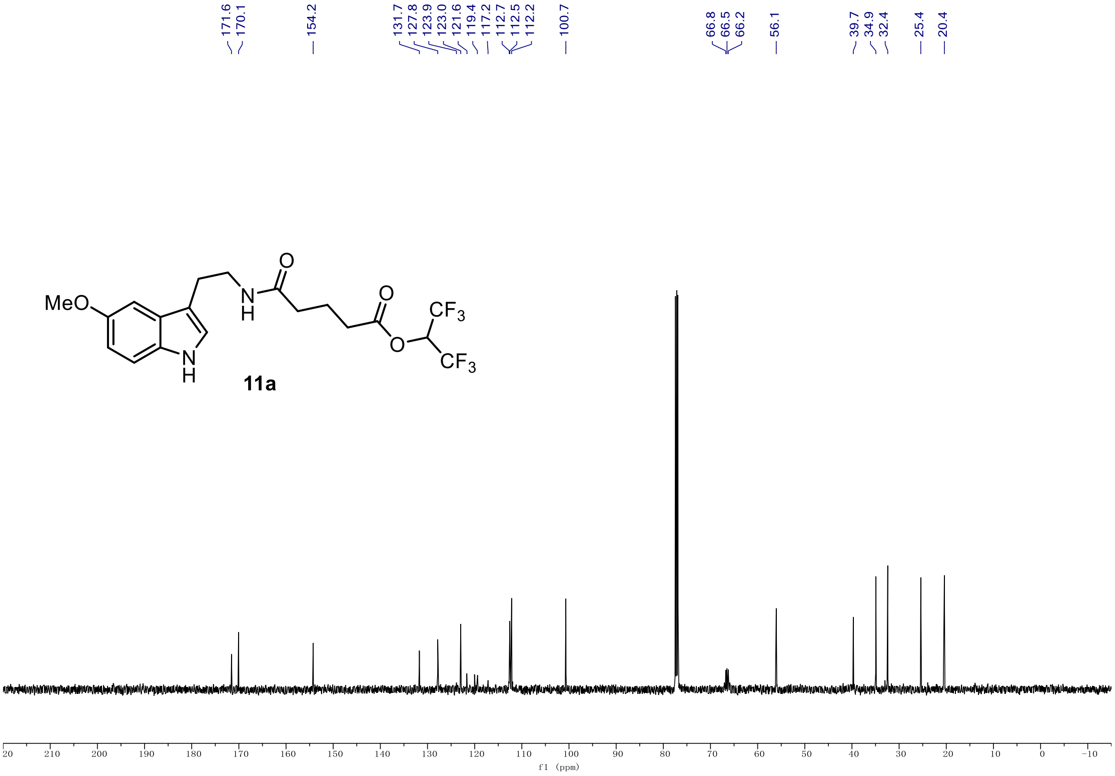

**$^{19}$F NMR** (565 MHz, $CDCl_3$) **11a**.

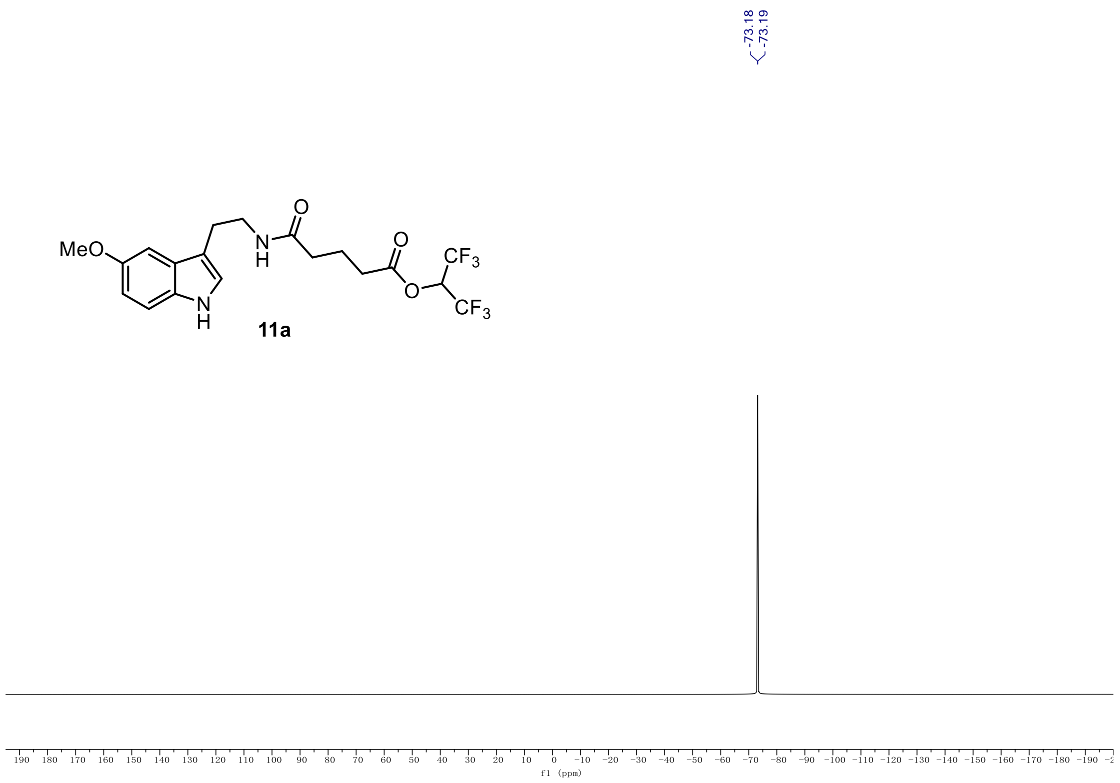

**$^{1}$H NMR** (500 MHz, $CD_2Cl_2$) **11b**.

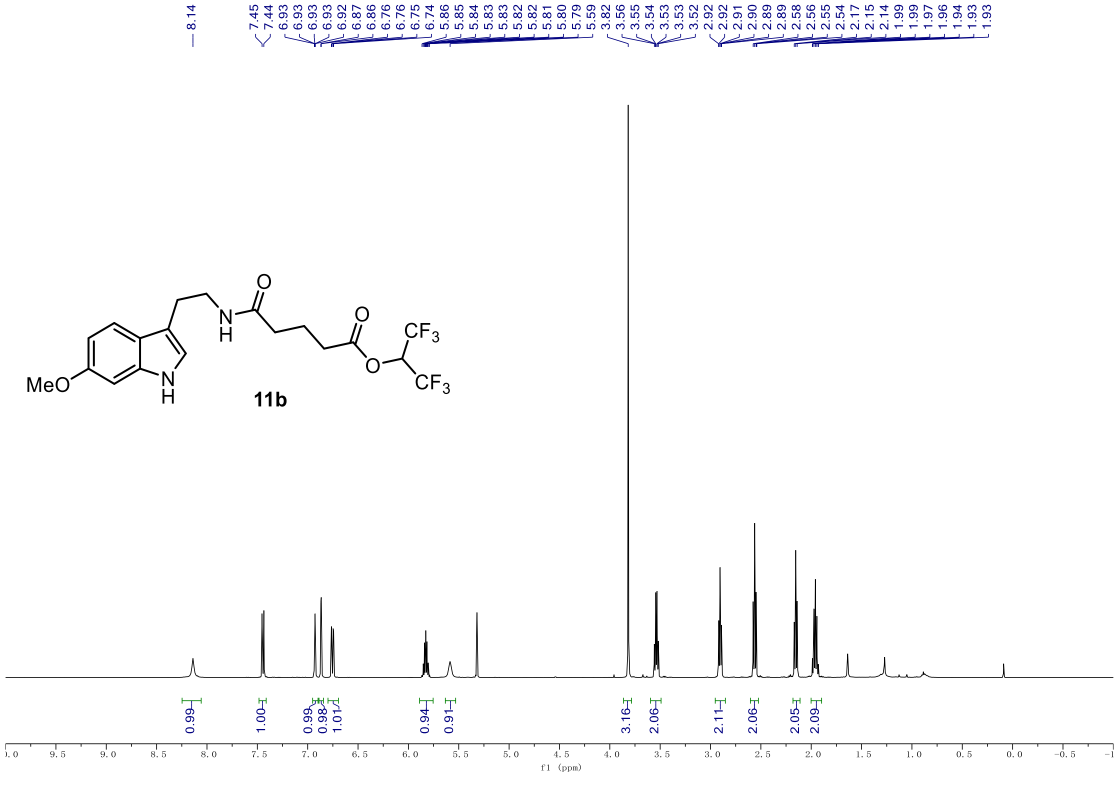

**$^{13}$C NMR** (126 MHz, $CD_2Cl_2$) **11b**.

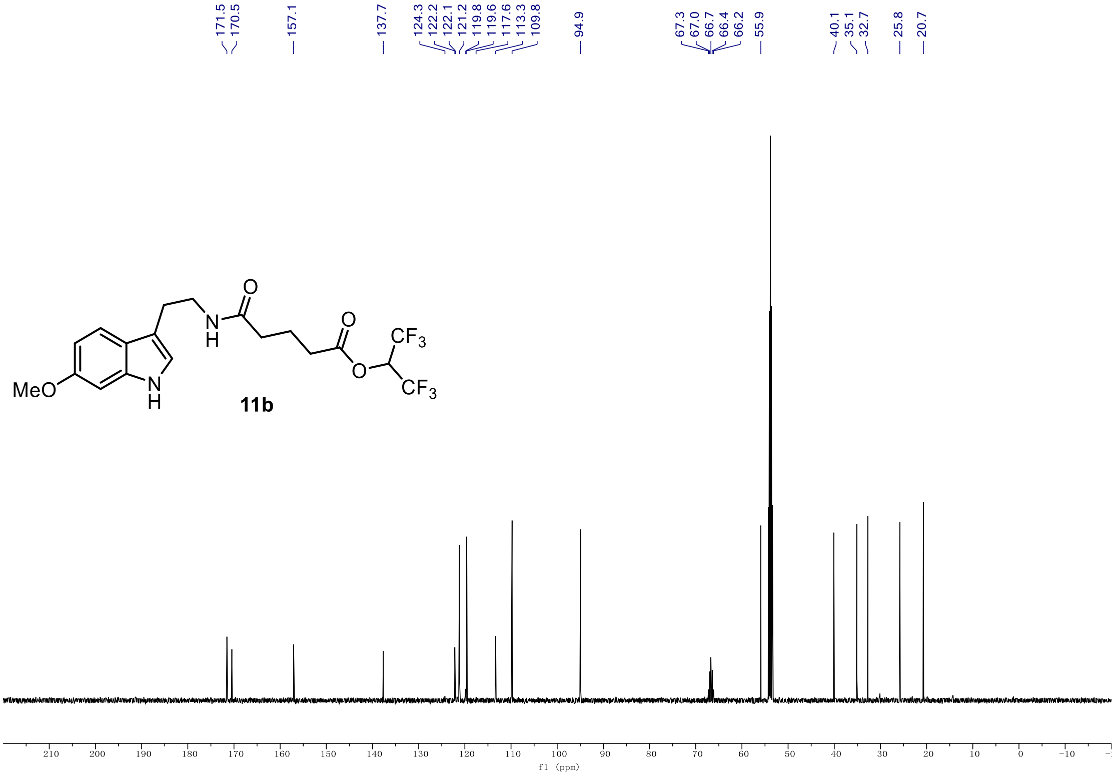


**$^{19}$F NMR** (471 MHz, $CD_2Cl_2$) **11b**.

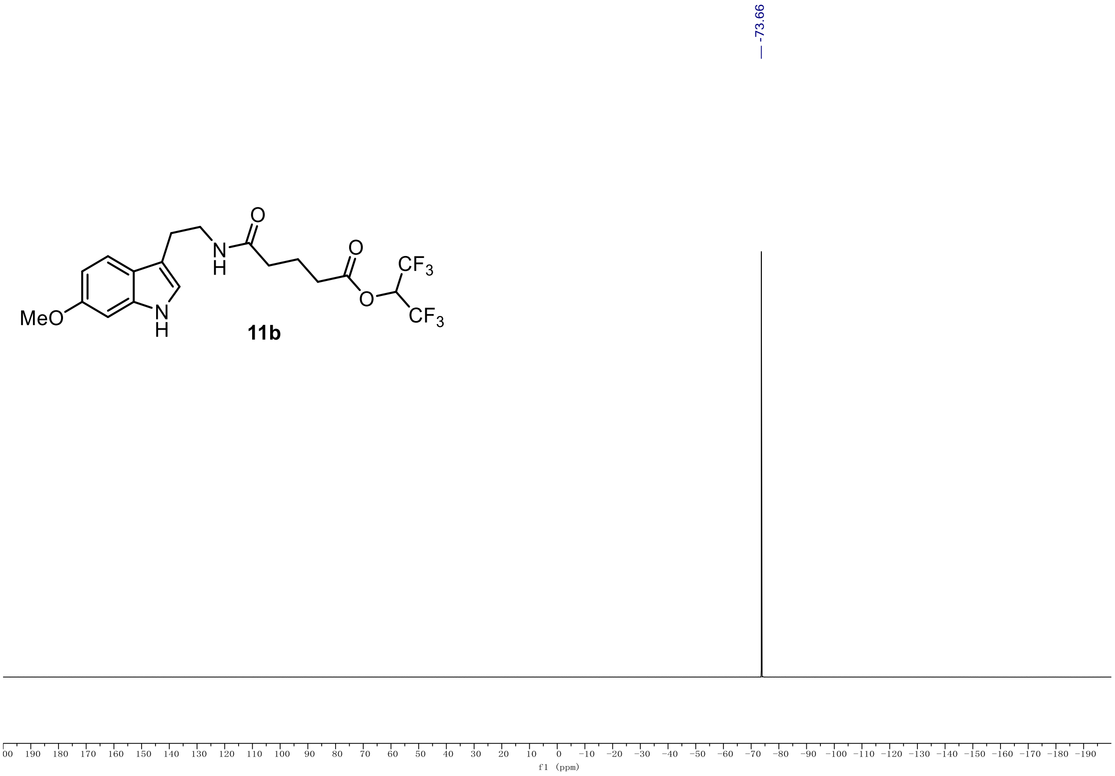

**$^{1}$H NMR** (700 MHz, $CDCl_3$) **11c**.

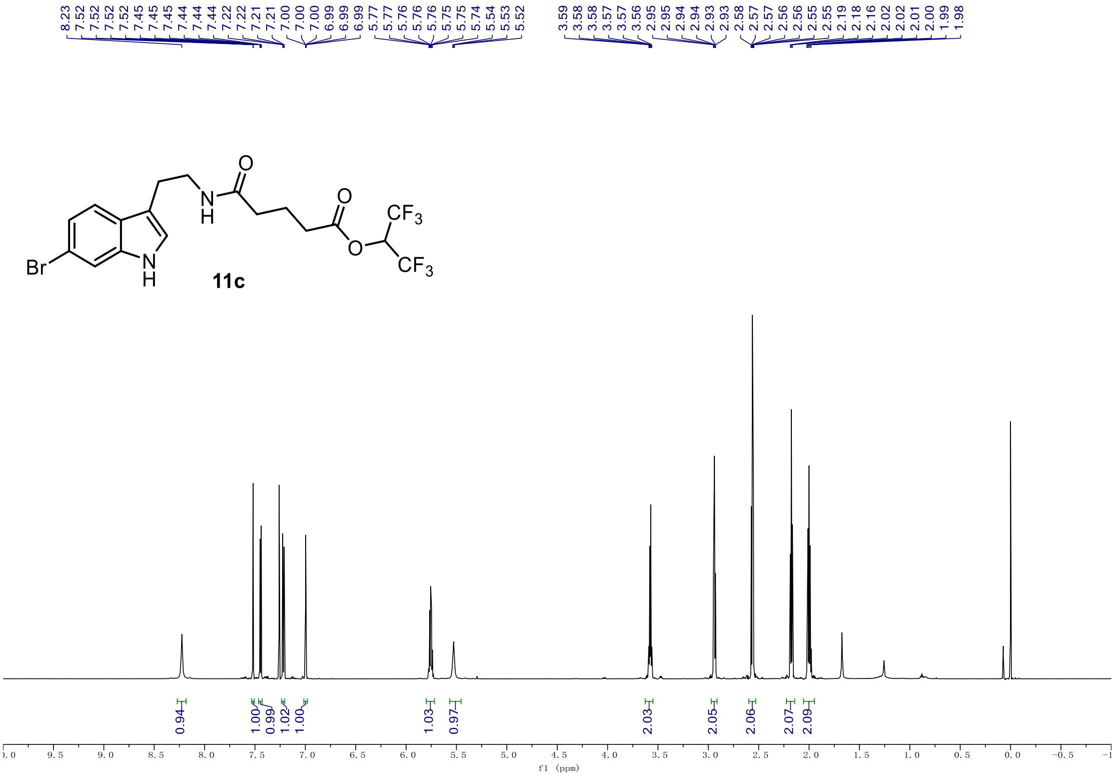


**$^{13}$C NMR** (176 MHz, $CDCl_3$) **11c**.

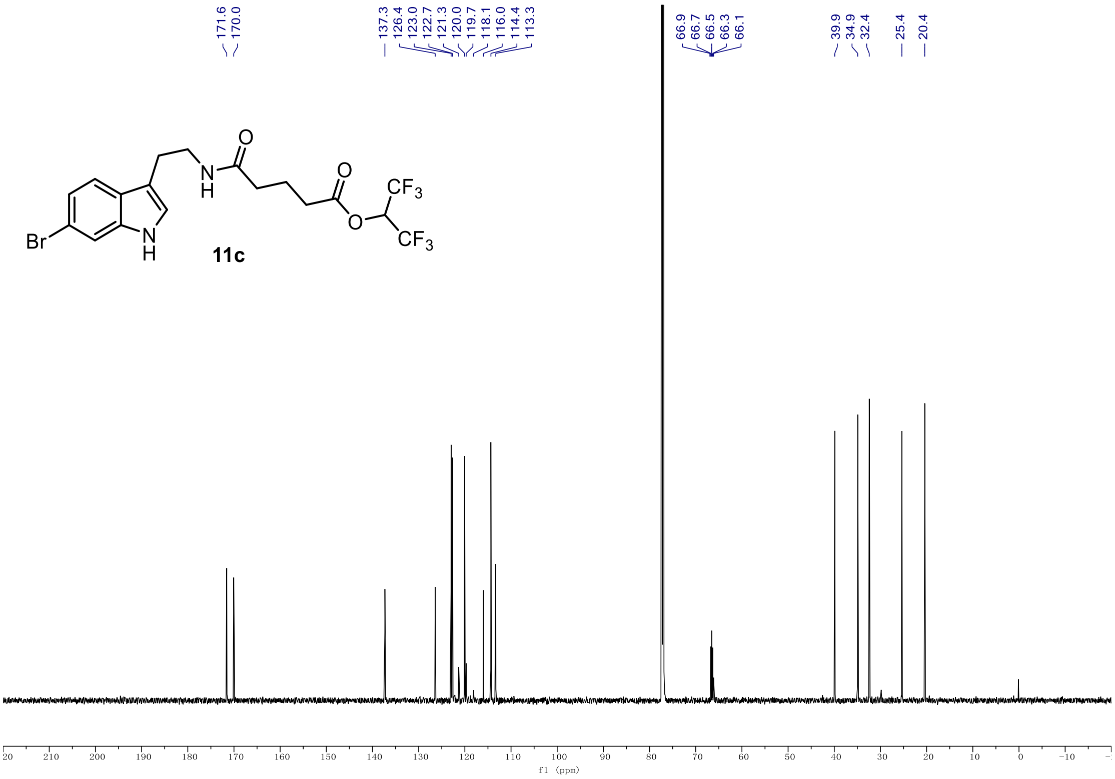

**$^{19}$F NMR** (471 MHz, $CDCl_3$) **11c**.

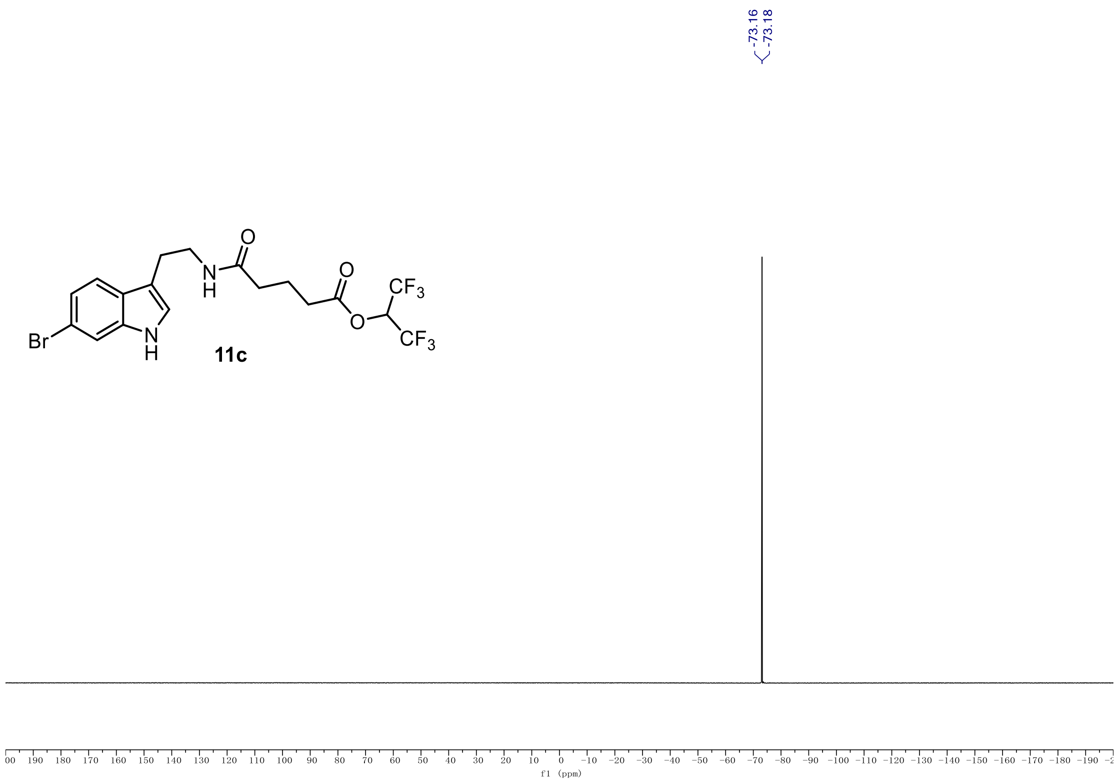


**$^{1}$H NMR** (400 MHz, $CDCl_3$) **11d**.

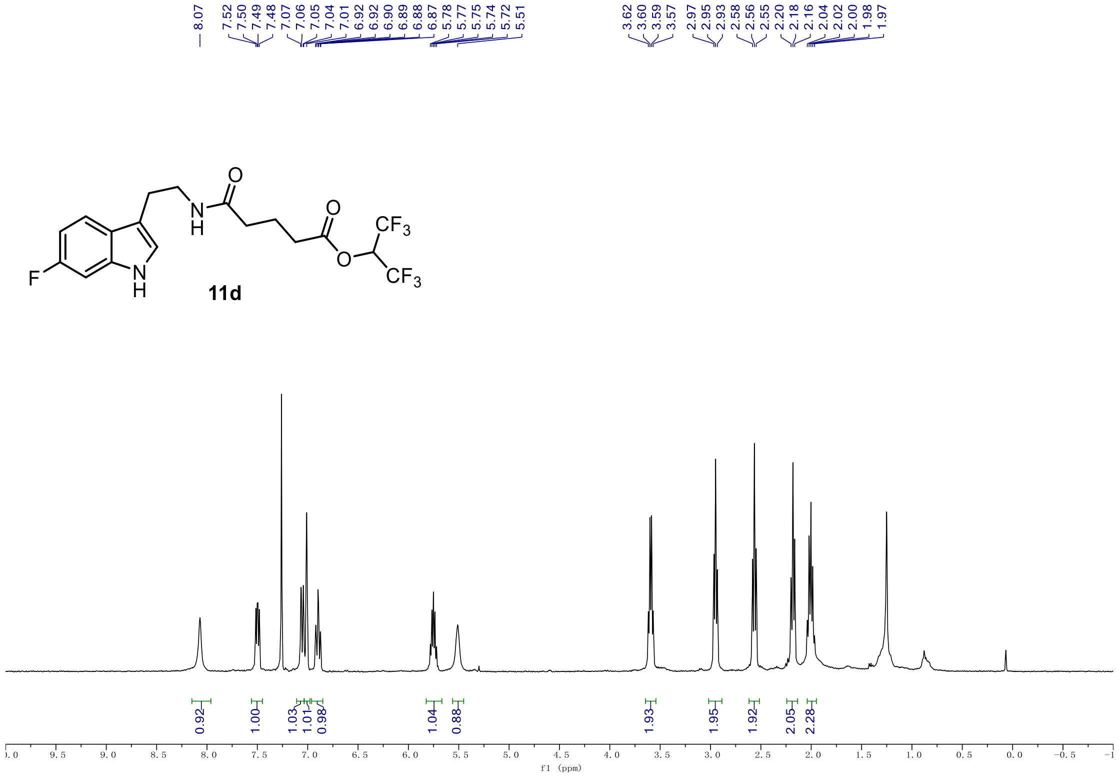

**$^{13}$C NMR** (176 MHz, $CDCl_3$) **11d**.

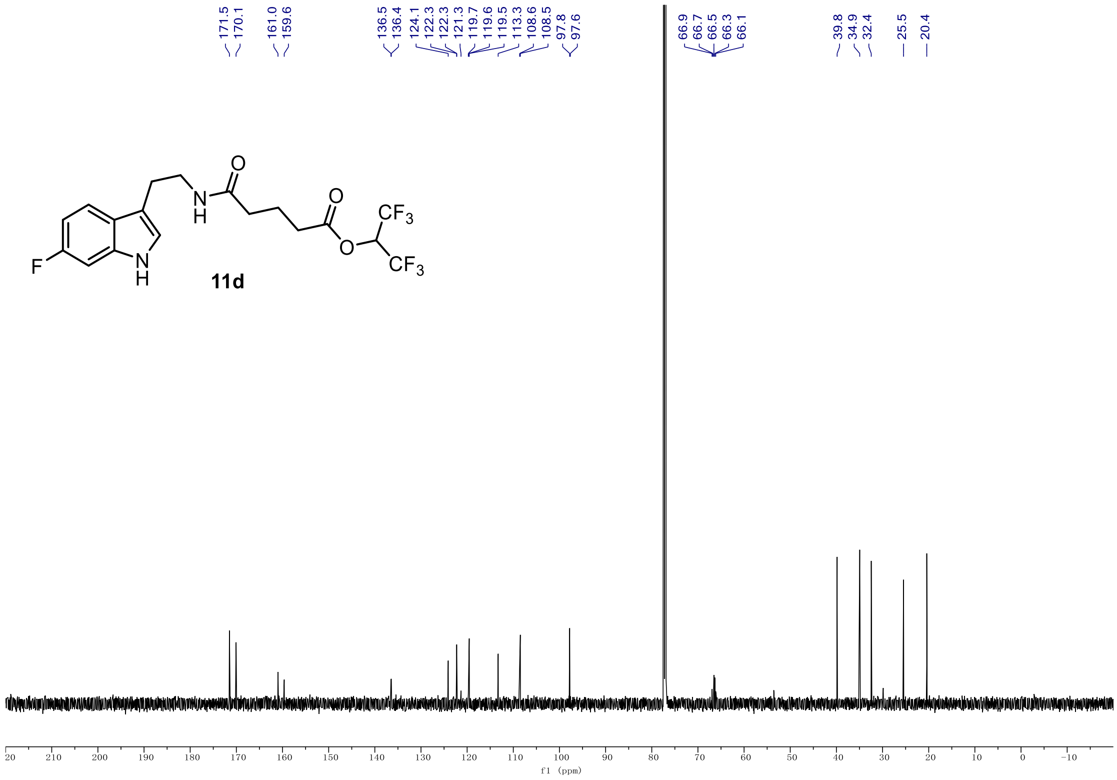


**$^{19}$F NMR** (471 MHz, $CDCl_3$) **11d**.

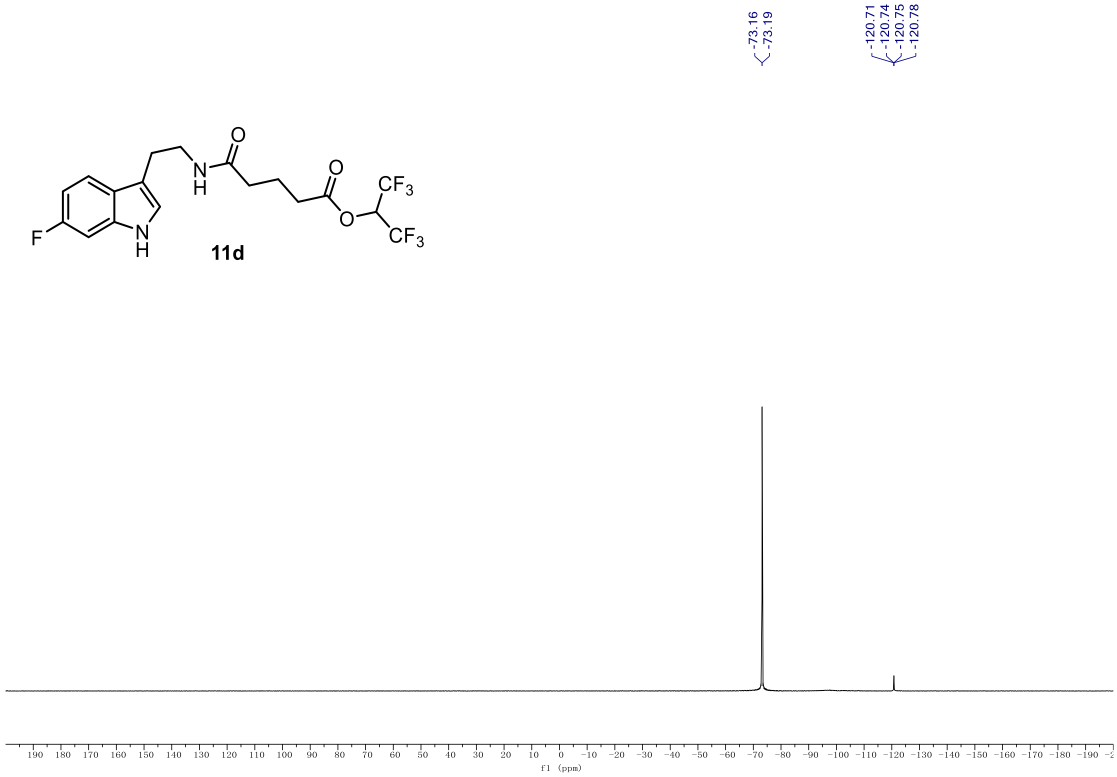

**$^{1}$H NMR** (500 MHz, $CD_2Cl_2$) **11e**.

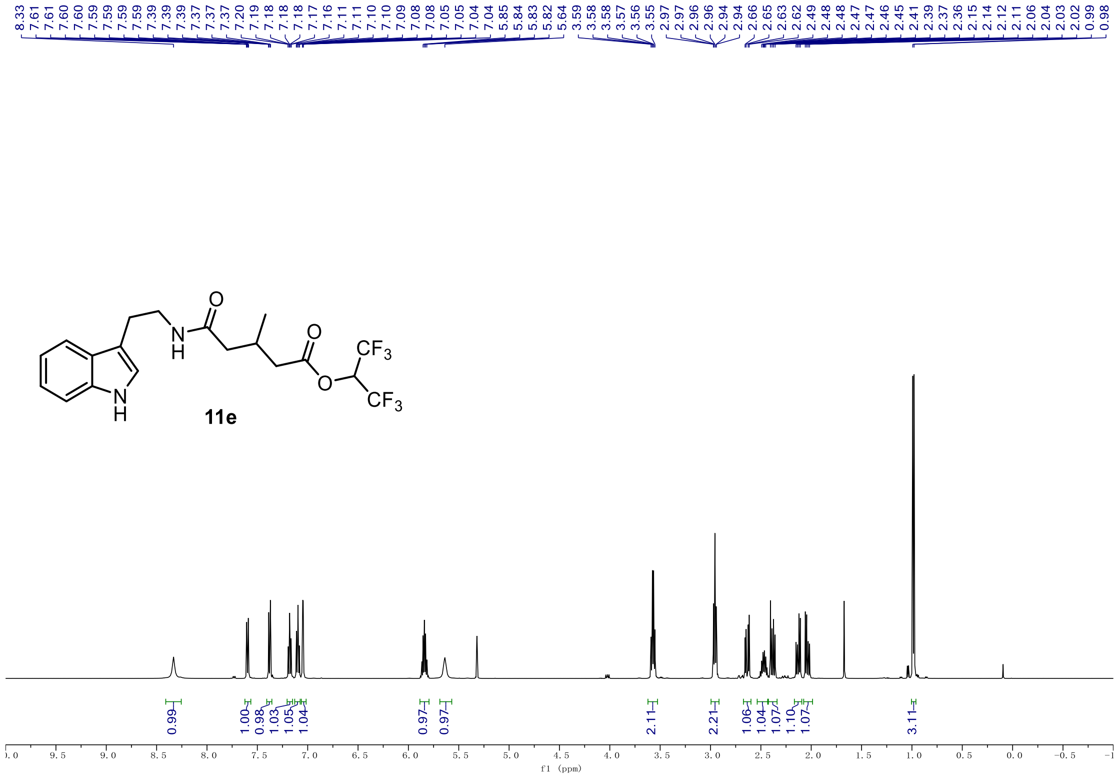


**$^{13}$C NMR** (126 MHz, $CD_2Cl_2$) **11e**.

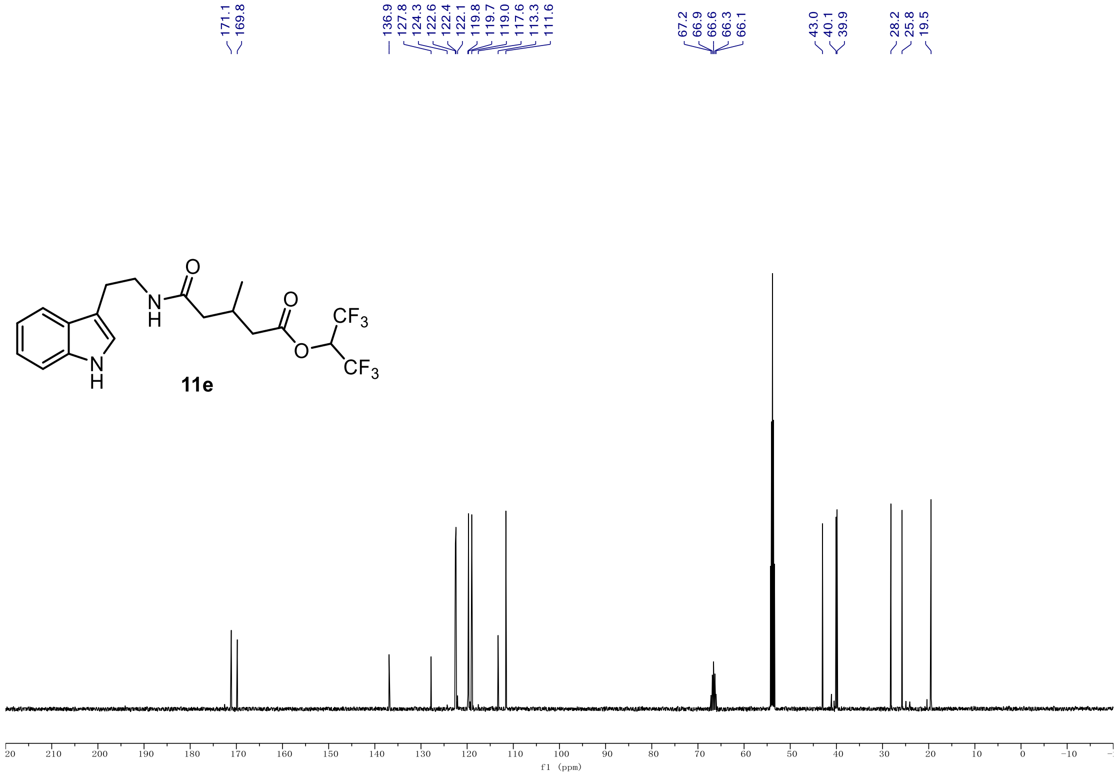

**$^{19}$F NMR** (471 MHz, $CD_2Cl_2$) **11e**.

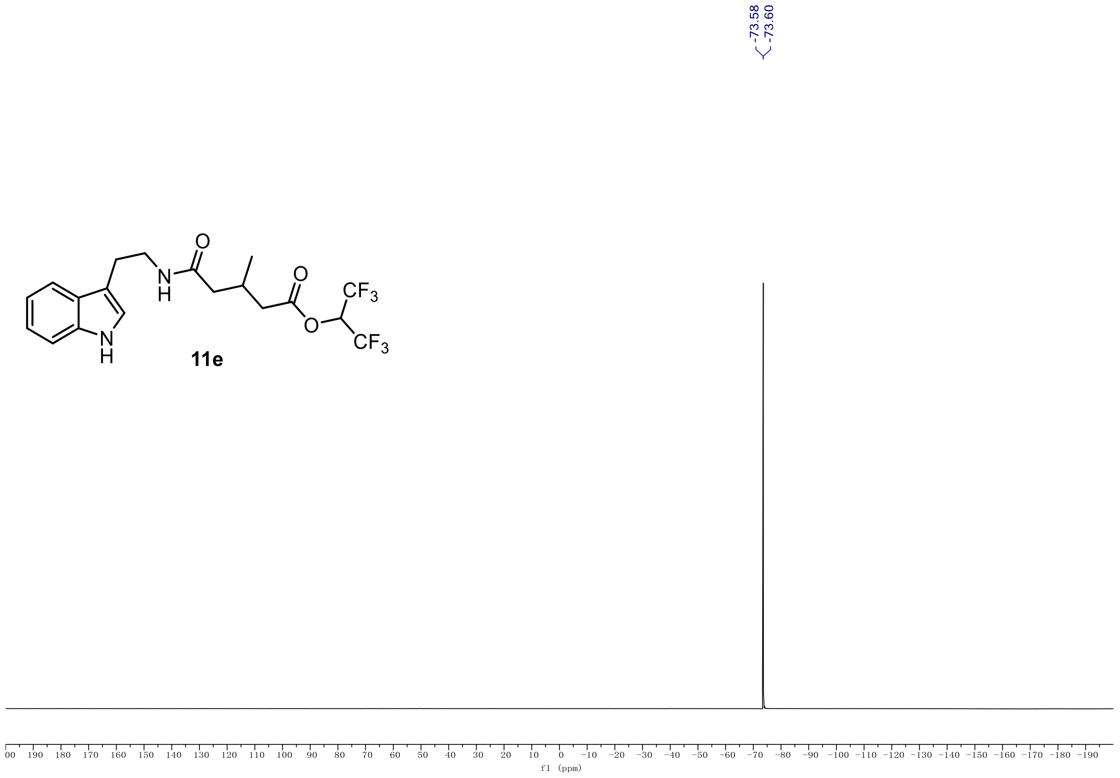


**$^{1}$H NMR** (500 MHz, $CD_2Cl_2$) **11f**.

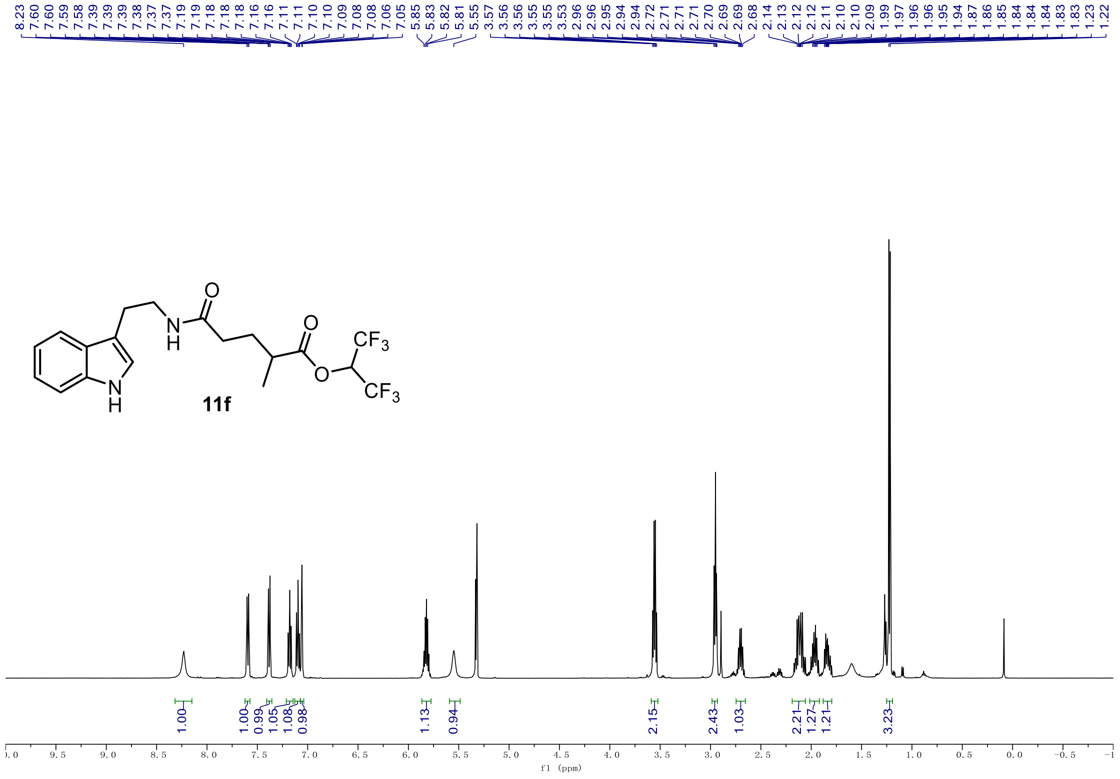

**$^{1}$H NMR** (500 MHz, $CD_2Cl_2$) **11f**.

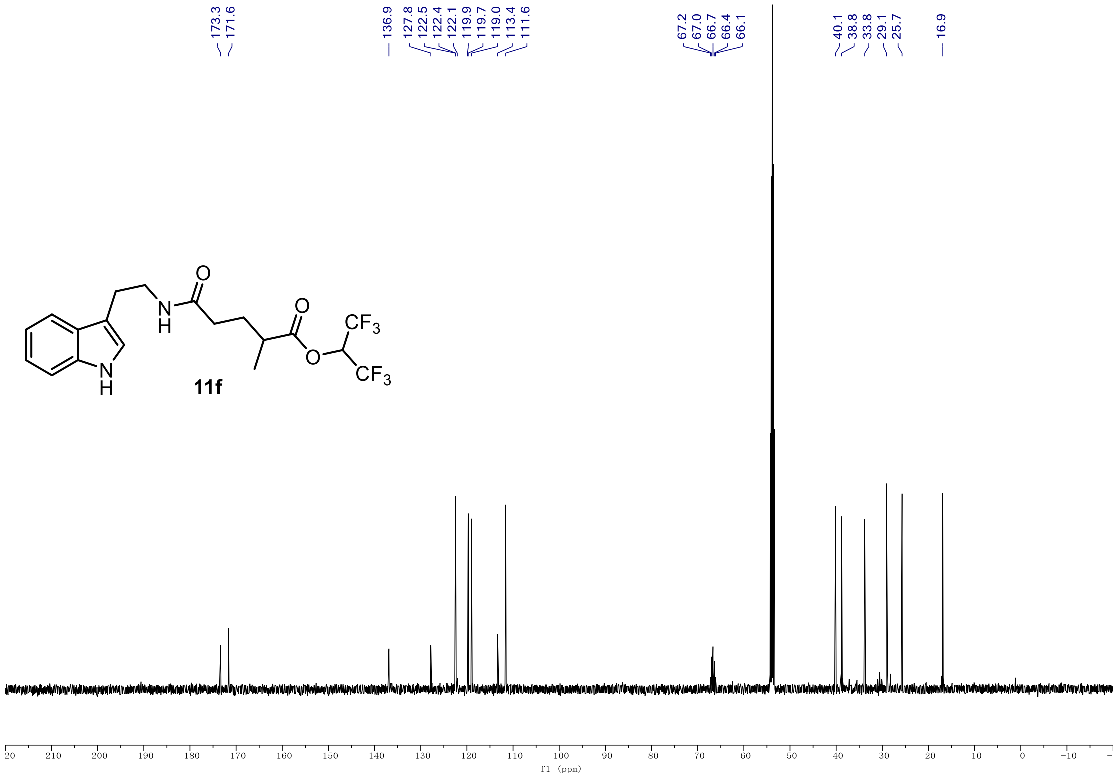


**$^{1}$H NMR** (500 MHz, $CD_2Cl_2$) **11f**.

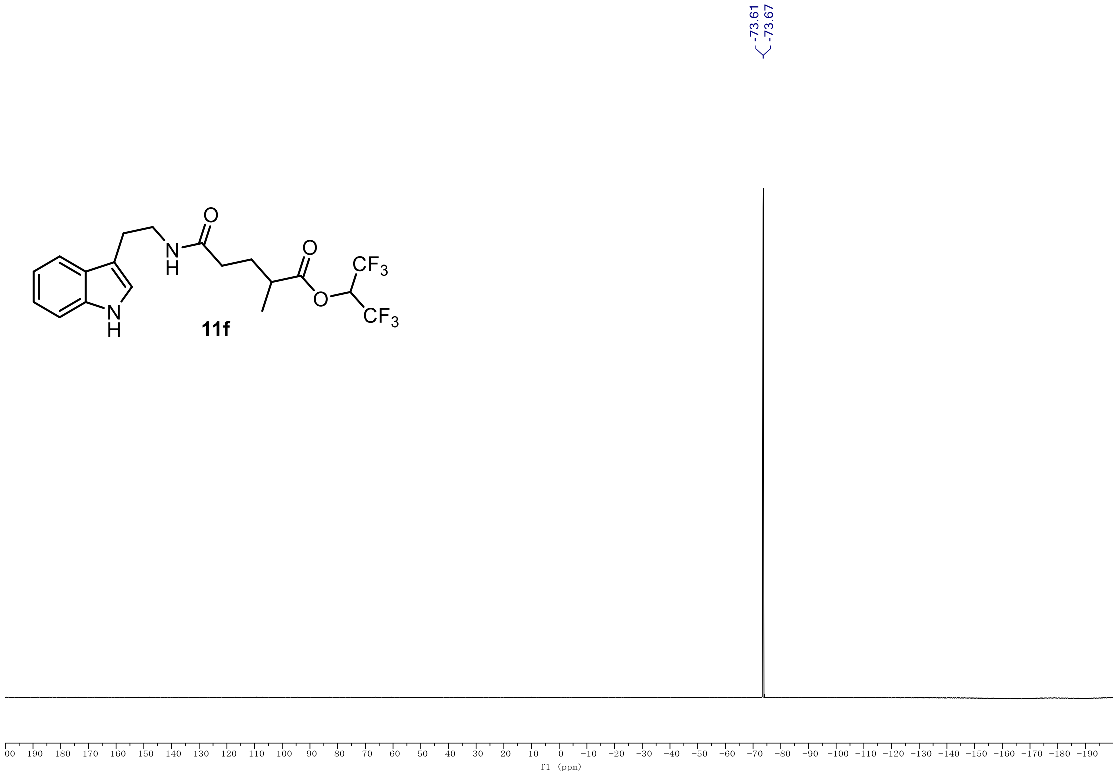

**$^{1}$H NMR** (600 MHz, $CDCl_3$) **11g**.

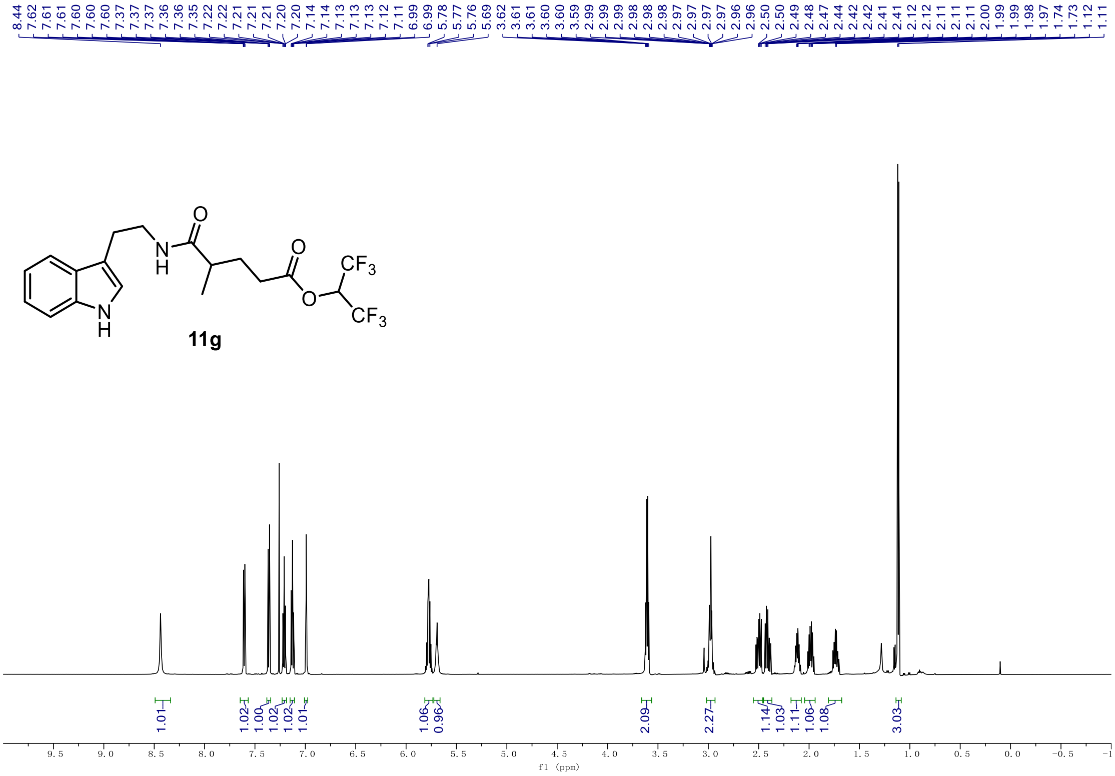


**$^{13}$C NMR** (151 MHz, $CDCl_3$) **11g**.

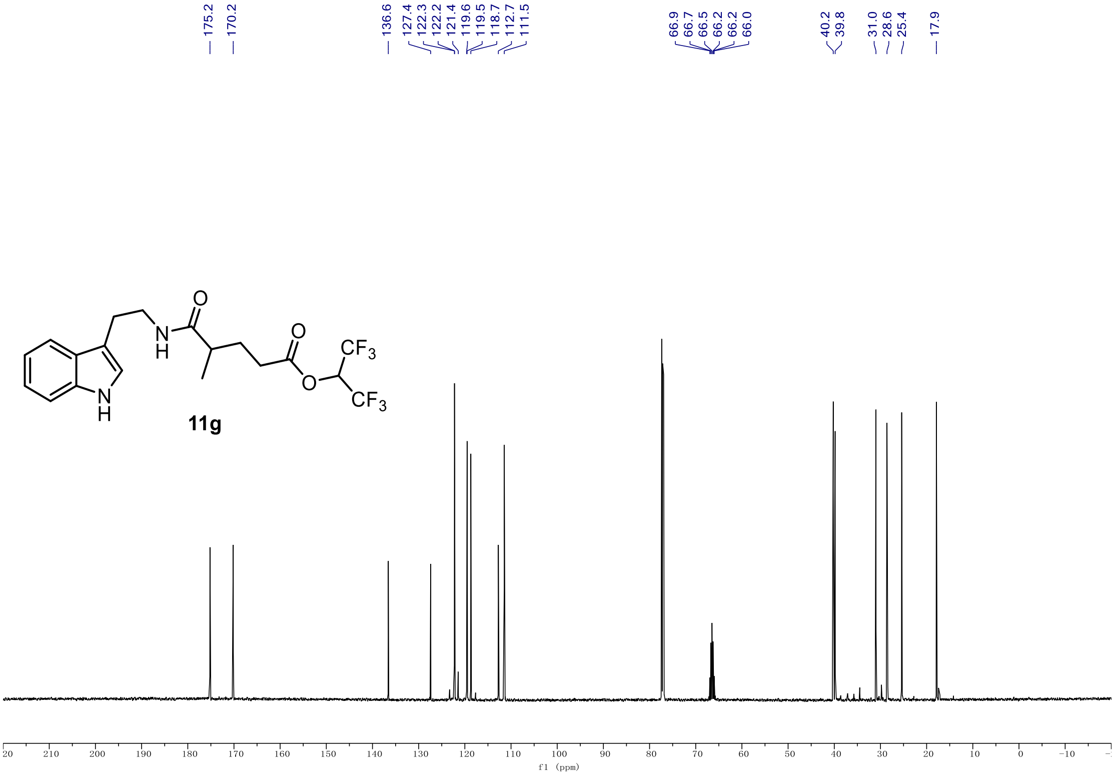

**$^{19}$F NMR** (565 MHz, $CDCl_3$) **11g**.

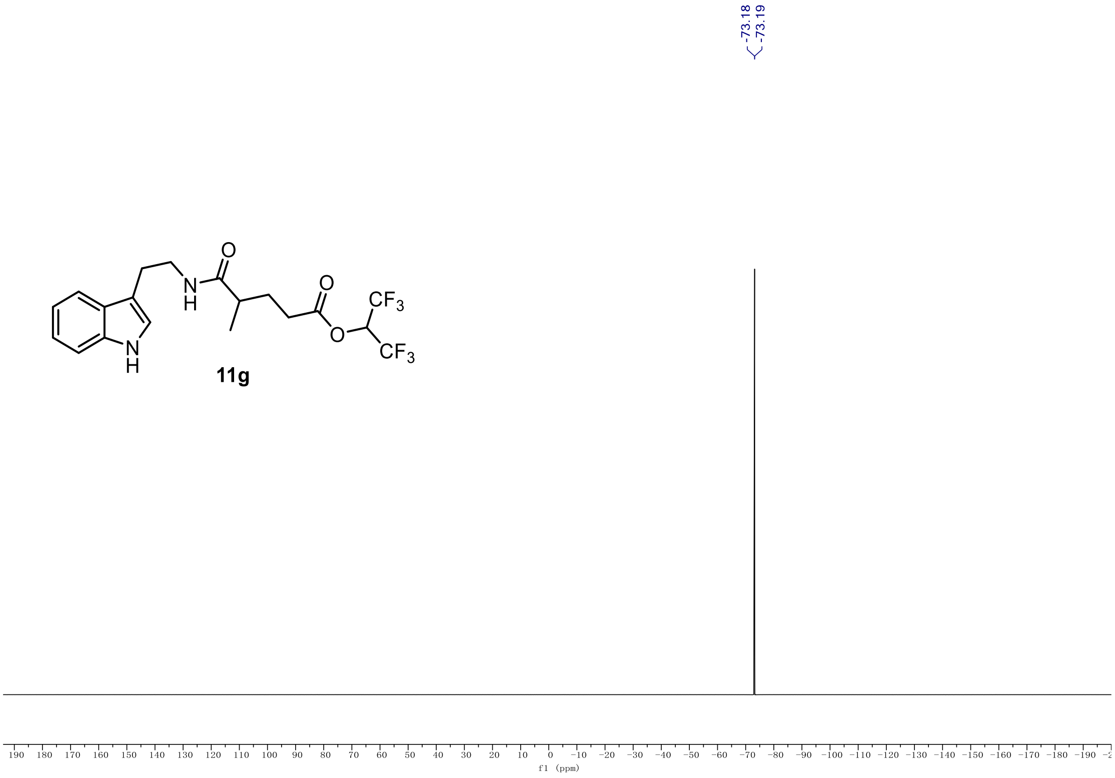


11g

**$^{1}$H NMR** (500 MHz, $CDCl_3$) **11h**.

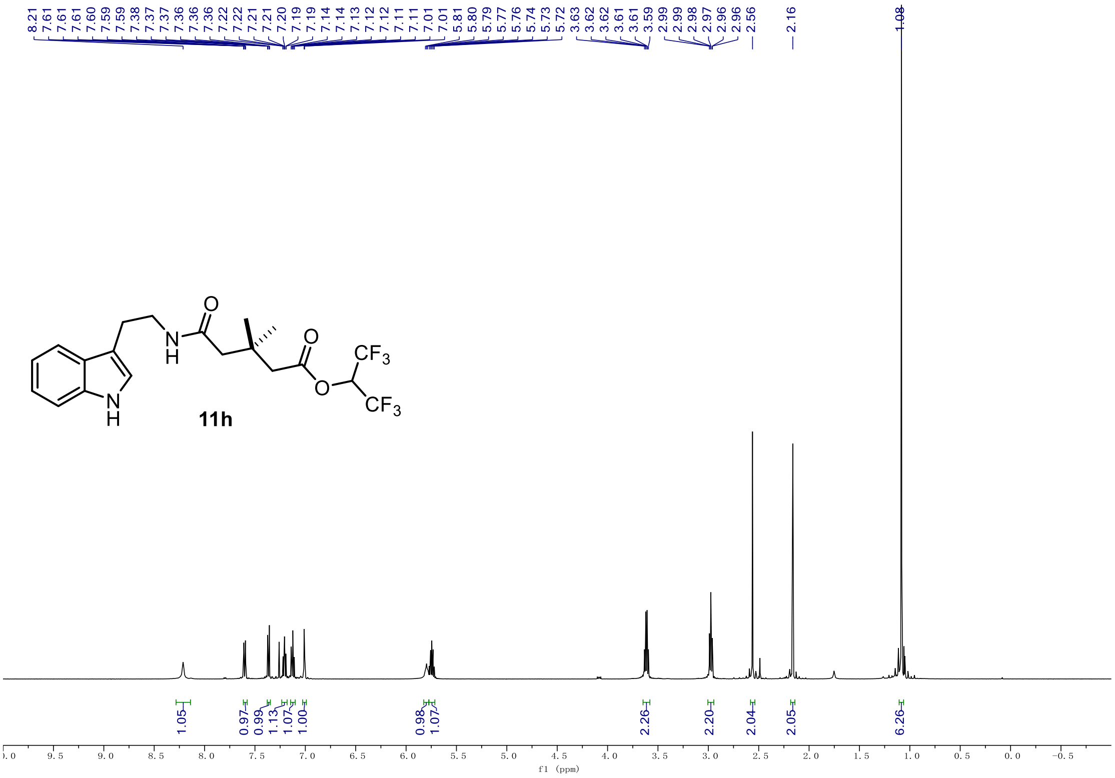


11h

**$^{13}$C NMR** (126 MHz, $CDCl_3$) **11h**.

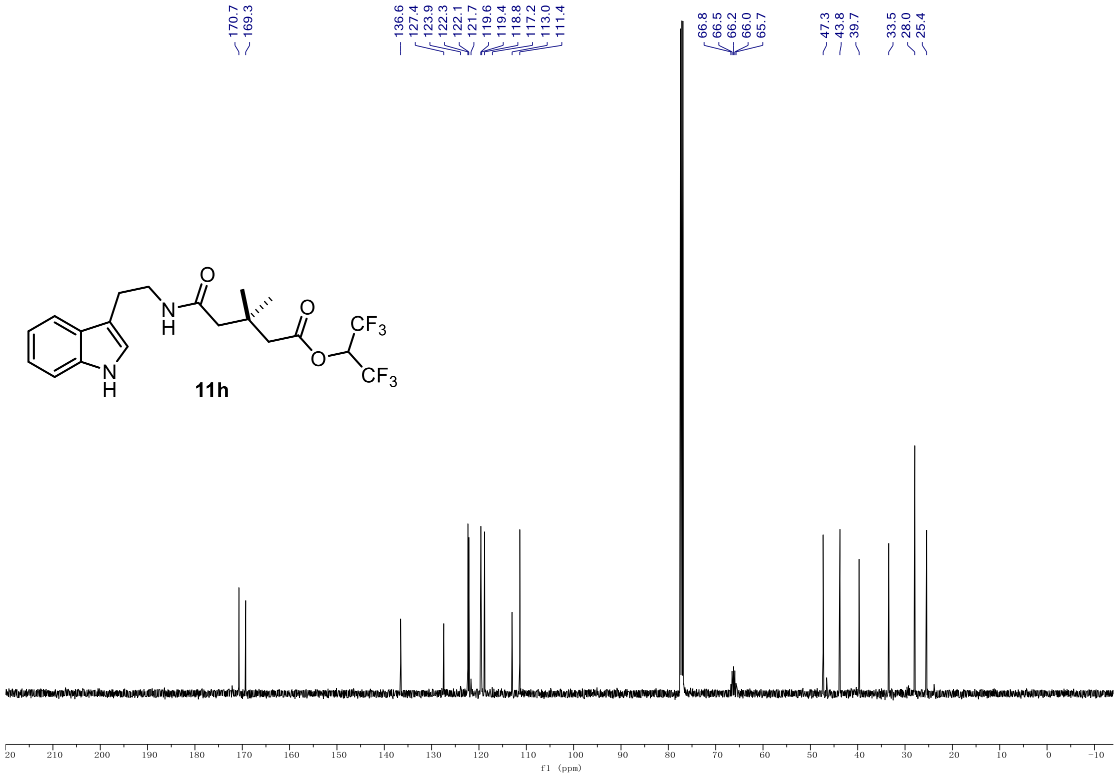


**$^{19}$F NMR** (565 MHz, $CDCl_3$) **11h**.

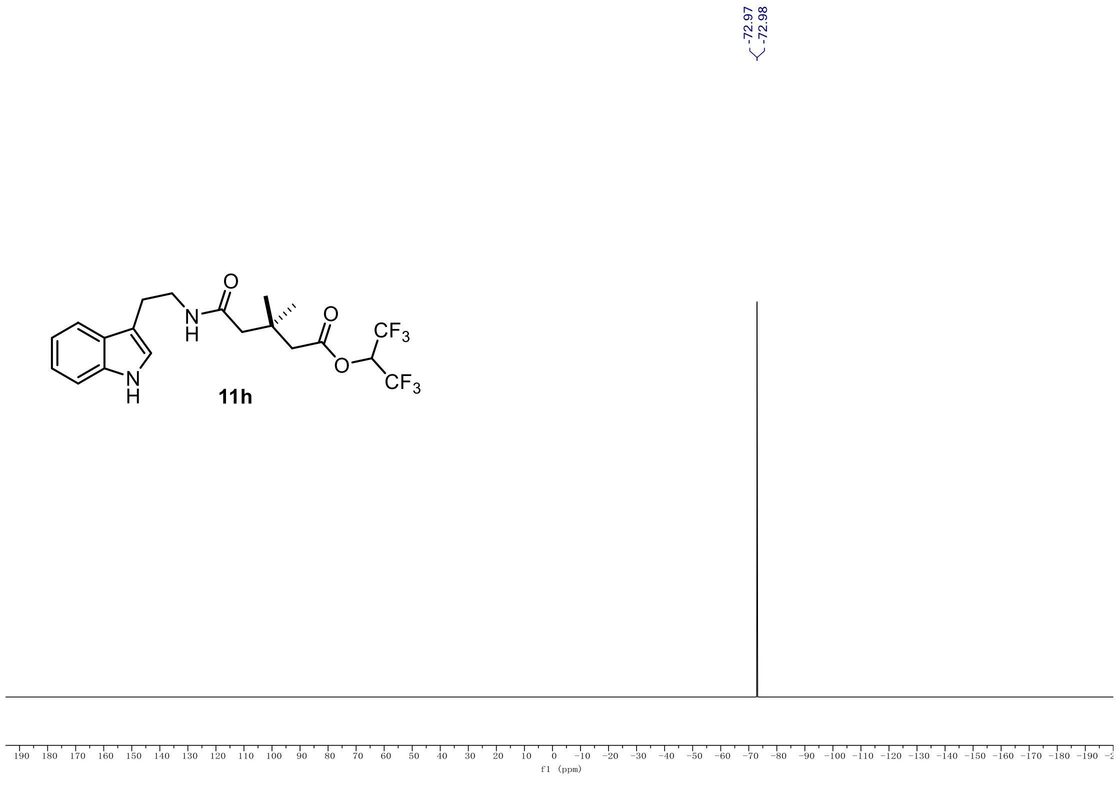

$^{1}$H NMR (500 MHz, $CDCl_3$) **11i**.

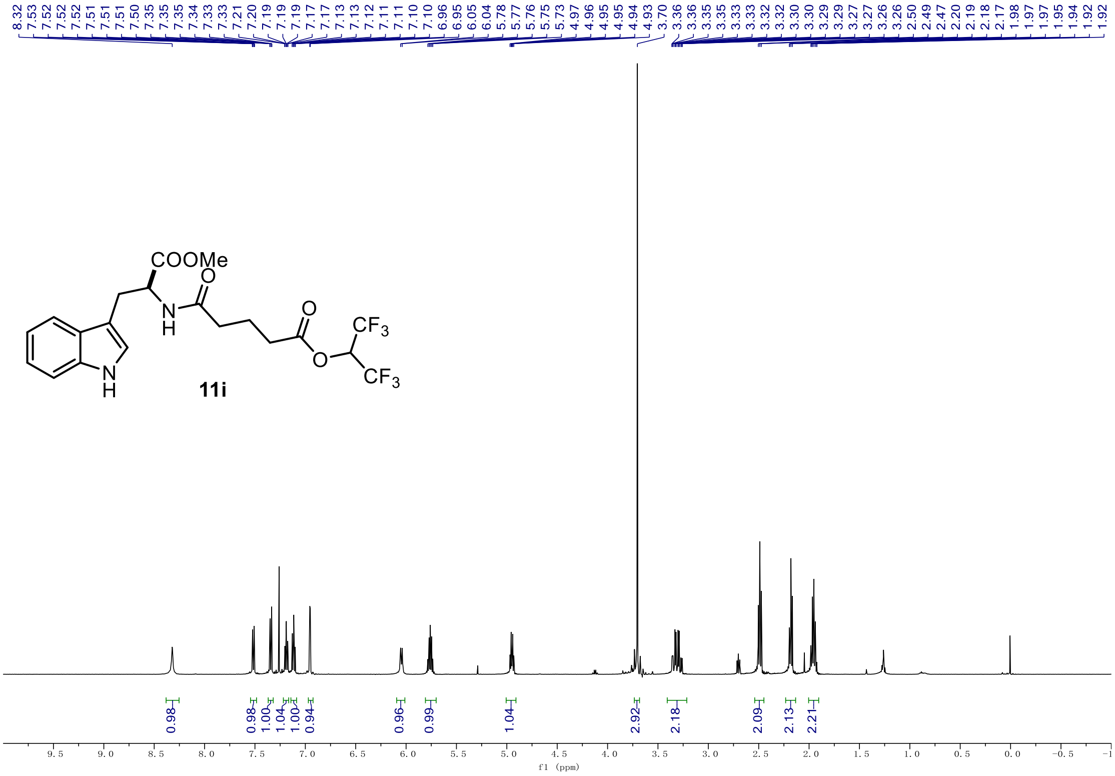


$^{13}$C NMR (126 MHz, $CDCl_3$) **11i**.

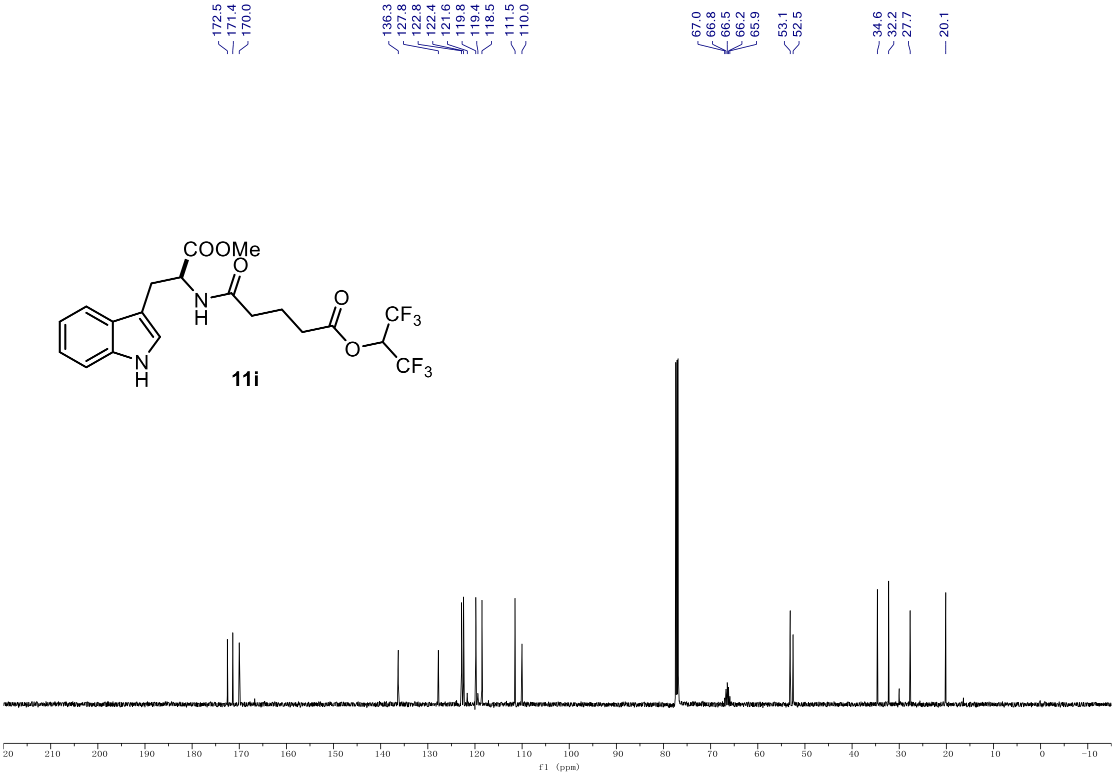

**$^{19}$F NMR** (471 MHz, $CDCl_3$) **11i**.

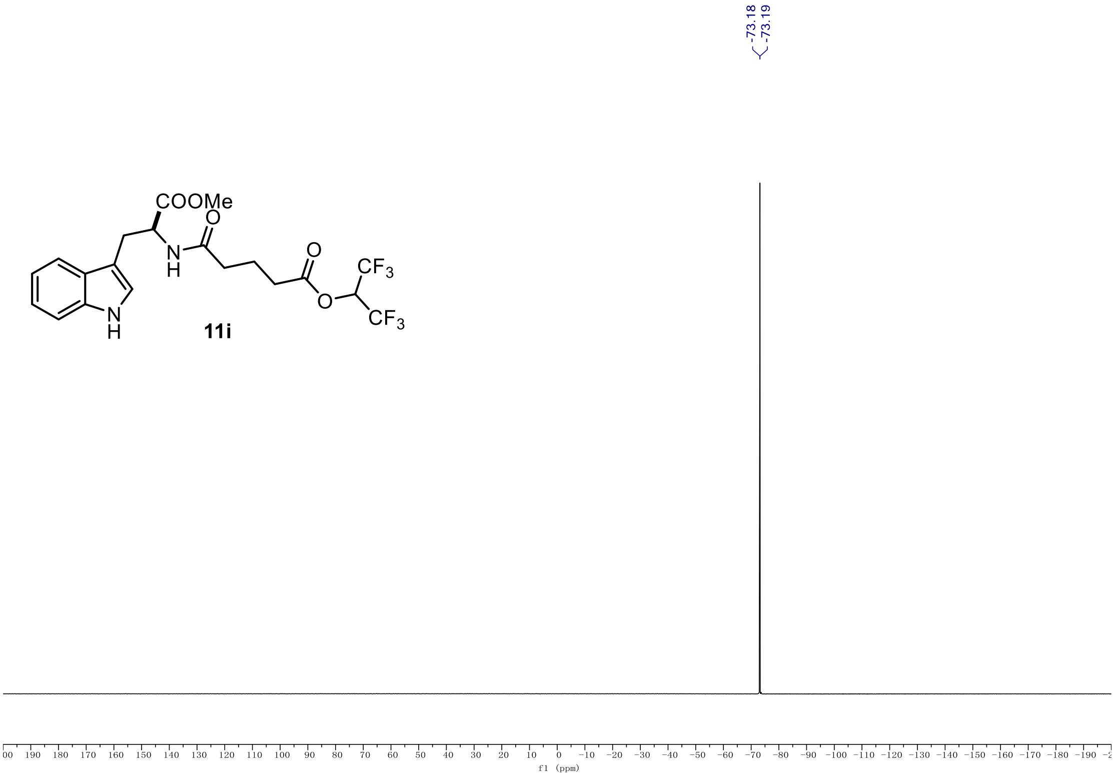


**$^{1}$H NMR** (500 MHz, $CDCl_3$) **11j**.

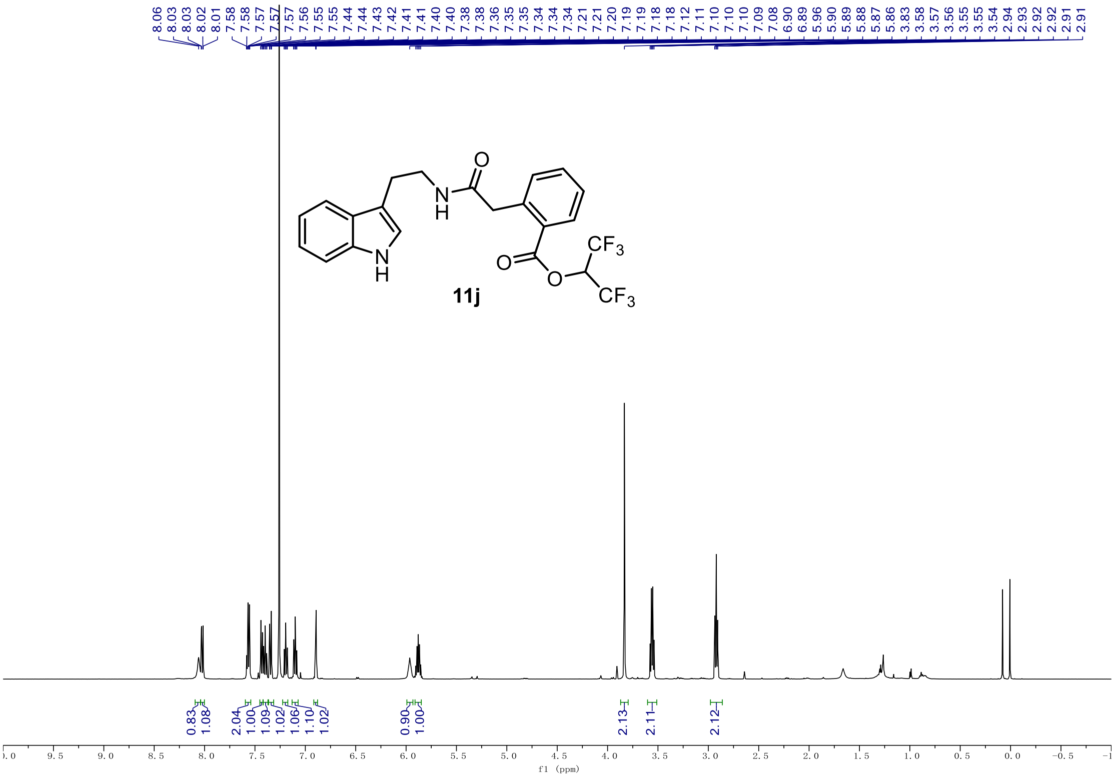

**$^{13}$C NMR** (126 MHz, $CDCl_3$) **11j**.

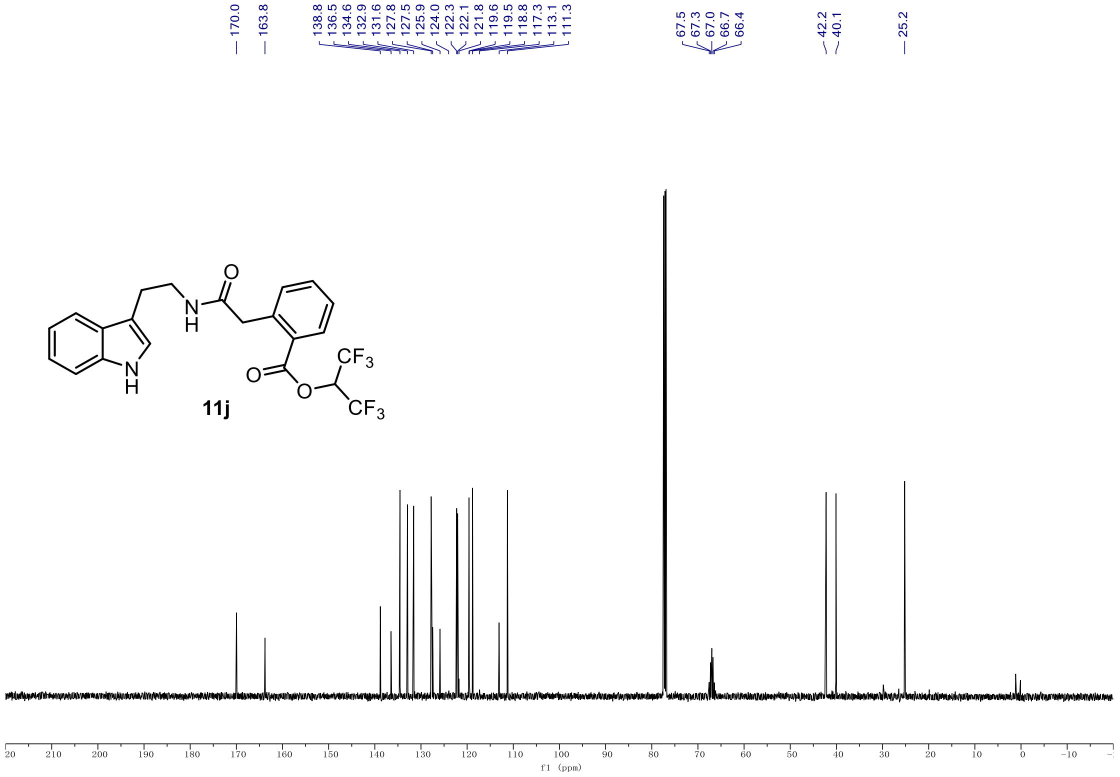


**$^{19}$F NMR** (471 MHz, $CDCl_3$) **11j**.

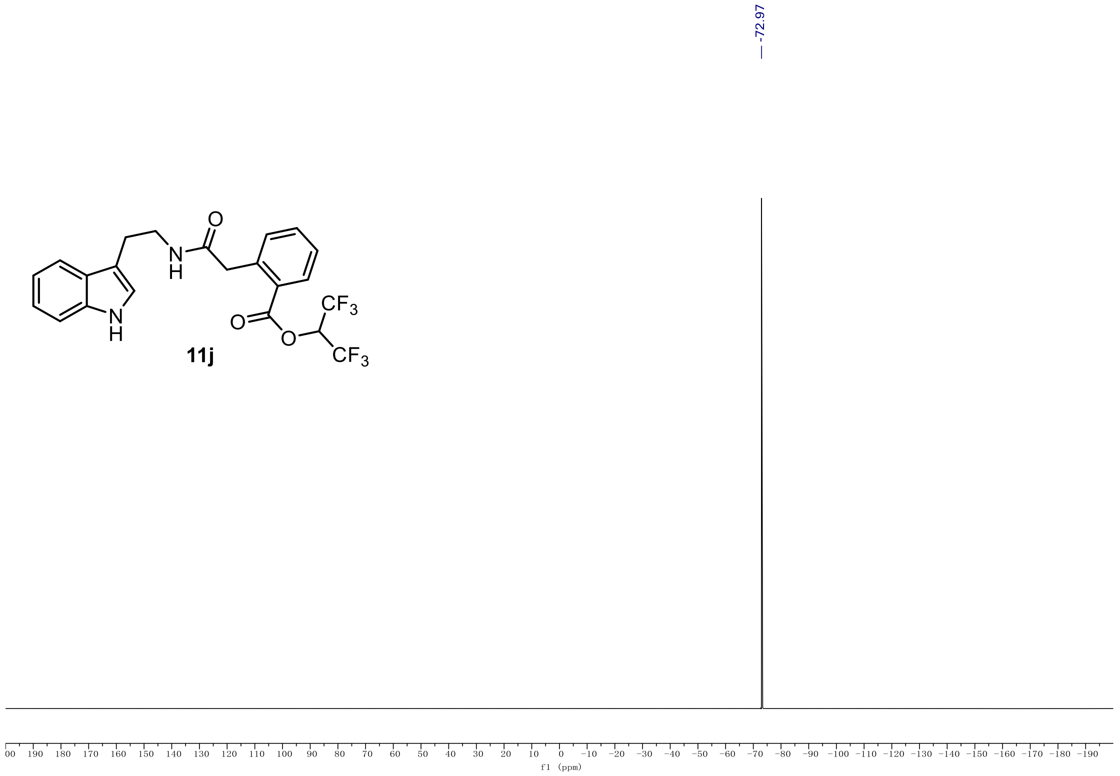

**¹H NMR** (500 MHz, $CDCl_3$) **11k**.

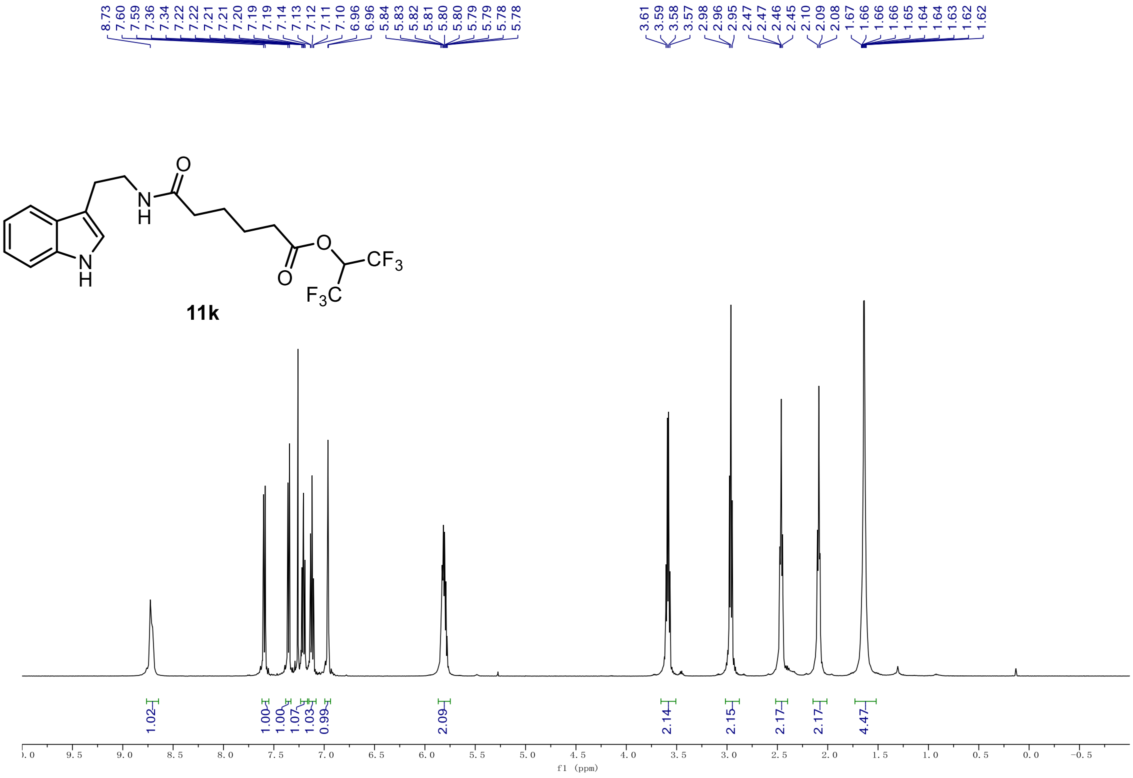


**¹³C NMR** (126 MHz, $CDCl_3$) **11k**.

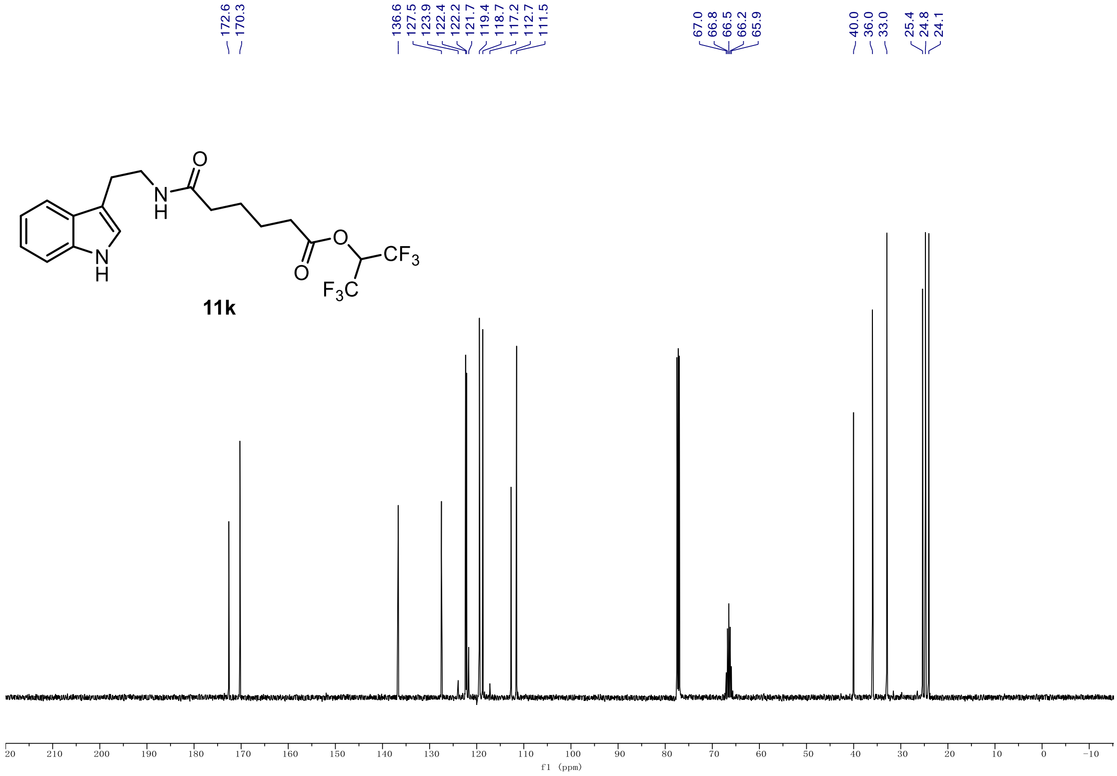

**$^{19}$F NMR** (565 MHz, $CDCl_3$) **11k**.

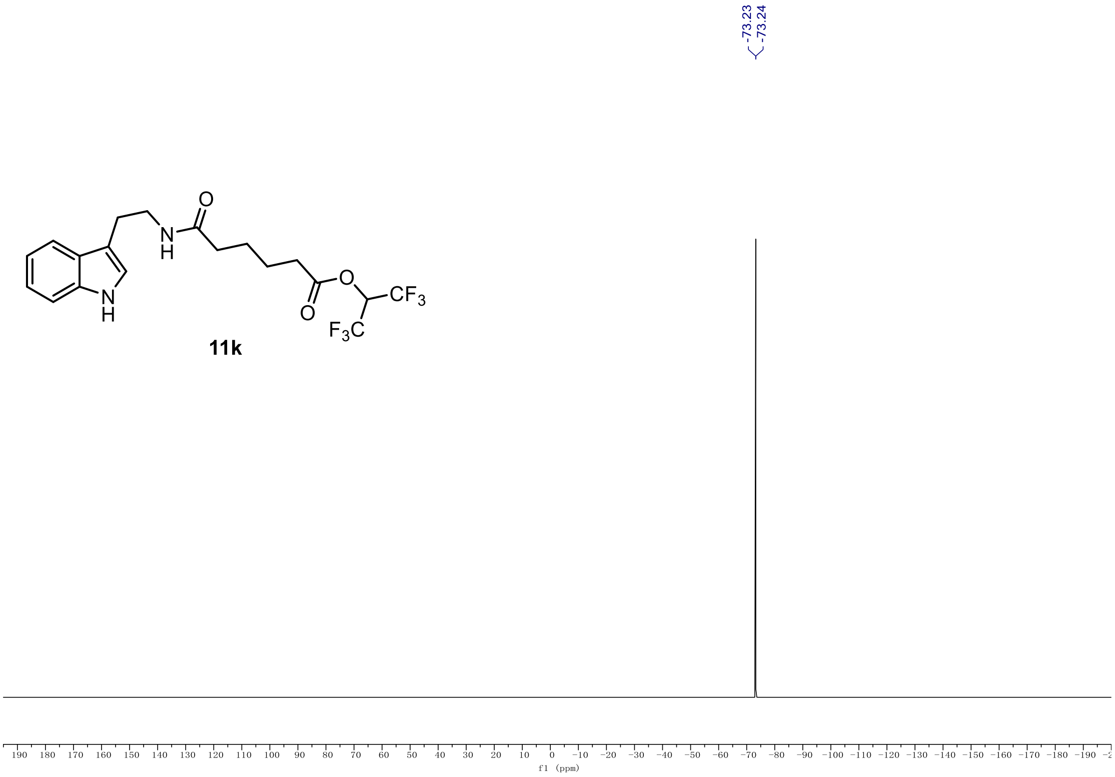


**$^{1}$H NMR** (500 MHz, $CDCl_3$) **11l**.

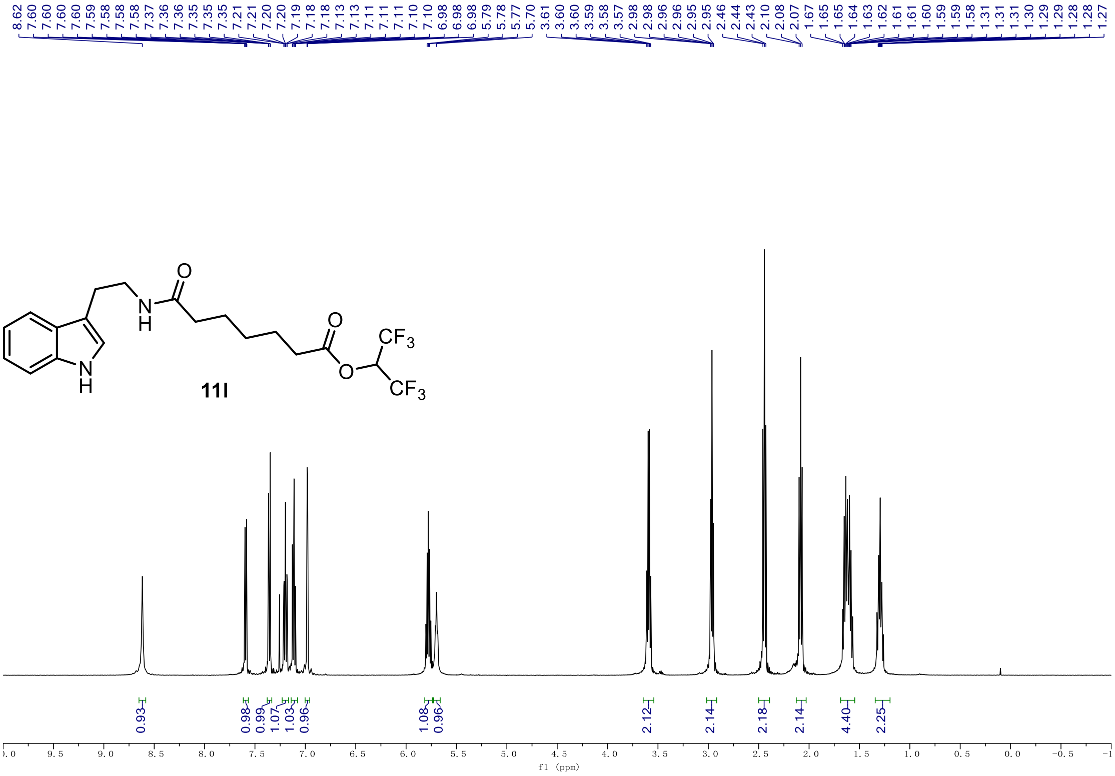

**$^{13}$C NMR** (126 MHz, $CDCl_3$) **11l**.

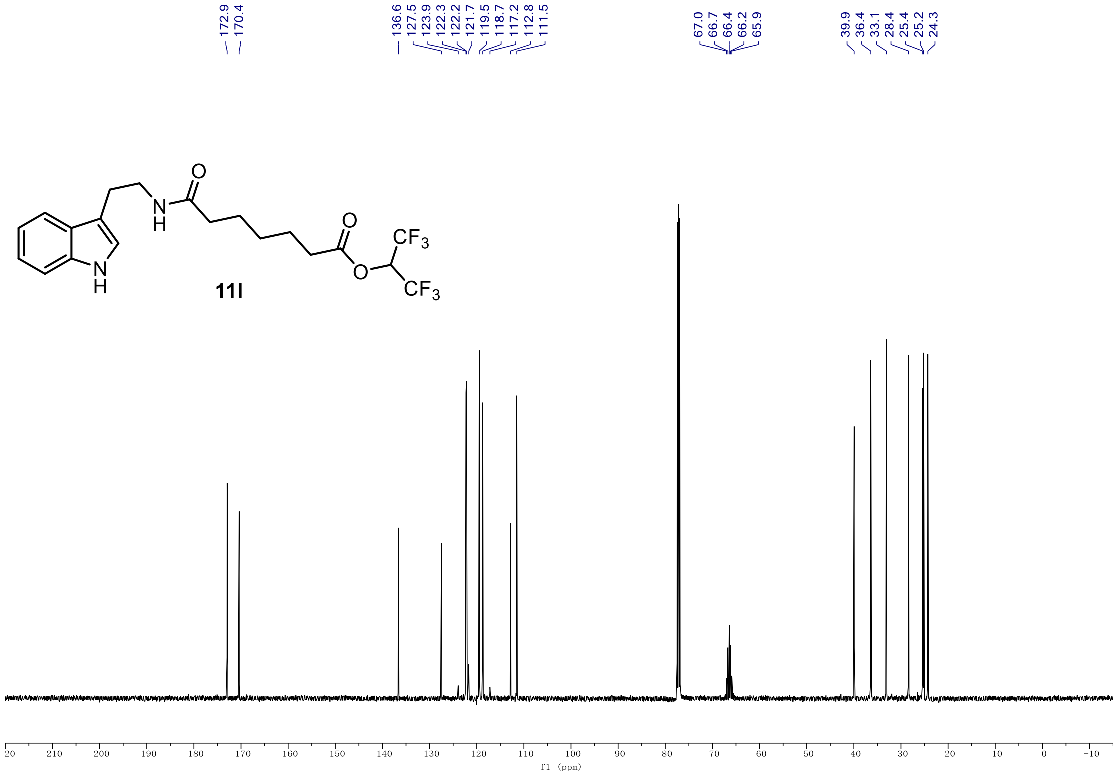


**$^{19}$F NMR** (565 MHz, $CDCl_3$)**11l**.

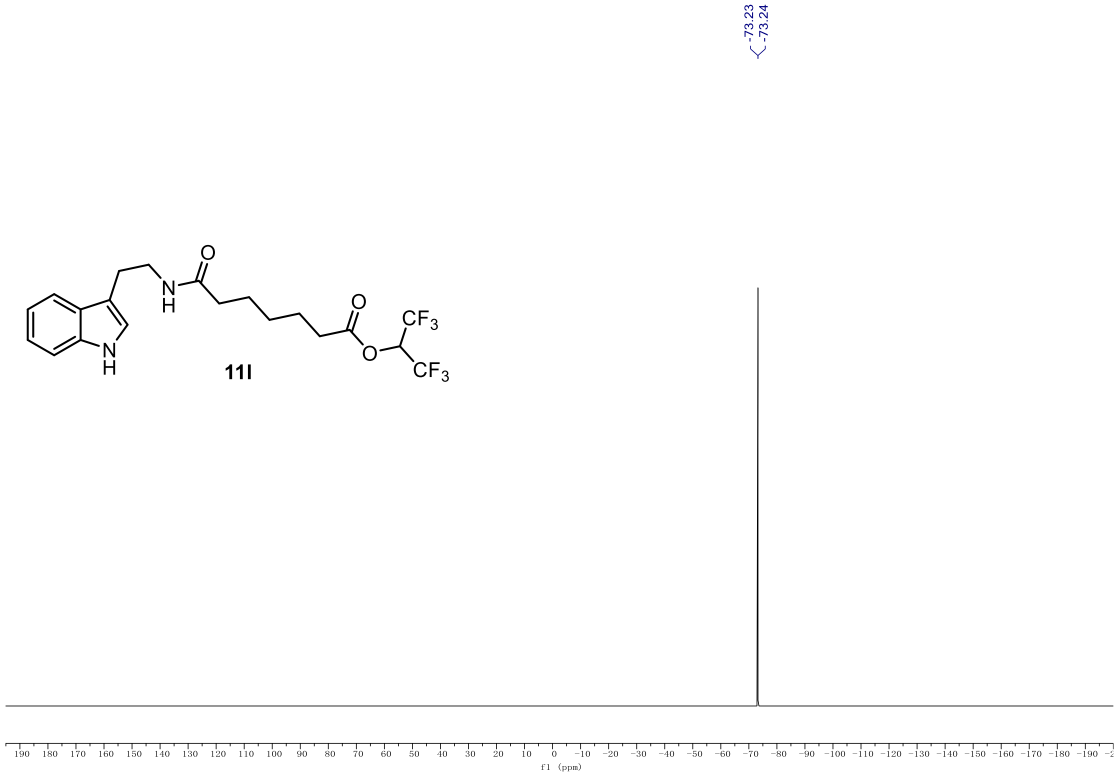

**$^{1}$H NMR** (500 MHz, $CDCl_3$) **11m**.

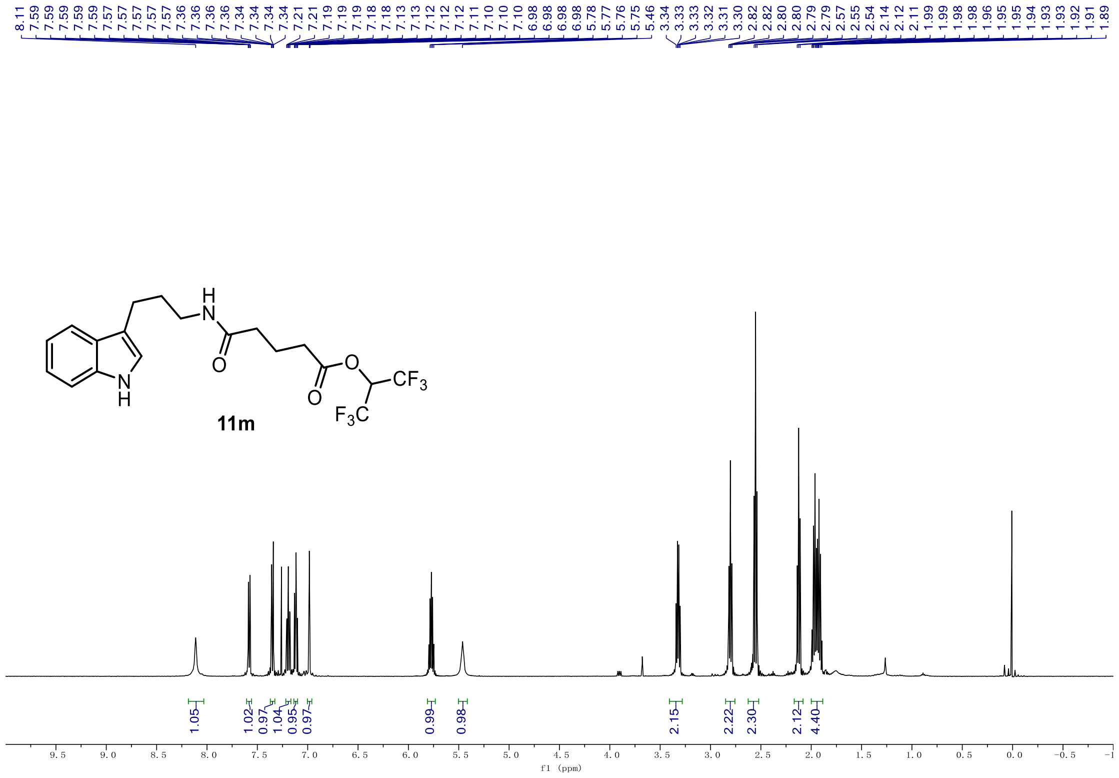

**$^{13}$C NMR** (126 MHz, $CDCl_3$) **11m**.

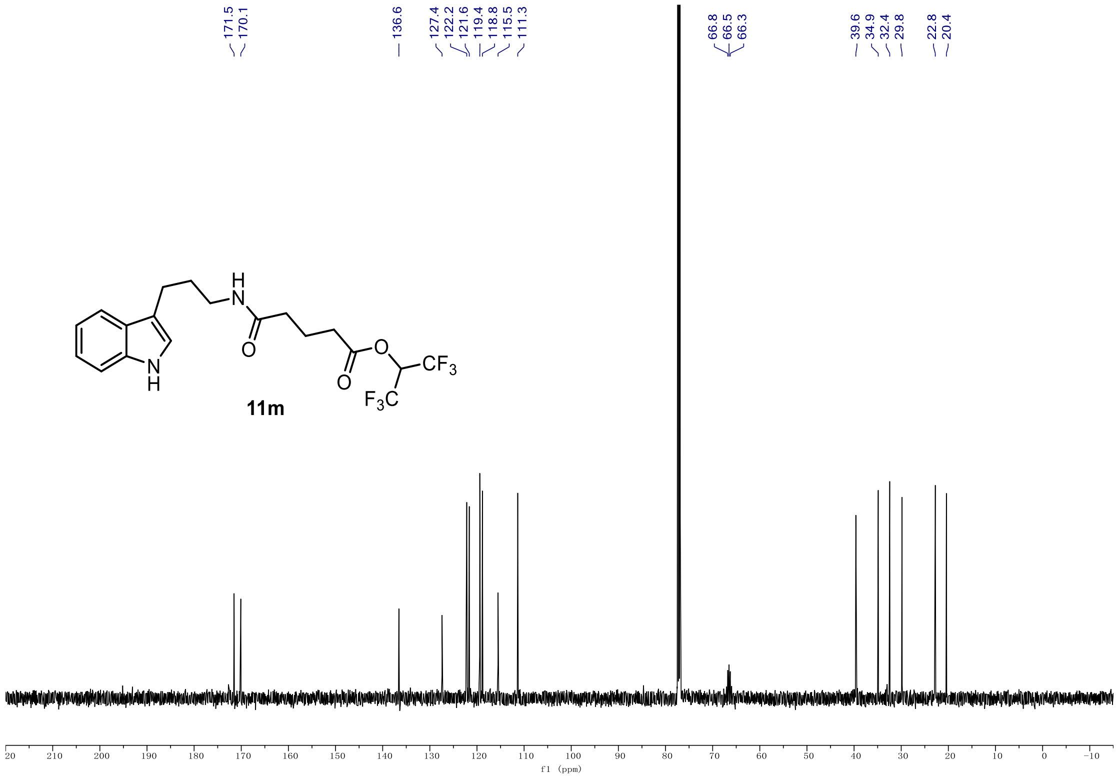

**$^{19}$F NMR** (471 MHz, $CDCl_3$) **11m**.

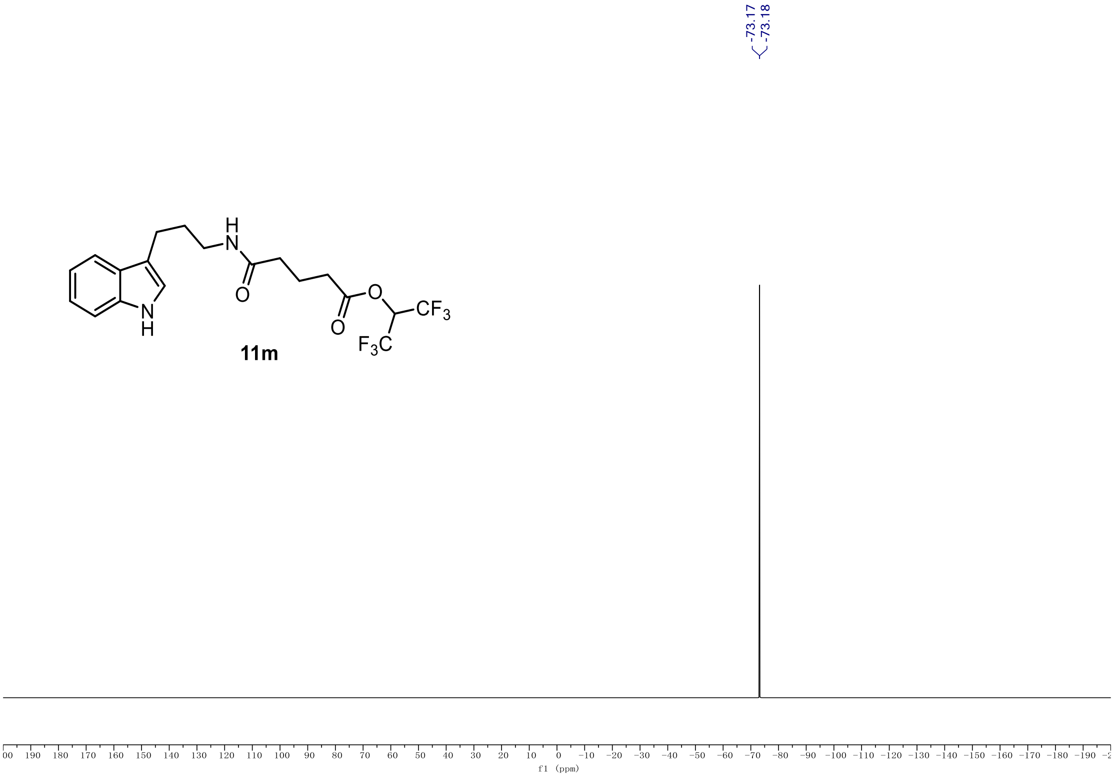


**$^{1}$H NMR** (500 MHz, $CDCl_3$) **11n**.

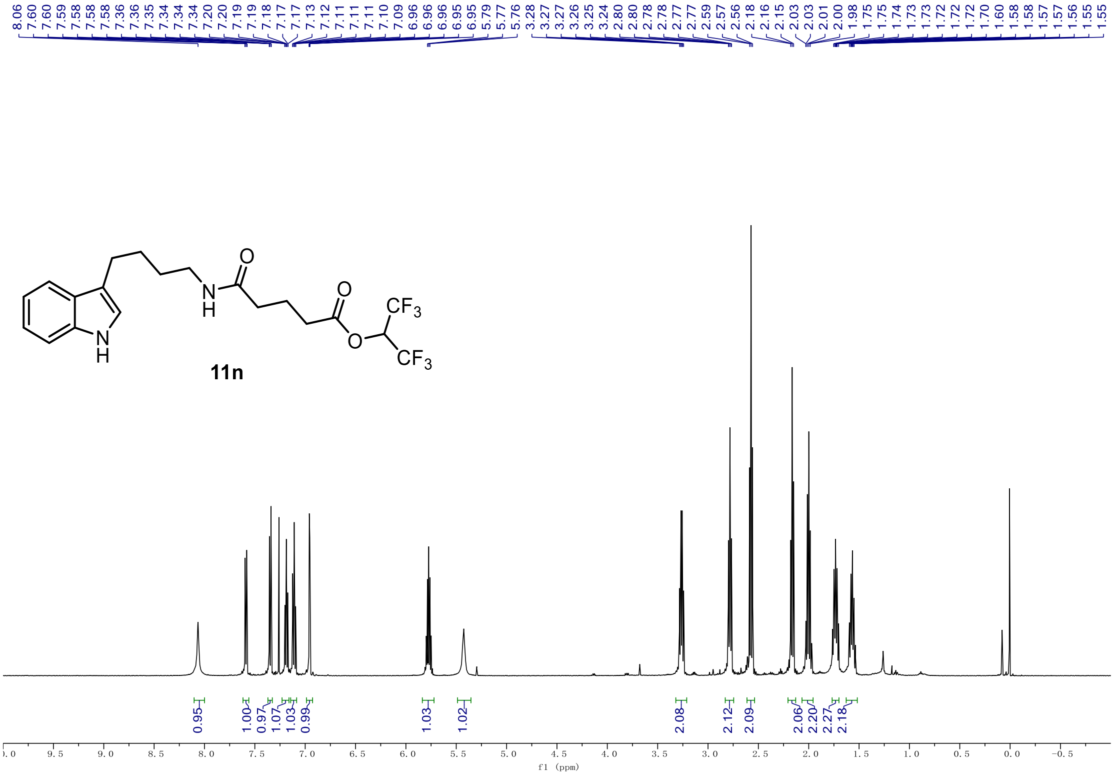

**$^{13}C$ NMR** (126 MHz, $CDCl_3$) **11n**.

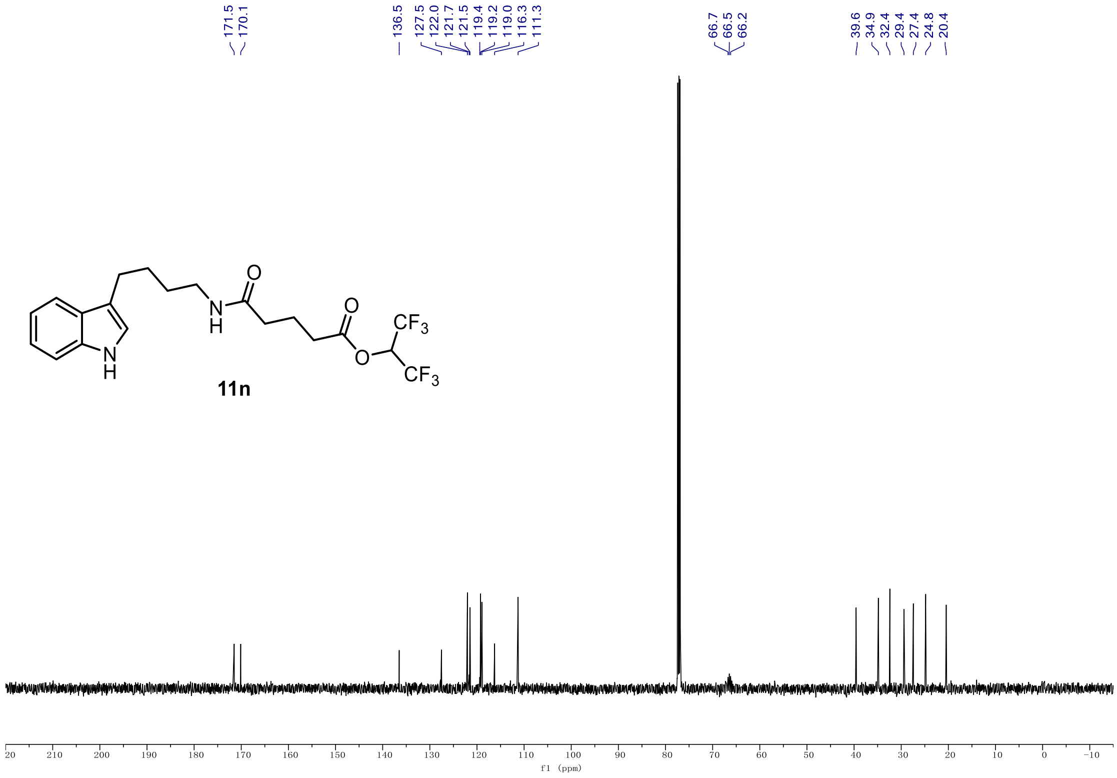


**$^{19}F$ NMR** (471 MHz, $CDCl_3$) **11n**.

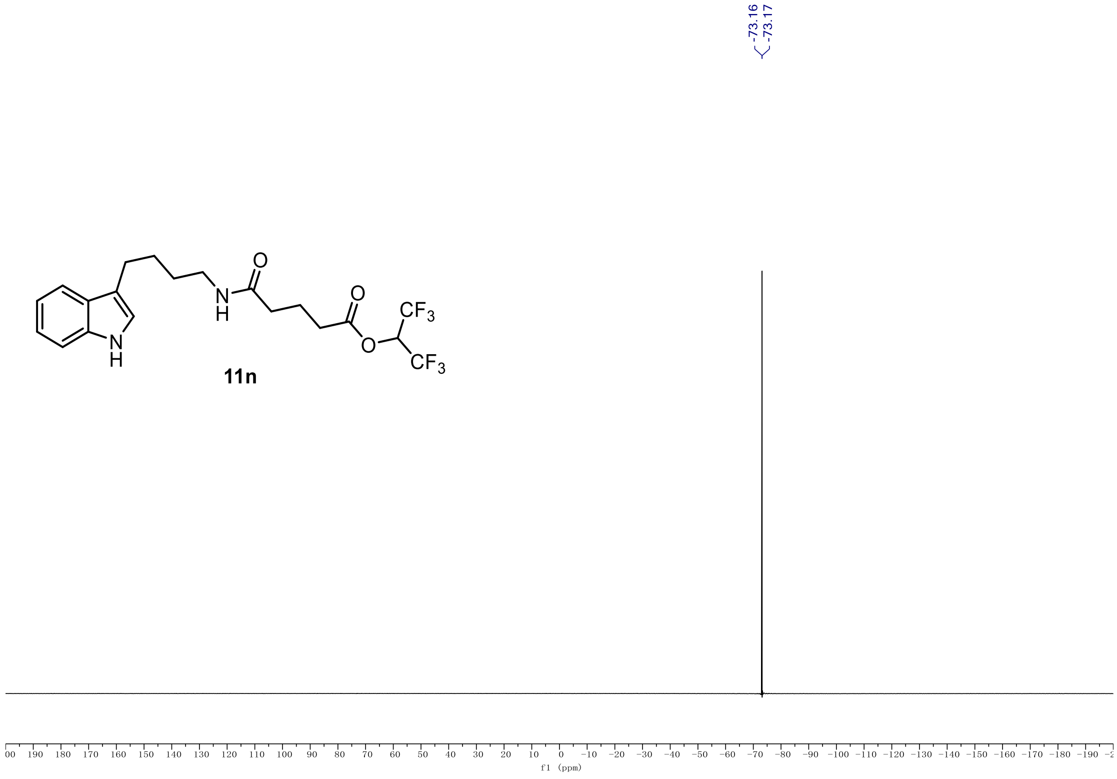

**$^{1}$H NMR** (500 MHz, $CDCl_3$) **11o**.

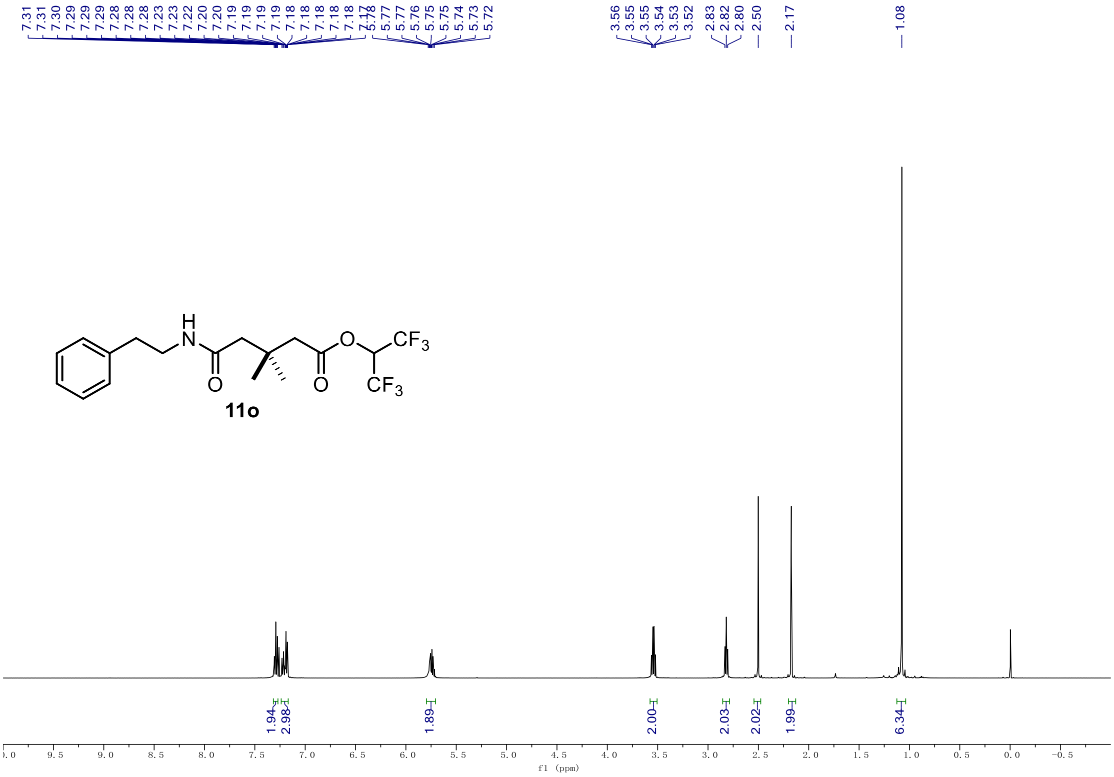


**$^{13}$C NMR** (126 MHz, $CDCl_3$) **11o**.

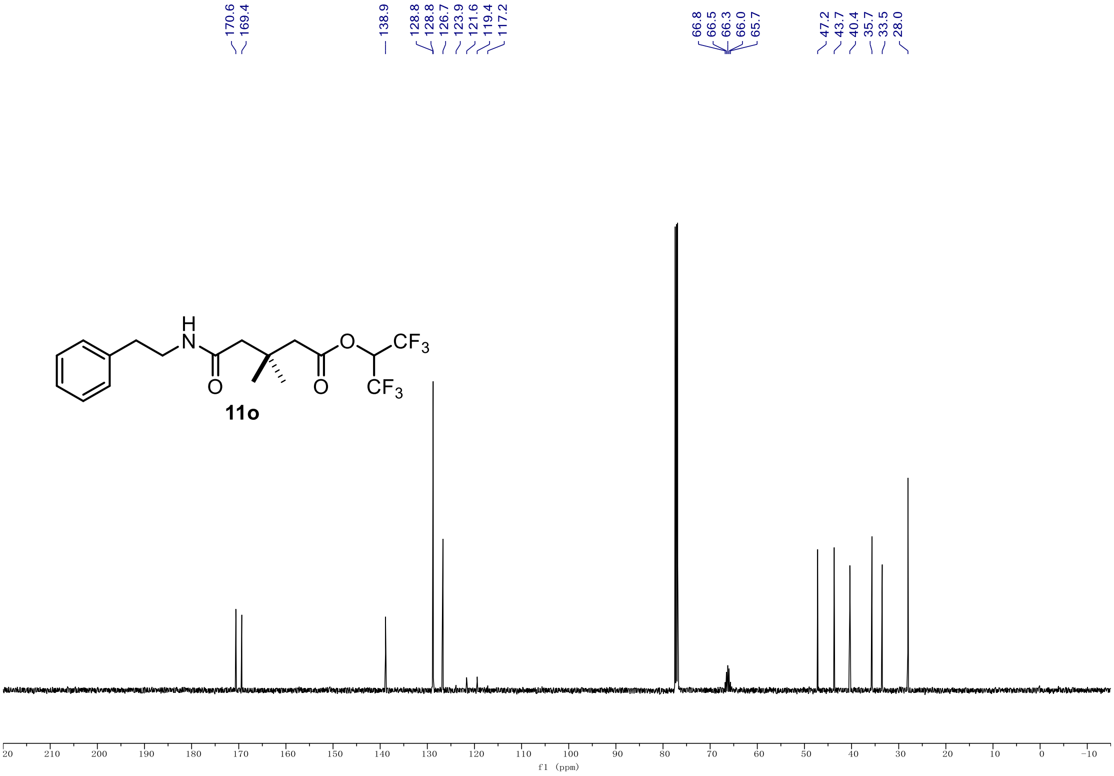

**$^{19}$F NMR** (471 MHz, $CDCl_3$) **11o**.

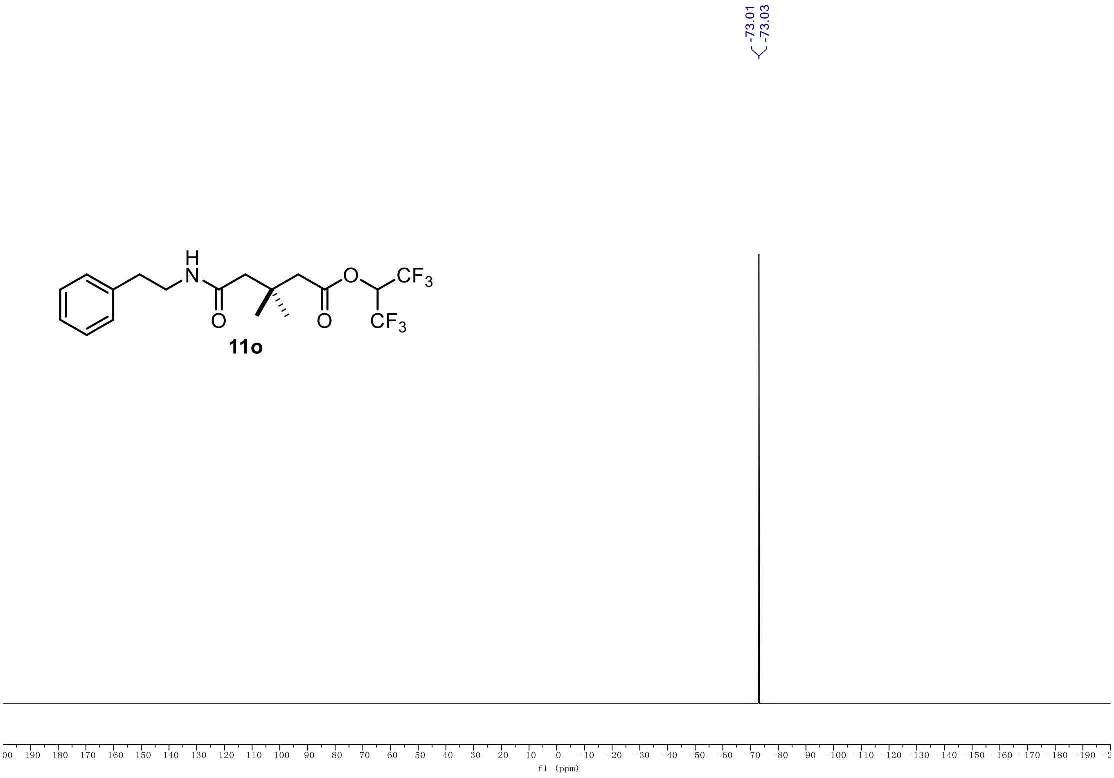


**$^{1}$H NMR** (500 MHz, $CDCl_3$) **11p**.

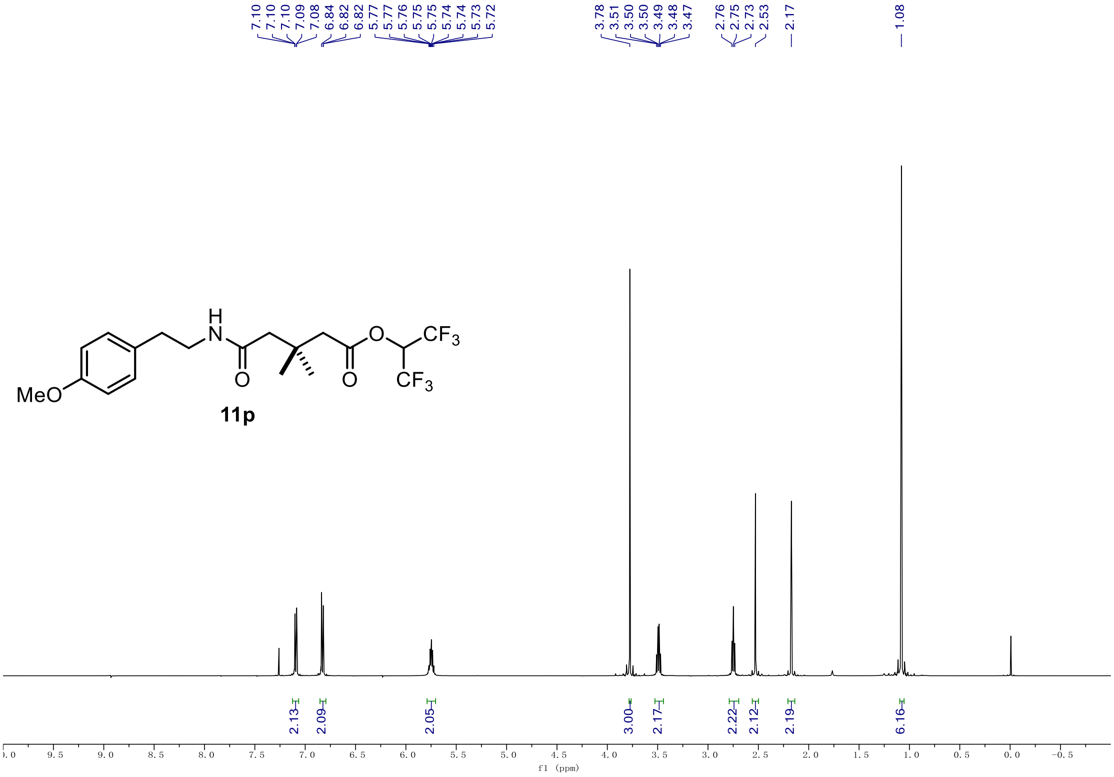

**$^{13}$C NMR** (126 MHz, $CDCl_3$) **11p**.

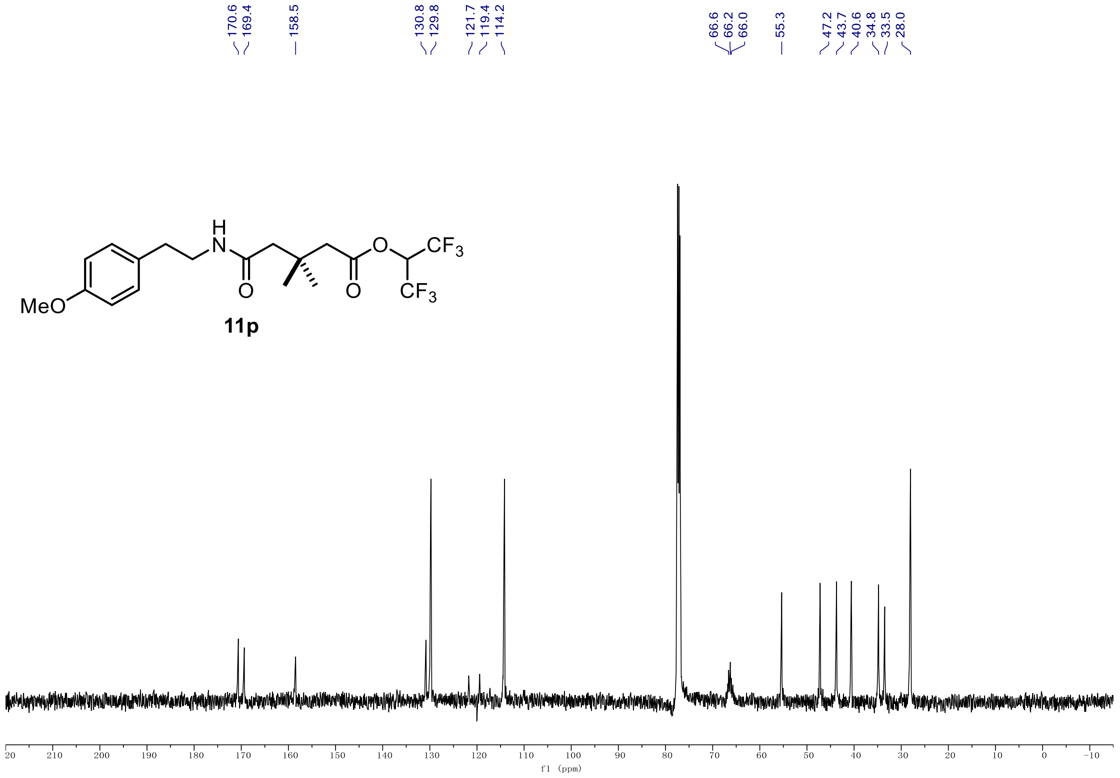


**$^{19}$F NMR** (471 MHz, $CDCl_3$) **11p**.

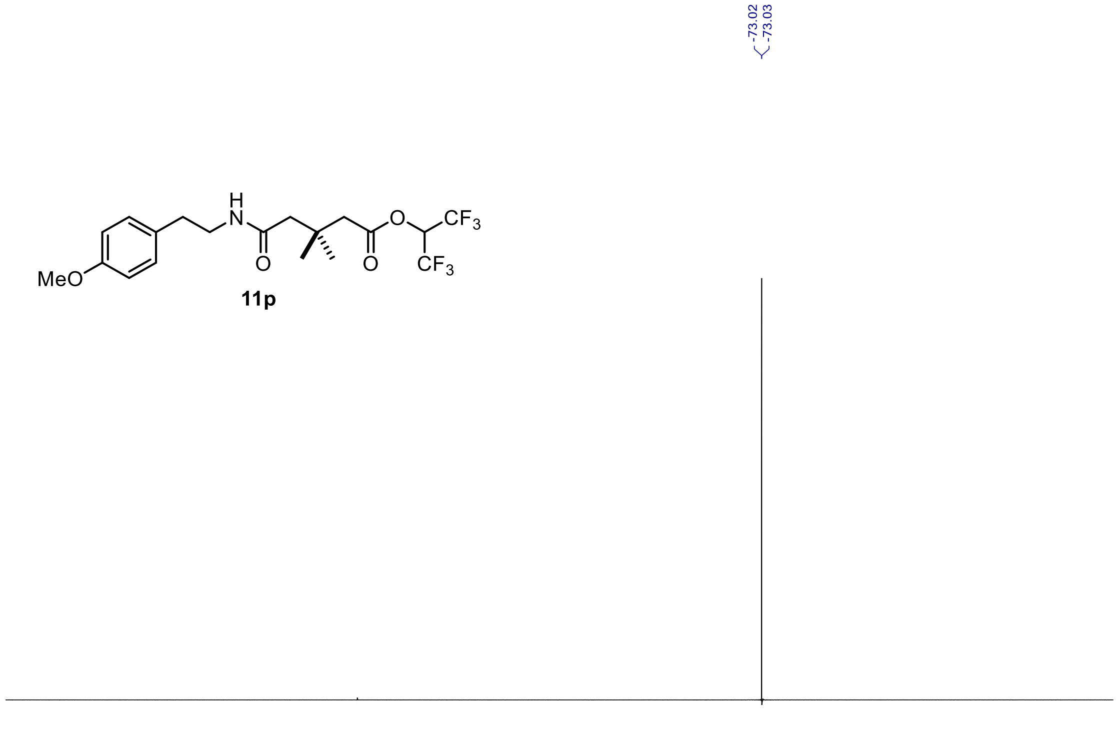

**$^{1}$H NMR** (500 MHz, $CDCl_3$) **11q**.

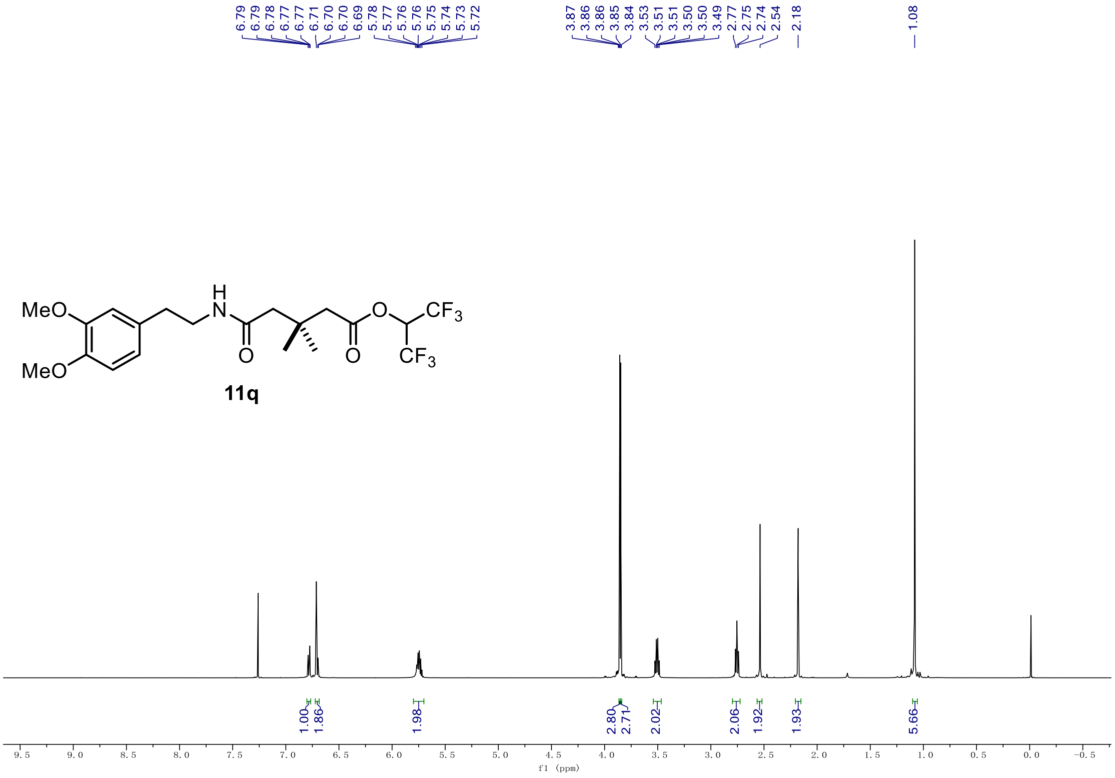


**$^{13}$C NMR** (126 MHz, $CDCl_3$) **11q**.

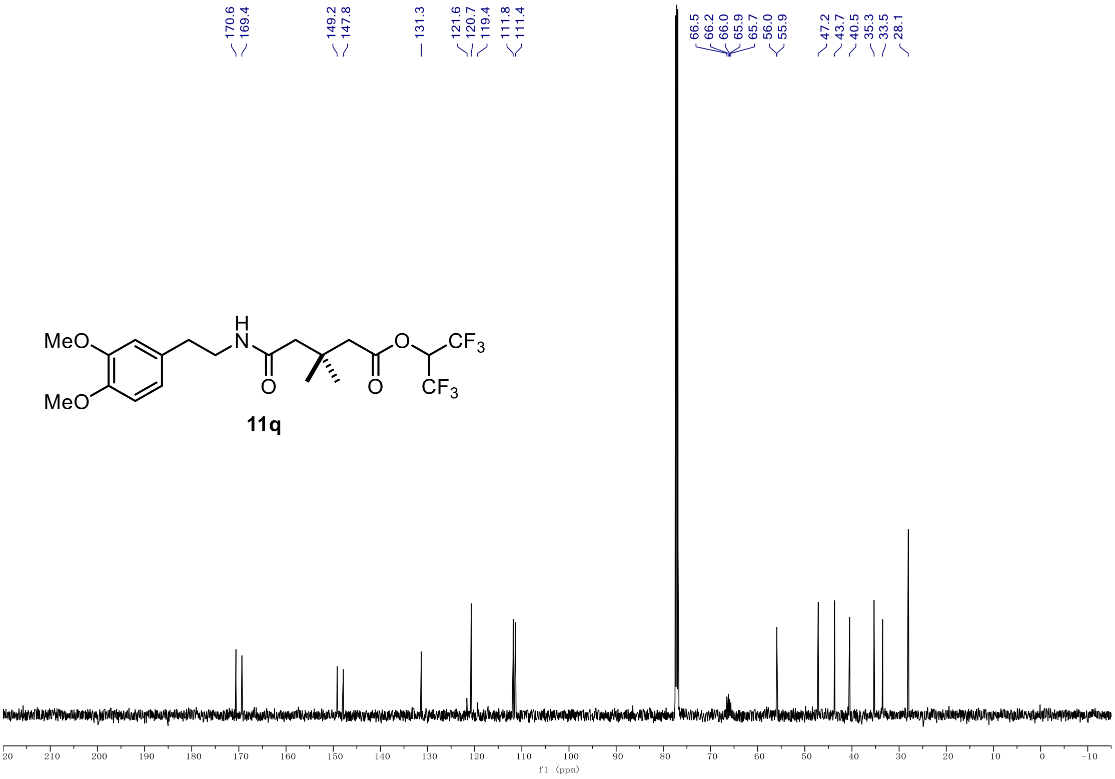

## $^{19}$F NMR (471 MHz, $CDCl_3$) 11q.

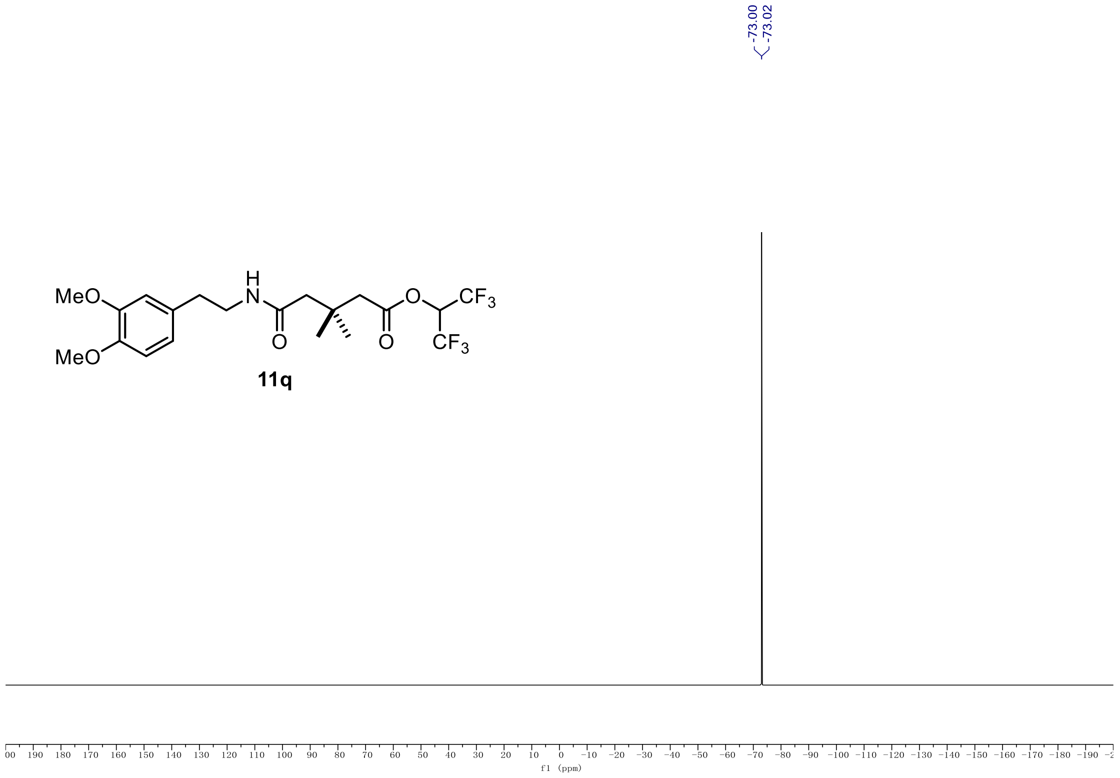


## $^{1}$H NMR (500 MHz, $CDCl_3$) 11r.

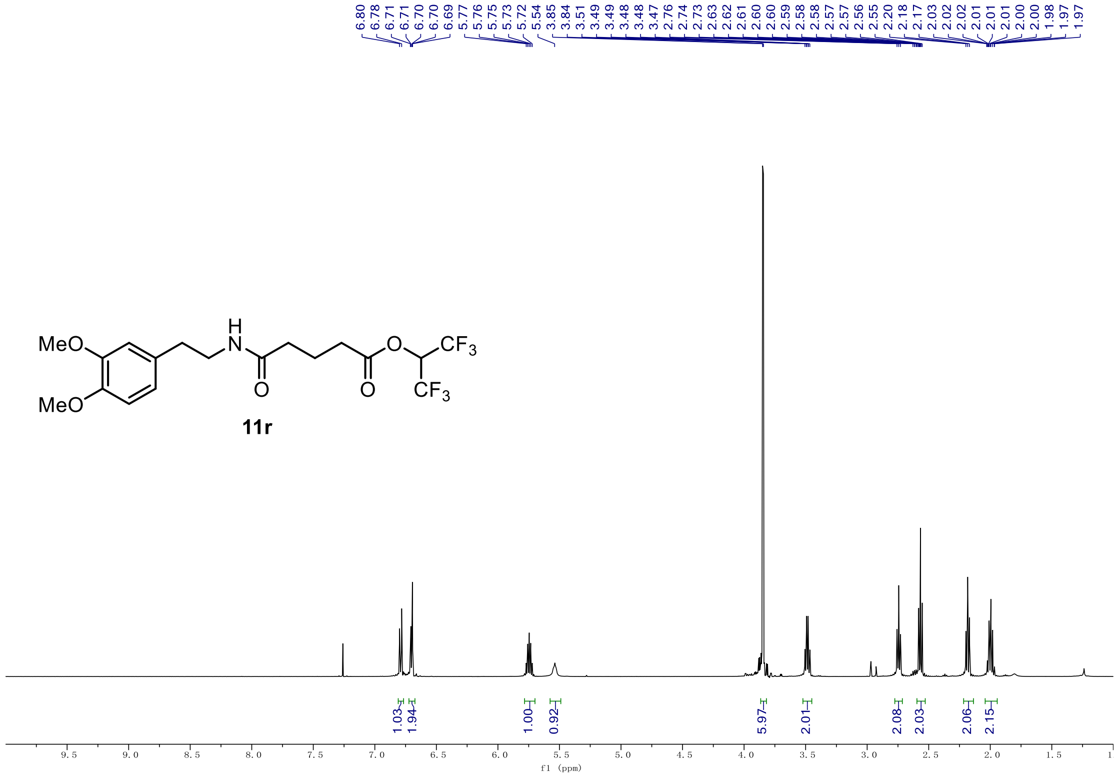

**$^{13}$C NMR** (126 MHz, $CDCl_3$) **11r**.

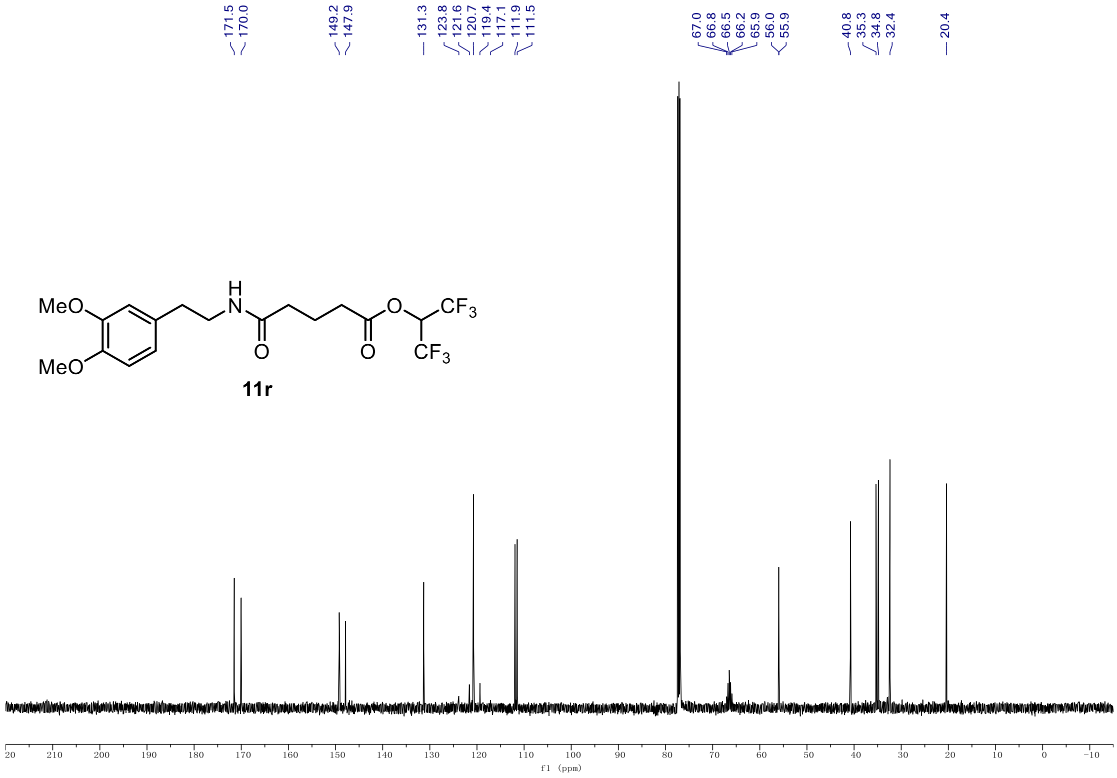


**$^{19}$F NMR** (565 MHz, $CDCl_3$) **11r**.

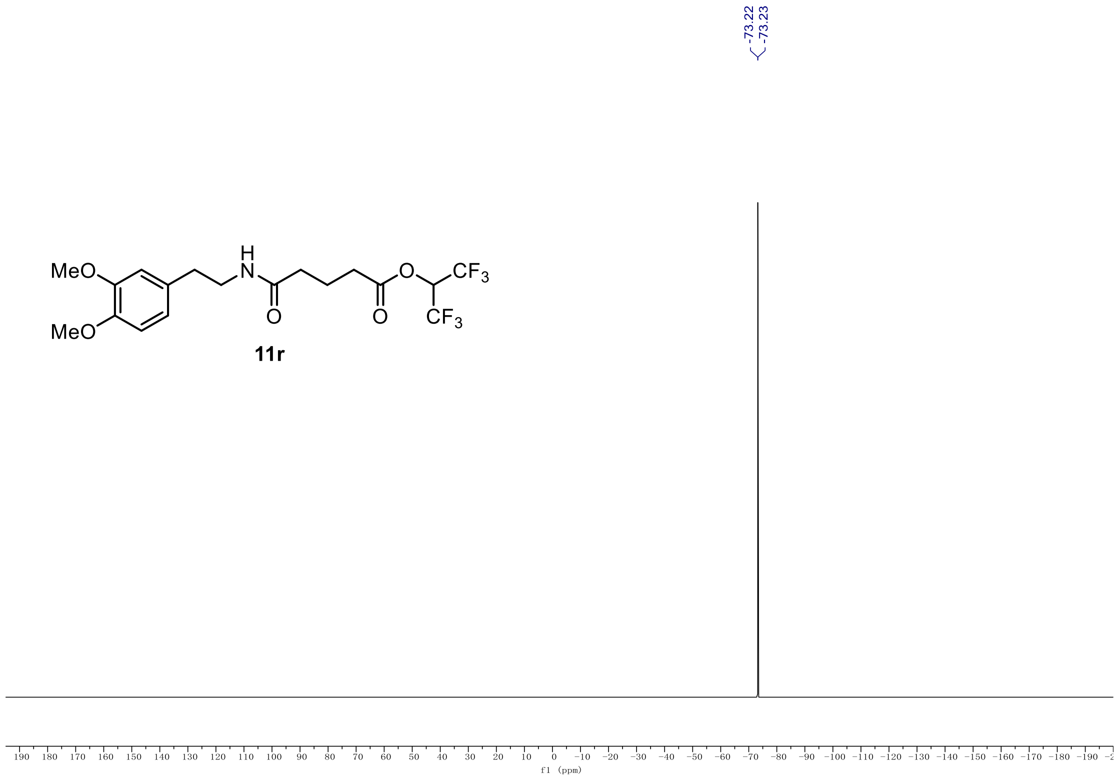

**$^{1}$H NMR** (600 MHz, $CDCl_3$) **11s**.

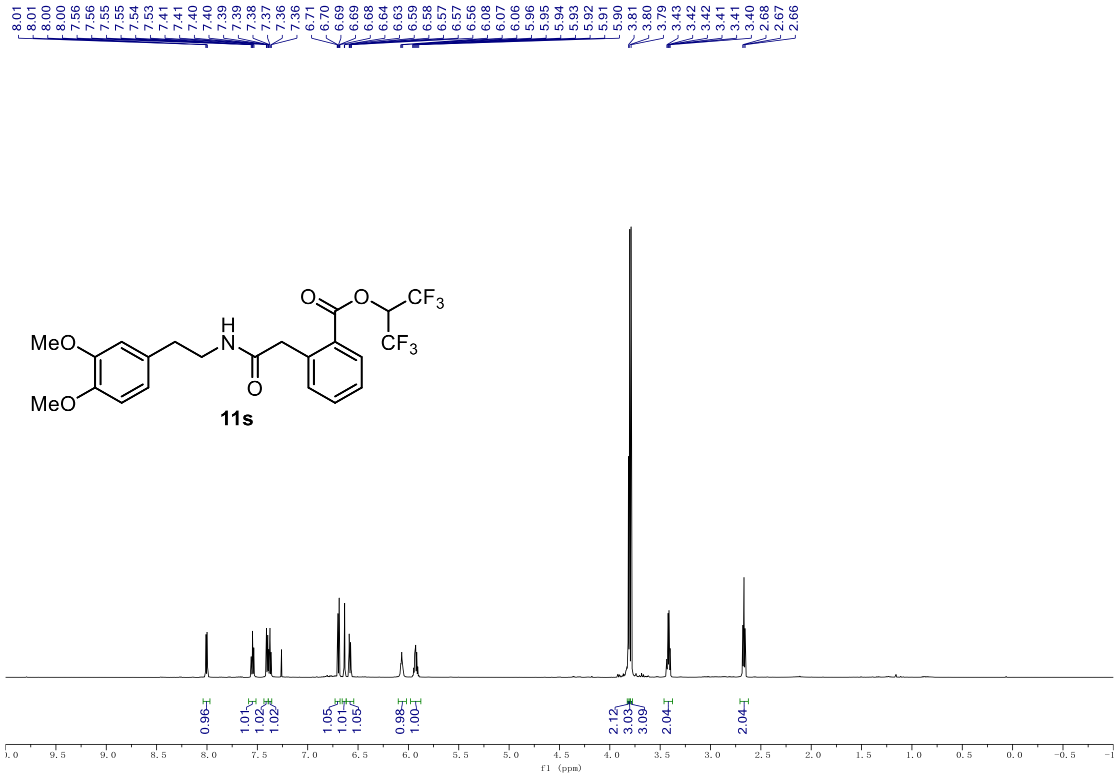

**$^{13}$C NMR** (151 MHz, $CDCl_3$) **11s**.

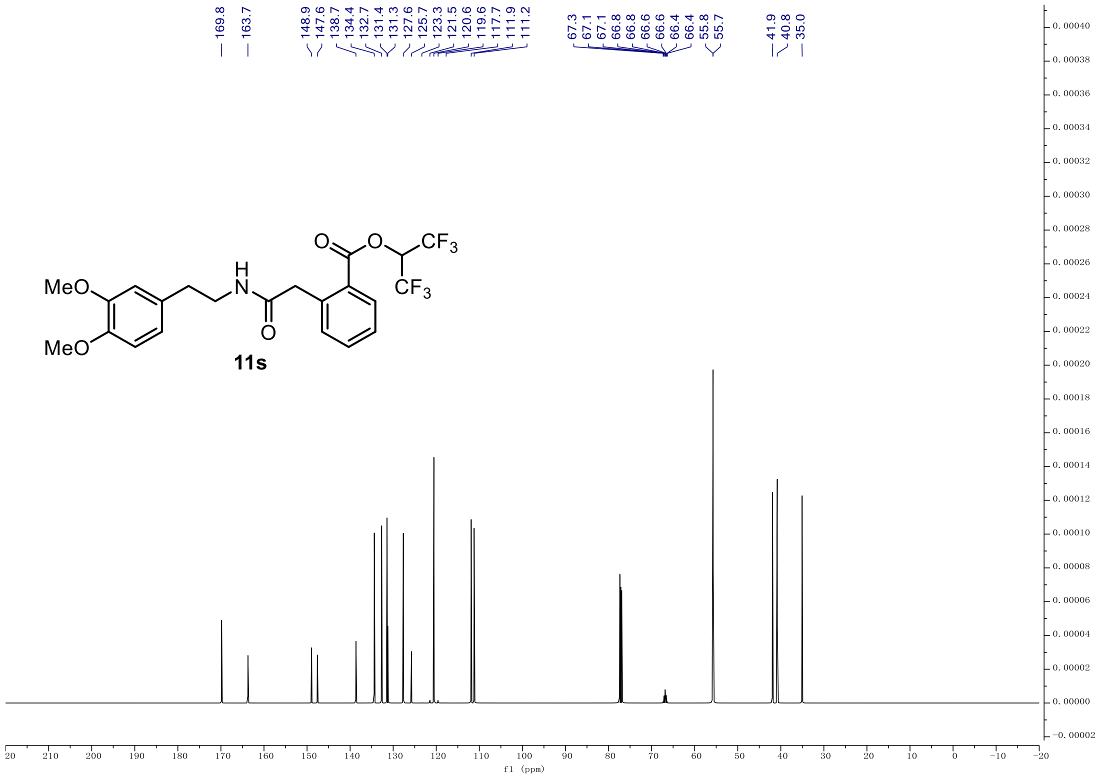

**$^{19}$F NMR** (565 MHz, $CDCl_3$) **11s**.

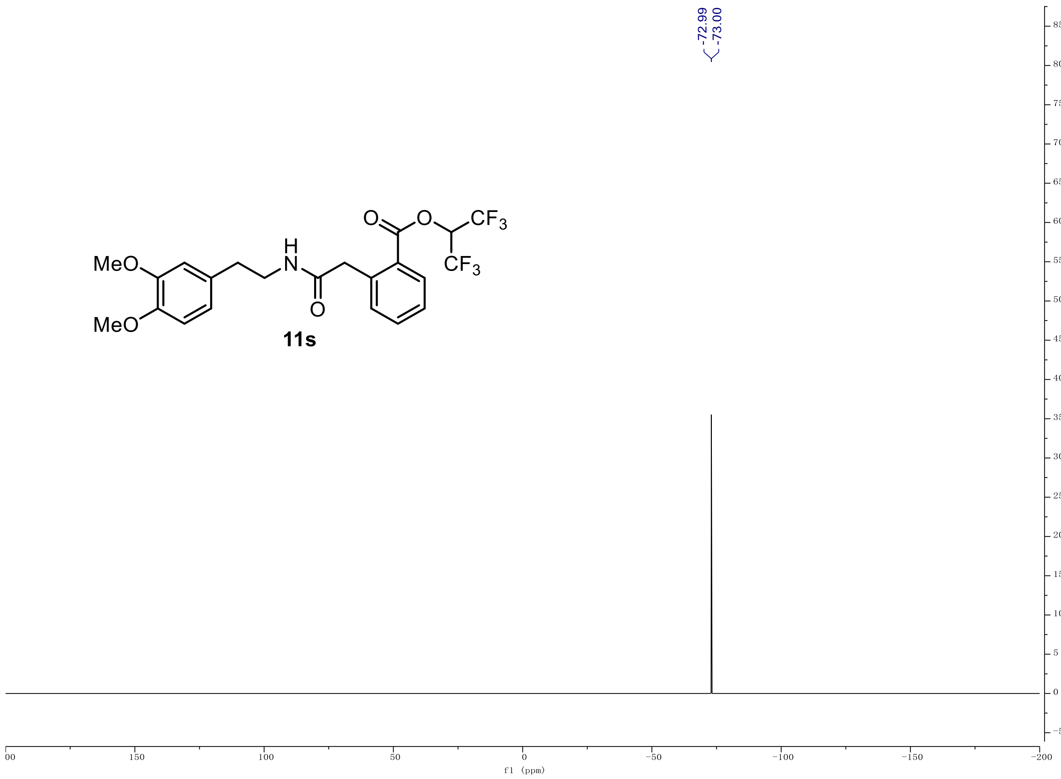


**$^{1}$H NMR** (700 MHz, $CDCl_3$) **11t**.

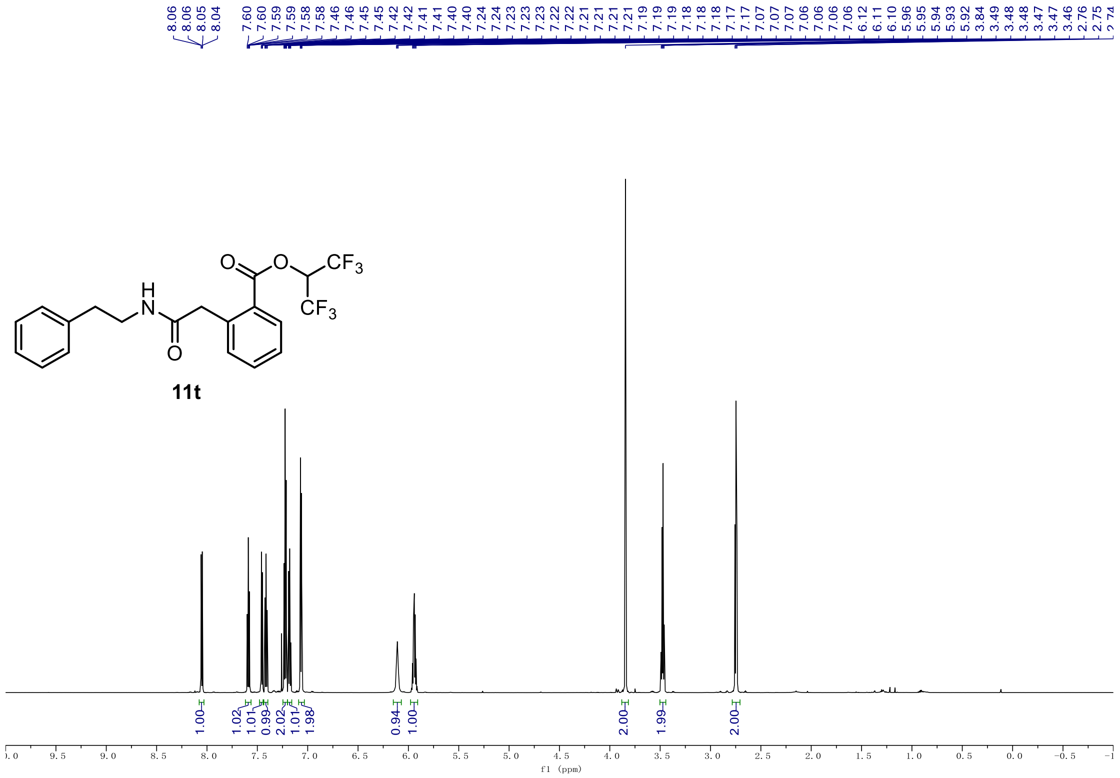

**$^{13}$C NMR** (176 MHz, $CDCl_3$) **11t**.

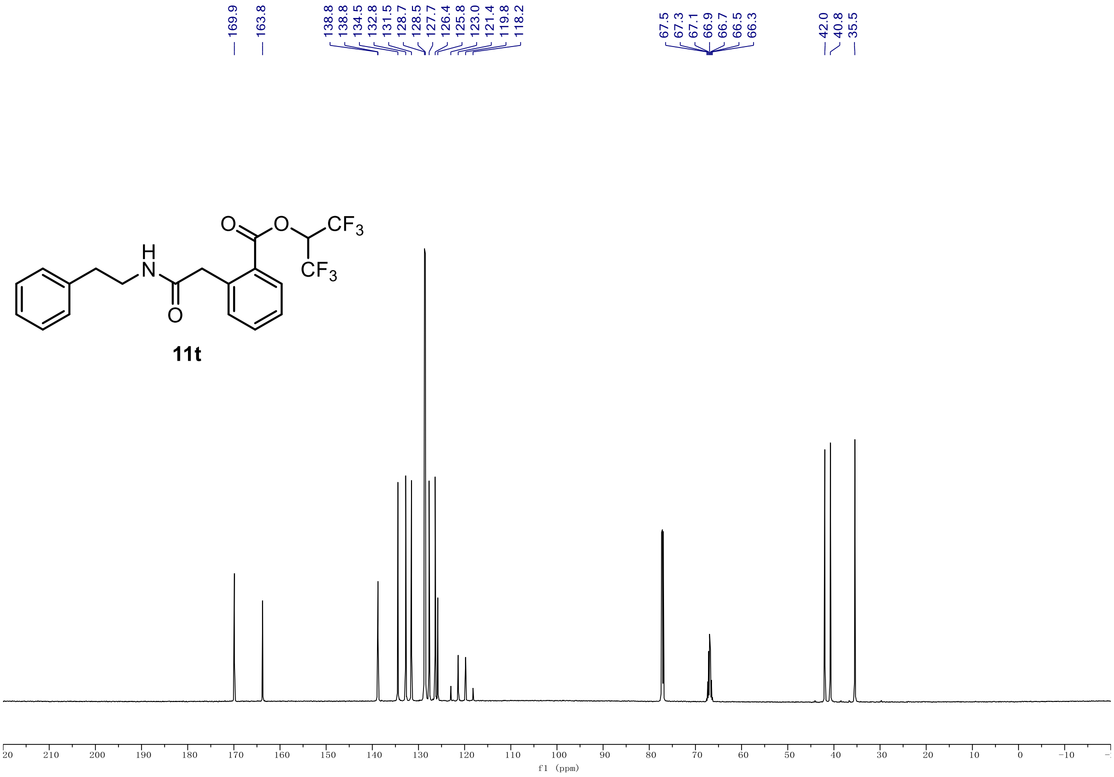


**$^{19}$F NMR** (376 MHz, $CDCl_3$) **11t**.

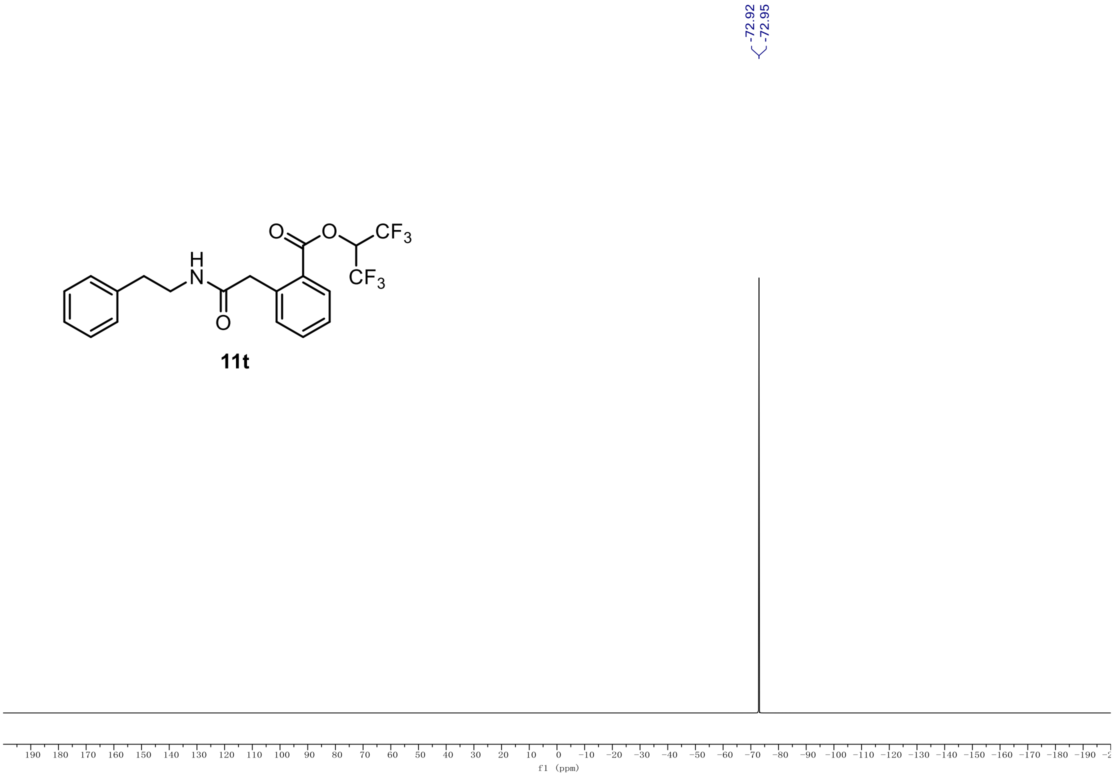

**$^{1}$H NMR** (700 MHz, $CDCl_3$) **11u**.

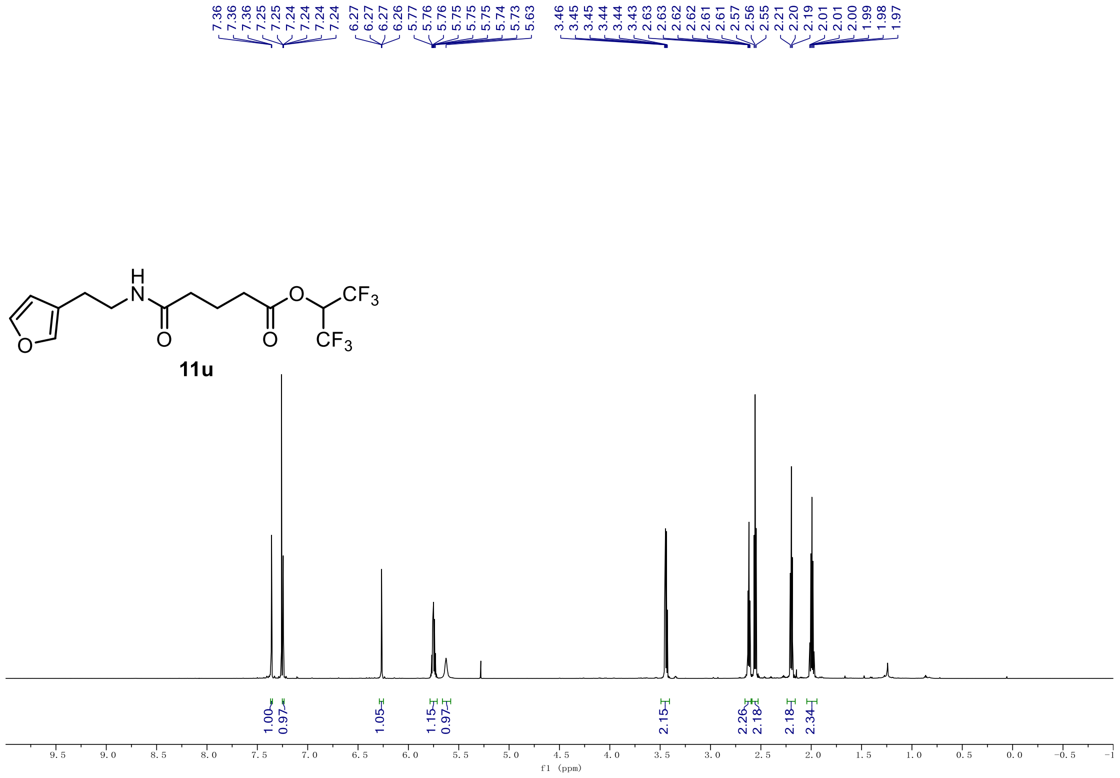


**$^{13}$C NMR** (176 MHz, $CDCl_3$) **11u**.

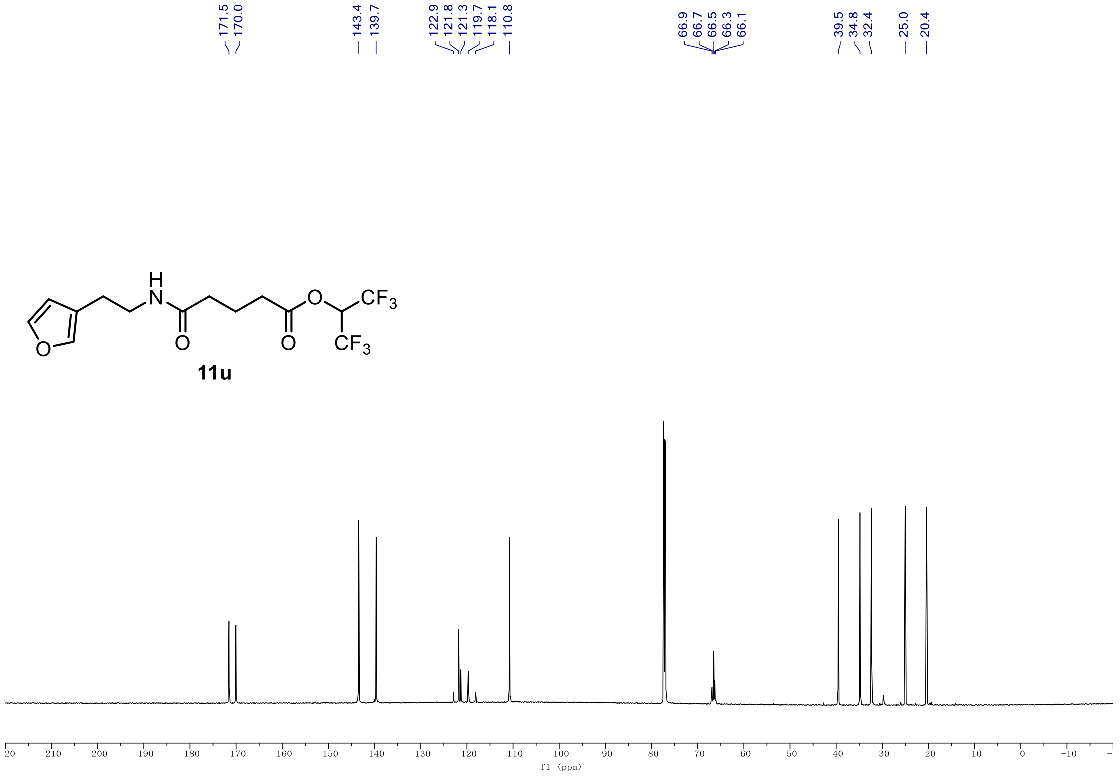

**$^{19}$F NMR** (565 MHz, $CDCl_3$) **11u**.

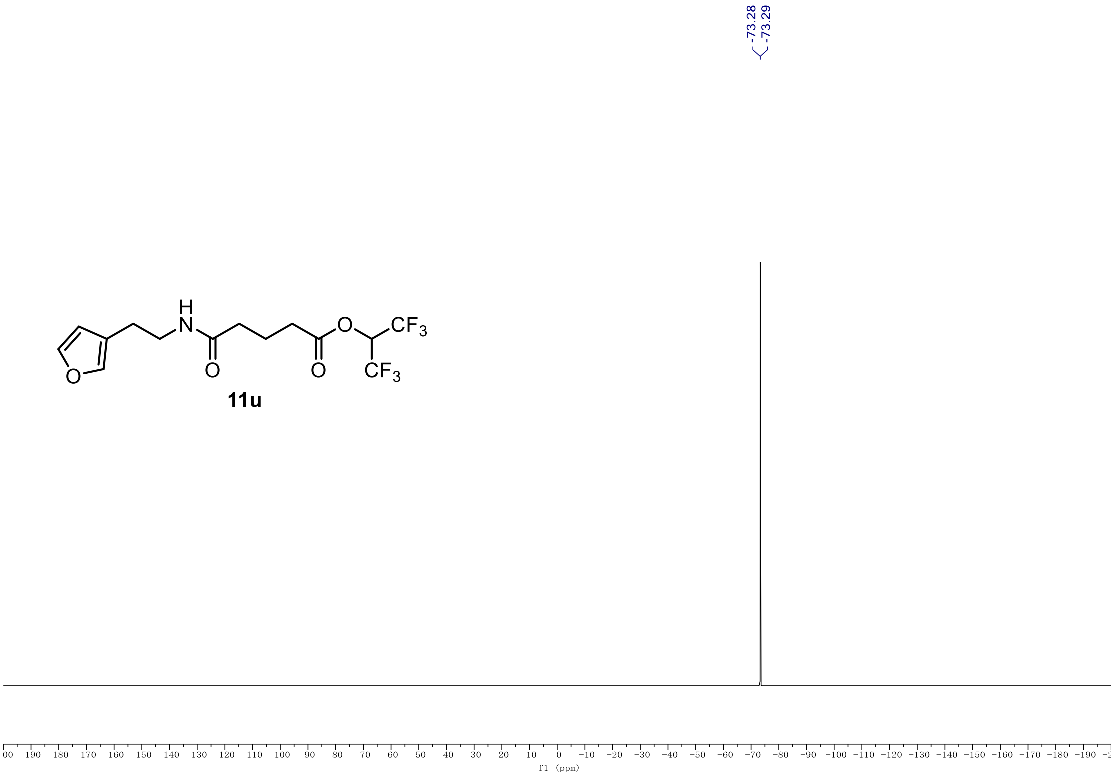


**$^{1}$H NMR** (700 MHz, $CDCl_3$) **11v**.

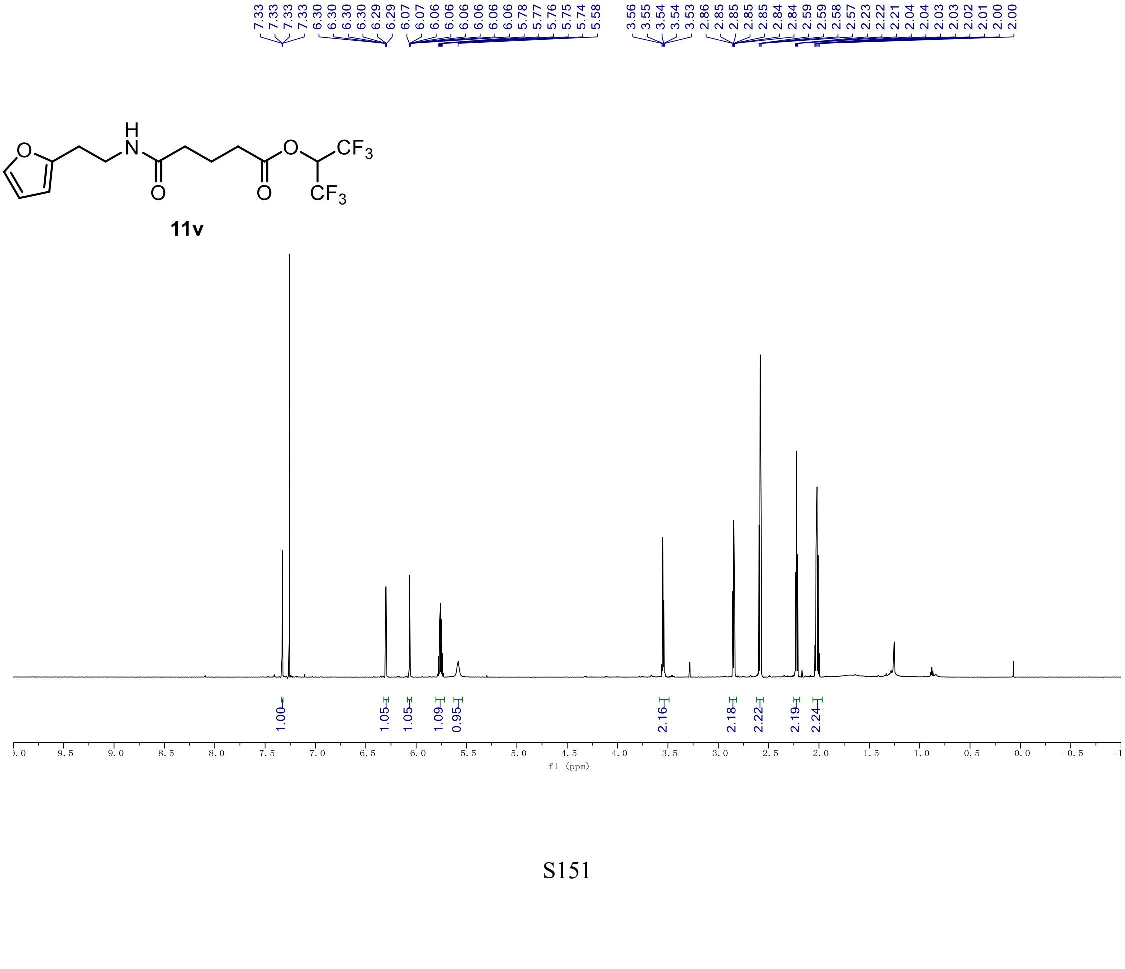

**$^{13}$C NMR** (176 MHz, $CDCl_3$) **11v**.

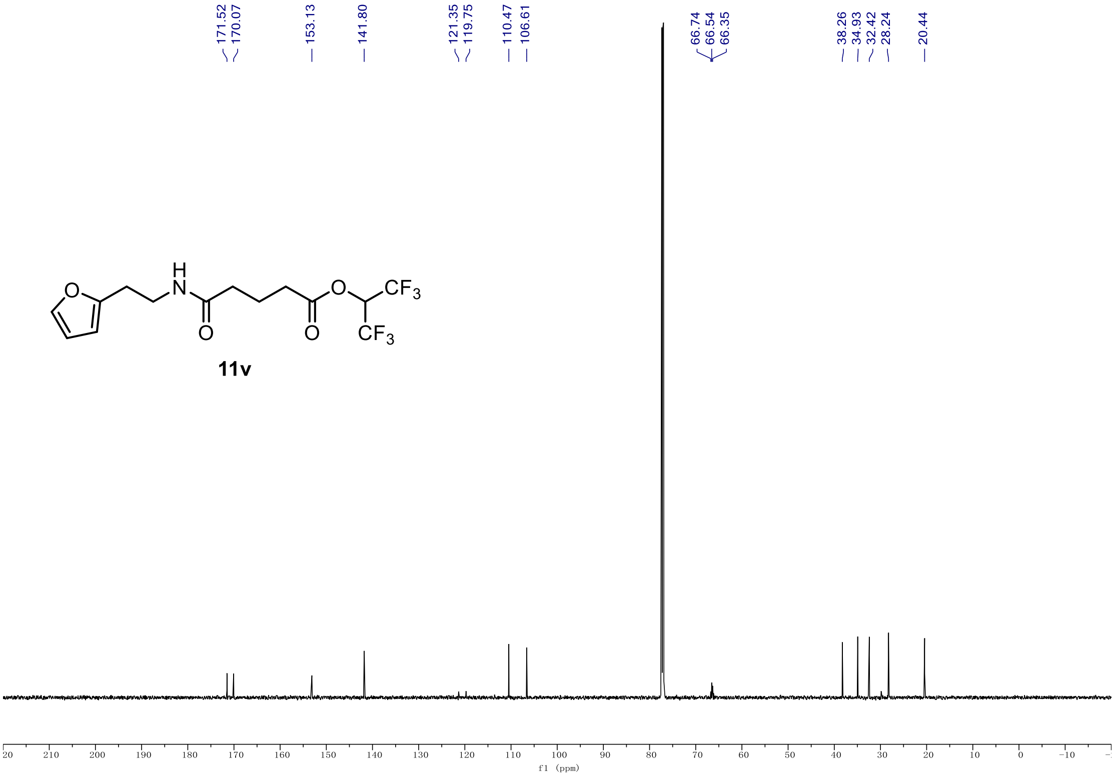


**$^{19}$F NMR** (376 MHz, $CDCl_3$) **11v**.

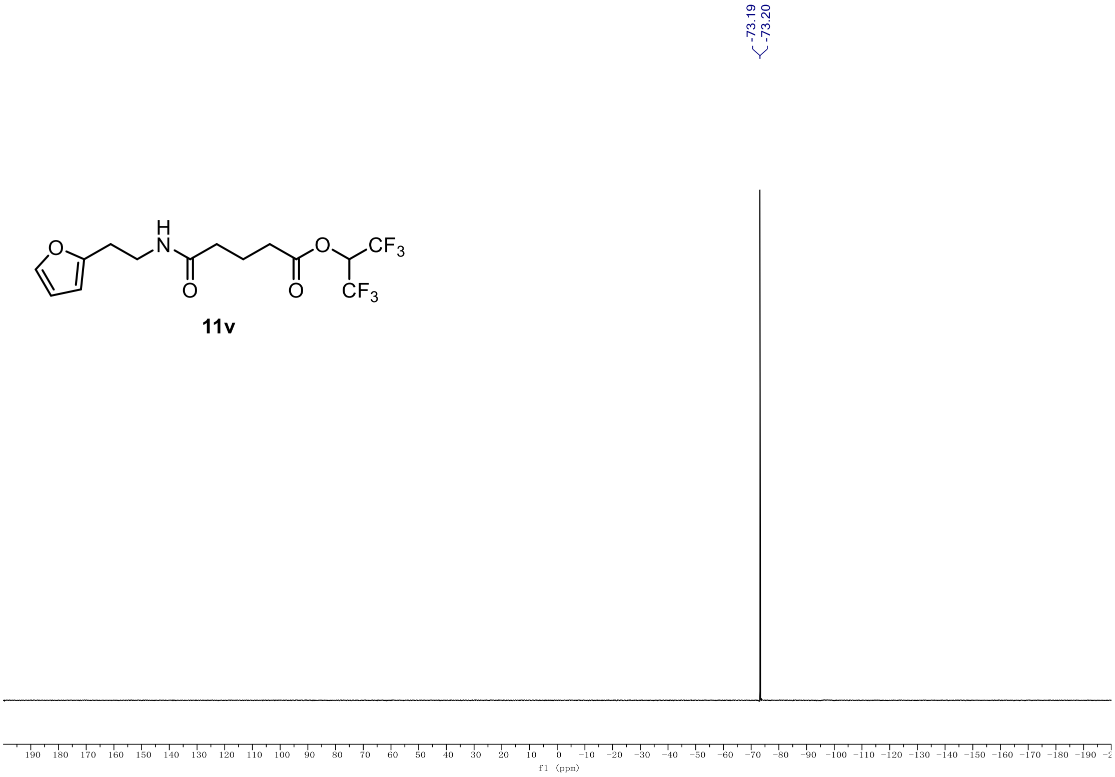

**$^{1}$H NMR** (500 MHz, $CDCl_3$) **11w**.

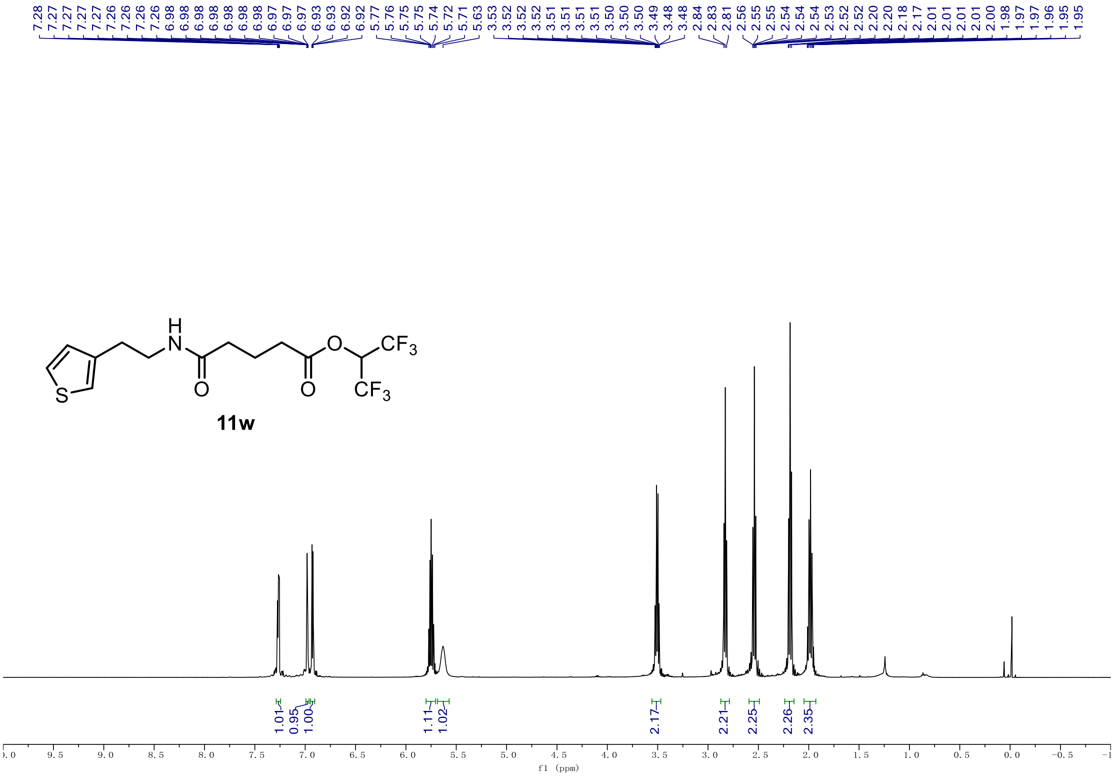


**$^{13}$C NMR** (126 MHz, $CDCl_3$) **11w**.

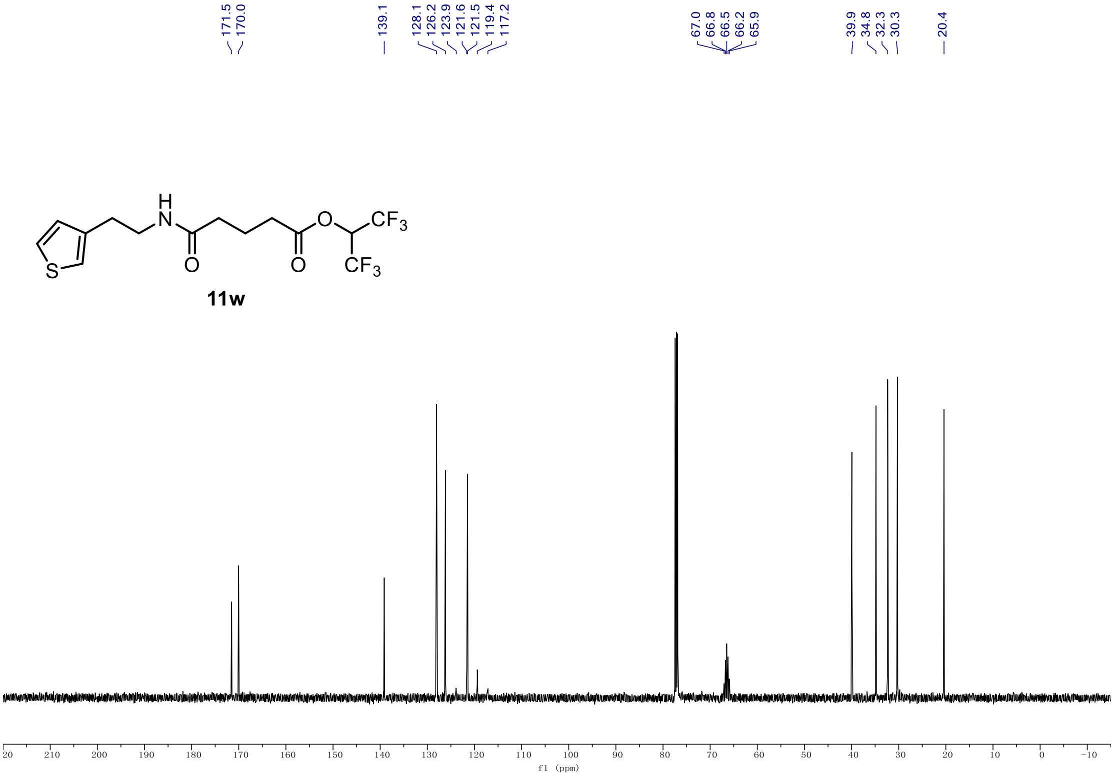

**$^{19}$F NMR** (471 MHz, $CDCl_3$) **11w**.

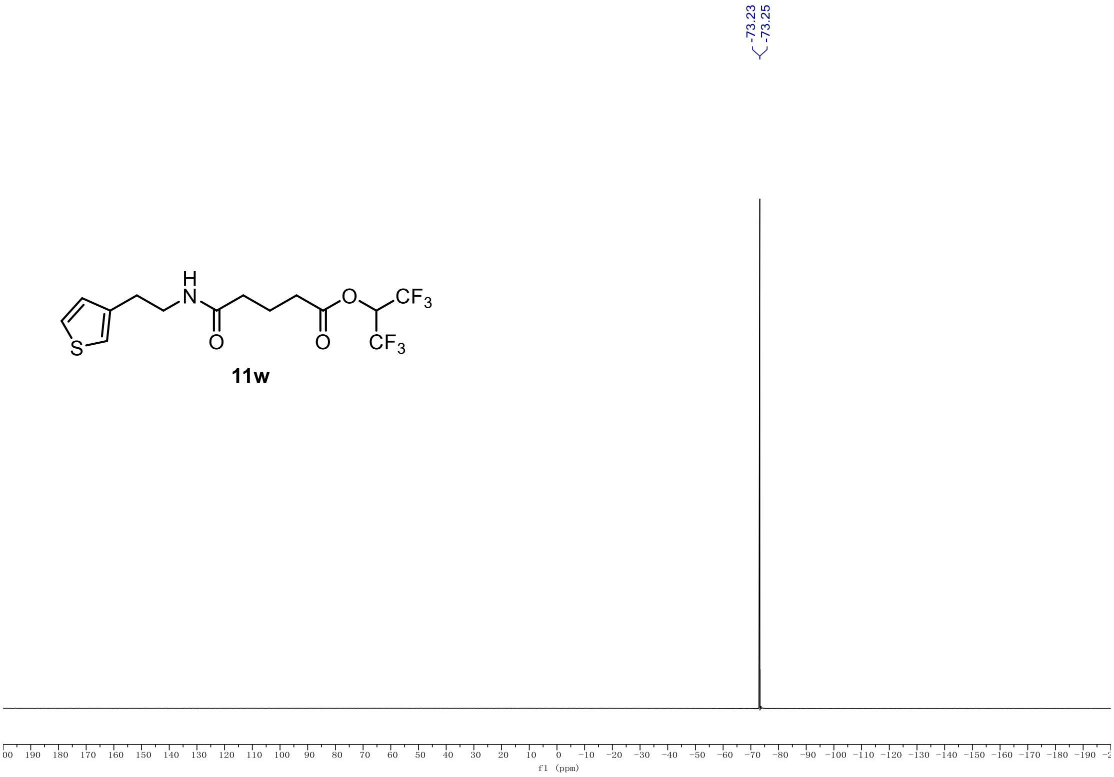


**$^{1}$H NMR** (700 MHz, $CDCl_3$) **11x**.

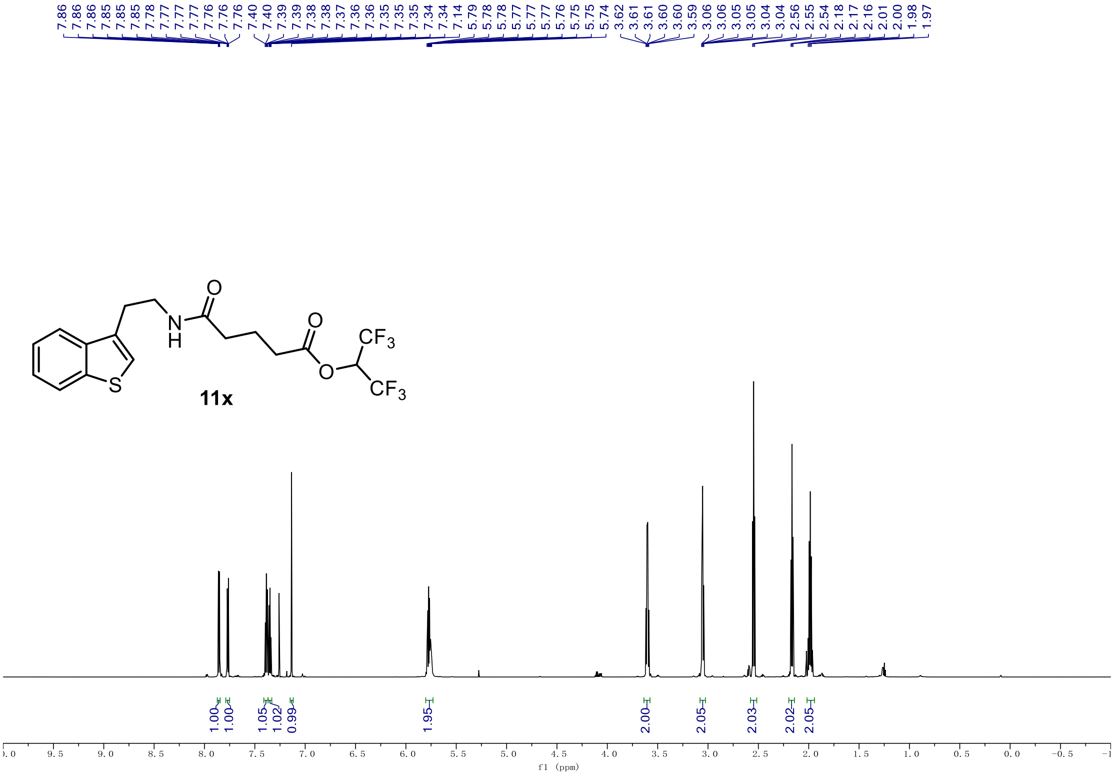

**$^{13}$C NMR** (176 MHz, $CDCl_3$) **11x**.

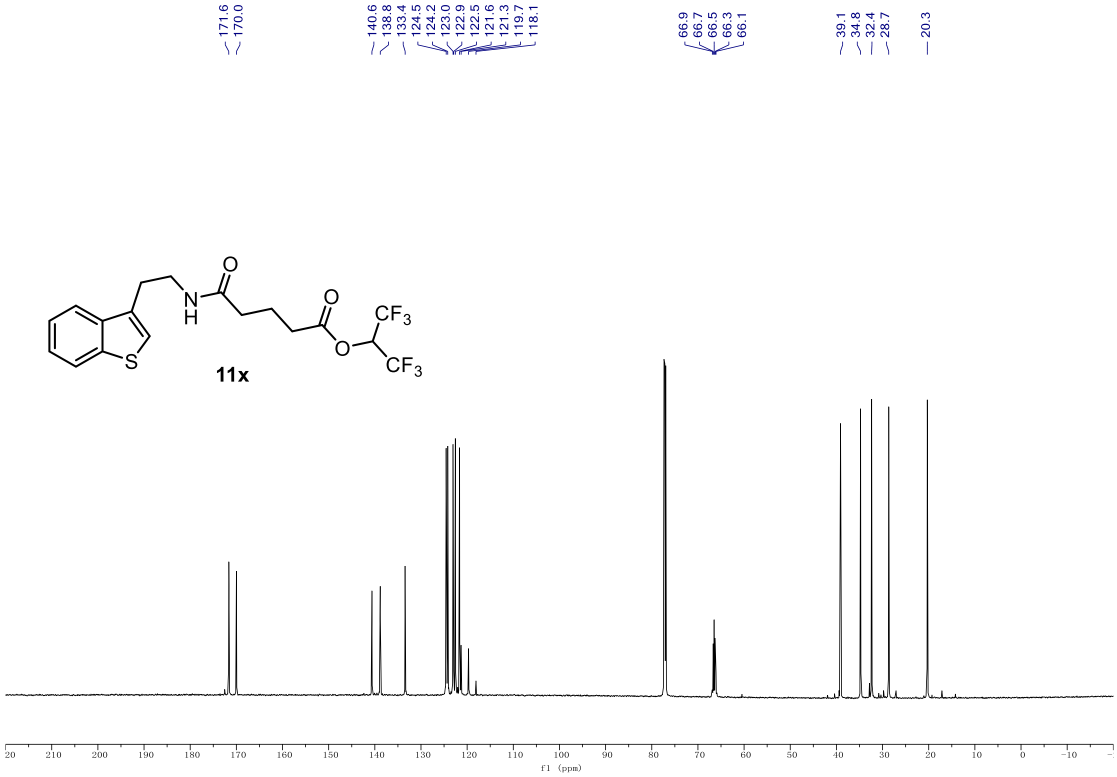


**$^{19}$F NMR** (376 MHz, $CDCl_3$) **11x**.

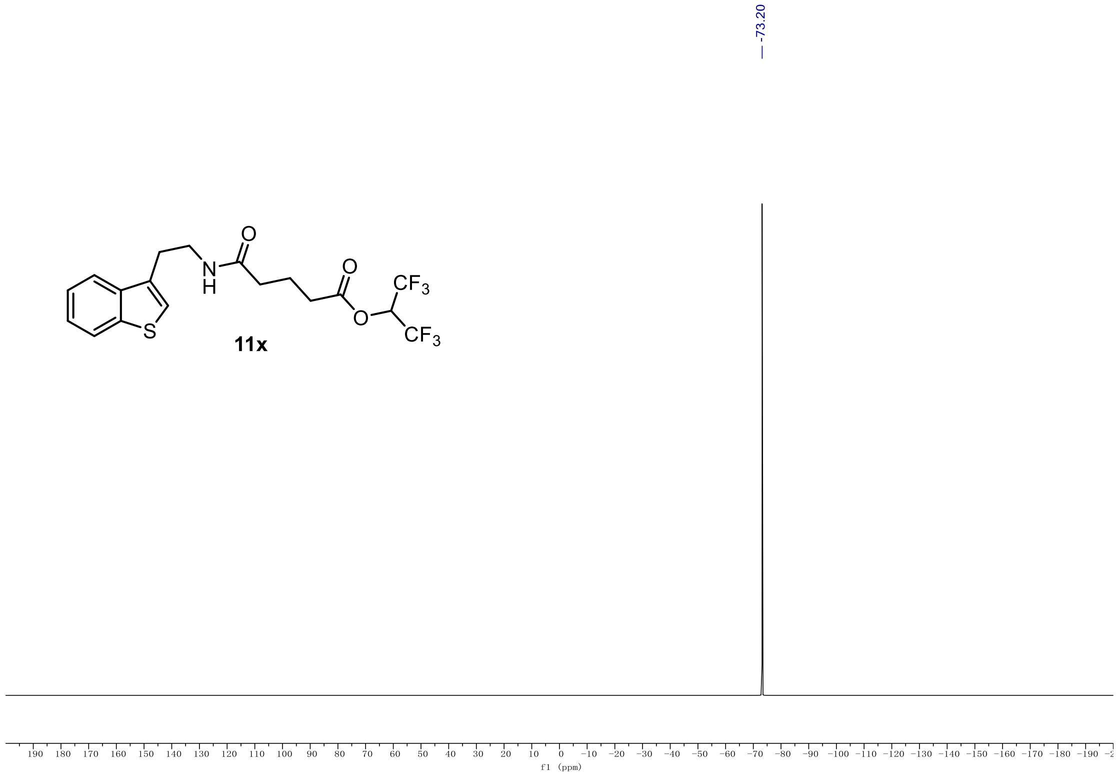

**$^{1}$H NMR** (700 MHz, $CDCl_3$) of **11y**.

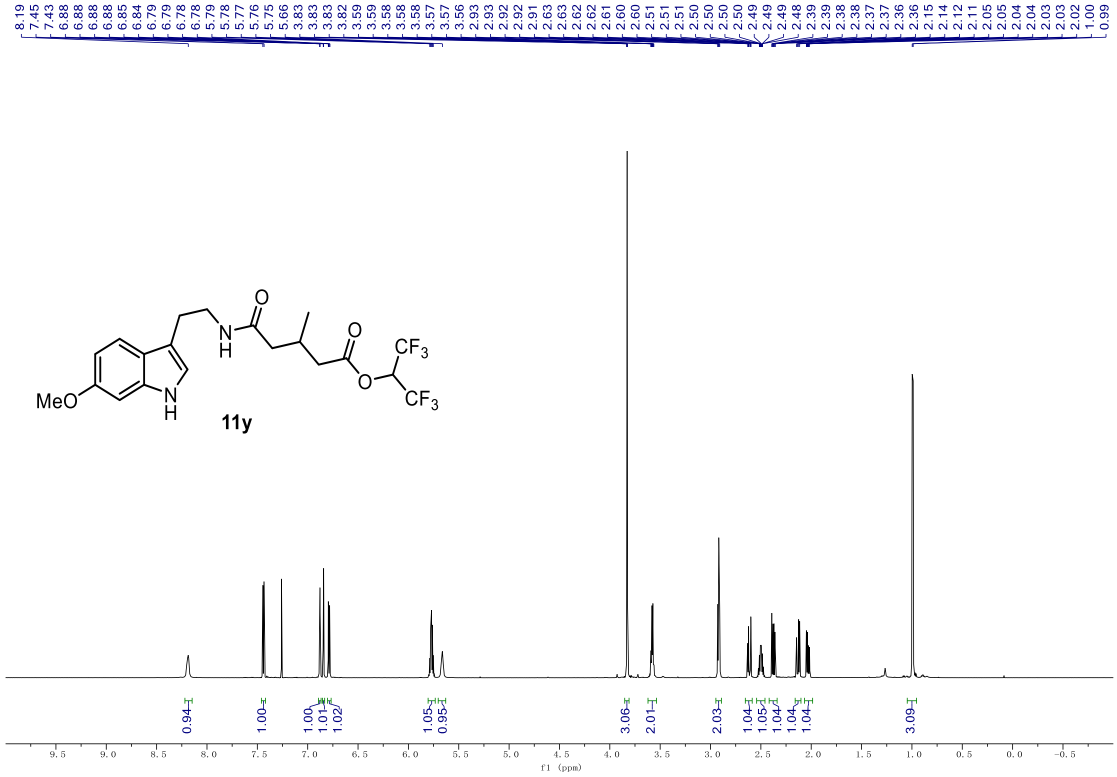


**$^{13}$C NMR** (176 MHz, $CDCl_3$) of **11y**.

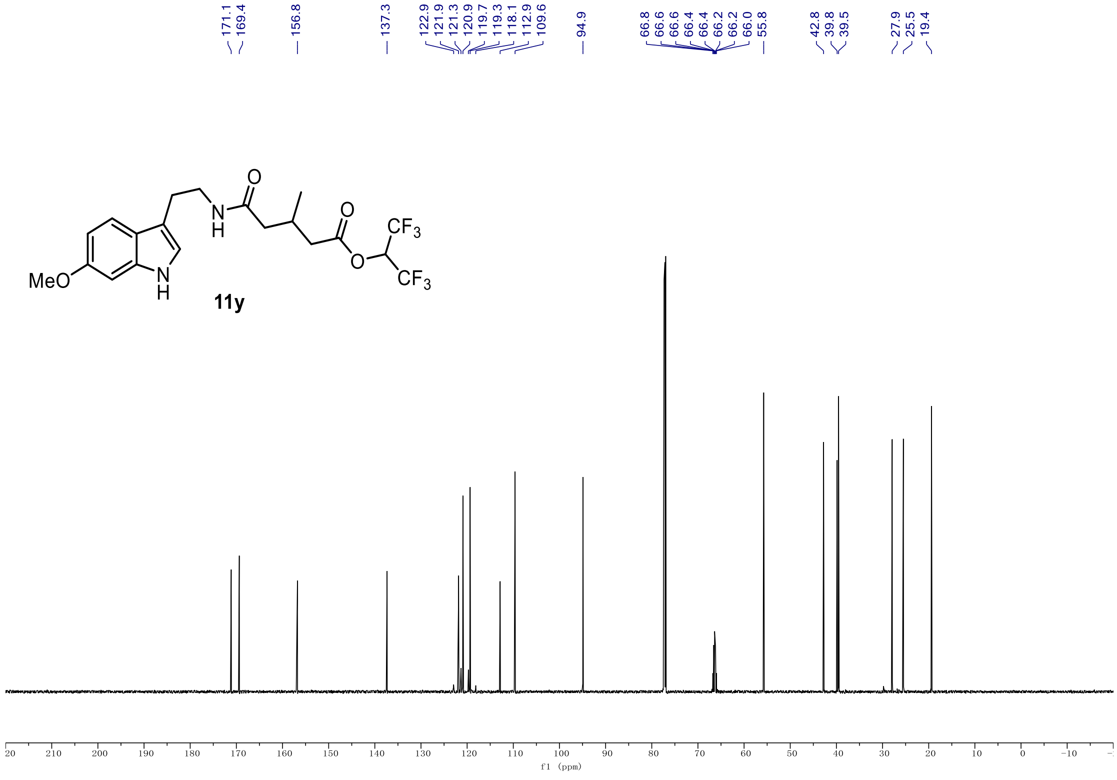

**$^{19}$F NMR** (376 MHz, $CDCl_3$) of **11y**.

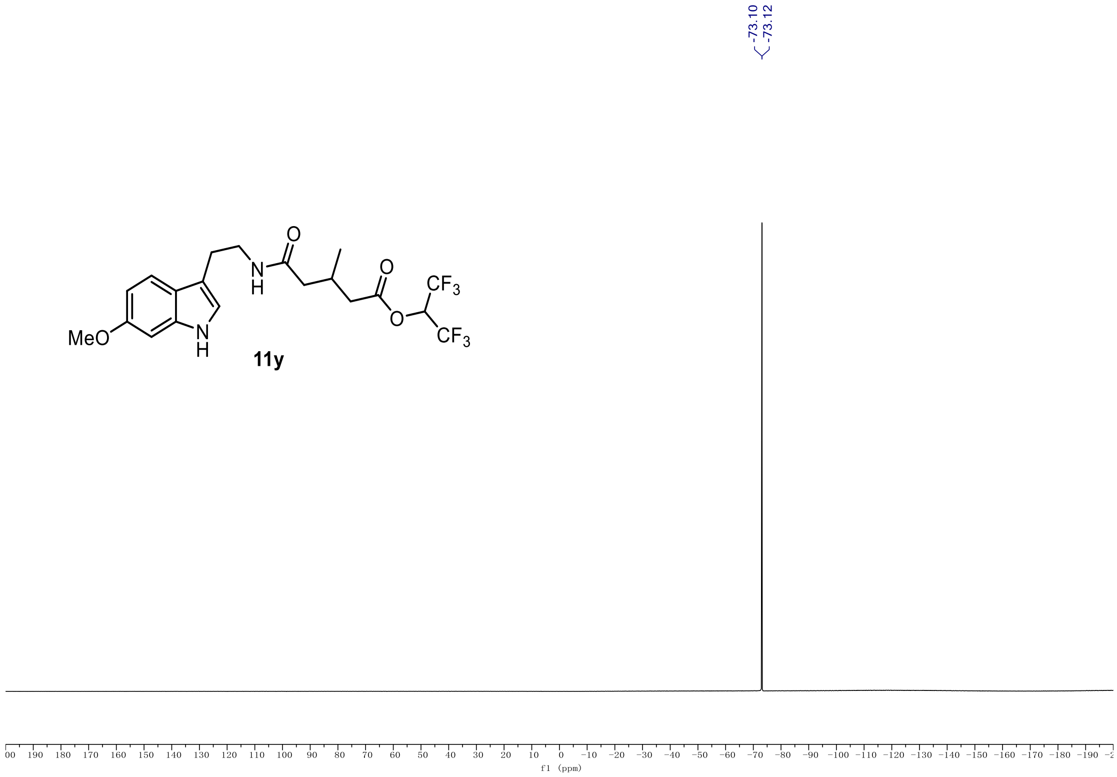


**$^{1}$H NMR** (600 MHz, $CDCl_3$) of **S1**.

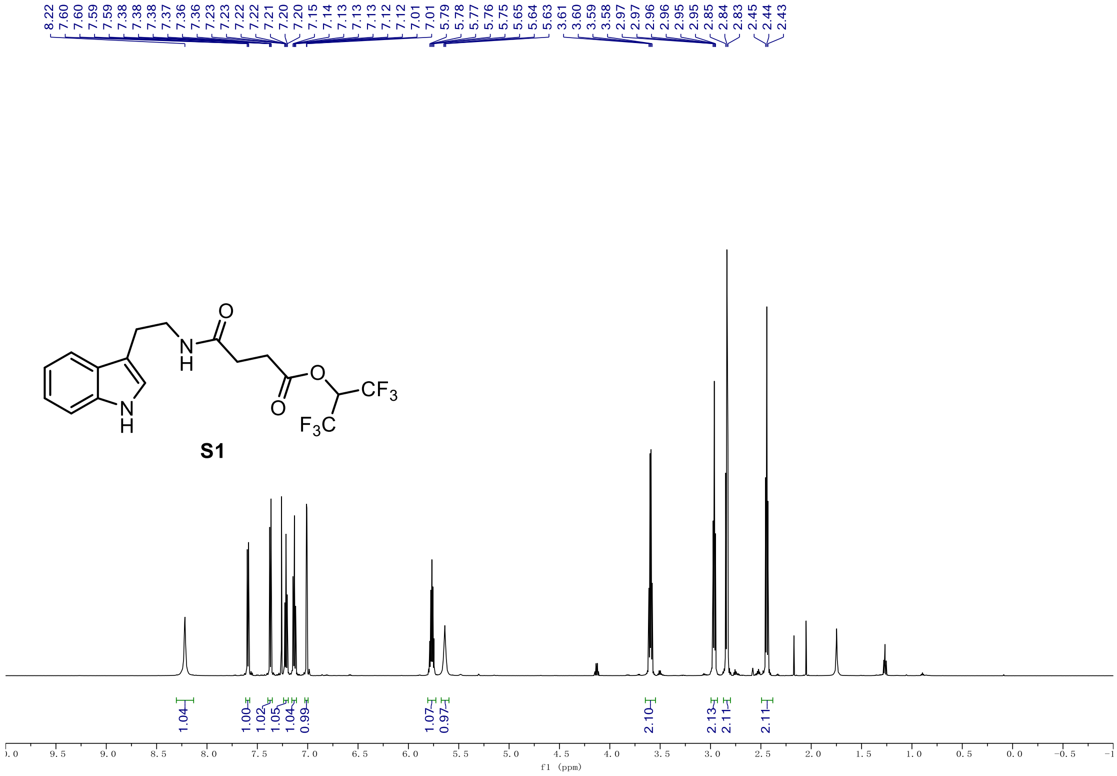

**$^{13}$C NMR** (151 MHz, $CDCl_3$) of **S1**.

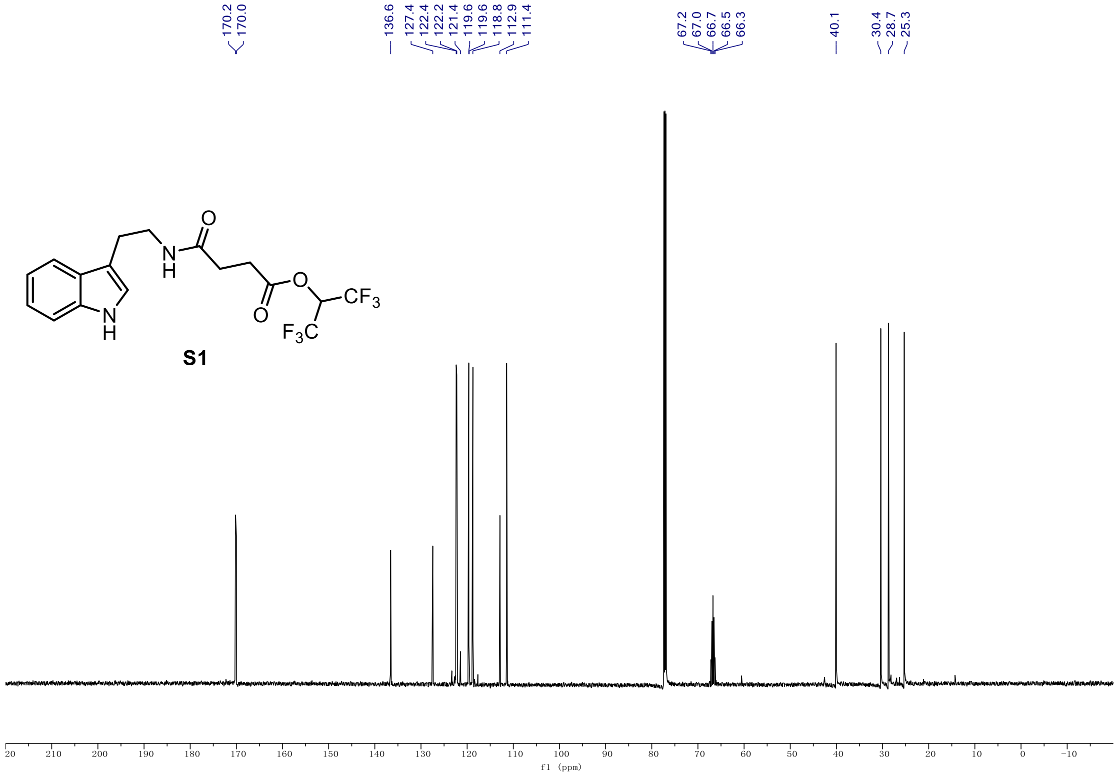


**$^{19}$F NMR** (565 MHz, $CDCl_3$) of **S1**.

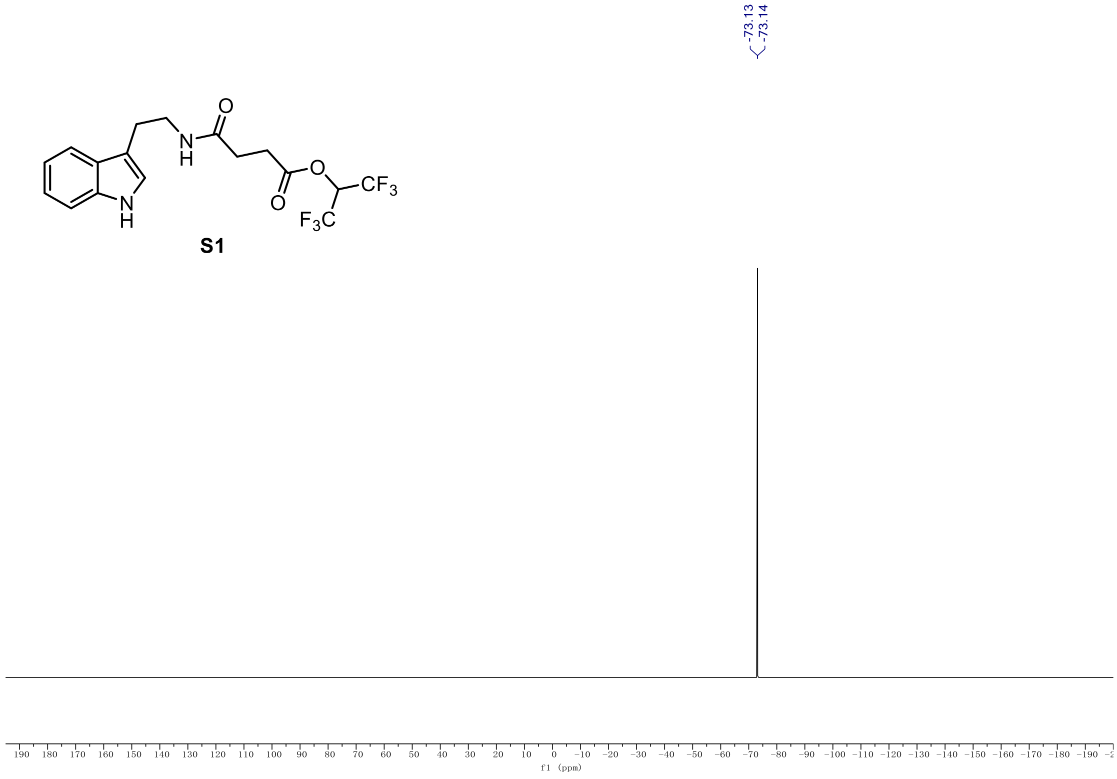

**$^{1}$H NMR** (700 MHz, $CDCl_3$) of **S2**.

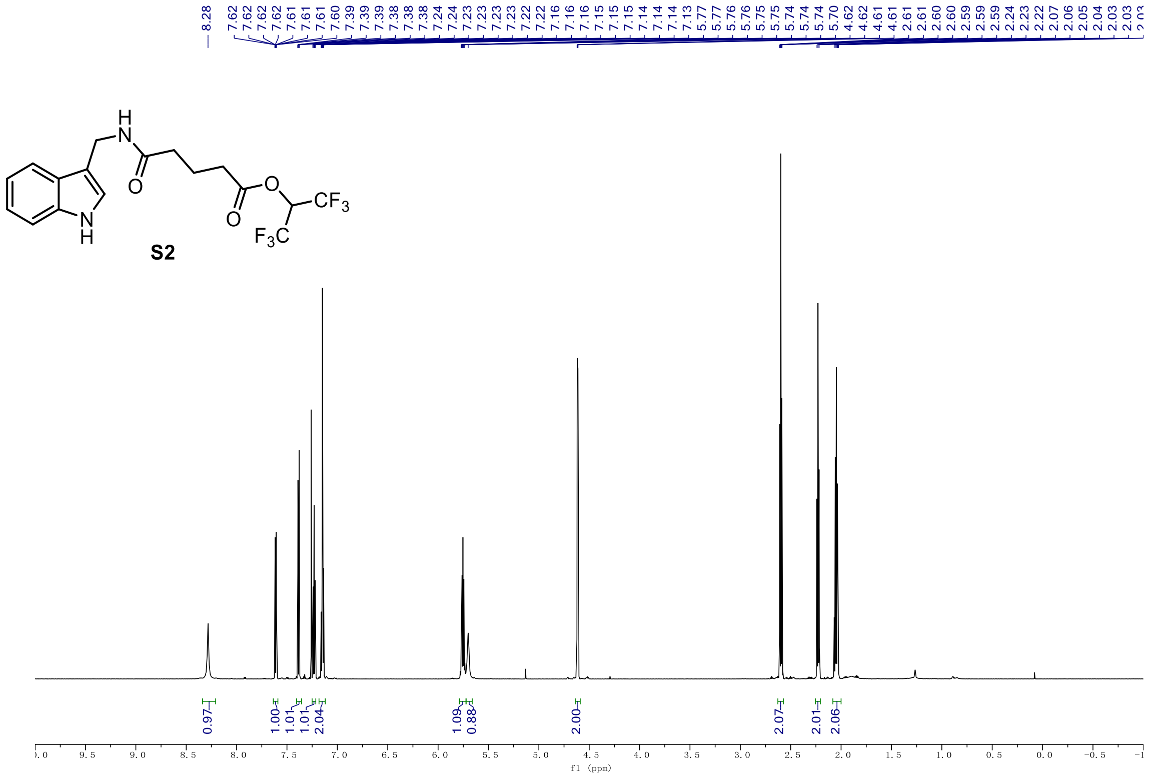


**$^{13}$C NMR** (176 MHz, $CDCl_3$) of **S2**.

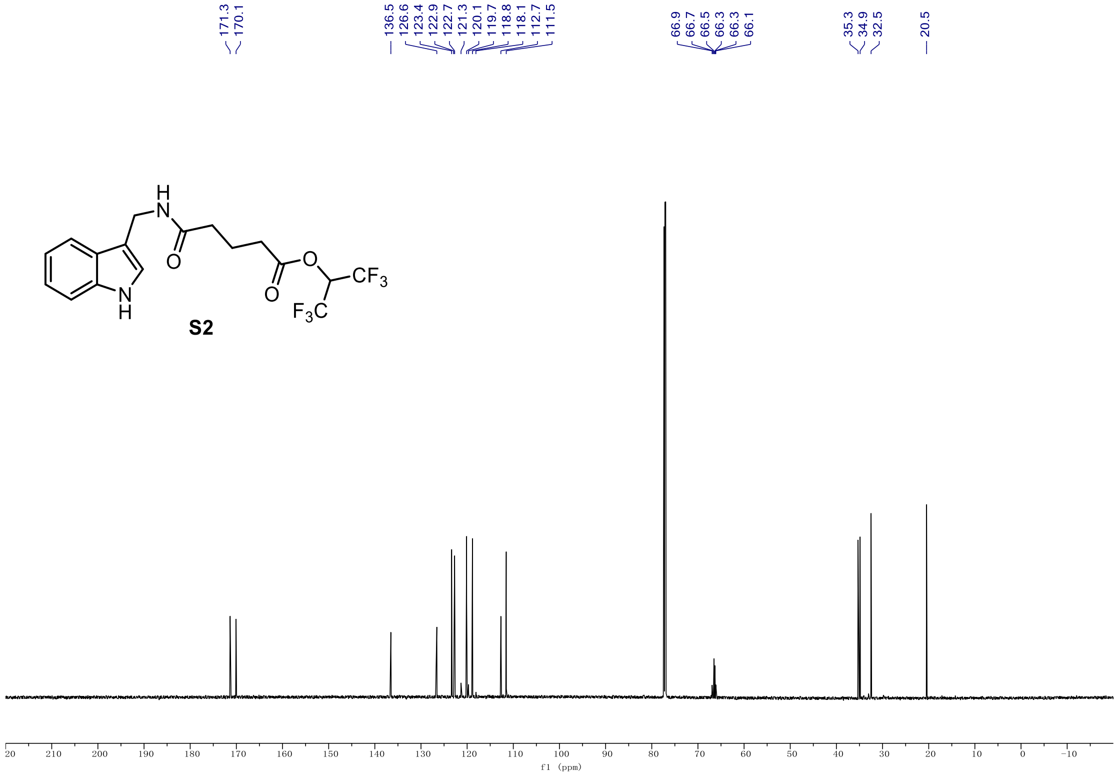

**$^{19}$F NMR** (565 MHz, $CDCl_3$) of **S2**.

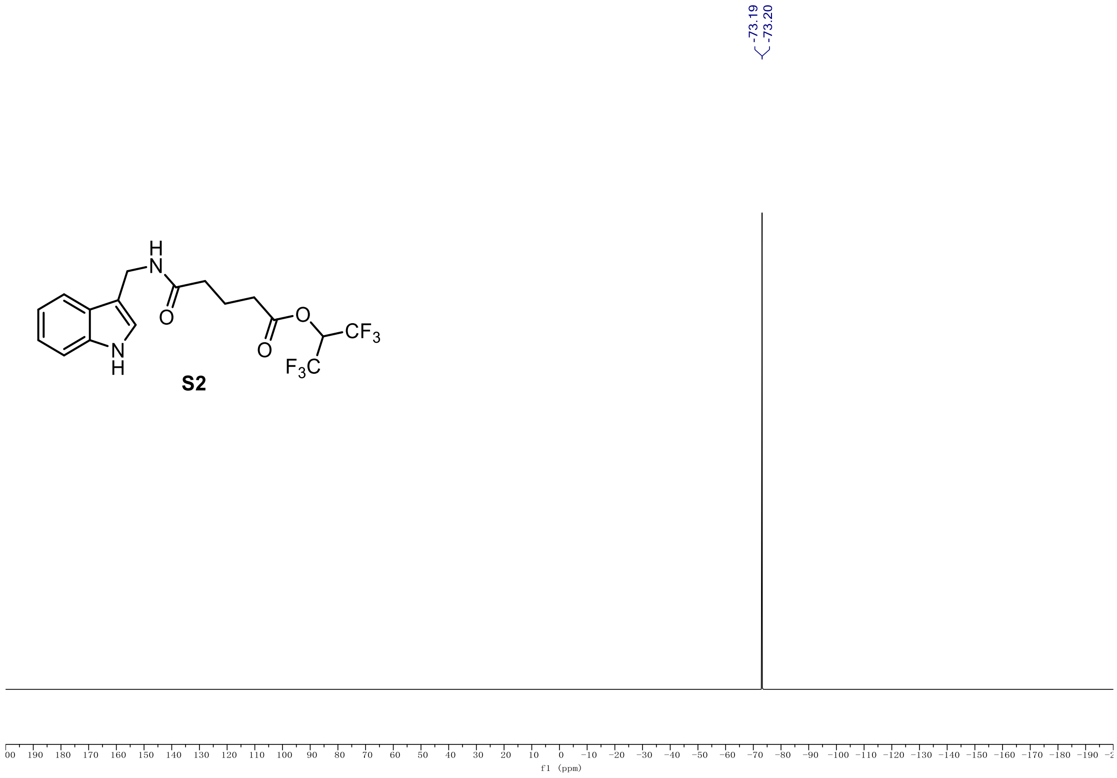

**$^{1}$H NMR** (500 MHz, $CDCl_3$) of **S3**.

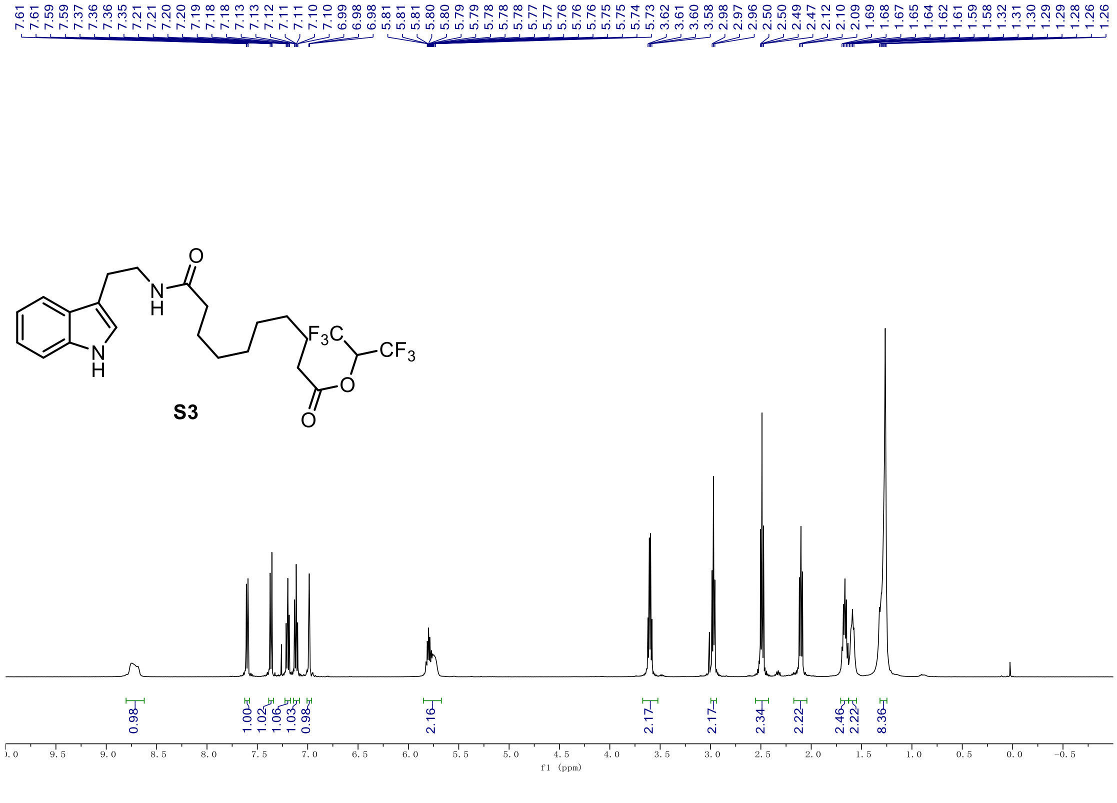

**$^{13}$C NMR** (126 MHz, $CDCl_3$) of **S3**.

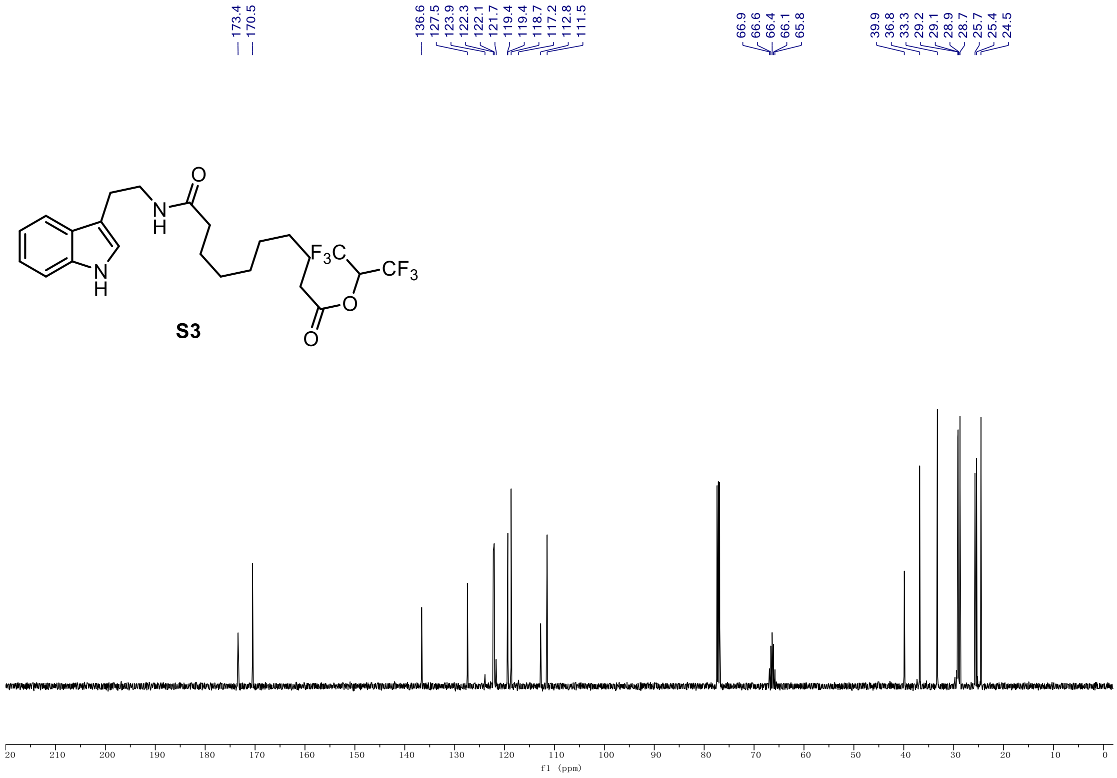


**$^{19}$F NMR** (471 MHz, $CDCl_3$) of **S3**.

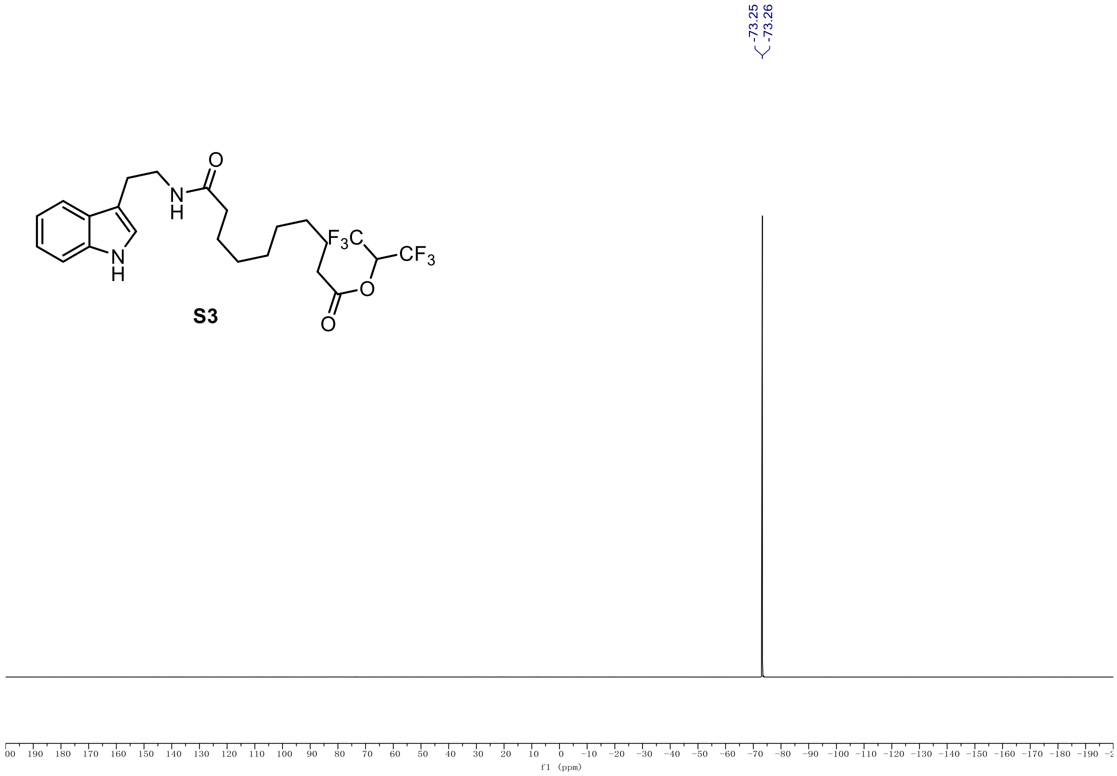

$^{1}$**H NMR** (500 MHz, $CDCl_3$) of **S4**.

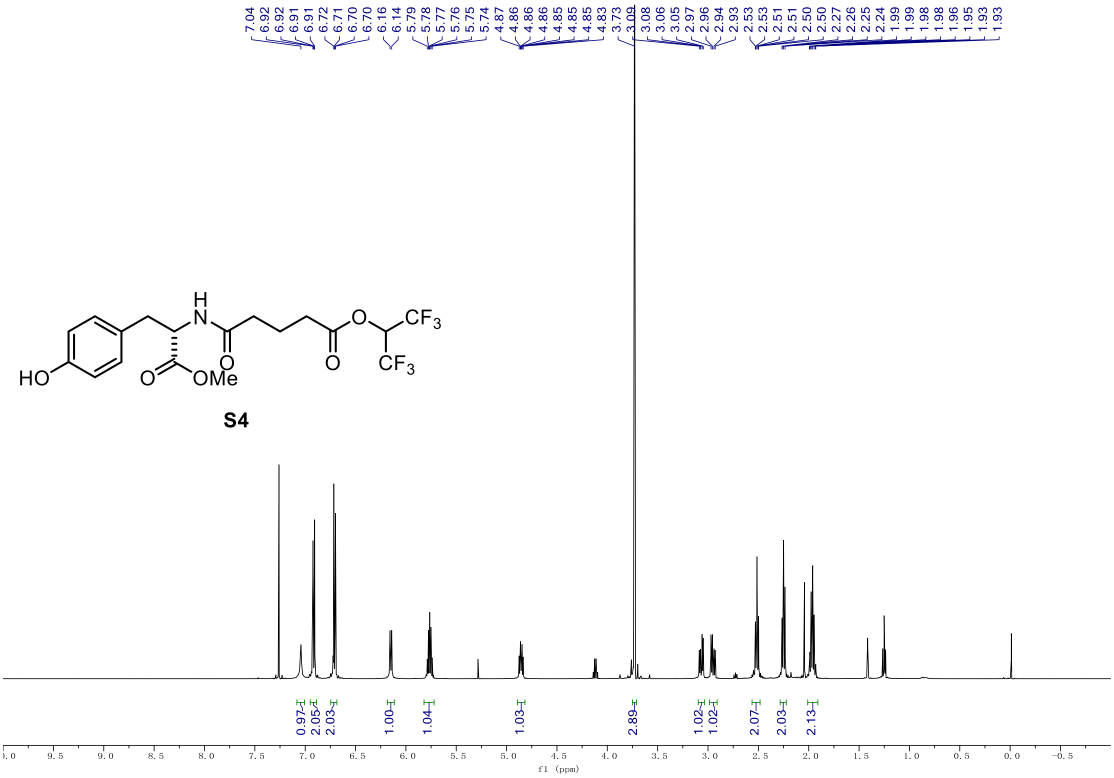

$^{13}$**C NMR** (126 MHz, $CDCl_3$) of **S4**.

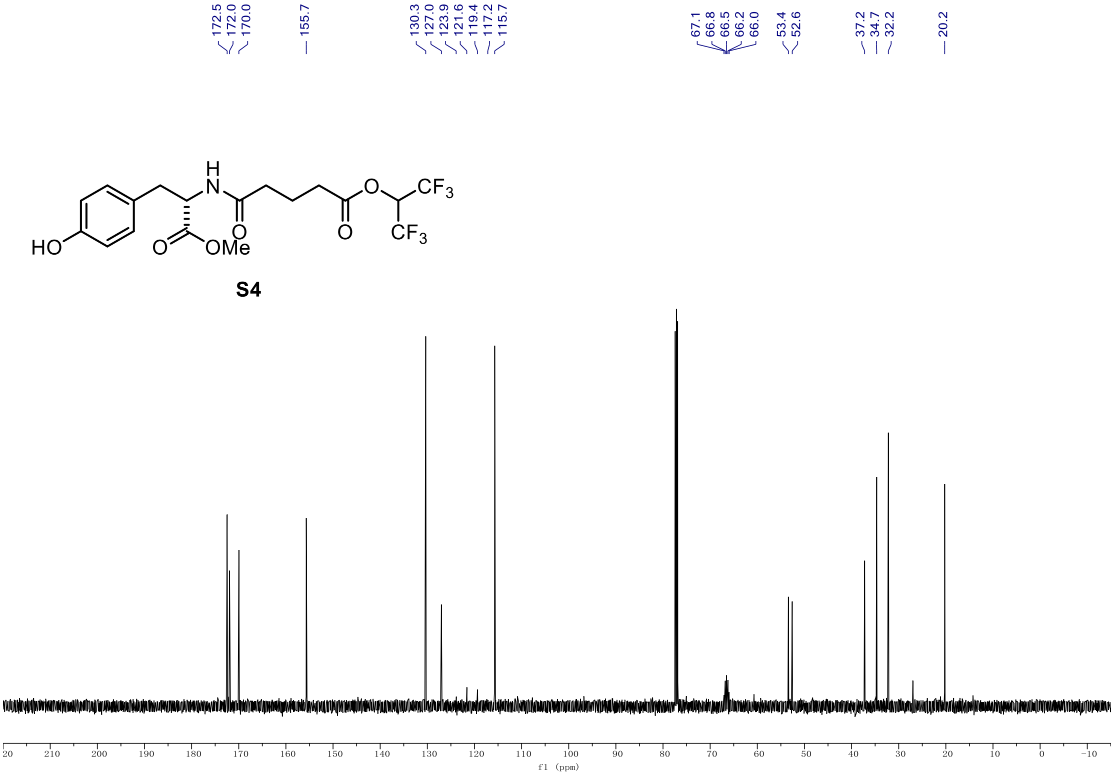

**$^{19}$F NMR** (471 MHz, $CDCl_3$) of **S4**.

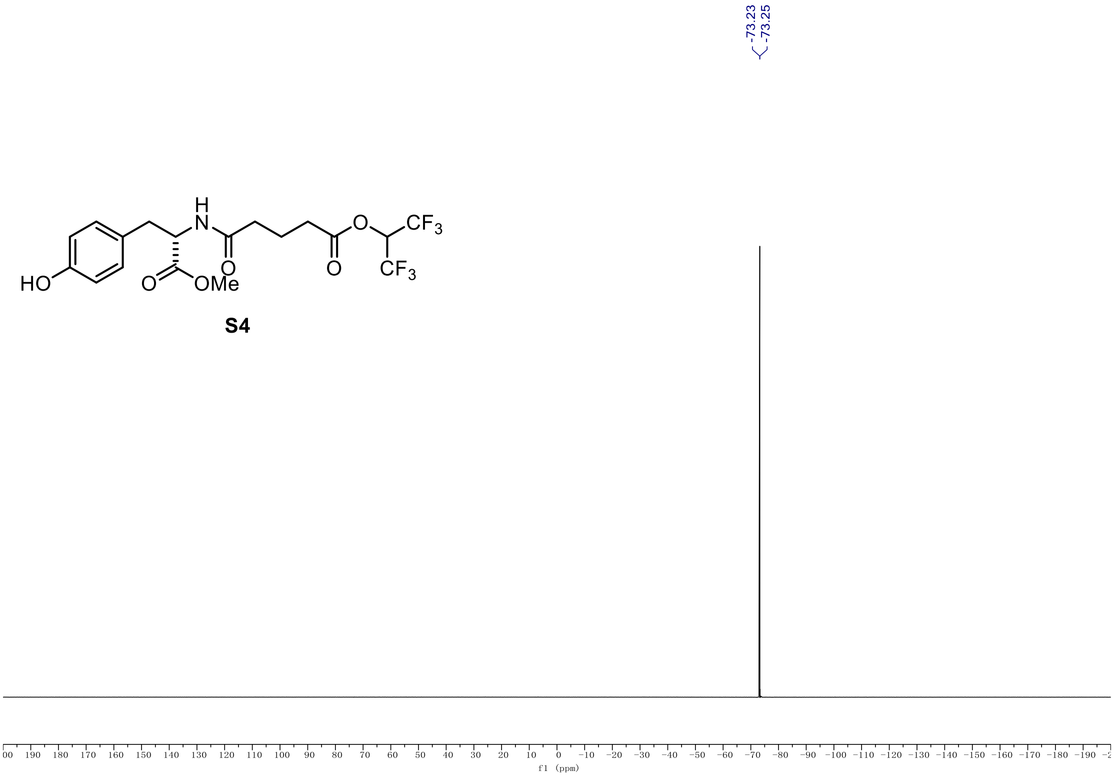


**$^{1}$H NMR** (500 MHz, $CDCl_3$) of **S5**.

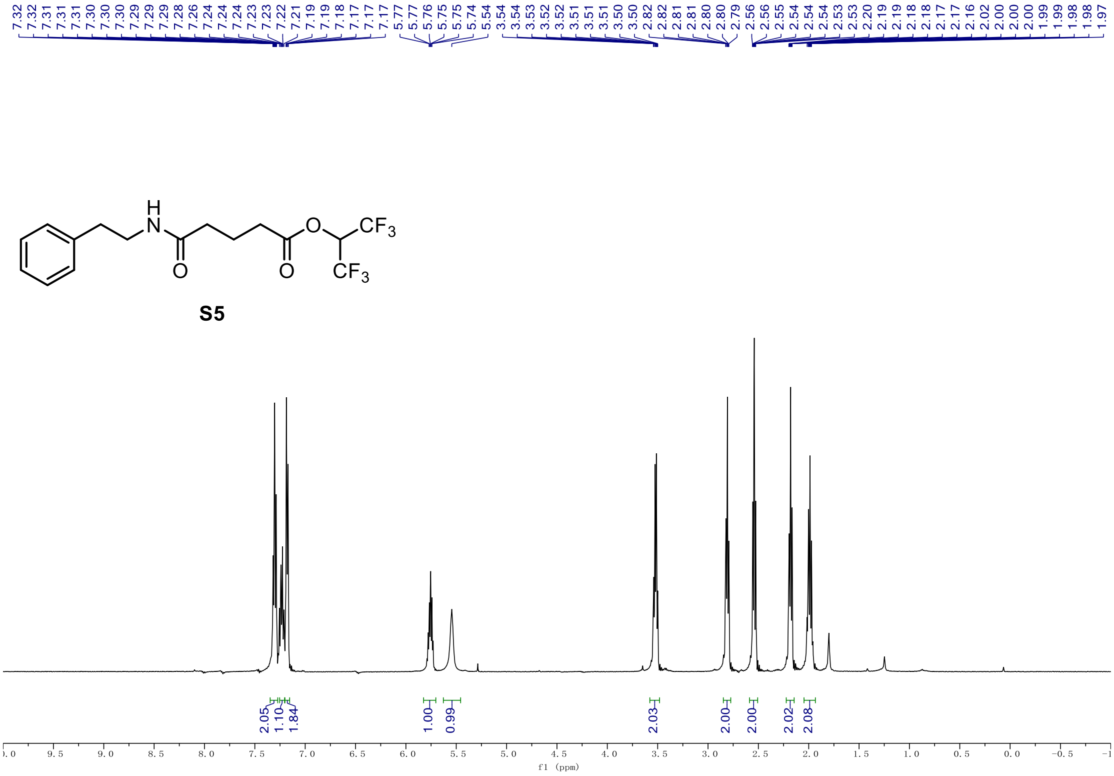

**$^{13}$C NMR** (126 MHz, $CDCl_3$) of **S5**.

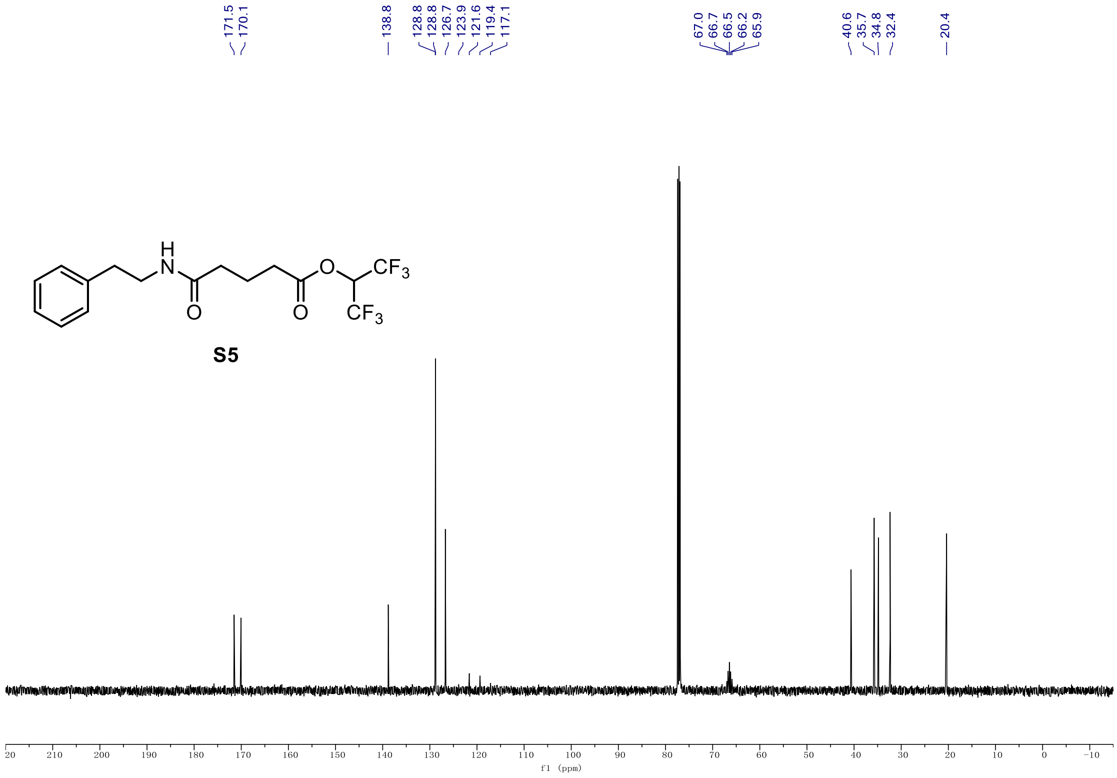


**$^{19}$F NMR** (471 MHz, $CDCl_3$) of **S5**.

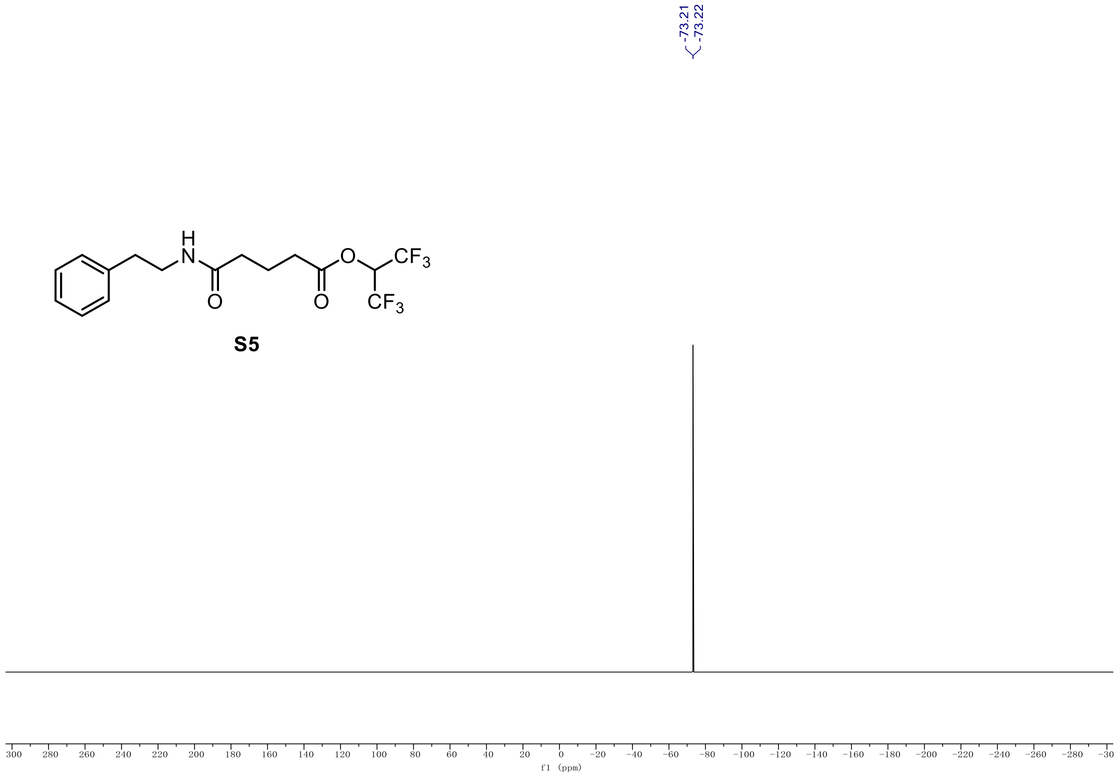

**$^{1}$H NMR** (500 MHz, $CDCl_3$) of **S6**.

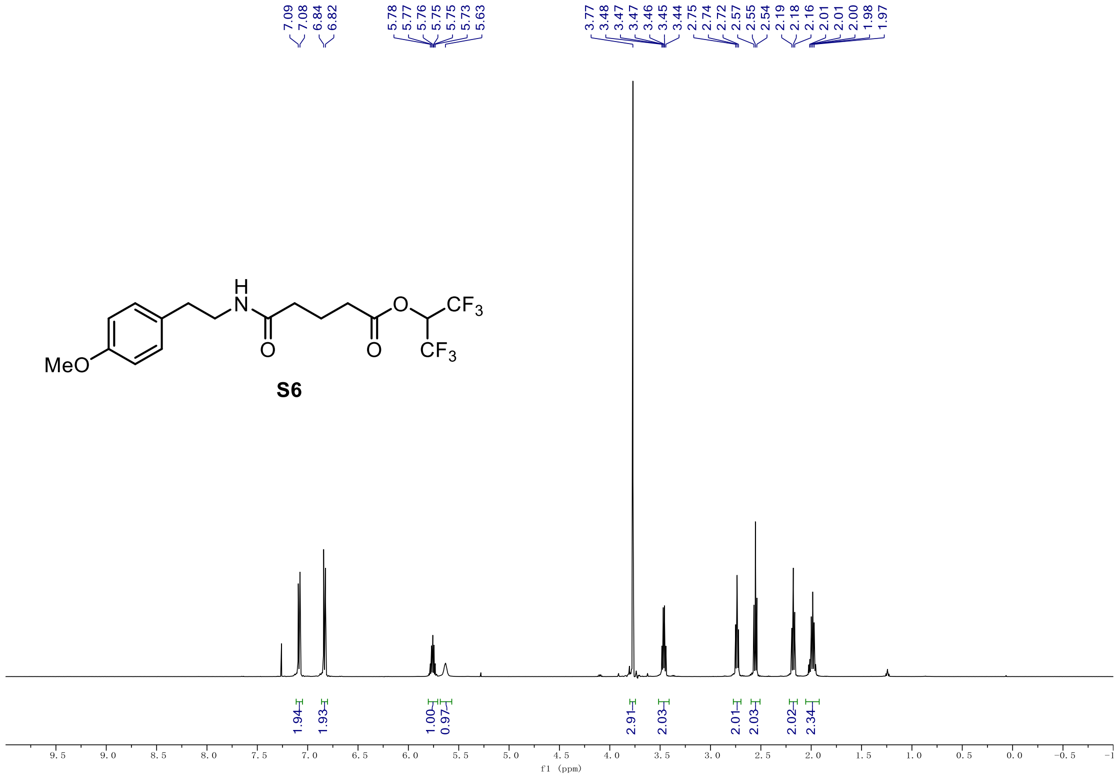

**$^{13}$C NMR** (126 MHz, $CDCl_3$) of **S6**.

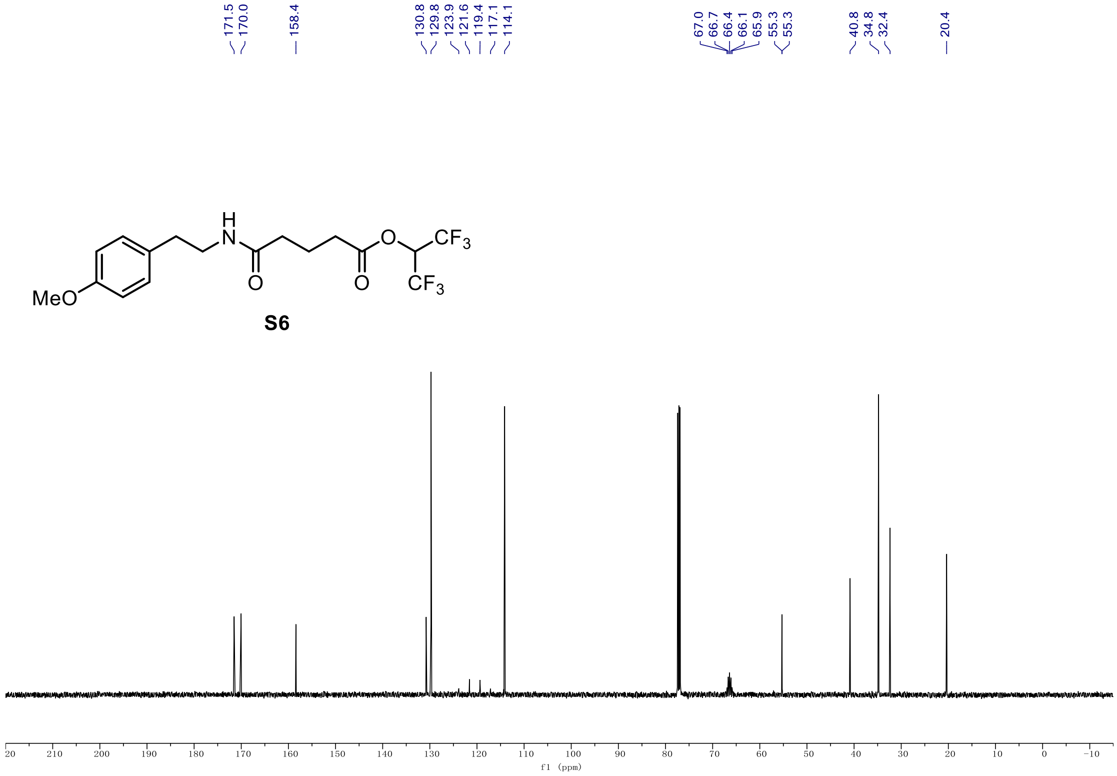

**$^{19}$F NMR** (471 MHz, $CDCl_3$) of **S6**.

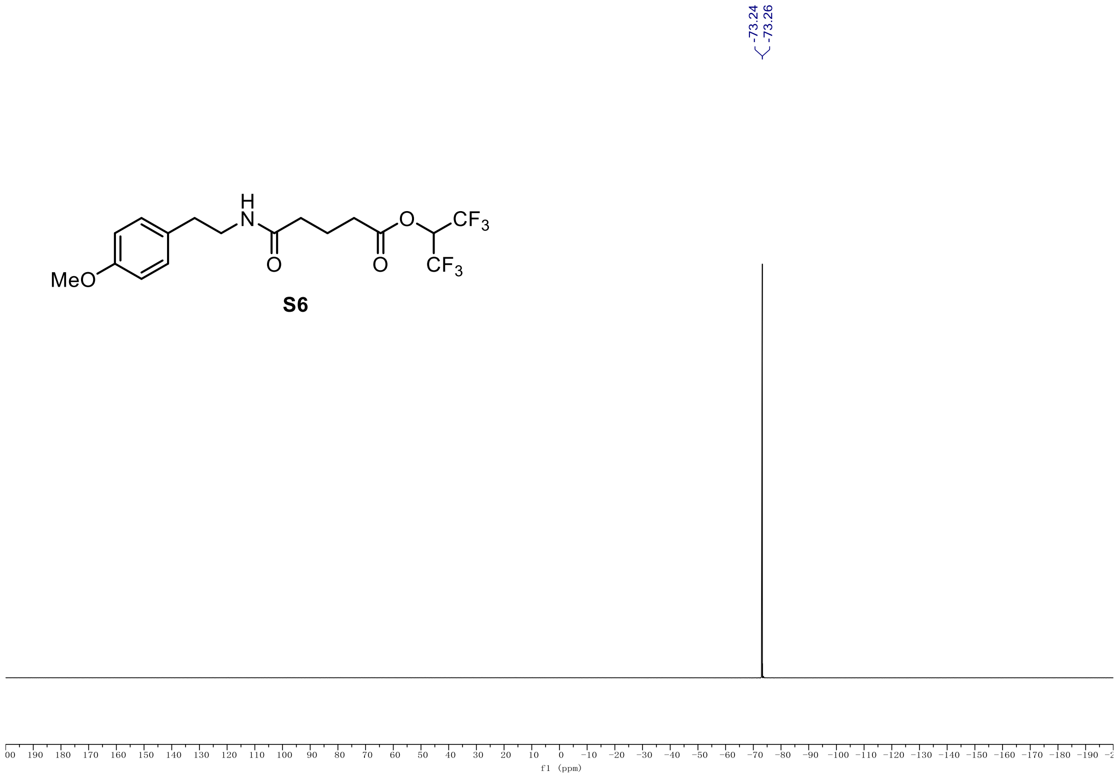


**$^{1}$H NMR** (500 MHz, $CDCl_3$) of **S7**.

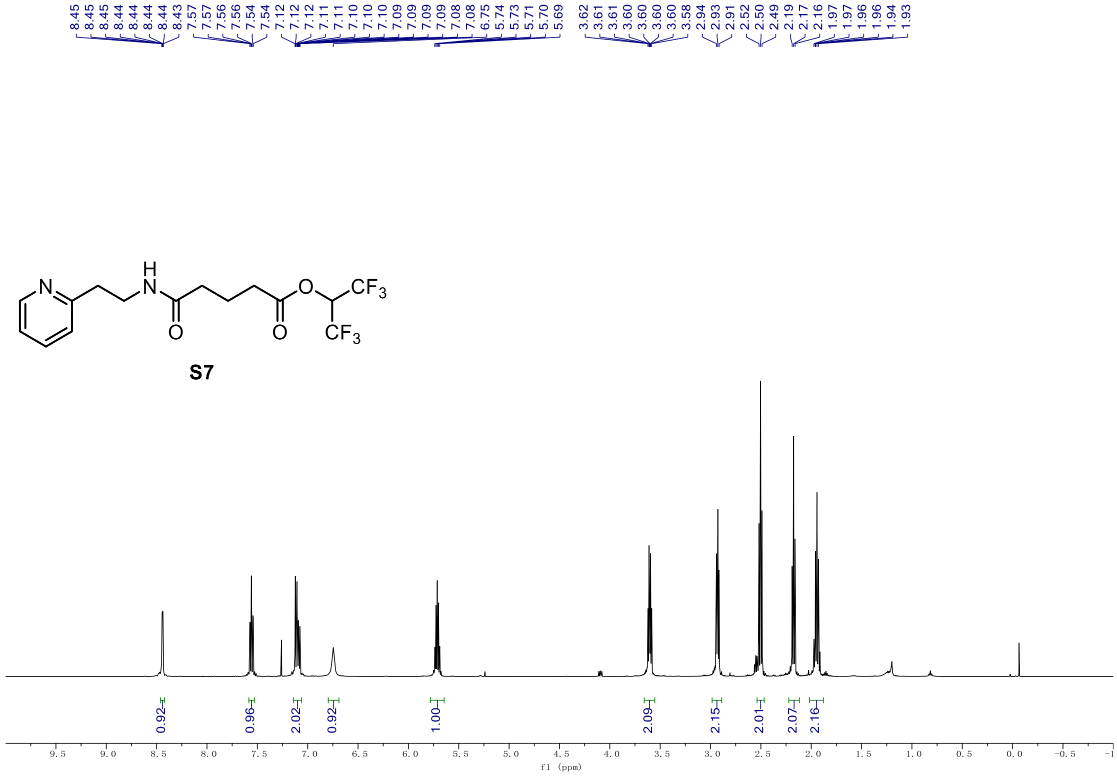

**$^{13}$C NMR** (126 MHz, $CDCl_3$) of **S7**.

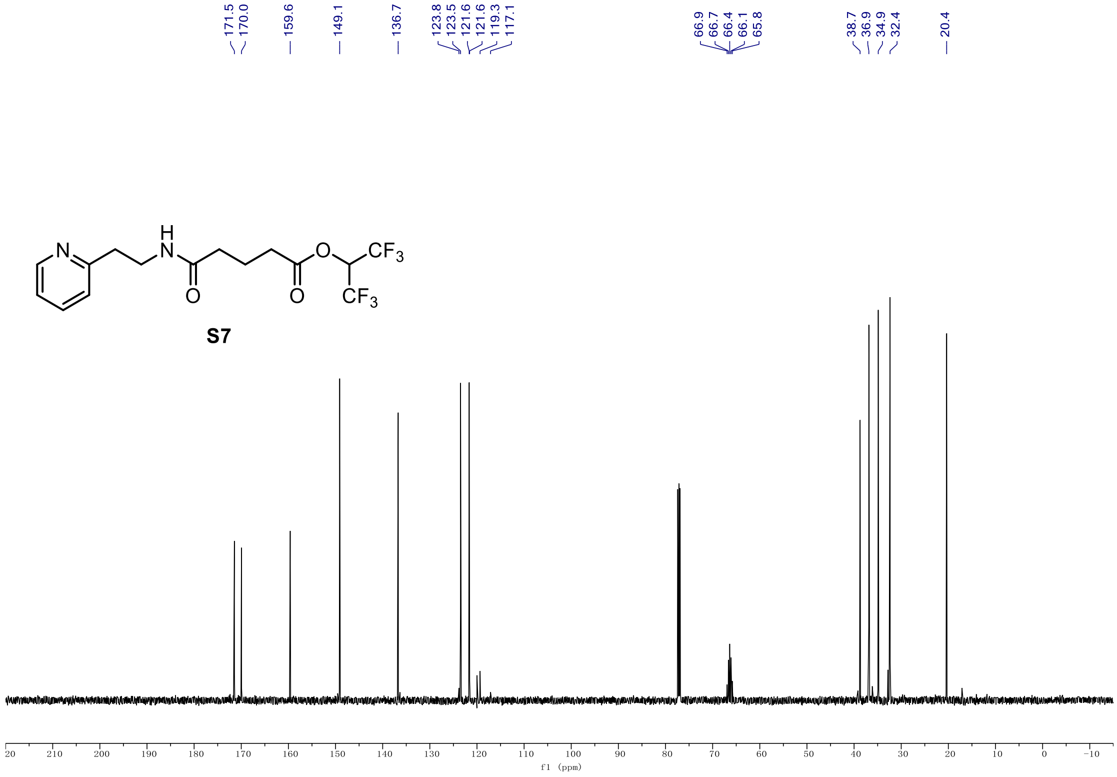


**$^{19}$F NMR** (471 MHz, $CDCl_3$) of **S7**.

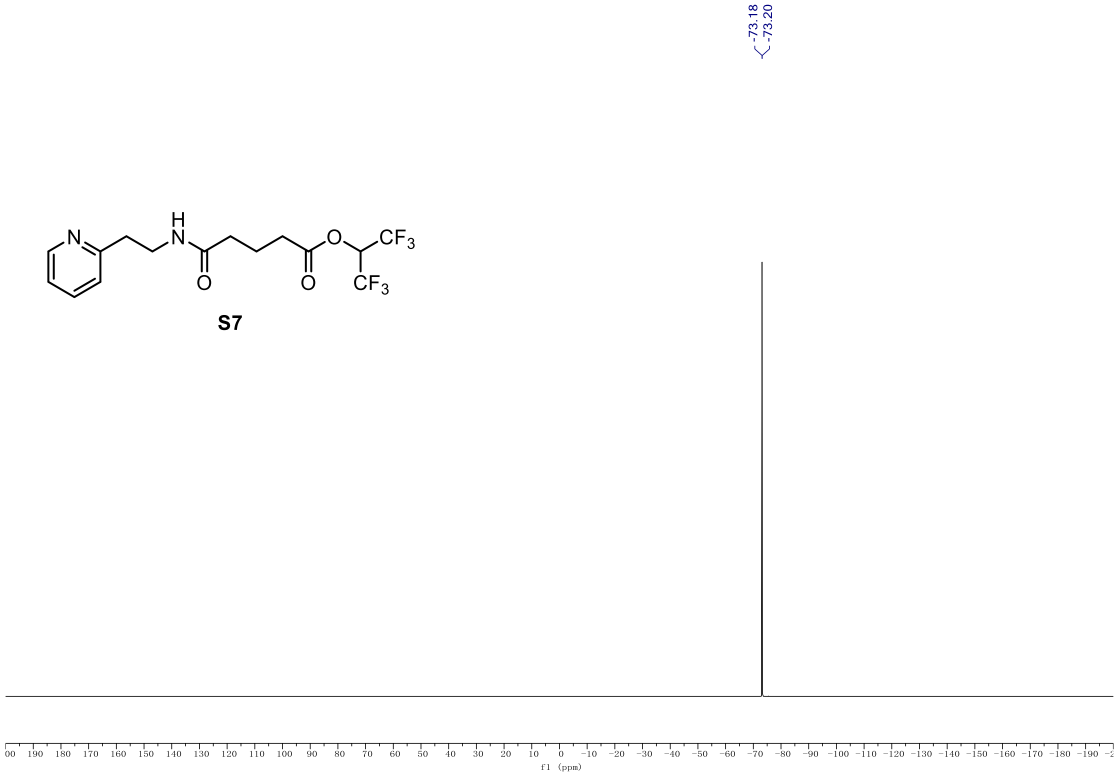

**$^{1}$H NMR** (500 MHz, $CDCl_3$) of **9**.

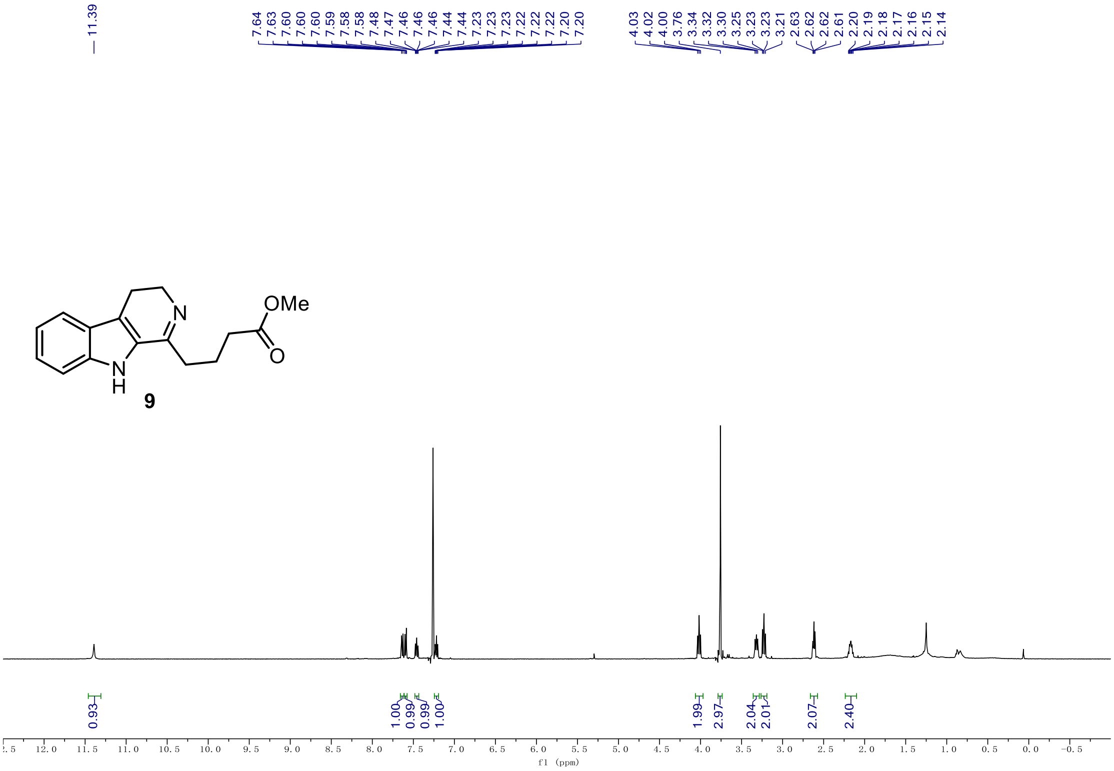


**$^{13}$C NMR** (126 MHz, $CDCl_3$) of **9**.

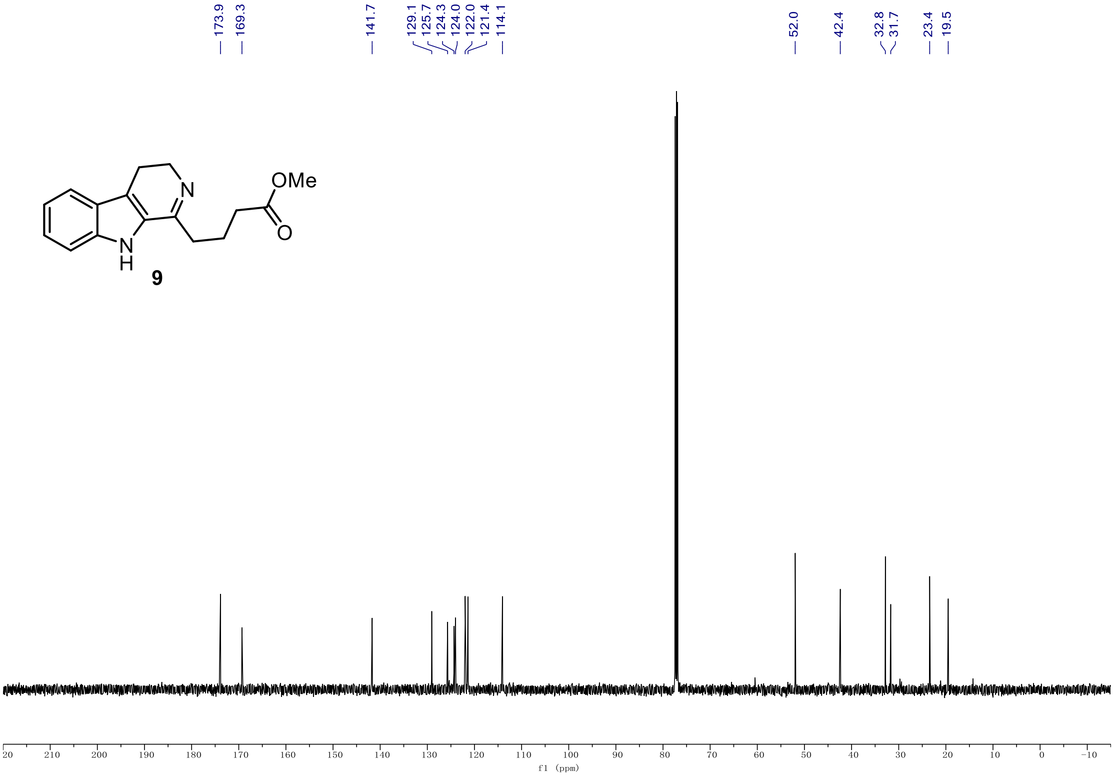

## $^{1}$H NMR (500 MHz, $CDCl_3$) of **10**.

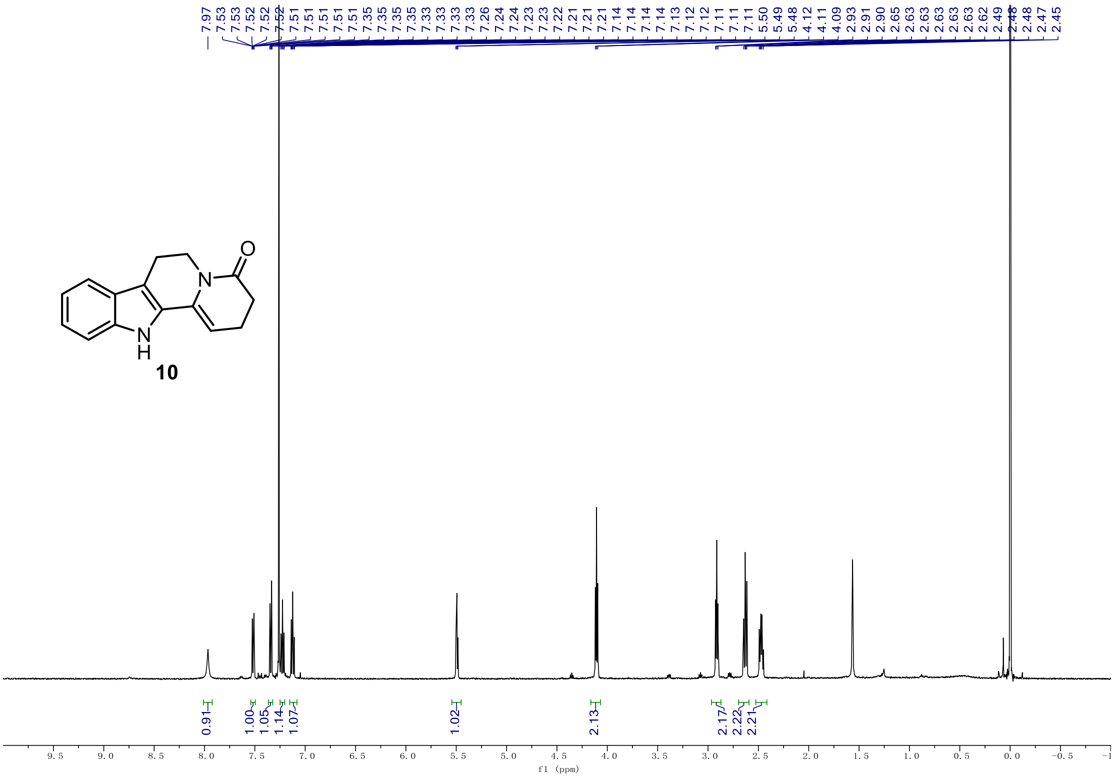

## $^{13}$C NMR (126 MHz, $CDCl_3$) of **10**.

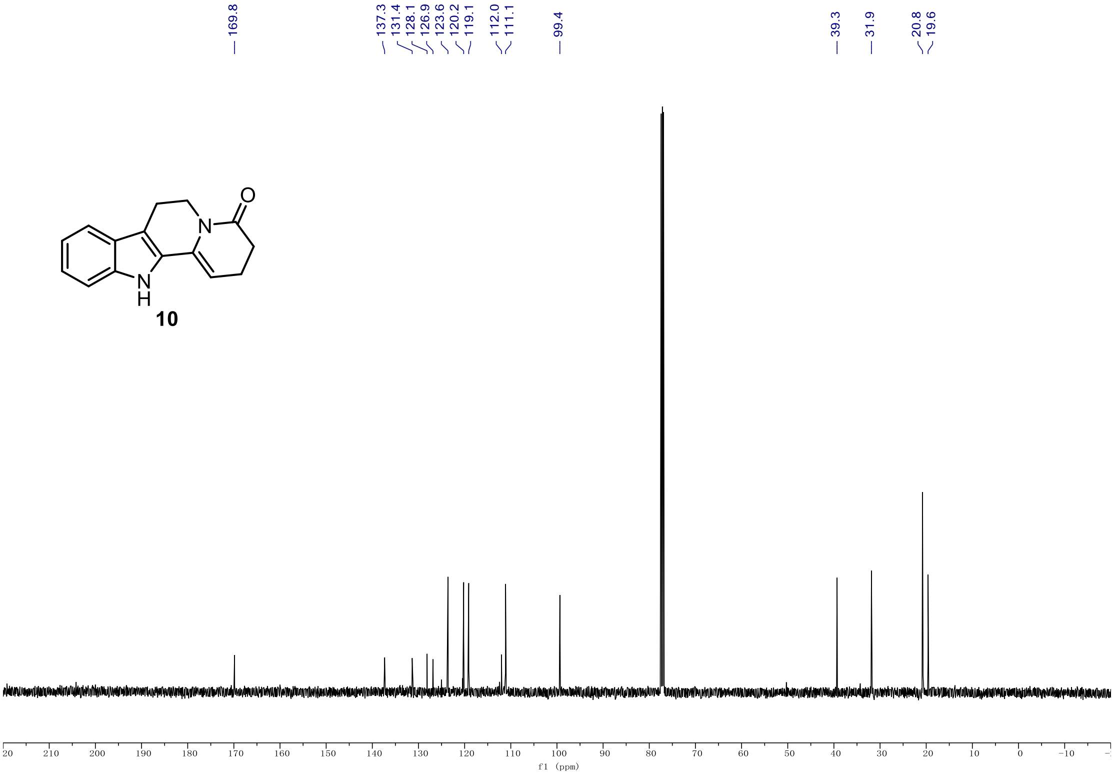

## $^{1}$H NMR (500 MHz, $CDCl_3$) of **12a**.

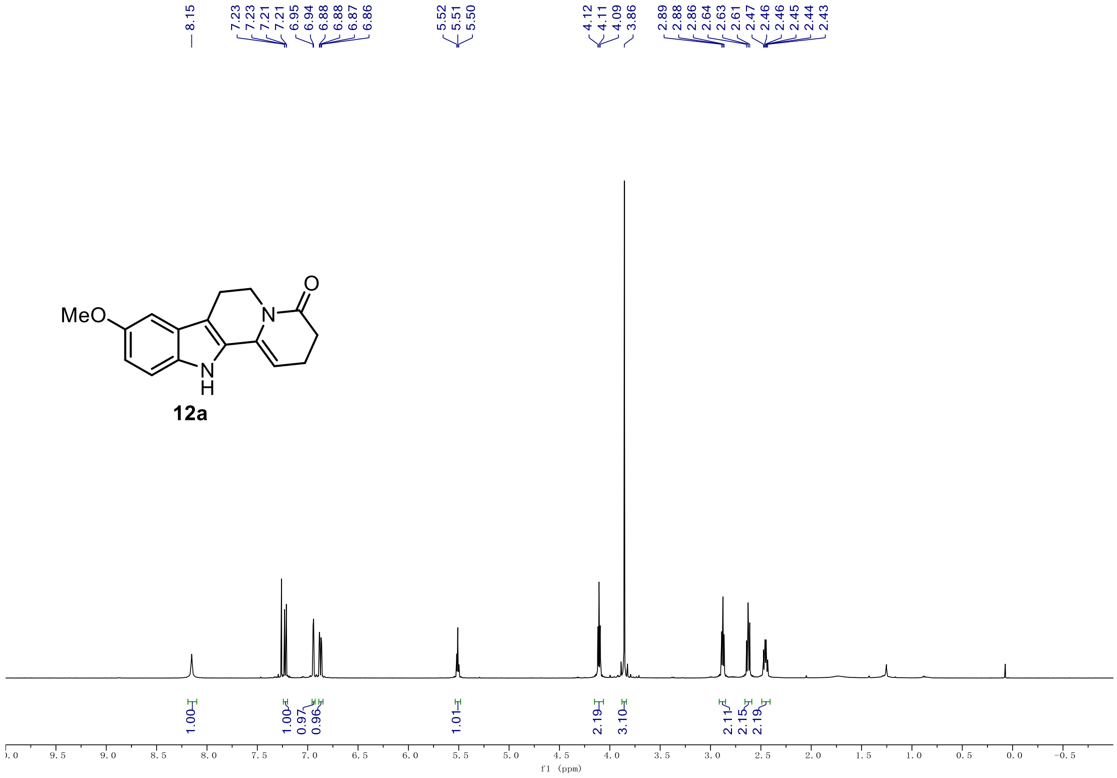


## $^{13}$C NMR (126 MHz, $CDCl_3$) of **12a**.

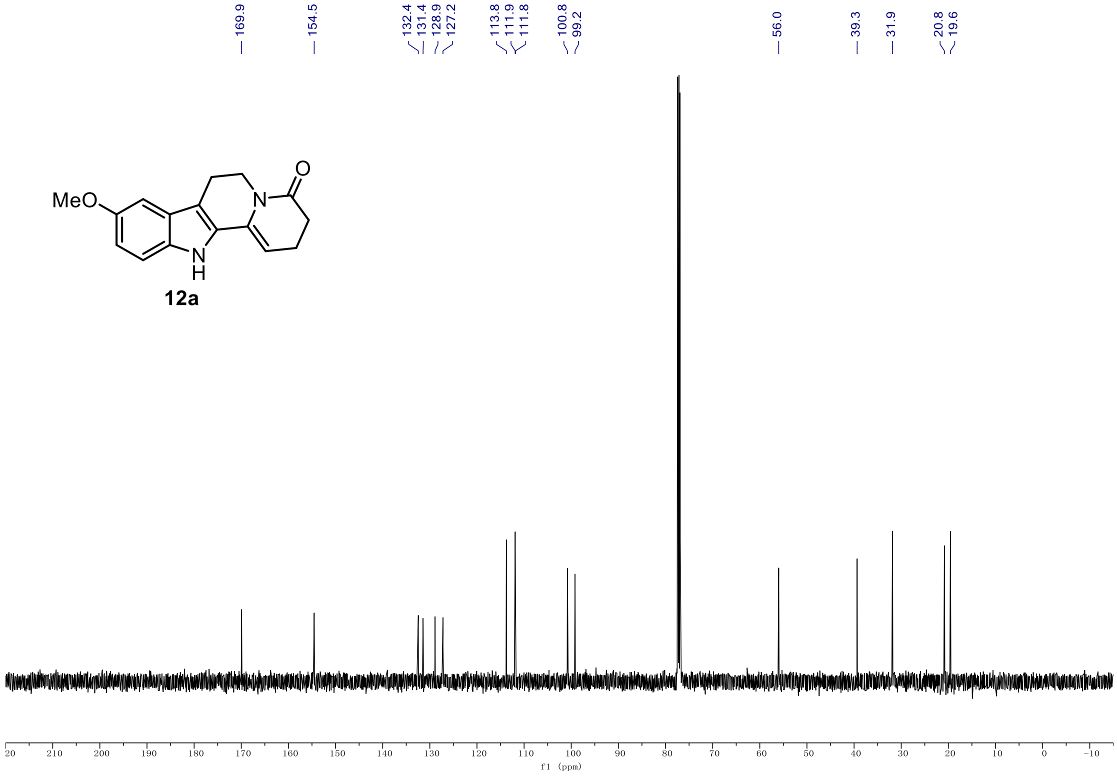

**$^{1}$H NMR** (500 MHz, $CD_2Cl_2$) of **12b**.

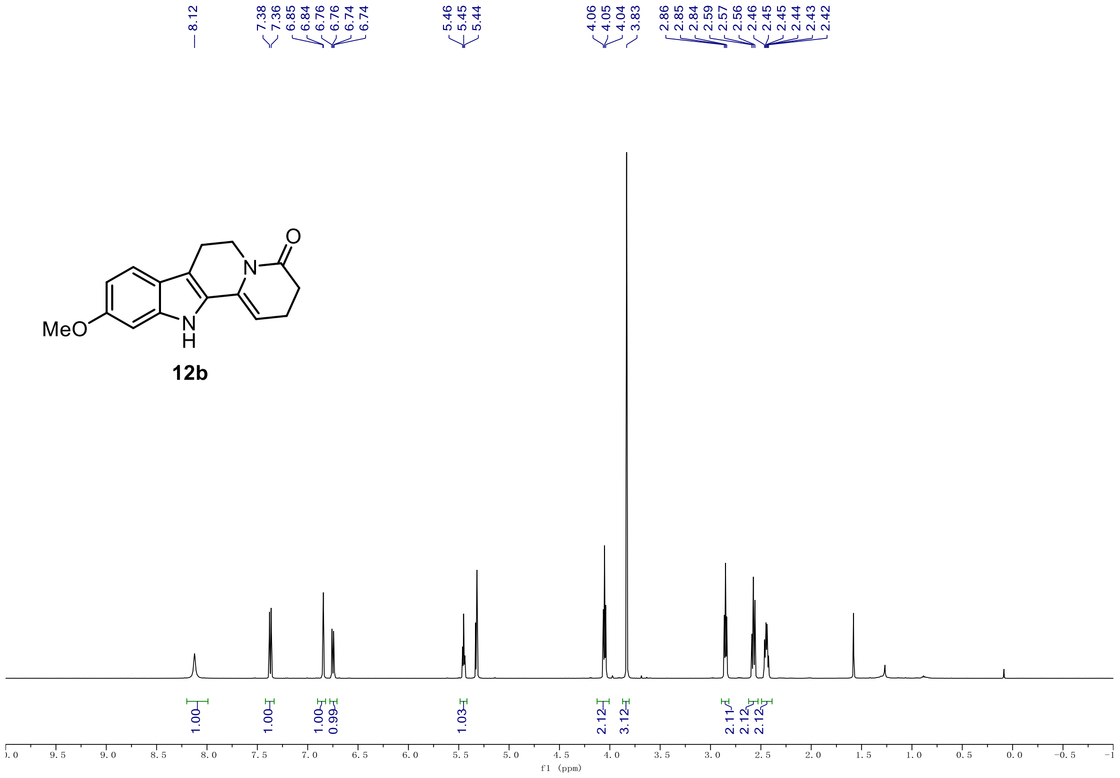


**$^{13}$C NMR** (126 MHz, $CD_2Cl_2$) of **12b**.

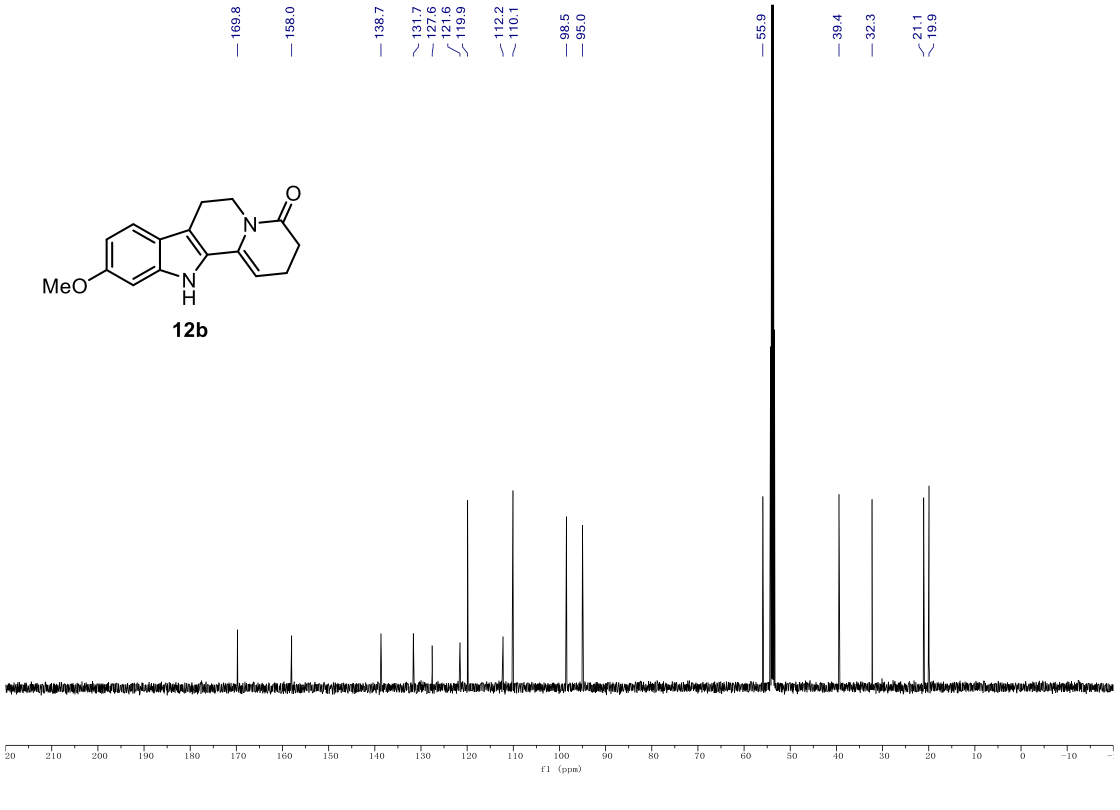

**$^{1}$H NMR** (700 MHz, $d_6$-DMSO) of **12c**.

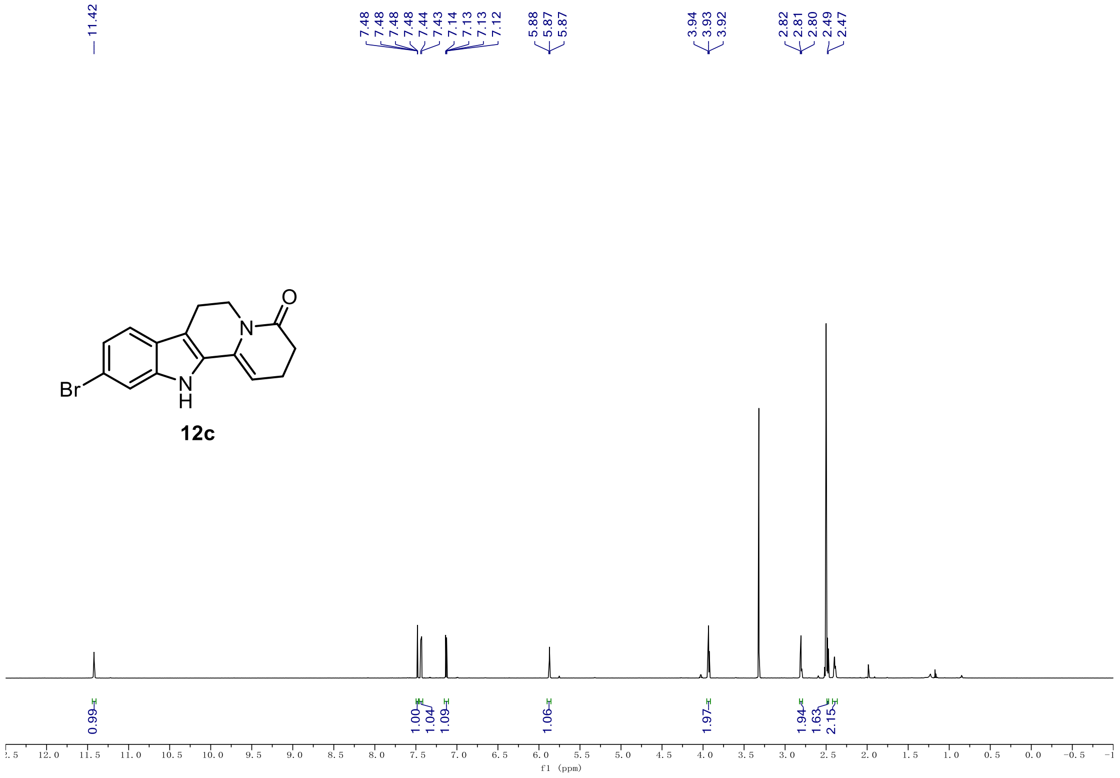


**$^{13}$C NMR** (176 MHz, $d_6$-DMSO) of **12c**.

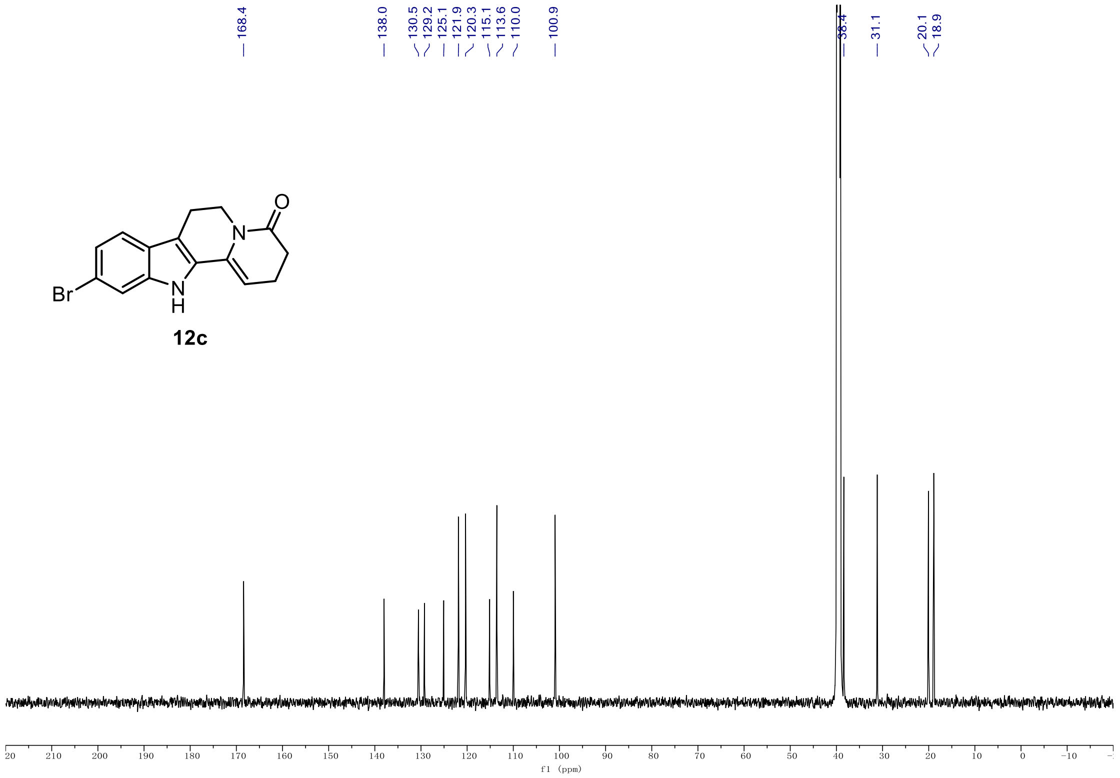

**$^{1}$H NMR** (700 MHz, $d_6$-DMSO) of **12d**.

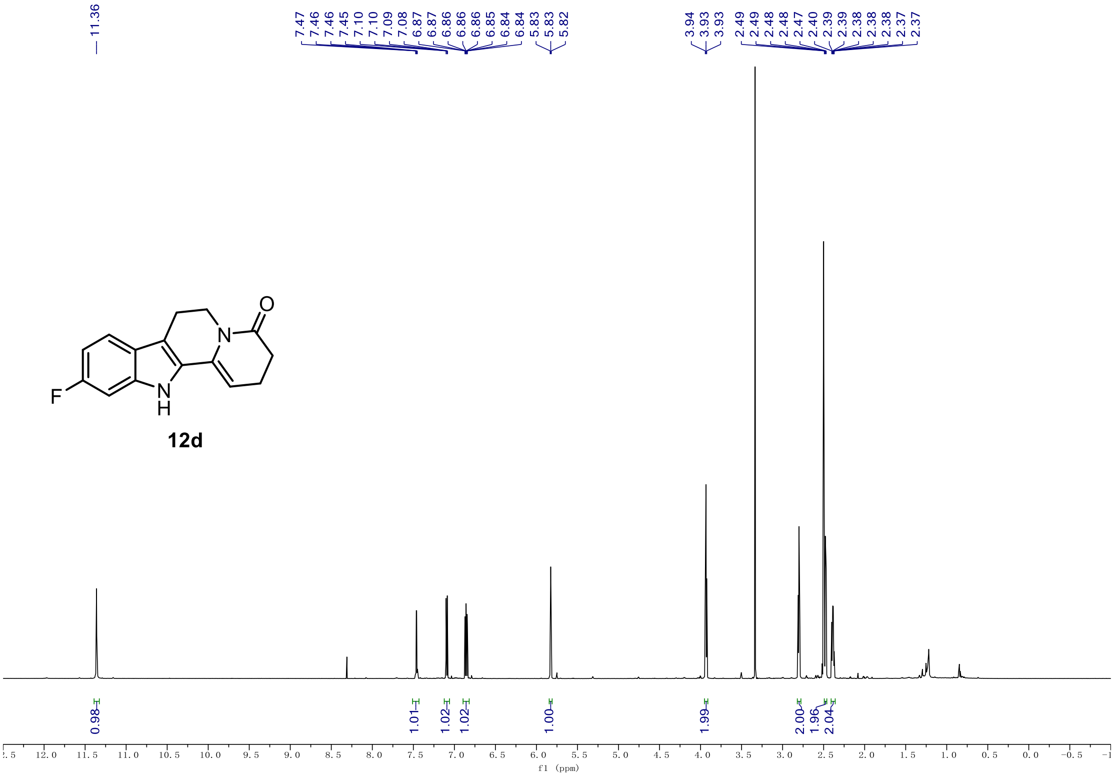

**$^{13}$C NMR** (176 MHz, $d_6$-DMSO) of **12d**.

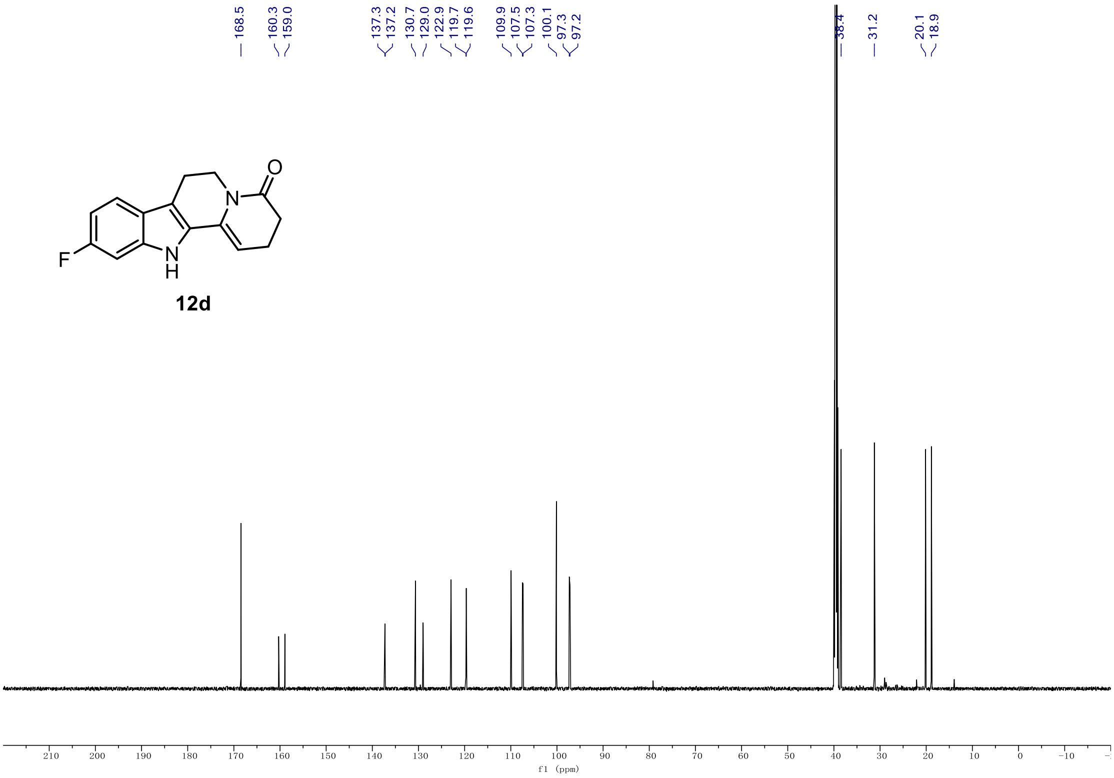

**$^{19}$F NMR** (471 MHz, $d_6$-DMSO) of **12d**.

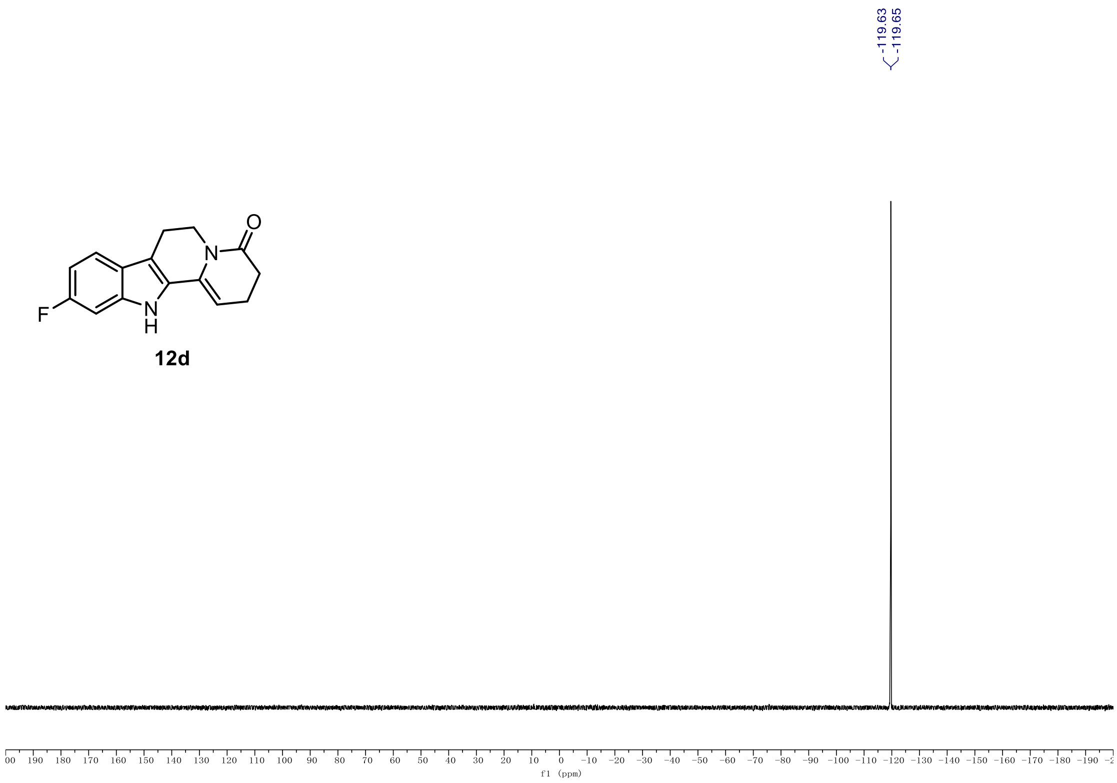


**12d**

**$^1$H NMR** (500 MHz, $CDCl_3$) of **12e**.

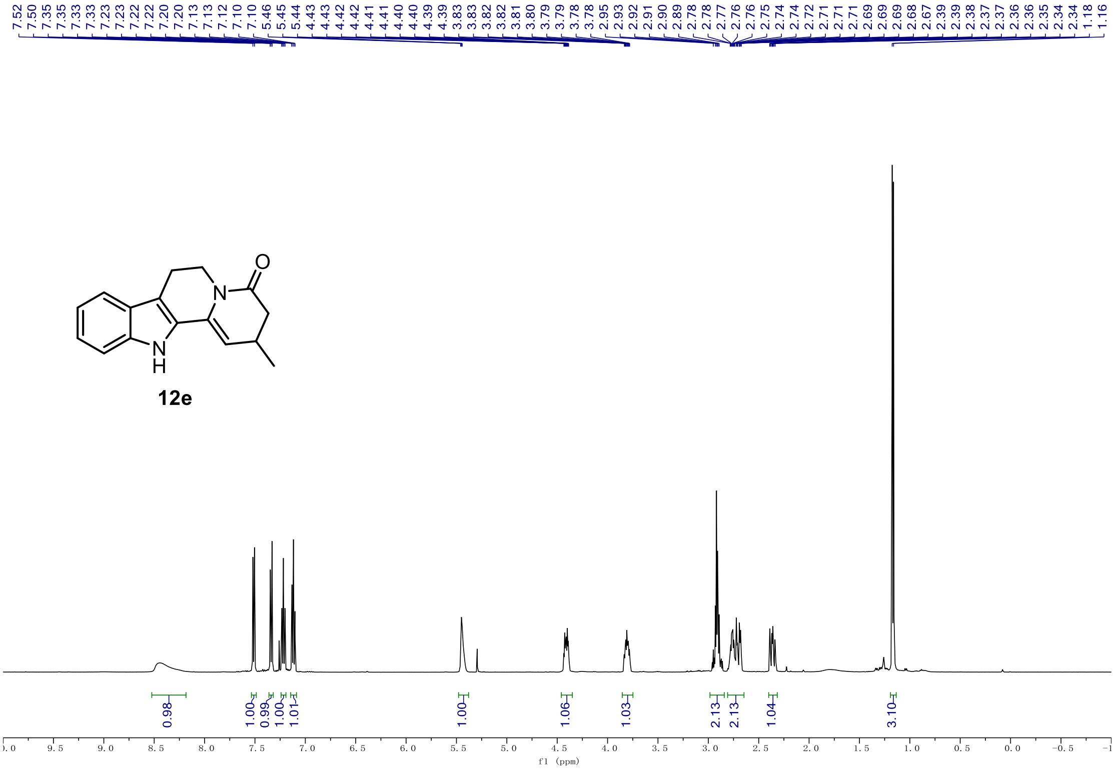

**$^{13}$C NMR** (126 MHz, $CDCl_3$) of **12e**.

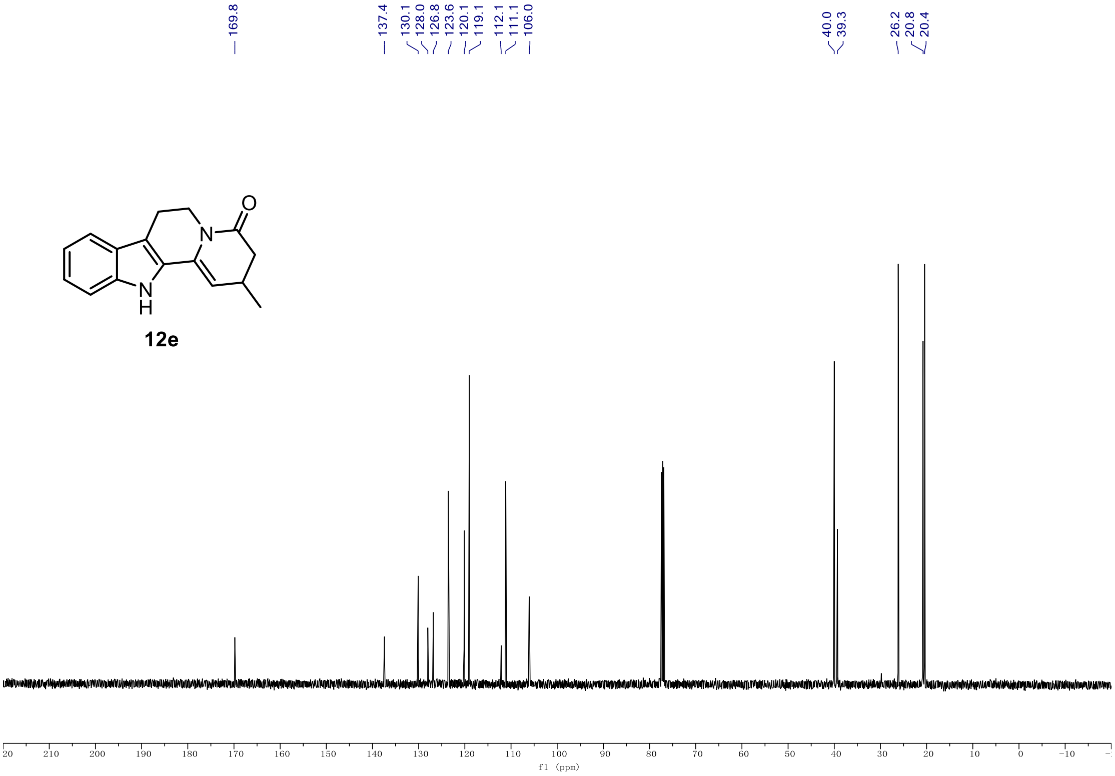

## $^{1}$H NMR (700 MHz, $CD_2Cl_2$) of **12f**.

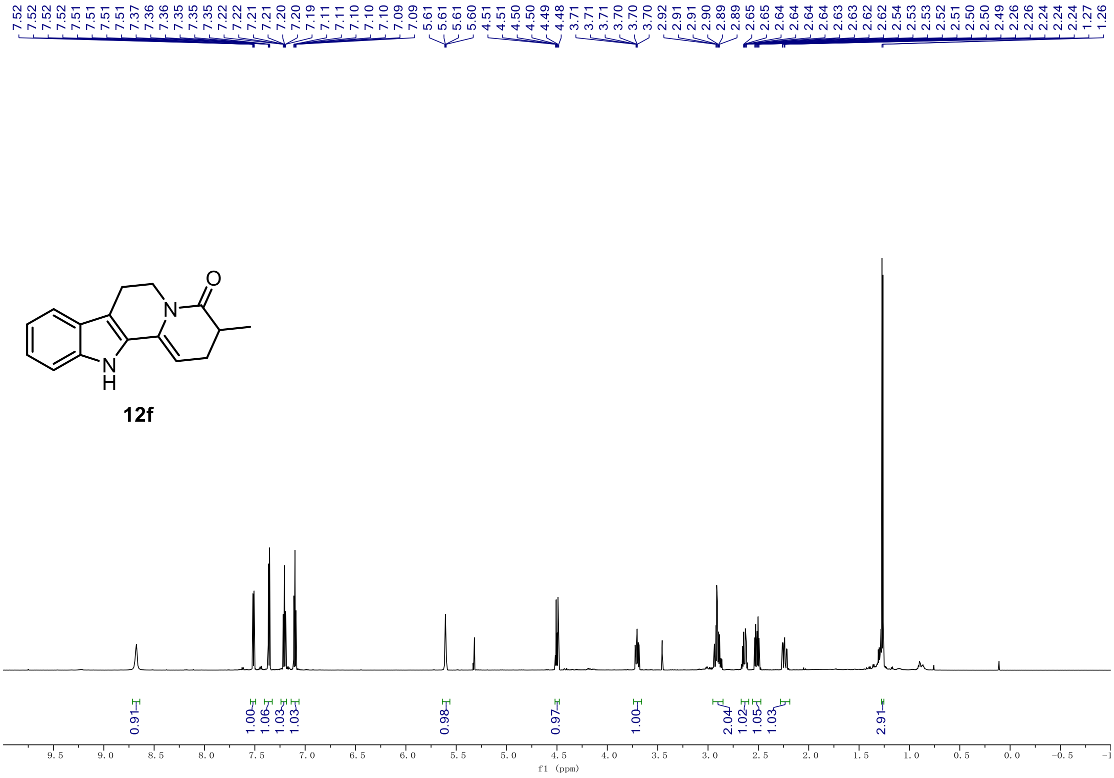


## $^{13}$C NMR (176 MHz, $CD_2Cl_2$) of **12f**.

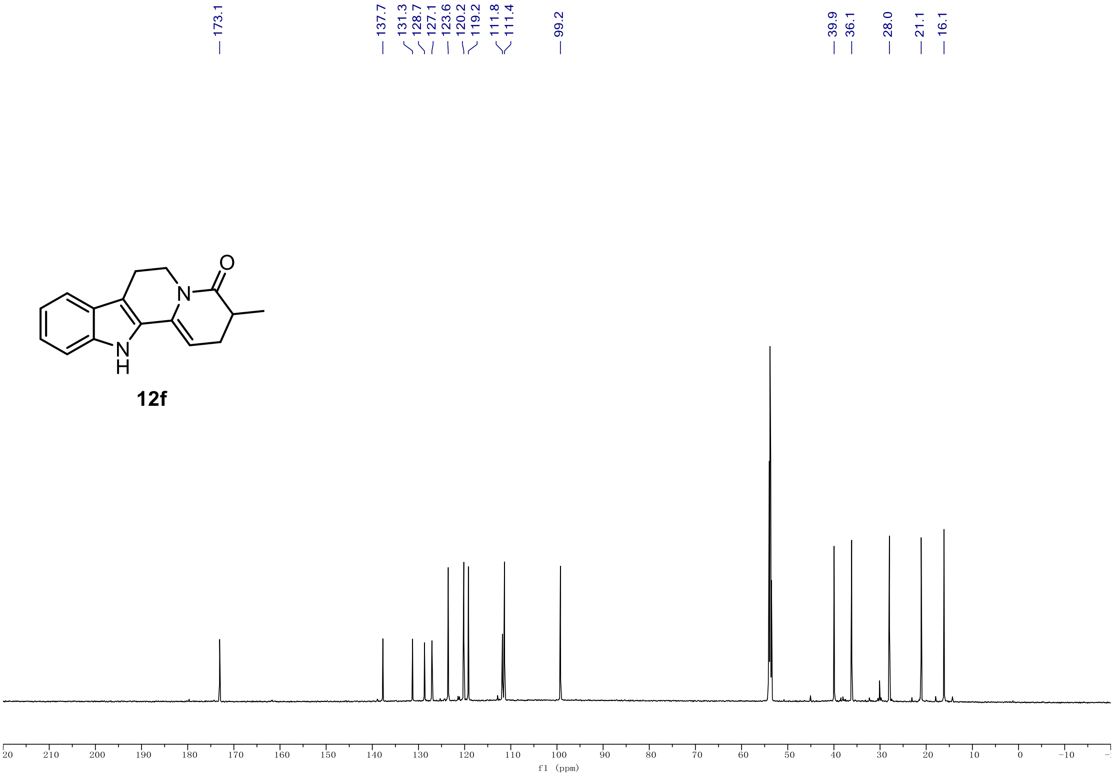

## $^{1}$H NMR (700 MHz, $CD_2Cl_2$) of 12g.

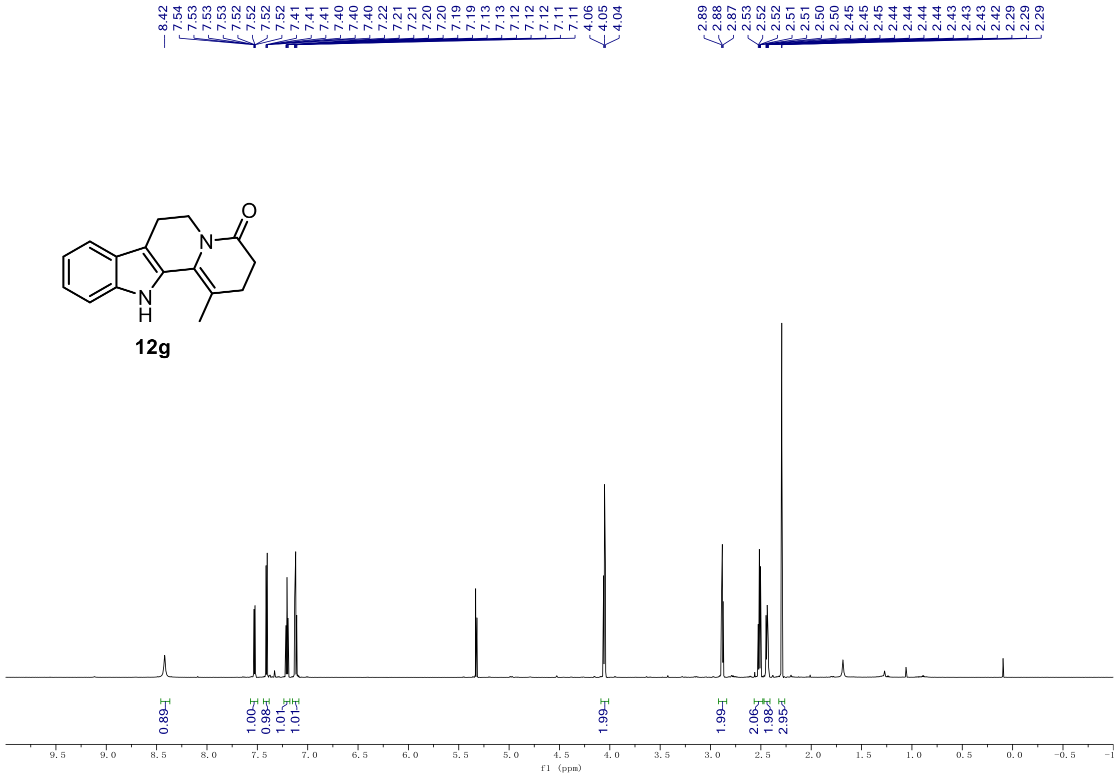

## $^{13}$C NMR (176 MHz, $CD_2Cl_2$) of 12g.

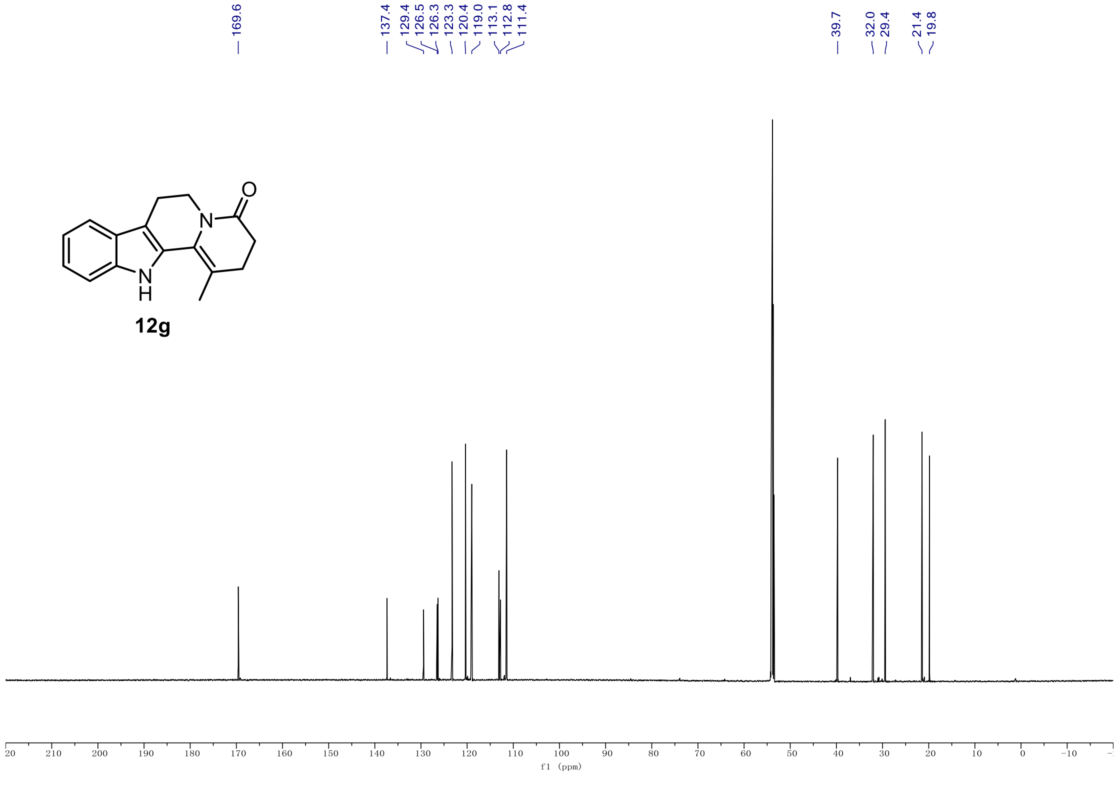

$^{1}$**H NMR** (500 MHz, $CDCl_3$) of **12h**.

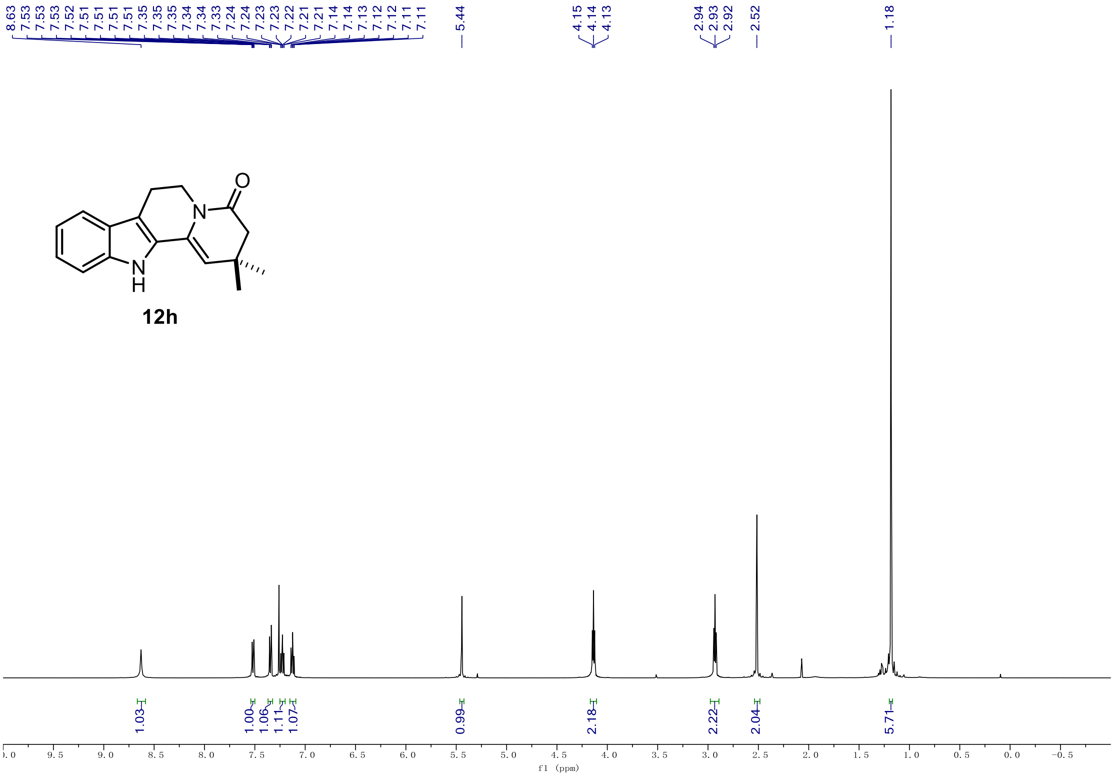


$^{13}$**C NMR** (126 MHz, $CDCl_3$) of **12h**.

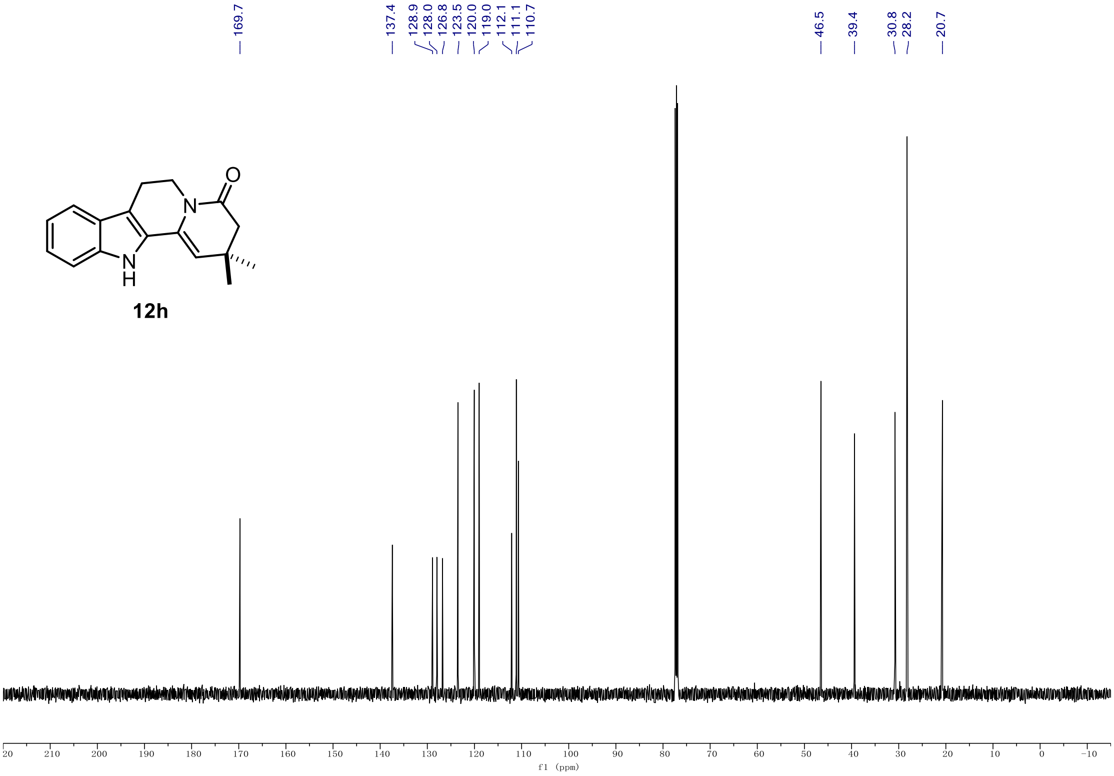

$^{1}$**H NMR** (700 MHz, $CDCl_3$) of **12i**.

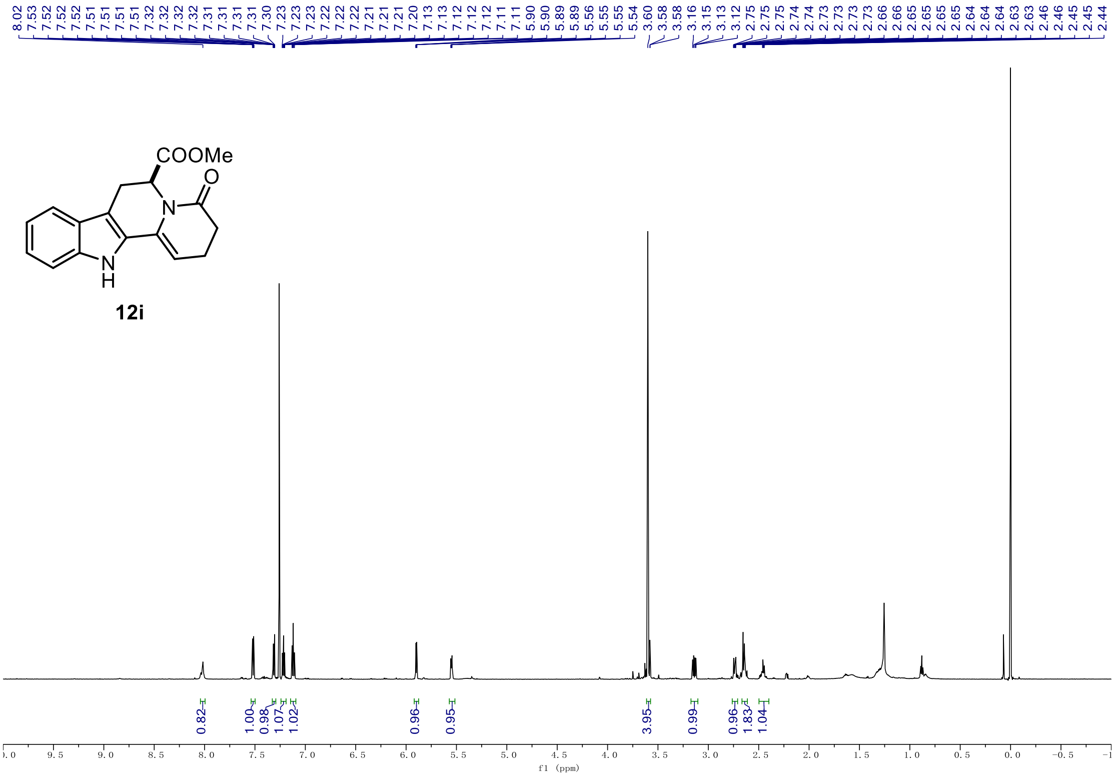


$^{13}$**C NMR** (176 MHz, $CDCl_3$) of **12i**.

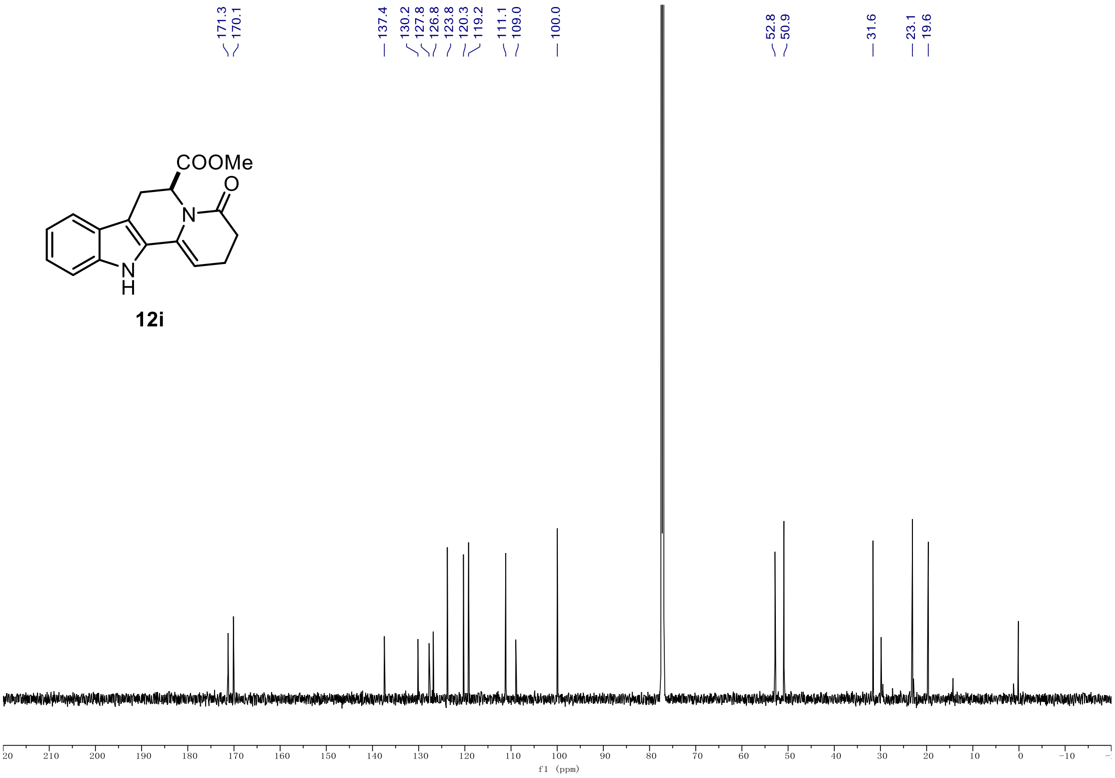

**$^{1}$H NMR** (500 MHz, $d_6$-DMSO) of **12j**.

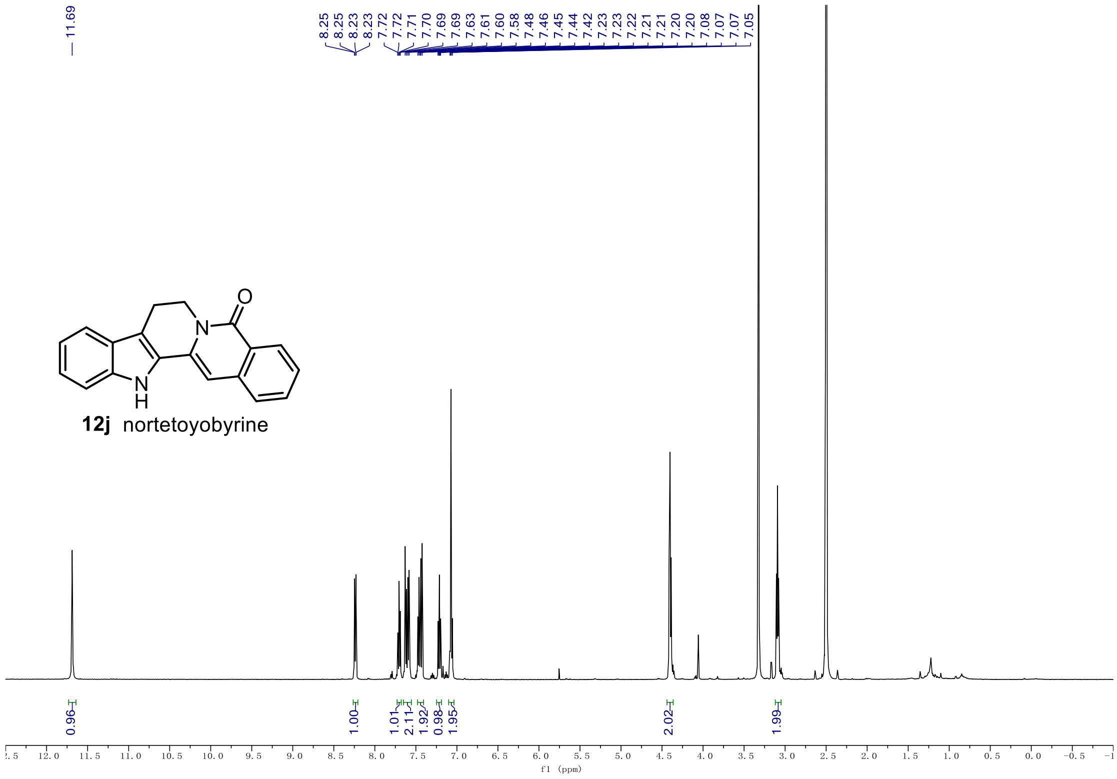


**$^{13}$C NMR** (126 MHz, $d_6$-DMSO) of **12j**.

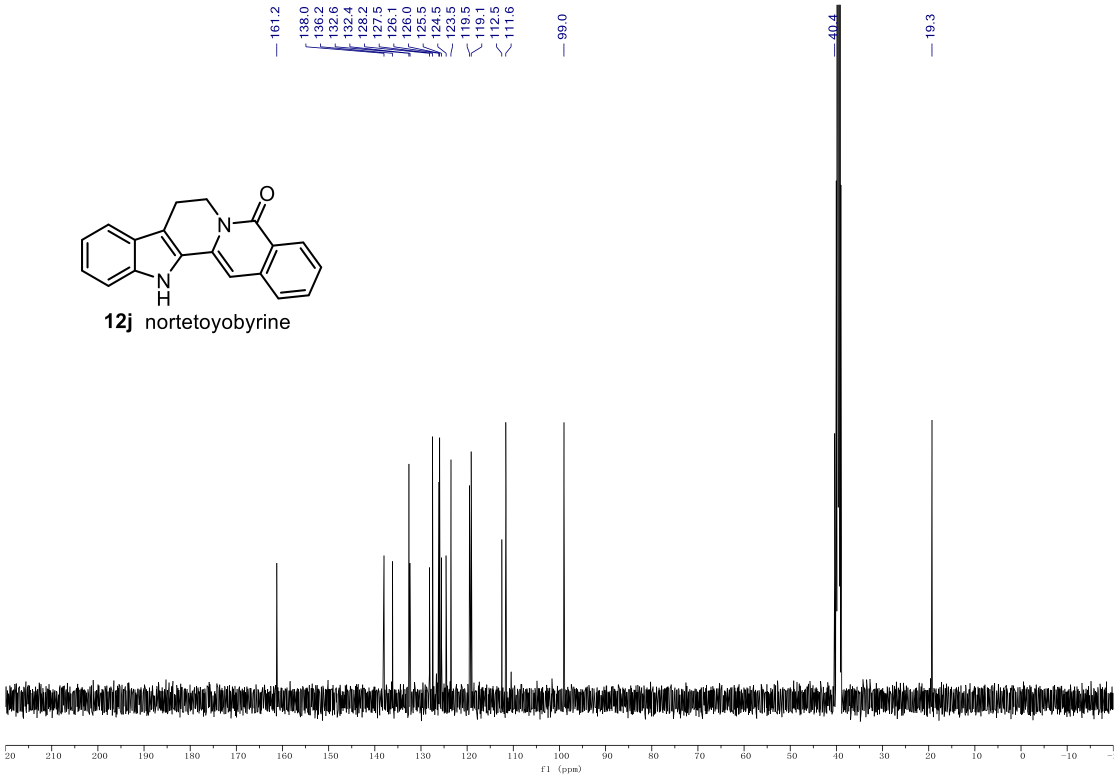

**$^{1}H$ NMR** (700 MHz, $CD_2Cl_2$) of **12k**.

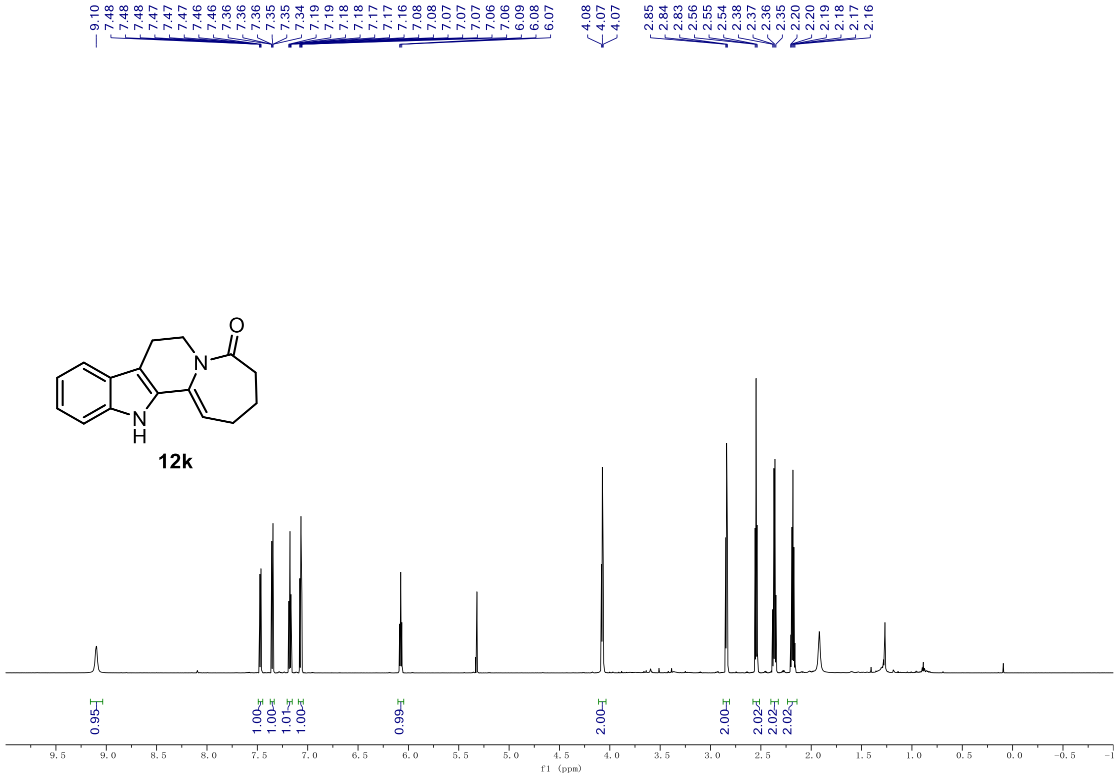


**$^{13}C$ NMR** (176 MHz, $CD_2Cl_2$) of **12k.**

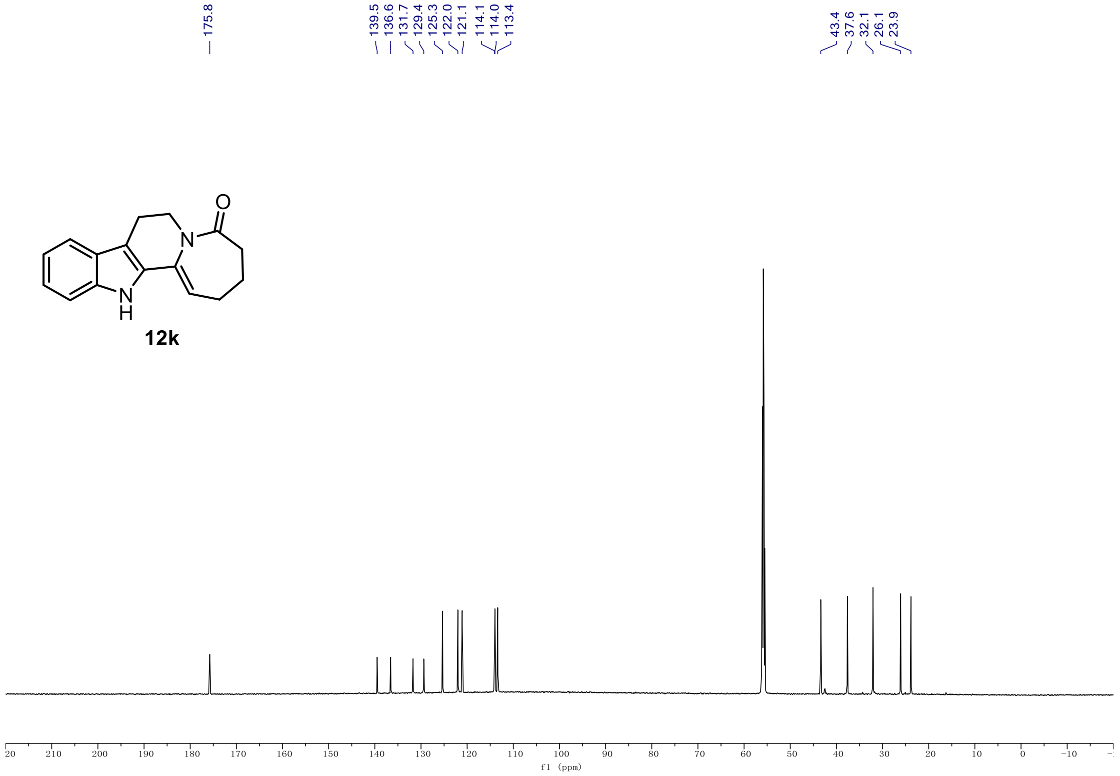

## $^{1}$H NMR (700 MHz, $CD_2Cl_2$) of **12l**.

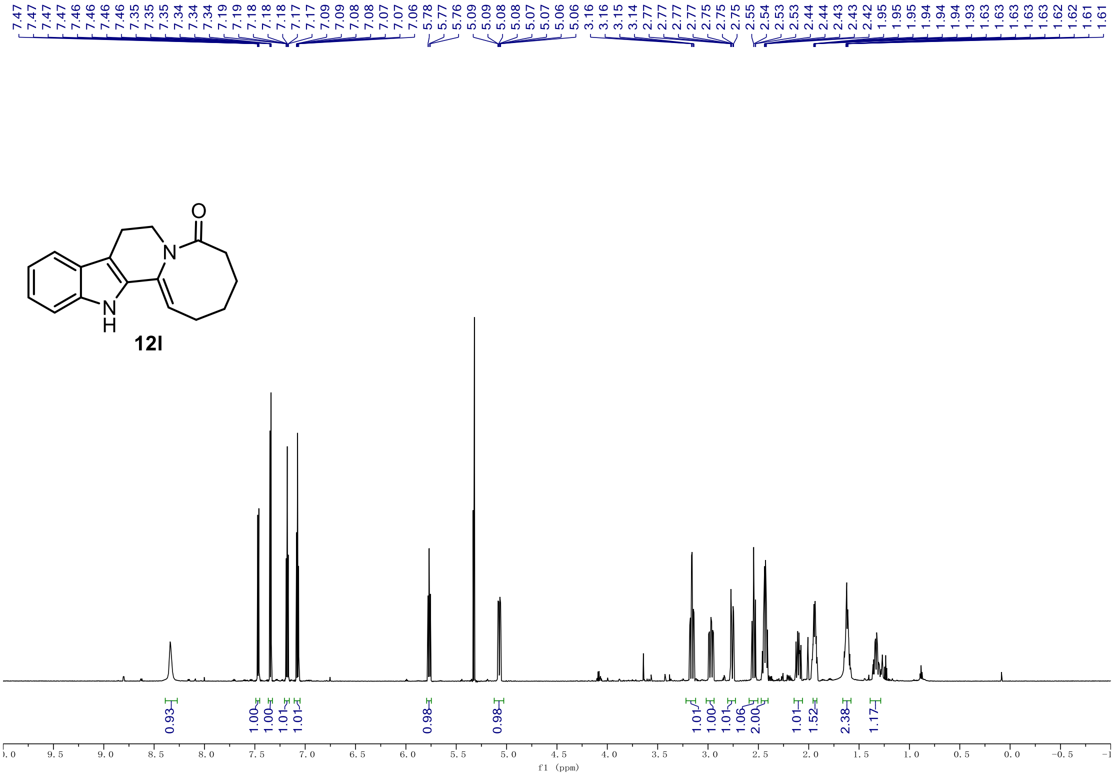


## $^{13}$C NMR (176 MHz, $CD_2Cl_2$) of **12l**.

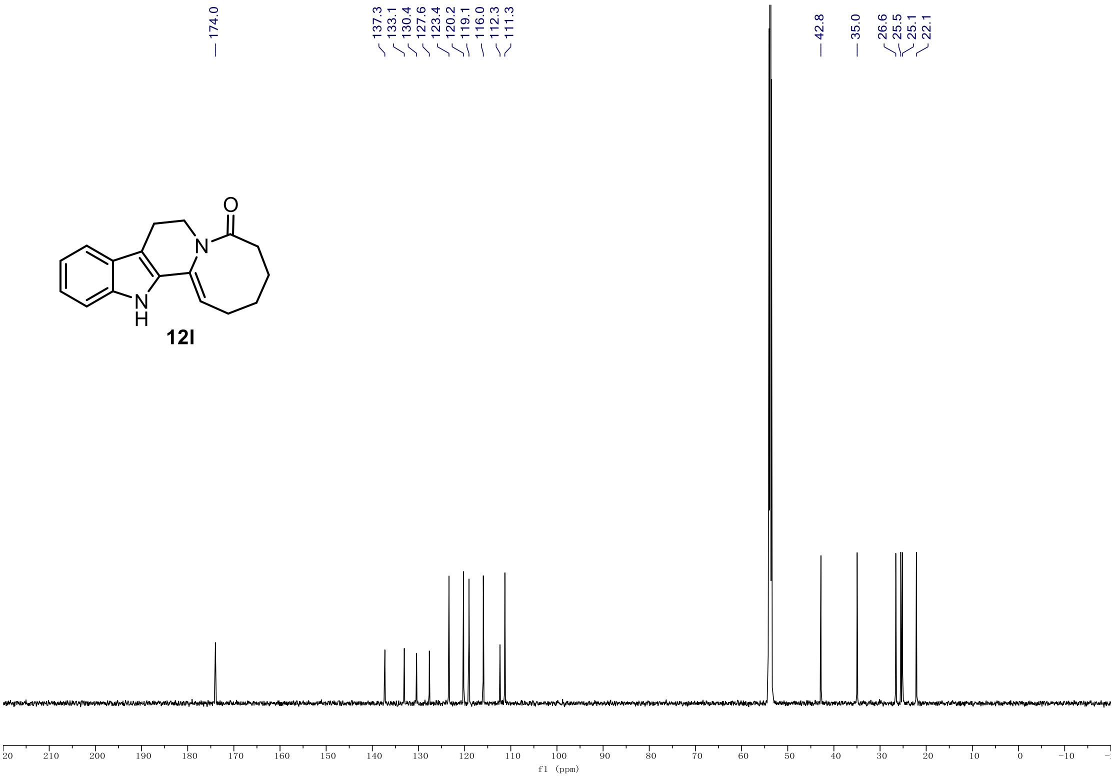

[1]**H NMR** (700 MHz, $CDCl_3$) of **12m**.

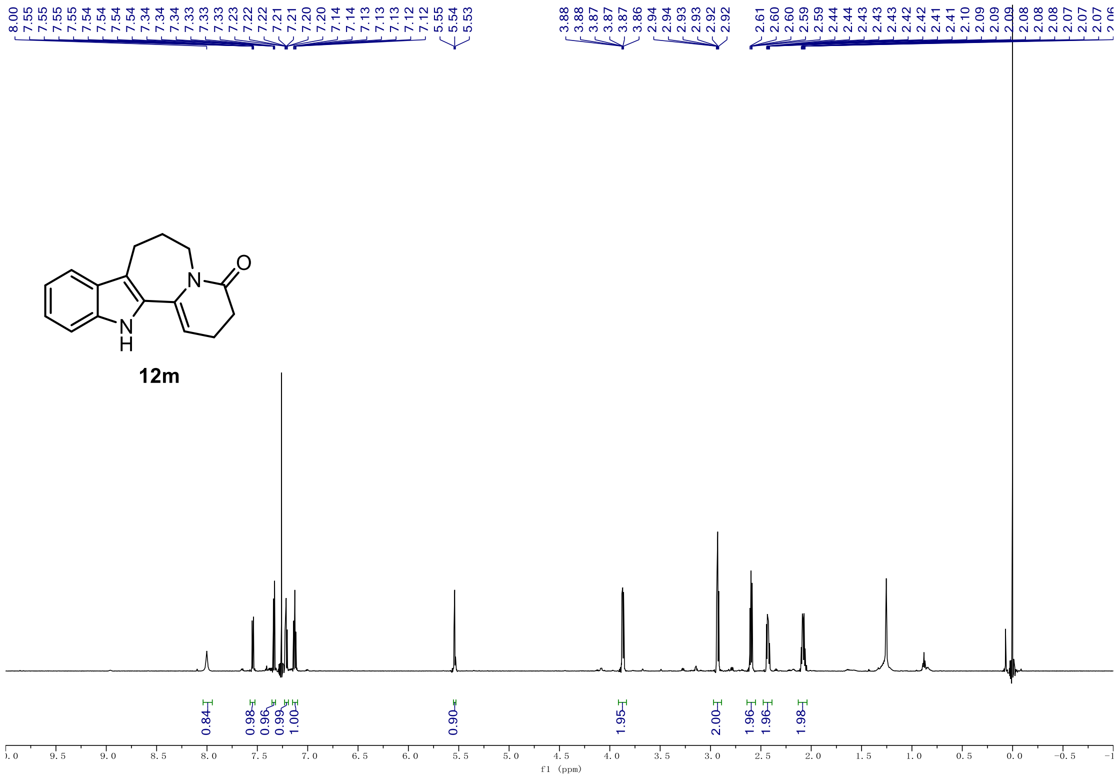


[13]**C NMR** (176 MHz, $CD_2Cl_2$) of **12m**.

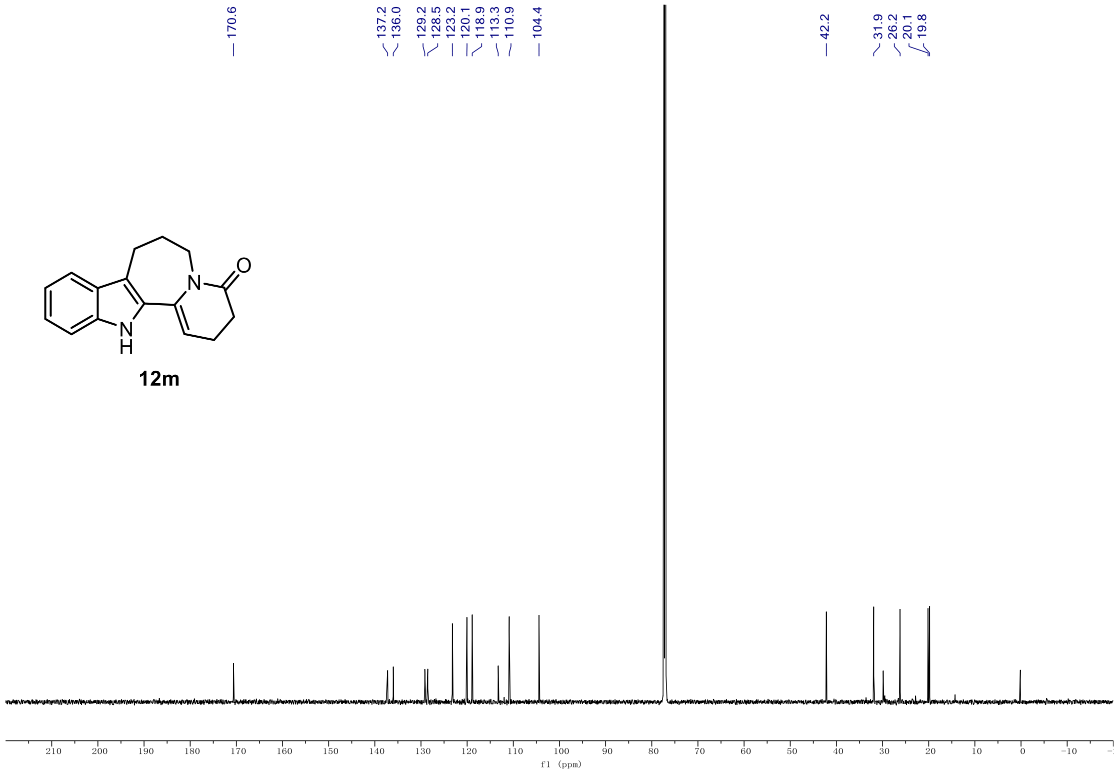

## $^{1}$H NMR (700 MHz, $CDCl_3$) of **12n.**

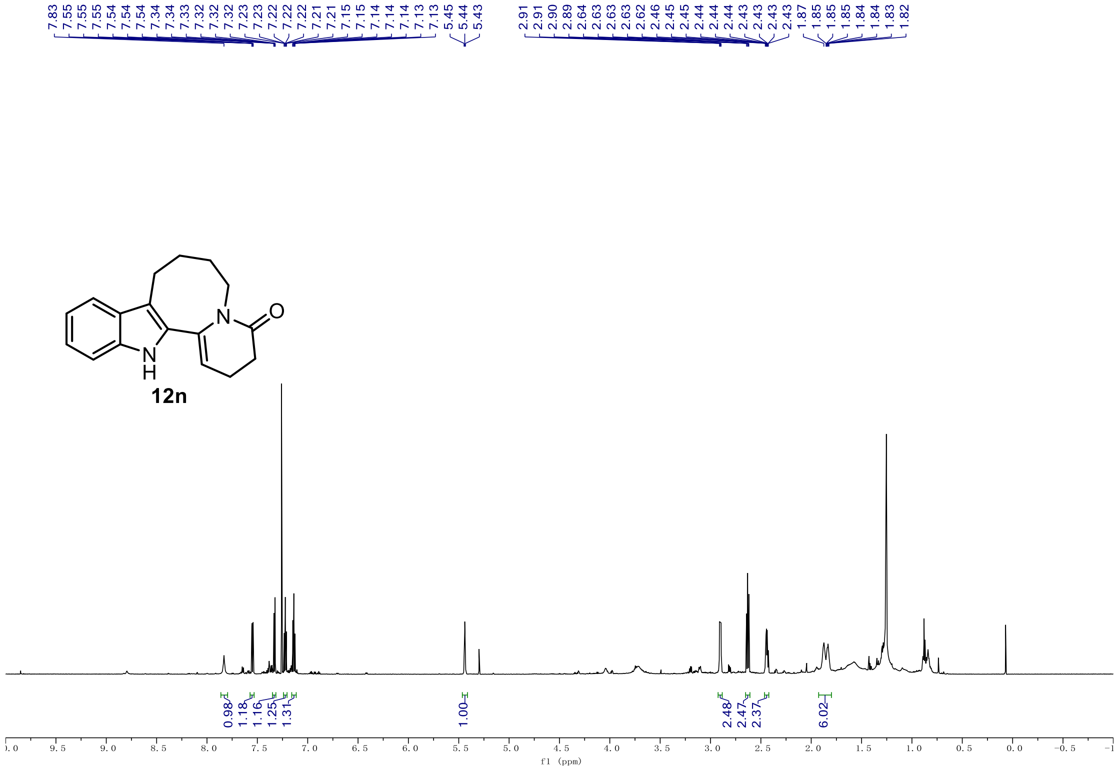


## $^{13}$C NMR (176 MHz, $CDCl_3$) of **12n**.

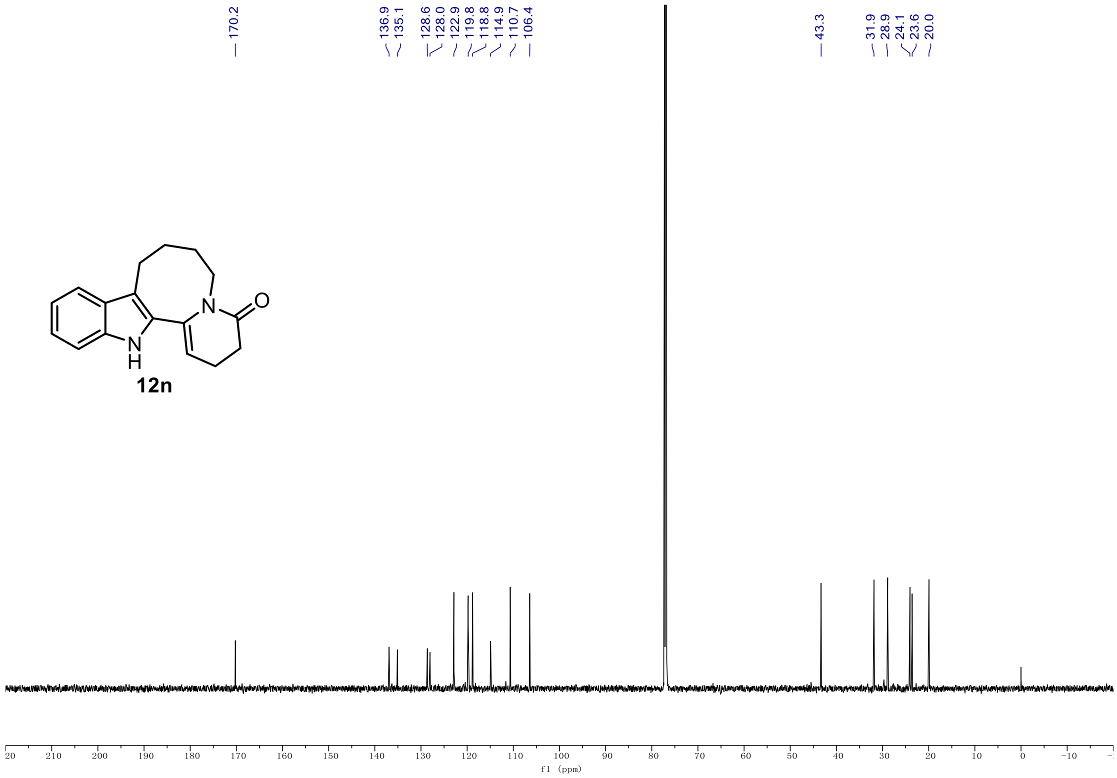

**$^{1}$H NMR** (700 MHz, $C_6D_6$) of **12o.**

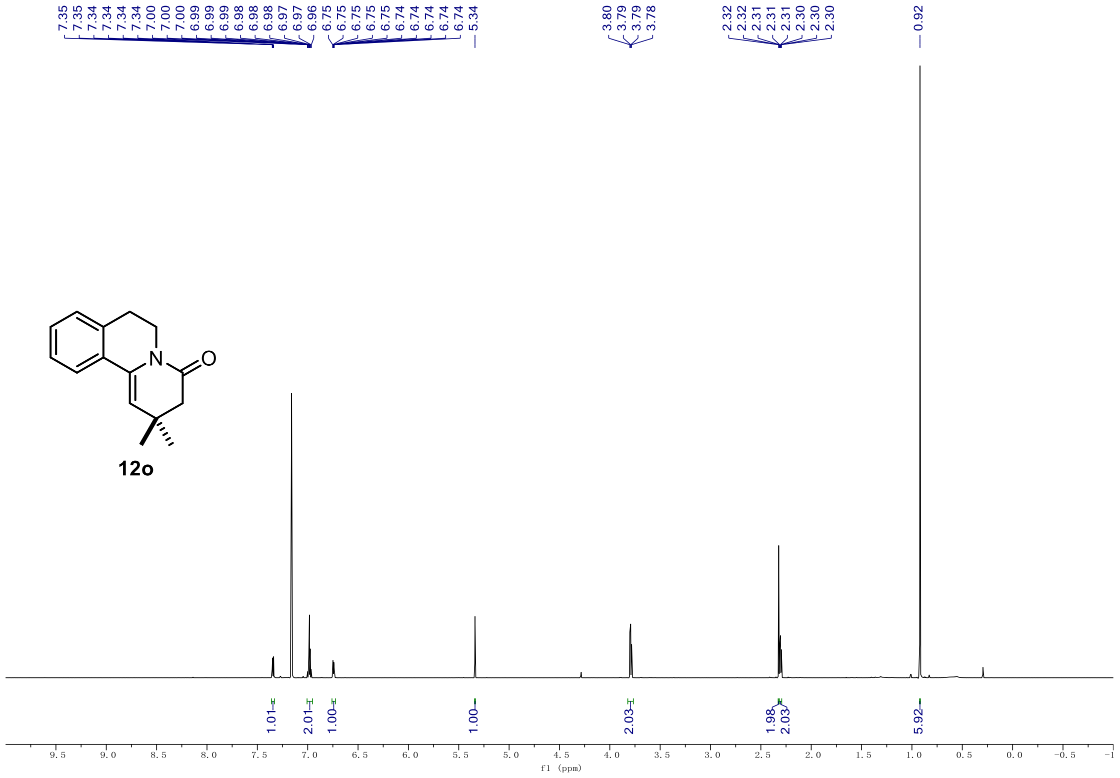


**$^{13}$C NMR** (176 MHz, $C_6D_6$) of **12o.**

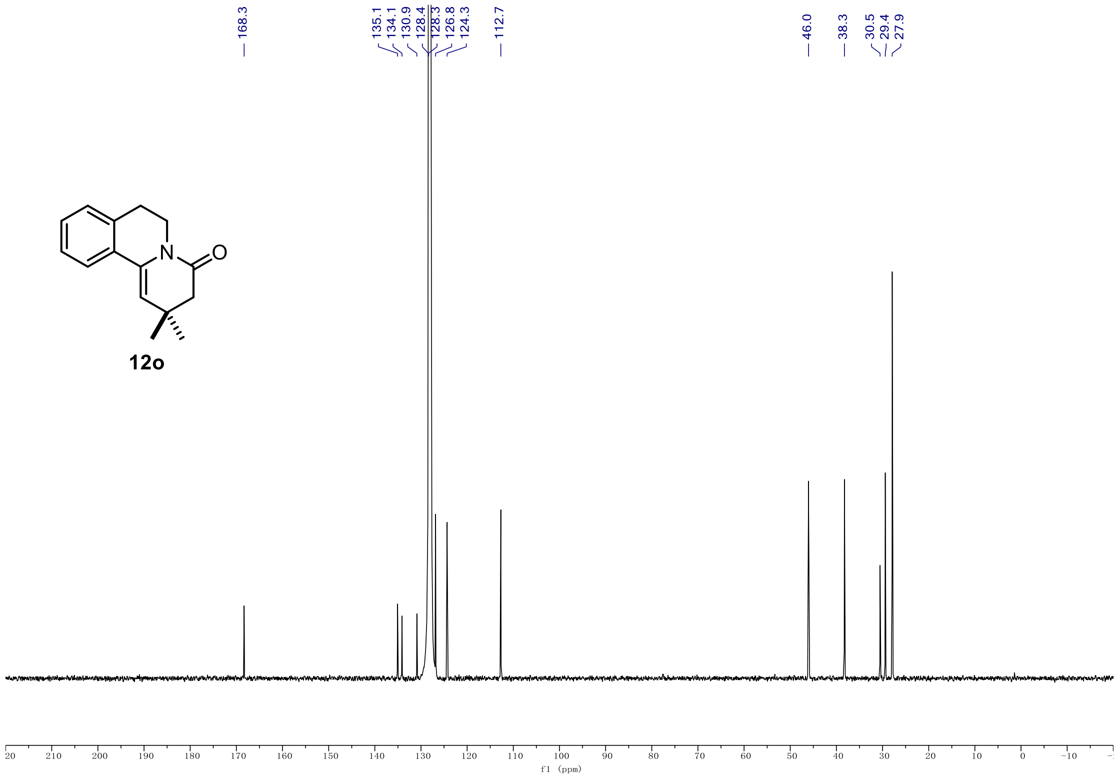

**$^{1}$H NMR** (700 MHz, $C_6D_6$) of **12p.**

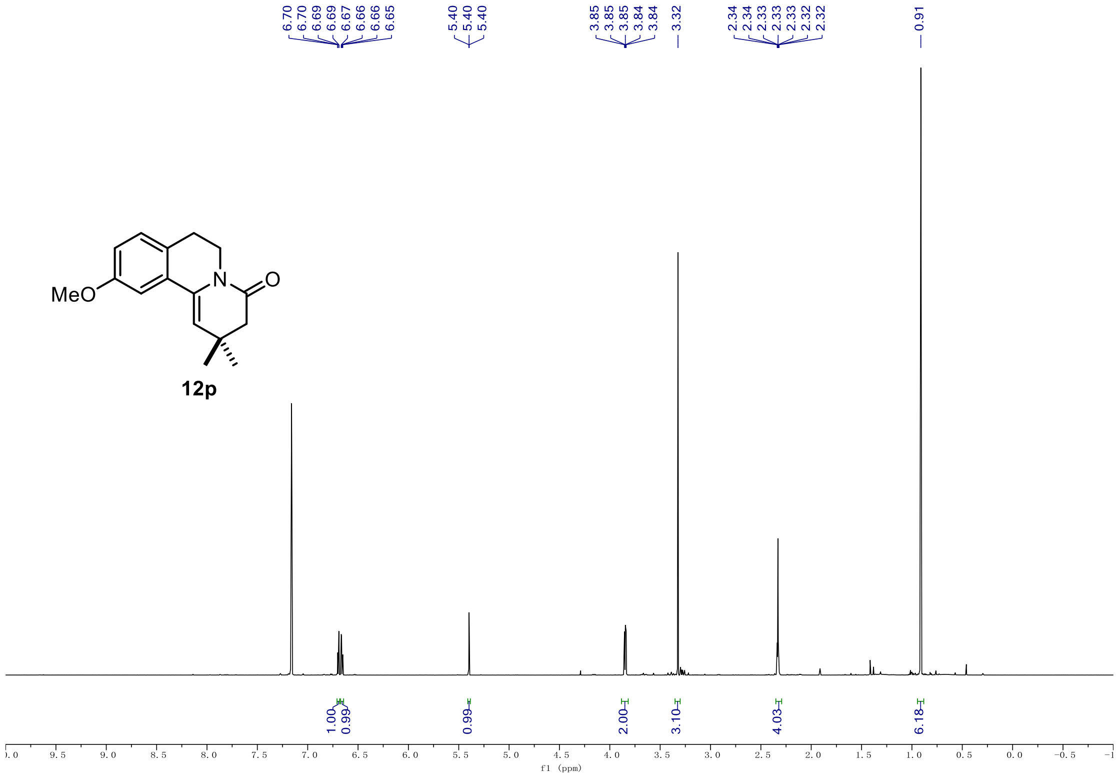


**$^{13}$C NMR** (176 MHz, $C_6D_6$) of **12p.**

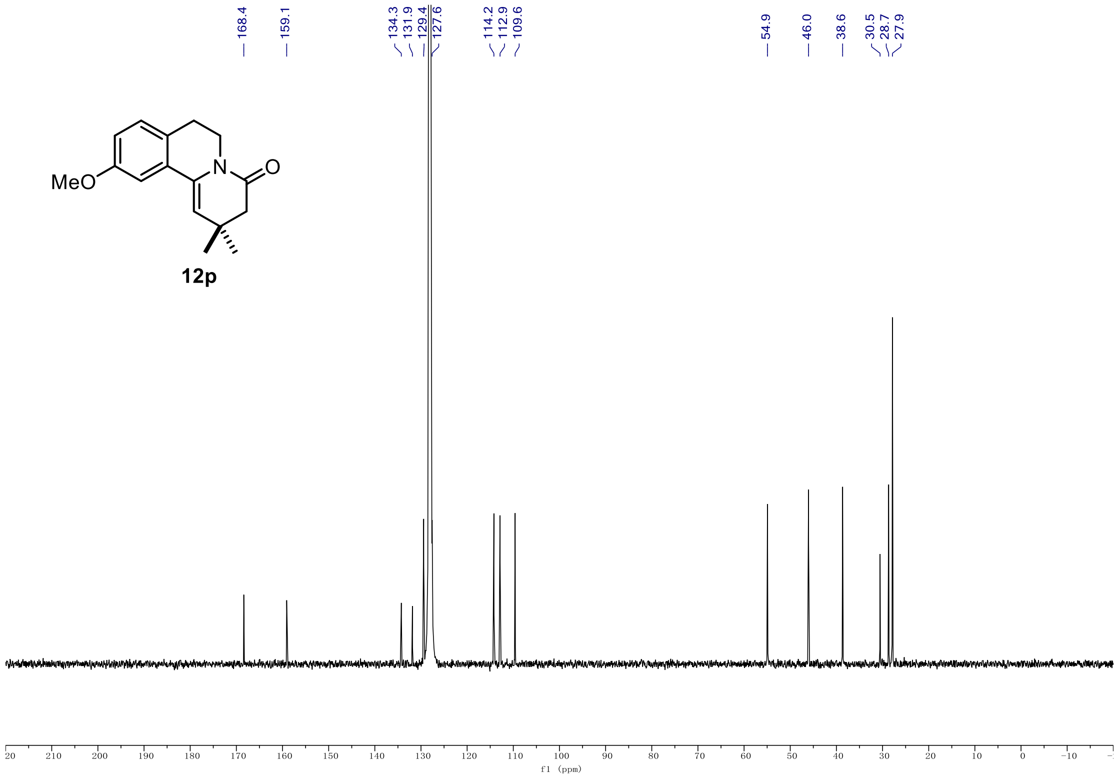

## $^{1}$H NMR (500 MHz, $CDCl_3$) of 12q.

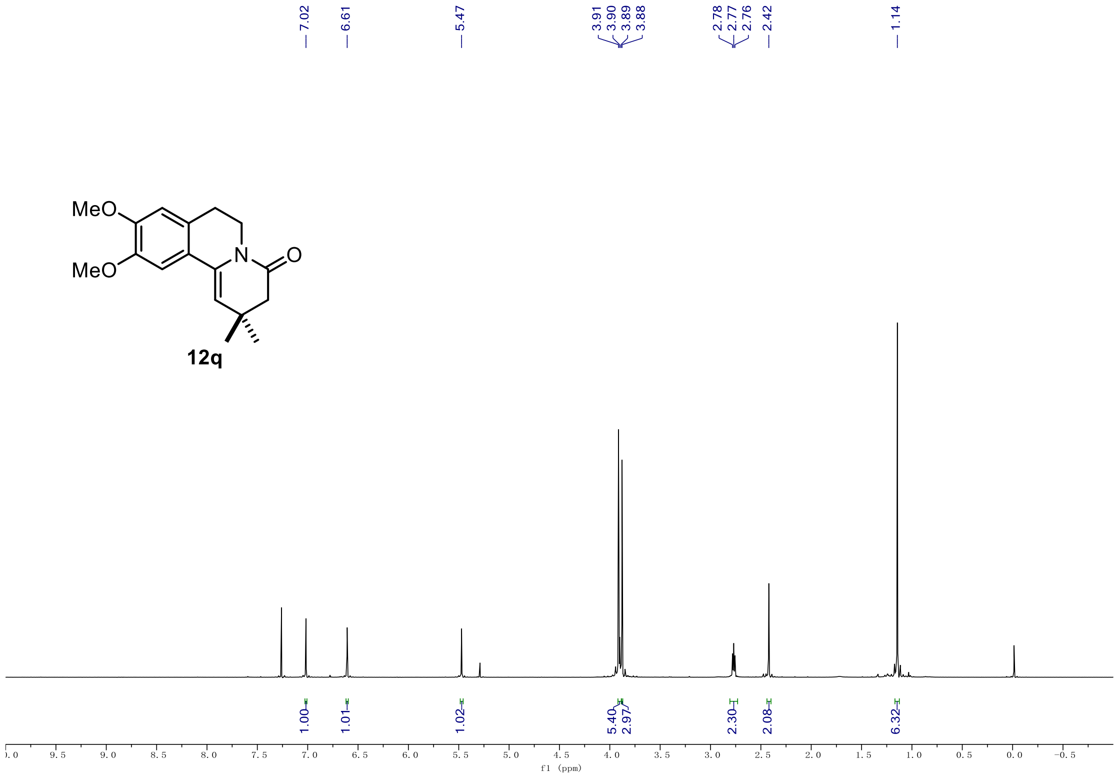


## $^{13}$C NMR (126 MHz, $CDCl_3$) of 12q.

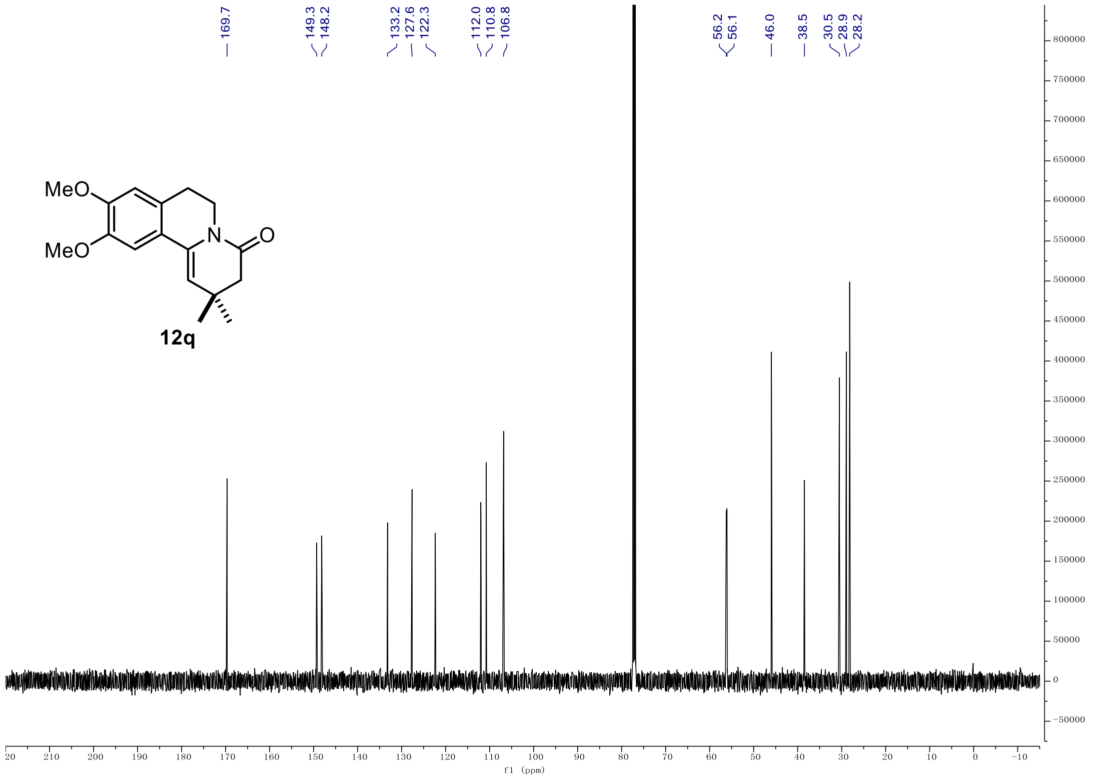

**$^{1}$H NMR** (500 MHz, $C_6D_6$) of **12r.**

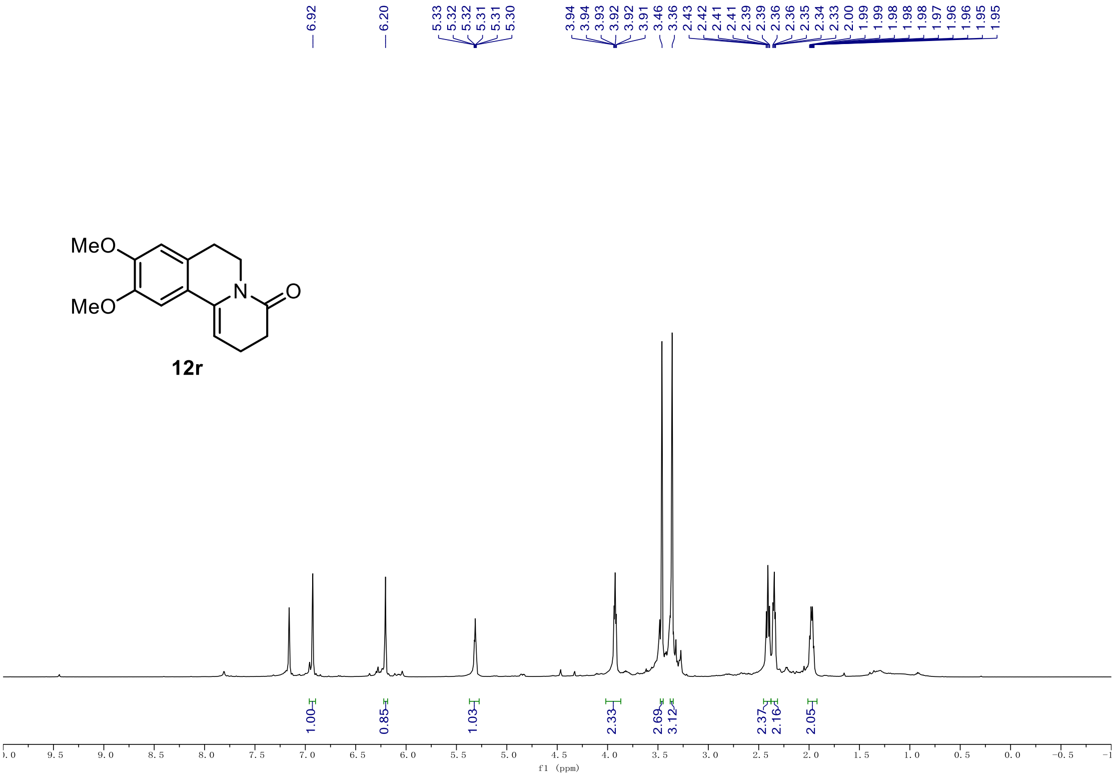


**$^{13}$C NMR** (126 MHz, $C_6D_6$) of **12r.**

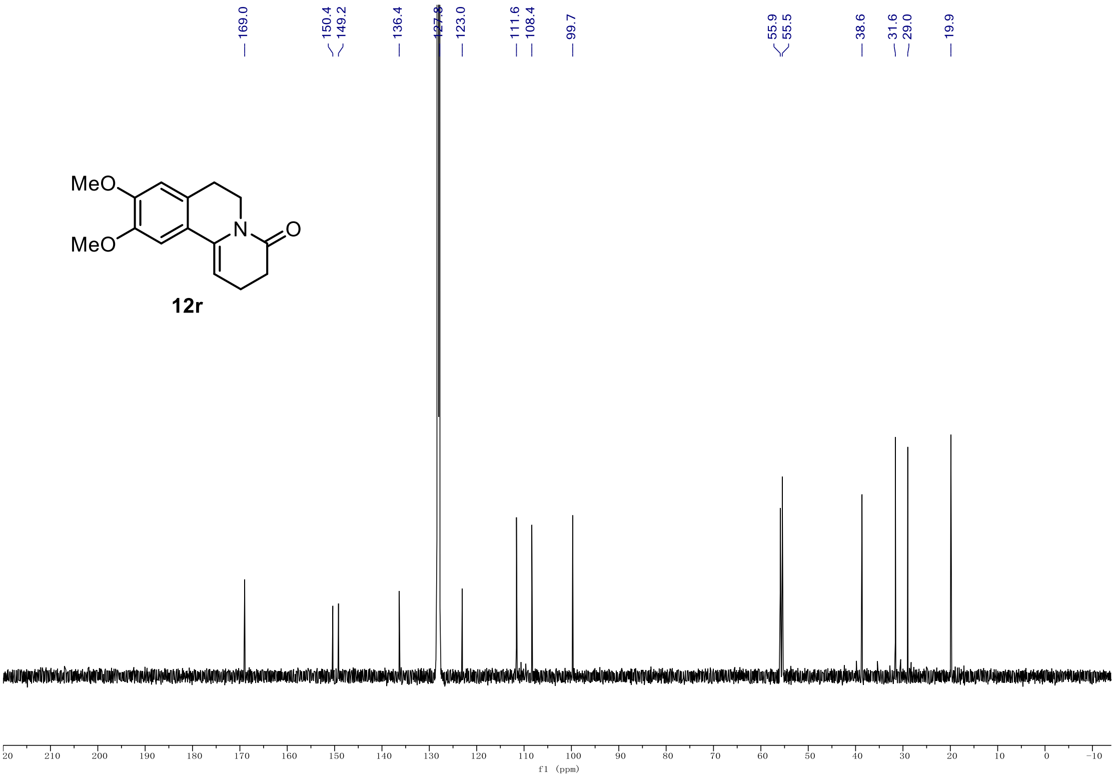

**$^{1}$H NMR** (600 MHz, $CDCl_3$) of **12s.**

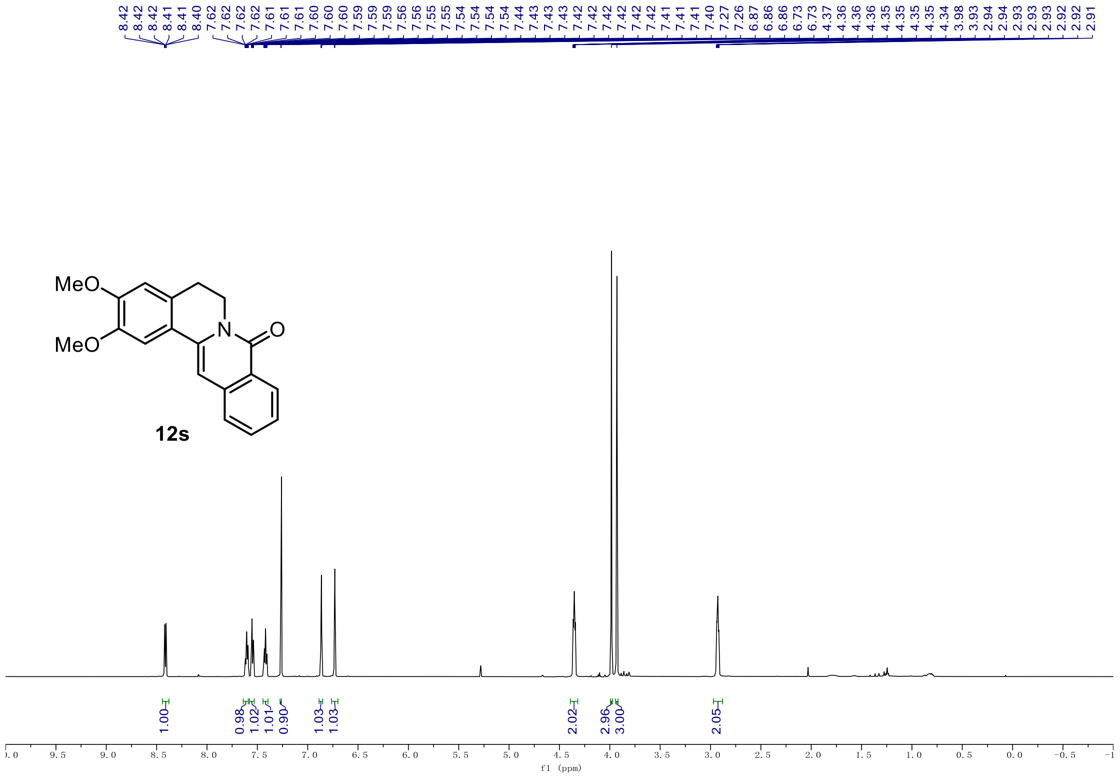


**$^{13}$C NMR** (151 MHz, $CDCl_3$) of **12s.**

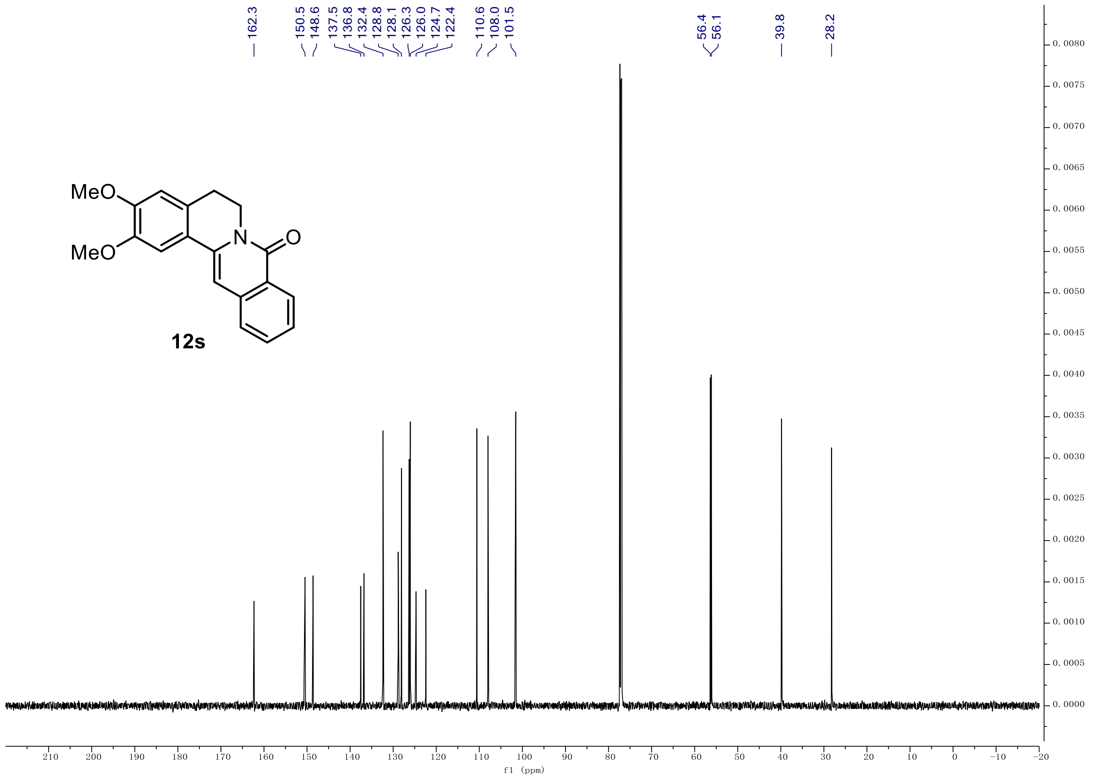

**$^{1}$H NMR** (600 MHz, $CD_2Cl_2$) of **12t.**

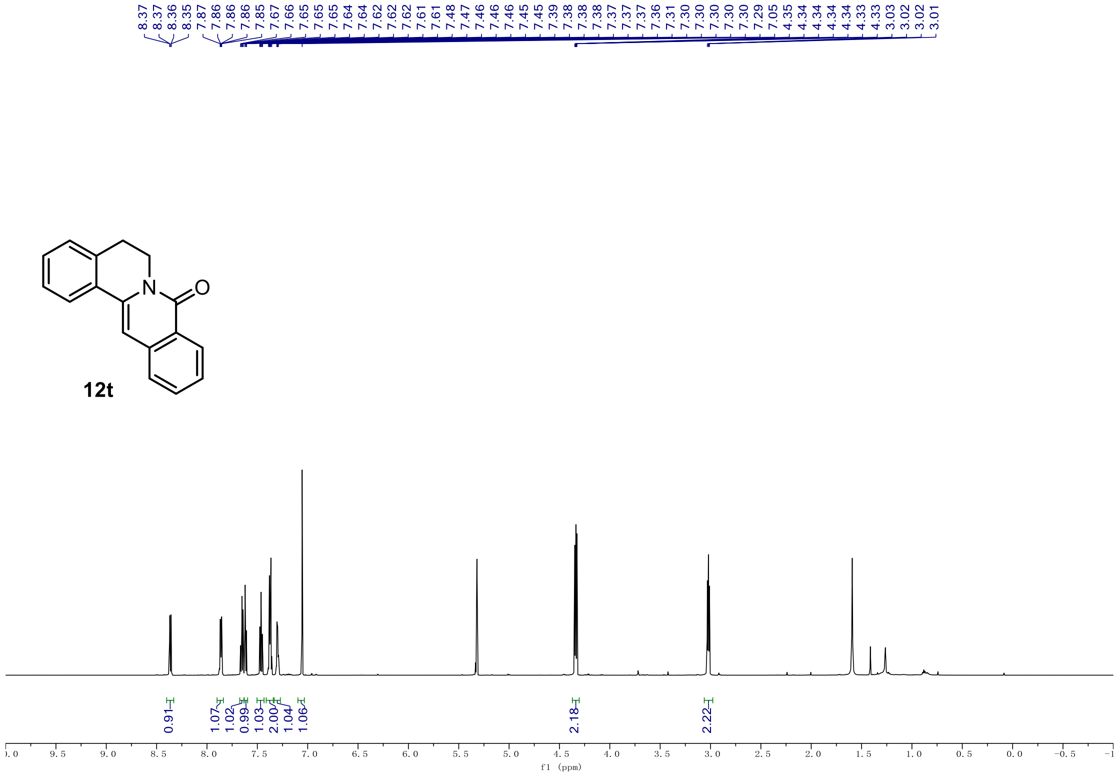


**$^{13}$C NMR** (151 MHz, $CD_2Cl_2$) of **12t.**

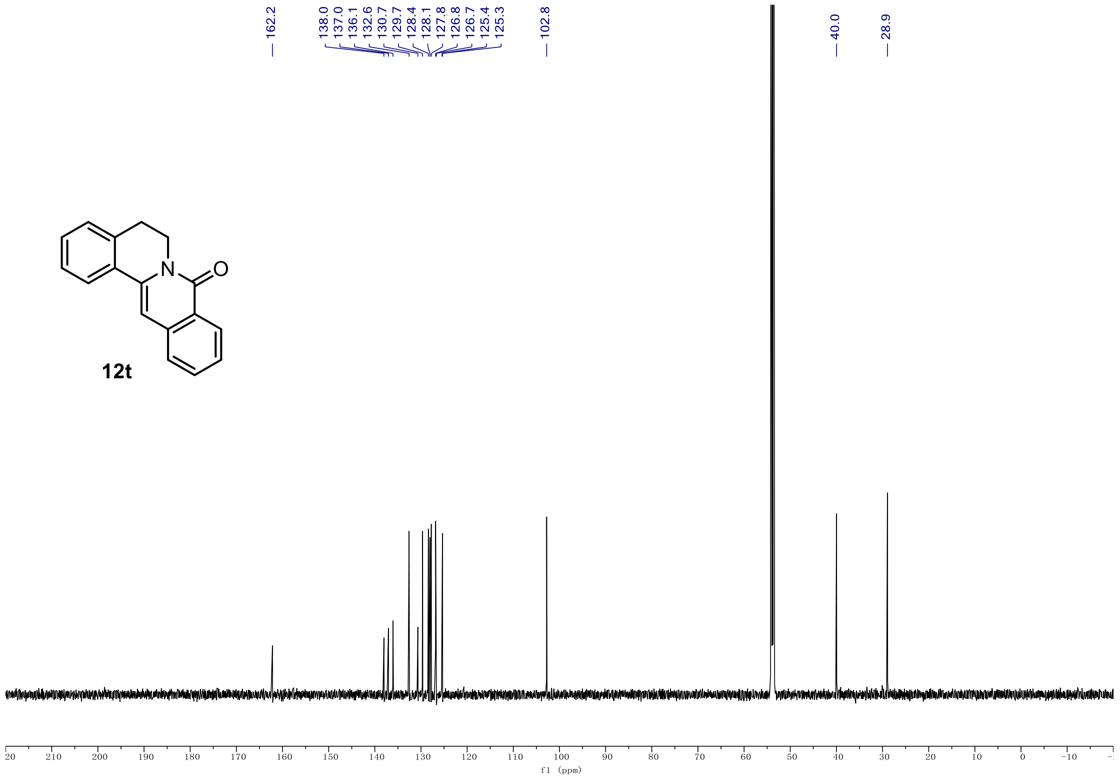

**$^{1}$H NMR** (700 MHz, $C_6D_6$) of **12u.**

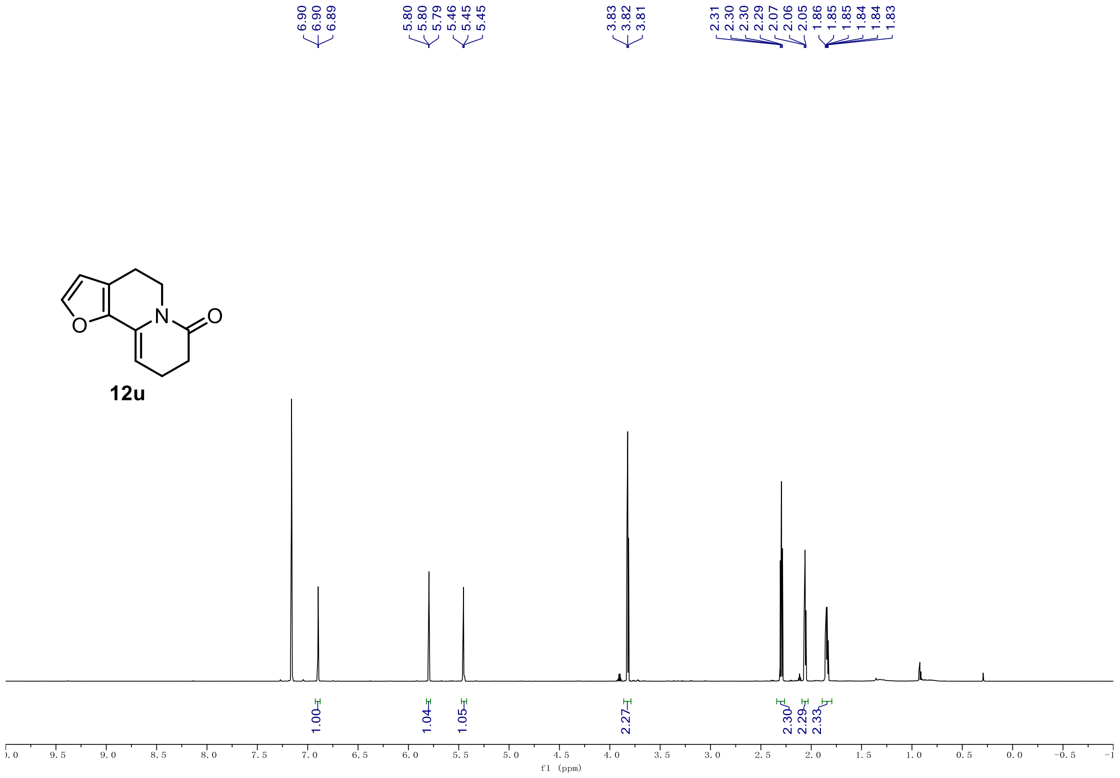


**$^{13}$C NMR** (176 MHz, $C_6D_6$) of **12u.**

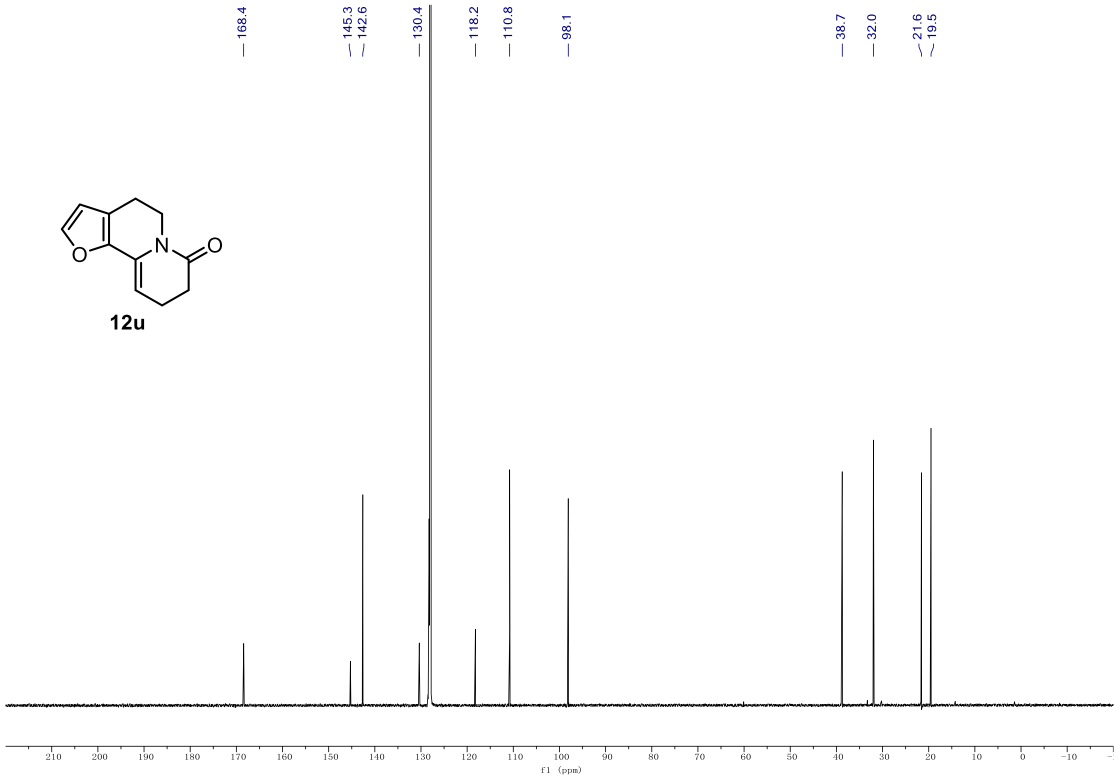

**$^{1}$H NMR** (700 MHz, $C_6D_6$) of **12v.**

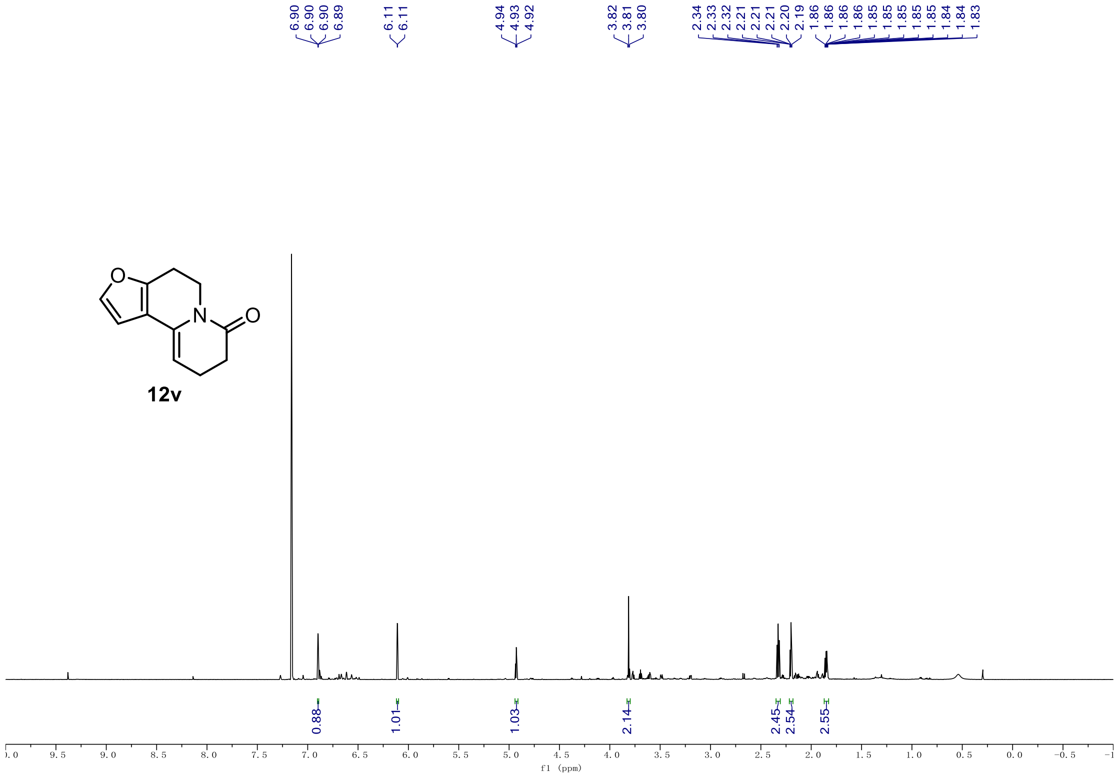


**$^{13}$C NMR** (176 MHz, $C_6D_6$) of **12v.**

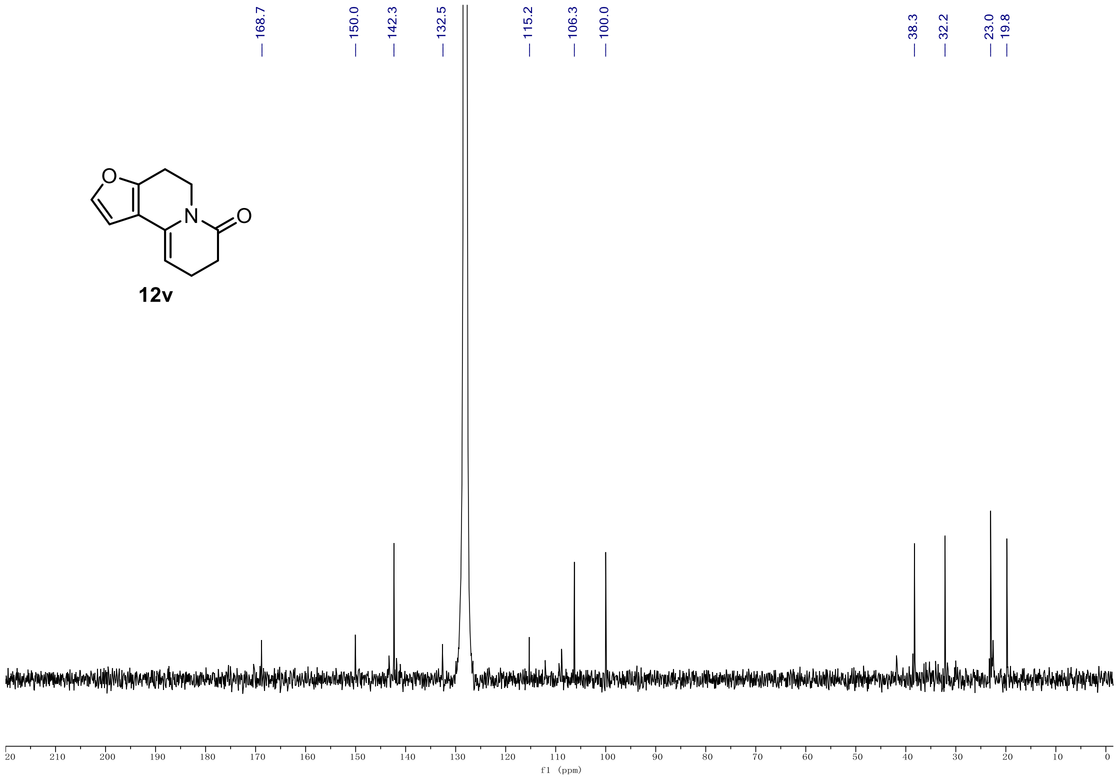

**$^{1}$H NMR** (500 MHz, $C_6D_6$) of **12w.**

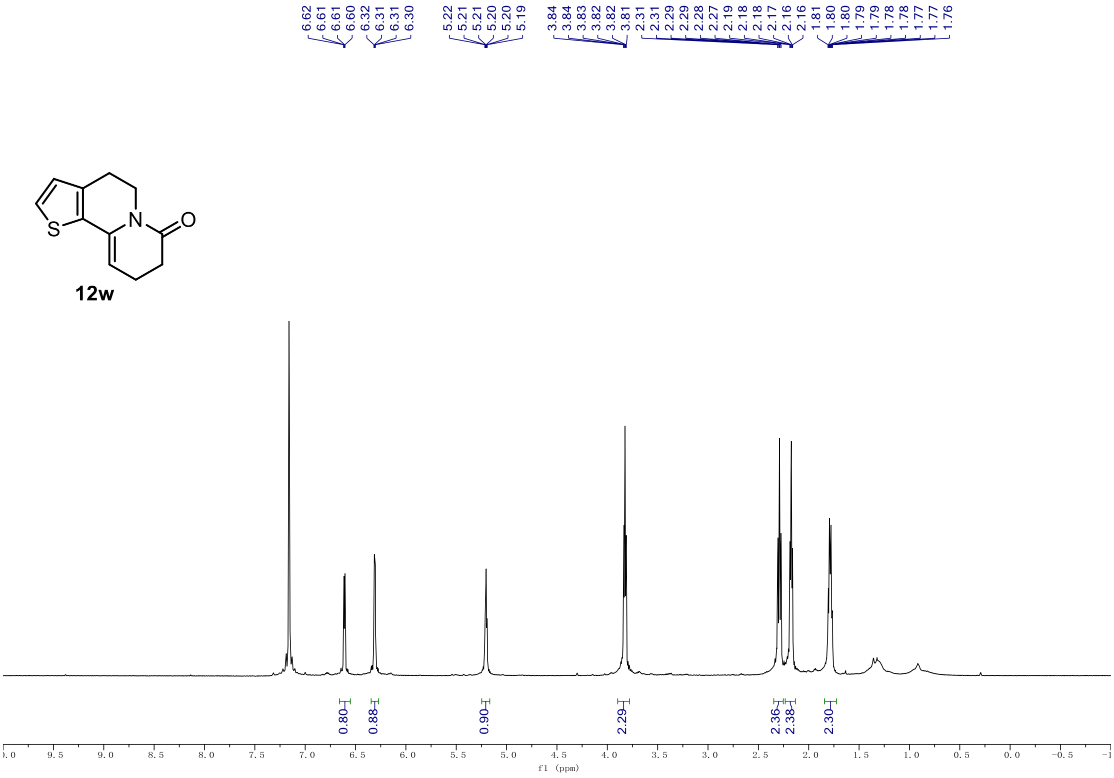


**$^{13}$C NMR** (126 MHz, $C_6D_6$) of **12w.**

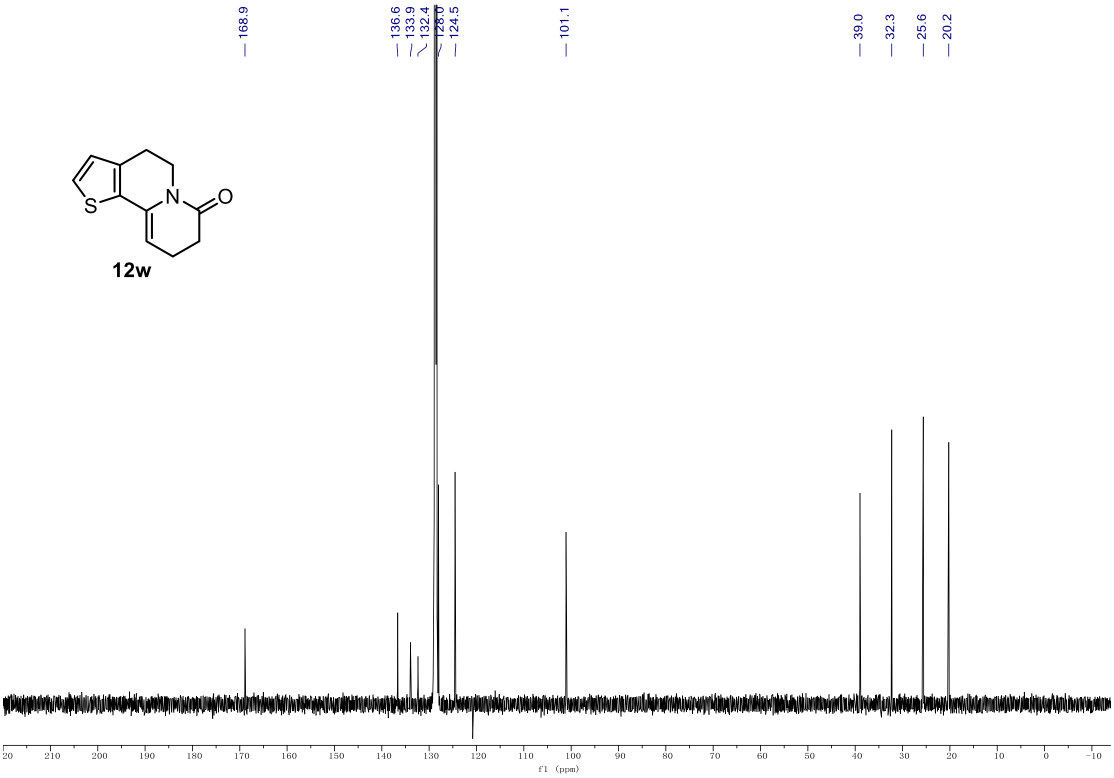

**$^1$H NMR** (700 MHz, $CD_2Cl_2$) of **12x.**

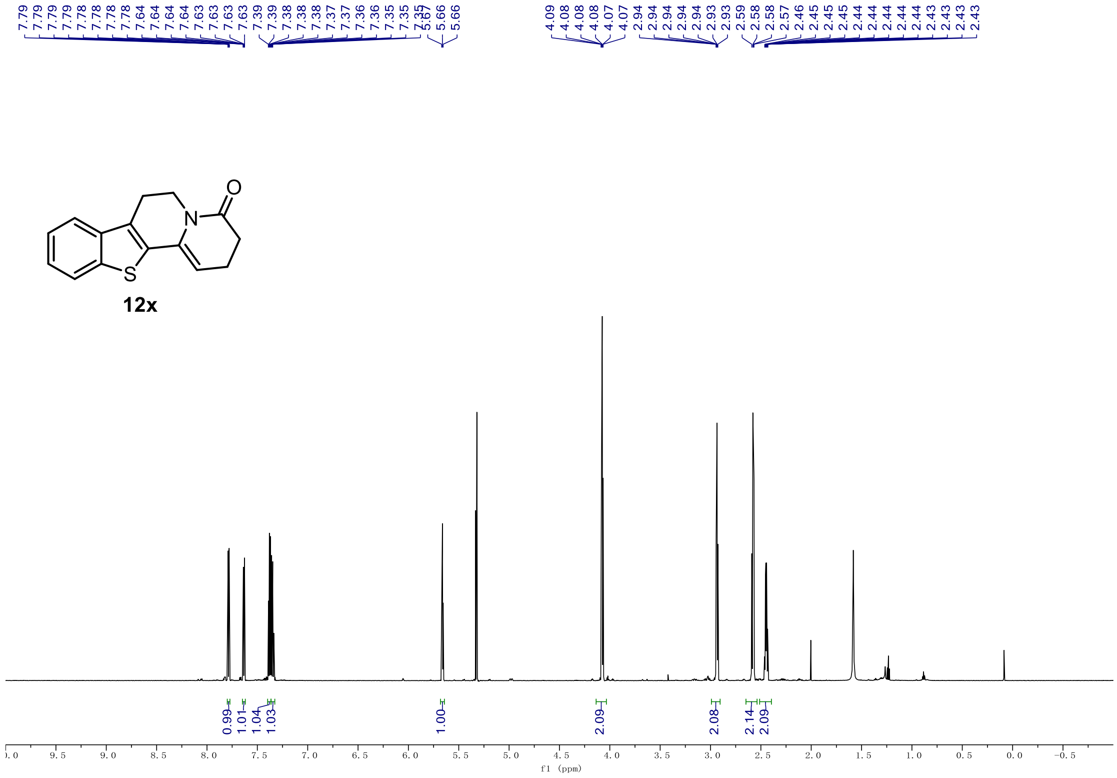

**$^{13}$C NMR** (176 MHz, $CD_2Cl_2$) of **12x.**

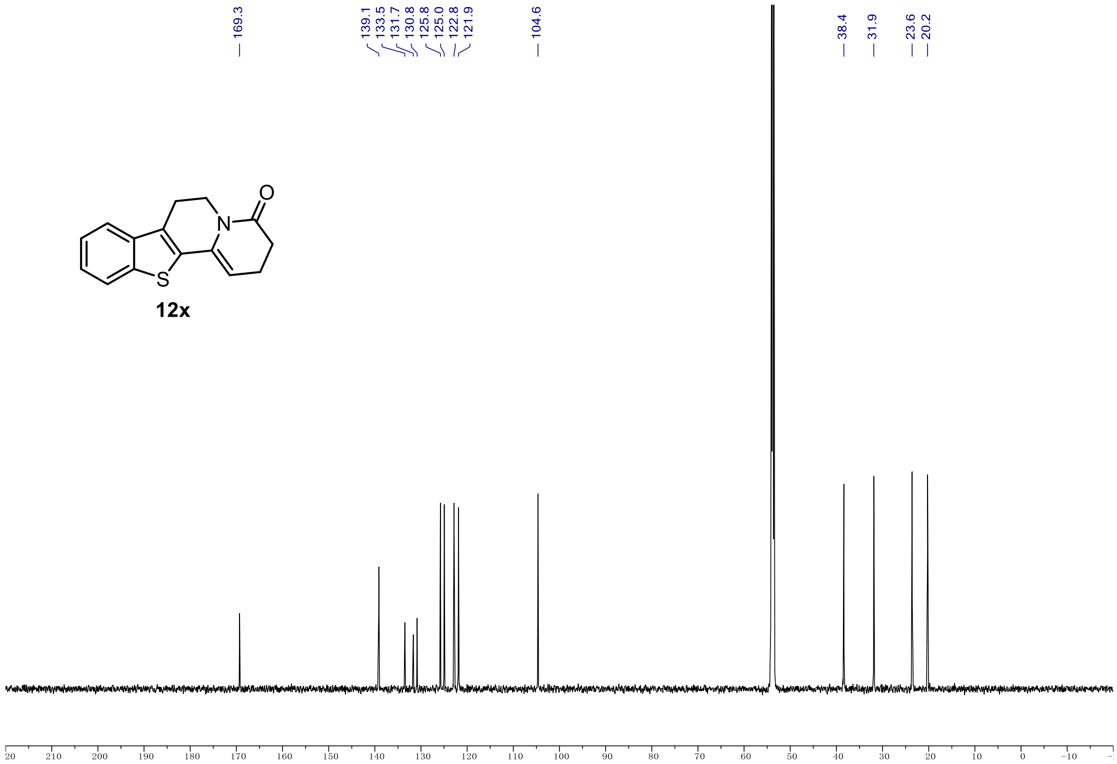

## $^{1}$H NMR (700 MHz, $CD_2Cl_2$) of 13.

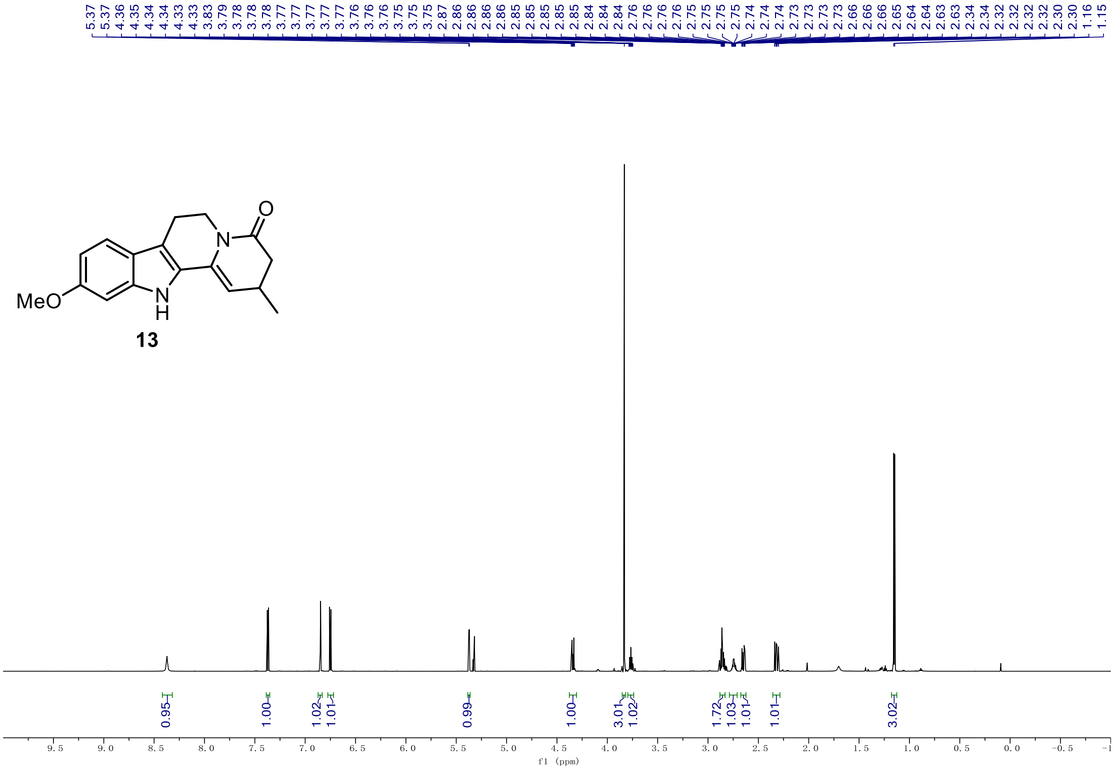


## $^{13}$C NMR (176 MHz, $CD_2Cl_2$) of 13.

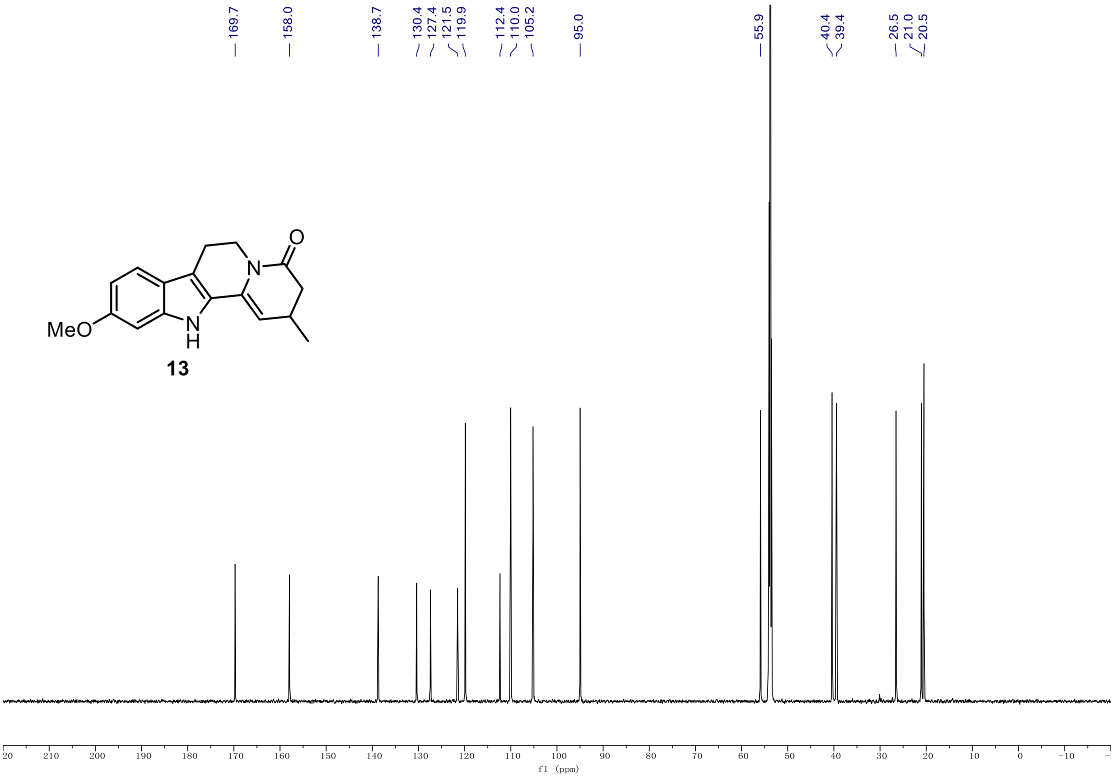

**$^{1}$H NMR** (700 MHz, $CD_3OD$) of **14a.**

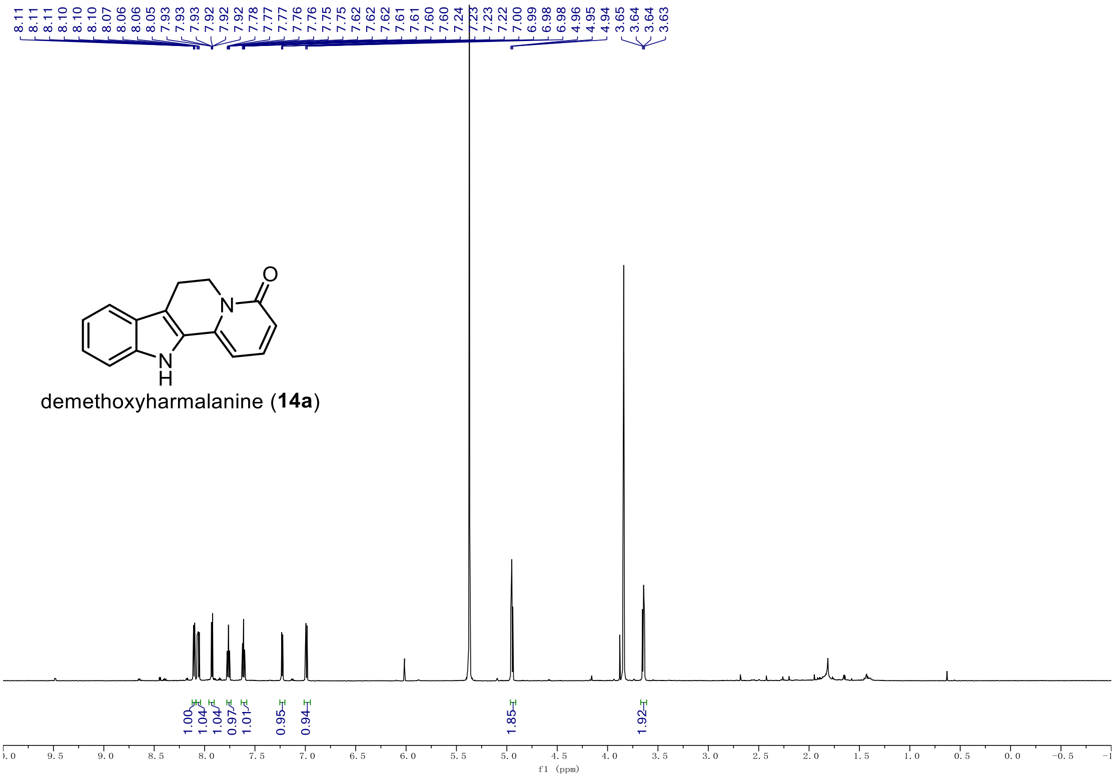


**$^{13}$C NMR** (176 MHz, $CD_3OD$) of **14a.**

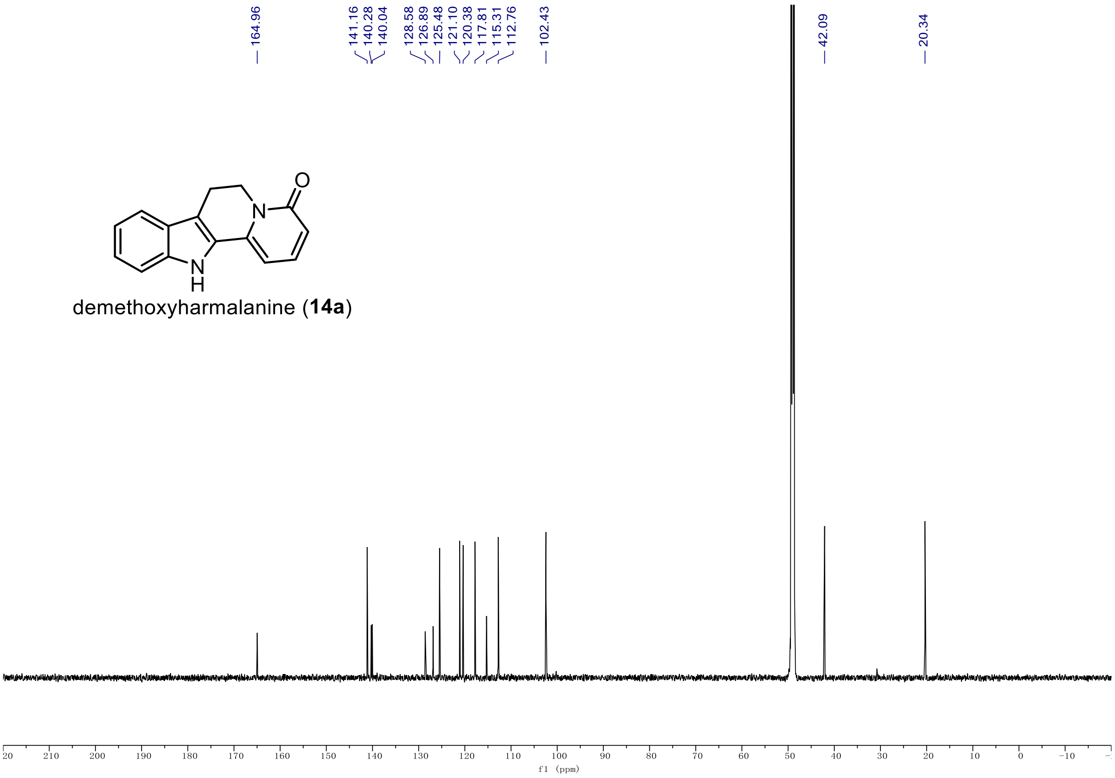

**$^{1}$H NMR** (500 MHz, $CDCl_3$) of **14b.**

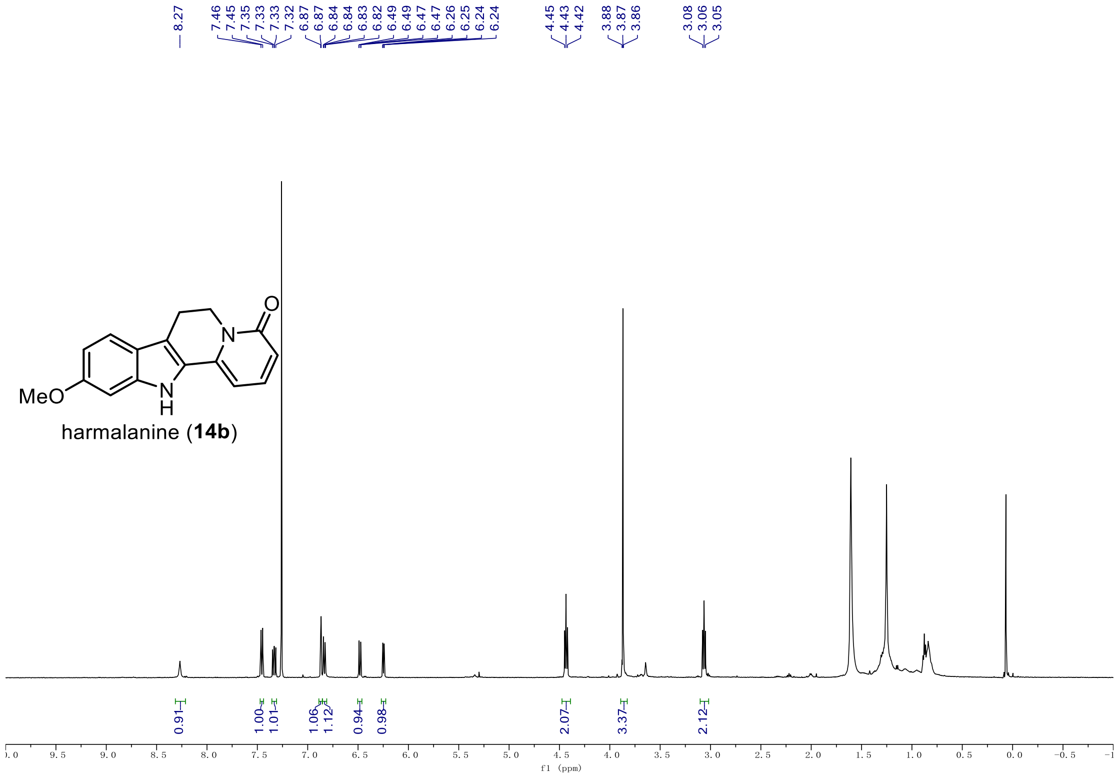


**$^{13}$C NMR** (126 MHz, $CD_3OD$) of **14b.**

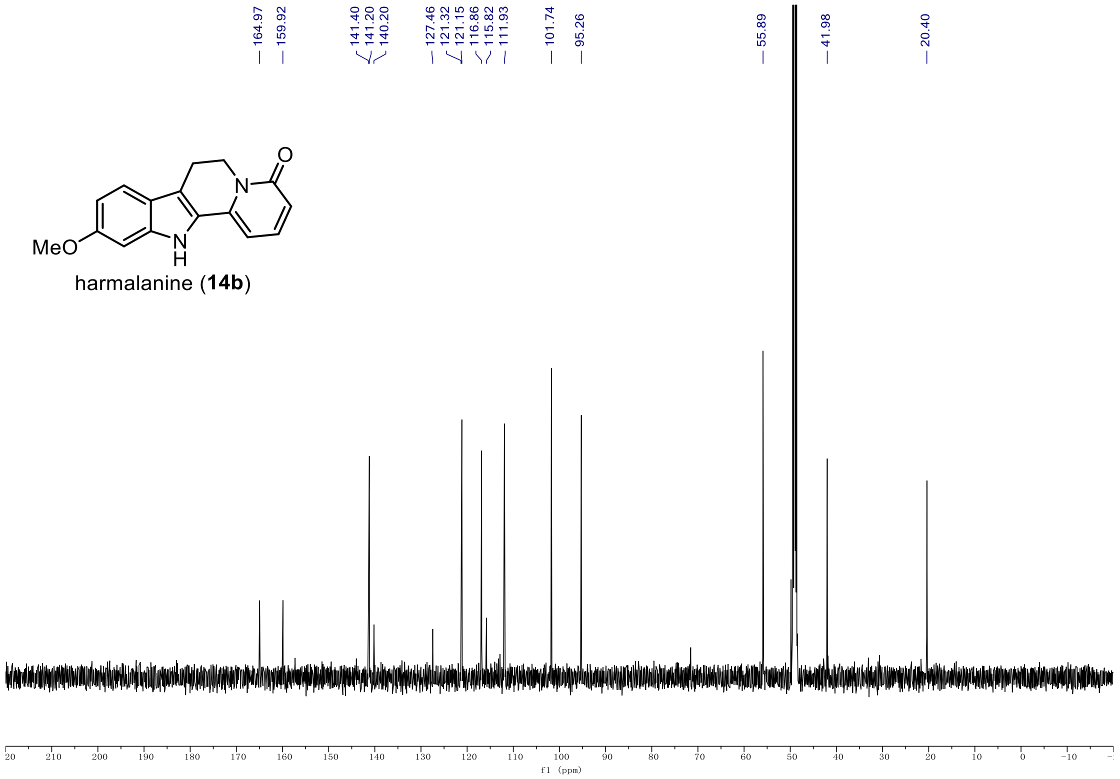

**$^{1}$H NMR** (500 MHz, $CDCl_3$+$CD_3OD$ (v/v = 1:1)) of **14c.**

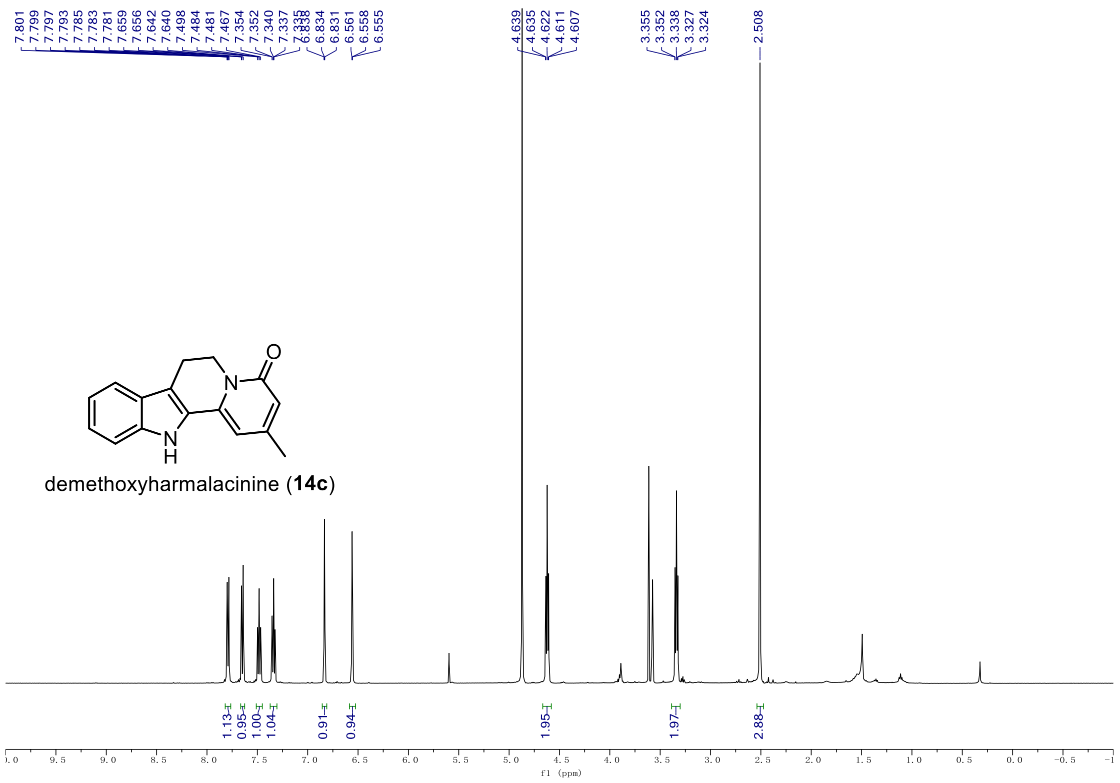


**$^{13}$C NMR** (126 MHz, $CDCl_3$+$CD_3OD$ (v/v = 1:1)) of **14c.**

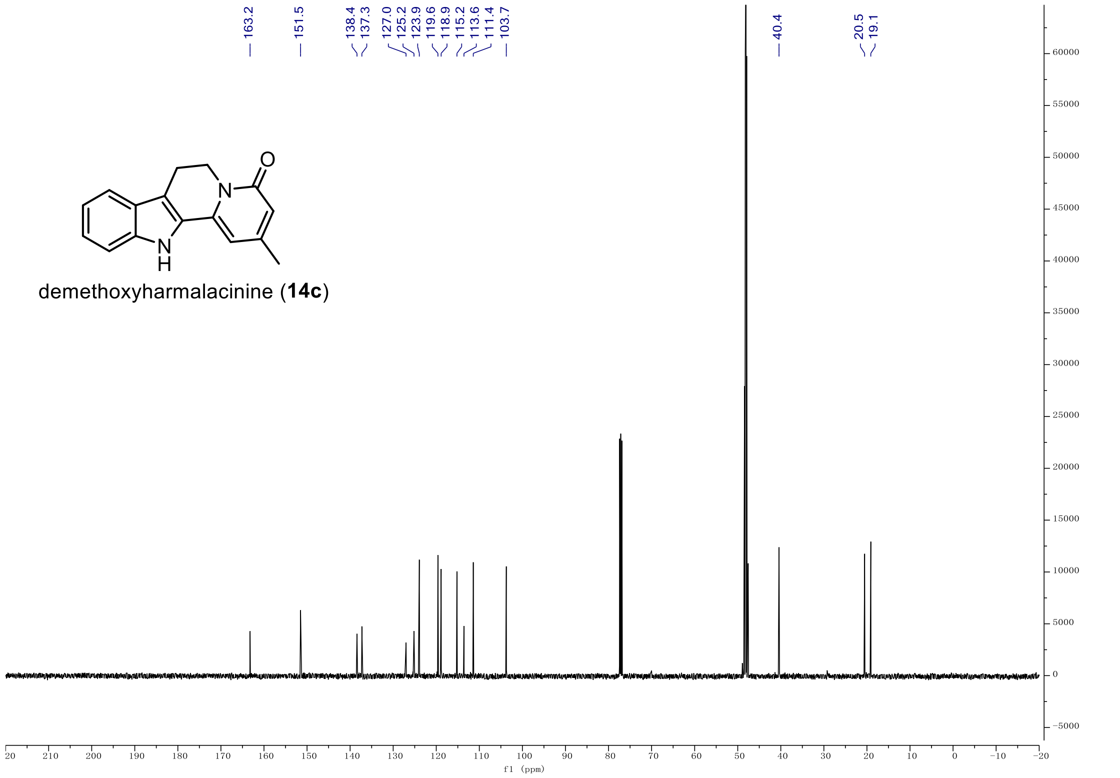

**$^{1}$H NMR** (500 MHz, CDCl$_3$) of **14d.**

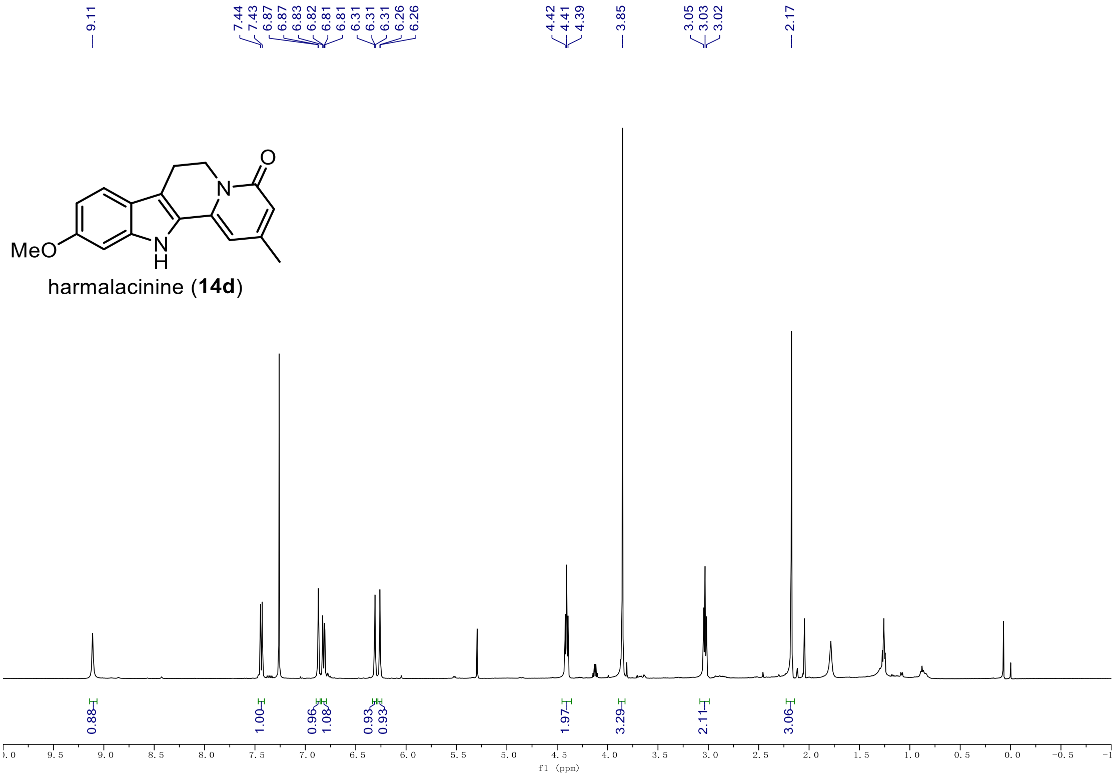


**$^{13}$C NMR** (126 MHz, CDCl$_3$) of **14d.**

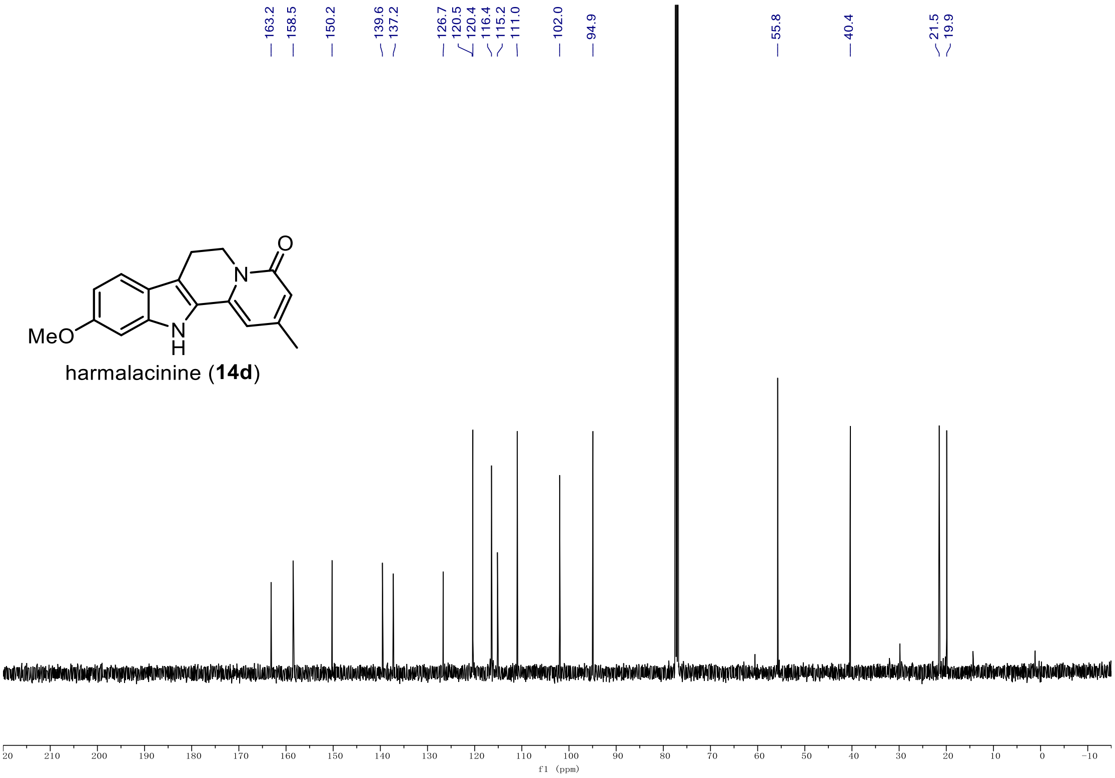

## $^{1}$H NMR (500 MHz, $CDCl_3$) of **15.**

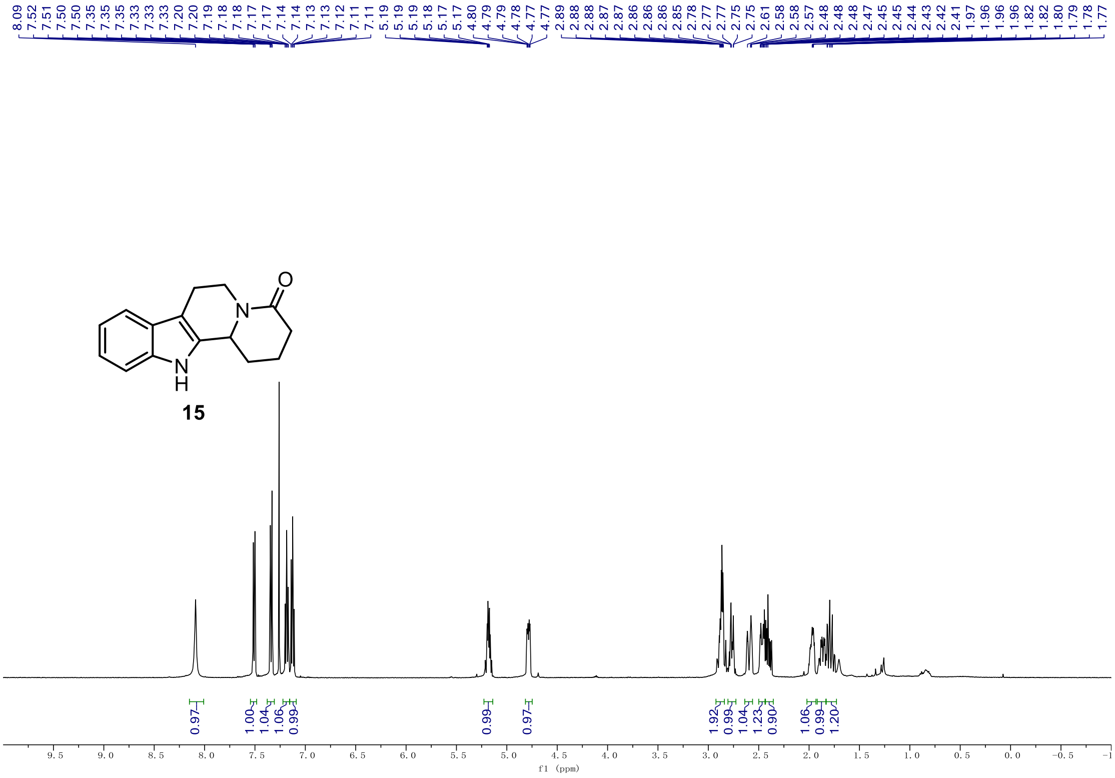

## $^{13}$C NMR (126 MHz, $CDCl_3$) of **15.**

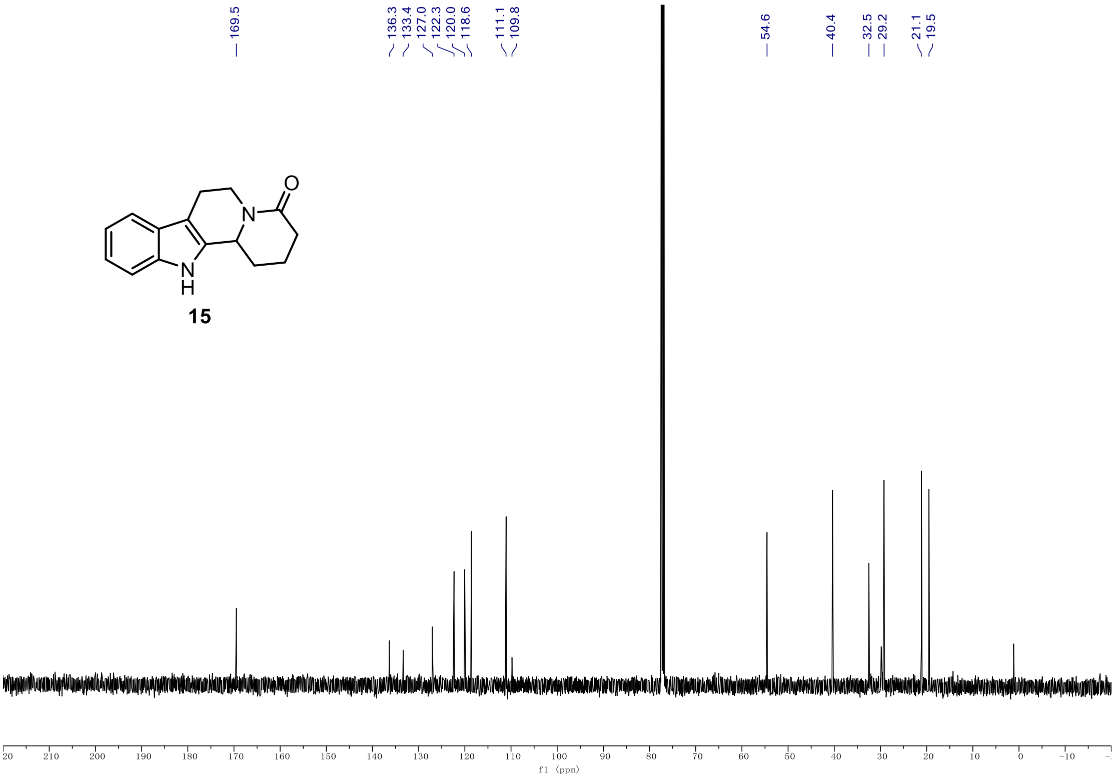

**$^{1}$H NMR** (500 MHz, $d_6$-DMSO) of **16.**

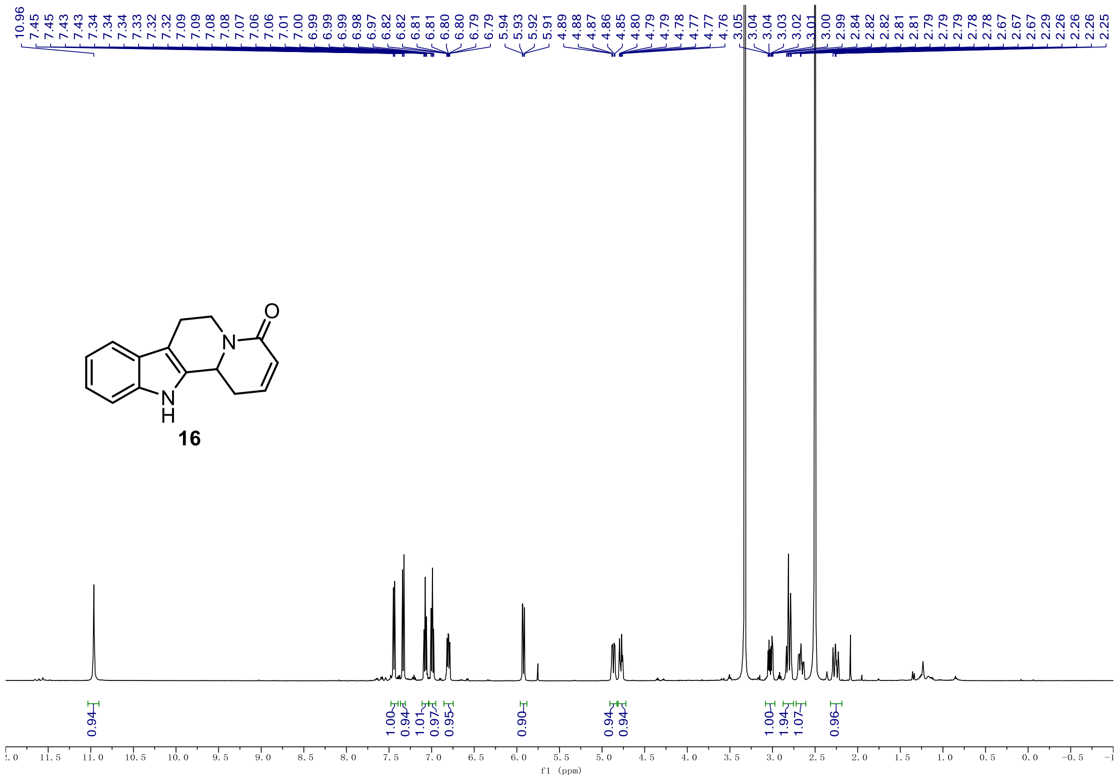


**$^{13}$C NMR** (126 MHz, $d_6$-DMSO) of **16.**

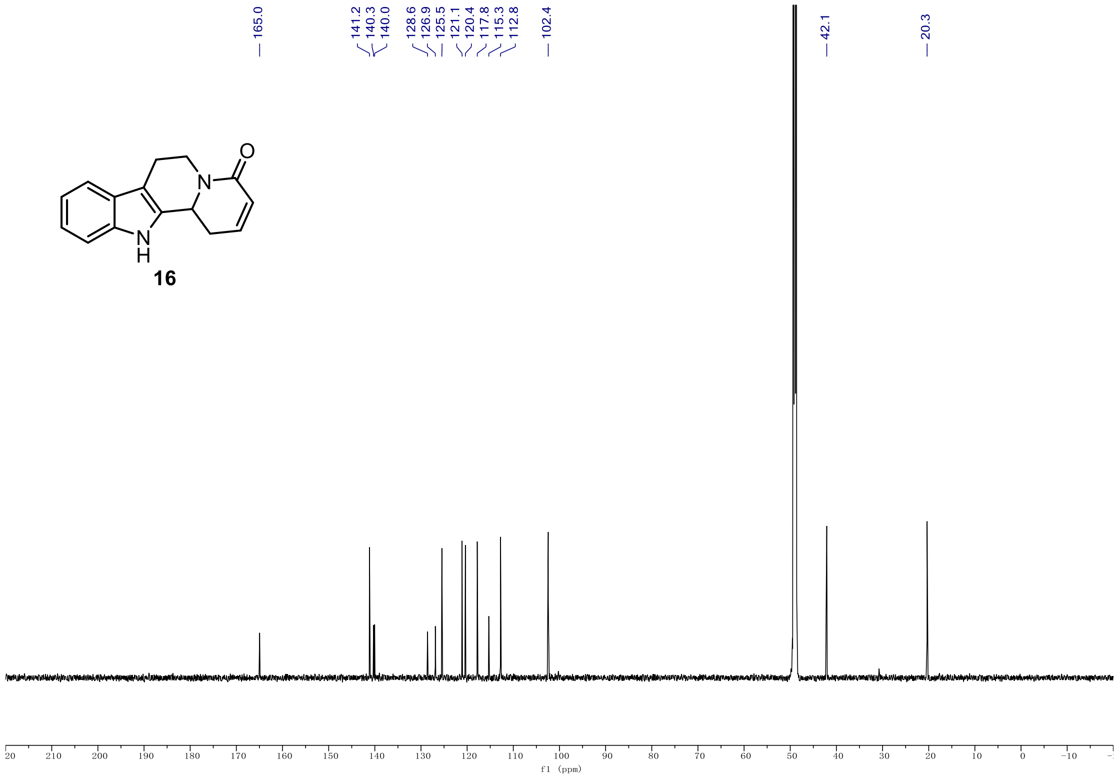

**$^{1}$H NMR** (500 MHz, $CD_2Cl_2$) of **24.**

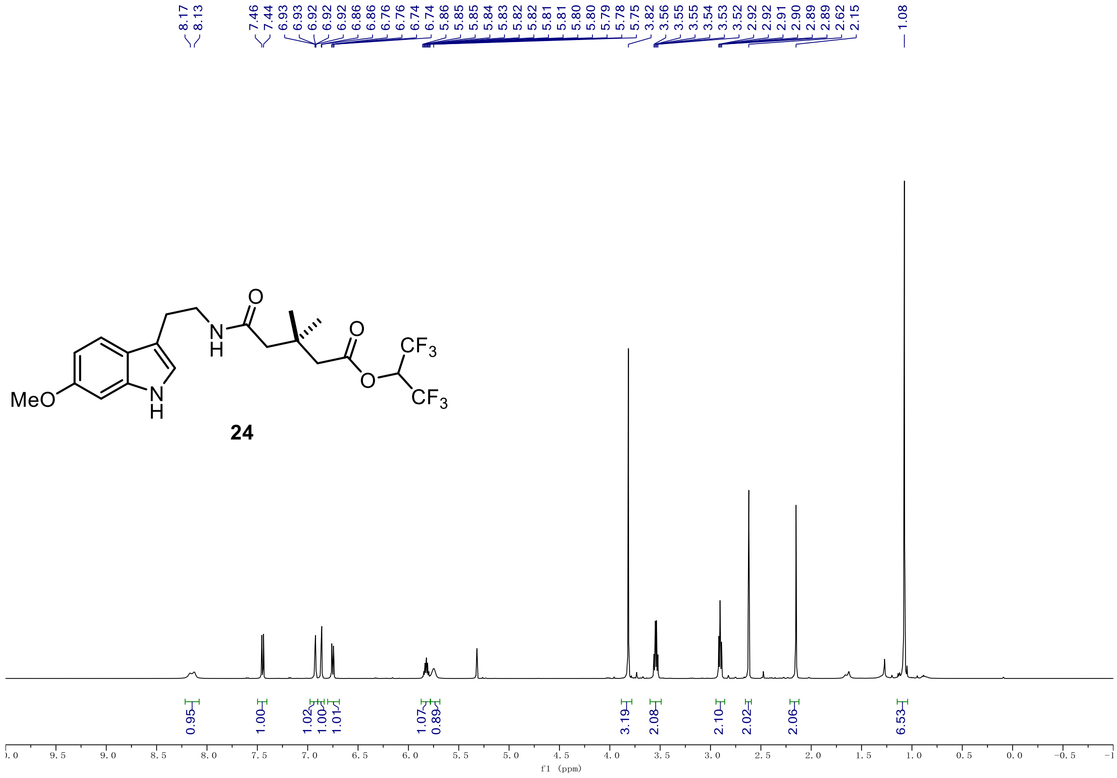

**$^{13}$C NMR** (126 MHz, $CD_2Cl_2$) of **24.**

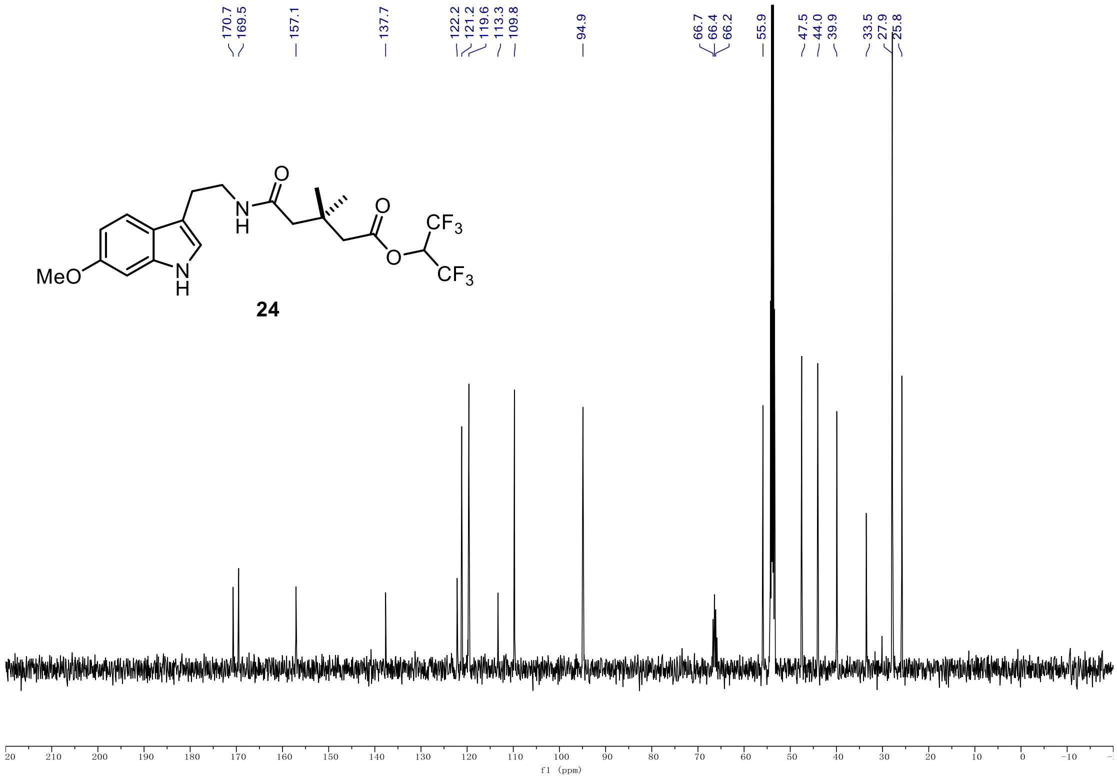

**$^{19}$F NMR** (471 MHz, $CD_2Cl_2$) of **24.**

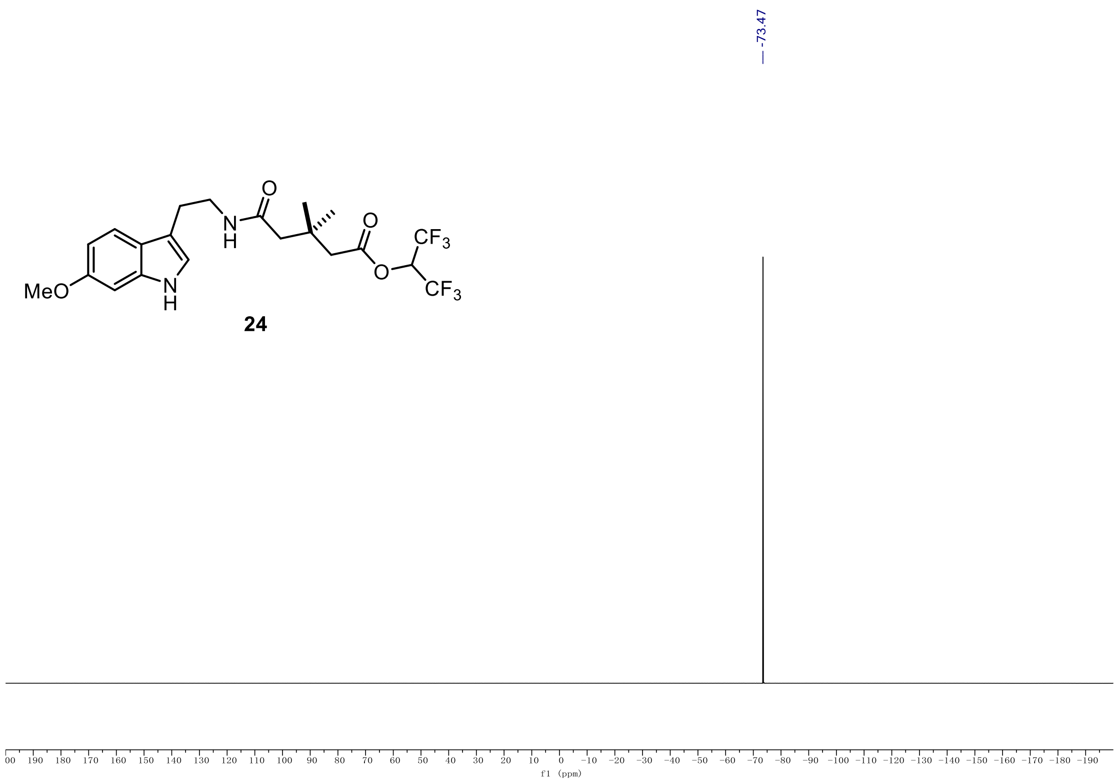


**$^{1}$H NMR** (700 MHz, $CDCl_3$) of **25.**

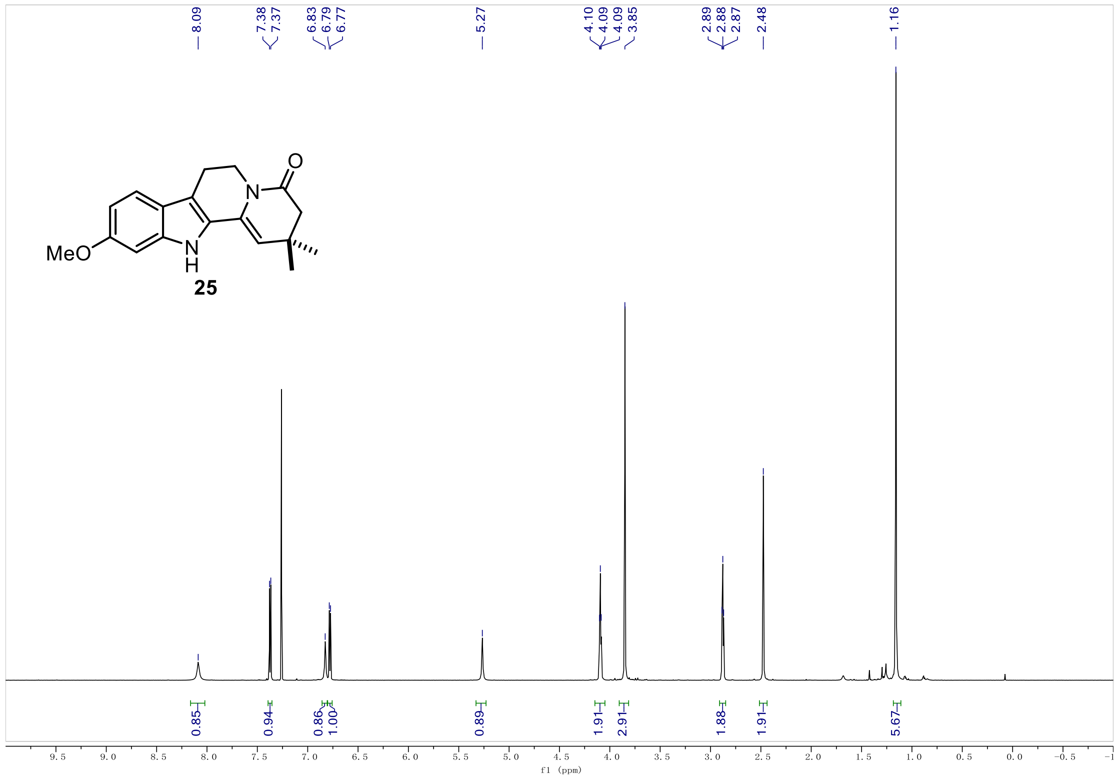

**$^{13}$C NMR** (176 MHz, $CDCl_3$) of **25.**

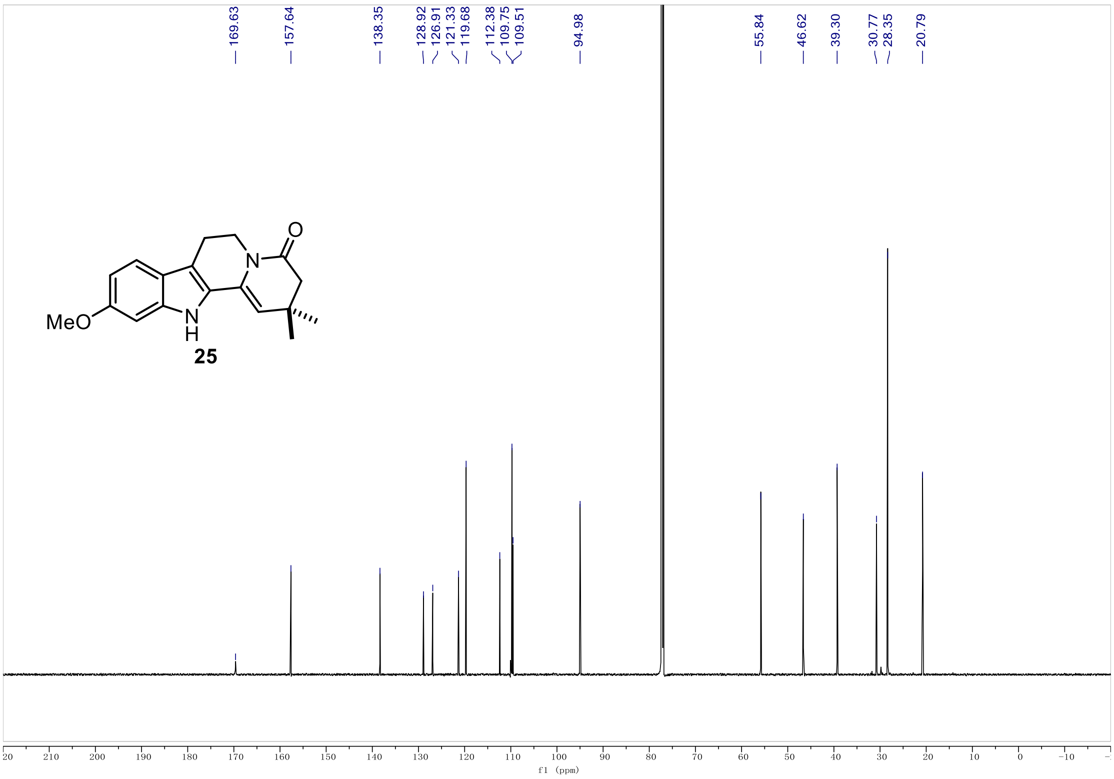


**$^{1}$H NMR** (500 MHz, $CDCl_3$) of **S15.**

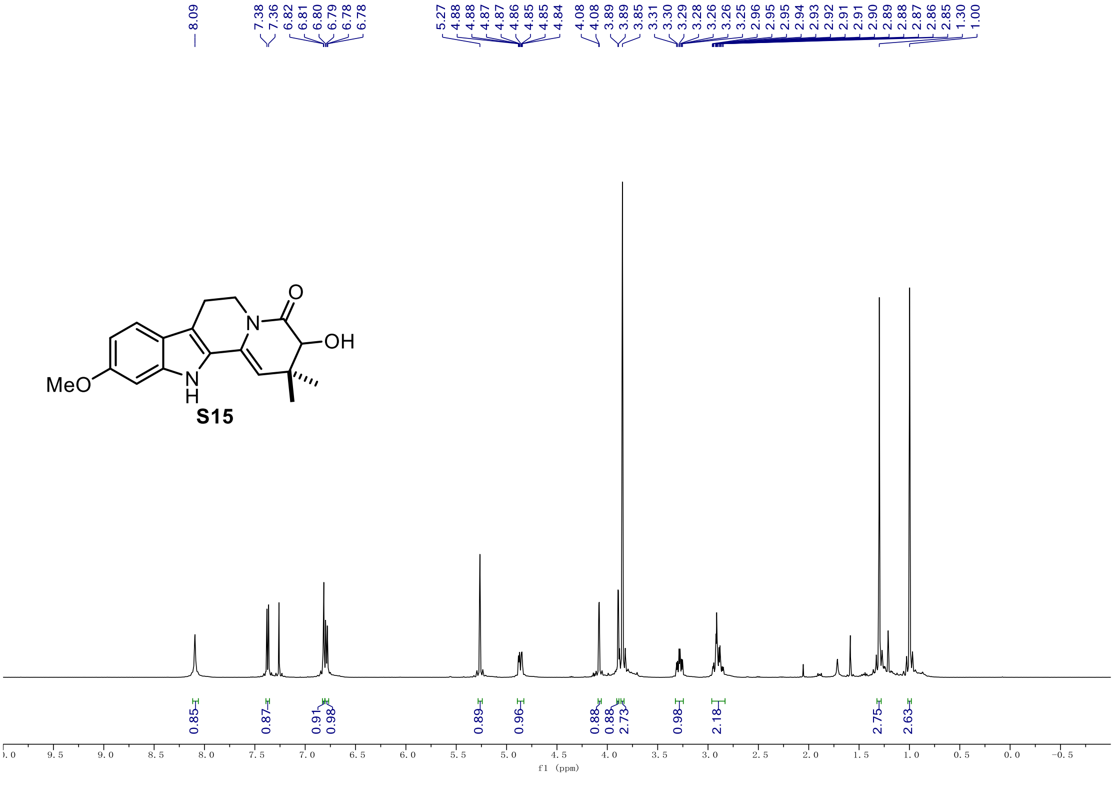

**$^{13}$C NMR** (126 MHz, $CDCl_3$) of **S15.**

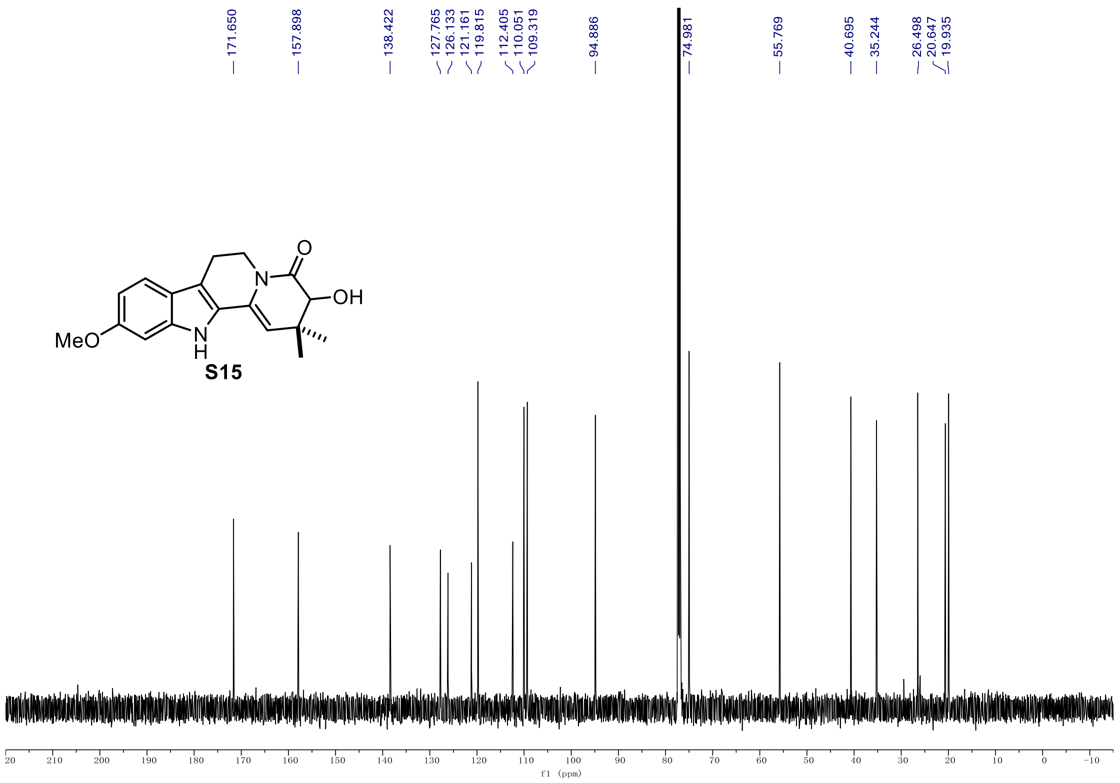


**$^{1}$H NMR** (700 MHz, $CDCl_3$) of **26.**

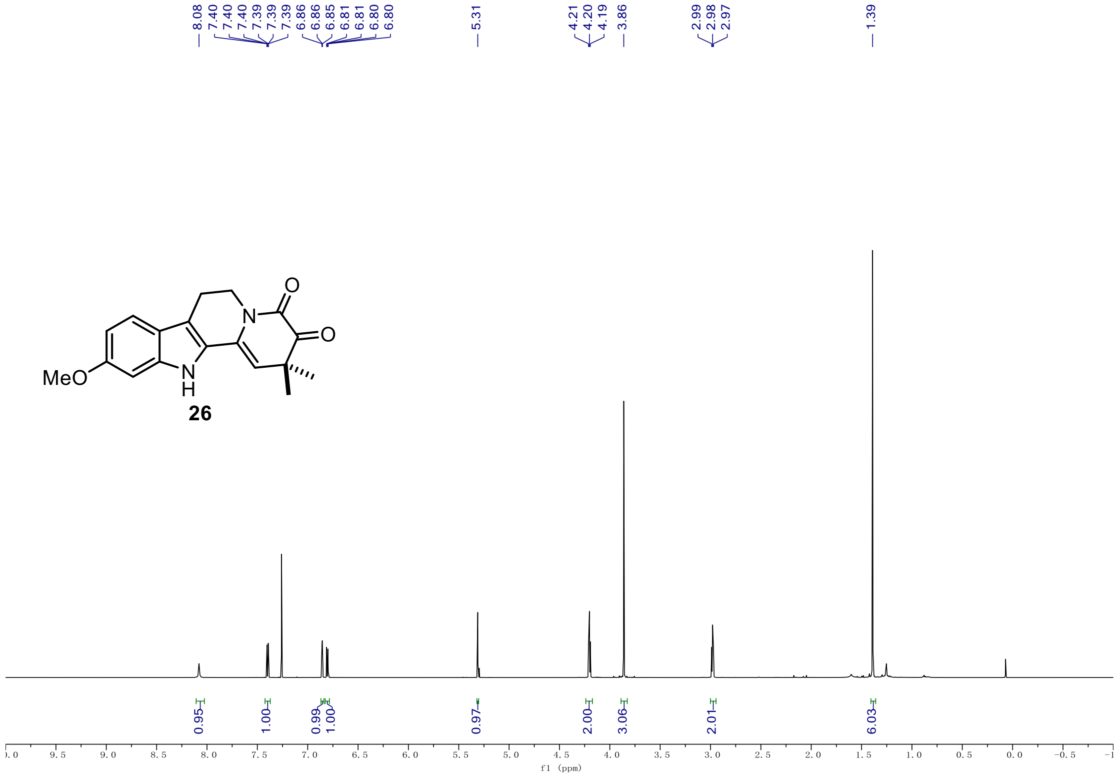

**$^{13}$C NMR** (176 MHz, $CDCl_3$) of **26.**

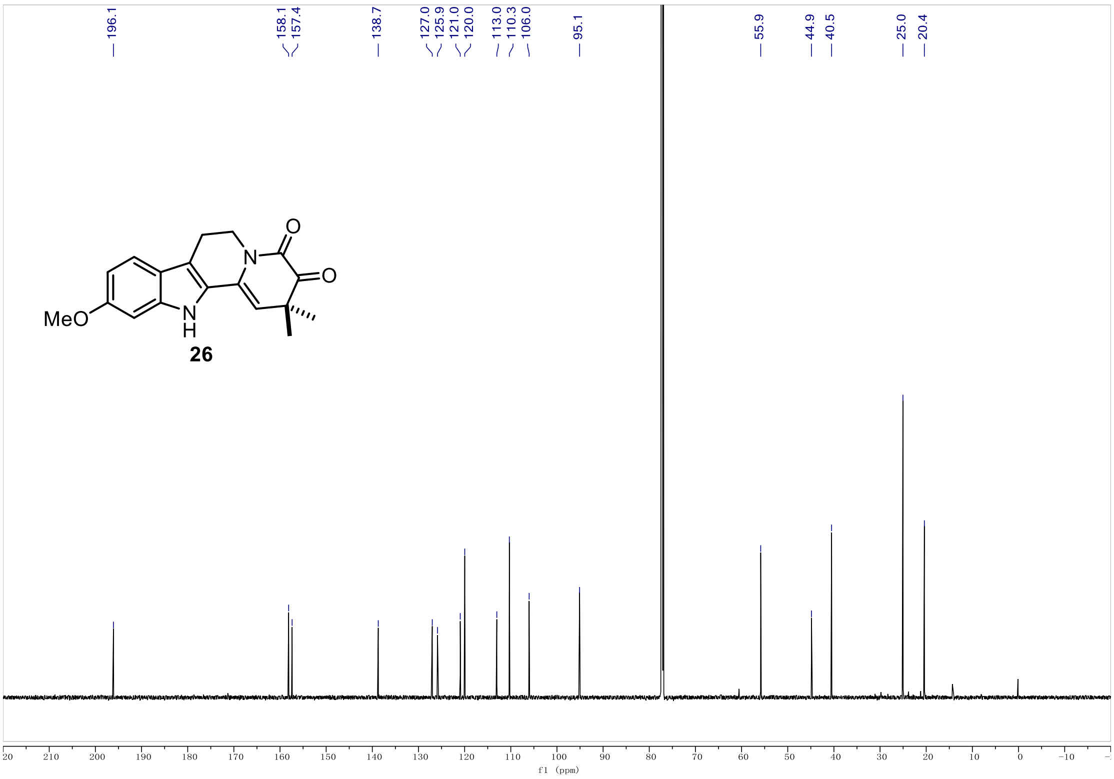


**$^{1}$H NMR** (700 MHz, **$CD_3OD$**) of peganumine A (**6**).

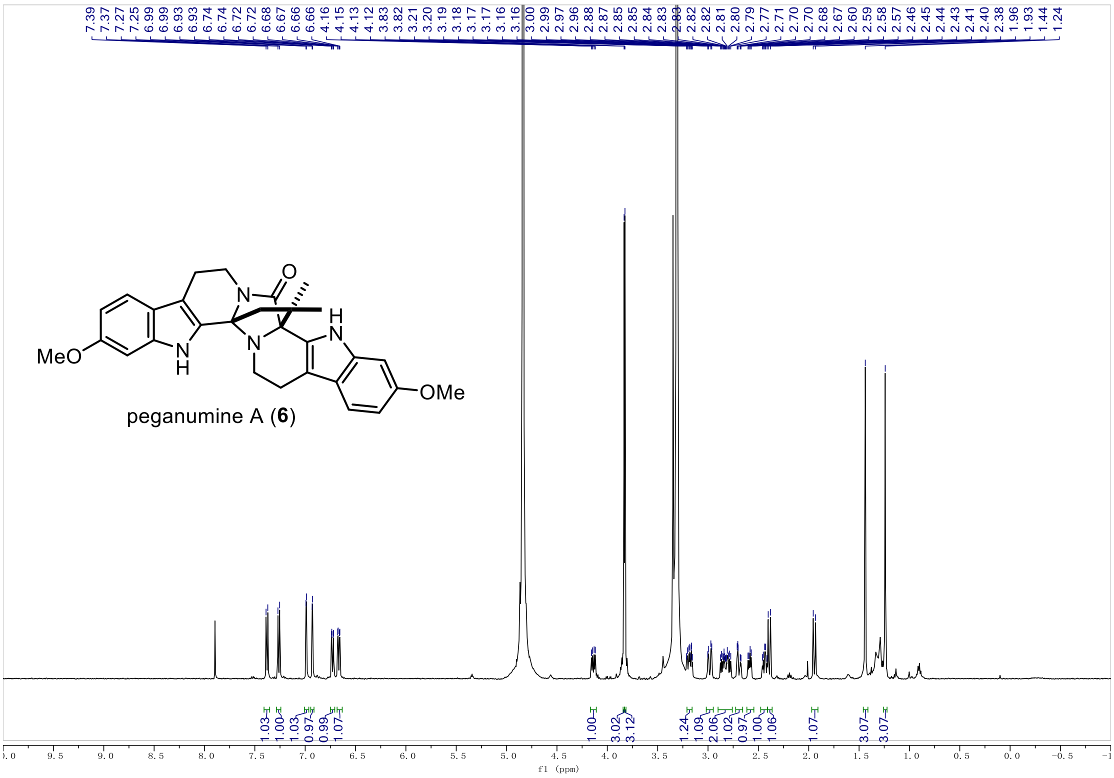

**$^{13}$C NMR** (176 MHz, **$CD_3OD$**) of peganumine A (**6**).

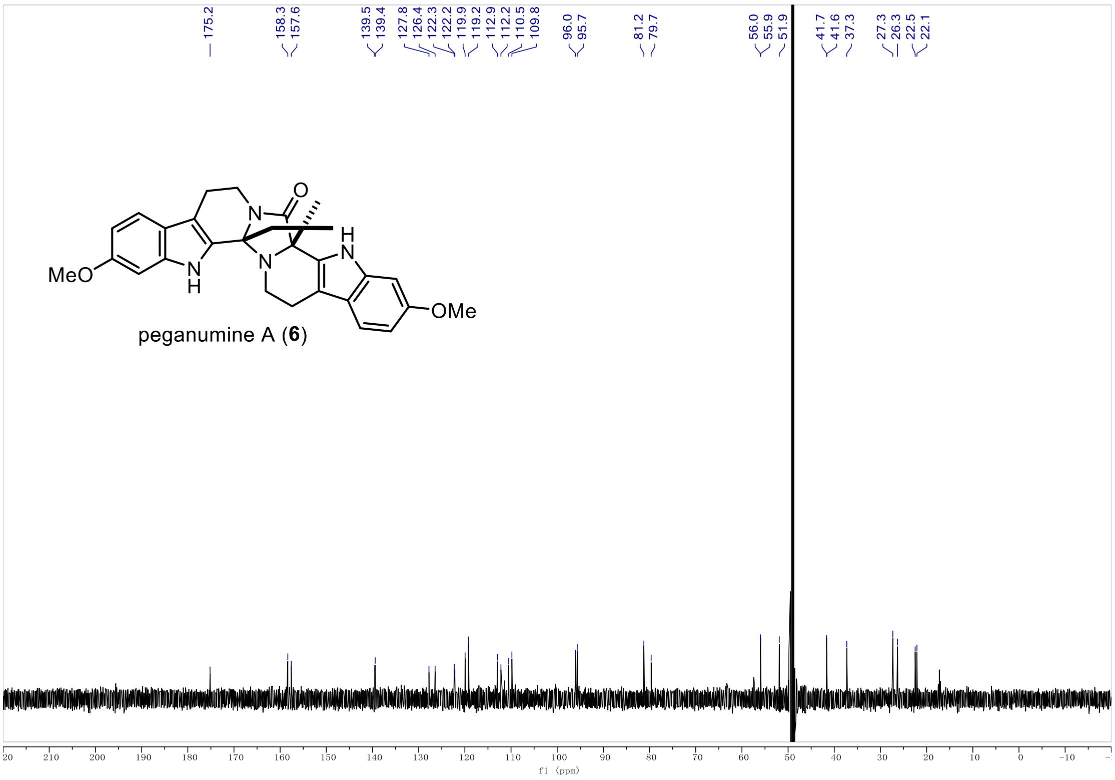


**$^{1}$H NMR** (500 MHz, ***$d_6$*-DMSO**) of peganumine A (**6**).

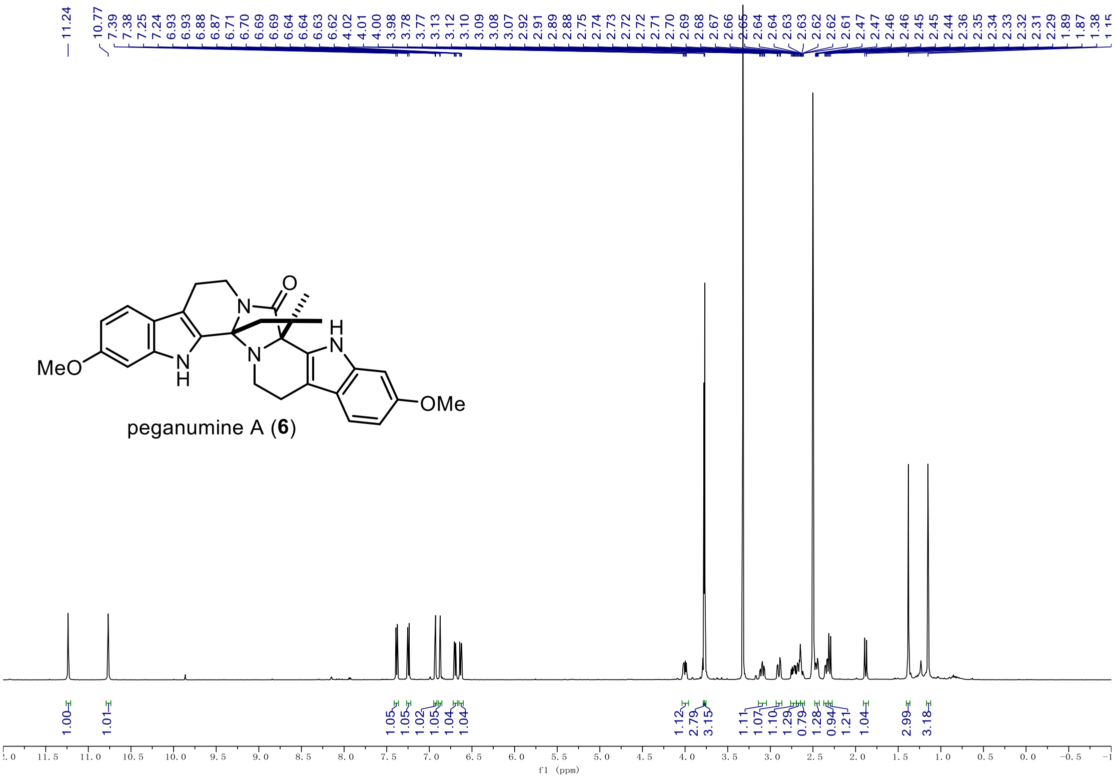

**$^{13}$C NMR** (126 MHz, ***d*₆-DMSO**) of peganumine A (**6**).

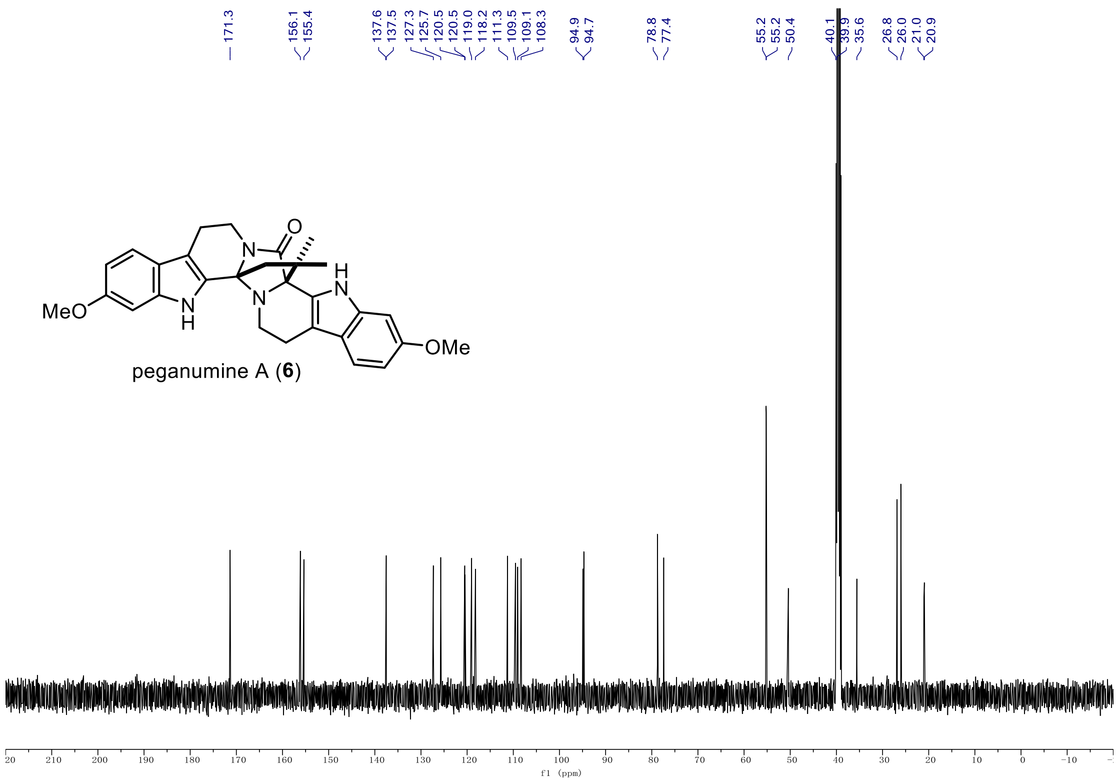


**$^{1}$H NMR** (500 MHz, $CDCl_3$) of **S21.**

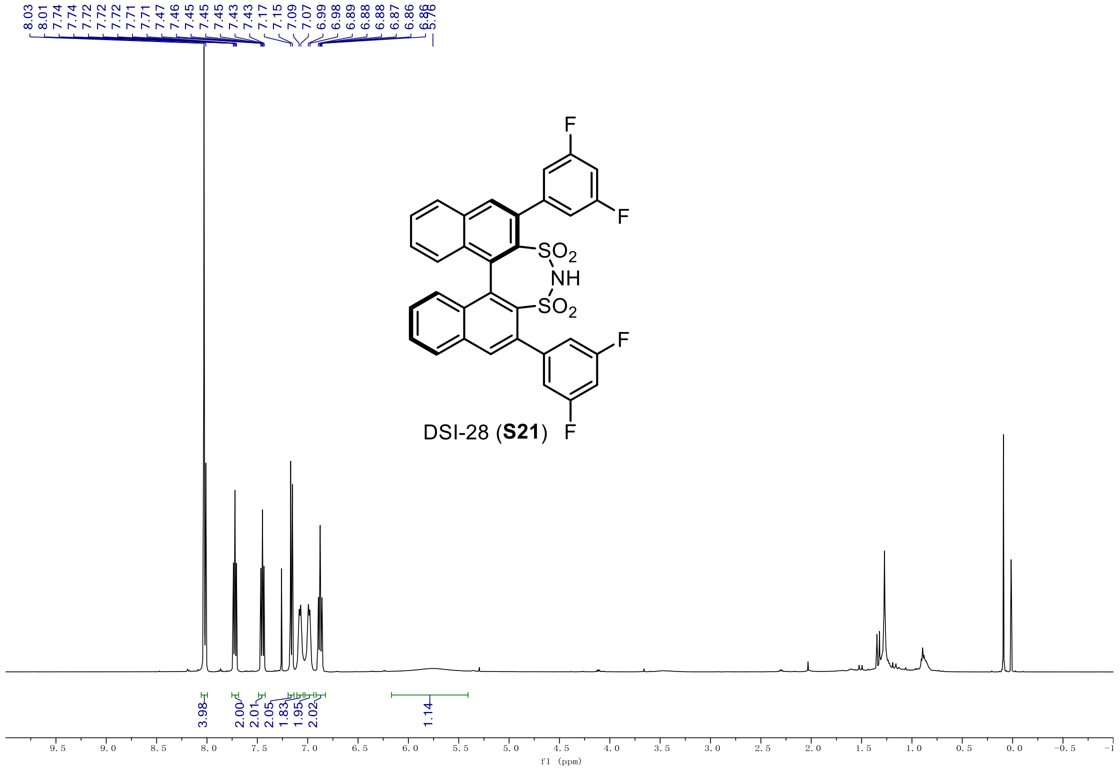

**$^{13}$C NMR** (126 MHz, $CDCl_3$) of **S21.**

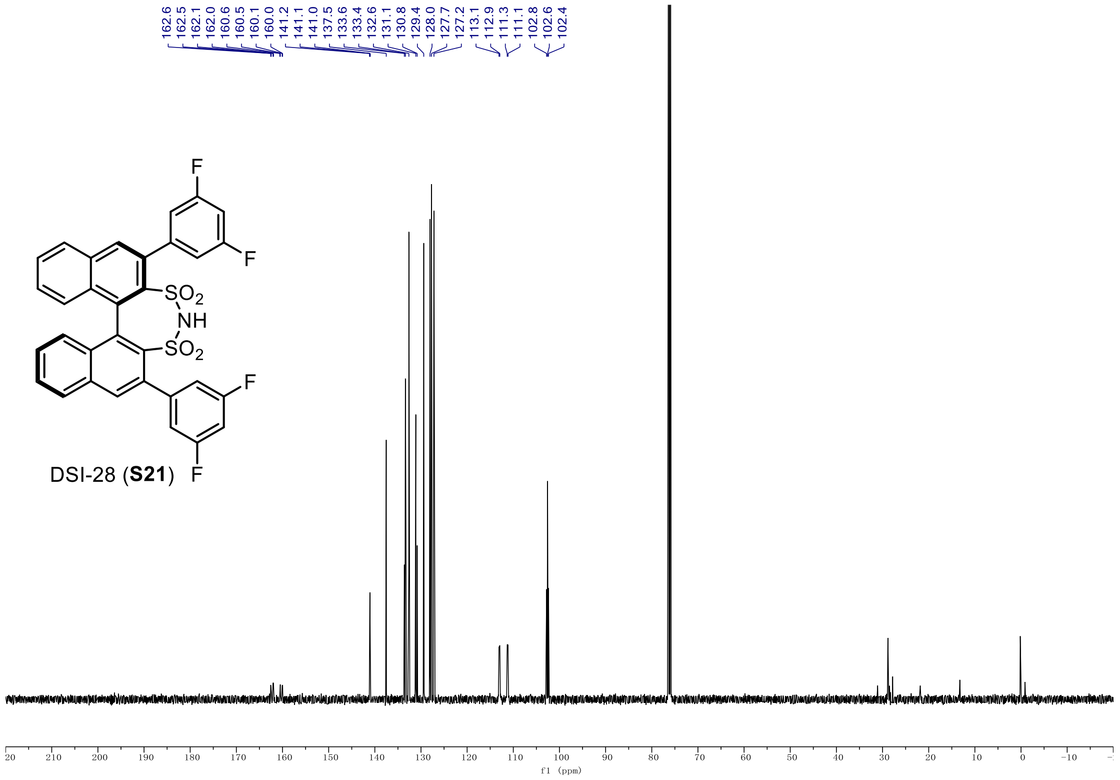


**$^{19}$F NMR** (471 MHz, $CDCl_3$) of **S21.**

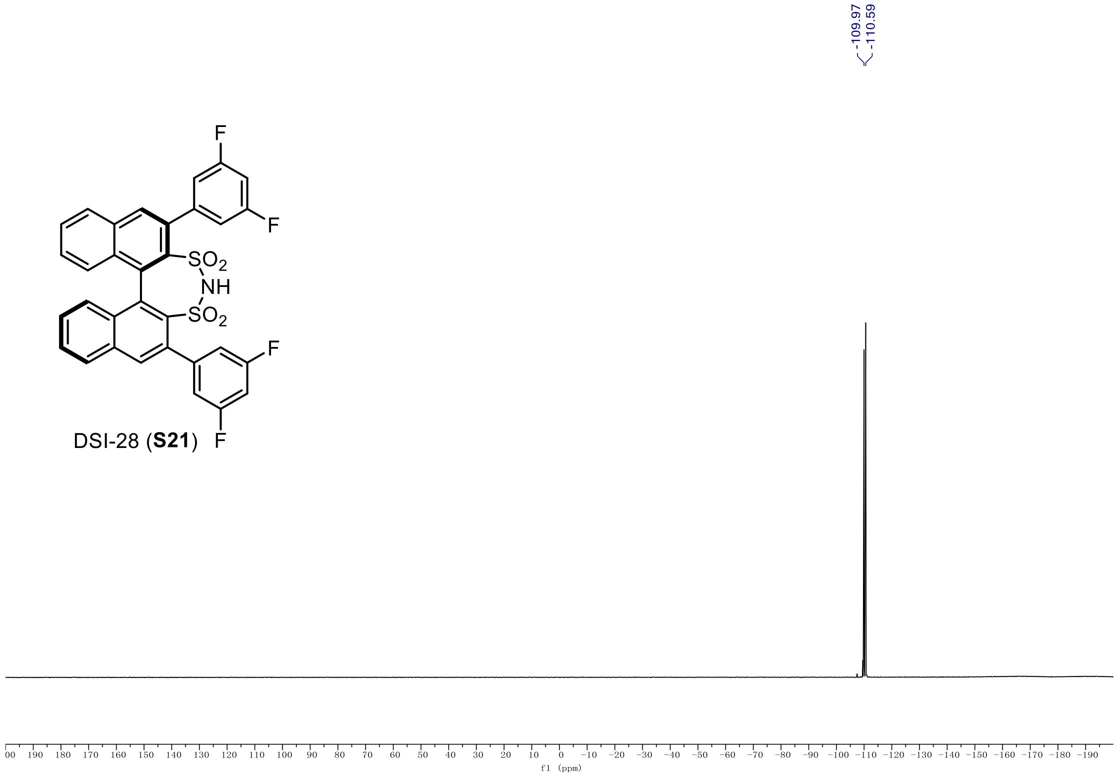

**$^{1}$H NMR** (500 MHz, $CDCl_3$) of **S22.**

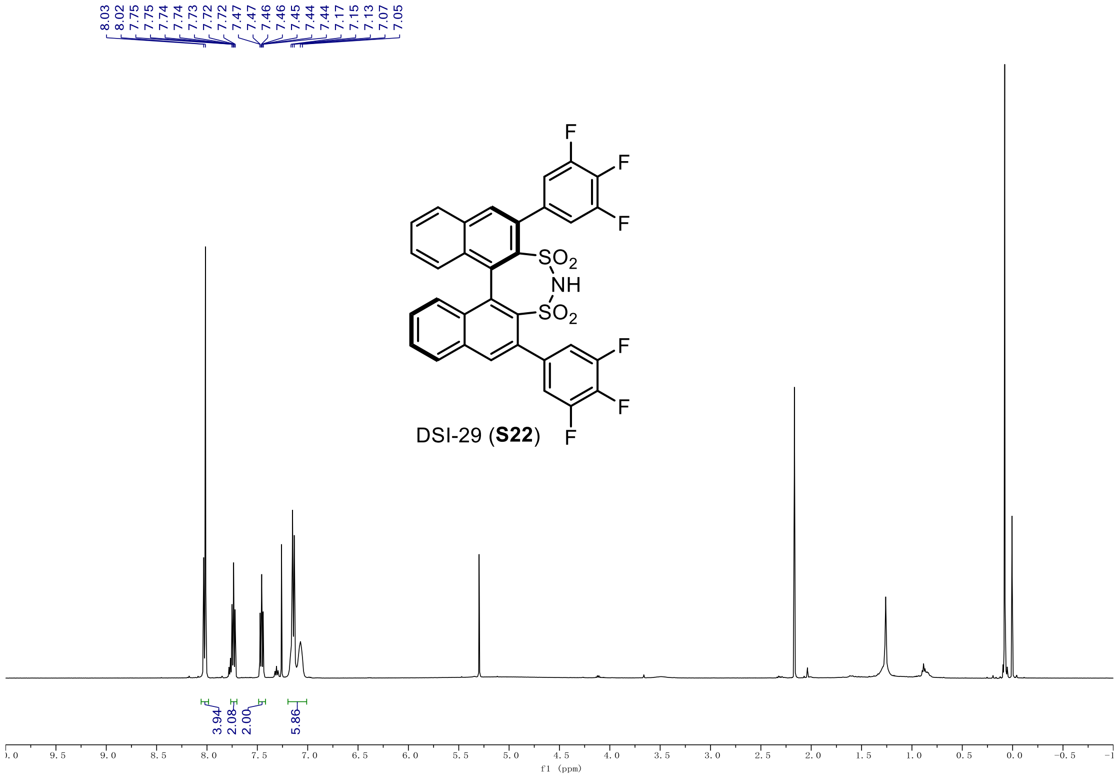


**$^{13}$C NMR** (126 MHz, $CDCl_3$) of **S22.**

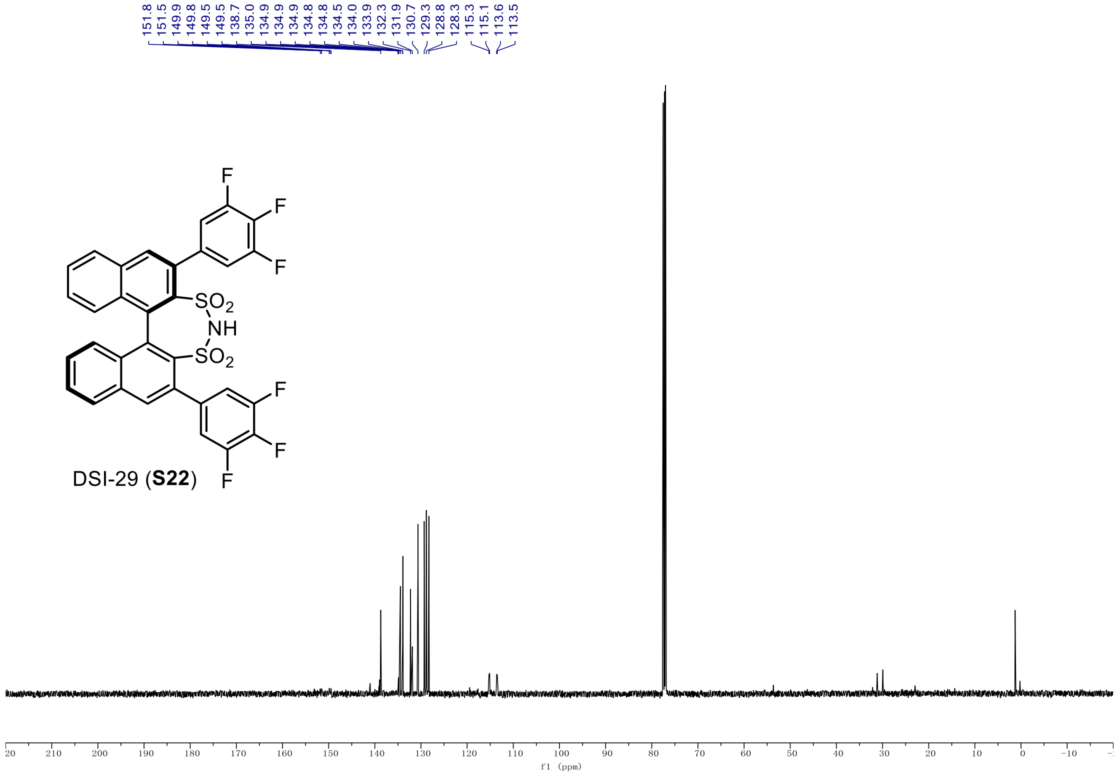

**$^{19}$F NMR** (471 MHz, $CDCl_3$) of **S22.**

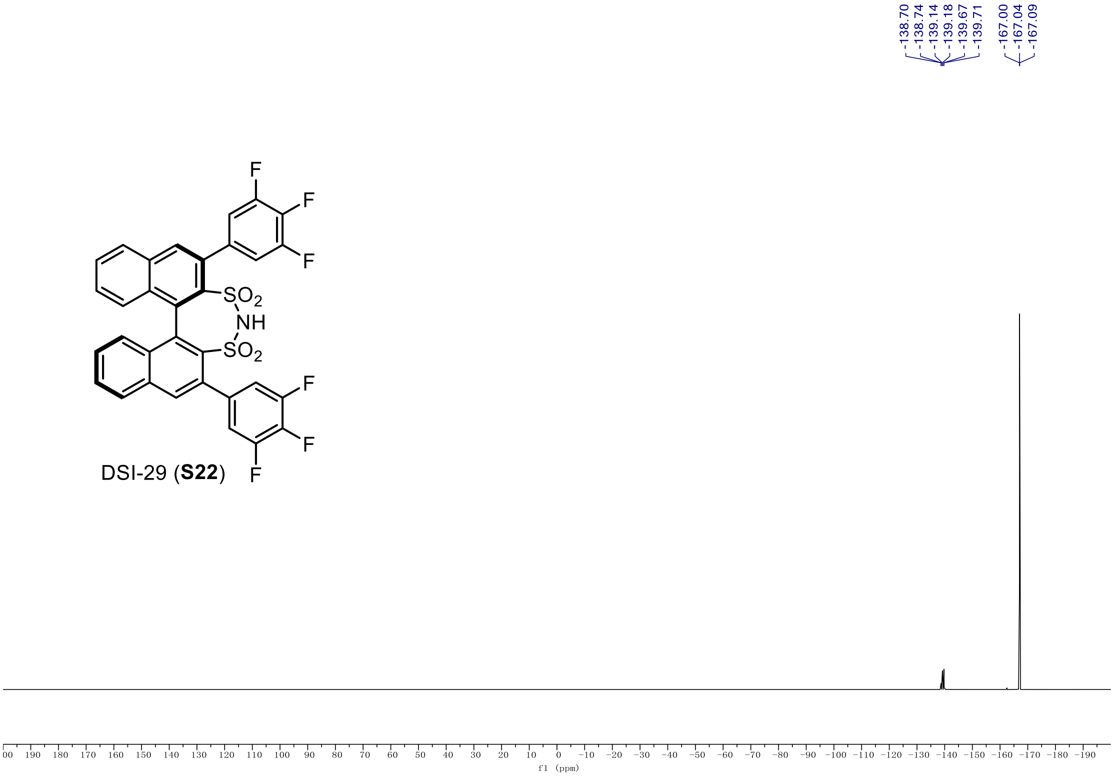


**$^{1}$H NMR** (500 MHz, $CD_2Cl_2$) of **S23.**

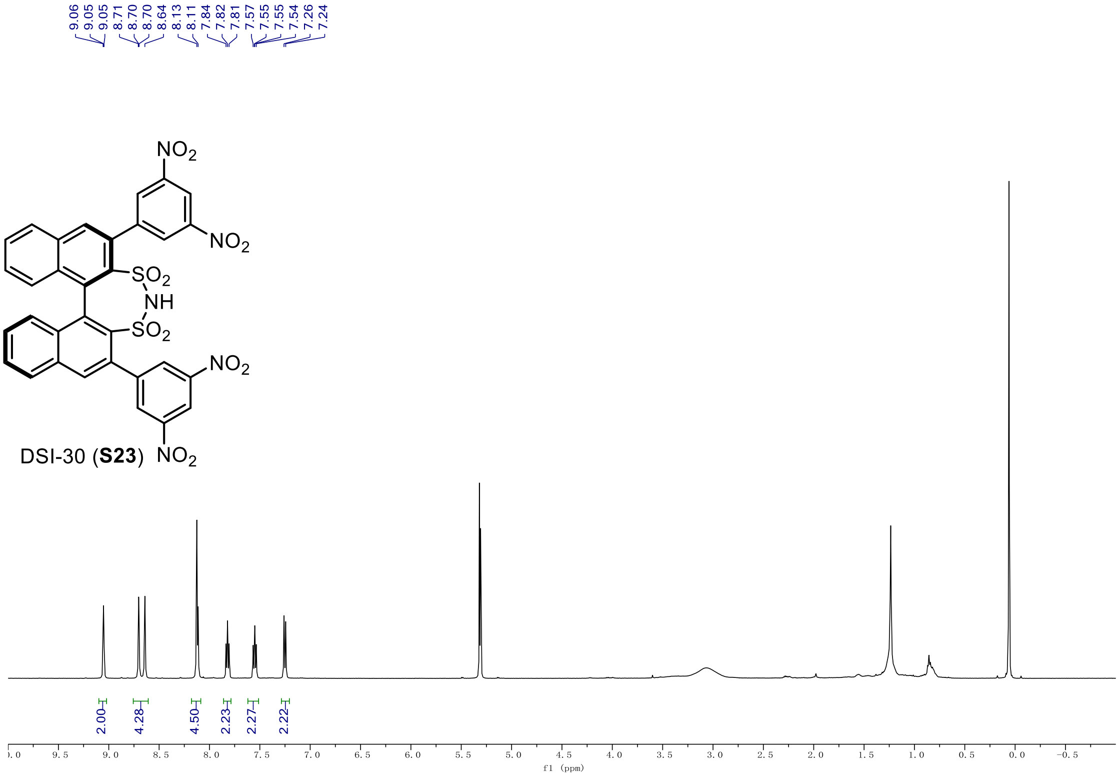

**$^{13}$C NMR** (126 MHz, $CD_2Cl_2$) of **S23.**

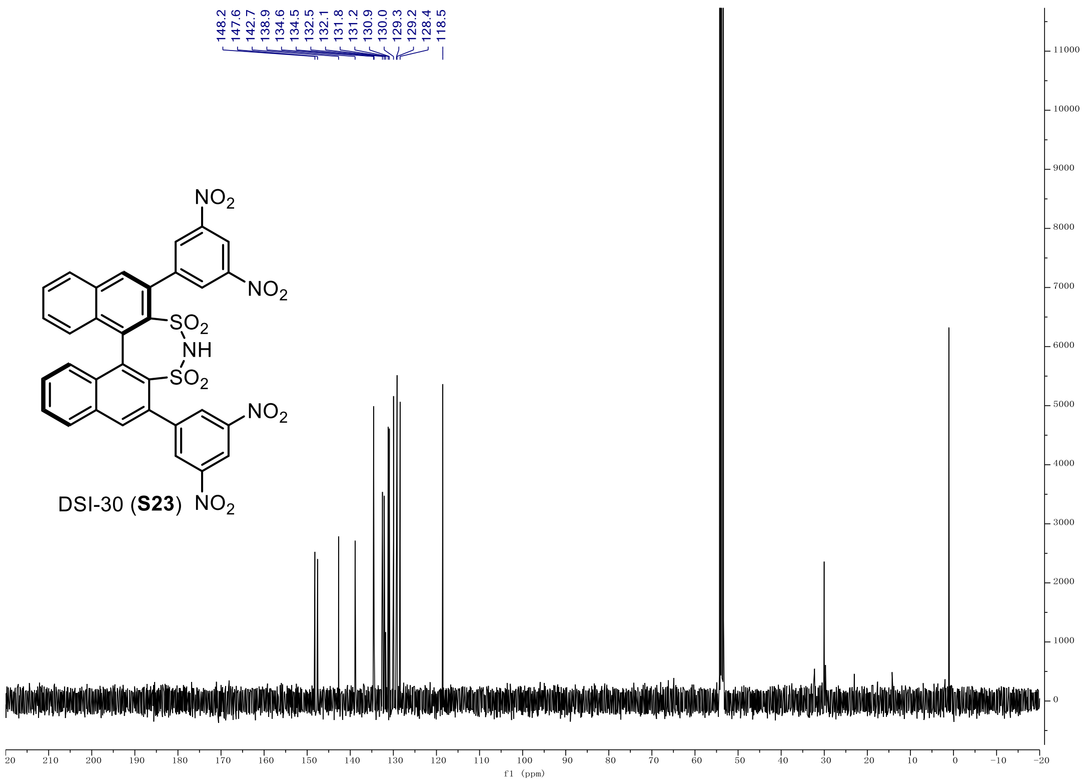


**$^{1}$H NMR** (500 MHz, $CDCl_3$) of **32.**

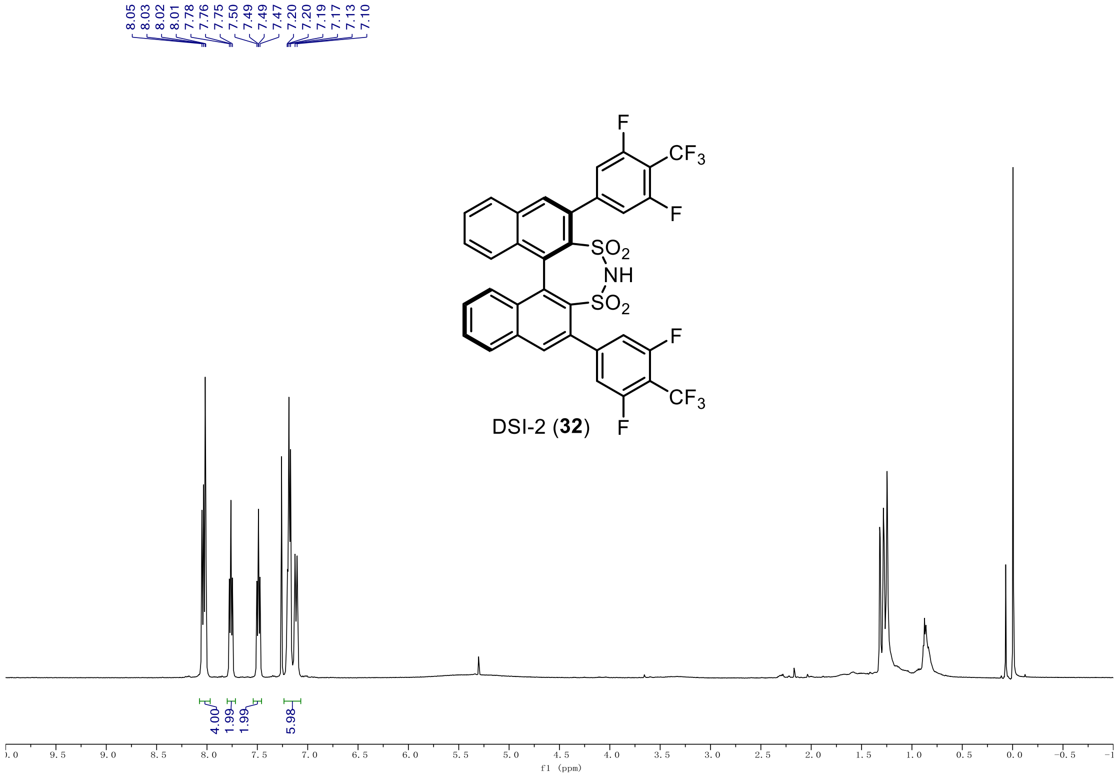

**$^{13}$C NMR** (126 MHz, $CDCl_3$) of **32.**

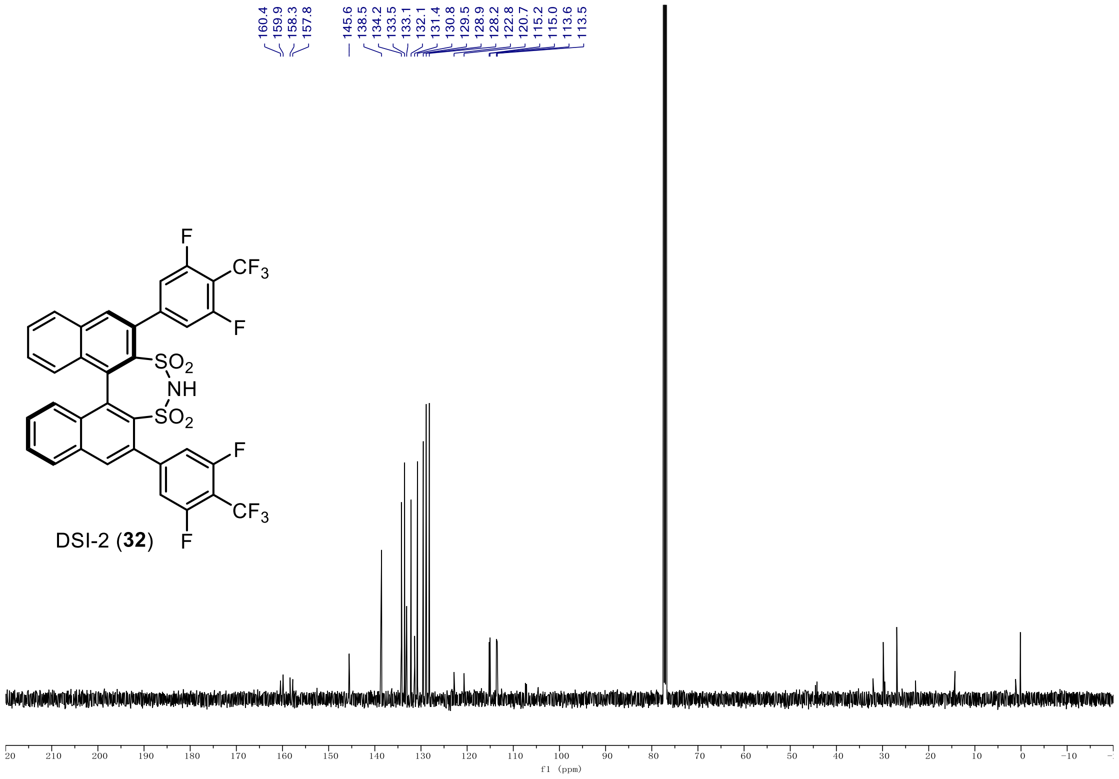


**$^{19}$F NMR** (471 MHz, $CDCl_3$) of **32.**

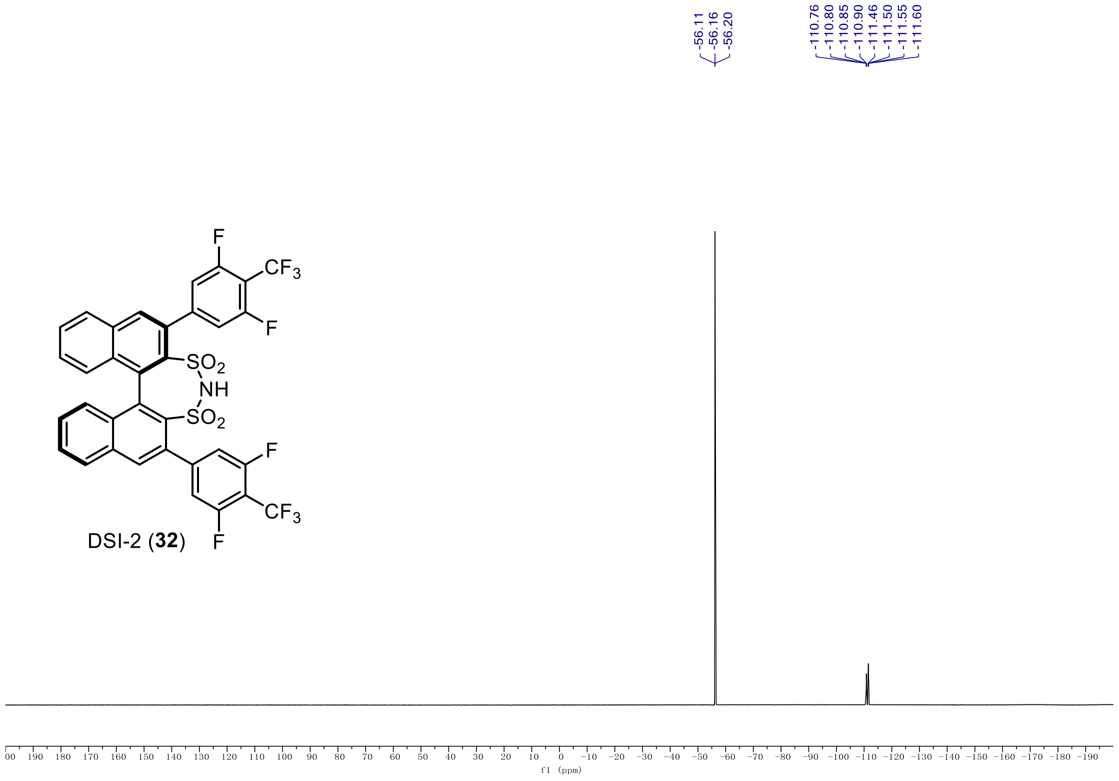

## $^{1}$H NMR (700 MHz, $CDCl_3$) of **35.**

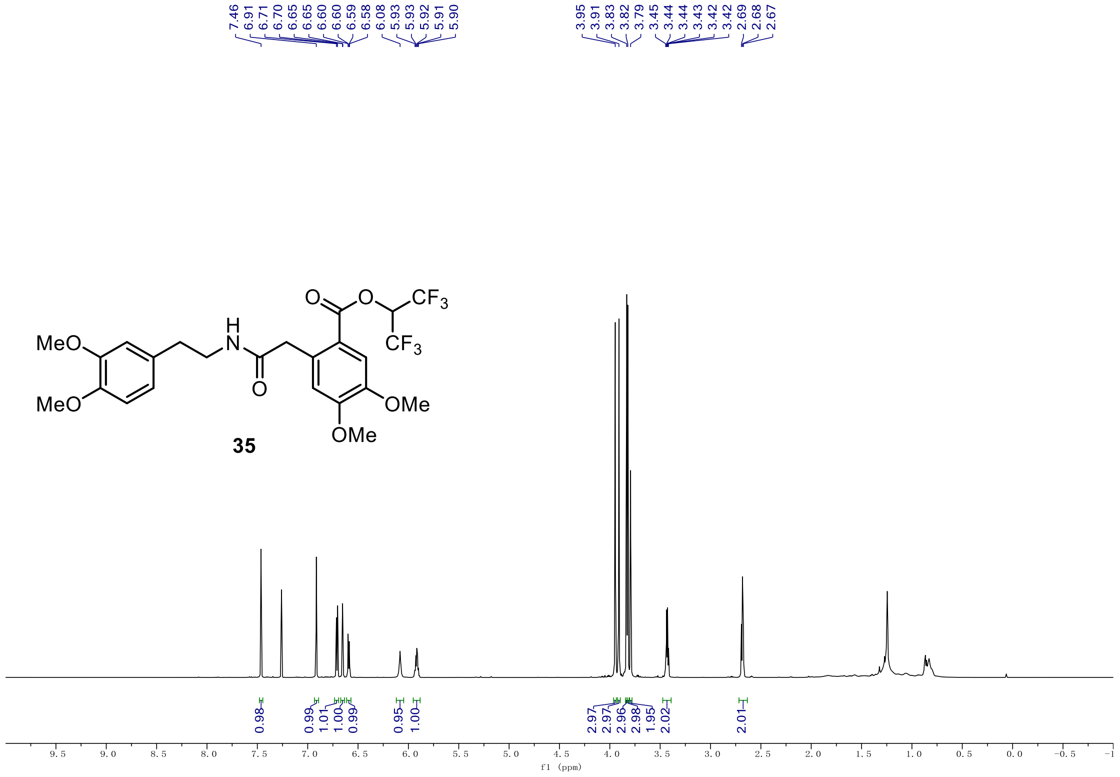


## $^{13}$C NMR (176 MHz, $CDCl_3$) of **35.**

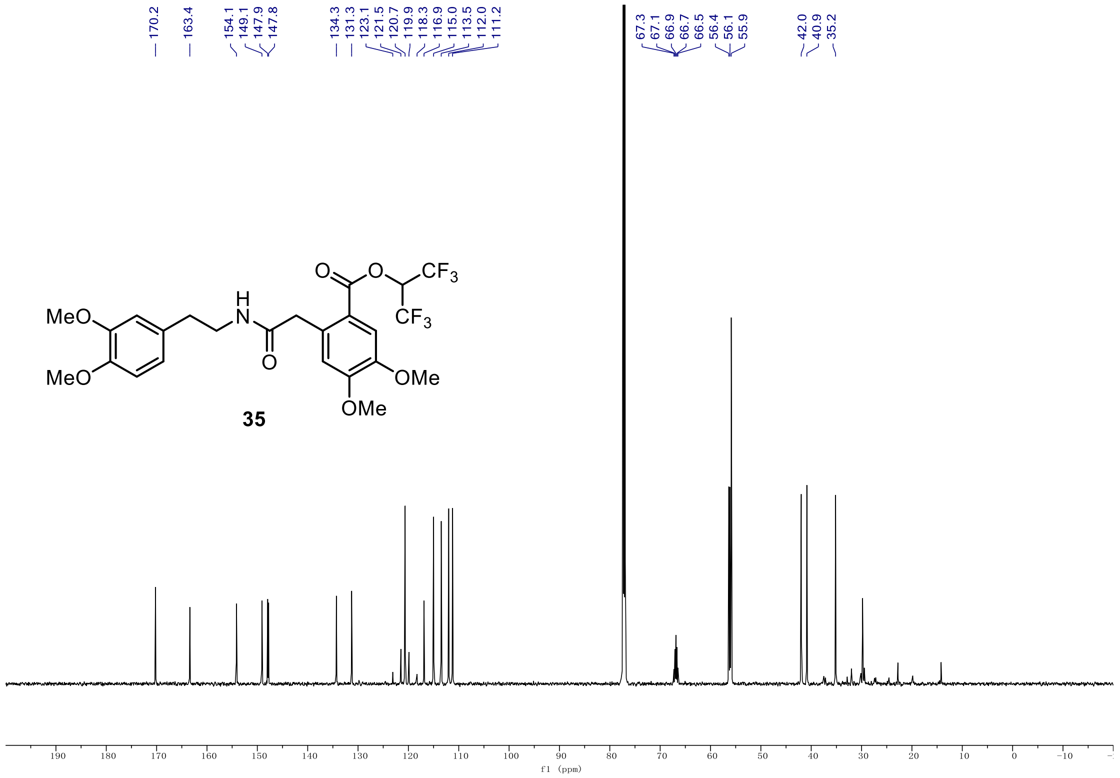

**$^{19}$F NMR** (565 MHz, $CDCl_3$) of **35.**

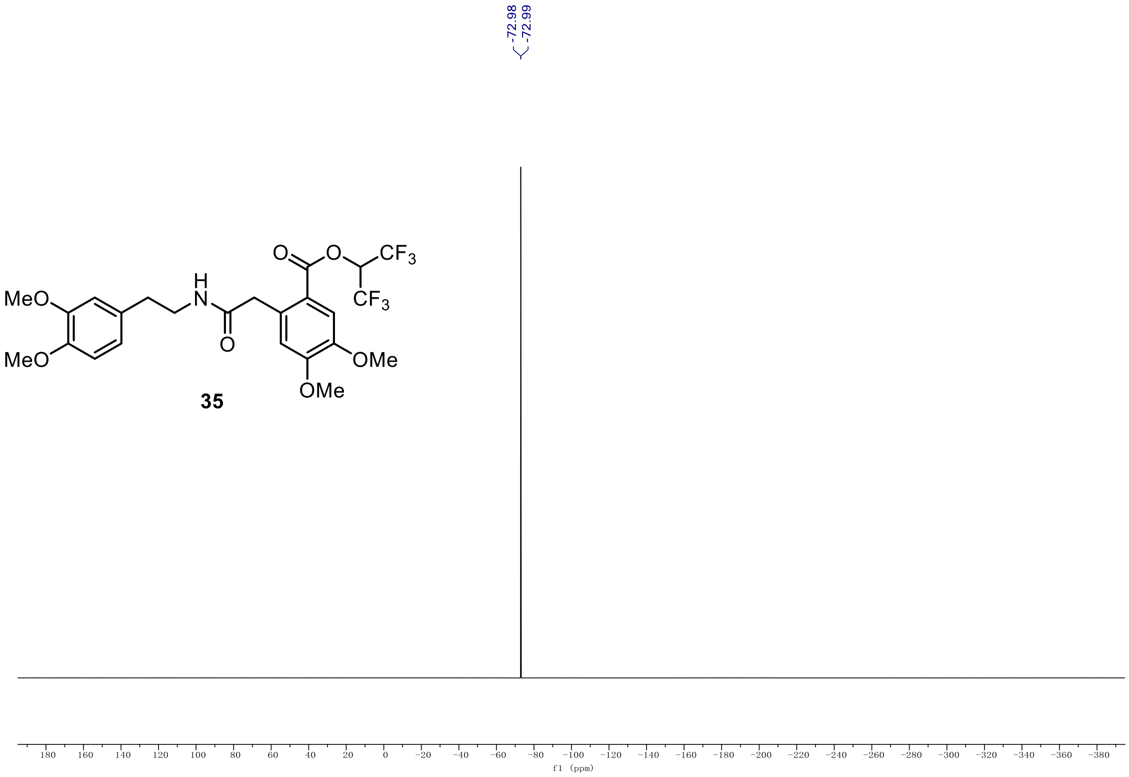


**$^{1}$H NMR** (700 MHz, $CDCl_3$) of **36.**

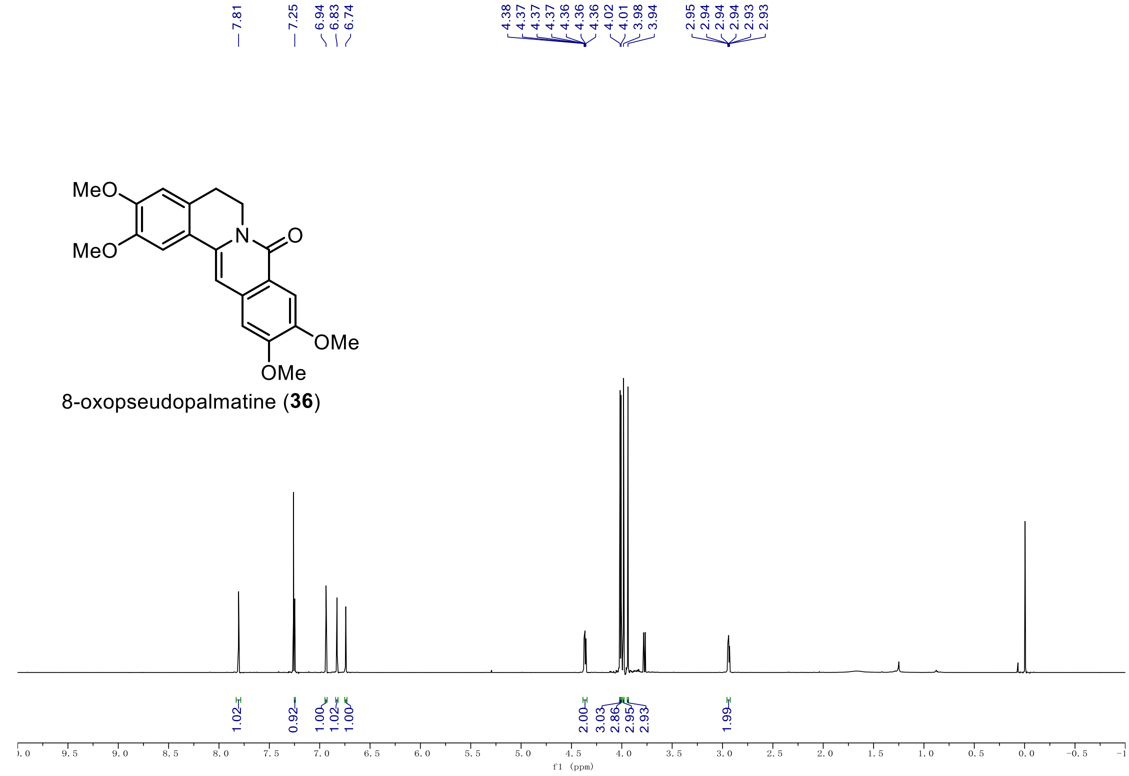

## $^{13}$C NMR (176 MHz, $CDCl_3$) of **36.**

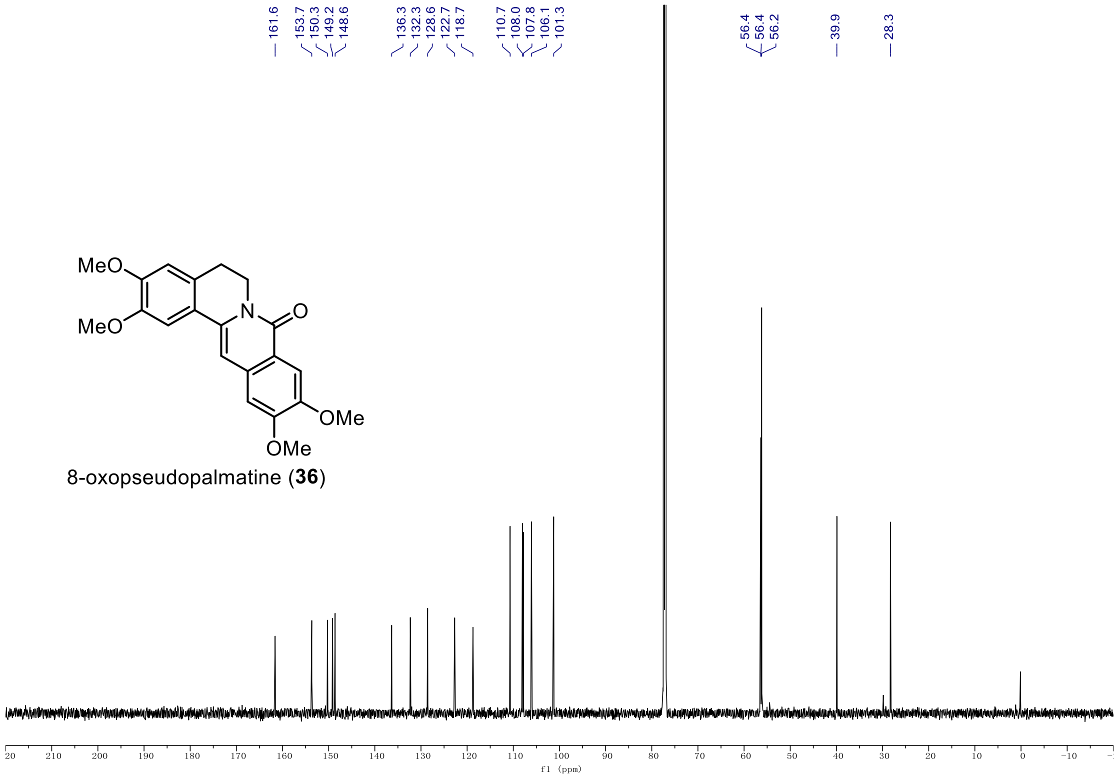


## $^{1}$H NMR (700 MHz, $CDCl_3$) of ilicifoline B **(5).**

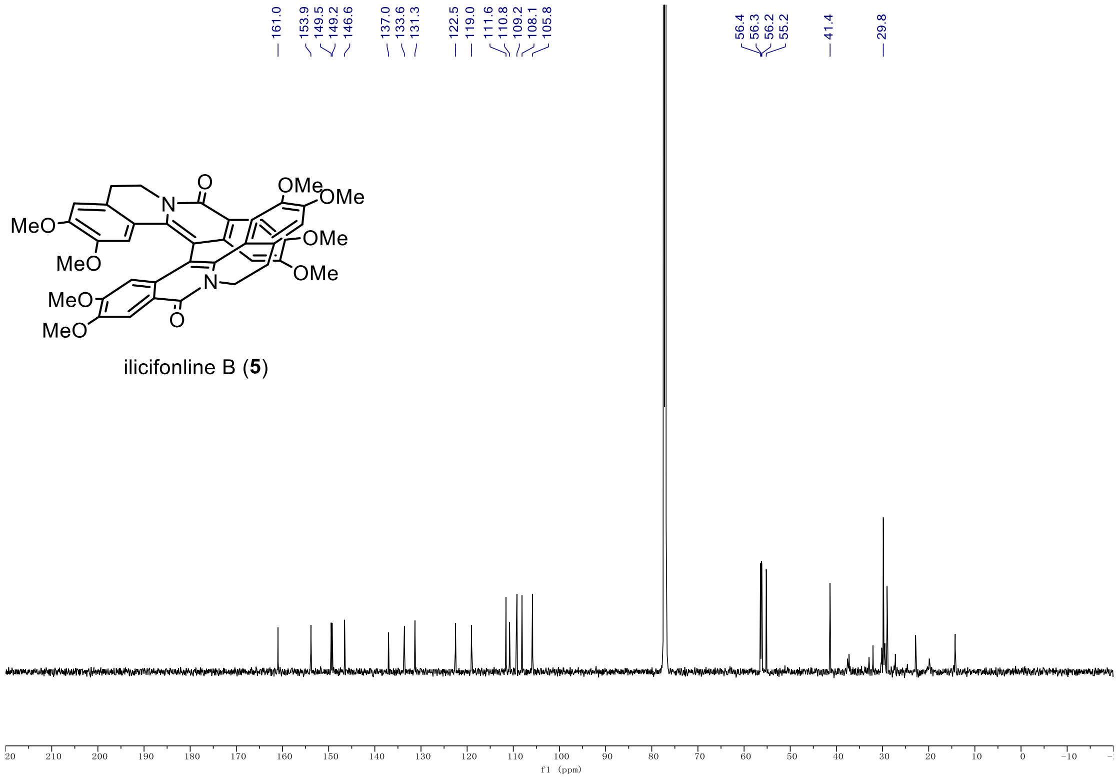

**$^{13}$C NMR** (176 MHz, $CDCl_3$) of ilicifoline B **(5).**

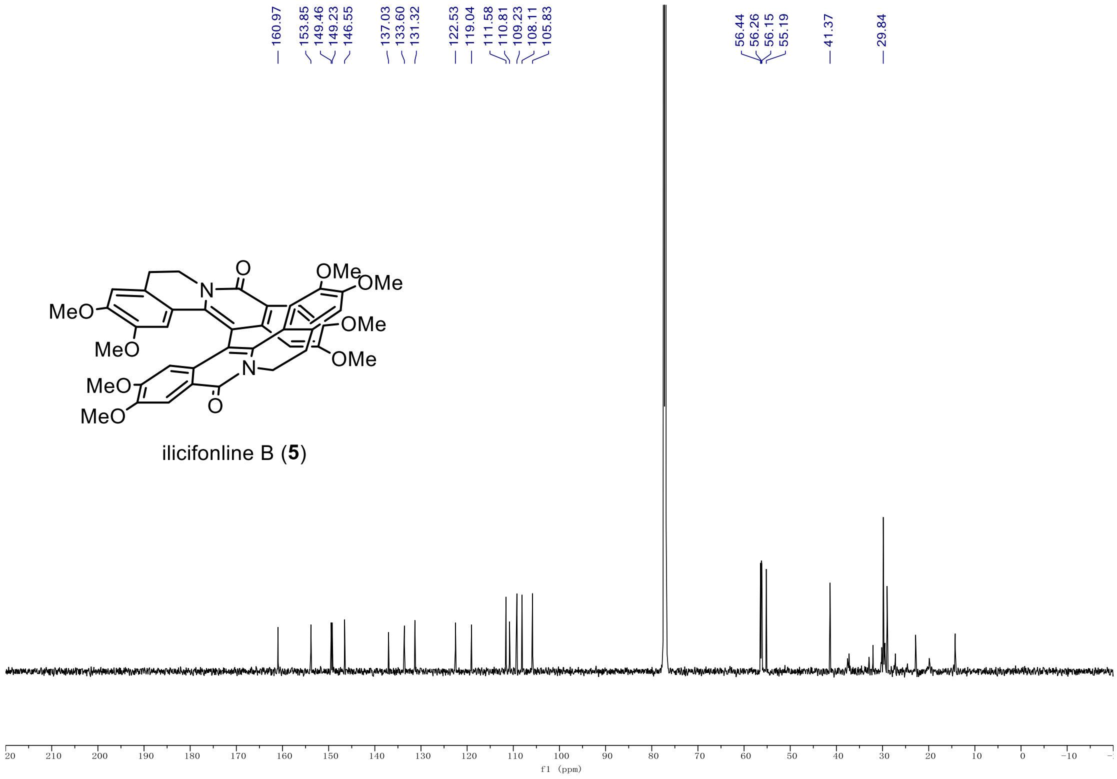


**$^{1}$H NMR** (500 MHz, $CD_3OD$) of **22.**

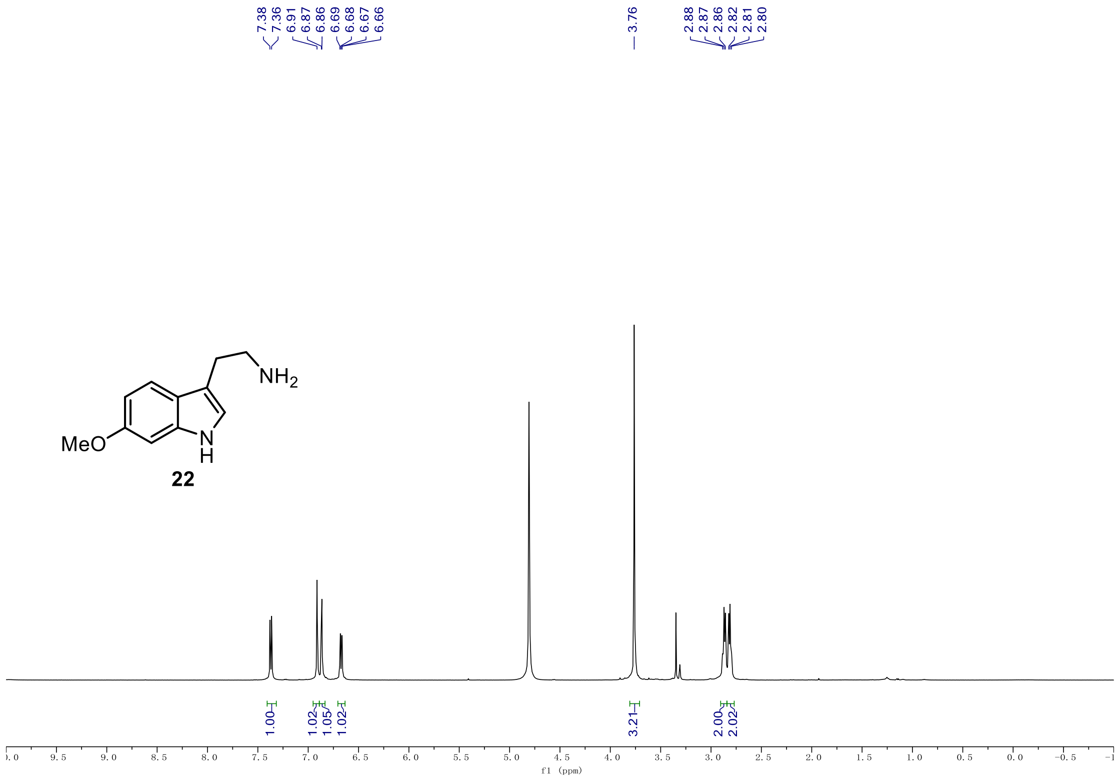

**$^{13}$C NMR** (126 MHz, $CD_3OD$) of **22.**

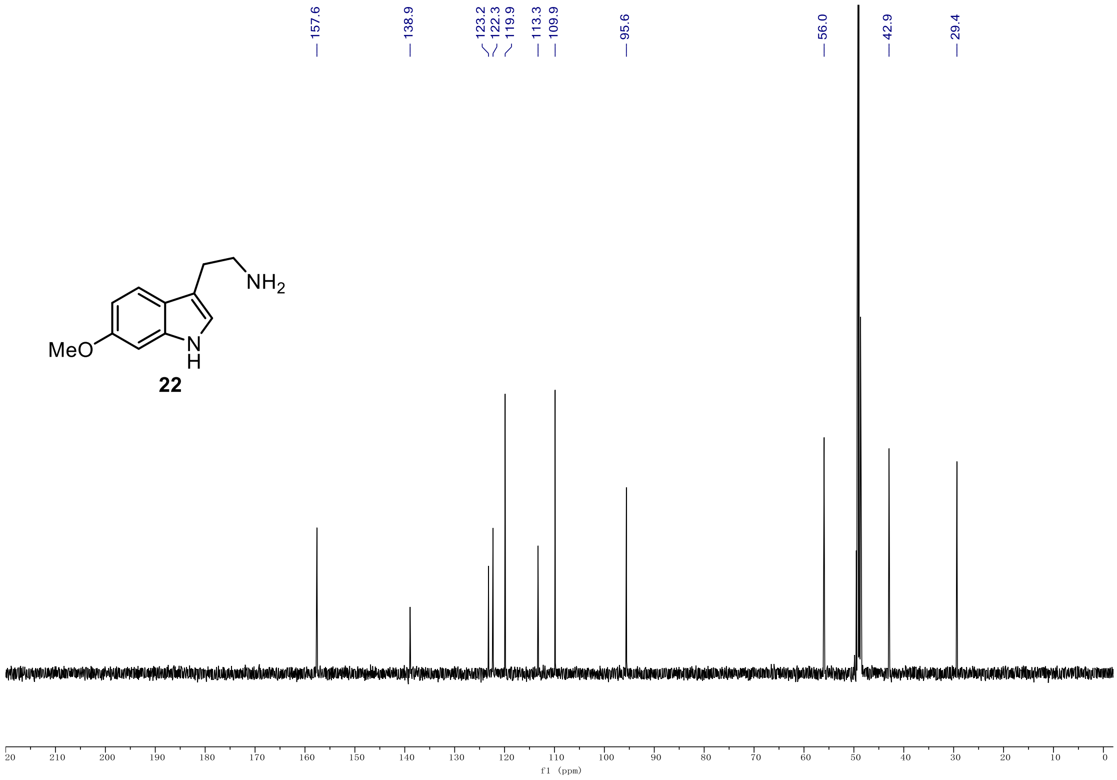


**$^{1}$H NMR** (500 MHz, $CD_3OD$) of **S36.**

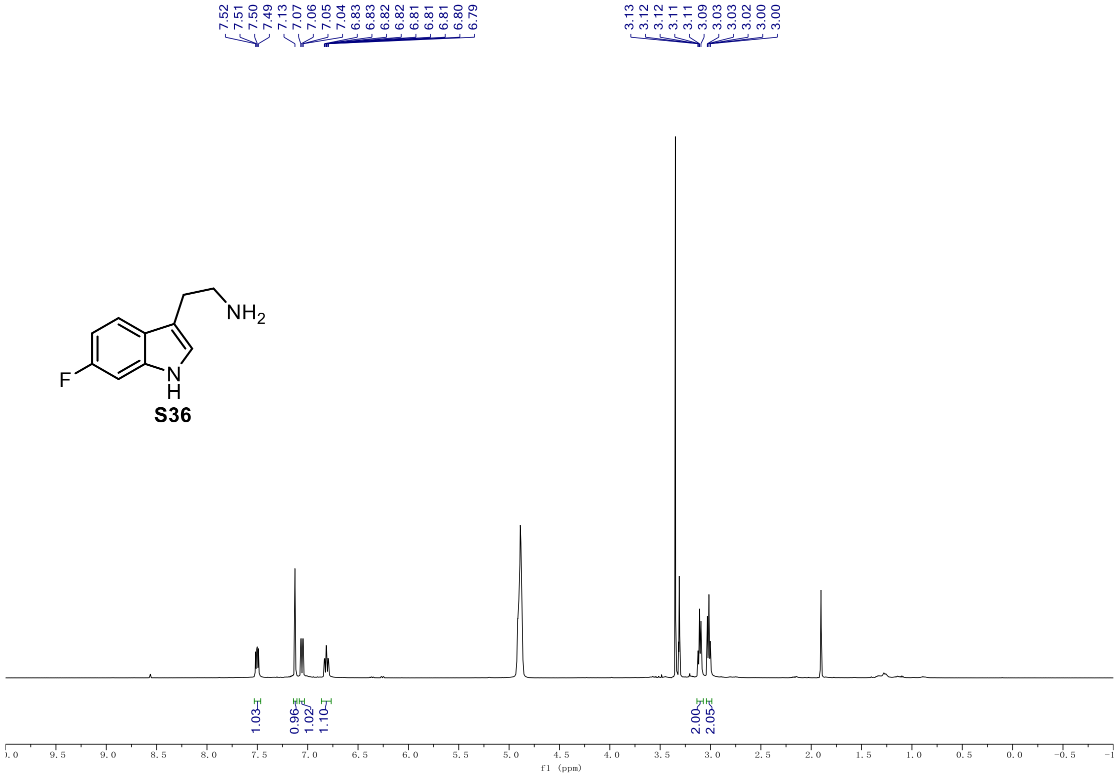

**$^{13}$C NMR** (126 MHz, $CD_3OD$) of **S36.**

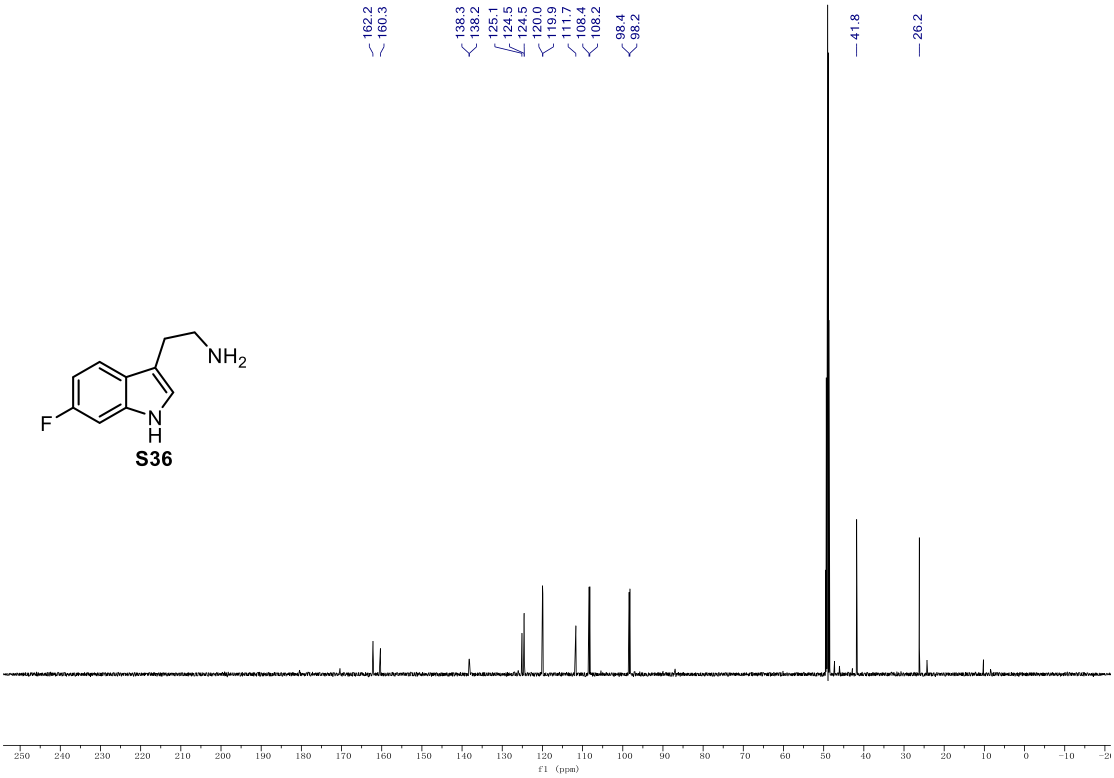


**$^{19}$F NMR** (471 MHz, $CD_3OD$) of **S36.**

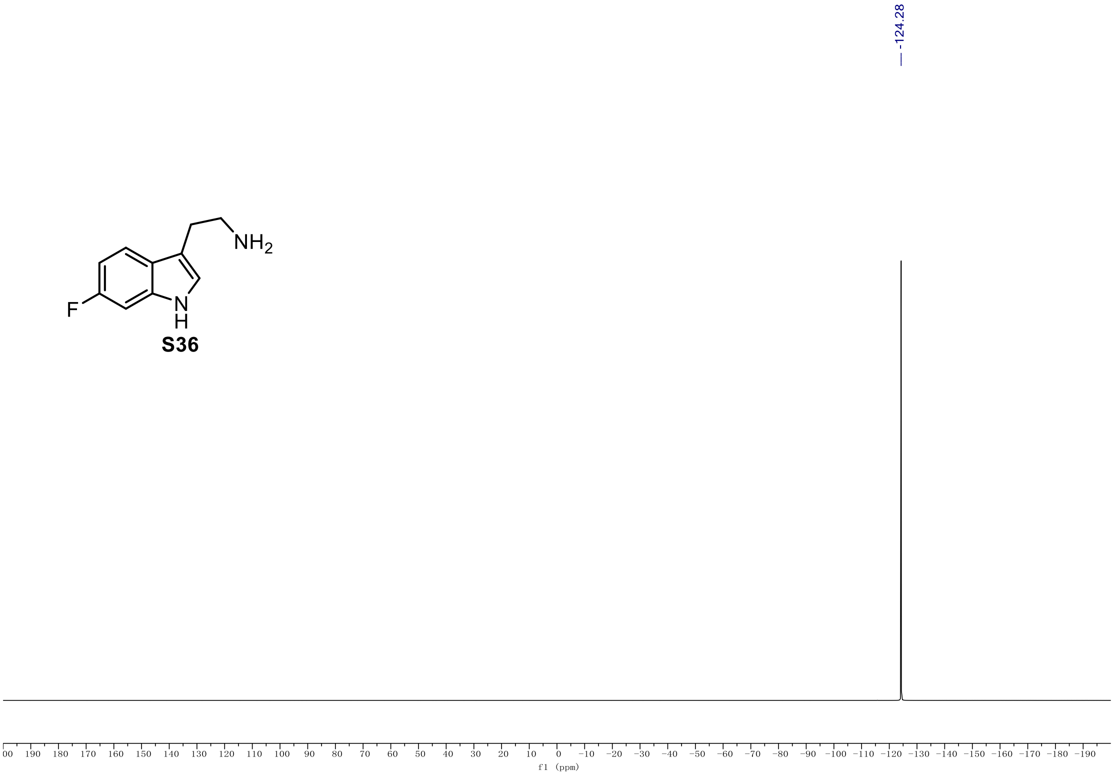

**$^1$H NMR** (500 MHz, $CD_3OD$) of **S37.**

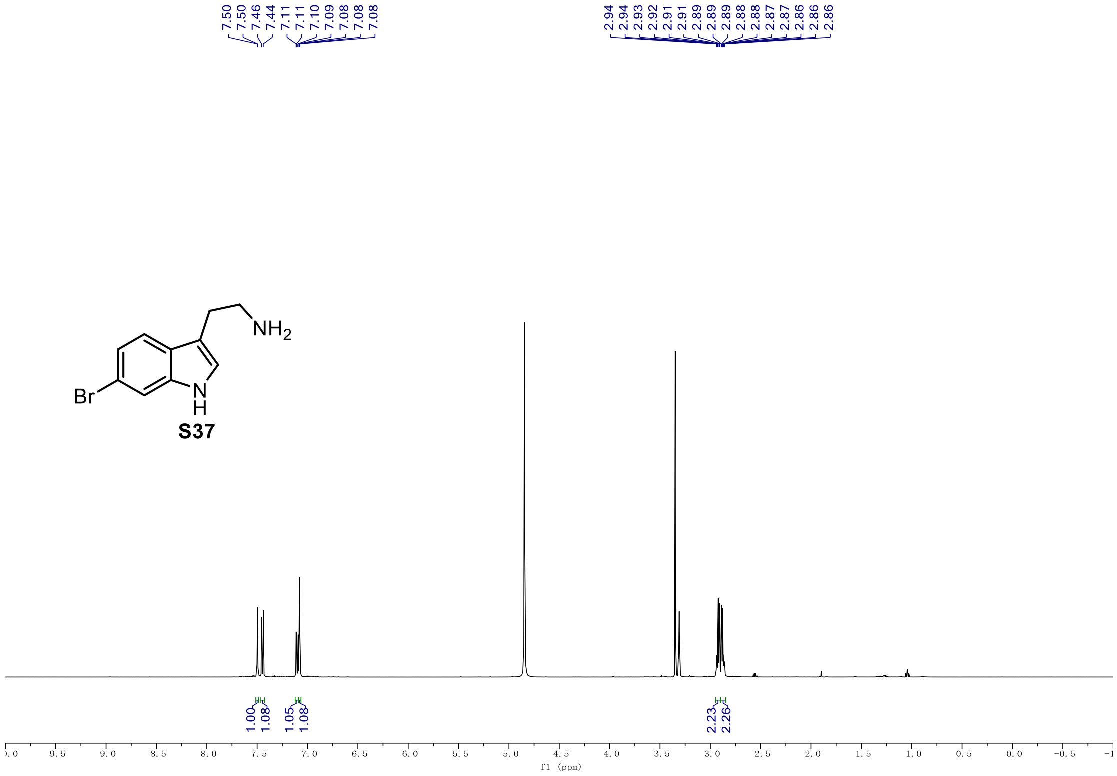


**$^{13}$C NMR** (126 MHz, $CD_3OD$) of **S37.**

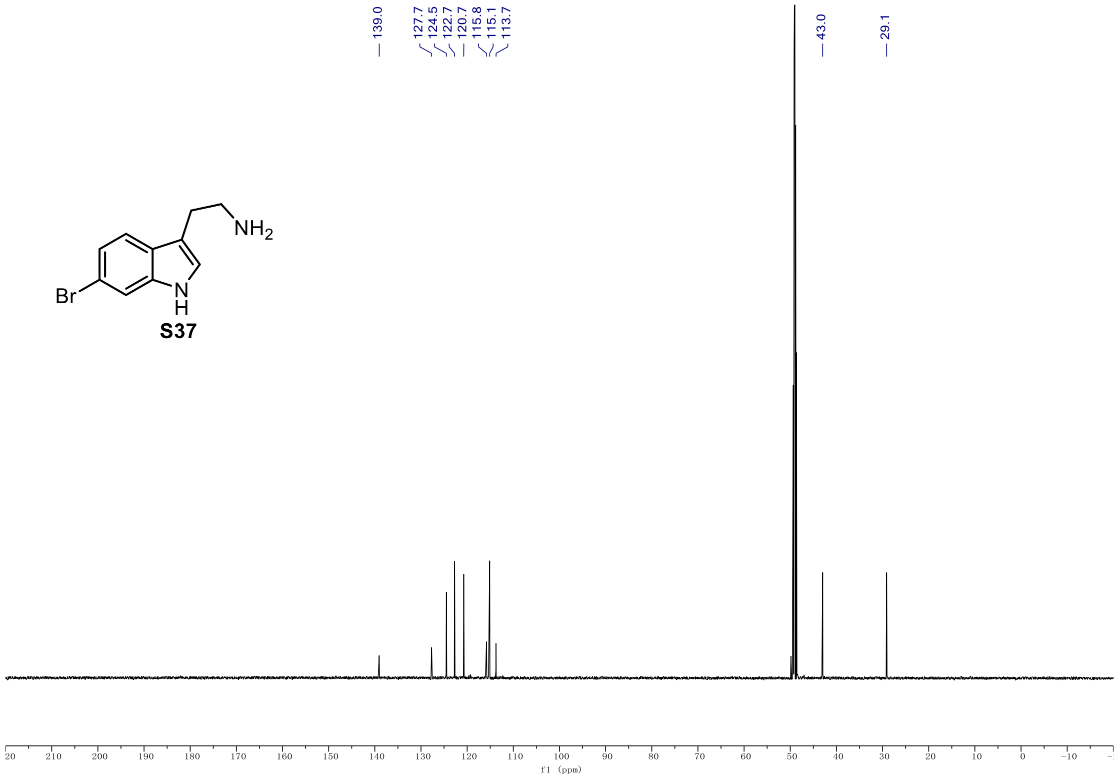

**$^{1}$H NMR** (500 MHz, $CD_3OD$: $CDCl_3$ (v/v = 1:1)) of **S38.**

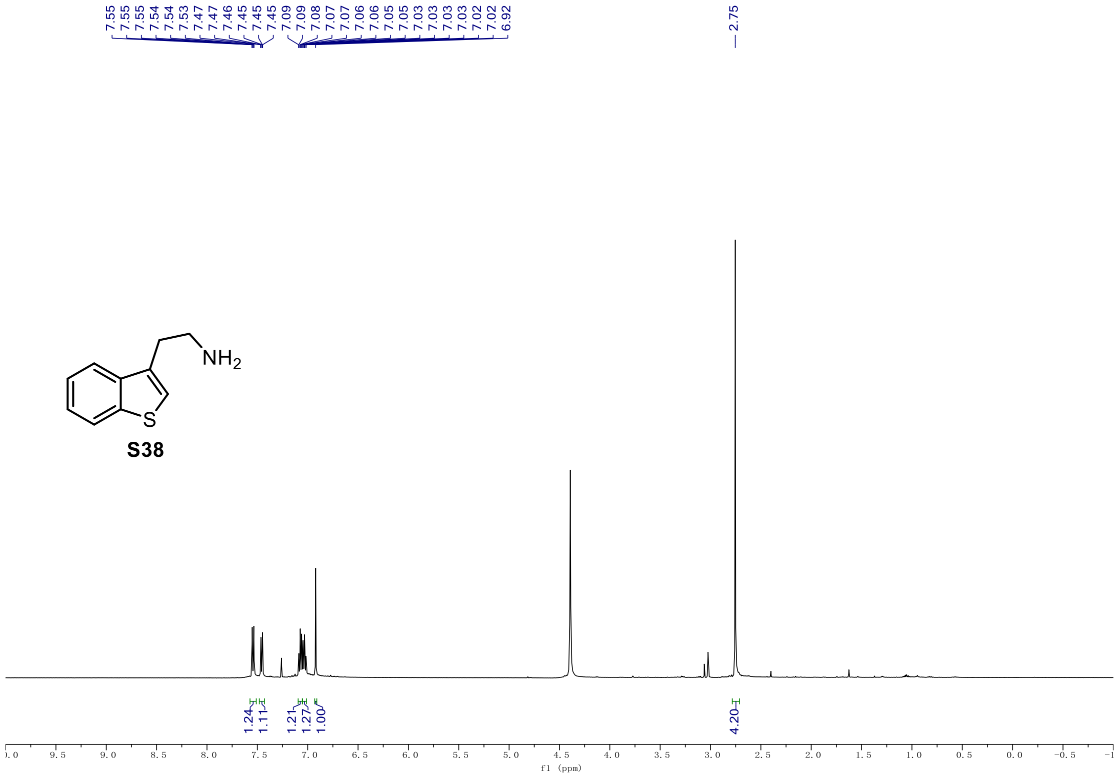


**$^{13}$C NMR** (126 MHz, $CD_3OD$: $CDCl_3$ (v/v = 1:1)) of **S38.**

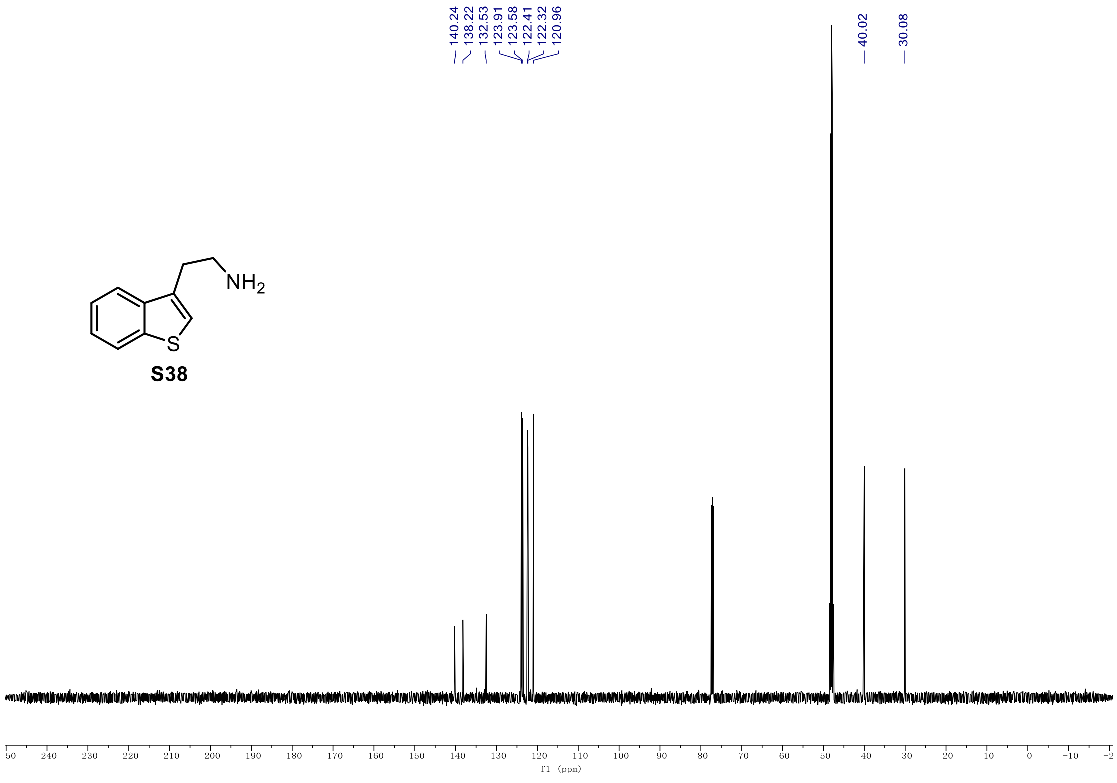

Supplement: Supplementary file 1 — Supplementary [file ANIE-60-13591-s001.pdf]
